# Insight into Medicinal Chemistry Behind Traditional Chinese Medicines: *p*-Hydroxybenzyl Alcohol-Derived Dimers and Trimers from *Gastrodia elata*

**DOI:** 10.1007/s13659-020-00258-w

**Published:** 2020-08-06

**Authors:** Yanan Wang, Min Zhang, Xue Zhou, Chengbo Xu, Chenggen Zhu, Yuhe Yuan, Naihong Chen, Yongchun Yang, Qinglan Guo, Jiangong Shi

**Affiliations:** State Key Laboratory of Bioactive Substance and Function of Natural Medicines, Institute of Materia Medica, Chinese Academy of Medical Sciences and Peking Union Medical College, Beijing, 100050 China

**Keywords:** Orchidaceae, *Gastrodia elata*, *p*-Hydroxybenzyl alcohol dimer, *p*-Hydroxybenzyl alcohol trimer, Gastrodibenzins, Gastrotribenzins, Medicinal chemistry behind Chinese medicines

## Abstract

From an aqueous extract of “tian ma” (the steamed and dried rhizomes of *Gastrodia elata*), ten new compounds gastrodibenzins A−D (**1**−**4**) and gastrotribenzins A−F (**5**−**10**), along with known analogues (**11**−**20**), having structure features coupling between two and three *p*-hydroxybenzyl-derived units via carbon- and/or ether-bonds, were isolated and characterized by spectroscopic data analysis. Meanwhile, the new compounds **5a**, **6a**, **8a**, **22**, and **23**, as well as the known derivatives **13a**, **14a**, **15**, **17**−**21**, **24**, **25**, and *p*-hydroxybenzyl aldehyde were isolated and identified from a refluxed aqueous solution of *p*-hydroxybenzyl alcohol. Methylation of **5a** and **6a** in methanol and ethylation of **6a**, **8a**, **13a**, and **14a** in ethanol produced **5** and **6** and **7**, **8**, **13**, and **14**, respectively. using ultra-performance liquid chromatography high-resolution electrospray ionization mass spectrometry (UPLC-HRESIMS) analysis of the refluxed solutions of *p*-hydroxybenzyl alcohol and the refluxed extracts of the fresh *G. elata* rhizome and “tian ma” extracts indicated consistent production and variation of the dimeric and trimeric derivatives of *p*-hydroxybenzyl alcohol upon extracting solvents and refluxing time. In various assays, the dimeric and trimeric derivatives showed more potent activities than *p*-hydroxybenzyl alcohol itself and gastrodin, which are the main known active constituents of “tian ma”. These results revealed for the first time that the more effective dimers and trimers can be produced through condensation of the co-occurring *p*-hydroxybenzyl alcohol during processing and decocting of the *G. elata* rhizomes, demonstrating insights into medicinal chemistry behind application protocols of traditional Chinese medicines.

**Electronic supplementary material:**

The online version of this article (10.1007/s13659-020-00258-w) contains supplementary material, which is available to authorized users.

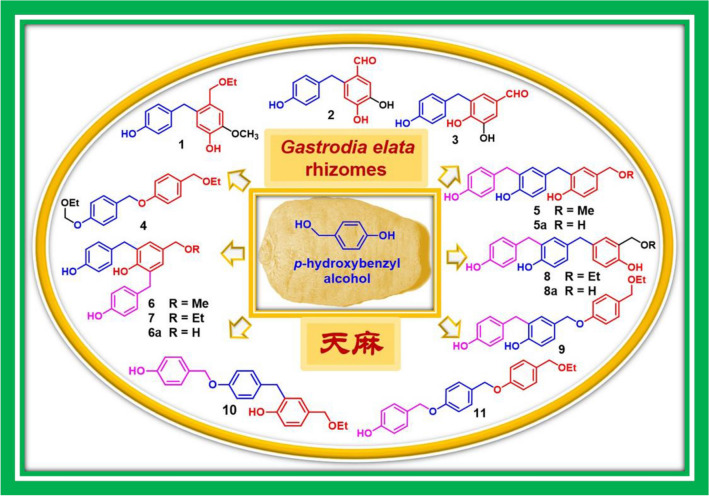

## Introduction

The steamed and dried rhizomes of *Gastrodia elata* Blume (Orchidaceae) is a precious and important tonic herbal medicine, named “tian ma” in Chinese, having health benefits enhancing strength and virility as well as improving memory and blood circulation [1]. Since antiquity this traditional medicine is mainly used for the treatment of various neuralgic and nervous disorders in China [2]. The plant *G. elata* is an endangered holomycotrophic species living on several symbiotic mycorrhizal fungi at specific stages of its life cycle [3]. To satisfy medicinal utilization and to protect the wildly endangered species and ecological environment, starting in the late 1950′s, tremendous efforts were made and succeeded in agricultural cultivation of this plant by Chinese scientists [3, 4]. Meanwhile, considerable chemical and pharmacological studies of the raw material and the processed “tian ma” led to isolation and identification of around 100 constituents with diverse chemical structures and biological activities from extracts of this herbal medicine [2, 5–8]. Most the constituents feature characteristic structures deriving from or modified by *p*-hydroxybenzyl alcohol [2, 5–8]. Notably, vanillyl alcohol and vanillin exhibited antiepileptic and anticonvulsant activities similar to that of the water extract of *G. elata* rhizomes, which promoted the clinic application of vanillin as an antiepileptic drug in China [9–13] ^3^. The major component of “tian ma”, gastrodin and its synthetic acetate were developed for the treatment of neurasthenia, neurasthenic syndrome, angioneurotic headache, and insomnia [14–20]. Recent pharmacological studies showed that 4-hydroxybenzyl analogues [21–36] and gastrodin [37–42] possessed various in vivo and in vitro neurological activities. Gastrodin was found also to have effects on cardiac hypertrophy and fibrosis [43] and vasodilation [44] as well as on anti-cancer immune response [45]. However, some studies showed that after removing of gastrodin the aqueous extract of “tian ma” retained the anti-hypoxia, sedative, hypnotic and anti-inflammatory effects and that at high dosages gastrodin did not exhibit the effects [46, 47]. The occurrence of antiepileptic vanillin in “tian ma” was questioned until 2006 [48, 49]. Meanwhile, new constituents from “tian ma” and their bioactivities were frequently discovered [2, 5–8].

**Fig. 1:**
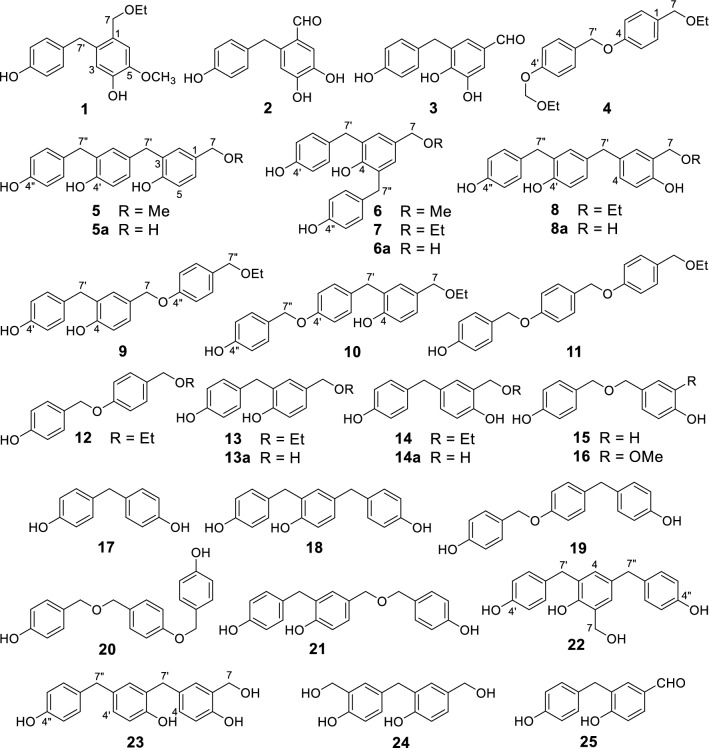
Structures of compounds **1**−**25**

According to the theory of traditional Chinese medicines (TCM), the drug materials are commonly processed and/or decocted to detoxify and/or to enhance effects of the herbal medicines. Chemical reaction must take place during processing and/or decocting to alter the chemical compositions of final decoctions used for the treatment of patients. This suggests that the aqueous decoction of the processed drug material might contain more benefit components for patients. Thus, there are important secrets of medicinal chemistry hiding behind processing and/or decocting protocols in TCM though these are yet to be confirmed in many cases including “tian ma” [50–58]. Because the previous phytochemical studies of “tian ma” were performed mostly by extracting the drug material with ethanol or methanol [2, 5–8], the extracting protocol completely differed from that of conventional application by decocting with water. Therefore, an aqueous extract of “tian ma” (the steamed and dried *G. elata* rhizomes [50–58]) was investigated as part of our project to investigate chemical diversity and biological activities of several commonly used TCM [59–70]. Previously we reported 27 new and 40 known chemical constituents of the aqueous extract, along with their bioassays and pharmacological activities [71–79]. Especially we found that several *p*-hydroxybenzyl-modified gastrodins from the extract could be produced from a coupling reaction of the co-occurring *p*-hydroxybenzyl alcohol and gastrodin in H_2_O under refluxing [80]. This unraveled production of the new components during processing and decocting of “tian ma” in the classical application protocol. A further investigation resulted in characterization of ten new compounds gastrodibenzins A−D (**1**−**4**) and gastrotribenzins A−F (**5**−**10**) as well as ten known derivatives (**11**−**20**) (Fig. 1) from the remaining subfractions of the extract. Viewing the structures of **4**−**20**, these compounds may be derived from condensations of two or three *p*-hydroxybenzyl alcohol units at different positions via carbon- and/or ether-bonds, followed by etherification with the solvents MeOH or EtOH (**4**−**14**). With the speculation, a refluxed H_2_O solution of *p*-hydroxybenzyl alcohol was isolated to yield **5a**, **6a**, **8a**, **13a**, **14a**, **15**, **17**−**19**, **21**−**25** (Fig. 1), and *p*-hydroxybenzaldehyde. UPLC-HRESIMS analysis of the refluxed methanol solutions of **5a** and **6a** and ethanol solutions of **6a**, **8a**, **13a**, and **14a** confirmed production of **5** and **6** and **7**, **8**, **13**, and **14**, respectively. Subsequent UPLC-HRESIMS analysis of the refluxed H_2_O, MeOH, and EtOH solutions of *p*-hydroxybenzyl alcohol and the extracts of the fresh *G. elata* rhizomes and “tian ma” provides insights into medicinal chemistry behind the processing and decocting protocols of TCM. Herein described are details.

## Results and Discussion

### Isolation and Structure Elucidation of **1**−**20**

The pulverized “tian ma” (the steamed and air-dried *G. elata* rhizomes) was extracted by ultrasonicating with H_2_O. After concentrated, the aqueous extract was chromatographed over macroporous adsorbent resin, eluting with a gradient increasing EtOH in H_2_O to give fractions A−D. Fraction C was chromatographed over MCI gel, with successive elution using H_2_O, 30% EtOH, 50% EtOH, 95% EtOH, and Me_2_CO, to yield subfractions C1−C5. Further separation of the subfractions by column chromatography (CC) over Sephadex LH-20 and normal phase silica gel, middle-pressure liquid chromatography (MPLC) over reversed phase (C_18_) silica gel, and reversed phase high-performance liquid chromatography (RP-HPLC) afforded compounds **1**−**20** (see ‘Experimental’ section).

**Fig. 2:**
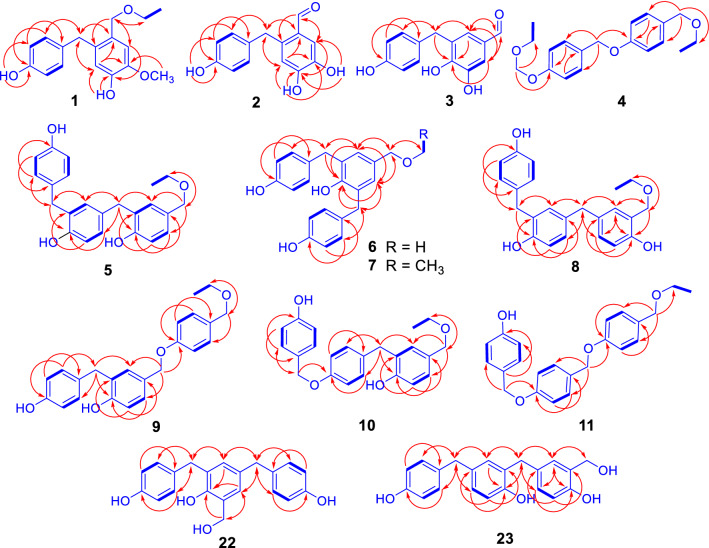
Main ^1^H-^1^H COSY (thick lines) and HMBC (arrows, from ^1^H to ^13^C) correlations of compounds **1**−**11**, **22**, and **23**

Compound **1**, a white amorphous powder, showed infrared (IR) absorptions for hydroxy (3392 cm^−1^) and aromatic ring (1614 and 1514 cm^−1^) functionalities. High resolution electrospray ionization mass spectrometry (HRESIMS) at *m/z* 311.1254 [M + Na]^+^ (calcd. for C_17_H_20_O_4_Na, 311.1254), together with the nuclear magnetic resonance (NMR) spectroscopic data (Table 1), determined the molecular formula of **1** as C_17_H_20_O_4_. The ^1^H NMR spectrum of **1** showed resonances assignable to a 2-substituted 4-hydroxy-5-methoxybenzyloxy unit at *δ*_H_ 7.46 (brs, 4-O*H*), 6.94 (s, H-6), 6.61 (1H, s, H-3), 3.82 (s, 5-OC*H*_*3*_), and 4.38 (s, H_2_-7); a *p*-hydroxybenzyl unit at *δ*_H_ 8.13 (brs, 4′-O*H*), 6.99 (d, *J* = 8.4 Hz, H-2′/6′), 6.74 (d, *J* = 8.4 Hz, H-3′/5′), and 3.84 (s, H_2_-7′); and an ethoxy group at *δ*_H_ 3.47 (q, *J* = 7.2 Hz, OC*H*_2_CH_3_) and 1.15 (t, *J* = 7.2 Hz, OCH_2_C*H*_3_). The ^13^C NMR spectrum of **1** exhibited corresponding signals to the above units (Table 1). These data indicated that **1** was a dimeric benzyl derivative containing an ethoxy group [71], which was confirmed by 2D NMR spectroscopic data analysis of **1** (Fig. 2). Especially, the heteronuclear multiple bond correlation (HMBC) spectrum of **1** showed correlations from H-3 to C-1 and C-5, from H-6 to C-2, C-4, and C-7; from H_2_-7 to C-2, C-6, and O*C*H_2_CH_3_; from 4-O*H* to C-3 and C-4; from 5-OC*H*_3_ to C-5; and from OC*H*_2_CH_3_ to C-7 (Fig. 2). This, together with the chemical shifts of the proton and carbon resonances, revealed the presence of an ethyl 2-substituted 4-hydroxy-5-methoxybenzyl ether moiety in **1**. The location of the *p*-hydroxybenzyl unit at C-2 in **1** was deduced from the HMBC correlations from H-3 to C-7′ and from H_2_-7′ to C-1, C-2′/6′, and C-3. Therefore, the structure of compound **1** was determined as ethyl 4-hydroxy-5-methoxy-2-(4′-hydroxybenzyl)benzyl ether and named gastrodibenzin A.

**Table 1:** The NMR spectroscopic data of compounds **1**−**4**

No.	**1**^a,c^		**2**^a,d^		**3**^b,c^		**4**^b,d,e^	
	*δ*_H_	*δ*_C_	*δ*_H_	*δ*_C_	*δ*_H_	*δ*_C_	*δ*_H_	*δ*_C_
1		128.6		125.5		129.9		130.7
2		133.9		137.6	7.20 s	126.5	7.21 d (8.5)	129.0
3	6.61 s	117.9	6.61 s	117.9		145.8	6.95 d (8.5)	114.5
4		146.7		151.5		150.3		157.7
5		146.2		144.0		130.6	6.95 d (8.5)	114.5
6	6.94 s	113.8	7.17 s	116.2	7.24 s	112.7	7.21 d (8.5)	129.0
7	4.38 s	71.0	10.01 s	190.4	9.69 s	191.2	4.34 s	71.2
1′		132.8		131.5		132.0		130.0
2′/6′	6.99 d (8.4)	130.5	6.90 d (8.4)	129.3	7.11 d (8.5)	130.6	7.36 d (8.5)	129.3
3′/5′	6.74 d (8.4)	115.9	6.63 d (8.4)	115.2	6.72 d (8.5)	115.9	7.01 d (8.5)	116.0
4'		156.4		155.4		156.5		156.6
7'	3.84 s	37.2	4.11 s	35.0	3.93 s	35.1	4.99 s	68.9
O*CH*_*2*_CH_3_	3.47 q (7.2)	65.8					3.42 q (7.0)	64.6
OCH_2_*CH*_*3*_	1.15 t (7.2)	15.6					1.11 t (7.0)	15.1
4-O*H*	7.46 brs		9.98 brs					
4′-O*H*	8.13 brs		9.17 brs					
5-O*H*/O*CH*_*3*_	/3.82 s	56.3	9.32 brs/					

Proton coupling constants (*J*) in Hz are given in parentheses. Assignments were based on DEPT, ^1^H-^1^H COSY, HSQC, and HMBC experiments

^a^NMR data (*δ*) were measured at 600 MHz for ^1^H and at 150 MHz for ^13^C

^b^Measured at 500 MHz for ^1^H and at 125 MHz for ^13^C

^c^Measured in acetone-*d*_6_

^d^Measured in DMSO-*d*_6_

^e^Data for OCH_2_OCH_2_CH_3_ in DMSO-*d*_6_: *δ*_H_ 5.22 (2H, s, OC*H*_*2*_OCH_2_CH_3_), 3.64 (2H, q, *J* = 7.0 Hz, OCH_2_OC*H*_*2*_CH_3_), 1.11 (3H, t, *J* = 7.0 Hz, OCH_2_OCH_2_C*H*_*3*_); *δ*_C_ 92.5 (O*C*H_2_OCH_2_CH_3_), 63.6 (OCH_2_O*C*H_2_CH_3_),15.0 (OCH_2_OCH_2_*C*H_3_)

Compound **2** was obtained as a yellowish amorphous powder. Its molecular formula was determined as C_14_H_12_O_4_ by HRESIMS at *m*/*z* 245.0807 [M + H]^+^ (calcd. for C_14_H_13_O_4_, 245.0808). The NMR spectroscopic data of **2** (Table 1) indicated the presence of a 2-subustituted 4,5-dihydroxybenzaldehyde unit, in addition to the *p*-hydroxybenzyl identical to that in **1**. This was verified by the HMBC correlations from H-3 to C-1 and C-5; from H-6 to C-2, C-4, and C-7; from H-7 to C-2 and C-6; from 4-O*H* to C-3 and C-5; and from 5-O*H* to C-4 and C-6, in combination with their chemical shifts. Meanwhile, the connection between the two units was demonstrated by the HMBC correlations from H-3 to C-7′ and from H_2_-7′ to C-1, C-2′/C-6′, and C-3. Therefore, the structure of **2** was determined as 4,5-dihydroxy-2-(4′-hydroxybenzyl)benzaldehyde and named gastrodibenzin B.

Compound **3**, a brownish amorphous powder, is an isomer of **2** as indicated by its spectroscopic data (Experimental and Table 1). Comparison of the NMR spectroscopic data between **3** and **2** suggested that the *p*-hydroxybenzyl was at C-3 of the 4,5-dihydroxybenzaldehyde unit in **3** instead of at C-2 in **2**. The suggestion was confirmed by 2D NMR data analysis of **3**, particularly by the HMBC correlations (Fig. 2) from H-2 to C-4, C-6, C-7, and C-7′; from H-6 to C-2, C-4, and C-7; from H-7 to C-2 and C-6; and from H_2_-7′ to C-2, C-2′/6′, and C-4, together with their chemical shifts. Therefore, the structure of compound **3** was determined as 4,5-dihydroxy-3-(4′-hydroxybenzyl)benzaldehyde and named gastrodibenzin C.

Compound **4**, a white amorphous powder, has the molecular formula of C_19_H_24_O_4_ as determined by HRESIMS at *m/z* 339.1572 [M + Na]^+^ (calcd. for C_19_H_24_O_4_Na, 339.1567). The NMR spectroscopic data of **4** (Table 1) indicated the presence of two inequivalent *p*-oxybenzyloxys, two inequivalent ethoxys, and an isolated dioxymethylene. In the HMBC spectrum of **4** (Fig. 2), the correlations from H_2_-7 to C-1 and C-2/C-6, from H-2/6 to C-4, and from H_2_-7′ to C-1′, C-2′/C-6′, and C-4, together with their chemical shifts demonstrated a head–tail connection of the two *p*-oxybenzyloxys via an ether bond between C-4 and C-7′. The HMBC correlations from H_2_-7 to the methylene carbon of one ethoxy unambiguously positioned the ethoxy at C-7. Moreover, the HMBC spectrum displayed the correlations from the dioxymethylene protons to C-4′ and the methylene carbon of the remaining ethoxy, indicating that an ethoxymethoxy unit^7^ was located at C-4′ of **4**. Accordingly, the structure of compound **4** was determined as ethyl 4-[4′-(ethoxymethoxy)benzyloxy]benzyl ether and named gastrodibenzin D.

Compound **5** was obtained as a white amorphous powder. Its molecular formula C_22_H_22_O_4_ was determined by HRESIMS at *m/z* 373.1397 [M + Na]^+^ (calcd. for C_22_H_22_O_4_Na, 373.1410). The NMR spectra of **5** showed resonances (Table 2) attributed to a *p*-hydroxybenzyl, two *m*-substituted *p*-hydroxybenzyls, and a methoxy group. This indicated that **5** was a methyl trimeric *p*-hydroxybenzyl ether analogue. In the HMBC spectrum of **5**, correlations of H-2 and H-6/C-4 and C-7; H-2′ and H-6′/C-4′ and C-7′, and H-2″ and H-6″/C-4″ and C-7″ (Fig. 2), together with the splitting patterns and shifts of these proton and carbon resonances, demonstrated that the three *p*-hydroxybenzyls connected each other via C-3−C-7′ and C-3′−C-7″ bonds. In addition, the HMBC correlations of OC*H*_3_/C-7 and H_2_-7/O*C*H_3_ located the methoxy group at C-7 in **5**. Therefore, the structure of **5** was determined as methyl 4-hydroxy-3-[4′-hydroxy-3′-(4″-hydroxybenzyl)benzyl]benzyl ether and named gastrotribenzin A.

**Table 2:** The NMR spectroscopic data of compounds **5**−**11**

No.	**5**^a,c^		**6**^a,c^		**7**^b,d^		**8**^b,d^		**9**^b,d^		**10**^b,d^		**11**^b,c^	
	*δ*_H_	*δ*_C_	*δ*_H_	*δ*_C_	*δ*_H_	*δ*_C_	*δ*_H_	*δ*_C_	*δ*_H_	*δ*_C_	*δ*_H_	*δ*_C_	*δ*_H_	*δ*_C_
1		127.6		128.9		131.1		133.7		130.6		130.9		130.7
2	6.86 d (1.8)	129.9	6.75 brs	127.8	6.89 s	128.9		154.2	7.15 d (2.4)	131.3	7.03 d (1.8)	131.0	7.22 d (8.4)	129.6
3		128.3		128.8		129.1	6.74 d (8.4)	115.8		129.2		128.7	6.95 d (8.4)	114.5
4		154.3		151.5		152.5	6.90 dd (8.4, 2.4)	129.6		155.5		155.0		157.7
5	6.91 d (8.0)	114.6		128.8		129.2		125.4	6.85 d (8.4)	115.8	6.80 d (7.8)	115.5	6.95 d (8.4)	114.5
6	6.73 dd (8.0, 1.8)	126.8	6.75 brs	127.8	6.89 s	128.9	7.04 brs	129.3	7.12 dd (8.4, 2.4)	127.9	6.99 dd (7.8, 1.8)	127.6	7.22 d (8.4)	129.6
7	4.18 s	73.7	4.14 s	73.8	4.27 s	73.0	4.51 s	69.2	4.38 s	72.6	4.30 s	72.9	4.35 s	71.2
1′		131.1		131.0		132.3		129.0		132.7		134.3		129.0
2′	6.88 d (1.8)	130.7	6.98 d (8.0)	129.6	7.05 d (8.4)	130.6	6.92 d (1.8)	131.6	7.07 d (8.4)	130.6	7.16 d (9.0)	130.5	7.35 d (9.0)	129.4
3′		127.8	6.65 d (8.0)	115.0	6.73 d (8.4)	115.9		133.6	6.71 d (8.4)	115.8	6.87 d (9.0)	115.4	6.99 d (9.0)	114.7
4′		152.9		155.3		156.4		153.9		156.4		158.1		158.2
5′	6.66 dd (8.0, 1.8)	114.8	6.65 d (8.0)	115.0	6.73 d (8.4)	115.9	6.72 d (7.8)	115.7	6.71 d (8.4)	115.8	6.87 d (9.0)	115.4	4.99 s	68.9
6′	6.80 d (8.0)	127.1	6.98 d (8.0)	129.6	7.05 d (8.4)	130.6	6.82 dd (7.8, 1.8)	128.0	7.07 d (8.4)	130.6	7.16 d (9.0)	130.5		127.1
7′	3.68 s	34.3	3.80 s	34.6	3.92 s	35.7	3.72 s	40.9	3.87 s	35.4	3.88 s	35.4	7.24 d (8.4)	129.0
1′′′		131.4		131.0		132.3		133.0		132.0		129.2	6.76 d (8.4)	115.1
2″/6″	6.96 d (7.0)	129.4	6.98 d (8.0)	129.6	7.05 d (8.4)	130.6	7.05 d (8.4)	130.5	7.22 d (9.0)	129.8	7.27 d (8.4)	130.2		157.2
3″/5″	6.61 d (7.0)	114.9	6.65 d (8.0)	115.0	6.73 d (8.4)	115.9	6.69 d (8.4)	115.7	6.92 d (9.0)	115.4	6.81 d (8.4)	115.9	4.95 s	69.2
4″		155.2		155.3		156.4		156.3		159.3		158.0		
7″	3.68 s	34.4	3.80 s	34.6	3.92 s	35.7	3.81 s	35.5	4.92 s	70.6	4.93 s	70.4		
7-OCH_3_	3.17 s	57.0	3.16 s	57.1										
O*CH*_*2*_CH_*3*_					3.41 q (7.2)	65.6	3.53 q (7.2)	66.3	3.45 q (7.2)	65.7	3.43 q (6.6)	65.6	3.43 q (7.2)	64.6
OCH_2_*CH*_*3*_					1.10 t (7.2)	15.5	1.17 t (7.2)	15.5	1.14 t (7.2)	15.5	1.11 t (6.6)	15.5	1.12 t (7.2)	15.1
4-O*H*	9.31		8.37 s		7.14 s		8.16 s		8.05 s					
4′-O*H*	9.31		9.12 s		8.09 s		8.26 s							
4″-O*H*	9.10		9.12 s		8.09 s		8.24 s		8.38 s				9.46 s	

Proton coupling constants (*J*) in Hz are given in parentheses. Assignments were based on DEPT, ^1^H-^1^H COSY, HSQC, and HMBC experiments

^a^NMR data (*δ*) were measured at 500 MHz for ^1^H and at 125 MHz for ^13^C

^b^Measured at 600 MHz for ^1^H and at 150 MHz for ^13^C

^c^Measured in DMSO-*d*_6_

^d^Measured in acetone-*d*_6_

Compound **6**, a white amorphous powder, is an isomer of **5** as indicated by the IR, HRESIMS (see ‘Experimental’ section) and NMR spectroscopic data (Table 2). Comparison of the NMR spectroscopic data of the two compounds indicated the presence of two equivalent *p*-hydroxybenzyls, a symmetrically disubstituted *p*-hydroxybenzyl, and an methoxy group in **5**. This suggested that the terminal 4-hydroxybenzyl moiety at C-3′ in **5** was migrated to C-5 in **6**. which was confirmed by 2D NMR data analysis. Especially, the HMBC correlations from O*H*-4 and H_2_-7′/H_2_-7″ to C-4 and from OC*H*_3_ and H-2/H-6 to C-7 demonstrated that the two equivalent *p*-hydroxybenzyls were substituted at C-3 and C-5 of the methyl 4-hydroxybenzyl ether unit to give a symmetric structure. Therefore, the structure of compound **6** was determined as methyl 4-hydroxy-3,5-di-(4′-hydroxybenzyl)benzyl ether and named gastrotribenzin B.

Compound **7** was isolated as a white amorphous powder. Comparison of the spectroscopic data between **7** and **6** revealed replacement of the methyl group in **6** by an ethyl group [*δ*_H_ 3.41 (2H, q, *J* = 7.2 Hz, OC*H*_*2*_CH_3_) and 1.10 (3H, t, *J* = 7.2 Hz, OCH_2_C*H*_*3*_), and *δ*_C_ 65.6 (O*C*H_2_CH_3_) and 15.5 (OCH_2_*C*H_3_)] in **7**. This was further confirmed by the correlations of OC*H*_*2*_CH_3_/C-7 and H_2_-7/O*C*H_2_CH_3_ in the HMBC spectrum of **7** (Fig. 2). Thus, the structure of compound **7** was determined as ethyl 4-hydroxy-3,5-di-(4′-hydroxybenzyl)benzyl ether and named gastrotribenzin C.

Compound **8**, a white amorphous powder, is an isomer of **7** having a different connection of hydroxybenzyls as indicated by the spectroscopic data (Experimental and Table 2). The HMBC spectrum of **8** showed the correlations (Fig. 2) from H_2_-7 to C-1, C-2, and O*C*H_2_CH_3_; from H_2_-7′ to C-2′, C-4, C-6, and C-6′; and from H_2_-7″ to C-2′, C-2″, C-4′, and C-6″. These correlations, together with their coupling constants and chemical shifts, demonstrated C-5−C-7′ and C-3′−C-7″ linkage of the three hydroxybenzyls and location of the three hydroxy groups and the ethoxy at C-2, C-4′, and C-4″ and C-7, respectively. Hence, the structure of compound **8** was determined as ethyl 2-hydroxy-5-[4′-hydroxy-3′-(4″-hydroxybenzyl)-benzyl]benzyl ether and named gastrotribenzin D.

Compound **9** was obtained as a white amorphous powder. The spectroscopic data showed that this compound was another isomer of **7** and **8**. In the HMBC spectrum of **9**, the correlations (Fig. 2) from H_2_-7 to C-2 and C-6; from H_2_-7′ to C-2, C-2′, C-4, and C-6′; from 4-O*H* to C-3, C-4, and C-5; and from 4′-O*H* to C-3′/5′ and C-4′, together with their chemical shifts, revealed the presence of a 4-hydroxy-3-(4′-hydroxybenzyl)benzyloxy moiety. In addition, the HMBC spectrum showed the correlations from H_2_-7 to C-4″, from H_2_-7″ to C-2″/C-6″, and O*C*H_2_CH_3_; and from OC*H*_2_CH_3_ to C-7″. These correlations, in combination with their chemical shifts, unambiguously revealed the oxygen-bridged connections between C-4″ and C-7 and between C-7″ and the ethyl group. Thus, the structure of compound **9** was determined as 4-hydroxy-3-(4′-hydroxybenzyl)benzyl 4″-ethoxymethylphenyl ether and named gastrotribenzin E.

The spectroscopic data of compound **10** indicated that it was one more isomer of **7**−**9**. The HMBC spectrum of **10** exhibited the correlations (Fig. 2) from H_2_-7 to C-2, C-6, and O*C*H_2_CH_3_; from H_2_-7′ to C-2, C-2′/6′, C-3, and C-4; from H_2_-7″ to C-2″/6″ and C-4′; and from OC*H*_*2*_CH_3_ to C-7. These correlations, together with their chemical shifts, indicated the connection of C-3 to C-7′ as well as the ether-bond linkages of C-4′ to C-7″ and C-7 to the ethyl group in **10**. Therefore, the structure of compound **10** was determined as ethyl 4-hydroxy-3-[4′-(4″-hydroxybenzyloxy)benzyl]benzyl ether and named gastrotribenzin F.

By comparison of spectroscopic data with those reported in literature, the known compounds were identified as ethyl 4-[4′-(4″-hydroxybenzyloxy)benzyloxy]benzyl ether (**11**) [81], ethyl 4-(4′-hydroxybenzyloxy)benzyl ether (**12**) [82], ethyl 4-hydroxy-3-(4′-hydroxybenzyl)benzyl ether (gastropolybenzylol C, **13**) [8], gastropolybenzylol B (**14**) [8], bis(4-hydroxybenzyl)ether (**15**) [82], 4-hydroxybenzyl vanillyl ether (**16**) [83], 4,4′-methylenediphenol (**17**) [82], 2,4-bis(4-hydroxybenzyl)phenol (**18**) [84], gastropolybenzylol A (**19**) [8], gastrol A (**20**) [85]. The structure of **11** was previously determined only by UPLC/Q-TOF MS analysis [81] and identified in this study by comprehensive analysis of the spectroscopic data including 2D NMR experiments (Fig. 2), for which the detailed physical–chemical properties are reported (‘Experimental’ section).

### Products from a Refluxed Aqueous Solution of *p*-Hydroxybenzyl Alcohol and Their Etherification with MeOH and EtOH

Among the 20 isolates, compounds **1**−**3** and **16** contain *p*-hydroxybenzyl and vanillyl alcohol (**1** and **16**) or protocatechualdehyde units (**2** and **3**), while the others are analogues of *p*-hydroxybenzyl-derived dimers (**4**, **12**−**15** and **17**) and trimers (**5**−**11** and **18**−**20**). Because *p*-hydroxybenzyl alcohol, which abundantly occurs in *G. elata* [50–58], is highly reactive to produce quinone methide and complex derivative via self-condensation or inter-condensation with other reactants under various conditions [86–89], this suggests that, (a) *p*-hydroxybenzyl alcohol is an origin of the *p*-hydroxybenzyl unit in the *p*-hydroxybenzyl-containing chemical constituents of “tian ma”; (b) alcoholic forms of the *p*-hydroxybenzyl-derived dimers and trimers are generable from *p*-hydroxybenzyl alcohol during processing and/or extracting of the drug material; and (c) the ethyl and methyl ethers were formed in the subsequent isolation procedure through contacting to the solvents EtOH and MeOH. To verify the suggestions, following experiments were performed: (a) an aqueous solution of *p*-hydroxybenzyl alcohol was refluxed, from which the products were isolated and structurally identified; (b) methylation and ethylation of the *p*-hydroxybenzyl alcohol-generating products were examined by UPLC-HRESIMS after refluxing of their MeOH and EtOH solutions; (c) the refluxed solutions of *p*-hydroxybenzyl alcohol in H_2_O, MeOH, and EtOH were compared by UPLC-HRESIMS analysis using the identified pure compounds as references.

As expected, all the experimental results supported the suggestions. Briefly, the dibenzyl and tribenzyl alcohols **5a**, **6a**, **8a**, **13a**, and **14a** as well as compounds **15**, **17**−**19**, **21**−**25**, and *p*-hydroxybenzaldehyde were isolated from the refluxed aqueous solution of *p*-hydroxybenzyl alcohol. The known compounds **13a** [90], **15** [82], **17** [82], **18** [84], **19** [8], **21** [90], **25** [71], and *p*-hydroxybenzaldehyde [82] were previously reported as the “tian ma” constituents, while **14a** and **24** were found as intermediates in the cure process of resol phenol–formaldehyde resins [91]. The structures of the new compounds **5a**, **6a**, **8a**, **22**, and **23** were readily determined by comparison of their spectroscopic data with those of **5**−**8** (‘Experimental’ section and Tables 2 and 3) and confirmed by analysis of the 2D NMR spectroscopic data (Fig. 2 and Supporting Information Figs. S133−S177).

**Table 3:** The NMR spectroscopic data of compounds **5a**, **6a**, **8a**, **22**, and **23**

No.	**5a**		**6a**		**8a**		**22**		**23**	
	*δ*_H_	*δ*_C_	*δ*_H_	*δ*_C_	*δ*_H_	*δ*_C_	*δ*_H_	*δ*_C_	*δ*_H_	*δ*_C_
1		133.4		133.8		127.7		126.3		128.9
2	6.99 brs	129.1	6.90 s	127.1		154.3		153.1		154.3
3		128.0		128.2	6.67 d (7.8)	115.8		129.5	6.73 d (7.8)	115.8
4		153.8		151.2	6.96 dd (7.8, 2.4)	129.1	6.87 d (1.8)	130.6	6.81 dd (7.8, 1.8)	128.0
5	6.77 d (7.8)	114.6		128.2		133.0		133.7		133.7
6	6.99 brd (7.8)	125.6	6.90 s	127.1	7.11 d (2.4)	128.8	6.73 d (1.8)	126.4	7.03 d (1.8)	128.6
7	4.43 d (6.0)	63.8	4.41 d (6.0)	63.8	4.67 d (5.4)	62.1	4.76 d (4.8)	64.3	4.67 s	62.0
1′		132.2		131.6		133.7		132.9		127.9
2′	7.01 d (1.8)	131.1	7.04 d (8.4)	129.7	6.93 d (2.4)	131.7	7.05 d (8.4)	130.5		153.9
3′		127.8	6.72 d (8.4)	115.0		128.9	6.70 d (8.4)	115.7	6.68 d (8.4)	115.7
4′		152.9		155.5		153.8		156.3	6.88 dd (8.4, 1.8)	128.9
5′	6.71 d (7.8)	114.8	6.72 d (8.4)	115.0	6.74 d (8.4)	115.8	6.70 d (8.4)	115.7		133.7
6′	6.88 dd (7.8, 1.8)	127.3	7.04 d (8.4)	129.7	6.81 dd (8.4, 2.4)	128.0	7.05 d (8.4)	130.5	6.89 d (1.8)	131.6
7′	3.81 s	34.6	3.90 s	34.9	3.82 s	35.5	3.82 s	35.2	3.81 s	35.5
1′′′		132.1		131.6		133.7		133.4		133.0
2″/6″	7.05d (7.8)	129.6	7.04 d (8.4)	129.7	6.97 d (8.4)	130.4	6.97 d (8.4)	130.4	7.05 d (7.8)	130.5
3″/5″	6.99 d (7.8)	114.8	6.72 d (8.4)	115.0	6.71 d (8.4)	115.9	6.71 d (8.4)	115.9	6.70 d (7.8)	115.7
4″		155.3		155.5		156.3		156.2		156.2
7″	3.81 s	34.7	3.90 s	34.9	3.71 s	40.8	3.70 s	40.8	3.71 s	40.9
7-O*H*	3.83 t (6.0)		3.86 t (6.0)		4.39 t (5.4)		5.04 t (4.8)		4.38 brs	
2-O*H*					8.22 s		8.21 s		8.26	
4-O*H*	8.11 s		7.05 s							
2′-O*H*									8.12 s	
4′-O*H*	7.98 s		8.05 s		8.11 s		8.05 s			
4″-O*H*	7.99 s		8.05 s		8.14 s		8.09 s		8.12 s	

NMR data (*δ*) were measured in acetone-*d*_6_ at 600 MHz for ^1^H and at 150 MHz for ^13^C, respectively. Proton coupling constants (*J*) in Hz are given in parentheses. Assignments were based on DEPT, ^1^H-^1^H COSY, HSQC, and HMBC experiments

**Fig. 3:**
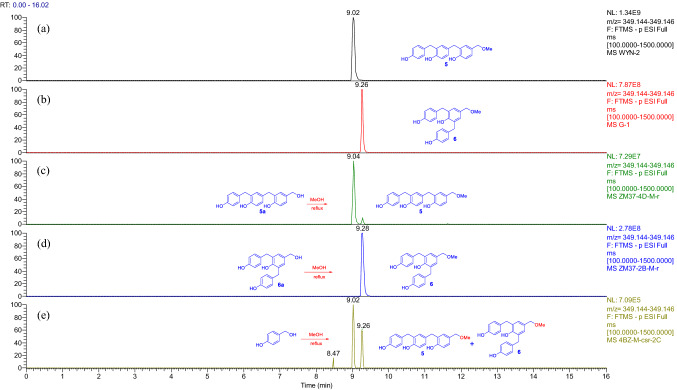
Overlaid chromatograms of the extracted negative ion at *m/z* 349.145 [M − H]^−^:** a**,** b** compounds **5** and **6** in CH_3_CN, respectively;** c**,** d** MeOH solutions of **5a** and **6a** were refluxed for 1 h, respectively;** e** MeOH solution of *p*-hydroxybenzyl alcohol was sonicated for 0.5 h then refluxed for 1.0 h

**Fig. 4:**
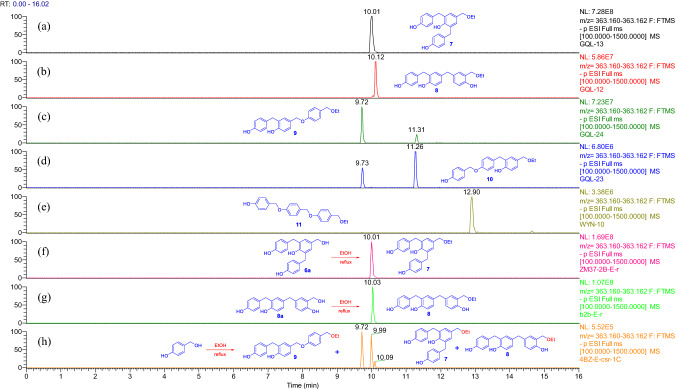
Overlaid chromatograms of the extracted negative ion at *m/z* 363.161 [M − H]^−^:** a**−**e** compounds **7**−**11** in CH_3_CN, respectively; (f, g) EtOH solutions of **6a** and **8a** were refluxed for 1.0 h, respectively;** h** EtOH solution of *p*-hydroxybenzyl alcohol was sonicated for 0.5 h then refluxed for 1.0 h

**Fig. 5:**
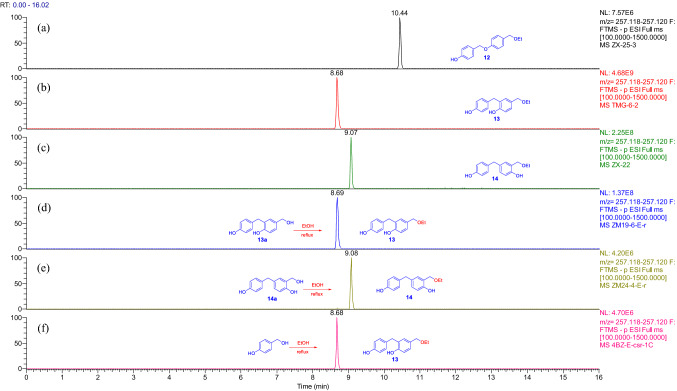
Overlaid chromatograms of the extracted negative ion at *m/z* 257.119 [M–H]^–^:** a**–**c** compounds **12**–**14** in CH_3_CN, respectively;** d**,** e** EtOH solutions of **13a** and **14a** were refluxed for 1.0 h, respectively;** f** EtOH solution of *p*-hydroxybenzyl alcohol was sonicated for 0.5 h then refluxed for 1.0 h

UPLC-HRESIMS analysis proved that **5** and **6** were produced respectively by refluxing of **5a** and **6a** or *p*-hydroxybenzyl alcohol in methanol (Fig. 3), while **7**, **8**, **13**, and **14** were yielded by refluxing of **6a**, **8a**, **13a**, and **14a**, of which only **14** was undetectable from the refluxed EtOH solution of *p*-hydroxybenzyl alcohol (Figs. 4 and 5). All the isolated compounds from the refluxed H_2_O solution of *p*-hydroxybenzyl alcohol were detectable in the refluxed MeOH and EtOH solutions (Supporting Information Figs. S178−S183). However, except for compounds **8**, **9**, and **20**, the phenolic ethers **10**−**12** and **19** were undetectable in the refluxed EtOH solution of *p*-hydroxybenzyl alcohol (Figs. 4, 5, and S178−S213), while the corresponding alcoholic forms of **9**−**12** as well as compounds **19** and **20** were not obtained from the refluxed H_2_O solution of *p*-hydroxybenzyl alcohol. This may be due to a structural instability of the phenolic ethers and/or their relative low abundance, which was preliminarily supported by interconversion of **9** and **10** in CH_3_CN (Fig. 4). In addition, compositions and abundances of the isomeric dimers and trimers were significantly varied in the sonicated and refluxed solutions (Figs. S184−S213). With increase of the refluxing time, the relative abundances of the *p*-hydroxybenzyl ethers **15** and **21** were significantly decreased in the H_2_O solution, whereas the corresponding *p*-hydroxybenzyl-substituted *p*-hydroxybenzyl alcohols **5a/6a** and **13a** were significantly increased. Meanwhile, the relative contents of **8a**, **14a**, **22**, and **23** were decreased also with increase of the refluxing time, and the content-decreased compounds had higher relative abundances in the sonicated H_2_O solution without refluxing (Supporting Information Figs. S190, S191, and S193). However, relative content variations of the isomeric trimers **5a**/**6a**, **8a**, and **21**−**23** in the refluxed MeOH and EtOH solutions of *p*-hydroxybenzyl alcohol (Figs. S198, S199, S205, and S206) were insignificant as compared with the refluxed H_2_O solution (Figs. S190 and S191), while the relative content variations of the isomeric dimers **13a**, **14a**, and **15** in the refluxed MeOH solution (Fig. S201) were insignificant as compared with that in the H_2_O and EtOH solutions (Figs. S193 and S210). The aforementioned observation suggested that the preferentially formed **15** and **21** were instable in the solutions to convert into the other compounds. The suggestion was further confirmed by sonicating and refluxing of the H_2_O, MeOH, and EtOH solutions of **15** and **21**, respectively (Figs. S214−S232). Compounds **5** and **6** were generated in the sonicated MeOH solution of **15** (Fig. S214) while several unidentified isomers were produced in the sonicated solutions of **21** (Fig. S222). Compound **15** was readily converted into **21** by sonicating in H_2_O, MeOH, and EtOH (Fig. S215), whereas the reverse conversion from **21** into **15** was detectable after the MeOH and EtOH solutions were refluxed for 2 h and after the sonicated H_2_O solution was refluxed for 4 h (Figs. S228−S230). Compounds **17**, **18**, and **25** were consistently detectable in the sonicated H_2_O, MeOH, and EtOH solutions of both **15** and **21** (Figs. S217, S220, S221, S226, S231, and S232); meanwhile, **13a** was detectable after the sonicated solutions of **15** were refluxed for 2 h (Fig. S219), but abundantly appeared in the sonicated solutions of **21** (Fig. S228). In addition, the ethyl ethers **7**, **9**, **10**, and **13** were observable in the sonicated EtOH solution of **15** (Figs. S216 and S218), and production of **7**, **9**, and **10** from **21** was secured after the sonicated EtOH solution was further refluxed for 1.0 h (Figs. S224 and S225). Moreover, generation of **22** was detectable in the sonicated solutions of **21** (Figs. S223). Particularly the transformation from **21** to the ethyl ethers **7** and **13** in the H_2_O and MeOH solutions (Figs. S223, S224, and S227) was unexpected and of interesting, which was confirmed by the paralleled and repeated experiments. Because the ethyl unit to form **7** and **13** was highly suspected to be producible in the reaction system, its ambient ethanol origin should not be excluded.

The above experiments demonstrated that the composition of the refluxed solutions of *p*-hydroxybenzyl alcohol are highly dependent upon the solvent and refluxing time. Because of the abundant occurrence of *p*-hydroxybenzyl alcohol in *G. elata* [50–58] (Fig. S233), the *p*-hydroxybenzyl-derived dimers and trimers from the “tian ma” extracts must be formed at least partially by refluxing of the drug materials in H_2_O, MeOH, or EtOH. Meanwhile, some dimers and trimers can be converted and/or transformed each other. This supports that indeed the chemical reactions take place during the processing and decocting of “tian ma” to produce the compounds and to modify the chemical composition.

### UPLC/HRESIMS Analysis of H_2_O, MeOH, and EtOH Extracts of the *G. elata* rhizomes

To confirm the chemical reactions during processing and decocting of the herbal medicine [80], the fresh *G. elata* rhizomes were collected at the same field of “tian ma” (the steamed and dried rhizomes) and the extracts were prepared by sonicating of the fresh *G. elata* rhizomes and “tian ma” in H_2_O, MeOH, and EtOH for 0.5 h, respectively, followed by refluxing (sampling time: 0.5 h, 1.0 h, 1.5 h, 2.0 h, 4.0 h, and 6.0 h). The extract samples were analyzed by UPLC-HRESIMS using the aforementioned pure compounds as the references, showed that the composition and relative content of the extracts were varied with the extracting solvent and refluxing time.

The precursor *p*-hydroxybenzyl alcohol and compounds **5a**/**6a**, **13a**, **15**, **17**, **18**, **21**, and **25** were detectable in the extracts obtained by sonicating of the fresh *G. elata* rhizomes and “tian ma” in H_2_O, MeOH, and EtOH, respectively (Supporting Information Figs. S233−S239), except that the trimers **5a**/**6a**, **18**, and **21** were undetectable in the H_2_O extract (Figs. S234 and S235). In addition, the relative content of the isomers **13a** and **15** in the fresh rhizome extracts were reversed in the “tian ma” extracts (Fig. S236), suggesting that **13a** was generated at least partially during processing of the drug material.

**Fig. 6:**
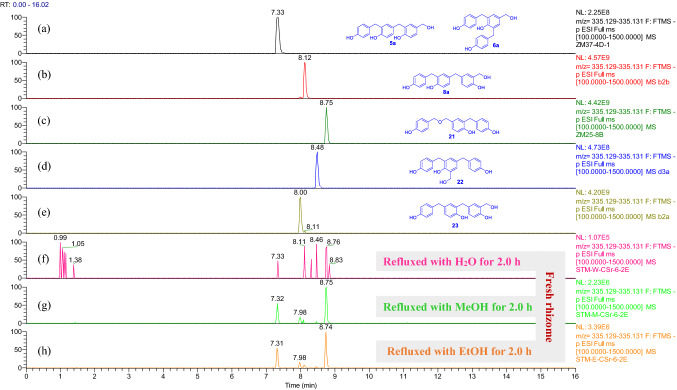
Overlaid chromatograms of the extracted negative ion at *m/z* 335.130 [M − H]^−^: (a−e) compounds **5a**/**6a**, **8a**, and **21**−**23** in CH_3_CN, respectively; (f−h) extracts obtained by sonicating of fresh *G. elata* rhizomes with H_2_O, MeOH, and EtOH for 0.5 h then refluxing for 2.0 h, respectively

**Fig. 7:**
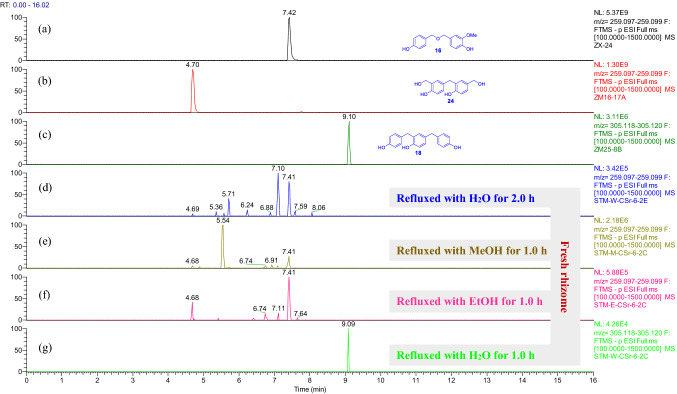
Overlaid chromatograms of the extracted negative ions at *m/z* 259.098 [M − H]^−^ for (a, b, d−f) and at 305.119 [M − H]^−^ for (c, g), respectively: (a−c) compounds **16**, **24**, and **18** in CH_3_CN, respectively; (d−g) extracts obtained by sonicating of fresh *G. elata* rhizomes with H_2_O, MeOH, and EtOH for 0.5 h then refluxing for 1.0 h or 2.0 h, respectively

For the fresh *G. elata* rhizomes (Figs. S240−S261), the trimeric isomers **5a**/**6a**, **8a**, and **21**−**23** were detectable in the H_2_O, MeOH, and EtOH extracts after refluxed for 2 h, 4 h, and 6 h (Fig. 6 and Supporting Information Figs. S240, S241, S247, S248, S254, and S255). The dimers **14a** and **24** were detectable in the MeOH and EtOH extracts after refluxed for 1 h and 2 h (Figs. S250, S251, S258, and S259). The dimer **16** and trimer **18** were detectable in all the H_2_O, MeOH, and EtOH extracts after refluxed for more than 1 h (Fig. 7 and Supporting Information Figs. S242, S244, S249, S251, S256, and S259). Interestingly compound **18** disappeared in the MeOH extract after refluxed for 6 h (Fig. S249). Among the ethers, compound **13** was detectable only in the EtOH extracts of the fresh *G. elata* rhizomes (Fig. S257), indicating that this compound was formed from contacting with EtOH in the experimental procedure. When compared with the refluxed H_2_O extracts (Figs. S240 and S243), with increase of the refluxing time, the relative contents of **5a**/**6a** and **13a** were decreased in the MeOH and EtOH extracts (Figs. S247, S250, S254, and S258) while **13** was relatively increased in the EtOH extracts (Fig. S257). This demonstrated that **5a**/**6a** and **13a** were reacted with the solvents to be transformed into the corresponding methyl and ethyl ethers during refluxing, though compounds **5**−**7** were undetectable in the fresh *G. elata* rhizome extracts possibly due to low content.

As compared with extracts of the fresh *G. elata* rhizomes, more compounds were detectable in the “tian ma” extracts (Figs. S262−S289). Compounds **2** and **3** appeared in all the refluxed “tian ma” extracts (Figs. S262, S270, and S280) and **1** in the EtOH extracts after refluxed for 2 h and 6 h (Fig. S279). Particularly the methyl ethers **5** and **6** appeared only in the refluxed MeOH extracts (Fig. 8 and Supporting Information Fig. S271) and the ethyl ethers **7**, **9**, and **14** in the refluxed EtOH extracts (Figs. 8, S284, and S285). This further supports that the methyl and ethyl ethers were produced from reaction of the corresponding dimeric and trimeric benzyl alcohols (such as **5a**/**6a**, **13a**, and **14a**) with the solvents.

**Fig. 8:**
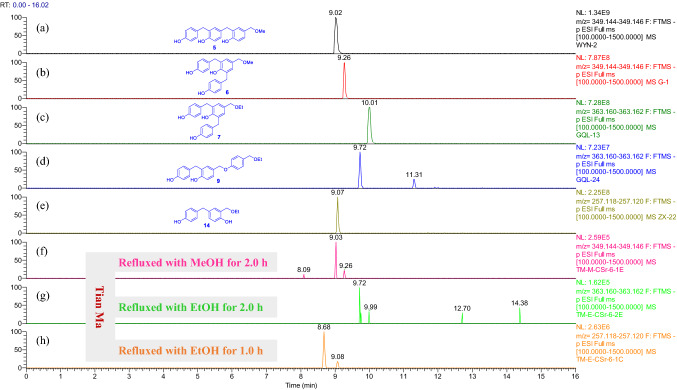
Overlaid chromatograms of the extracted negative ions at *m/z* 349.145 [M − H]^−^ for (a, b, f); and at 363.161 [M − H]^−^ for (c, d, g); and at 257.119 [M − H]^−^ for (e, h), respectively: (a−e) compounds **5**, **6**, **7**, **9**, and **14** in CH_3_CN, respectively; (f−h) extracts obtained by sonicating of “tian ma” with MeOH and EtOH for 0.5 h then refluxing for 1.0 h or 2.0 h, respectively

The isolated minor compounds **4**, **8**, **9**−**12**, **19**, and **20** were undetectable in the extracts of either the fresh *G. elata* rhizomes or “tian ma”, this may be explained by their low contents, since **4**, **10**−**12**, and **19** were also undetectable in the refluxed EtOH solutions of *p*-hydroxybenzyl alcohol and since relative low peak intensities of **8**, **9**, and **20** were observed in chromatograms of the refluxed EtOH and/or H_2_O solutions of *p*-hydroxybenzyl alcohol. The detectable main compounds in the extracts were completely identical to the main products from the refluxed solutions of *p*-hydroxybenzyl alcohol. Particularly the dimeric analogues **13a**, **15**, **17**, **21**, and **25** were readily detectable in all the sonicated extracts of the fresh *G. elata* rhizomes and “tian ma” as well as the refluxed solutions of *p*-hydroxybenzyl alcohol.

Because the high abundance and reactivity of *p*-hydroxybenzyl alcohol in the fresh *G. elata* rhizomes and “tian ma” were repeatedly confirmed [50–58, 86–89], the above results unraveled that the constituents of the extracts were modified at least partially by the chemical reactions of *p*-hydroxybenzyl alcohol when the drug materials were refluxed with H_2_O, MeOH, and EtOH. Nevertheless, the natural occurrence and production of the *p*-hydroxybenzyl alcohol-derived dimers and trimers should not be excluded since there is no evidence for exception of the reactions under physiological environments.

### Activities of the Purified Compounds

Because previous studies revealed neuronal protection, anti-inflammatory, and antioxidant played important roles in the neurological effects of the extracts and chemical constituents of *G. elata* [92], the purified compounds from the aqueous extract were assayed preliminarily on the corresponding cell-based models [72, 79]. At a concentration of 10 μmol/L, as compared with the blank control, compounds **5**, **7**, **17**−**19** attenuated rotenone-induced PC12 cell damage by increasing the cell viability from 56.08 ± 4.4% to 71.28 ± 9.4%, 82.43 ± 0.08%, 77.91 ± 0.07%, 62.25 ± 7.89%, and 76.26 ± 11.3%, respectively. At the same concentration, as compared with the model group, **17** and the positive control bicyclol protected dl-galactosamine (GalN)-induced hepatocyte (WB-F344 cell) damage by increasing cell survive rates from 16 to 21% and 19%, respectively. In addition, compounds **5**, **7**, **8**, **17**, and **19** and the positive control dexamethasone inhibited LPS-induced NO production in mouse peritoneal macrophages with inhibition rates of 78.1%, 74.7%, 91.4%, 92.1% and 83.4%, respectively, while **5**, **7**, **8**, **12**, **15**, **17**−**19**, and the positive control glutathione inhibited Fe^2+^-cystine-induced rat liver microsomal lipid peroxidation with inhibition rates of 74%, 59%, 82%, 62%, 93%, 67%, 89%, 78%, and 49%. The results indicated that compound **17** was active in all the four assays while **5**, **7**, and **19** were active in the three assays. The remaining compounds including gastrodin and *p*-hydroxybenzyl alcohol were inactive at the same concentration.

The previous study on the isolated guinea-pig ileum smooth muscle showed that **17** and the alcohol form of **11** had inhibitory effects on neurotransmitter release induced by stimulation of nicotine, serotonin, and vanilloid receptors, while **18** and **21** affected acetylcholine-induced contraction more directly [90]. Compounds **13**, **14**, **17**, **18**, and **21** were activators of melatonin receptors [7]. In addition, **15**, **17**, and the methyl ether analogue of **11** exhibited significant inhibitory effects on collagen, epinephrine, arachidonic acid, U46619 induced platelet aggregation [93], **17** had vasodilatory effect [94], and **18** was found to be a heat shock factor 1 (HSF1) inhibitor [95]. The studies also demonstrated that the main components gastrodin and *p*-hydroxybenzyl alcohol were less active or inactive as compared with the “tian ma” extracts as well as the dimers and trimers [90, 93, 94]. Thus, the *p*-hydroxybenzyl alcohol-derived dimers and trimers, which are the modified and recombined components during processing and decocting of the drug material, have important contributions to the clinic effects of the “tian ma” decoction.

## Conclusions

Ten new *p*-hydroxybenzyl-derived dimers and trimers, gastrodibenzins A−D and gastrotribenzins A−F, together with ten known analogues, were isolated from an aqueous extract of “tian ma”. Compounds **2** and **3** represents the first examples of *p*-hydroxybenzyl-coupled protocatechualdehydes. From the refluxed aqueous solution of *p*-hydroxybenzyl alcohol, isolation and identification of **5a**, **6a**, **8a**, **13a**, **14a**, **15**, **17**−**19**, **21**, **24**−**25**, and *p*-hydroxybenzaldehyde, in combination with UPLC-HRESIMS analysis, unraveled that: (a) the *p*-hydroxybenzyl unit in the “tian ma” chemical constituents were originated from *p*-hydroxybenzyl alcohol through self-condensation (**4**−**15** and **17**−**20**) and inter-condensation with other molecules (**1**−**3** and **16**), which could be produced, modified, and recombined during processing and decocting of the drug material; (b) the *p*-hydroxybenzyl-derived methyl and ethyl ethers (such as **4**−**14**) could readily be formed by contacting to the solvents MeOH and EtOH in the experimental procedure, respectively; (c) the chemical constituents of ‘tian ma” extracts were highly dependent upon processing and extracting protocols including the solvents and refluxing time. This study, together with our previous results^71−80^, provides valuable insights into medicinal chemistry behind the processing and decocting protocols of TCM. This unravels that the composition and content of the diverse *p*-hydroxybenzyl-derived constituents of “tian ma” and their contributions to the pharmacological effects are modified and recombined by the processing and decocting. The processing and decocting protocols of TCM indeed enhance the medicinal values of the herbal medicine and deserve to be further investigated and to be in deep validated for more complex formulations.

## Experimental

### General experimental procedures

See Supporting Information.

### Plant material

See Supporting Information.

### Extraction and isolation

For preliminary extraction and isolation, see Refs. 71−75 and Supporting Information. Fraction C1 (66 g) was separated by CC over Sephadex LH-20, successively eluting with H_2_O, 30% EtOH, 50% EtOH, and 95% EtOH, to give subfractions C1-1–C1-4. Further fractionation of C1-2 (36 g) by RP-MPLC (C_18_ silica gel, 50 μm, YMC), eluting with a gradient of increasing MeOH (0–100%) in H_2_O, yielded subfractions C1-2-1–C1-2-4. Subfraction C1-2-3 (9.3 g) was chromatographed over silica gel, eluting with a gradient of increasing MeOH (0–100%) in CHCl_3_, to yield C1-2-3-1−C1-2-3-5. Fraction C1-2-3-2 (3.98 g) was separated by CC over Sephadex LH-20 (MeOH) yielded C1-2-3-2-1−C1-2-3-2-3, of which C1-2-3-2-2 (1.31 g) and C1-2-3-2-3 (1.26 g) were individually isolated by RP-HPLC (60% MeOH in H_2_O, C_18_ column, 2.0 mL/min) to afford **6** (8.6 mg, *t*_R_ = 26.5 min) from the former and **11** (19.3 mg, *t*_R_ = 27.6 min) from the latter. Fraction C1-2-3-3 (0.61 g) was chromatographed over Sephadex LH-20 (50% MeOH) to give a mixture, which was successively isolated by CC over silica gel (CHCl_3_-MeOH, 15:1) and RP-HPLC (70% MeOH in H_2_O, C_18_ column, 2.0 mL/min) to obtain **14** (13.8 mg, *t*_R_ = 11.9 min). Further fractionation of C1-3 (36 g) by CC over Sephadex LH-20 (50% MeOH) gave C1-3-1−C1-3-4. Fraction C1-3-2 (1.2 g) was isolated by CC over silica gel, eluting with a gradient of increasing MeOH (0−100%) in CHCl_3_, to yield C1-3-2-1 (0.48 g), which was further separated by RP-HPLC (65% MeOH in H_2_O, C_18_ column, 2.0 mL/min) to afford **4** (56.2 mg, *t*_R_ = 31.2 min).

Fraction C2 (302 g) was subjected CC over silica gel (ethyl acetate–EtOH–H_2_O, 16:2:1−2:2:1) to give subfractions C2-1−C2-5. Subfraction C2-1 (52.5 g) was chromatographed over silica gel, eluting with a gradient of increasing MeOH (0−100%) in CHCl_3_, to yield C2-1-1−C2-1-6. Fraction C2-1-2 (7.2 g) was re-separated by CC (CHCl_3_–MeOH, 100:0−0:10) to yield C2-1-2-1−C2-1-2-5, of which C2-1-2-3 (1.8 g) C2-1-2-5 (1.5 g) was further fractionated by CC over Sephadex LH-20 (MeOH) to yield C2-1-2-5-1−C2-1-2-5–3. Fraction C2-1-2-5–2 (180 mg) was separated by CC over silica gel (CHCl_3_–MeOH, 10:1) to give C2-1-2-5-2-1−C2-1-2-5-2-3, of which C2-1-2-5-2-1 (28 mg) was isolated by preparative TLC (CHCl_3_-MeOH, 10:1), followed by purification with RP-HPLC (52% MeOH in H_2_O, Ph column, 2.0 mL/min) to obtain **2** (2.3 mg, *t*_R_ = 12.5 min) and **3** (2.1 mg, *t*_R_ = 13.6 min).

Fraction C3 (237 g) was subjected to CC over silica gel, eluting with a gradient of increasing MeOH (0−100%) in EtOAc followed by 30% EtOH, to yield fractions C3-1−C3-5 based on TLC analysis. Fraction C3-1 (27.3 g) was separated by silica gel CC (petroleum ether-ethyl acetate, 50:1–1:1) to give C3-1-1−C3-1-6, of which C3-1-1 (780 mg) was further fractionated by Flash CC over reversed phase silica gel (C_18_) (10–70% MeOH in H_2_O) to yield C3-1-1-1−C3-1-1-8. Purification of C3-1-1-2 (34 mg) by RP HPLC (50% MeOH in H_2_O, C_18_ column, 2.0 mL/min) obtained **17** (22.3 mg, *t*_R_ = 47.5 min). Fraction C3-1-2 (2.15 g) was further separated by MPLC over reversed phase silica gel (C_18_), eluting with a gradient of increasing MeOH (0−80%) in H_2_O, to afford C3-1-2-1−C3-1-2-6, of which C3-1-2-1 (840 mg) was chromatographed over Sephadex LH-20 (MeOH) to yield C3-1-2-1-1 and C3-1-2-1-2. Isolation of C3-1-2-1-2 (402 mg) by preparative TLC (CHCl_3_–MeOH, 20:1) afforded **15** (350 mg). Fraction C3-1-2-2 (100 mg) was separated by preparative TLC (CHCl_3_–MeOH, 10:1) to give C3-1-2-2-1−C3-1-2-2-3. Purification of C3-1-2-2-1 (32 mg) by RP HPLC (55% MeOH in H_2_O, C_18_ column, 2.0 mL/min) to afford **13** (24 mg, *t*_R_ = 38.2 min). Isolation of C3-1-2-3 (5.3 g) by silica gel CC (CHCl_3_-MeOH, 25:1) afforded **18** (400 mg). Fraction C3-1-3 (900 mg) was separated by silica gel CC (CHCl_3_–MeOH, 15:1) to give C3-1-3-1−C3-1-3-7, of which C3-1-3-5 (240 mg) was further isolated by silica gel CC, eluting with a gradient of increasing acetone in petroleum ether (20:1−5:1), to yielded C3-1-3-5-1−C3-1-3-5-4. Fraction C3-1-3-5-3 (8.5 mg) was successively separated by RP-HPLC (60% MeCN in H_2_O, C_18_ column, 2.0 mL/min) and chiral HPLC (hexane–isopropanol, 3:1, AD-H column, 2.0 mL/min) to obtain **9** (1.2 mg, *t*_R_ = 29.0 min) and **10** (1.5 mg, *t*_R_ = 27.0 min). Fraction C3-1-3-6 (180 mg) was separated by silica gel CC (petroleum ether-acetone, 10:1–5:1) to afford C3-1-3-6-1−C3-1-3-6-4, of which C3-1-3-6-2 (16.5 mg) isolated by preparative TLC (petroleum ether-acetone, 2:1) and purified by RP-HPLC (50% MeCN in H_2_O, Ph column, 2.0 mL/min) to yield **19** (11.5 mg, *t*_R_ = 48.2 min). Isolation of C3-1–4 (1.25 g) by silica gel CC (CHCl_3_–MeOH, 10:1–1:1) yielded C3-1-4-1−C3-1-4-4, of which C3-1-4-1 (68 mg) was separated by preparative TLC (petroleum ether-acetone, 2:1) and further purified by RP-HPLC (45% MeCN in H_2_O, C_18_ column, 2.0 mL/min) to obtain **5** (33.2 mg, *t*_R_ = 41.5 min). Separation of C3-1-4-3 (230 mg) by CC over Sephadex LH-20 (CHCl_3_–MeOH, 1:1) gave C3-1-4-3-1−C3-1-4-3-3, of which C3-1-4-3-1 (46 mg) was further separated by RP-HPLC (59% MeOH in H_2_O, C_18_ column, 2.0 mL/min) to afford **7** (22 mg, *t*_R_ = 60.5 min) and **8** (12.7 mg, *t*_R_ = 42 min).

Fraction C4 (7 g) was isolated by silica gel CC, eluting with a gradient of increasing acetone (0−100%) in petroleum ether, to yield fractions C4-1−C4-18. Fractionation of C4-3 (29 mg) by CC over Sephadex LH-20 (petroleum ether–CH_2_Cl_2_–MeOH, 5:5:1) gave C4-3-1‒C4-3-2, of which C4-3-1 (11.1 mg) was purified by RP HPLC (80% MeOH in H_2_O, C_18_ column, 2.0 mL/min) to yield **12** (4.2 mg, *t*_R_ = 12.3 min). Separation of C4-8 by CC over Sephadex LH-20 (petroleum ether–CH_2_Cl_2_–MeOH, 5:5:1) yielded C4-8-1−C4-8-3, of which C4-8-1 (13 mg) was isolated by RP HPLC (70% MeOH in H_2_O, C_18_ column, 2.0 mL/min) to give C4-8-1-1 and C4-8-1-2. Further purification of C4-8-1-1 (7 mg) by RP HPLC (50% MeOH in H_2_O, C_18_ column, 2.0 mL/min) obtained **1** (3.4 mg, *t*_R_ = 12.1 min). Isolation of C4-9 (597 mg) by CC over Sephadex LH-20 (petroleum ether–CH_2_Cl_2_–MeOH, 5:5:1) gave C4-9-1‒C4-9-4, of which C4-9-2 (20.0 mg) was purified by RP HPLC (56% MeOH in H_2_O, C_18_ column, 2.0 mL/min) to obtain **16** (16.6 mg, *t*_R_ = 24.1 min). Separation of C4-10 (95 mg) by CC over Sephadex LH-20 (petroleum ether–CH_2_Cl_2_–MeOH, 5:5:1) yielded C4-10-1 and C4-10-2, of which C4-10-1 (7 mg) was isolated by preparative TLC over silica gel (petroleum ether-acetone, 1:1) then purified by RP HPLC (70% MeOH in H_2_O, C_18_ column, 2.0 mL/min) to afford **20** (3.2 mg, *t*_R_ = 24.2 min).

Gastrodibenzin A (**1**): white amorphous powder (MeOH); UV (MeOH) *λ*_max_ (log *ε*) 205 (3.03), 233 (2.51), 284 (2.00) nm; IR *ν*_max_ 3392, 2975, 1681, 1614, 1514, 1444, 1376, 1206, 1143, 1101, 845, 803, 725 cm^–1^; ^1^H NMR (acetone-*d*_6_, 600 MHz) and ^13^C NMR (acetone-*d*_6_, 150 MHz) data, see Table 1; (+)-ESIMS: *m*/*z* 311 [M + Na]^+^; (−)-ESIMS: *m*/*z* 288 [M − H]^−^; (+)-HRESIMS: *m*/*z* 311.1254 [M + Na]^+^ (calcd. for C_17_H_20_O_4_Na, 311.1254).

Gastrodibenzin B (**2**): yellowish amorphous powder (MeOH); UV (MeOH) *λ*_max_ (log *ε*) 210 (3.58), 238 (3.57), 283 (3.30), 322 (3.10); IR *ν*_max_ 3218, 2730, 1675, 1597, 1514, 1450, 1417, 1372, 1299, 1241, 1202, 1144, 1100, 1046, 1023, 991, 888, 825, 803, 762, 724, 623 cm^−1^; ^1^H NMR (DMSO-*d*_6_, 600 MHz) and ^13^C NMR (DMSO-*d*_6_, 150 MHz) data, see Table 1; (−)-ESIMS: *m*/*z* 243 [M − H]^−^, 487 [2 M − H]^−^; ( +)-HRESIMS: *m*/*z* 245.0807 [M + H]^+^ (calcd. for C_14_H_13_O_4_, 245.0808), 267.0625 [M + Na]^+^ (calcd. for C_14_H_12_O_4_Na, 267.0628), 283.0362 [M + K]^+^ (calcd. for C_14_H_12_O_4_K, 283.0367).

Gastrodibenzin C (**3**): Brownish amorphous powder (MeOH); UV (MeOH) *λ*_max_ (log *ε*) 205 (3.85), 229 (3.83), 286 (3.53), 315 (3.37); IR *ν*_max_ 3354, 2844, 2731, 1722, 1663, 1593, 1513, 1446, 1370, 1303, 1222, 1142, 1102, 1016, 980, 917, 868, 834, 783, 745, 699, 627, 587 cm^−1^; ^1^H NMR (acetone-*d*_6_, 500 MHz) and ^13^CNMR (acetone-*d*_6_, 125 MHz) data, see Table 1; (−)-ESIMS: *m*/*z* 243 [M − H]^−^, 487 [2 M − H]^−^; (+)-HRESIMS: *m*/*z* 245.0805 [M + H]^+^ (calcd. for C_14_H_13_O_4_, 245.0808), 267.0620 [M + Na]^+^ (calcd. for C_14_H_12_O_4_Na, 267.0628).

Gastrodibenzin D (**4**): white amorphous powder (MeOH); UV(MeOH) *λ*_max_ (log *ε*) 207 (4.03), 227 (3.58), 276 (3.21) nm; IR *ν*_max_ 3036, 2975, 2930, 2867, 1729, 1613, 1586, 1513, 1466, 1378, 1353, 1301, 1227, 1172, 1106, 1002, 937, 872, 825, 748, 709, 659, 598, 519 cm^−1^; ^1^H NMR (DMSO-*d*_6_, 500 MHz) and ^13^C NMR (DMSO-*d*_6_, 125 MHz) data, see Table 1; (+)-ESIMS: *m*/*z* 339 [M + Na]^+^; ( +)-HRESIMS: *m*/*z* 339.1572 [M + Na]^+^ (calcd. for C_19_H_24_O_4_Na, 339.1567).

Gastrotribenzin A (**5**): white amorphous powder (MeOH); UV (MeOH) *λ*_max_ (log *ε*) 205 (3.93), 226 (3.48), 281 (3.04) nm; IR *ν*_max_ 3282, 3018, 2927, 2828, 2728, 2607, 2257, 2127, 1891, 1611, 1511, 1440, 1376, 1264, 1173, 1107, 1076, 1022, 1001, 949, 911, 879, 825, 780, 707, 648, 620, 532 cm^−1^; ^1^H NMR (DMSO-*d*_6_, 500 MHz) and ^13^C NMR (DMSO-*d*_6_, 125 MHz) data, see Table 2; (+)-ESIMS: *m*/*z* 373 [M + Na]^+^, 389 [M + K]^+^; (+)-HRESIMS: *m*/*z* 373.1397 [M + Na]^+^ (calcd. for C_22_H_22_O_4_Na, 373.1410).

Gastrotribenzin B (**6**): White amorphous powder (MeOH); UV (MeOH) *λ*_max_ (log *ε*) 206 (4.09), 229 (3.51), 279 (3.12) nm; IR *ν*_max_ 3385, 3013, 2918, 2851, 1612, 1512, 1476, 1441, 1381, 1346, 1257, 1216, 1174, 1139, 1074, 1018, 995, 952, 911, 877, 830, 788, 770, 720, 596, 543, 514 cm^−1^; ^1^H NMR (DMSO-*d*_6_, 500 MHz) and ^13^C NMR (DMSO-*d*_6_, 125 MHz) data, see Table 2; (+)-ESIMS: *m*/*z* 389 [M + K]^+^; (+)-HRESIMS: *m*/*z* 373.1410 [M + Na]^+^ (calcd. for C_22_H_22_O_4_Na, 373.1410).

Gastrotribenzin C (**7**): white amorphous powder (MeOH); UV (MeOH) *λ*_max_ (log *ε*) 206 (4.13), 225 (3.59), 280 (3.19); IR *ν*_max_ 3357, 2977, 1612, 1598, 1513, 1477, 1445, 1373, 1355, 1228, 1172, 1140, 1098, 1070, 1013, 980, 911, 868, 833, 789, 736 cm^−1^; ^1^H NMR (acetone-*d*_6_, 600 MHz) and ^13^C NMR (acetone-*d*_6_, 150 MHz) data, see Table 2; (+)-ESIMS: *m*/*z* 387 [M + Na]^+^, 403 [M + K]^+^, (−)-ESIMS: *m*/*z* 727 [2 M − H]^−^; ( +)-HRESIMS: *m*/*z* 387.1566 [M + Na]^+^ (calcd. for C_23_H_24_O_4_Na, 387.1567), 403.1313 [M + K]^+^ (calcd. for C_23_H_24_O_4_K, 403.1306).

Gastrotribenzin D (**8**): white amorphous powder (MeOH); UV (MeOH) *λ*_max_ (log *ε*) 205 (4.42), 227 (4.12), 282 (3.57); IR *ν*_max_ 3379, 3020, 2975, 2926, 1673, 1510, 1440, 1354, 1244, 1173, 1108, 1072, 1013, 893, 819, 781 cm^−1^; ^1^H NMR (acetone-*d*_6_, 600 MHz) and ^13^C NMR (acetone-*d*_6_, 150 MHz) data, see Table 2; (+)-ESIMS: *m*/*z* 387 [M + Na]^+^, 403 [M + K]^+^, (−)-ESIMS: *m*/*z* 363 [M − H]^−^; (+)-HRESIMS: *m*/*z* 387.1572 [M + Na]^+^ (calcd. for C_23_H_24_O_4_Na, 387.1567), 403.1314 [M + K]^+^ (calcd. for C_23_H_24_O_4_K, 403.1306).

Gastrotribenzin E (**9**): white amorphous powder (MeOH); UV (MeOH) *λ*_max_ (log *ε*) 204 (4.19), 228 (3.98), 279 (3.19); IR *ν*_max_ 3374, 3264, 3020, 2924, 2852, 1704, 1647, 1612, 1512, 1442, 1375, 1301, 1231, 1173, 1108, 1071, 1006, 941, 914, 894, 823, 781 cm^−1^; ^1^H NMR (acetone-*d*_6_, 600 MHz) spectroscopic data (Table 2); ^13^C NMR (acetone-*d*_6_, 150 MHz) spectroscopic data (Table 2); (+)-ESIMS: *m*/*z* 387 [M + Na]^+^, 403 [M + K]^+^; (+)-HRESIMS: *m*/*z* 387.1563 [M + Na]^+^ (Calcd. for C_23_H_24_O_4_Na, 387.1567).

Gastrotribenzin F (**10**): white amorphous powder (MeOH); UV (MeOH) *λ*_max_ (log *ε*) 204 (4.19), 228 (3.98), 279 (3.19); IR *ν*_max_ 3361, 3228, 3028, 2926, 2855, 1704, 1660, 1612, 1511, 1443, 1375, 1230, 1172, 1109, 1071, 1008, 941, 913, 873, 822, 776 cm^−1^; ^1^H NMR (acetone-*d*_6_, 600 MHz) and ^13^C NMR (acetone-*d*_6_, 150 MHz) data, see Table 2; (+)-ESIMS: *m/z* 387 [M + Na]^+^, 403 [M + K]^+^; (+)-HRESIMS: *m*/*z* 387.1564 [M + Na]^+^ (calcd. for C_23_H_24_O_4_Na, 387.1567).

4-[4′-(4″-Hydroxybenzyloxy) benzyloxy]benzyl ethyl ether (**11**): white amorphous powder (MeOH); UV(MeOH) *λ*_max_ (log *ε*) 205 (4.13), 224 (3.55), 280 (3.32) nm; IR *ν*_max_ 3348, 3070, 2976, 2898, 2860, 2811, 1894, 1614, 1584, 1518, 1460, 1418, 1390, 1354, 1309, 1257, 1121, 1080, 1045, 1012, 955, 897, 872, 825, 764, 708, 616, 568, 518 cm^−1^; ^1^H NMR (DMSO-*d*_6_, 500 MHz) and ^13^C NMR (DMSO-*d*_6_, 125 MHz) data, see Table 2; (+)-ESIMS: *m/z* 403 [M + K]^+^; (+)-HRESIMS: *m*/*z* 387.1566 [M + Na]^+^ (calcd. for C_23_H_24_O_4_Na, 387.1567), 403.1308 [M + K]^+^ (calcd. for C_23_H_24_O_4_K, 403.1306).

### Isolation of Products from the Refluxed Aqueous Solution of *p*-Hydroxybenzyl Alcohol

*p*-Hydroxybenzyl alcohol (10 g) were refluxed in water (250 mL) for 40 h, then the solution was concentrated under reduced pressure to yield a residue. The residue was chromatographed over reversed phase silica gel (C_18_, 300 g) with a gradient elution increasing MeCN in H_2_O (5−100%) to afford subfractions Fr.1-1−Fr.1-25 based on TLC and UPLC analysis. Fraction Fr.1-2 (60 mg) was further separated by PTLC (petroleum ether–EtOAc, 3:1) to yield **26** (2.5 mg). Isolation of Fr.1-3 (1.1 g) by CC over silica gel (MeOH-CHCl_2_, 20:1) afforded **13a** (1.0 g) and a mixture. The mixture was separated by PTLC (CH_2_Cl_2_–MeOH, 15:1) to give 4-hydroxybenzaldehyde (10.2 mg), **14a** (63.6 mg), **24** (2.0 mg), and **25** (3.4 mg). Fr.1-4 (112 mg) was chromatographed over silica gel (MeOH–CHCl_2_, 30:1−10:1) to yield Fr.1-4-1 and Fr.1-4-2, which were separately isolated by PTLC (CH_2_Cl_2_–MeOH, 10:1) to afford **5a** (2.6 mg), **6a** (2.8 mg), **15** (2.6 mg), and Fr.1-4-1-1 from the former and **18** (26 mg) and **21** (22 mg) from the latter. Fr.1-4-1-1 was further separated by RP-HPLC (ph column, 60% MeCN in H_2_O, 2.0 mL/min) gave **17** (32 mg) and **19** (3.4 mg). Reversed-phase (C_18_) flash chromatography of Fr.1-5 (156 mg) yielded subfractions Fr. 1-5-1−Fr.1-5-6, of which of which Fr. 1-5-1 (15 mg) and Fr. 1-5-2 (20.0 mg) were separately isolated by RP-HPLC (PBT column, 64% MeCN in H_2_O, 2.0 mL/min) to yield **22** (6.1 mg) from the former and **8a** (10.3 mg) and **23** (7.1 mg) from the latter. The measured spectroscopic data of the isolated compounds were identical to those reported for 4-hydroxybenzaldehyde [82], 4-hydroxy-3-(4′-hydroxybenzyl)benzyl alcohol (**13a**) [90], 4-(4′-hydroxybenzyl)-2-hydroxymethylphenol (**14a**) [91], bis(4-hydroxybenzyl)ether (**15**) [82], 4,4′-methylenediphenol (**17**) [82], 2,4-bis(4-hydroxybenzyl)phenol (**18**) [84], gastropolybenzylol A (**19**) [8], gastrol (**21**) [85], 4-hydroxy-3-(4′-hydroxy-3′-hydroxymethylbenzyl)benzyl alcohol (**24**) [91], and 4-hydroxy-3-(4′-hydroxybenzyl)benzaldehyde (**25**) [71], respectively. The structures of the new compounds **5a**, **6a**, **8a**, **22**, and **23** were determined by analysis of spectroscopic data (see below) including 2D NMR spectroscopic data (Fig. 2 and Supporting Information Figs. 133−177).

4-Hydroxy-3-[4′-hydroxy-3′-(4″-hydroxybenzyl)benzyl] benzyl alcohol (**5a**): white amorphous powder (MeOH); UV (MeOH) *λ*_max_ (log *ε*) 204 (3.94), 223 (3.58), 281 (3.00); IR *ν*_max_ 3351, 3020, 2960, 2920, 2850, 1611, 1539, 1510, 1440, 1365, 1259, 1174, 1106, 1033, 821, 706 cm^−1^; ^1^H NMR (acetone-*d*_6_, 600 MHz) and ^13^C NMR (acetone-*d*_6_, 150 MHz) data, see Table 3; (−)-HRESIMS: *m*/*z* 335.1295 [M − H]^−^ (calcd. for C_21_H_19_O_4_, 335.1278), 371.1061 [M + Cl]^−^ (calcd. for C_21_H_20_O_4_Cl, 371.1045).

4-Hydroxy-3,5-di-(4-hydroxybenzyl)benzyl alcohol (**6a**): white amorphous powder (MeOH); UV (MeOH) *λ*_max_ (log *ε*) 205 (4.09), 224 (3.84), 279 (3.12); IR *ν*_max_ 3490, 3436, 3352, 3209, 2917, 2885, 2858, 1610, 1601, 1512, 1472, 1445, 1380, 1350, 1311, 1293, 1271, 1248, 1217, 1171, 1135, 1106, 1017, 975, 955, 914, 899, 875, 824, 793, 779, 726 cm^−1^; ^1^H NMR (acetone-*d*_6_, 600 MHz) and ^13^C NMR (acetone-*d*_6_, 150 MHz) data, see Table 3; (−)-HRESIMS: *m*/*z* 335.1293 [M − H]^−^ (calcd. for C_21_H_19_O_4_, 335.1278), 371.1058 [M + Cl]^−^ (calcd. for C_21_H_20_O_4_Cl, 371.1045).

2-Hydroxy-5-[4′-hydroxy-3′-(4″-hydroxybenzyl)benzyl]benzyl alcohol (**8a**): white amorphous powder (MeOH); UV (MeOH) *λ*_max_ (log *ε*) 203 (4.07), 236 (3.37), 282 (3.10), 290 (3.17); IR *ν*_max_ 3320, 3230, 3022, 2956, 2911, 2842, 2710, 2593, 2484, 1644, 1613, 1511, 1437, 1361, 1264, 1243, 1201, 1155, 1140, 1120, 1103, 987, 976, 937, 913, 842, 815, 800, 772, 645 cm^−1^; ^1^H NMR (acetone-*d*_6_, 600 MHz) and ^13^C NMR (acetone-*d*_6_, 150 MHz) data, see Table 3; (−)-HRESIMS: *m*/*z* 335.1294 [M − H]^−^ (calcd. for C_21_H_19_O_4_, 335.1278), 371.1058 [M + Cl]^−^ (calcd. for C_21_H_20_O_4_Cl, 371.1045).

2-Hydroxy-3,5-di-[4-hydroxybenzyl]benzyl alcohol (**22**): white amorphous powder (MeOH); UV (MeOH) *λ*_max_ (log *ε*) 204 (4.22), 236 (3.24), 291 (2.96); IR *ν*_max_ 3330, 3021, 2913, 2842, 2709, 2608, 2494, 1673, 1612, 1600, 1513, 1481, 1448, 1366, 1227, 1173, 1143, 1102, 1043, 1013, 961, 883, 831, 775 cm^−1^; ^1^H NMR (acetone-*d*_6_, 600 MHz) and ^13^C NMR (acetone-*d*_6_, 150 MHz) data, see Table 3; (−)-HRESIMS: *m*/*z* 335.1294 [M − H]^−^ (calcd. for C_21_H_19_O_4_, 335.1278), 371.1060 [M + Cl]^−^ (calcd. for C_21_H_20_O_4_Cl, 371.1045).

2-Hydroxy-5-[2′-hydroxy-5′-(4″-hydroxybenzyl)benzyl]benzyl alcohol (**23**): white amorphous powder (MeOH); UV (MeOH) *λ*_max_ (log *ε*) 203 (4.18), 236 (3.64), 282 (3.40), 290 (3.40); IR *ν*_max_ 3516, 3452, 3372, 3179, 3019, 2928, 2898, 2841, 2711, 2605, 1650, 1608, 1506, 1444, 1428, 1370, 1297, 1253, 1205, 1157, 1143, 1124, 1106, 978, 961, 934, 917, 880, 841, 818, 773 cm^−1^; ^1^H NMR (acetone-*d*_6_, 600 MHz) and ^13^C NMR (acetone-*d*_6_, 150 MHz) data, see Table 3; (−)-HRESIMS: *m*/*z* 335.1293 [M − H]^−^ (calcd. for C_21_H_19_O_4_, 335.1278), 371.1059 [M + Cl]^−^ (calcd. for C_21_H_20_O_4_Cl, 371.1045).

### Preparation and UPLC-HRESIMS Analysis of the *p*-Hydroxybenzyl Alcohol, **15** and **21** Solutions

*p*-Hydroxybenzyl alcohol (each 25.0 mg, purchased from Beijing Ouhe Technology CO., LTD), was dissolved in round-bottom flasks with 25.0 mL of H_2_O, MeOH, EtOH, respectively, and compound **15** (each 1.0 mg) or **21** (each 1.0 mg) was dissolved with 1.0 mL of the solvents. Two parallel experiments were set for each compound and solvent. The solutions were ultrasonicated (280 W) for 0.5 h, then heated in a liquid alloy bath to reflux. Two parallel experiments were set for each solvent. The solutions (each 50 μL) were sampled after ultrasonicated and at refluxing times of 0.5 h, 1.0 h, 1.5 h, 2.0 h, 4.0 h, and 6.0 h, respectively. Each the sampled H_2_O solution was diluted with MeCN to 1.0 mL. The sampled MeOH and EtOH extracts were diluted with MeOH and EtOH to 1.0 mL, respectively. The diluted samples were individually filtrated and the filtrates were analyzed by UPLC-HRESIMS under following conditions: Q Exactive Focus LC–MS/MS spectrometer; ACQUITY UPLC BEH C_18_ column (1.7 μm, 2.1 × 100 mm); temperature, 25 °C; flow rate, 0.4 mL/min; gradient elution of increasing CH_3_CN in H_2_O from 5 to 45% in 15.0 min then to 100% in 1.0 min.

### Preparation and UPLC/HR-ESI–MS Analysis of the Rresh *G. elata* Rhizomes and “tian ma” Extracts

The fresh *G. elata* rhizomes and “tian ma” were cut into small pieces, respectively. The pieces of plant materials (each 12.0 g) were ultrasonicated (280 W) in round-bottom flasks with 30 mL of H_2_O, MeOH, and EtOH for 0.5 h, respectively, followed by heating in a liquid alloy bath to reflux. Two parallel experiments were set for each the plant material and solvent. The extracts (each 400 μL) were sampled after ultrasonicated and at refluxing times of 0.5 h, 1.0 h, 1.5 h, 2.0 h, 4.0 h, and 6.0 h, respectively. Each the sampled H_2_O extract was diluted with 65% MeCN in H_2_O to 1.0 mL. The sampled MeOH and EtOH extracts were diluted with MeOH and EtOH to 1.0 mL, respectively. The diluted samples were individually filtrated and the filtrates were analyzed by UPLC-HRESIMS under the above described conditions, respectively.

### Protective Assay Against Rotenone-induced PC12 Cell Damage

See Ref.[79].

### Protective Assay Against dl-Galactosamine (GalN)-induced WB-F344 Cell Damage

See Ref.[96].

### Inhibitory Assay Against LPS-induced NO Production in Mouse Peritoneal Macrophages

See Ref.[97].

#### Inhibitory Assay Against Fe^2+^-cystine-induced Rat Liver Microsomal Lipid Peroxidation

See Refs.[72, 73].

## Electronic supplementary material

Below is the link to the electronic supplementary material.

Supplementary file1 (PDF 30353 kb)
