# Supplementary material for: Insight into Medicinal Chemistry Behind Traditional Chinese Medicines: p-Hydroxybenzyl Alcohol-Derived Dimers and Trimers from Gastrodia elata
Source: Nat Prod Bioprospect. 2020 Aug 6;11(1):31–50. doi: 10.1007/s13659-020-00258-w (PMC7933327; doi:10.1007/s13659-020-00258-w)
Supplement: Supplementary file 1 — Supplementary file1 (PDF 30353 kb) [file 13659_2020_258_MOESM1_ESM.pdf]

Insight into medicinal chemistry behind traditional Chinese medicines: *p*-hydroxybenzyl alcohol-derived dimers and trimers from *Gastrodia elata*

Yanan Wang†, Min Zhang†, Xue Zhou, Chengbo Xu, Chenggen Zhu, Yuhe Yuan, Naihong Chen, Yongchun Yang, Qinglan Guo*, and Jiangong Shi*

State Key Laboratory of Bioactive Substance and Function of Natural Medicines, Institute of Materia Medica, Chinese Academy of Medical Sciences and Peking Union Medical College, Beijing 100050, China

Supplementary Material

* Corresponding author.

Tel.: 86-10-63025166

Fax: 86-10-63017757

E-mail: shijg@imm.ac.cn (Jian-Gong Shi).

We dedicate this paper to Prof. Zhou Jun in commemoration of his life-time contribution to researches in plant resource and phytochemistry.

†These authors made equal contributions to this work.

List of Content

1	**General experimental procedures**	S10
2	**Plant material**	S11
3	**Preliminary extraction and isolation**	S12
4	**Fig. S1** The UV spectrum of compound **1**	S13
5	**Fig. S2** The IR spectrum of compound **1**	S14
6	**Fig. S3** The ESI-MS of compound **1**	S15
7	**Fig. S4** The (+)-HR-ESI-MS report of compound **1**, page 1	S15
8	**Fig. S5** The (+)-HR-ESI-MS report of compound **1**, page 2	S16
9	**Fig. S6** The (+)-HR-ESI-MS report of compound **1**, page 3	S17
10	**Fig. S7** The ^{1}H NMR spectrum of compound **1** in acetone-d_6 (600 MHz)	S18
11	**Fig. S8** The ^{13}C NMR spectrum of compound **1** in acetone-d_6 (150 MHz)	S19
12	**Fig. S9** The DEPT spectrum of compound **1** in acetone-d_6 (150 MHz)	S20
13	**Fig. S10** The ^{1}H-^{1}H COSY spectrum of compound **1** in acetone-d_6 (600 MHz)	S21
14	**Fig. S11** The HSQC spectrum of compound **1** in acetone-d_6 (600 MHz for ^{1}H)	S22
15	**Fig. S12** The HMBC spectrum of compound **1** in acetone-d_6 (600 MHz for ^{1}H)	S23
16	**Fig. S13** The UV spectrum of compound **2**	S24
17	**Fig. S14** The IR spectrum of compound **2**	S25
18	**Fig. S15** The ESI-MS of compound **2**	S26
19	**Fig. S16** The (+)-HR-ESI-MS report of compound **2**, page 1	S27
20	**Fig. S17** The (+)-HR-ESI-MS report of compound **2**, page 2	S28
21	**Fig. S18** The (+)-HR-ESI-MS report of compound **2**, page 3	S29
22	**Fig. S19** The ^{1}H NMR spectrum of compound **2** in DMSO-d_6 (600 MHz)	S30
23	**Fig. S20** The ^{13}C NMR spectrum of compound **2** in DMSO-d_6 (150 MHz)	S31
24	**Fig. S21** The DEPT spectrum of compound **2** in DMSO-d_6 (150 MHz)	S32
25	**Fig. S22** The ^{1}H-^{1}H COSY spectrum of compound **2** in DMSO-d_6 (600 MHz)	S33
26	**Fig. S23** The HSQC spectrum of compound **2** in DMSO-d_6 (600 MHz for ^{1}H)	S34
27	**Fig. S24** The HMBC spectrum of compound **2** in DMSO-d_6 (600 MHz for ^{1}H)	S35
28.	**Fig. S25** The UV spectrum of compound **3**	S36
29	**Fig. S26** The IR spectrum of compound **3**	S37
30	**Fig. S27** The ESI-MS of compound **3**	S38
31	**Fig. S28** The (+)-HR-ESI-MS report of compound **3**, page 1	S39
32	**Fig. S29** The (+)-HR-ESI-MS report of compound **3**, page 2	S40
33	**Fig. S30** The (+)-HR-ESI-MS report of compound **3**, page 3	S41
34	**Fig. S31** The ^{1}H NMR spectrum of compound **3** in acetone-d_6 (500 MHz)	S42
35	**Fig. S32** The ^{13}C NMR spectrum of compound **3** in acetone-d_6 (125 MHz)	S43
36	**Fig. S33** The DEPT spectrum of compound **3** in acetone-d_6 (125 MHz)	S44
37	**Fig. S34** The ^{1}H-^{1}H COSY spectrum of compound **3** in acetone-d_6 (500 MHz)	S45
38	**Fig. S35** The HSQC spectrum of compound **3** in acetone-d_6 (500 MHz for ^{1}H)	S46
39	**Fig. S36** The HMBC spectrum of compound **3** in acetone-d_6 (500 MHz for ^{1}H)	S47
40	**Fig. S37** The UV spectrum of compound **4**	S48
41	**Fig. S38** The IR spectrum of compound **4**	S49
42	**Fig. S39** The ESI-MS of compound **4**	S50
43	**Fig. S40** The (+)-HR-ESI-MS report of compound **4**, page 1	S51
44	**Fig. S41** The (+)-HR-ESI-MS report of compound **4**, page 2	S52
45	**Fig. S42** The (+)-HR-ESI-MS report of compound **4**, page 3	S53
46	**Fig. S43** The ^{1}H NMR spectrum of compound **4** in DMSO-d_6 (500 MHz)	S54
47	**Fig. S44** The ^{13}C NMR spectrum of compound **4** in DMSO-d_6 (125 MHz)	S55
48	**Fig. S45** The DEPT spectrum of compound **4** in DMSO-d_6 (125 MHz)	S56
49	**Fig. S46** The ^{1}H-^{1}H COSY spectrum of compound **4** in DMSO-d_6 (500 MHz)	S57
50	**Fig. S47** The HSQC spectrum of compound **4** in DMSO-d_6 (500 MHz for ^{1}H)	S58
51	**Fig. S48** The HMBC spectrum of compound **4** in DMSO-d_6 (500 MHz for ^{1}H)	S59
52	**Fig. S49** The UV spectrum of compound **5**	S60
53	**Fig. S50** The IR spectrum of compound **5**	S61

54 **Fig. S51** The ESI-MS of compound **5** S62
55 **Fig. S52** The (+)-HR-ESI-MS report of compound **5**, page 1 S63
56 **Fig. S53** The (+)-HR-ESI-MS report of compound **5**, page 2 S64
57 **Fig. S54** The (+)-HR-ESI-MS report of compound **5**, page 3 S65
58 **Fig. S55** The ^{1}H NMR spectrum of compound **5** in DMSO-d_6 (500 MHz) S66
59 **Fig. S56** The ^{13}C NMR spectrum of compound **5** in DMSO-d_6 (125 MHz) S67
60 **Fig. S57** The DEPT spectrum of compound **5** in DMSO-d_6 (125 MHz) S68
61 **Fig. S58** The ^{1}H-^{1}H COSY spectrum of compound **5** in DMSO-d_6 (500 MHz) S69
62 **Fig. S59** The HSQC spectrum of compound **5** in DMSO-d_6 (500 MHz for ^{1}H) S70
63 **Fig. S60** The HMBC spectrum of compound **5** in DMSO-d_6 (500 MHz for ^{1}H) S71
64 **Fig. S61** The UV spectrum of compound **6** S72
65 **Fig. S62** The IR spectrum of compound **6** S73
66 **Fig. S63** The ESI-MS of compound **6** S74
67. **Fig. S64** The (+)-HR-ESI-MS report of compound **6**, page 1 S75
68 **Fig. S65** The (+)-HR-ESI-MS report of compound **6**, page 2 S76
69 **Fig. S66** The (+)-HR-ESI-MS report of compound **6**, page 3 S77
70 **Fig. S67** The ^{1}H NMR spectrum of compound **6** in DMSO-d_6 (500 MHz S78
71 **Fig. S68** The ^{13}C NMR spectrum of compound **6** in DMSO-d_6 (125 MHz) S79
72 **Fig. S69** The DEPT spectrum of compound **6** in DMSO-d_6 (125 MHz) S80
73 **Fig. S70** The ^{1}H-^{1}H COSY spectrum of compound **6** in DMSO-d_6 (500 MHz) S81
74 **Fig. S71** The HSQC spectrum of compound **6** in DMSO-d_6 (500 MHz for ^{1}H) S82
75 **Fig. S72** The HMBC spectrum of compound **6** in DMSO-d_6 (500 MHz for ^{1}H) S83
76 **Fig. S73** The UV spectrum of compound **7** S84
77 **Fig. S74** The IR spectrum of compound **7** S85
78 **Fig. S75** The ESI-MS of compound **7** S86
79 **Fig. S76** The (+)-HR-ESI-MS report of compound **7**, page 1 S87
80 **Fig. S77** The (+)-HR-ESI-MS report of compound **7**, page 2 S88
81 **Fig. S78** The (+)-HR-ESI-MS report of compound **7**, page 3 S89
82 **Fig. S79** The ^{1}H NMR spectrum of compound **7** in acetone-d_6 (600 MHz) S90
83 **Fig. S80** The ^{13}C NMR spectrum of compound **7** in acetone-d_6 (150 MHz) S91
84 **Fig. S81** The DEPT spectrum of compound **7** in acetone-d_6 (125 MHz) S92
85 **Fig. S82** The ^{1}H-^{1}H COSY spectrum of compound **7** in acetone-d_6 (600 MHz) S93
86 **Fig. S83** The HSQC spectrum of compound **7** in acetone-d_6 (600 MHz for ^{1}H) S94
87 **Fig. S84** The HMBC spectrum of compound **7** in acetone-d_6 (600 MHz for ^{1}H) S95
88 **Fig. S85** The UV spectrum of compound **8** S96
89 **Fig. S86** The IR spectrum of compound **8** S97
90 **Fig. S87** The ESI-MS of compound **8** S98
91 **Fig. S88** The (+)-HR-ESI-MS report of compound **8**, page 1 S99
92 **Fig. S89** The (+)-HR-ESI-MS report of compound **8**, page 2 S100
93 **Fig. S90** The (+)-HR-ESI-MS report of compound **8**, page 3 S101
94 **Fig. S91** The ^{1}H NMR spectrum of compound **8** in acetone-d_6 (600 MHz) S102
95 **Fig. S92** The ^{13}C NMR spectrum of compound **8** in acetone-d_6 (150 MHz) S103
96 **Fig. S93** The DEPT spectrum of compound **8** in acetone-d_6 (150 MHz) S104
97 **Fig. S94** The ^{1}H-^{1}H COSY spectrum of compound **8** in acetone-d_6 (600 MHz) S105
98 **Fig. S95** The HSQC spectrum of compound **8** in acetone-d_6 (600 MHz for ^{1}H) S106
99 **Fig. S96** The HMBC spectrum of compound **8** in acetone-d_6 (600 MHz for ^{1}H) S107
100 **Fig. S97** The UV spectrum of compound **9** S108
101 **Fig. S98** The IR spectrum of compound **9** S109
102 **Fig. S99** The ESI-MS of compound **9** S110
103 **Fig. S100** The (+)-HR-ESI-MS report of compound **9**, Page1 S111
104 **Fig. S101** The (+)-HR-ESI-MS report of compound **9**, Page2 S112
105 **Fig. S102** The (+)-HR-ESI-MS report of compound **9**, page 3 S113
106 **Fig. S103** The ^{1}H NMR spectrum of compound **9** in acetone-d_6 (600 MHz) S114
107 **Fig. S104** The ^{13}C NMR spectrum of compound **9** in acetone-d_6 (150 MHz) S115
108 **Fig. S105** The DEPT spectrum of compound **9** in acetone-d_6 (150 MHz) S116
109 **Fig. S106** The ^{1}H-^{1}H COSY spectrum of compound **9** in acetone-d_6 (600 MHz) S117
110 **Fig. S107** The HSQC spectrum of compound **9** in acetone-d_6 (600 MHz for ^{1}H) S118
111 **Fig. S108** The HMBC spectrum of compound **9** in acetone-d_6 (600 MHz for ^{1}H) S119
112 **Fig. S109** The UV spectrum of compound **10** S120
113 **Fig. S110** The IR spectrum of compound **10** S121
114 **Fig. S111** The ESI-MS of compound **10** S122
115 **Fig. S112** The (+)-HR-ESI-MS report of compound **10**, Page1 S123
116 **Fig. S113** The (+)-HR-ESI-MS report of compound **10**, Page2 S124
117 **Fig. S114** The (+)-HR-ESI-MS report of compound **10**, page 3 S125
118 **Fig. S115** The ^{1}H NMR spectrum of compound **10** in acetone-d_6 (600 MHz) S126
119 **Fig. S116** The ^{13}C NMR spectrum of compound **10** in acetone-d_6 (150 MHz) S127
120 **Fig. S117** The DEPT spectrum of compound **10** in acetone-d_6 (150 MHz) S128
121 **Fig. S118** The ^{1}H-^{1}H COSY spectrum of compound **10** in acetone-d_6 (600 MHz) S129
122 **Fig. S119** The HSQC spectrum of compound **10** in acetone-d_6 (600 MHz for ^{1}H) S130
123 **Fig. S120** The HMBC spectrum of compound **10** in acetone-d_6 (600 MHz for ^{1}H) S131
124 **Fig. S121** The UV spectrum of compound **11** S132
125 **Fig. S122** The IR spectrum of compound **11** S133
126 **Fig. S123** The ESI-MS of compound **11** S134
127 **Fig. S124** The (+)-HR-ESI-MS report of compound **11**, page 1 S135

128 **Fig. S125** The (+)-HR-ESI-MS report of compound **11**, page 2 S136

129 **Fig. S126** The (+)-HR-ESI-MS report of compound **11**, page 3 S137

130 **Fig. S127** The ^{1}H NMR spectrum of compound **11** in DMSO-*d*$_6$ (600 MHz) S138

131 **Fig. S128** The ^{13}C NMR spectrum of compound **11** in DMSO-*d*$_6$ (150 MHz) S139

132 **Fig. S129** The DEPT spectrum of compound **11** in DMSO-*d*$_6$ (150 MHz) S140

133 **Fig. S130** The ^{1}H-^{1}H COSY spectrum of compound **11** in DMSO-*d*$_6$ (600 MHz) S141

134 **Fig. S131** The HSQC spectrum of compound **11** in DMSO-*d*$_6$ (600 MHz for ^{1}H) S142

135 **Fig. S132** The HMBC spectrum of compound **11** in DMSO-*d*$_6$ (600 MHz for ^{1}H) S143

136 **Fig. S133** The UV spectrum of compound **5a** S144

137 **Fig. S134** The IR spectrum of compound **5a** S145

138 **Fig. S135** The (–)-HR-ESI-MS report of compound **5a** S146

139 **Fig. S136** The ^{1}H NMR spectrum of compound **5a** in acetone-*d*$_6$ (600 MHz) S147

140 **Fig. S137** The ^{13}C NMR spectrum of compound **5a** in acetone-*d*$_6$ (150 MHz) S148

141 **Fig. S138** The DEPT spectrum of compound **5a** in acetone-*d*$_6$ (150 MHz) S149

142 **Fig. S139** The ^{1}H-^{1}H COSY spectrum of compound **5a** in acetone-*d*$_6$ (600 MHz) S150

143 **Fig. S140** The HSQC spectrum of compound **5a** in acetone-*d*$_6$ (600 MHz for ^{1}H) S151

144 **Fig. S141** The HMBC spectrum of compound **5a** in acetone-*d*$_6$ (600 MHz for ^{1}H) S152

145 **Fig. S142** The UV spectrum of compound **6a** S153

146 **Fig. S143** The IR spectrum of compound **6a** S154

147 **Fig. S144** The (–)-HR-ESI-MS report of compound **6a** S155

148 **Fig. S145** The ^{1}H NMR spectrum of compound **6a** in acetone-*d*$_6$ (600 MHz) S156

149 **Fig. S146** The ^{13}C NMR spectrum of compound **6a** in acetone-*d*$_6$ (150 MHz) S157

150 **Fig. S147** The DEPT spectrum of compound **6a** in acetone-*d*$_6$ (150 MHz) S158

151 **Fig. S148** The ^{1}H-^{1}H COSY spectrum of compound **6a** in acetone-*d*$_6$ (600 MHz) S159

152 **Fig. S149** The HSQC spectrum of compound **6a** in acetone-*d*$_6$ (600 MHz for ^{1}H) S160

153 **Fig. S150** The HMBC spectrum of compound **6a** in acetone-*d*$_6$ (600 MHz for ^{1}H) S161

154 **Fig. S151** The UV spectrum of compound **8a** S162

155 **Fig. S152** The IR spectrum of compound **8a** S163

156 **Fig. S153** The (–)-HR-ESI-MS report of compound **8a** S164

157 **Fig. S154** The ^{1}H NMR spectrum of compound **8a** in acetone-*d*$_6$ (600 MHz) S165

158 **Fig. S155** The ^{13}C NMR spectrum of compound **8a** in acetone-*d*$_6$ (150 MHz) S166

159 **Fig. S156** The DEPT spectrum of compound **8a** in acetone-*d*$_6$ (150 MHz) S167

160 **Fig. S157** The ^{1}H-^{1}H COSY spectrum of compound **8a** in acetone-*d*$_6$ (600 MHz) S168

161 **Fig. S158** The HSQC spectrum of compound **8a** in acetone-*d*$_6$ (600 MHz for ^{1}H) S169

162 **Fig. S159** The HMBC spectrum of compound **8a** in acetone-*d*$_6$ (600 MHz for ^{1}H) S170

163 **Fig. S160** The UV spectrum of compound **22** S171

164 **Fig. S161** The IR spectrum of compound **22** S172

165 **Fig. S162** The (–)-HR-ESI-MS report of compound **22** S173

166 **Fig. S163** The ^{1}H NMR spectrum of compound **22** in acetone-*d*$_6$ (600 MHz) S174

167 **Fig. S164** The ^{13}C NMR spectrum of compound **22** in acetone-*d*$_6$ (150 MHz) S175

168 **Fig. S165** The DEPT spectrum of compound **22** in acetone-*d*$_6$ (150 MHz) S176

169 **Fig. S166** The ^{1}H-^{1}H COSY spectrum of compound **22** in acetone-*d*$_6$ (600 MHz) S177

170 **Fig. S167** The HSQC spectrum of compound **22** in acetone-*d*$_6$ (600 MHz for ^{1}H) S178

171 **Fig. S168** The HMBC spectrum of compound **22** in acetone-*d*$_6$ (600 MHz for ^{1}H) S179

172 **Fig. S169** The UV spectrum of compound **23** S180

173 **Fig. S170** The IR spectrum of compound **23** S181

174 **Fig. S171** The (–)-HR-ESI-MS report of compound **23** S182

175 **Fig. S172** The ^{1}H NMR spectrum of compound **23** in acetone-*d*$_6$ (600 MHz) S183

176 **Fig. S173** The ^{13}C NMR spectrum of compound **23** in acetone-*d*$_6$ (150 MHz) S184

177 **Fig. S174** The DEPT spectrum of compound **23** in acetone-*d*$_6$ (150 MHz) S185

178 **Fig. S175** The ^{1}H-^{1}H COSY spectrum of compound **23** in acetone-*d*$_6$ (600 MHz) S186

179 **Fig. S176** The HSQC spectrum of compound **23** in acetone-*d*$_6$ (600 MHz for ^{1}H) S187

180 **Fig. S177** The HMBC spectrum of compound **23** in acetone-*d*$_6$ (600 MHz for ^{1}H) S188

181 **Fig. S178** Overlaid chromatograms of the extracted negative ion at *m/z* 335.130 [M–H]$^-$: (a)–(c) compounds **5a**, **6a**, and **8a** in CH_3CN, respectively; (d)–(f) H_2O, MeOH, and EtOH solutions of *p*-hydroxybenzyl alcohol were sonicated for 0.5 h then refluxed for 1.0 h, respectively S189

182 **Fig. S179** Overlaid chromatograms of the extracted negative ion at *m/z* 335.130 [M–H]$^-$: (a)–(d) compounds **20**, **21**, **22**, and **23** in CH_3CN, respectively; (e)–(g) H_2O, MeOH, and EtOH solutions of *p*-hydroxybenzyl alcohol were sonicated for 0.5 h then refluxed for 1.0 h, respectively S190

183 **Fig. S180** Overlaid chromatograms of the extracted negative ion at *m/z* 305.119 [M–H]$^-$: (a) and (b) compounds **18** and **19** in CH_3CN, respectively; (c)–(e) H_2O, MeOH, and EtOH solutions of *p*-hydroxybenzyl alcohol were sonicated for 0.5 h then refluxed for 1.0 h, respectively S191

184 **Fig. S181** Overlaid chromatograms of the extracted negative ion at *m/z* 229.087 [M–H]$^-$: (a)–(c) compounds **13a**, **14a**, and **15** in CH_3CN, respectively; (d)–(f) H_2O, MeOH, and EtOH solutions of *p*-hydroxybenzyl alcohol were sonicated for 0.5 h then refluxed for 1.0 h, respectively S192

185 **Fig. S182** Overlaid chromatograms of the extracted negative ion at *m/z* 199.077 [M–H]$^-$: (a) compound **17** in CH_3CN; (b)–(d) H_2O, MeOH, and EtOH solutions of *p*-hydroxybenzyl alcohol were sonicated for 0.5 h then refluxed for 1.0 h, respectively S193

186 **Fig. S183** Overlaid chromatograms of the extracted negative ion at *m/z* 227.072 [M–H]$^-$: (a) compound **25**; (b)–(d) H_2O, MeOH, and EtOH solutions of *p*-hydroxybenzyl alcohol were sonicated for 0.5 h then refluxed for 1.0 h, respectively. S194

187 **Fig. S184** Overlaid chromatograms of the extracted negative ion at *m/z* 335.130 [M–H]$^-$: (a)–(e) compounds **5a**/**6a**, **8a**, **21**, **22**, and **23** in CH_3CN, respectively; (f)–(h) H_2O, MeOH, and EtOH solutions of *p*-hydroxybenzyl alcohol were S195

sonicated for 0.5 h, respectively

188 **Fig. S185** Overlaid chromatograms of the extracted negative ion at *m/z* 305.119 [M−H]$^-$: (a) and (b) compound **18** and **19** in CH_3CN, respectively; (c)–(e) H_2O, MeOH, and EtOH solutions of *p*-hydroxybenzyl alcohol were sonicated for 0.5 h, respectively S196

189 **Fig. S186** Overlaid chromatograms of the extracted negative ion at *m/z* 229.087 [M−H]$^-$: (a)–(c) compounds **13a**, **14a**, and **15** in CH_3CN, respectively; (d)–(f) H_2O, MeOH, and EtOH solutions of *p*-hydroxybenzyl alcohol were sonicated for 0.5 h, respectively S197

190 **Fig. S187** Overlaid chromatograms of the extracted negative ion at *m/z* 257.119 [M−H]$^-$: (a)–(c) compounds **12**, **13**, and **14** in CH_3CN, respectively; (d)–(f) H_2O, MeOH, and EtOH solutions of *p*-hydroxybenzyl alcohol were sonicated for 0.5 h, respectively. S198

191 **Fig. S188** Overlaid chromatograms of the extracted negative ion at *m/z* 199.077 [M−H]$^-$: (a) compound **17** in CH_3CN; (b)–(d) H_2O, MeOH, and EtOH solutions of *p*-hydroxybenzyl alcohol were sonicated for 0.5 h, respectively S199

192 **Fig. S189** Overlaid chromatograms of the extracted negative ion at *m/z* 227.072 [M−H]$^-$: (a) compound **25** in CH_3CN; (b)–(d) H_2O, MeOH, and EtOH solutions of *p*-hydroxybenzyl alcohol were sonicated for 0.5 h, respectively. S200

193 **Fig. S190** Overlaid chromatograms of the extracted negative ion at *m/z* 335.130 [M−H]$^-$: (a)–(c) compounds **5a**/**6a**, **8a**, and **21** in CH_3CN, respectively; (d)–(h) H_2O solution of *p*-hydroxybenzyl alcohol was sonicated for 0.5 h then refluxed for 1.0 h, 2.0 h, 4.0 h, and 6.0 h, respectively S201

194 **Fig. S191** Overlaid chromatograms of the extracted negative ion at *m/z* 335.130 [M−H]$^-$: (a) – (c) compounds **20**, **22**, and **23** in CH_3CN, respectively; (d)–(h) H_2O solution of *p*-hydroxybenzyl alcohol was sonicated for 0.5 h then refluxed for 1.0 h, 2.0 h, 4.0 h, and 6.0 h, respectively S202

195 **Fig. S192** Overlaid chromatograms of the extracted negative ion at *m/z* 305.119 [M−H]$^-$: (a) and (b) compounds **18** and **19** in CH_3CN, respectively; (c)–(g) H_2O solution of *p*-hydroxybenzyl alcohol was sonicated for 0.5 h then refluxed for 1.0 h, 2.0 h, 4.0 h, and 6.0 h, respectively S203

196 **Fig. S193** Overlaid chromatograms of the extracted negative ion at *m/z* 229.087 [M−H]$^-$: (a)–(c) compounds **13a**, **14a**, and **15** in CH_3CN, respectively; (d)–(h) H_2O solution of *p*-hydroxybenzyl alcohol was sonicated for 0.5 h then refluxed for 1.0 h, 2.0 h, 4.0 h, and 6.0 h, respectively S204

197 **Fig. S194** Overlaid chromatograms of the extracted negative ion at *m/z* 259.098 [M−H]$^-$: (a) and (b) compounds **16** and **24** in CH_3CN, respectively; (c)–(g) H_2O solution of *p*-hydroxybenzyl alcohol was sonicated for 0.5 h then refluxed for 1.0 h, 2.0 h, 4.0 h, and 6.0 h, respectively S205

198 **Fig. S195** Overlaid chromatograms of the extracted negative ion at *m/z* 199.077 [M−H]$^-$: (a) compound **17** in CH_3CN; (b)–(f) H_2O solution of *p*-hydroxybenzyl alcohol was sonicated for 0.5 h then refluxed for 1.0 h, 2.0 h, 4.0 h, and 6.0 h, respectively S206

199 **Fig. S196** Overlaid chromatograms of the extracted negative ion at *m/z* 227.072 [M−H]$^-$: (a) compound **25** in CH_3CN; (b)–(f) H_2O solution of *p*-hydroxybenzyl alcohol was sonicated for 0.5 h then refluxed for 1.0 h, 2.0 h, 4.0 h, and 6.0 h, respectively S207

200 **Fig. S197** Overlaid chromatograms of the extracted negative ion at *m/z* 349.145 [M−H]$^-$: (a)–(c) compounds **5** and **6** in CH_3CN, respectively; (d)–(g) MeOH solution of *p*-hydroxybenzyl alcohol was sonicated for 0.5 h then refluxed for 1.0 h, 2.0 h, 4.0 h, and 6.0 h, respectively S208

201 **Fig. S198** Overlaid chromatograms of the extracted negative ion at *m/z* 335.130 [M−H]$^-$: (a)–(c) compounds **5a**/**6a**, **8a**, and **21** in CH_3CN, respectively; (d)–(h) MeOH solution of *p*-hydroxybenzyl alcohol was sonicated for 0.5 h then refluxed for 1.0 h, 2.0 h, 4.0 h, and 6.0 h, respectively S209

202 **Fig. S199** Overlaid chromatograms of the extracted negative ion at *m/z* 335.130 [M−H]$^-$: (a)–(c) compounds **20**, **22**, and **23** in CH_3CN, respectively; (d)–(h) MeOH solution of *p*-hydroxybenzyl alcohol was sonicated for 0.5 h then refluxed for 1.0 h, 2.0 h, 4.0 h, and 6.0 h, respectively S210

203 **Fig. S200** Overlaid chromatograms of the extracted negative ion at *m/z* 305.119 [M−H]$^-$**:** (a) and (b) compounds **18** and **19** in CH_3CN, respectively; (c)–(g) MeOH solution of *p*-hydroxybenzyl alcohol was sonicated for 0.5 h then refluxed for 1.0 h, 2.0 h, 4.0 h, and 6.0 h, respectively S211

204 **Fig. S201** Overlaid chromatograms of the extracted negative ion at *m/z* 229.087 [M−H]$^-$: (a)–(c) compounds **13a**, **14a**, and **15** in CH_3CN, respectively; (d)–(h) MeOH solution of *p*-hydroxybenzyl alcohol was sonicated for 0.5 h then refluxed for 1.0 h, 2.0 h, 4.0 h, and 6.0 h, respectively S212

205 **Fig. S202** Overlaid chromatograms of the extracted negative ion at *m/z* 259.098 [M−H]$^-$: (a) and (b) compounds **16** and **24** in CH_3CN, respectively; (c)–(g) MeOH solution of *p*-hydroxybenzyl alcohol was sonicated for 0.5 h then refluxed for 1.0 h, 2.0 h, 4.0 h, and 6.0 h, respectively S213

206 **Fig. S203** Overlaid chromatograms of the extracted negative ion at *m/z* 199.077 [M−H]$^-$: (a) compound **17** in CH_3CN; (b)–(f) MeOH solution of *p*-hydroxybenzyl alcohol was sonicated for 0.5 h then refluxed for 1.0 h, 2.0 h, 4.0 h, and 6.0 h, respectively S214

207 **Fig. S204** Overlaid chromatograms of the extracted negative ion at *m/z* 227.072 [M−H]$^-$: (a) compound **25** in CH_3CN; (b)–(f) MeOH solution of *p*-hydroxybenzyl alcohol was sonicated for 0.5 h then refluxed for 1.0 h, 2.0 h, 4.0 h, and 6.0 h, respectively S215

208 **Fig. S205** Overlaid chromatograms of the extracted negative ion at *m/z* 335.130 [M−H]$^-$: (a)–(c) compounds **5a**/**6a**, **8a**, and **21** in CH_3CN, respectively; (d)–(h) EtOH solution of *p*-hydroxybenzyl alcohol was sonicated for 0.5 h then refluxed for 1.0 h, 2.0 h, 4.0 h, and 6.0 h, respectively S216

209 **Fig. S206** Overlaid chromatograms of the extracted negative ion at *m/z* 335.130 [M−H]$^-$: (a)–(c) compounds **20**, **22**, and **23** in CH_3CN, respectively; (d)–(h) EtOH solution of *p*-hydroxybenzyl alcohol was sonicated for 0.5 h then refluxed for 1.0 h, 2.0 h, 4.0 h, and 6.0 h, respectively S217

210 **Fig. S207** Overlaid chromatograms of the extracted negative ion at *m/z* 363.161 [M−H]$^-$: (a)–(c) compounds **7**, **8**, and **9** in CH_3CN, respectively; (d)–(h) EtOH solution of *p*-hydroxybenzyl alcohol was sonicated for 0.5 h then refluxed for 1.0 h, 2.0 h, 4.0 h, and 6.0 h, respectively S218

211 **Fig. S208** Overlaid chromatograms of the extracted negative ion at *m/z* 305.119 [M−H]$^-$: (a) and (b) compounds **18** and **19** in CH_3CN, respectively; (c)–(g) EtOH solution of *p*-hydroxybenzyl alcohol was sonicated for 0.5 h then refluxed for 1.0 h, 2.0 h, 4.0 h, and 6.0 h, respectively S219

212 **Fig. S209** Overlaid chromatograms of the extracted negative ion at *m/z* 257.119 $[M-H]^-$: (a)–(c) compounds **12**, **13**, and **14** in CH_3CN, respectively; (d)–(h) EtOH solution of *p*-hydroxybenzyl alcohol was sonicated for 0.5 h then refluxed for 1.0 h, 2.0 h, 4.0 h, and 6.0 h, respectively S220

213 **Fig. S210** Overlaid chromatograms of the extracted negative ion at *m/z* 229.087 $[M-H]^-$: (a)–(c) compounds **13a**, **14a**, and **15** in CH_3CN, respectively; (d)–(h) EtOH solution of *p*-hydroxybenzyl alcohol was sonicated for 0.5 h then refluxed for 1.0 h, 2.0 h, 4.0 h, and 6.0 h, respectively S221

214 **Fig. S211** Overlaid chromatograms of the extracted negative ion at *m/z* 259.098 $[M-H]^-$: (a) and (b) compounds **16** and **24** in CH_3CN, respectively; (c)–(g) EtOH solution of *p*-hydroxybenzyl alcohol was sonicated for 0.5 h then refluxed for 1.0 h, 2.0 h, 4.0 h, and 6.0 h, respectively S222

215 **Fig. S212** Overlaid chromatograms of the extracted negative ion at *m/z* 199.077 $[M-H]^-$: (a) compound **17** in CH_3CN; (b)–(f) EtOH solution of *p*-hydroxybenzyl alcohol was sonicated for 0.5 h then refluxed for 1.0 h, 2.0 h, 4.0 h, and 6.0 h, respectively S223

216 **Fig. S213** Overlaid chromatograms of the extracted negative ion at *m/z* 227.072 $[M-H]^-$: (a) compound **25** in CH_3CN; (b)–(f) EtOH solution of *p*-hydroxybenzyl alcohol was sonicated for 0.5 h then refluxed for 1.0 h, 2.0 h, 4.0 h, and 6.0 h, respectively S224

217 **Fig. S214** Overlaid chromatograms of the extracted negative ion at *m/z* 349.145 $[M-H]^-$: (a) and (b) compounds **5** and **6** in CH_3CN, respectively; (c)–(e) H_2O, MeOH, and EtOH solutions of compound **15** were sonicated for 0.5 h, respectively S225

218 **Fig. S215** Overlaid chromatograms of the extracted negative ion at *m/z* 335.130 $[M-H]^-$: (a)–(e) compounds **5a**/**6a**, **8a**, and **21**–**23** in CH_3CN, respectively; (f)–(h) H_2O, MeOH, and EtOH solutions of compound **15** were sonicated for 0.5 h, respectively S226

219 **Fig. S216** Overlaid chromatograms of the extracted negative ion at *m/z* 363.161 $[M-H]^-$: (a)–(e) compounds **7**–**11** in CH_3CN, respectively; (f)–(h) H_2O, MeOH, and EtOH solutions of compound **15** were sonicated for 0.5 h then refluxed for 1.0 h, respectively S227

220 **Fig. S217** Overlaid chromatograms of the extracted negative ion at *m/z* 305.119 $[M-H]^-$: (a) and (b) compounds **18** and **19** in CH_3CN, respectively; (c)–(e) H_2O, MeOH, and EtOH solutions of compound **15** were sonicated for 0.5 h, respectively S228

221 **Fig. S218** Overlaid chromatograms of the extracted negative ion at *m/z* 257.119 $[M-H]^-$: (a) and (b) compounds **13** and **14** in CH_3CN, respectively; (c)–(d) H_2O, MeOH, and EtOH solutions of compound **15** were sonicated for 0.5 h, respectively S229

222 **Fig. S219** Overlaid chromatograms of the extracted negative ion at *m/z* 229.087 $[M-H]^-$: (a)–(c) compounds **13a**, **14a**, and **15** in CH_3CN, respectively; (d)–(g) H_2O, MeOH, and EtOH solutions of compound **15** were sonicated for 0.5 h then refluxed for 2.0 h, respectively S230

223 **Fig. S220** Overlaid chromatograms of the extracted negative ion at *m/z* 199.077 $[M-H]^-$: (a) compound **17** in CH_3CN; (b)–(d) H_2O, MeOH, and EtOH solutions of compound **15** were sonicated for 0.5 h, respectively S231

224 **Fig. S221** Overlaid chromatograms of the extracted negative ion at *m/z* 227.072 $[M-H]^-$: (a) compound **25** in CH_3CN; (b)–(d) H_2O, MeOH, and EtOH solutions of compound **15** were sonicated for 0.5 h, respectively S232

225 **Fig. S222** Overlaid chromatograms of the extracted negative ion at *m/z* 349.145 $[M-H]^-$: (a) and (b) compounds **5** and **6** in CH_3CN, respectively; (c)–(e) H_2O, MeOH, and EtOH solutions of compound **21** were sonicated for 0.5 h, respectively. S233

226 **Fig. S223** Overlaid chromatograms of the extracted negative ion at *m/z* 335.130 $[M-H]^-$: (a)–(e) compounds **5a**/**6a**, **8a**, and **21**–**23** in CH_3CN, respectively; (f)–(h) H_2O, MeOH, and EtOH solutions of compound **21** were sonicated for 0.5 h, respectively S234

227 **Fig. S224** Overlaid chromatograms of the extracted negative ion at *m/z* 363.161 $[M-H]^-$: (a)–(e) compounds **7**–**11** in CH_3CN, respectively; (f)–(h) H_2O, MeOH, and EtOH solutions of compound **21** were sonicated for 0.5 h, respectively S235

228 **Fig. S225** Overlaid chromatograms of the extracted negative ion at *m/z* 363.161 $[M-H]^-$: (a)–(e) compounds **7**–**11** in CH_3CN, respectively; (f)–(h) H_2O, MeOH, and EtOH solutions of compound **21** were sonicated for 0.5 h then refluxed for 1.0 h, respectively. S236

229 **Fig. S226** Overlaid chromatograms of the extracted negative ion at *m/z* 305.119 $[M-H]^-$: (a) and (b) compounds **18** and **19** in CH_3CN, respectively; (c)–(e) H_2O, MeOH, and EtOH solutions of compound **21** were sonicated for 0.5 h, respectively S237

230 **Fig. S227** Overlaid chromatograms of the extracted negative ion at *m/z* 257.119 $[M-H]^-$: (a) and (b) compounds **13** and **14** in CH_3CN, respectively; (c)–(d) H_2O, MeOH, and EtOH solutions of compound **21** were sonicated for 0.5 h, respectively S238

231 **Fig. S228** Overlaid chromatograms of the extracted negative ion at *m/z* 229.087 $[M-H]^-$: (a)–(c) compounds **13a**, **14a**, and **15** in CH_3CN, respectively; (d)–(g) H_2O, MeOH, and EtOH solutions of compound **21** were sonicated for 0.5 h, respectively S239

232 **Fig. S229** Overlaid chromatograms of the extracted negative ion at *m/z* 229.087 $[M-H]^-$: (a)–(c) compounds **13a**, **14a**, and **15** in CH_3CN, respectively; (d)–(g) H_2O, MeOH, and EtOH solutions of compound **21** were sonicated for 0.5 h then refluxed for 2.0 h, respectively S240

233 **Fig. S230** Overlaid chromatograms of the extracted negative ion at *m/z* 229.087 $[M-H]^-$: (a)–(c) compounds **13a**, **14a**, and **15** in CH_3CN, respectively; (d)–(g) H_2O, MeOH, and EtOH solutions of compound **21** were sonicated for 0.5 h then refluxed for 4 h, respectively. S241

234 **Fig. S231** Overlaid chromatograms of the extracted negative ion at *m/z* 199.077 $[M-H]^-$: (a) compound **17** in CH_3CN; (b)–(d) H_2O, MeOH, and EtOH solutions of compound **21** were sonicated for 0.5 h, respectively S242

235 **Fig. S232** Overlaid chromatograms of the extracted negative ion at *m/z* 227.072 $[M-H]^-$: (a) compound **25** in CH_3CN; (b)–(d) H_2O, MeOH, and EtOH solutions of compound **21** were sonicated for 0.5 h, respectively S243

236 **Fig. S233** Overlaid chromatograms of the extracted negative ion at *m/z* 123.045 $[M-H]^-$: (a) *p*-hydroxybenzyl alcohol in CH_3CN; (b)–(d) extracts obtained by sonicating of fresh *G. elata* rhizomes with H_2O, MeOH, and EtOH, respectively; (e)–(g) extracts obtained by sonicating of "tian ma" (the steamed and dried *G. elata* rhizomes) with H_2O, S244

MeOH, and EtOH, respectively

237 **Fig. S234** Overlaid chromatograms of the extracted negative ion at *m/z* 335.130 $[M-H]^-$: (a) and (b) compounds **5a**/**6a** and **21** in CH_3CN, respectively; (c)–(e) extracts obtained by sonicating of fresh *G. elata* rhizomes with H_2O, MeOH, and EtOH, respectively; (f)–(h) extracts obtained by sonicating of "tian ma" (the steamed and dried *G. elata* rhizomes) with H_2O, MeOH, and EtOH, respectively S245

238 **Fig. S235** Overlaid chromatograms of the extracted negative ion at *m/z* 305.119 $[M-H]^-$: (a) compound **18** in CH_3CN; (b)–(d) extracts obtained by sonicating of fresh *G. elata* rhizomes with H_2O, MeOH, and EtOH, respectively; (e)–(g) extracts obtained by sonicating of "tian ma" (the steamed and dried *G. elata* rhizomes) with H_2O, MeOH, and EtOH, respectively S246

239 **Fig. S236** Overlaid chromatograms of the extracted negative ion at *m/z* 229.087 $[M-H]^-$: (a) and (b) compounds **13a** and **15** in CH_3CN, respectively; (c)–(e) extracts obtained by sonicating of fresh *G. elata* rhizomes with H_2O, MeOH, and EtOH, respectively; (f)–(h) extracts obtained by sonicating of "tian ma" (the steamed and dried *G. elata* rhizomes) with H_2O, MeOH, and EtOH, respectively S247

240 **Fig. S237** Overlaid chromatograms of the extracted negative ion at *m/z* 257.119 $[M-H]^-$: (a) compound **13** in CH_3CN; (b)–(d) extracts obtained by sonicating of fresh *G. elata* rhizomes with H_2O, MeOH, and EtOH, respectively; (e)–(g) extracts obtained by sonicating of "tian ma" (the steamed and dried *G. elata* rhizomes) with H_2O, MeOH, and EtOH, respectively S248

241 **Fig. S238** Overlaid chromatograms of the extracted negative ion at *m/z* 199.077 $[M-H]^-$: (a) compound **17** in CH_3CN; (b)–(d) extracts obtained by sonicating of fresh *G. elata* rhizomes with H_2O, MeOH, and EtOH, respectively; (e)–(g) extracts obtained by sonicating of "tian ma" (the steamed and dried *G. elata* rhizomes) with H_2O, MeOH, and EtOH, respectively S249

242 **Fig. S239** Overlaid chromatograms of the extracted negative ion at *m/z* 227.072 $[M-H]^-$: (a) compound **25** in CH_3CN; (b)–(d) extracts obtained by sonicating of fresh *G. elata* rhizomes with H_2O, MeOH, and EtOH, respectively; (e)–(g) extracts obtained by sonicating of "tian ma" (the steamed and dried *G. elata* rhizomes) with H_2O, MeOH, and EtOH, respectively S250

243 **Fig. S240** Overlaid chromatograms of the extracted negative ion at *m/z* 335.130 $[M-H]^-$: (a)–(c) compounds **5a**/**6a**, **8a**, and **21** in CH_3CN, respectively; (d)–(h) extracts obtained by sonicating of fresh *G. elata* rhizomes with H_2O for 0.5 h then refluxed for 1.0 h, 2. 0 h, 4.0 h, and 6.0 h, respectively S251

244 **Fig. S241** Overlaid chromatograms of the extracted negative ion at *m/z* 335.130 $[M-H]^-$: (a)–(c) compounds **20**, **22**, and **23** in CH_3CN, respectively; (d)–(h) extracts obtained by sonicating of fresh *G. elata* rhizomes with H_2O for 0.5 h then refluxed for 1.0 h, 2.0 h, 4.0 h, and 6.0 h, respectively S252

245 **Fig. S242** Overlaid chromatograms of the extracted negative ion at *m/z* 305.119 $[M-H]^-$: (a) and (b) compounds **18** and **19** in CH_3CN, respectively; (c)–(g) extracts obtained by sonicating of fresh *G. elata* rhizomes with H_2O for 0.5 h then refluxed for 1.0 h, 2.0 h, 4.0 h, and 6.0 h, respectively S253

246 **Fig. S243** Overlaid chromatograms of the extracted negative ion at *m/z* 229.087 $[M-H]^-$: (a)–(c) compounds **13a**, **14a**, and **15** in CH_3CN, respectively; (d)–(h) extracts obtained by sonicating of fresh *G. elata* rhizomes with H_2O for 0.5 h then refluxed for 1.0 h, 2.0 h, 4.0 h, and 6.0 h, respectively S254

247 **Fig. S244** Overlaid chromatograms of the extracted negative ion at *m/z* 259.098 $[M-H]^-$: (a) and (b) compounds **16** and **24** in CH_3CN, respectively; (c)–(g) extracts obtained by sonicating of fresh *G. elata* rhizomes with H_2O for 0.5 h then refluxed for 1.0 h, 2.0 h, 4.0 h, and 6.0 h, respectively S255

248 **Fig. S245** Overlaid chromatograms of the extracted negative ion at *m/z* 199.077 $[M-H]^-$: (a) compound **17** in CH_3CN; (b)–(f) extracts obtained by sonicating of fresh *G. elata* rhizomes with H_2O for 0.5 h then refluxed for 1.0 h, 2.0 h, 4.0 h, and 6.0 h, respectively S256

249 **Fig. S246** Overlaid chromatograms of the extracted negative ion at *m/z* 227.072 $[M-H]^-$: (a) compound **25** in CH_3CN; (b)–(f) extracts obtained by sonicating of fresh *G. elata* rhizomes with H_2O for 0.5 h then refluxed for 1.0 h, 2.0 h, 4.0 h, and 6.0 h, respectively S257

250 **Fig. S247** Overlaid chromatograms of the extracted negative ion at *m/z* 335.130 $[M-H]^-$: (a)–(c) compounds **5a**/**6a**, **8a**, and **21** in CH_3CN, respectively; (d)–(h) extracts obtained by sonicating of fresh *G. elata* rhizomes with MeOH for 0.5 h then refluxed for 1.0 h, 2.0 h, 4.0 h, and 6.0 h, respectively S258

251 **Fig. S248** Overlaid chromatograms of the extracted negative ion at *m/z* 335.130 $[M-H]^-$: (a)–(c) compounds **20**, **22**, and **23** in CH_3CN, respectively; (d)–(h) extracts obtained by sonicating of fresh *G. elata* rhizomes with MeOH for 0.5 h then refluxed for 1.0 h, 2.0 h, 4.0 h, and 6.0 h, respectively S259

252 **Fig. S249** Overlaid chromatograms of the extracted negative ion at *m/z* 305.119 $[M-H]^-$: (a) and (b) compounds **18** and **19** in CH_3CN, respectively; (c)–(g) extracts obtained by sonicating of fresh *G. elata* rhizomes with MeOH for 0.5 h then refluxed for 1.0 h, 2.0 h, 4.0 h, and 6.0 h, respectively S260

253 **Fig. S250** Overlaid chromatograms of the extracted negative ion at *m/z* 229.087 $[M-H]^-$: (a)–(c) compounds **13a**, **14a**, and **15** in CH_3CN, respectively; (d)–(h) extracts obtained by sonicating of fresh *G. elata* rhizomes with MeOH for 0.5 h then refluxed for 1.0 h, 2.0 h, 4.0 h, and 6.0 h, respectively S261

254 **Fig. S251** Overlaid chromatograms of the extracted negative ion at *m/z* 259.098 $[M-H]^-$: (a) and (b) compounds **16** and **24** in CH_3CN, respectively; (c)–(g) extracts obtained by sonicating of fresh *G. elata* rhizomes with MeOH for 0.5 h then refluxed for 1.0 h, 2.0 h, 4.0 h, and 6.0 h, respectively S262

255 **Fig. S252** Overlaid chromatograms of the extracted negative ion at *m/z* 199.077 $[M-H]^-$: (a) compound **17** in CH_3CN; (b)–(f) extracts obtained by sonicating of fresh *G. elata* rhizomes with MeOH for 0.5 h then refluxed for 1.0 h, 2.0 h, 4.0 h, and 6.0 h, respectively S263

256 **Fig. S253** Overlaid chromatograms of the extracted negative ion at *m/z* 227.072 $[M-H]^-$: (a) compound **25** in CH_3CN; (b)–(f) extracts obtained by sonicating of fresh *G. elata* rhizomes with MeOH for 0.5 h then refluxed for 1.0 h, 2.0 h, 4.0 h, and 6.0 h, respectively S264

257 **Fig. S254** Overlaid chromatograms of the extracted negative ion at *m/z* 335.130 $[M-H]^-$: (a)–(c) compounds **5a**/**6a**, **8a**, and **21** in CH_3CN, respectively; (d)–(h) extracts obtained by sonicating of fresh *G. elata* rhizomes with EtOH for 0.5 h then refluxed for 1.0 h, 2.0 h, 4.0 h, and 6.0 h, respectively S265

258 **Fig. S255** Overlaid chromatograms of the extracted negative ion at *m/z* 335.130 $[M-H]^-$: (a)–(c) compounds **20**, **22**, S266

and **23** in CH_3CN, respectively; (d)–(h) extracts obtained by sonicating of fresh *G. elata* rhizomes with EtOH for 0.5 h then refluxed for 1.0 h, 2.0 h, 4.0 h, and 6.0 h, respectively

259 **Fig. S256** Overlaid chromatograms of the extracted negative ion at *m/z* 305.119 $[M-H]^-$: (a) and (b) compounds **18** and **19** in CH_3CN, respectively; (c)–(g) extracts obtained by sonicating of fresh *G. elata* rhizomes with EtOH for 0.5 h then refluxed for 1.0 h, 2.0 h, 4.0 h, and 6.0 h, respectively S267

260 **Fig. S257** Overlaid chromatograms of the extracted negative ion at *m/z* 257.119 $[M-H]^-$: (a)–(c) compounds **12**, **13**, and **14** in CH_3CN, respectively; (d)–(h) extracts obtained by sonicating of fresh *G. elata* rhizomes with EtOH for 0.5 h then refluxed for 1.0 h, 2.0 h, 4.0 h, and 6.0 h, respectively S268

261 **Fig. S258** Overlaid chromatograms of the extracted negative ion at *m/z* 229.087 $[M-H]^-$: (a)–(c) compounds **13a**, **14a**, and **15** in CH_3CN, respectively; (d)–(h) extracts obtained by sonicating of fresh *G. elata* rhizomes with EtOH for 0.5 h then refluxed for 1.0 h, 2.0 h, 4.0 h, and 6.0 h, respectively S269

262 **Fig. S259** Overlaid chromatograms of the extracted negative ion at *m/z* 259.098 $[M-H]^-$: (a) and (b) compounds **16** and **24** in CH_3CN, respectively; (c)–(g) extracts obtained by sonicating of fresh *G. elata* rhizomes with EtOH for 0.5 h then refluxed for 1.0 h, 2.0 h, 4.0 h, and 6.0 h, respectively S270

263 **Fig. S260** Overlaid chromatograms of the extracted negative ion at *m/z* 199.077 $[M-H]^-$: (a) compound **17** in CH_3CN; (b)–(f) extracts obtained by sonicating of fresh *G. elata* rhizomes with EtOH for 0.5 h then refluxed for 1.0 h, 2.0 h, 4.0 h, and 6.0 h, respectively S271

264 **Fig. S261** Overlaid chromatograms of the extracted negative ion at *m/z* 227.072 $[M-H]^-$: (a) compound **25** in CH_3CN; (b)–(f) extracts obtained by sonicating of fresh *G. elata* rhizomes with EtOH for 0.5 h then refluxed for 1.0 h, 2.0 h, 4.0 h, and 6.0 h, respectively S272

265 **Fig. S262** Overlaid chromatograms of the extracted negative ion at *m/z* 243.066 $[M-H]^-$: (a) and (b) compounds **2** and **3** in CH_3CN, respectively; (c)–(g) extracts obtained by sonicating of "tian ma" (the steamed and dried *G. elata* rhizomes) with H_2O for 0.5 h then refluxed for 1.0 h, 2.0 h, 4.0 h, and 6.0 h, respectively S273

266 **Fig. S263** Overlaid chromatograms of the extracted negative ion at *m/z* 335.130 $[M-H]^-$: (a)–(c) compounds **5a**/**6a**, **8a**, and **21** in CH_3CN, respectively; (d)–(h) extracts obtained by sonicating of "tian ma" (the steamed and dried *G. elata* rhizomes) with H_2O for 0.5 h then refluxed for 1.0 h, 2.0 h, 4.0 h, and 6.0 h, respectively S274

267 **Fig. S264** Overlaid chromatograms of the extracted negative ion at *m/z* 335.130 $[M-H]^-$: (a)–(c) compounds **20**, **22**, and **23** in CH_3CN, respectively; (d)–(h) extracts obtained by sonicating of "tian ma" (the steamed and dried *G. elata* rhizomes) with H_2O for 0.5 h then refluxed for 1.0 h, 2.0 h, 4.0 h, and 6.0 h, respectively S275

268 **Fig. S265** Overlaid chromatograms of the extracted negative ion at *m/z* 305.119 $[M-H]^-$: (a) and (b) compounds **18** and **19** in CH_3CN, respectively; (c)–(g) extracts obtained by sonicating of "tian ma" (the steamed and dried *G. elata* rhizomes) with H_2O for 0. 5 h then refluxed for 1.0 h, 2.0 h, 4.0 h, and 6.0 h, respectively S276

269 **Fig. S266** Overlaid chromatograms of the extracted negative ion at *m/z* 229.087 $[M-H]^-$: (a)–(c) compounds **13a**, **14a**, and **15** in CH_3CN, respectively; (d)–(h) extracts obtained by sonicating of "tian ma" (the steamed and dried *G. elata* rhizomes) with H_2O for 0.5 h then refluxed for 1.0 h, 2.0 h, 4.0 h, and 6.0 h, respectively S277

270 **Fig. S267** Overlaid chromatograms of the extracted negative ion at *m/z* 259.098 $[M-H]^-$: (a) and (b) compounds **16** and **24** in CH_3CN, respectively; (c)–(g) extracts obtained by sonicating of "tian ma" (the steamed and dried *G. elata* rhizomes) with H_2O for 0.5 h then refluxed for 1.0 h, 2.0 h, 4.0 h, and 6.0 h, respectively S278

271 **Fig. S268** Overlaid chromatograms of the extracted negative ion at *m/z* 199.077 $[M-H]^-$: (a) compound **17** in CH_3CN; (b)–(f) extracts obtained by sonicating of "tian ma" (the steamed and dried *G. elata* rhizomes) with H_2O for 0. 5 h then refluxed for 1.0 h, 2.0 h, 4.0 h, and 6.0 h, respectively S279

272 **Fig. S269** Overlaid chromatograms of the extracted negative ion at *m/z* 227.072 $[M-H]^-$: (a) compound **25** in CH_3CN; (b)–(f) extracts obtained by sonicating of "tian ma" (the steamed and dried *G. elata* rhizomes) with H_2O for 0.5 h then refluxed for 1.0 h, 2.0 h, 4.0 h, and 6.0 h, respectively S280

273 **Fig. S270** Overlaid chromatograms of the extracted negative ion at *m/z* 243.066 $[M-H]^-$: (a) and (b) compounds **2** and **3** in CH_3CN, respectively; (c)–(g) extracts obtained by sonicating of "tian ma" (the steamed and dried *G. elata* rhizomes) with MeOH for 0.5 h then refluxed for 1.0 h, 2.0 h, 4.0 h, and 6.0 h, respectively S281

274 **Fig. S271** Overlaid chromatograms of the extracted negative ion at *m/z* 349.145 $[M-H]^-$: (a) and (b) compounds **5** and **6** in CH_3CN, respectively; (c)–(g) extracts obtained by sonicating of "tian ma" (the steamed and dried *G. elata* rhizomes) with MeOH for 0.5 h then refluxed for 1.0 h, 2.0 h, 4.0 h, and 6.0 h, respectively S282

275 **Fig. S272** Overlaid chromatograms of the extracted negative ion at *m/z* 335.130 $[M-H]^-$: (a)–(c) compounds **5a**/**6a**, **8a**, and **21** in CH_3CN, respectively; (d)–(h) extracts obtained by sonicating of "tian ma" (the steamed and dried *G. elata* rhizomes) with MeOH for 0.5 h then refluxed for 1.0 h, 2.0 h, 4.0 h, and 6.0 h, respectively S283

276 **Fig. S273** Overlaid chromatograms of the extracted negative ion at *m/z* 335.130 $[M-H]^-$: (a)–(c) compounds **20**, **22**, and **23** in CH_3CN, respectively; (d)–(h) extracts obtained by sonicating of "tian ma" (the steamed and dried *G. elata* rhizomes) with MeOH for 0.5 h then refluxed for 1.0 h, 2.0 h, 4.0 h, and 6.0 h, respectively S284

277 **Fig. S274** Overlaid chromatograms of the extracted negative ion at *m/z* 305.119 $[M-H]^-$: (a) and (b) compounds **18** and **19** in CH_3CN, respectively; (c)–(g) extracts obtained by sonicating of "tian ma" (the steamed and dried *G. elata* rhizomes) with MeOH for 0.5 h then refluxed for 1.0 h, 2.0 h, 4.0 h, and 6.0 h, respectively S285

278 **Fig. S275** Overlaid chromatograms of the extracted negative ion at *m/z* 229.087 $[M-H]^-$: (a)–(c) compounds **13a**, **14a**, and **15** in CH_3CN, respectively; (d)–(h) extracts obtained by sonicating of "tian ma" (the steamed and dried *G. elata* rhizomes) with MeOH for 0.5 h then refluxed for 1.0 h, 2.0 h, 4.0 h, and 6.0 h, respectively S286

279 **Fig. S276** Overlaid chromatograms of the extracted negative ion at *m/z* 259.098 $[M-H]^-$: (a) and (b) compounds **16** and **24** in CH_3CN, respectively; (c)–(g) extracts obtained by sonicating of "tian ma" (the steamed and dried *G. elata* rhizomes) with MeOH for 0.5 h then refluxed for 1.0 h, 2.0 h, 4.0 h, and 6.0 h, respectively S287

280 **Fig. S277** Overlaid chromatograms of the extracted negative ion at *m/z* 199.077 $[M-H]^-$: (a) compound **17** in CH_3CN; (b)–(f) extracts obtained by sonicating of "tian ma" (the steamed and dried *G. elata* rhizomes) with MeOH for 0.5 h then refluxed for 1.0 h, 2.0 h, 4.0 h, and 6.0 h, respectively S288

281 **Fig. S278** Overlaid chromatograms of the extracted negative ion at *m/z* 227.072 $[M-H]^-$: (a) compound **25** in CH_3CN; (b)–(f) extracts obtained by sonicating of "tian ma" (the steamed and dried *G. elata* rhizomes) with MeOH for 0.5 h then refluxed for 1.0 h, 2.0 h, 4.0 h, and 6.0 h, respectively S289

282	**Fig. S279** Overlaid chromatograms of the extracted negative ion at *m/z* 287.129 $[M-H]^-$: (a) compound **1** in CH_3CN; (b)–(f) extracts obtained by sonicating of "tian ma" (the steamed and dried *G. elata* rhizomes) with EtOH for 0.5 h then refluxed for 1.0 h, 2.0 h, 4.0 h, and 6.0 h, respectively	S290
283	**Fig. S280** Overlaid chromatograms of the extracted negative ion at *m/z* 243.066 $[M-H]^-$: (a) and (b) compounds **2** and **3** in CH_3CN, respectively; (c)–(g) extracts obtained by sonicating of "tian ma" (the steamed and dried *G. elata* rhizomes) with EtOH for 0.5 h then refluxed for 1.0 h, 2.0 h, 4.0 h, and 6.0 h, respectively	S291
284	**Fig. S281** Overlaid chromatograms of the extracted negative ion at *m/z* 335.130 $[M-H]^-$: (a)–(c) compounds **5a**/**6a**, **8a**, and **21** in CH_3CN, respectively; (d)–(h) extracts obtained by sonicating of "tian ma" (the steamed and dried *G. elata* rhizomes) with EtOH for 0.5 h then refluxed for 1.0 h, 2.0 h, 4.0 h, and 6.0 h, respectively	S292
285	**Fig. S282** Overlaid chromatograms of the extracted negative ion at *m/z* 335.130 $[M-H]^-$: (a)–(c) compounds **20**, **22**, and **23** in CH_3CN, respectively; (d)–(h) extracts obtained by sonicating of "tian ma" (the steamed and dried *G. elata* rhizomes) with EtOH for 0.5 h then refluxed for 1.0 h, 2.0 h, 4.0 h, and 6.0 h, respectively	S293
286	**Fig. S283** Overlaid chromatograms of the extracted negative ion at *m/z* 305.119 $[M-H]^-$: (a) and (b) compounds **18** and **19** in CH_3CN, respectively; (c)–(g) extracts obtained by sonicating of "tian ma" (the steamed and dried *G. elata* rhizomes) with EtOH for 0.5 h then refluxed for 1.0 h, 2.0 h, 4.0 h, and 6.0 h, respectively	S294
287	**Fig. S284** Overlaid chromatograms of the extracted negative ion at *m/z* 363.161 $[M-H]^-$: (a) and (b) compounds **7** and **9** in CH_3CN, respectively; (c)–(g) extracts obtained by sonicating of "tian ma" (the steamed and dried *G. elata* rhizomes) with EtOH for 0.5 h then refluxed for 1.0 h, 2.0 h, 4.0 h, and 6.0 h, respectively	S295
288	**Fig. S285** Overlaid chromatograms of the extracted negative ion at *m/z* 257.119 $[M-H]^-$: (a) – (c) compounds **12**, **13**, and **14** in CH_3CN, respectively; (d)–(h) extracts obtained by sonicating of "tian ma" (the steamed and dried *G. elata* rhizomes) with EtOH for 0.5 h then refluxed for 1.0 h, 2.0 h, 4.0 h, and 6.0 h, respectively	S296
289	**Fig. S286** Overlaid chromatograms of the extracted negative ion at *m/z* 229.087 $[M-H]^-$: (a)–(c) compounds **13a**, **14a**, and **15** in CH_3CN, respectively; (d)–(h) extracts obtained by sonicating of "tian ma" (the steamed and dried *G. elata* rhizomes) with EtOH for 0.5 h then refluxed for 1.0 h, 2.0 h, 4.0 h, and 6.0 h, respectively	S297
290	**Fig. S287** Overlaid chromatograms of the extracted negative ion at *m/z* 259.098 $[M-H]^-$: (a) and (b) compounds **16** and **24** in CH_3CN, respectively; (c)–(g) extracts obtained by sonicating of "tian ma" (the steamed and dried *G. elata* rhizomes) with EtOH for 0.5 h then refluxed for 1.0 h, 2.0 h, 4.0 h, and 6.0 h, respectively	S298
291	**Fig. S288** Overlaid chromatograms of the extracted negative ion at *m/z* 199.077 $[M-H]^-$: (a) compound **17** in CH_3CN; (b)–(f) extracts obtained by sonicating of "tian ma" (the steamed and dried *G. elata* rhizomes) with EtOH for 0.5 h then refluxed for 1.0 h, 2.0 h, 4.0 h, and 6.0 h, respectively	S299
292	**Fig. S289** Overlaid chromatograms of the extracted negative ion at *m/z* 227.072 $[M-H]^-$: (a) compound **25** in CH_3CN; (b)–(f) extracts obtained by sonicating of "tian ma" (the steamed and dried *G. elata* rhizomes) with EtOH for 0.5 h then refluxed for 1.0 h, 2.0 h, 4.0 h, and 6.0 h, respectively	S300

General experimental procedures

UV spectra were measured on a Cary 300 spectrometer (Agilent Technologies, California, USA). CD spectra were measured on a JASCO J-810 spectropolarimeter (JASCO, Tokyo, Japan). IR spectra were recorded on a Nicolet 5700 FT-IR microscope spectrometer (FT-IR microscope transmission) (Thermo Electron Corporation, Madison, WI, USA) by microscope transmission method. 1D- and 2D-NMR spectra were obtained at 500 MHz or 600 MHz for ^{1}H and 125 MHz or 150 MHz for ^{13}C, respectively, on INOVA 500 MHz or SYS 600 MHz spectrometers (Varian Associates Inc., Palo Alto, CA, USA) (Bruker BioSpin AG, Fällanden, Switzerland), with solvent peaks serving as references (unless otherwise noted). ESI-MS data were measured with a Q-Trap LC/MS/MS (Turbo Ionspray source) spectrometer. HR-ESI-MS data were measured using a Q Exactive Focus LC-MS/MS spectrometer (Thermo Fisher Scientific, WI, USA), or a Micromass Autospec−Ultima ETOF spectrometer (Waters Corporation, Milford, USA). Column chromatography (CC) was performed with silica gel (200–300 mesh, Qingdao Marine Chemical Inc. Qingdao, China) and Sephadex LH-20 (Pharmacia Biotech AB, Uppsala, Sweden), Toyopearl HW-40C and HW-40F (Tosoh Corporation, Tokyo, Japan), and MCI gel (CHP20P, 75–150 μm) (Mitsubishi Chemical Corporation, Tokyo, Japan). HPLC separation was performed on an instrument with a LabAlliance prep pump (Teledyne SSI, PA, USA) and a UV6000LP PDA Detector (Thermo Fisher Scientific, MA, USA) on a Grace (250 × 10 mm ID) semi-preparative column packed with C_{18} (5 μm) (Grace Inc., Columbia, USA), an YMC-Pack (250 × 10 mm ID) column packed with Ph (5 μm) (YMC Corporation, Kyoto, Japan), an Ulitimate (250 × 10 mm) column packed with C_8 (5μm) (Welch Materials Inc., Shanghai, China), and a PBT column packed with poly(butylene terephthalate) (5 μm) [Daicel Chiral Technologies (China) CO., LTD, Shanghai, China]. TLC was carried out with glass precoated silica gel GF_{254} plates (Qingdao Marine Chemical Inc.). Spots were visualized under UV light or by spraying with 5% H_2SO_4 in EtOH followed by heating.

Plant material

The steamed and dried rhizomes of *Gastrodia elata* were purchased at the plantation field Xiao Cao Ba, Yunnan province, China, in December 2009, and the fresh *G. elata* rhizomes were collected at the same field in December 2019.

Plant identification was verified by Mr. Lin Ma (Institute of Materia Medica, Beijing 100050, China). A voucher specimen (no. ID-S-2384) was deposited at the herbarium of the Department of Chemistry of Natural Products, Institute of Materia Medica.

Preliminary extraction and isolation

The steamed and air-dried *G. elata* rhizomes (50 kg) were pulverized and ultrasonicated with H_2O (150 L, 3 × 1 h). The aqueous extracts were combined and evaporated under reduced pressure to yield a concentrated solution (50 L), which was loaded on a macroporous adsorbent resin (HPD-100, 30 kg) column (20 × 200 cm), and eluted successively with H_2O (50 L), 30% EtOH (150 L), 50% EtOH (120 L), and 95% EtOH (80 L) to yield four corresponding fractions A−D. After removing the solvent under reduced pressure, fraction C (1.9 kg) was chromatographed over MCI gel (CHP 20P, 75–150 μm, 10 L), with successive elution using H_2O (30 L), 30% EtOH (70 L), 50% EtOH (70 L), 95% EtOH (30 L), and Me_2CO (20 L), to afford fractions C1−C5.

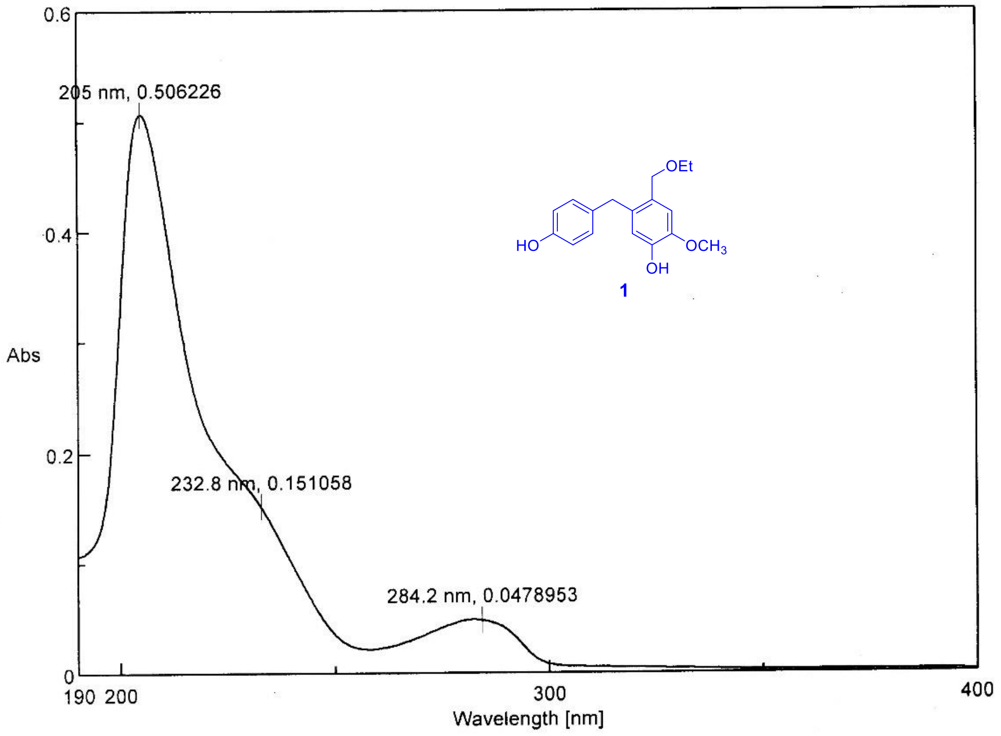

zx-65

[Comment]	
Sample Name	zx-65
Comment	0.02
User	
Division	UV
Company	324
[Measurement Information]	
Instrument Name	V-650
Model Name	V-650
Serial No.	A034461150
Accessory	PSC-718
Accessory S/N	A001761114
Position	2
Cell Length	10 mm
Temperature	19.97 C
Control Sonsor	Holder
Monitor Sensor	Holder
Start Mode	Start immediately
Photometric Mode	Abs
Measurement range	400 - 190 nm
Data pitch	0.2 nm
Band width(UV/Vis)	2.0 nm
Response	Medium
Scanning speed	200 nm/min
Source Change	340 nm
Light Source	D2/WI
Filter Exchange	Step
Correction	Baseline

[Data Information]	
Creation Date	2016-7-6 15:39
Data array type	Linear data array
Horizontal	Wavelength [nm]
Vertical	Abs
Start	400 nm
End	190 nm
Data pitch	0.2 nm
Data points	1051

Fig. S1 The UV spectrum of compound **1**.

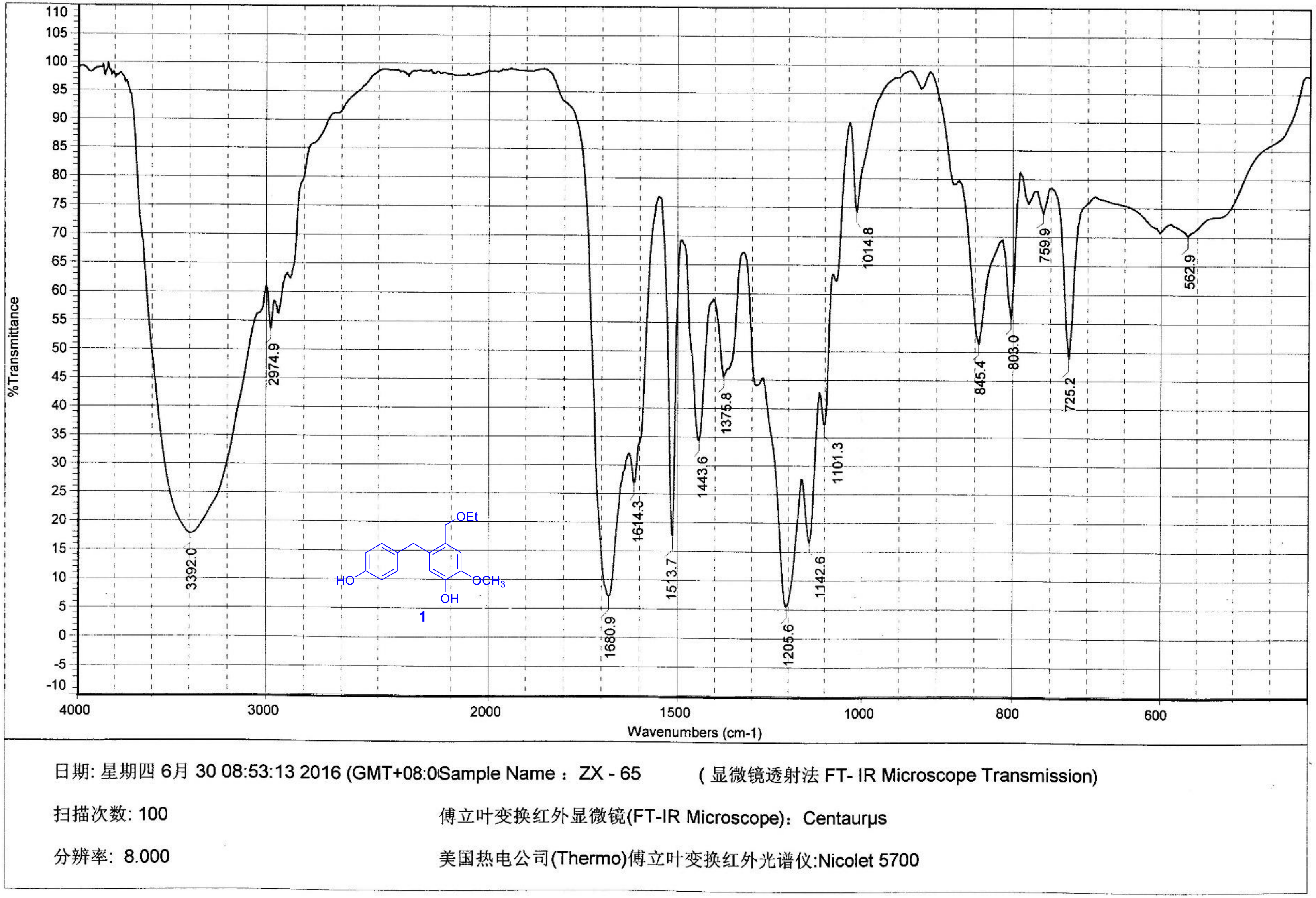

Fig. S2 The IR spectrum of compound **1**.

Display Report - Selected Window Selected

Analysis Name: zhoux016.d
Method: TEST.MS
Sample Name: zx-65
Analysis Info:

Instrument: LC-MSD-Trap-SL
Operator: Operator

Print Date: 12/23/20159:09:21 AM
Acq. Date: 12/23/2015 9:07:46 AM

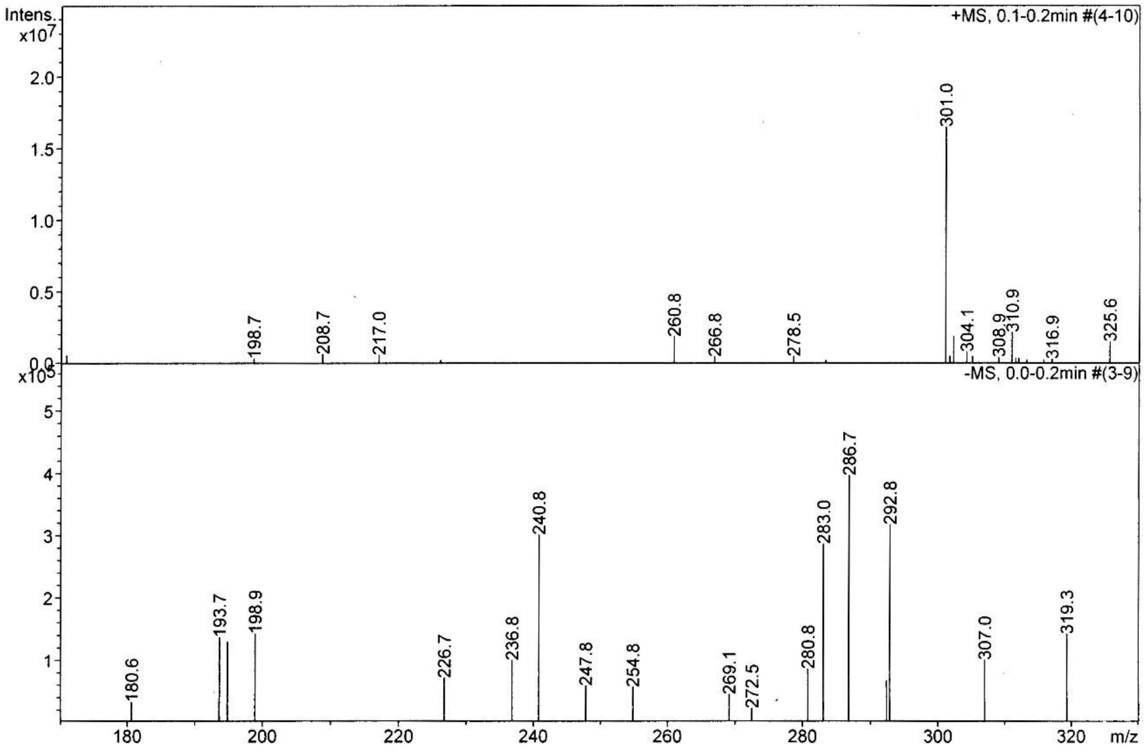

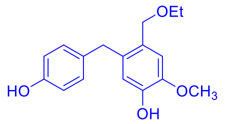

1

Agilent Technologies

MSD Trap Report v 4 (A

Page 1 of 1

Fig. S3 The ESI-MS of compound **1**.

Qualitative Analysis Report

**Data Filename**	2016060101.d	**Sample Name**	ZX-65
**Sample Type**	Sample	**Position**	P1-C1
**Instrument Name**	Instrument 1	**User Name**	
**Acq Method**		**IRM Calibration Status**	Success
**DA Method**	TEST LCMS.m	**Comment**	

User Chromatograms

Fragmentor Voltage 135 **Collision Energy** 0 **Ionization Mode** ESI

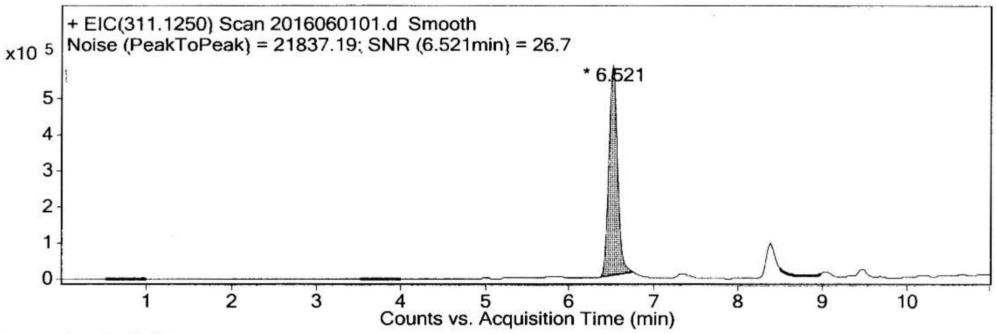

Integration Peak List

Peak	Start	RT	End	Height	Area	Area %	Signal To Noise
1	6.376	6.521	6.763	582236	4238297	100	26.7

User Spectra

**Fragmentor Voltage**	**Collision Energy**	**Ionization Mode**
135	0	Esi

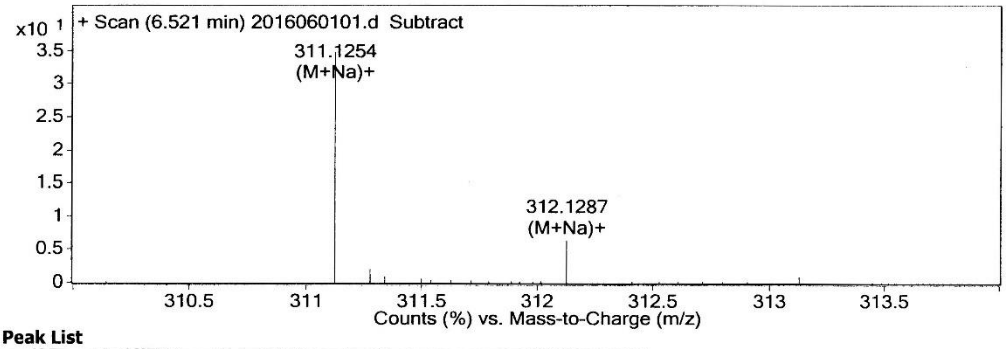

Peak List

m/z	z	Abund	Formula	Ion
243.1039	1	2275851		
243.2352		237198		
244.1044	1	521571		
311.1254	1	790003	C17 H20 Na O4	(M+Na)+
312.1287	1	143553	C17 H20 Na O4	(M+Na)+
327.0989		120287		

Formula Calculator Element Limits

Element	Min	Max
C	3	60
H	0	120
O	0	30
N	0	3
S	0	3
Cl	0	0
P	0	0

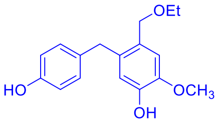

1

Fig. S4 The (+)-HR-ESI-MS report of compound **1**, page 1.

Qualitative Analysis Report

Element	Min	Max
Br	0	0
F	0	0
Si	0	0

Formula Calculator Results

Formula	Best	Mass	Tgt Mass	Diff (ppm)	Ion Species	Score
C17 H20 O4	TRUE	288.1362	288.1362	-0.03	C17 H20 Na O4	99.98

--- End Of Report ---

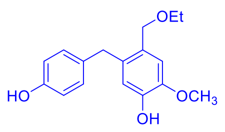

1

Fig. S5 The (+)-HR-ESI-MS report of compound **1**, page 2.

MS Formula Results: + Scan (6.521 min) Sub (2016060101.d)

	m/z	Ion	Formula	Abundance
-	311.1254	(M+Na)+	C17 H20 Na O4	790002.9

	Best	Formula (M)	Ion Formula	Score	Cross Sco	Mass	Calc Mass	Calc m/z	Diff (ppm)	Abs Diff (ppm)	Mass Match	Abund Match	Spacing Match	DBE
+	✓	C17 H20 O4	C17 H20 Na O4	99.98		288.1362	288.1362	311.1254	-0.03	0.03	100	99.92	100	8

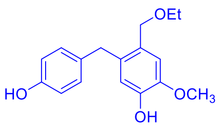

1

page 1

Fig. S6 The (+)-HR-ESI-MS report of compound **1**, page 3.

PROTON_02
VNS-600 PROTON zx-65 IN acetone Sep 25 2015

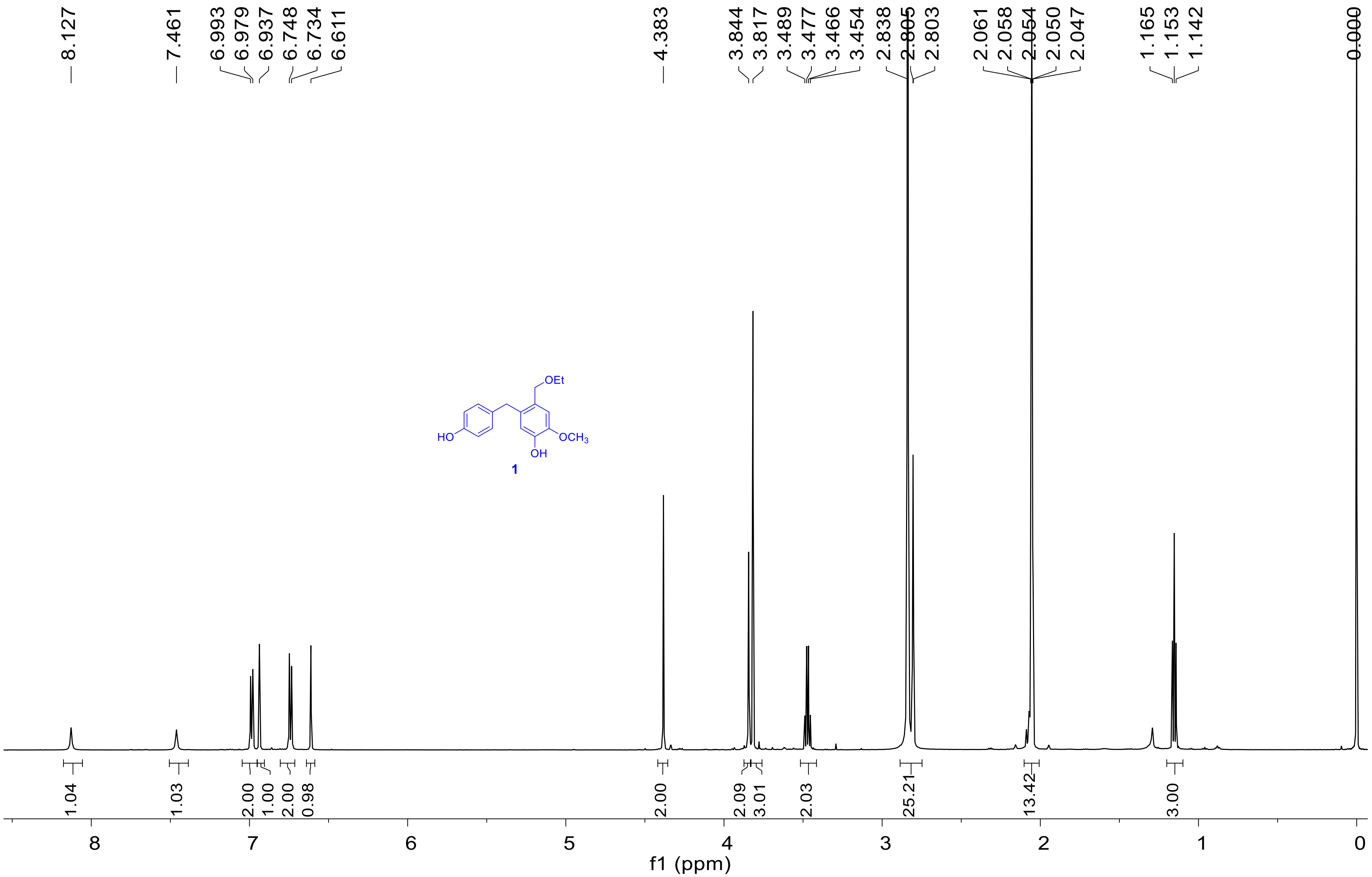

Fig. S7 The ^{1}H NMR spectrum of compound **1** in acetone-d_6 (600 MHz).

CARBON_01
VNS-600 CARBON zx-65 IN acetone Oct 10 2015

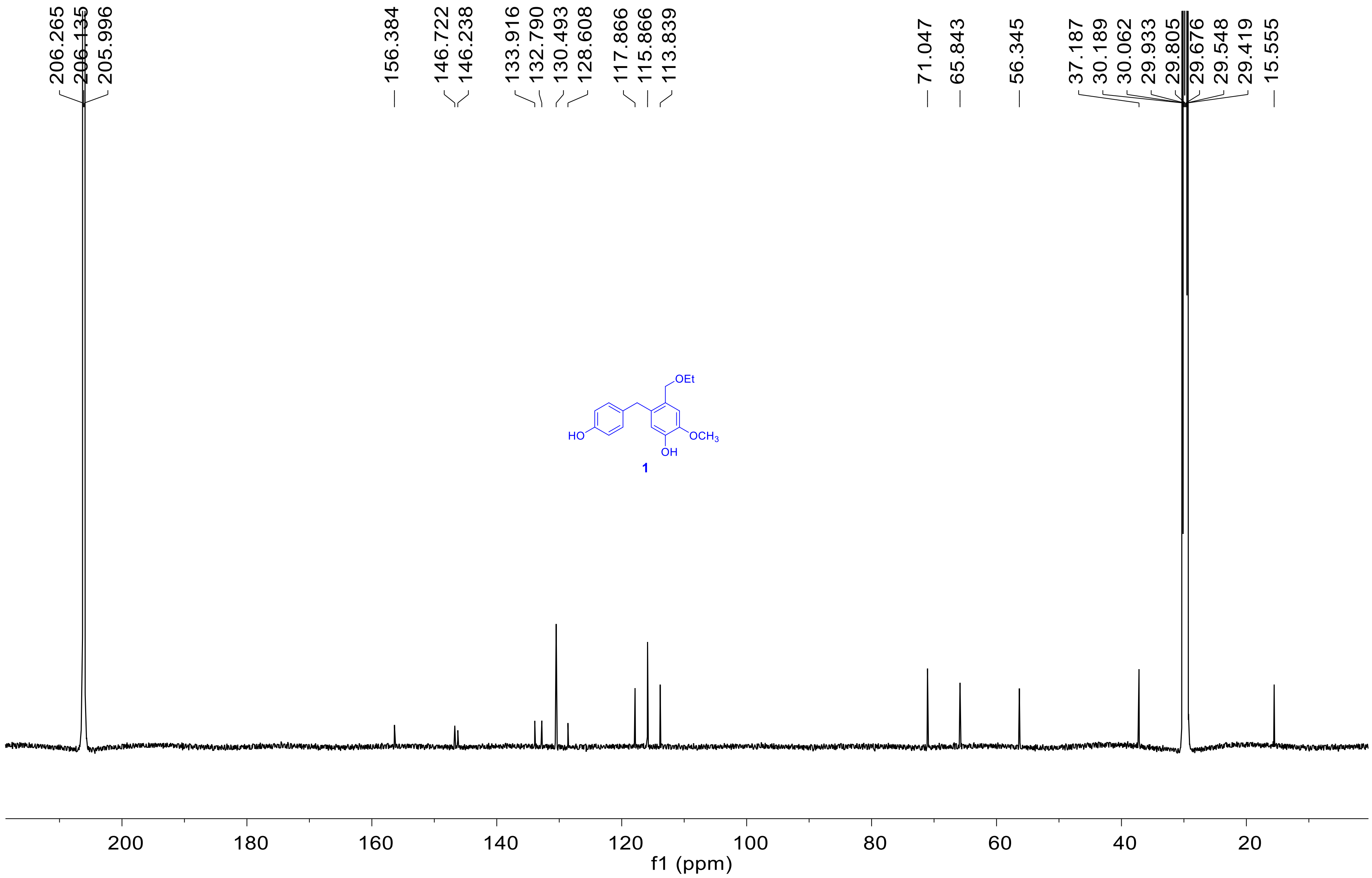

Fig. S8 The ^{13}C NMR spectrum of compound **1** in acetone-d_6 (150 MHz).

DEPT_01
VNS-600 DEPT zx-65 IN acetone Oct 10 2015

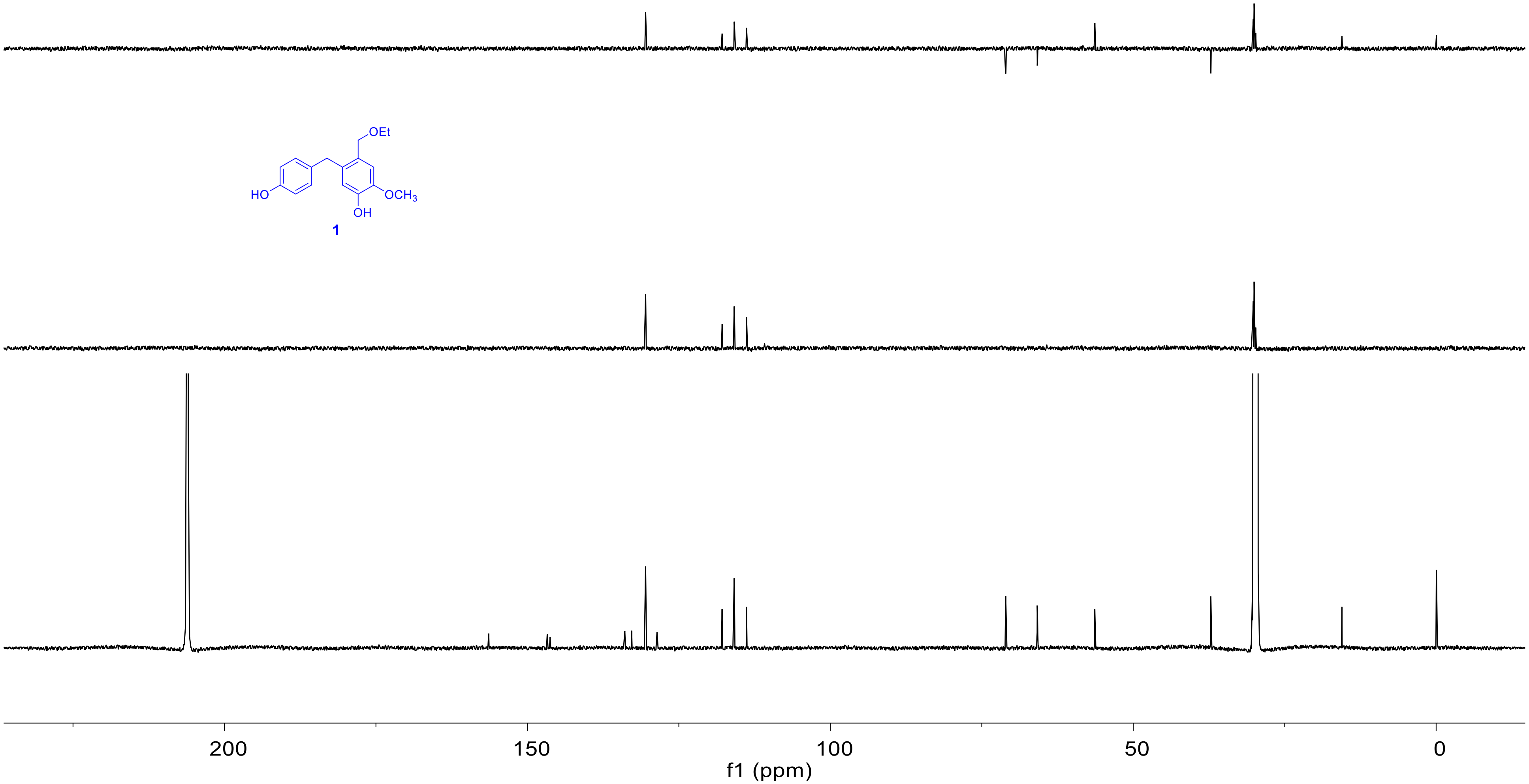

1

Fig. S9 The DEPT spectrum of compound **1** in acetone-d_6 (150 MHz).

gCOSY_01
VNS-600 gCOSY zx-65 IN acetone Jun 21 2016

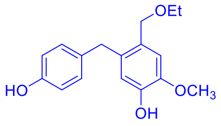
1

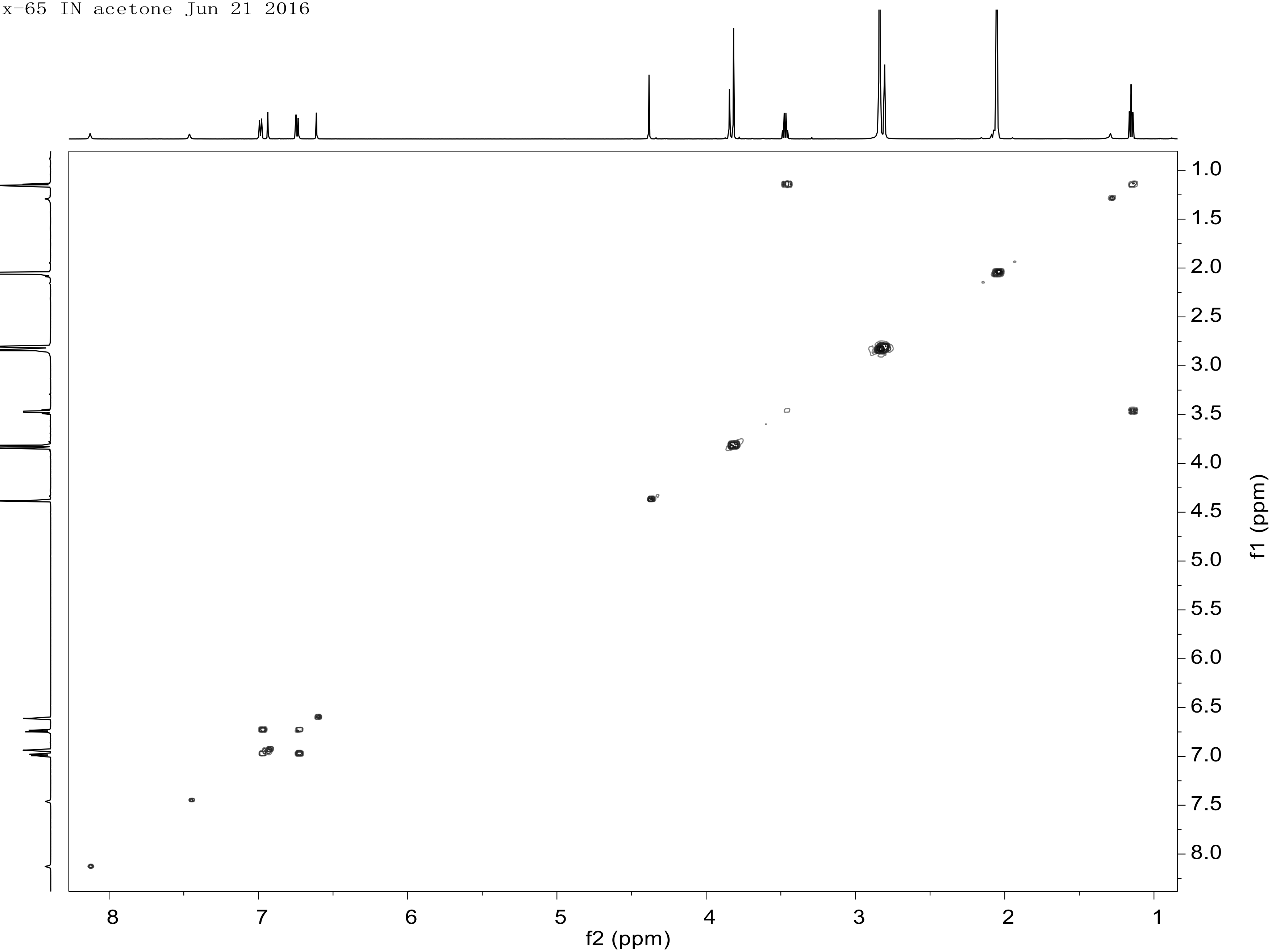

Fig. S10 The ^{1}H-^{1}H COSY spectrum of compound **1** in acetone-d_6 (600 MHz)

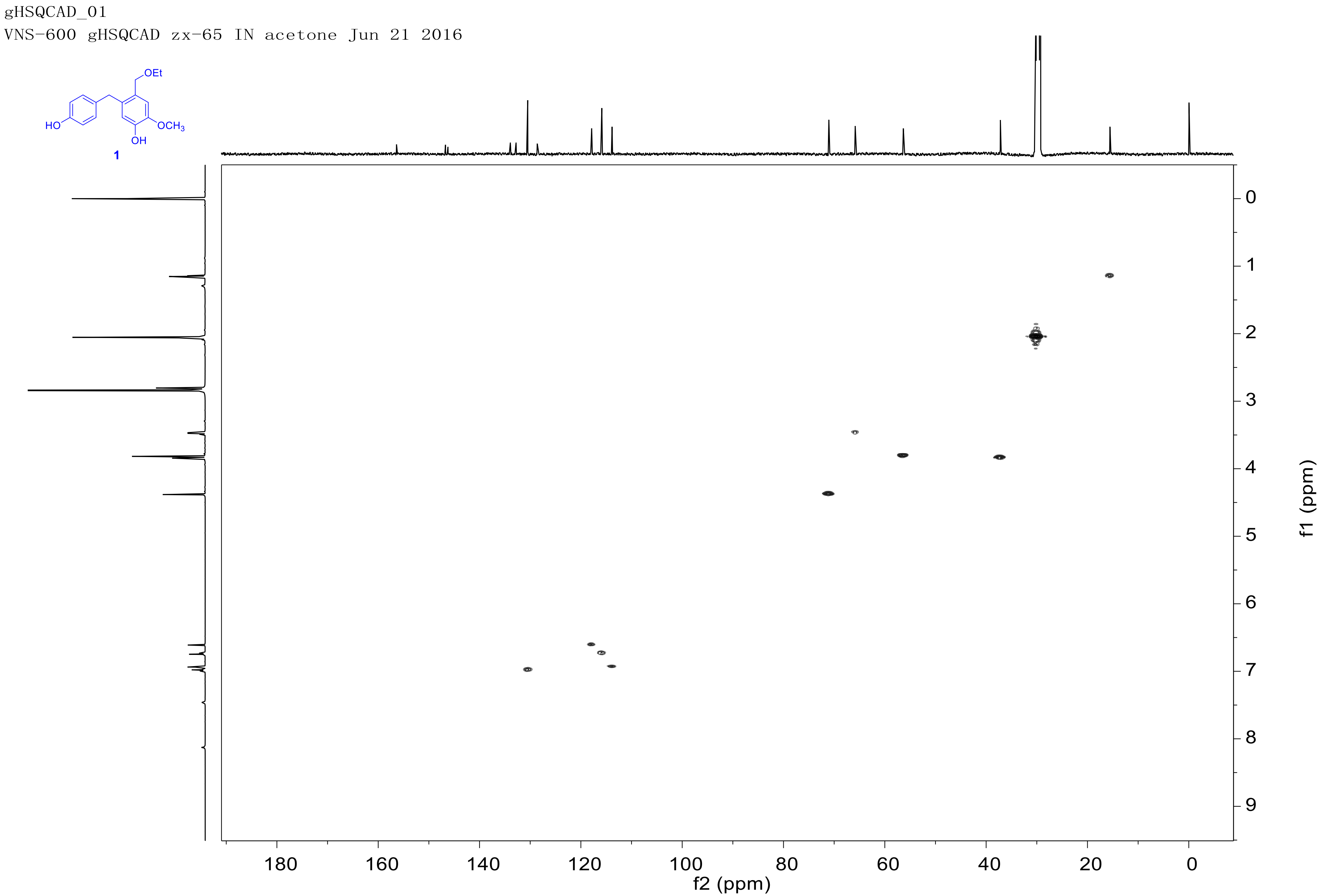

Fig. S11 The HSQC spectrum of compound **1** in acetone-d_6 (600 MHz for ^{1}H).

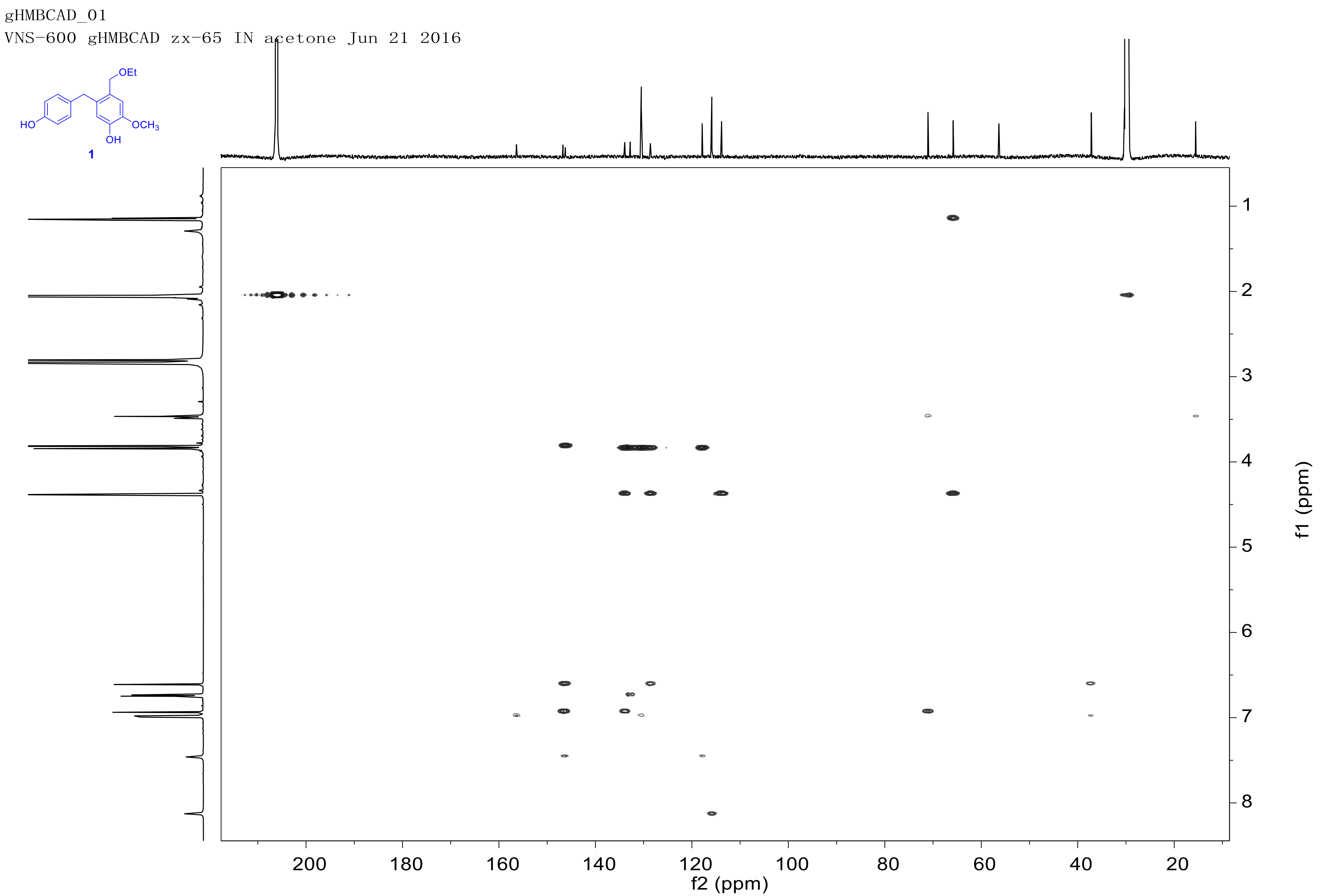

Fig. S12 The HMBC spectrum of compound **1** in acetone-d_6 (600 MHz for ^{1}H).

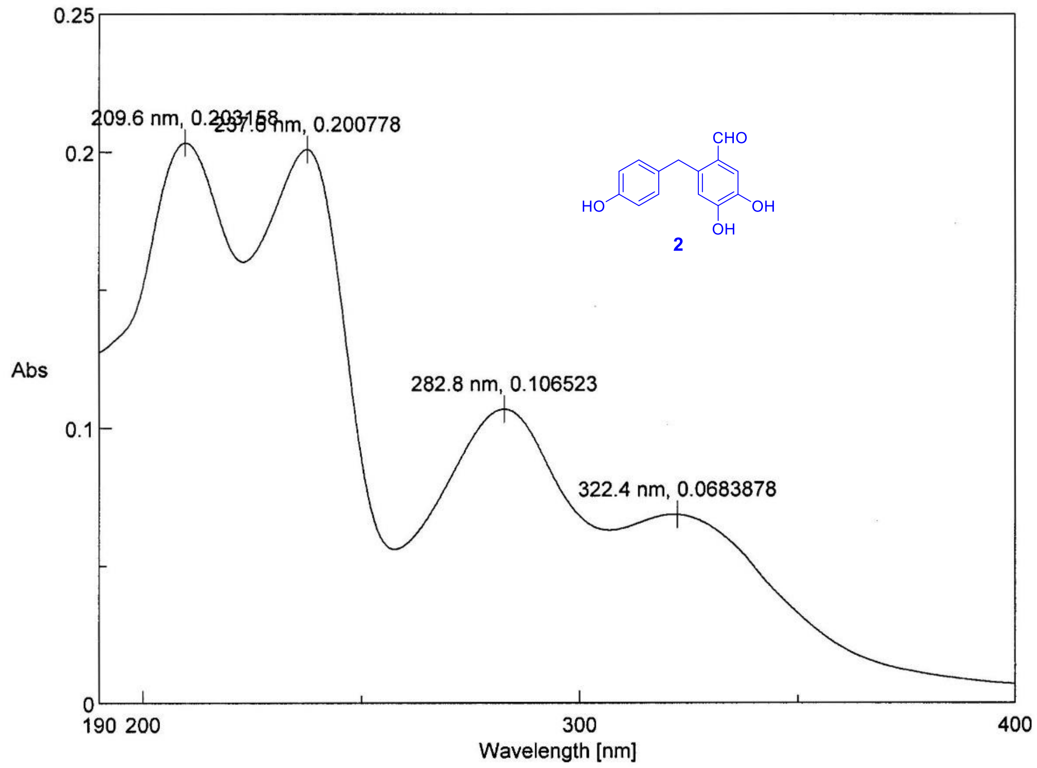

— TMG-55B

[Comment]

Sample Name	TMG-55B
Comment	0.02
User	
Division	UV
Company	324

[Measurement Information]

Instrument Name	V-650
Model Name	V-650
Serial No.	A034461150
Accessory	PSC-718
Accessory S/N	A001761114
Position	1
Cell Length	10 mm
Temperature	19.99 C
Control Sonsor	Holder
Monitor Sensor	Holder
Start Mode	Start immediately
Photometric Mode	Abs
Measurement range	400 - 190 nm
Data pitch	0.2 nm
Band width(UV/Vis)	2.0 nm
Response	Medium
Scanning speed	200 nm/min
Source Change	340 nm
Light Source	D2/WI
Filter Exchange	Step
Correction	Baseline

[Data Information]

Creation Date	2014-2-26 10:20
Data array type	Linear data array
Horizontal	Wavelength [nm]
Vertical	Abs
Start	400 nm
End	190 nm
Data pitch	0.2 nm
Data points	1051

Fig. S13 The UV spectrum of compound **2**.

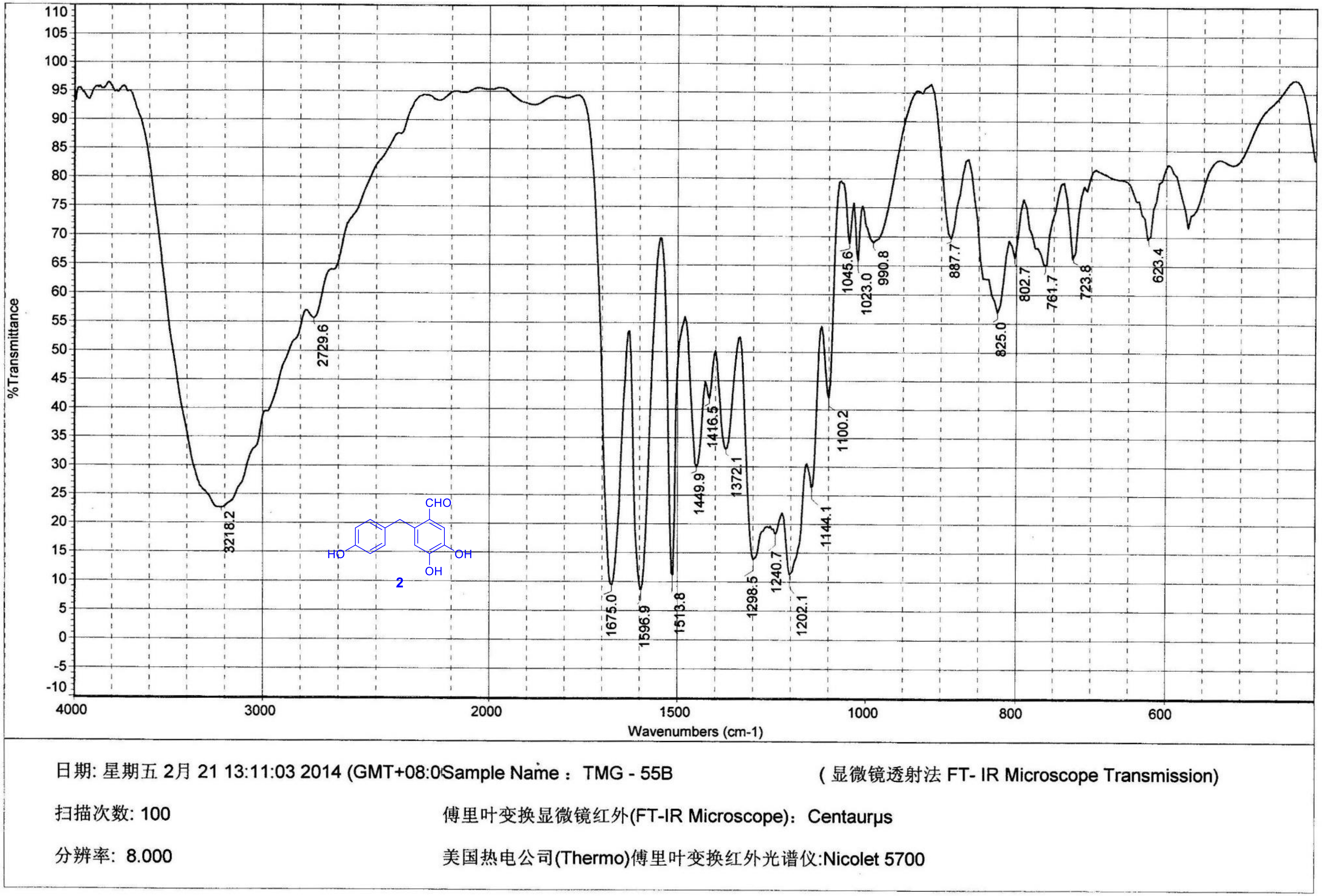

Fig. S14 The IR spectrum of compound **2**.

Single Mass Spectrum Deconvolution Report

**Analysis Name:**	guoql105.d	**Instrument:**	LC-MSD-Trap-SL	**Print Date:**	12/12/2012 11:35:02 AM
**Method:**	TEST.MS	**Operator:**	Operator	**Acq. Date:**	12/12/2012 10:58:23 AM
**Sample Name:**	TMG-55B				
**Analysis Info:**					

Acquisition Parameter:

Mass Range Mode	Std/Normal	Trap Drive	53.0	Scan Begin	100 m/z
Ion Polarity	Positive	Octopole RF Amplitude	171.0 Vpp	Scan End	800 m/z
Ion Source Type	ESI	Capillary Exit	-106.0 Volt	Averages	5 Spectra
Dry Temp (Set)	330 °C	Skimmer	-40.0 Volt	Max. Accu Time	200000 µs
Nebulizer (Set)	15.00 psi	Oct 1 DC	-12.00 Volt	ICC Target	20000
Dry Gas (Set)	6.00 l/min	Oct 2 DC	-1.70 Volt	Charge Control	on

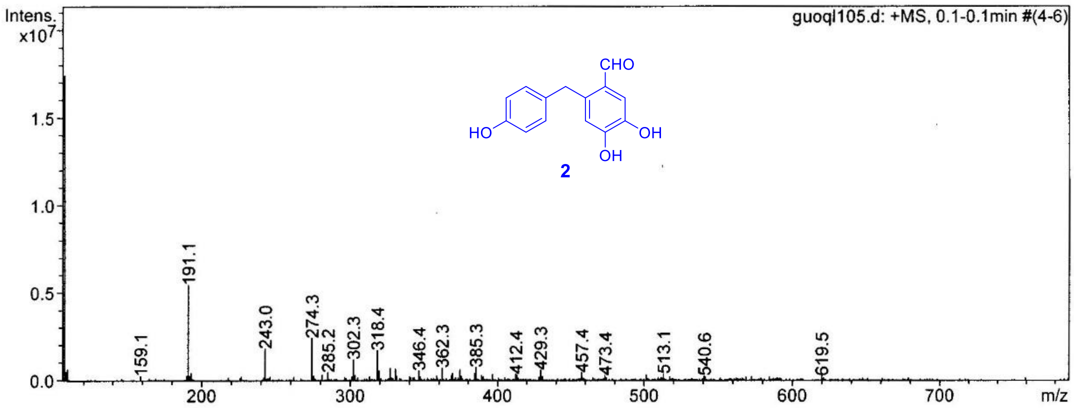

Component	Molecular Mass	Molecule	Absolute Abundance	Relative Abundance

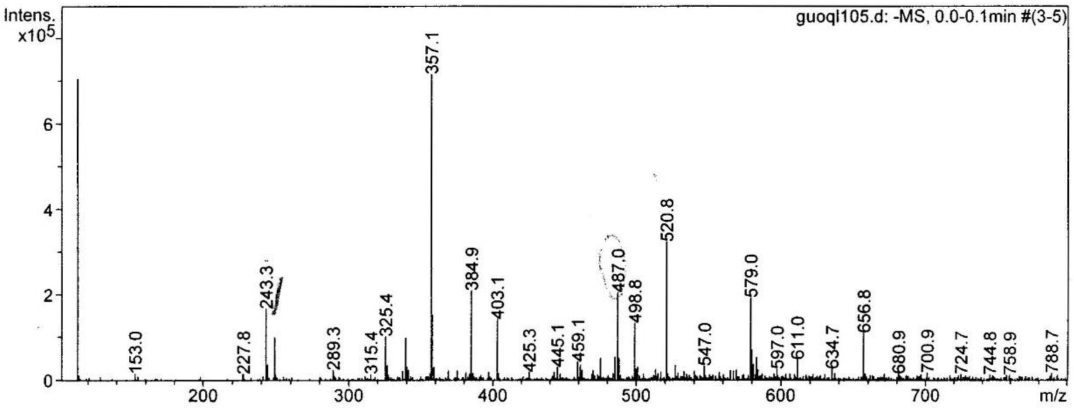

Component	Molecular Mass	Molecule	Absolute Abundance	Relative Abundance

Fig. S15 The ESI-MS of compound **2**.

Qualitative Analysis Report

**Data Filename**	2012112307.d	**Sample Name**	TMG-55B
**Sample Type**	Sample	**Position**	P1-D9
**Instrument Name**	Instrument 1	**User Name**	
**Acq Method**		**IRM Calibration Status**	Success
**DA Method**	TEST LCMS.m	**Comment**	

User Chromatograms

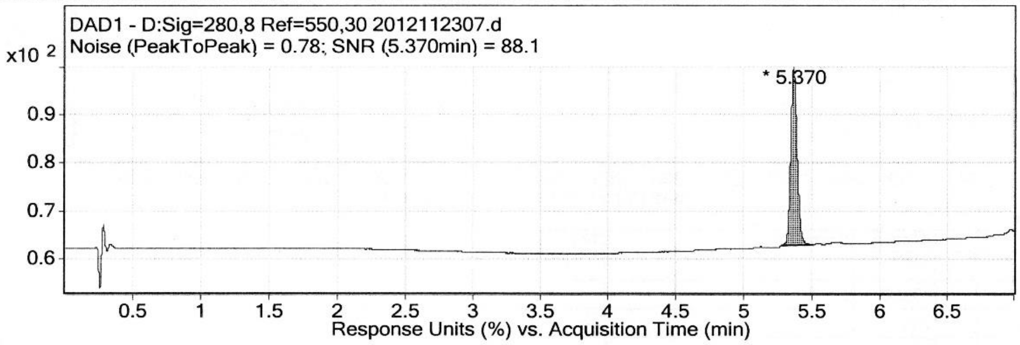

Integration Peak List

Peak	Start	RT	End	Height	Area	Area %	Signal To Noise
1	5.281	5.37	5.512	21.36	68.61	100	88.1

Noise Measurements

Noise Type	Signal Definition	Noise Multiplier	Noise Value
Peak-to-Peak	Area	1	0.779151917

Noise Regions

Start	End
0.5	0.8
4.5	5
8.6	9.4
9.8	11

Fragmentor Voltage 135 **Collision Energy** 0 **Ionization Mode** ESI

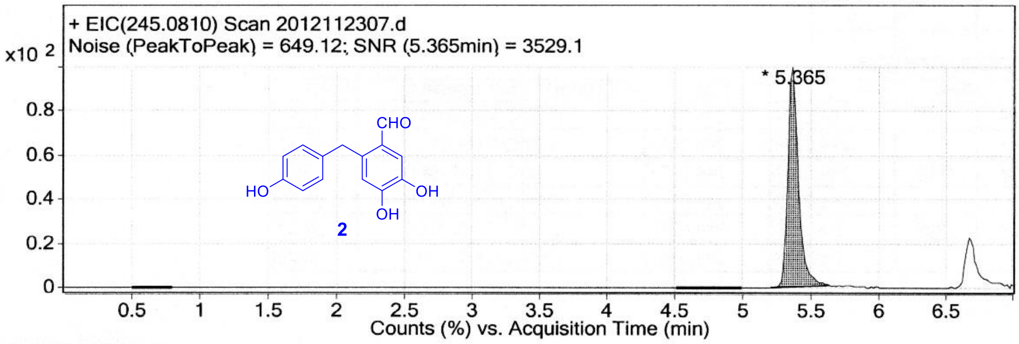

Integration Peak List

Peak	Start	RT	End	Height	Area	Area %	Signal To Noise
1	5.204	5.365	5.638	419217	2290795	100	3529.1

Noise Measurements

Noise Type	Signal Definition	Noise Multiplier	Noise Value
Peak-to-Peak	Area	1	649.1154785

Noise Regions

Start	End
0.5	0.8
4.5	5
8.6	9.4
9.8	11

Fig. S16 The (+)-HR-ESI-MS report of compound **2**, page 1.

User Spectra

Fragmentor Voltage	Collision Energy	Ionization Mode
135	0	Esi

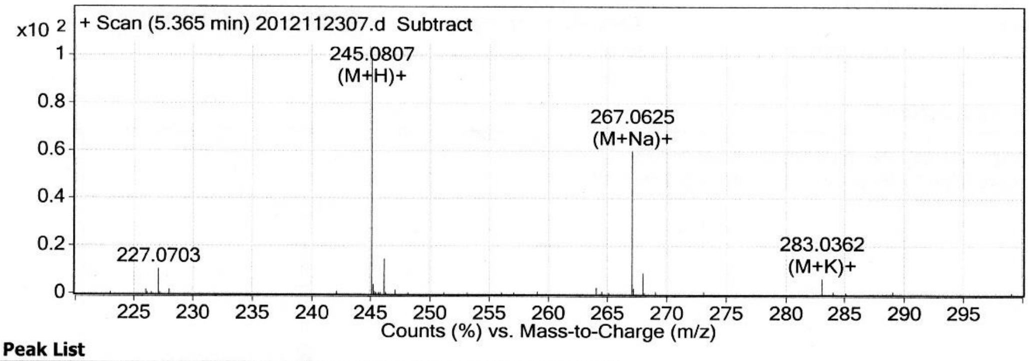

Peak List

m/z	z	Abund	Formula	Ion
107.0412		75702		
151.0383		40388		
169.1102		70519		
191.0921		46195		
227.0703		43443		
245.0807	1	420859	C14 H13 O4	(M+H)+
246.0839	1	62088	C14 H13 O4	(M+H)+
267.0625	1	252517	C14 H12 Na O4	(M+Na)+
268.066	1	36123	C14 H12 Na O4	(M+Na)+
283.0362		26324	C14 H12 K O4	(M+K)+

Formula Calculator Element Limits

Element	Min	Max
C	3	100
H	0	500
O	0	90
N	0	4
S	0	2
Cl	0	0
Br	0	1

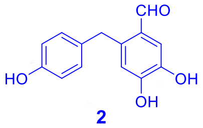

2

Formula Calculator Results

Formula	Best	Mass	Tgt Mass	Diff (ppm)	Ion Species	Score
C14 H12 O4	TRUE	244.0734	244.0736	0.55	C14 H13 O4	99.97
C15 H8 N4		244.0734	244.0749	6.02	C15 H9 N4	99.11
C14 H12 O4	TRUE	244.0733	244.0736	1	C14 H12 Na O4	99.9
C15 H8 N4		244.0733	244.0749	6.48	C15 H8 N4 Na	98.92
C14 H12 O4	TRUE	244.073	244.0736	2.11	C14 H12 K O4	99.39
C15 H8 N4		244.073	244.0749	7.59	C15 H8 K N4	98.23

--- End Of Report ---

Fig. S17 The (+)-HR-ESI-MS report of compound **2**, page 2.

MS Formula Results: + Scan (5.365 min) Sub (2012112307.d)

m/z	Ion	Formula	Abundance
245.0807	(M+H)+	C14 H13 O4	420859.1

Best	Formula (M)	Ion Formula	Calc m/z	Score	Cross S	Mass	Calc Mass	Diff (ppm)	Abs Diff (ppm)	Abund Match	Spacing Mat	Mass Match	m/z	DBE
☑	C14 H12 O4	C14 H13 O4	245.0808	99.97		244.0734	244.0736	0.55	0.55	99.93	99.98	99.99	245.0807	9
☐	C15 H8 N4	C15 H9 N4	245.0822	99.11		244.0734	244.0749	6.02	6.02	98.68	99.96	98.94	245.0807	14

m/z	Ion	Formula	Abundance
267.0625	(M+Na)+	C14 H12 Na O4	252516.6

Best	Formula (M)	Ion Formula	Calc m/z	Score	Cross S	Mass	Calc Mass	Diff (ppm)	Abs Diff (ppm)	Abund Match	Spacing Mat	Mass Match	m/z	DBE
☑	C14 H12 O4	C14 H12 Na O4	267.0628	99.9		244.0733	244.0736	1	1	99.78	99.91	99.97	267.0625	9
☐	C15 H8 N4	C15 H8 N4 Na	267.0641	98.92		244.0733	244.0749	6.48	6.48	98.15	99.78	98.95	267.0625	14

m/z	Ion	Formula	Abundance
283.0362	(M+K)+	C14 H12 K O4	26323.6

Best	Formula (M)	Ion Formula	Calc m/z	Score	Cross S	Mass	Calc Mass	Diff (ppm)	Abs Diff (ppm)	Abund Match	Spacing Mat	Mass Match	m/z	DBE
☑	C14 H12 O4	C14 H12 K O4	283.0367	99.39		244.073	244.0736	2.11	2.11	99.43	98.32	99.9	283.0362	9
☐	C15 H8 N4	C15 H8 K N4	283.0381	98.23		244.073	244.0749	7.59	7.59	97.8	97.83	98.69	283.0362	14

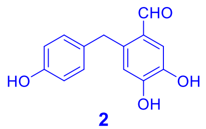

2

page 1

Fig. S18 The (+)-HR-ESI-MS report of compound **2**, page 3.

VNS-600 PROTON TMG-55B IN dmso Dec 8 2012

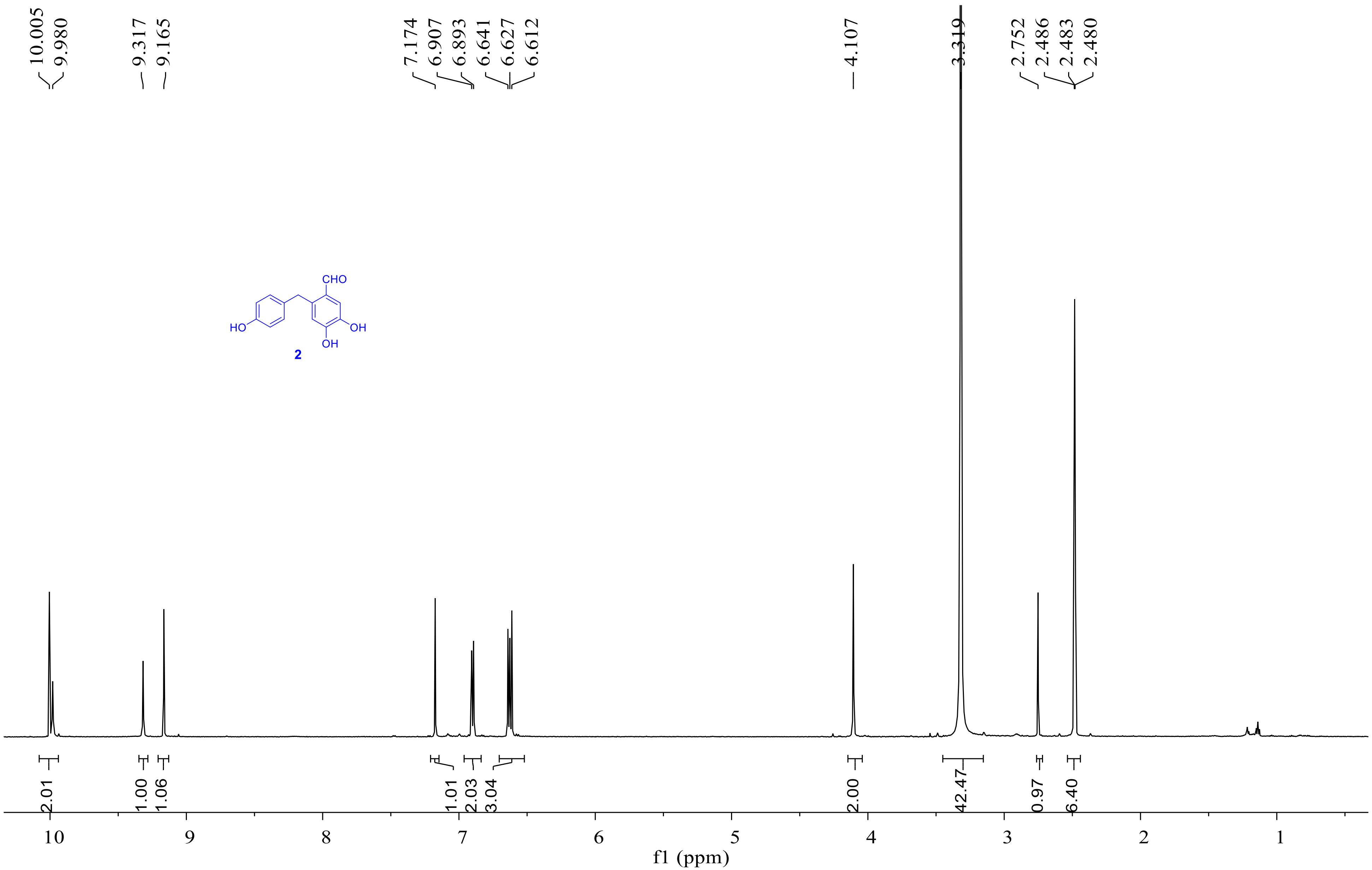

Fig. S19 The ^{1}H NMR spectrum of compound **2** in DMSO-d_6 (600 MHz).

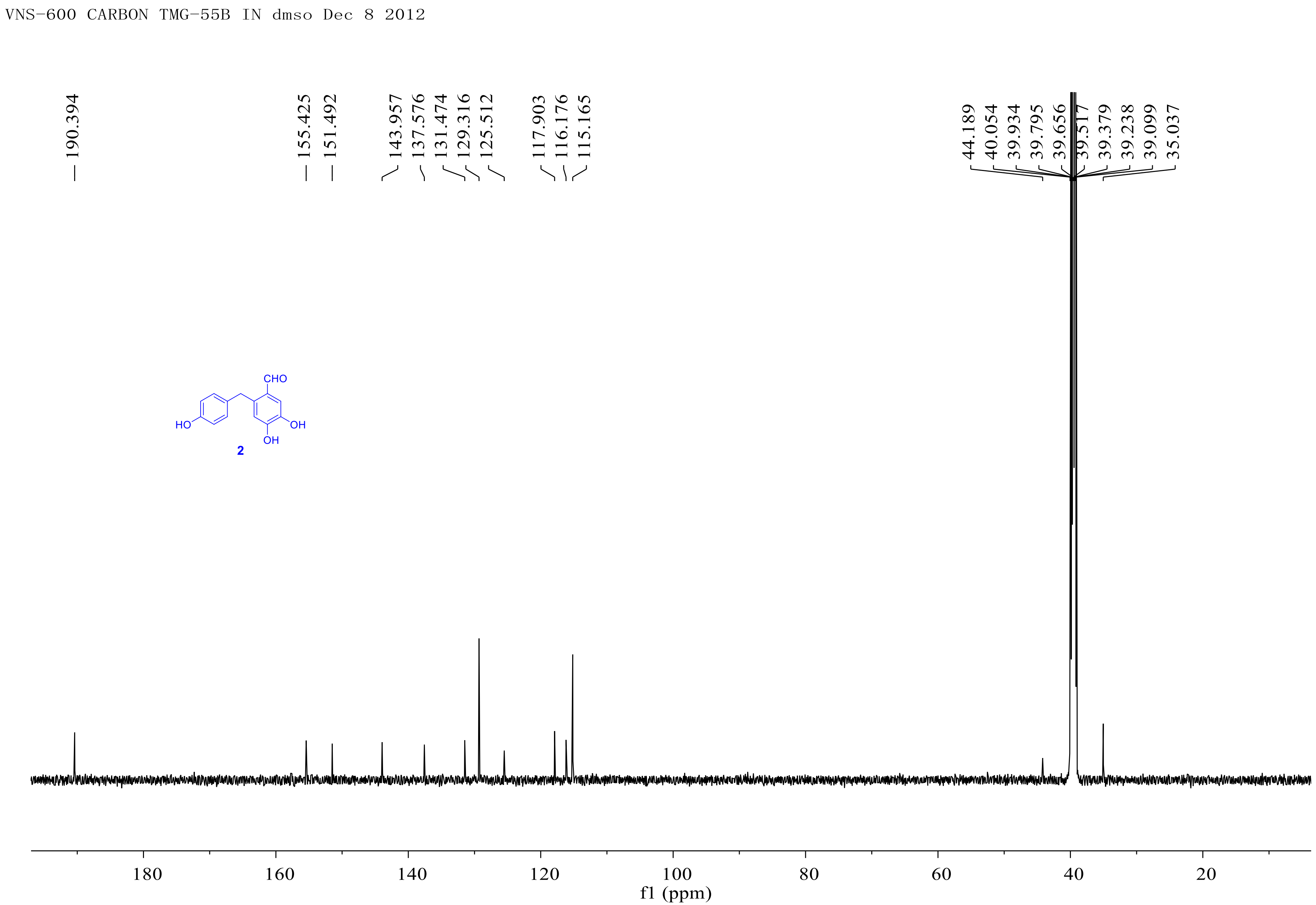

Fig. S20 The ^{13}C NMR spectrum of compound **2** in DMSO-d_6 (150 MHz).

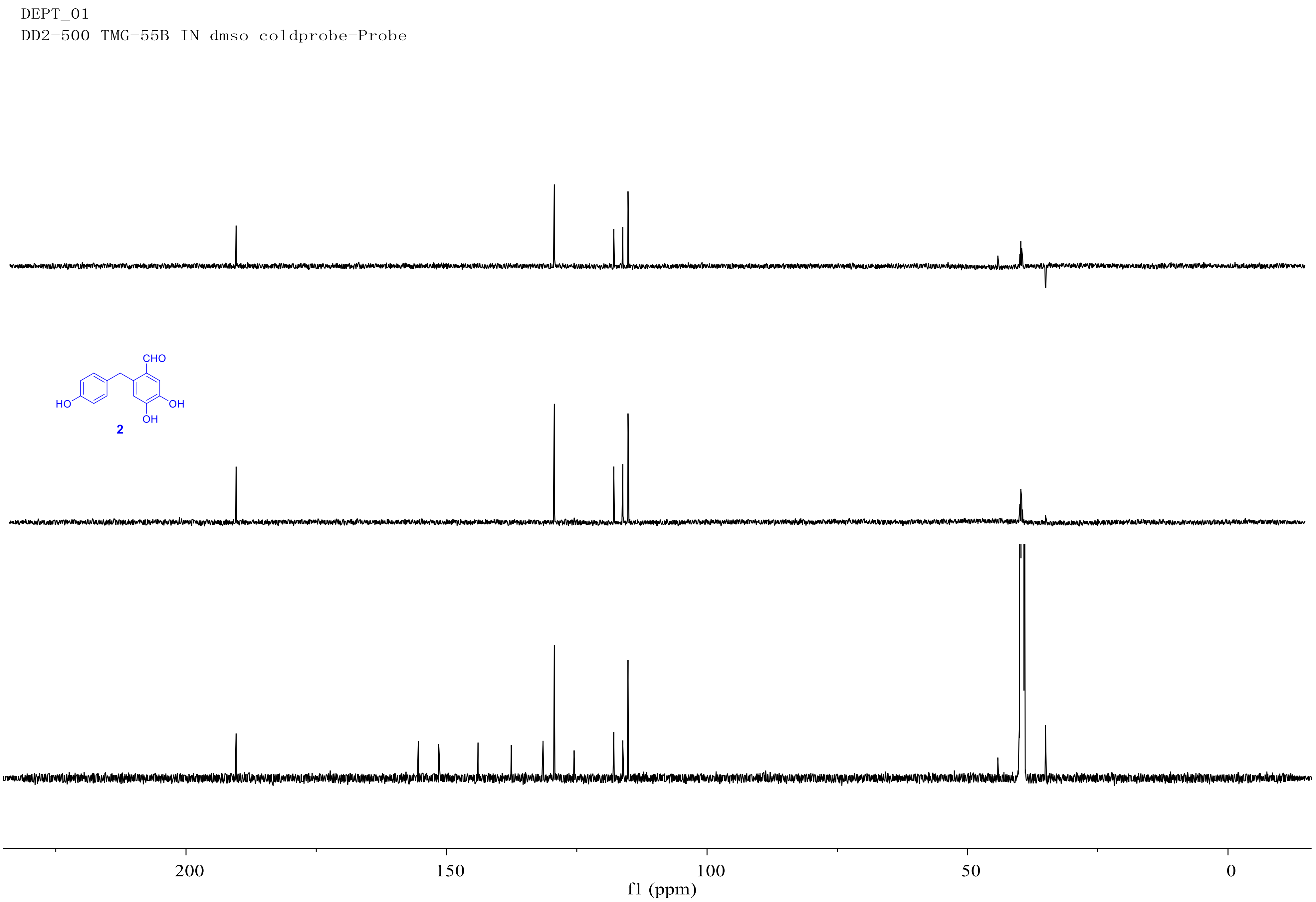

Fig. S21 The DEPT spectrum of compound **2** in DMSO-d_6 (125 MHz).

VNS-600 gCOSY_01 TMG-55B IN dmso Dec 8 2012

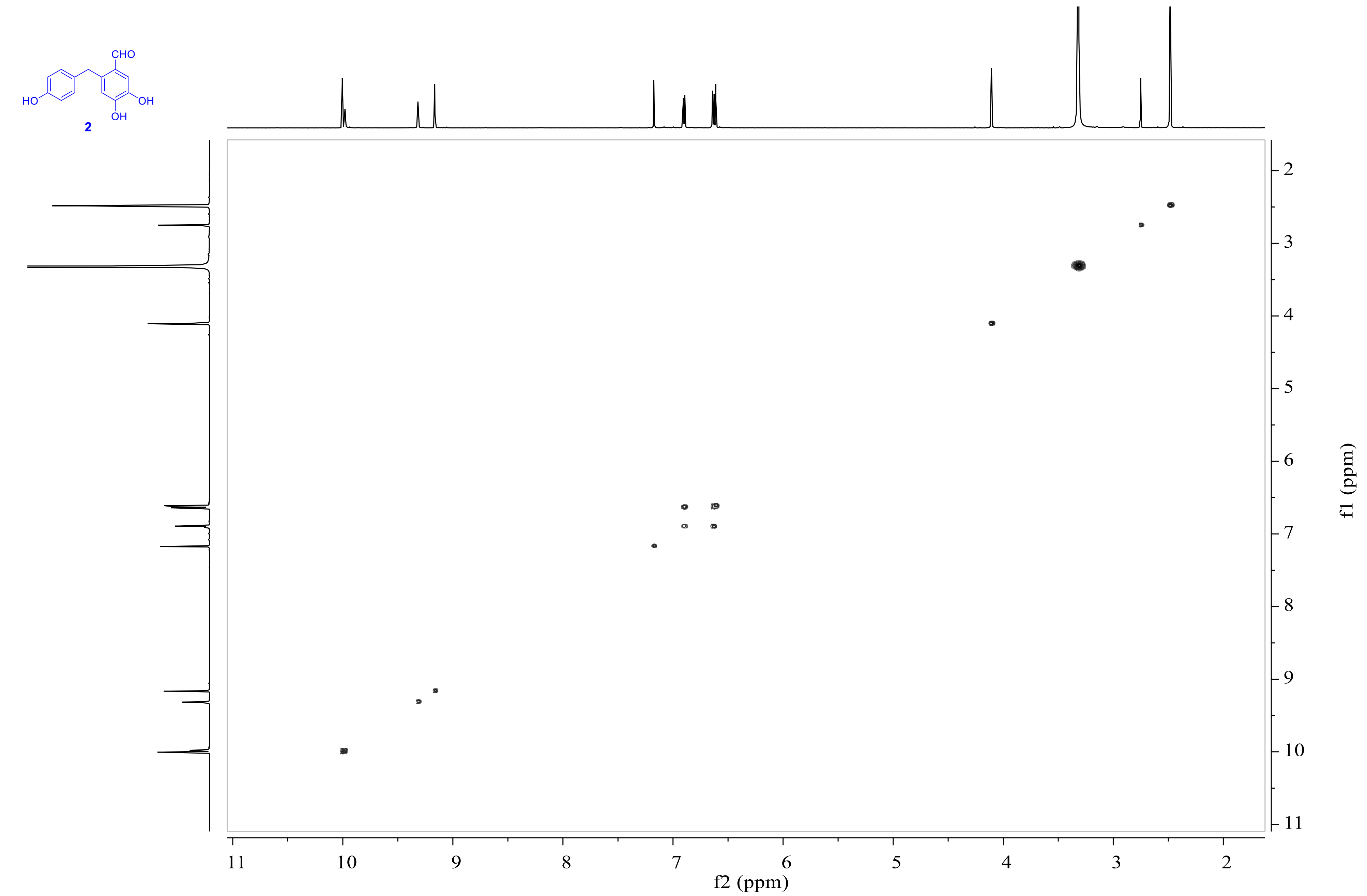

Fig. S22 The ^{1}H-^{1}H COSY spectrum of compound **2** in DMSO-d_6 (600 MHz).

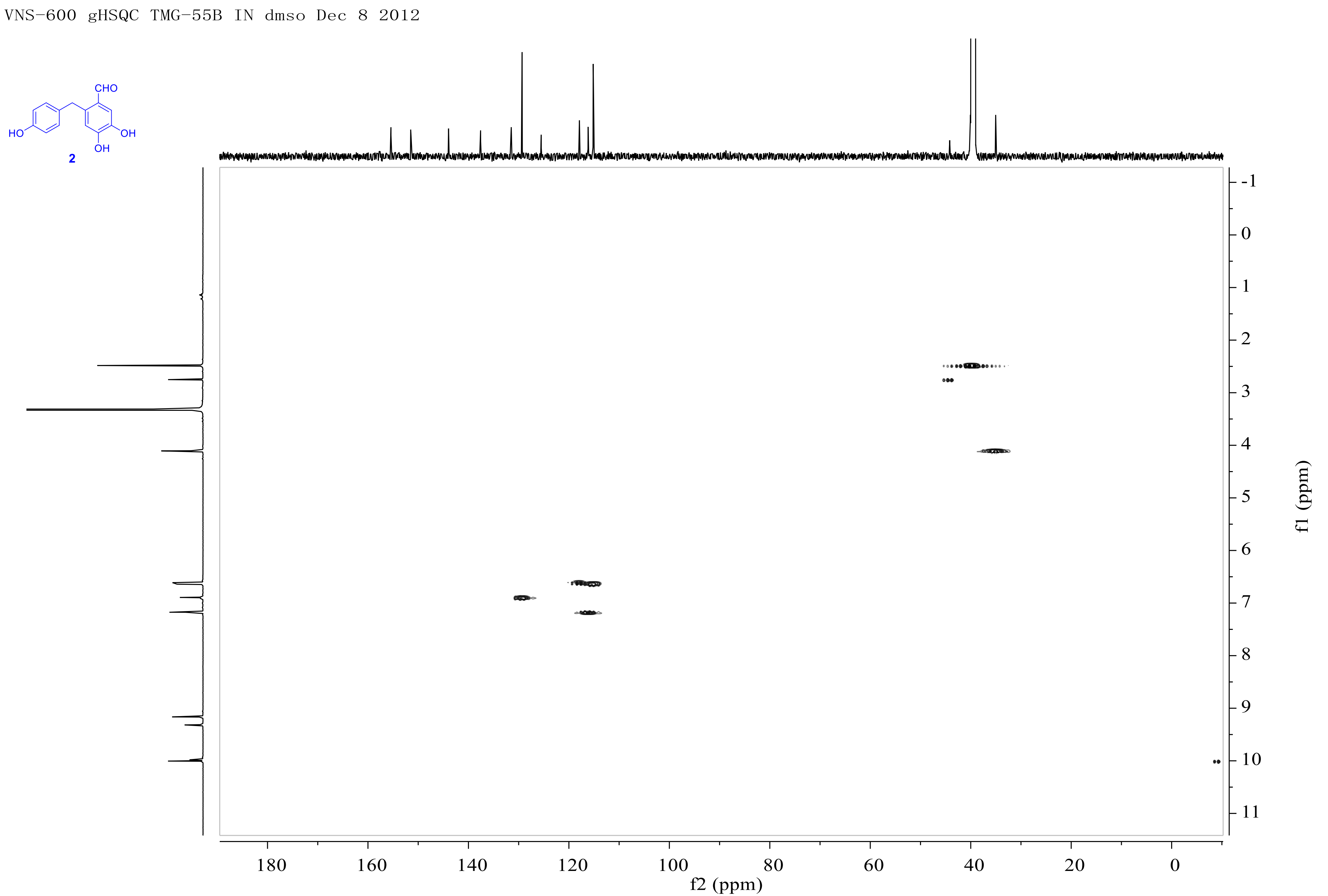

Fig. S23 The HSQC spectrum of compound **2** in DMSO-d_6 (600 MHz for ^{1}H).

VNS-600 gHMBC TMG-55B IN dmso Dec 8 2012

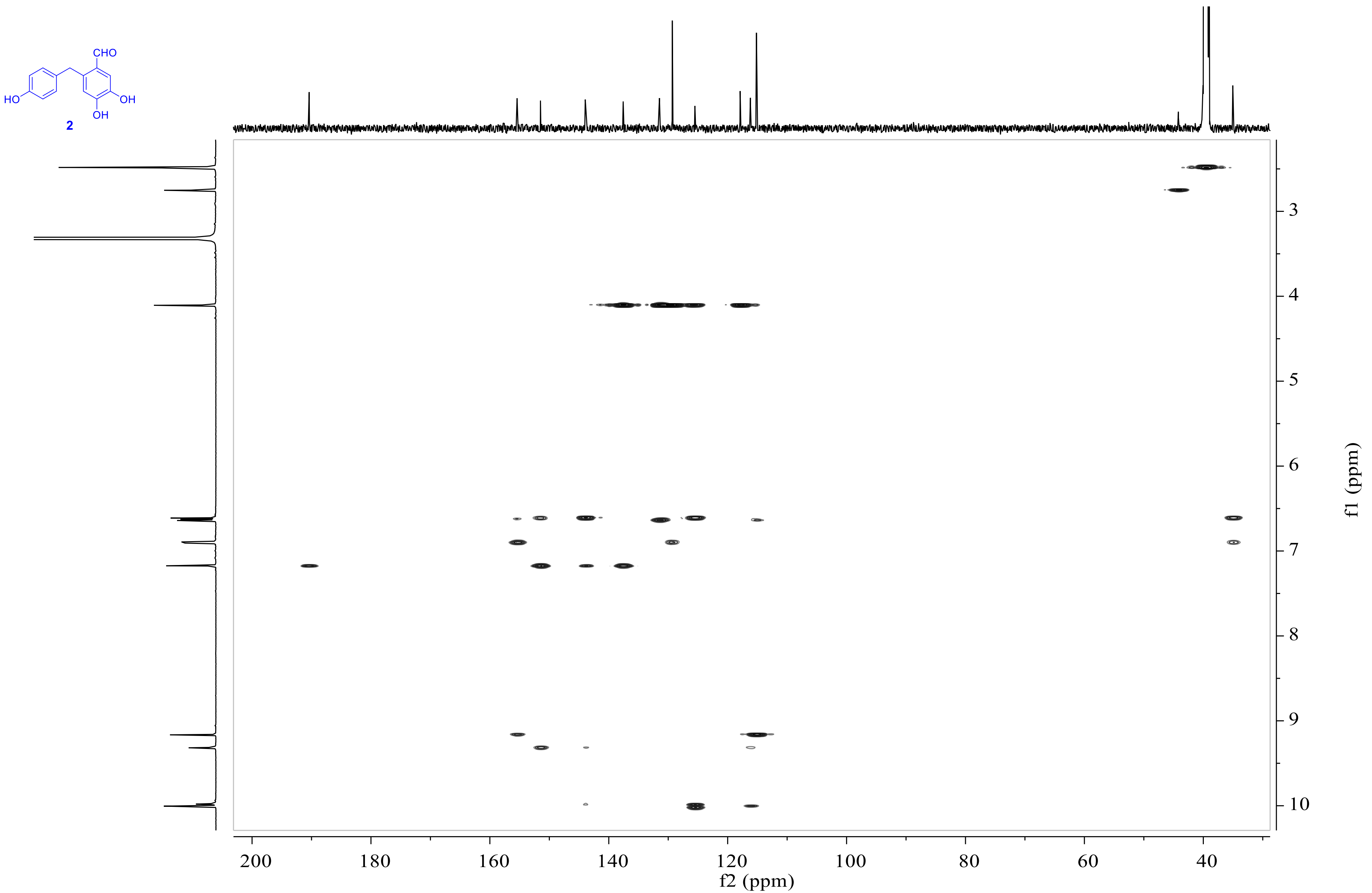

Fig. S24 The HMBC spectrum of compound **2** in DMSO-d_6 (600 MHz for ^{1}H).

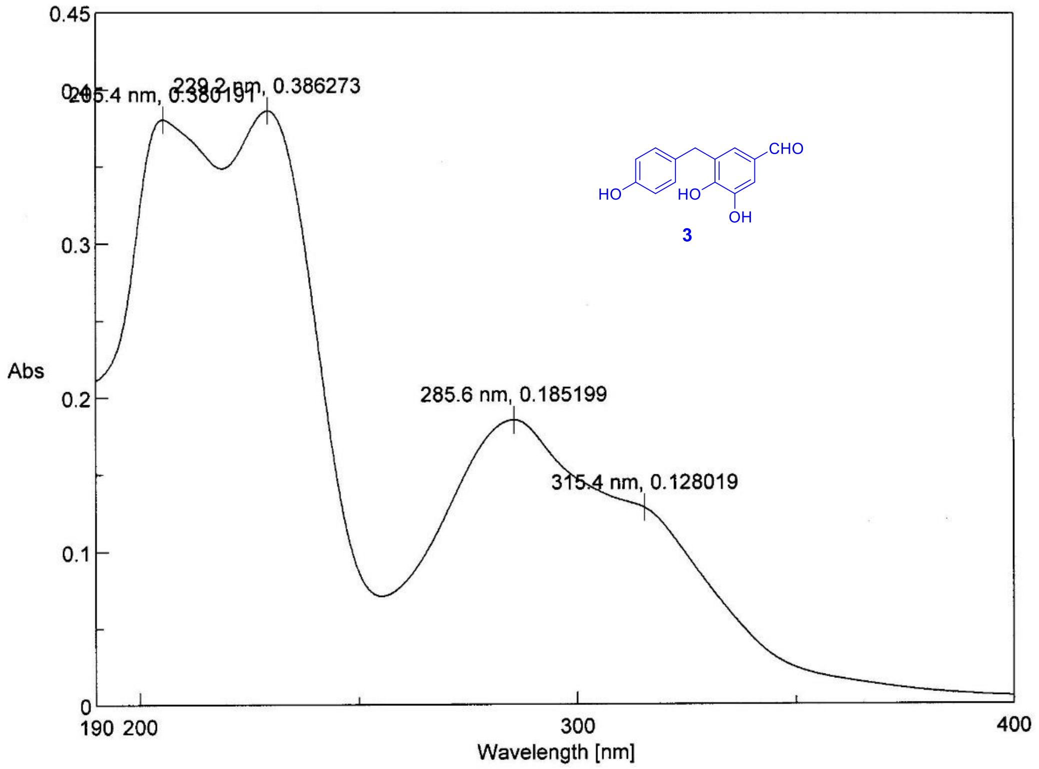

Memory-37

[Comment]
Sample Name TMG-57
Comment 0.02
User
Division UV
Company 324

[Measurement Information]
Instrument Name V-650
Model Name V-650
Serial No. A034461150

Accessory PSC-718
Accessory S/N A001761114
Position 1
Cell Length 10 mm
Temperature 20.01 C
Control Sonsor Holder
Monitor Sensor Holder
Start Mode Start immediately

Photometric Mode Abs
Measurement range 400 - 190 nm
Data pitch 0.2 nm
Band width(UV/Vis) 2.0 nm
Response Medium
Scanning speed 200 nm/min
Source Change 340 nm
Light Source D2/WI
Filter Exchange Step
Correction Baseline

[Data Information]
Creation Date 2014-2-26 10:15

Data array type Linear data array
Horizontal Wavelength [nm]
Vertical Abs
Start 400 nm
End 190 nm
Data pitch 0.2 nm
Data points 1051

Fig. S25 The UV spectrum of compound **3**.

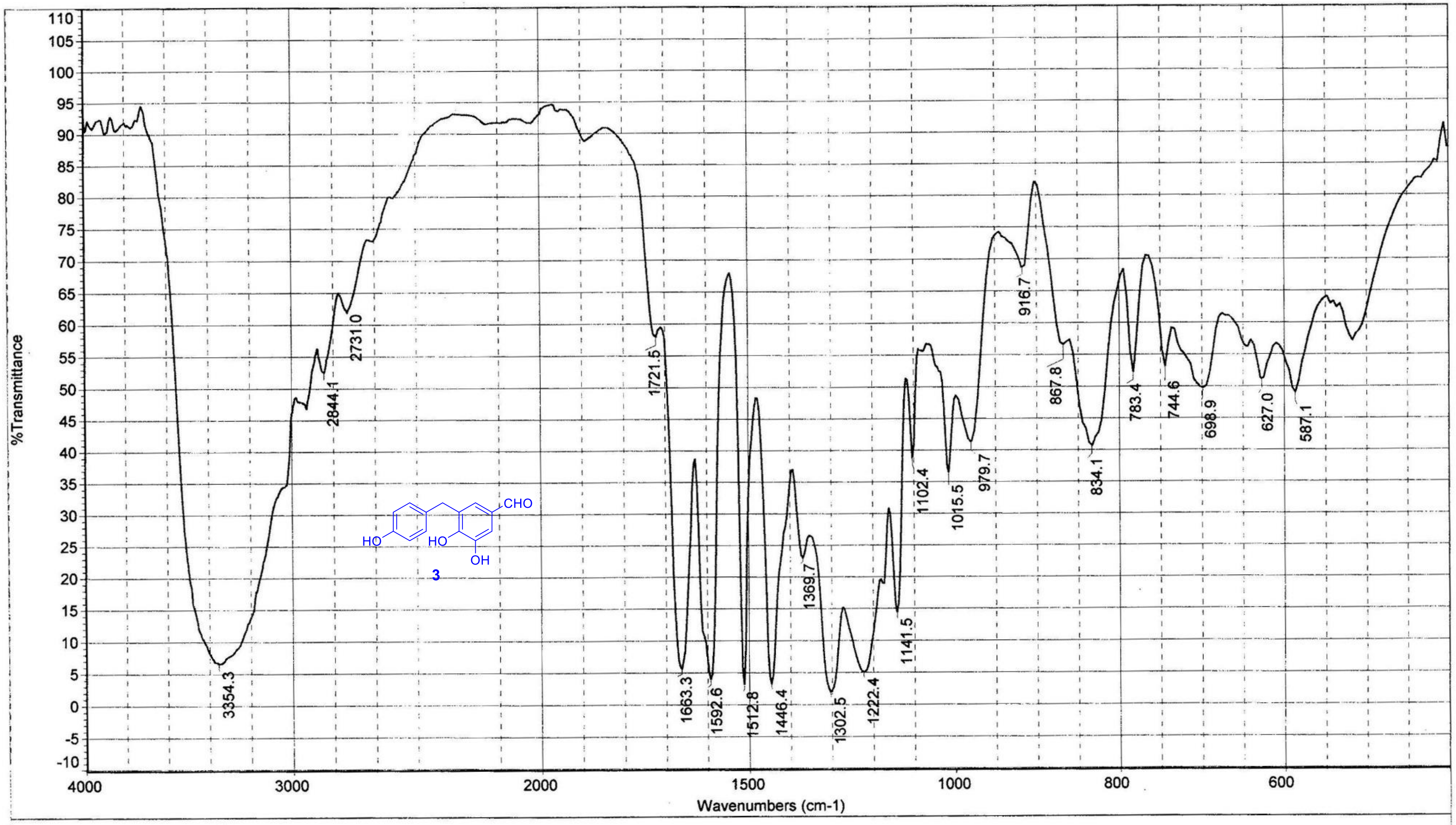

日期: 星期五 2月 21 09:19:58 2014 (GMT+08:0Sample Name ： TMG - 57 (显微镜透射法 FT- IR Microscope Transmission)

扫描次数: 100 傅里叶变换显微镜红外(FT-IR Microscope)：Centaurμs

分辨率: 8.000 美国热电公司(Thermo)傅里叶变换红外光谱仪:Nicolet 5700

Fig. S26 The IR spectrum of compound **3**.

Single Mass Spectrum Deconvolution Report

**Analysis Name:**	guoql071.d	**Instrument:**	LC-MSD-Trap-SL	**Print Date:**	10/30/2012 1:50:44 PM
**Method:**	TEST.MS	**Operator:**	Operator	**Acq. Date:**	10/30/2012 1:00:12 PM
**Sample Name:**	TMG-57				
**Analysis Info:**					

Acquisition Parameter:

Mass Range Mode	Std/Normal	Trap Drive	45.5	Scan Begin	100 m/z
Ion Polarity	Positive	Octopole RF Amplitude	152.8 Vpp	Scan End	700 m/z
Ion Source Type	ESI	Capillary Exit	-102.3 Volt	Averages	5 Spectra
Dry Temp (Set)	330 °C	Skimmer	-40.0 Volt	Max. Accu Time	200000 µs
Nebulizer (Set)	15.00 psi	Oct 1 DC	-12.00 Volt	ICC Target	20000
Dry Gas (Set)	6.00 l/min	Oct 2 DC	-1.70 Volt	Charge Control	on

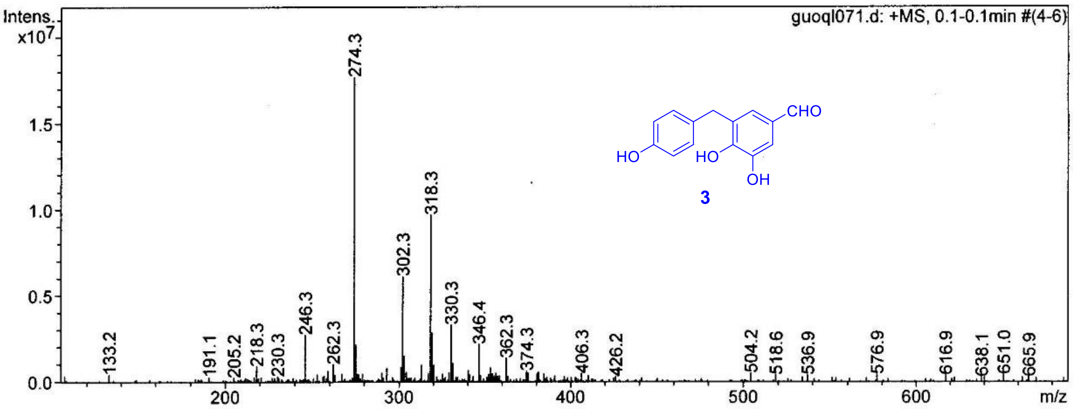

Component	Molecular Mass	Molecule	Absolute Abundance	Relative Abundance

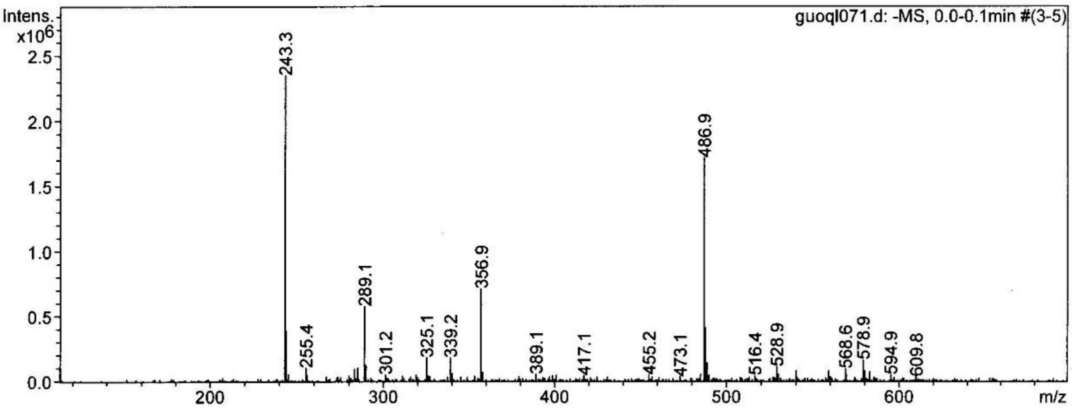

Component	Molecular Mass	Molecule	Absolute Abundance	Relative Abundance

Fig. S27 The ESI-MS of compound **3**.

Qualitative Analysis Report

**Data Filename**	2014022608.d	**Sample Name**	TMG-57
**Sample Type**	Sample	**Position**	P1-C8
**Instrument Name**	Instrument 1	**User Name**	
**Acq Method**		**IRM Calibration Status**	Success
**DA Method**	TEST LCMS.m	**Comment**	

User Chromatograms

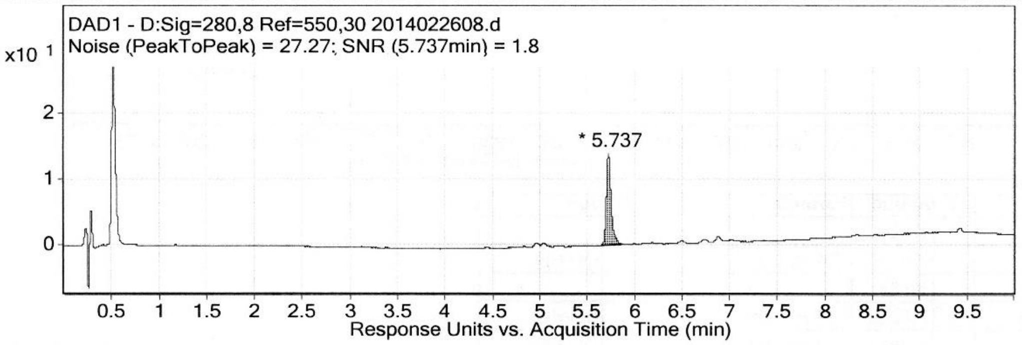

Integration Peak List

Peak	Start	RT	End	Height	Area	Area %	Signal To Noise
1	5.648	5.737	5.879	14.02	48.3	100	1.8

Noise Measurements

Noise Type	Signal Definition	Noise Multiplier	Noise Value
Peak-to-Peak	Area	1	27.26650238

Noise Regions

Start	End
0.5	1
5	5.3
9.99	11

Fragmentor Voltage 135 **Collision Energy** 0 **Ionization Mode** ESI

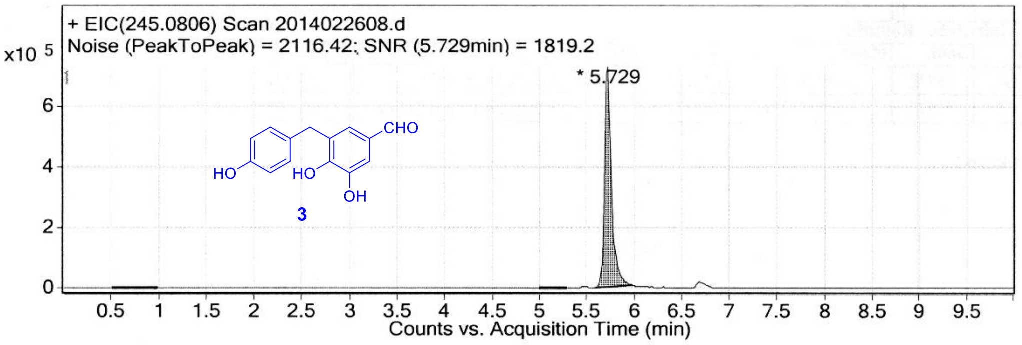

Integration Peak List

Peak	Start	RT	End	Height	Area	Area %	Signal To Noise
1	5.584	5.729	5.987	727229	3850093	100	1819.2

Noise Measurements

Noise Type	Signal Definition	Noise Multiplier	Noise Value
Peak-to-Peak	Area	1	2116.415283

Noise Regions

Start	End
0.5	1
5	5.3
9.99	11

User Spectra

Agilent Technologies Page 1 of 2 Printed at: 10:22 AM on: 2/26/2014

Fig. S28 The (+)-HR-ESI-MS report of compound **3**, page 1.

Qualitative Analysis Report

Fragmentor Voltage	Collision Energy	Ionization Mode
135	0	Esi

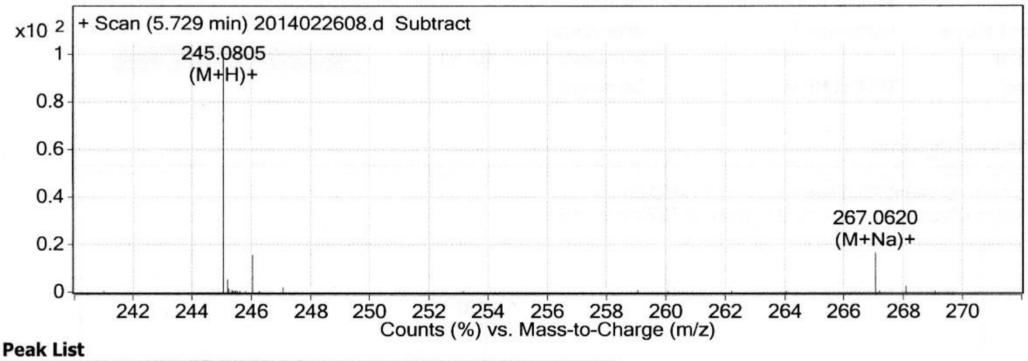

Peak List

m/z	z	Abund	Formula	Ion
163.039		37818		
245.0805	1	729872	C14 H13 O4	(M+H)+
245.2142		38989		
246.0833	1	116050	C14 H13 O4	(M+H)+
267.062		122412	C14 H12 Na O4	(M+Na)+
922.0096		101546		

Formula Calculator Element Limits

Element	Min	Max
C	3	100
H	0	500
O	0	90
N	0	5
S	0	5
Cl	0	2
Br	0	0
Si	0	0
F	0	0
P	0	0

Formula Calculator Results

Formula	Best	Mass	Tgt Mass	Diff (ppm)	Ion Species	Score
C14 H12 O4	TRUE	244.0732	244.0736	1.57	C14 H13 O4	99.92
C14 H12 O4	TRUE	244.0728	244.0736	3.29	C14 H12 Na O4	99.44

--- End Of Report ---

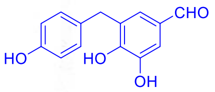

3

Agilent Technologies   Page 2 of 2   Printed at: 10:22 AM on: 2/26/2014

Fig. S29 The (+)-HR-ESI-MS report of compound **3**, page 2.

MS Formula Results: + Scan (5.729 min) Sub (2014022608.d)

m/z	Ion	Formula	Abundance
245.0805	(M+H)+	C14 H13 O4	729871.6

Best	Formula (M)	Ion Formula	Calc m/z	Score	Cross S	Mass	Calc Mass	Diff (ppm)	Abs Diff (ppm)	Abund Match	Spacing Mat	Mass Match	m/z	DBE
☑	C14 H12 O4	C14 H13 O4	245.0808	99.92		244.0732	244.0736	1.57	1.57	99.96	99.88	99.93	245.0805	9

m/z	Ion	Formula	Abundance
267.062	(M+Na)+	C14 H12 Na O4	122412.1

Best	Formula (M)	Ion Formula	Calc m/z	Score	Cross S	Mass	Calc Mass	Diff (ppm)	Abs Diff (ppm)	Abund Match	Spacing Mat	Mass Match	m/z	DBE
☑	C14 H12 O4	C14 H12 Na O4	267.0628	99.44		244.0728	244.0736	3.29	3.29	99.71	98.56	99.73	267.062	9

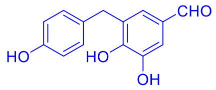

3

page 1

Fig. S30 The (+)-HR-ESI-MS report of compound **3**, page 3.

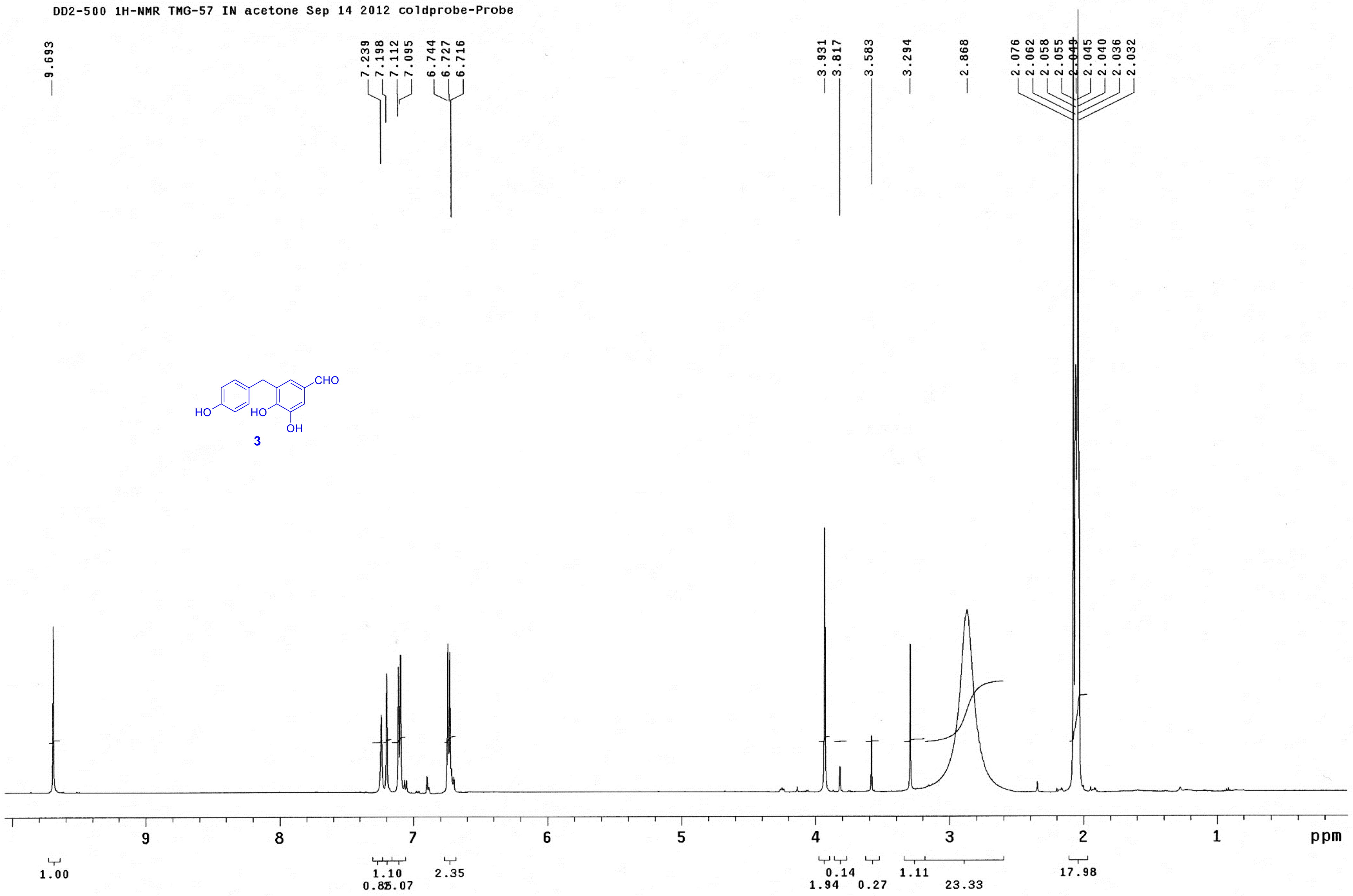

Fig. S31 The ^{1}H NMR spectrum of compound **3** in acetone-d_6 (500 MHz).

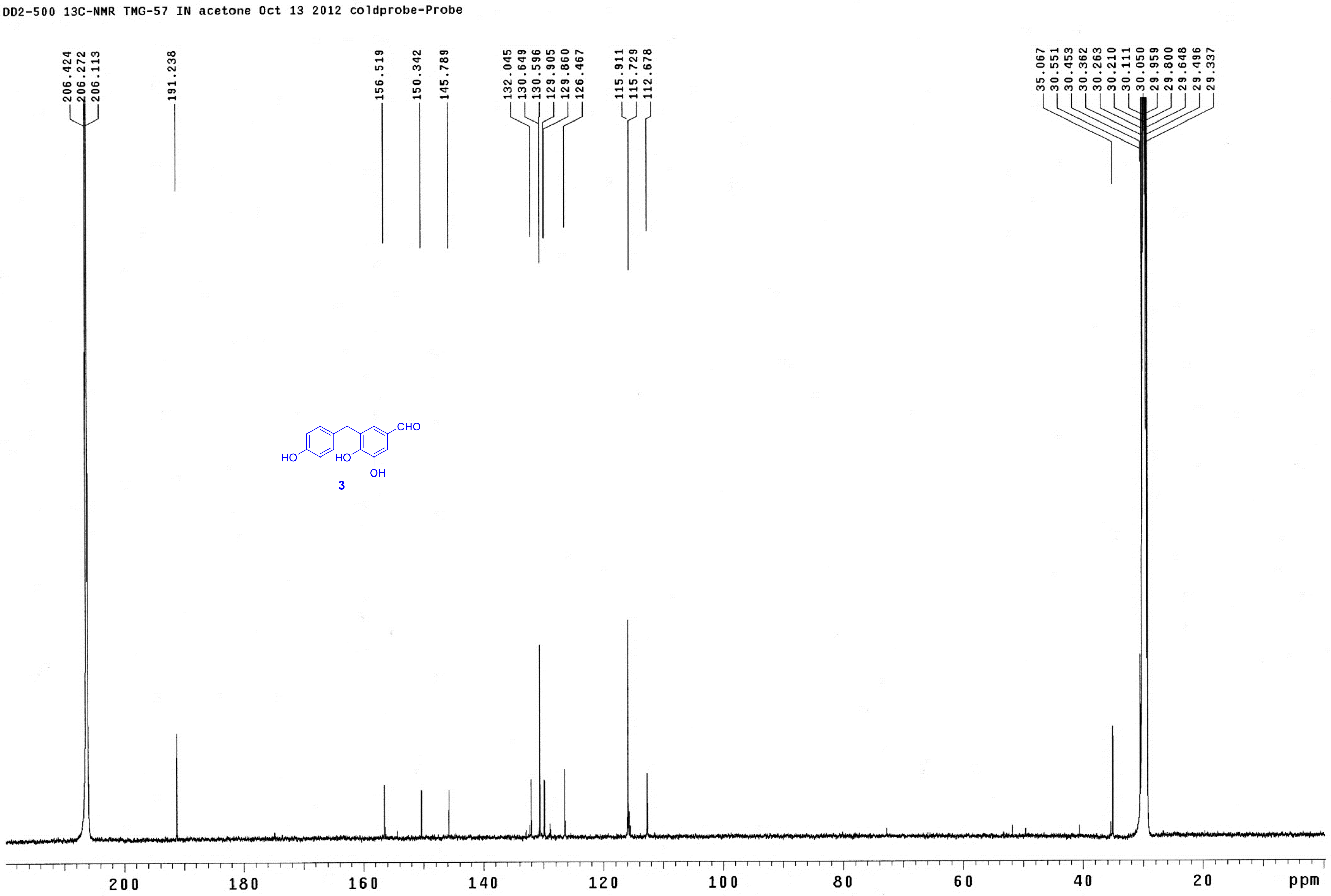

Fig. S32 The ^{13}C NMR spectrum of compound **3** in acetone-d_6 (125 MHz).

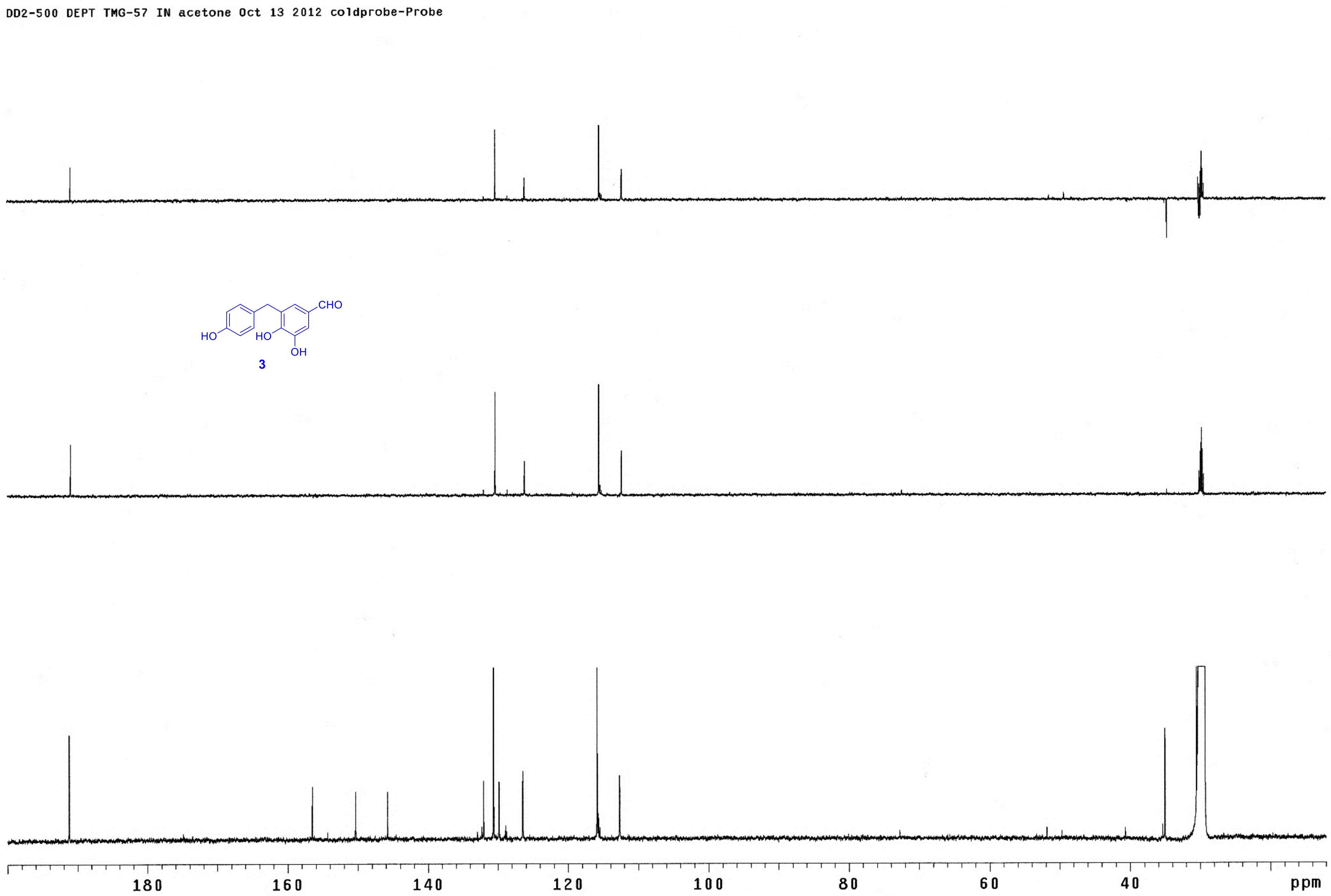

Fig. S33 The DEPT spectrum of compound **3** in acetone-d_6 (125 MHz).

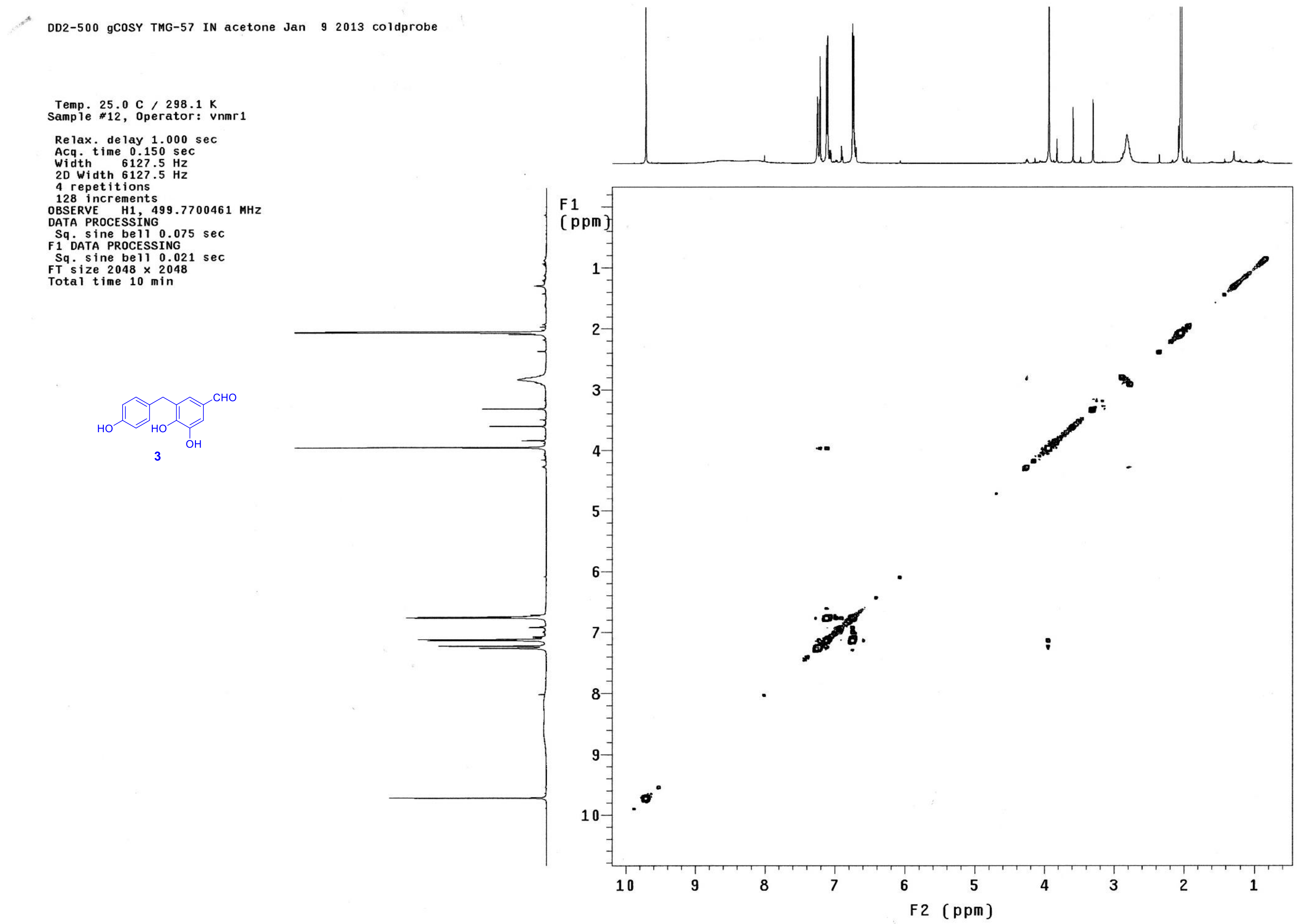

Fig. S34 The ^{1}H-^{1}H COSY spectrum of compound **3** in acetone-d_6 (500 MHz).

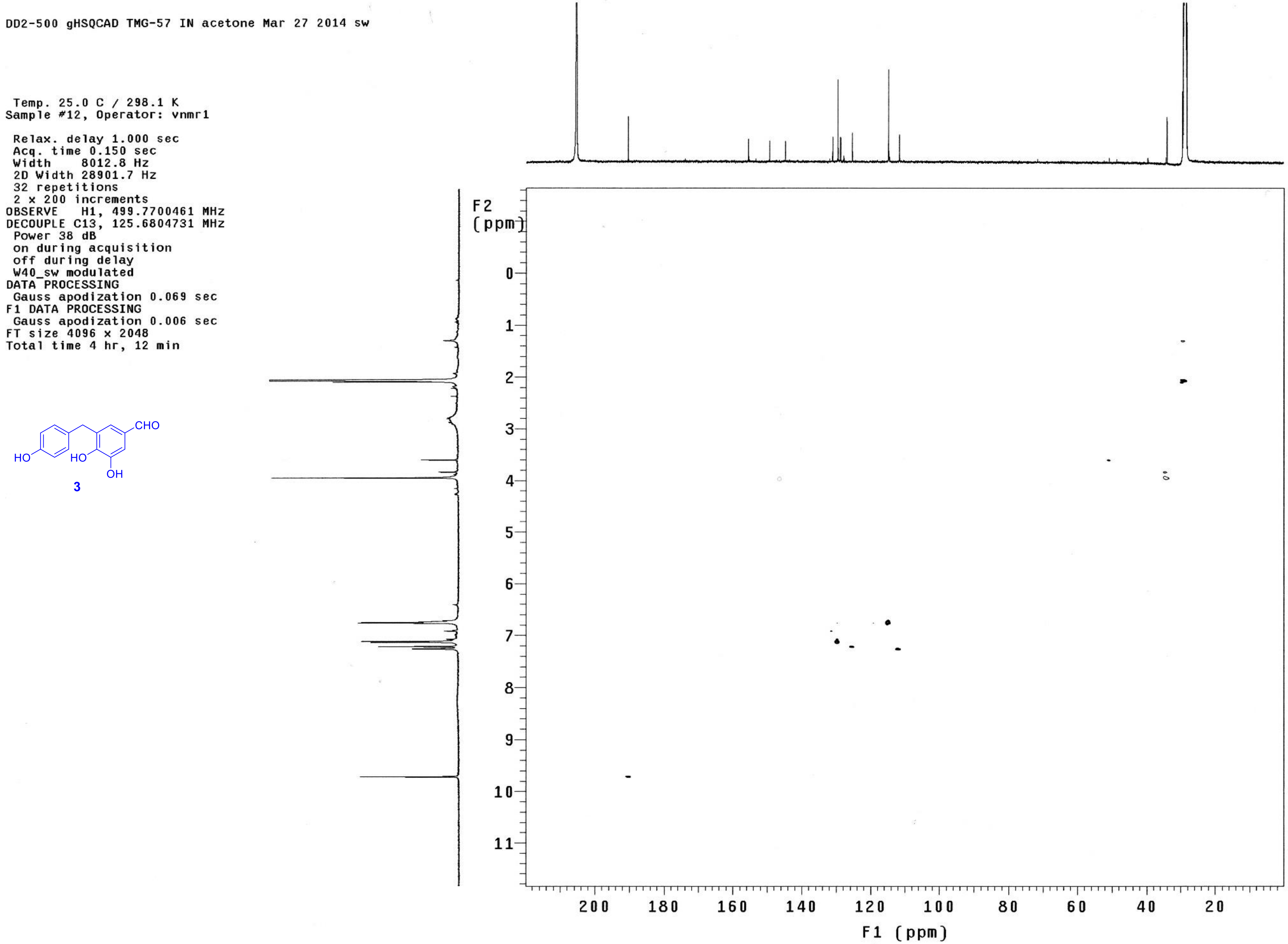

Fig. S35 The HSQC spectrum of compound **3** in acetone-d_6 (500 MHz for ^{1}H).

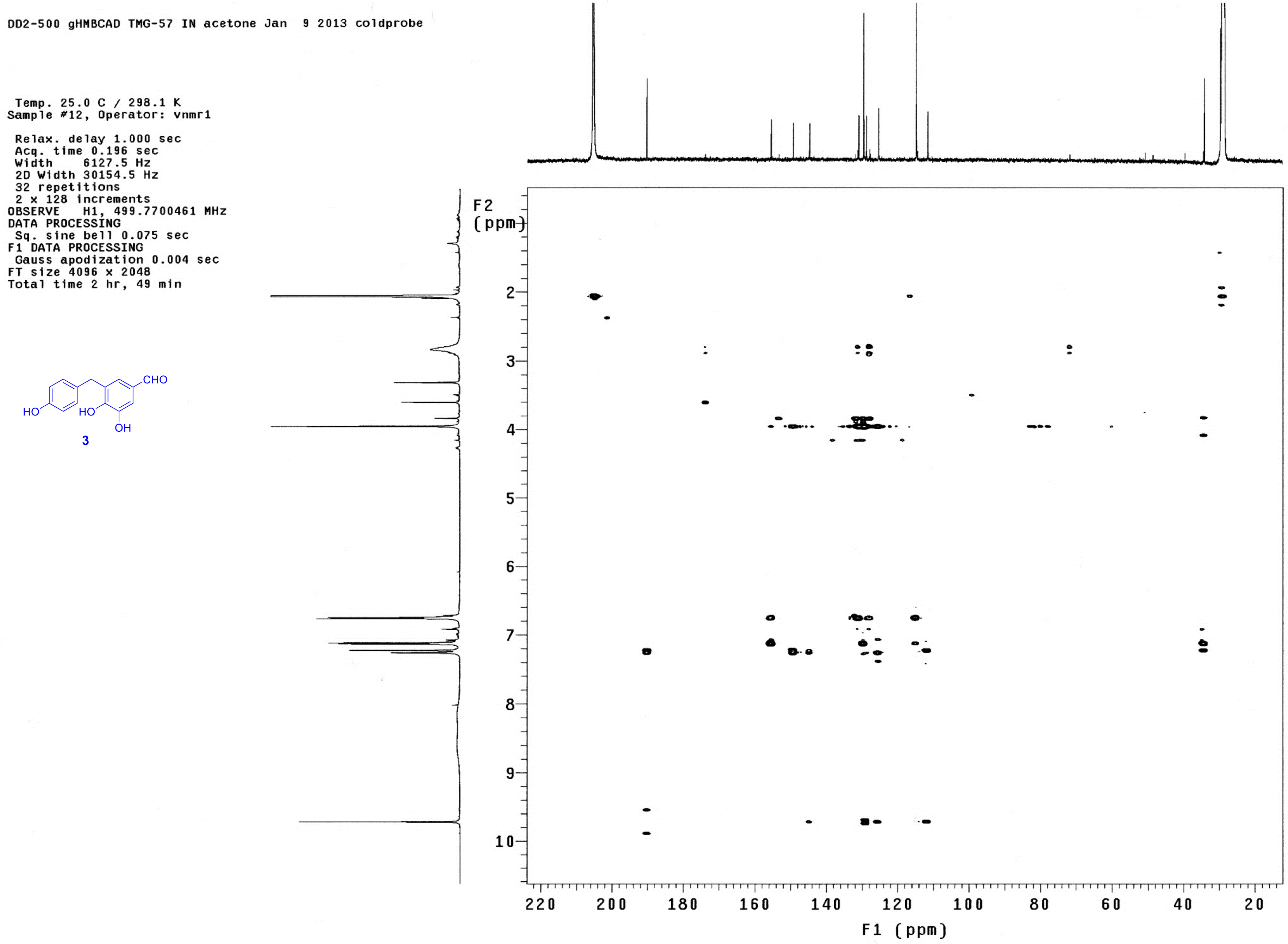

Fig. S36 The HMBC spectrum of compound **3** in acetone-d_6 (500 MHz for ^{1}H).

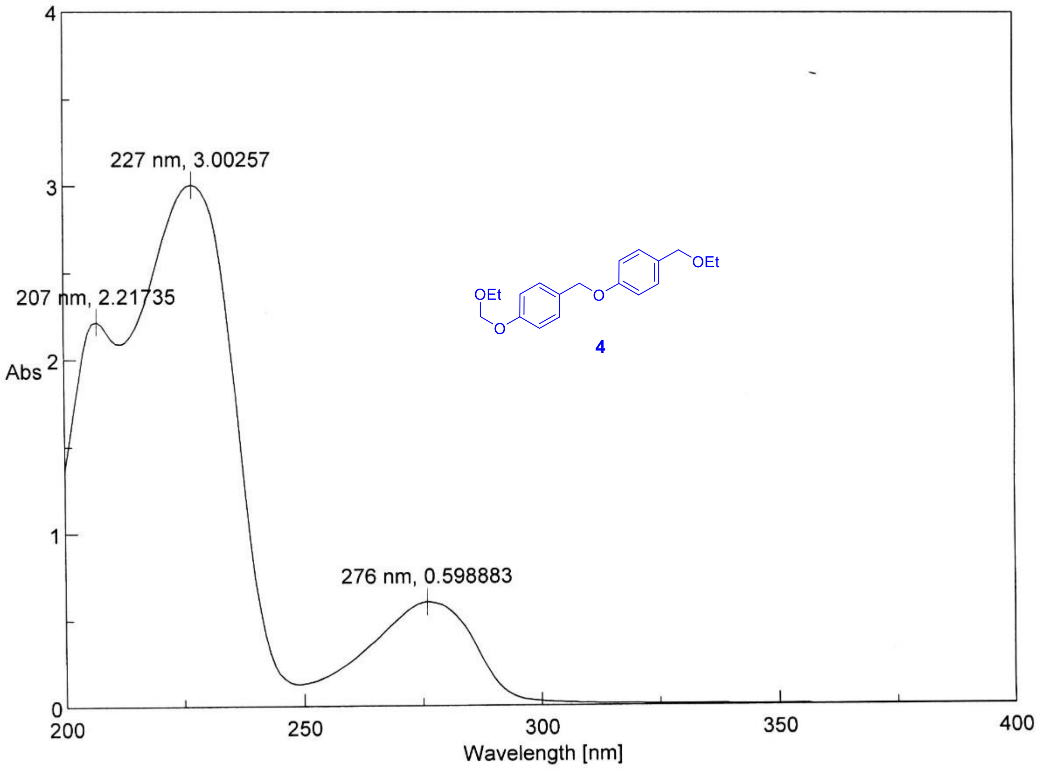

Memory-9

[Comment]
Sample Name wyn-113
Comment MeOH
User 王亚男
Division UV
Company 324

[Measurement Information]
Instrument Name V-650
Model Name V-650
Serial No. A034461150

Accessory PSC-718
Accessory S/N A001761114
Position 1
Cell Length 10 mm
Temperature 20.00 C
Control Sonsor Holder
Monitor Sensor Holder
Start Mode Start immediately

Photometric Mode Abs
Measurement range 400 - 200 nm
Data pitch 1 nm
Band width(UV/Vis) 1.0 nm
Response Medium
Scanning speed 200 nm/min
Source Change 340 nm
Light Source D2/WI
Filter Exchange Step
Correction Baseline

[Data Information]
Creation Date 2012-5-2 13:40

Data array type Linear data array
Horizontal Wavelength [nm]
Vertical Abs
Start 400 nm
End 200 nm
Data pitch 1 nm
Data points 201

Fig. S37 The UV spectrum of compound **4**.

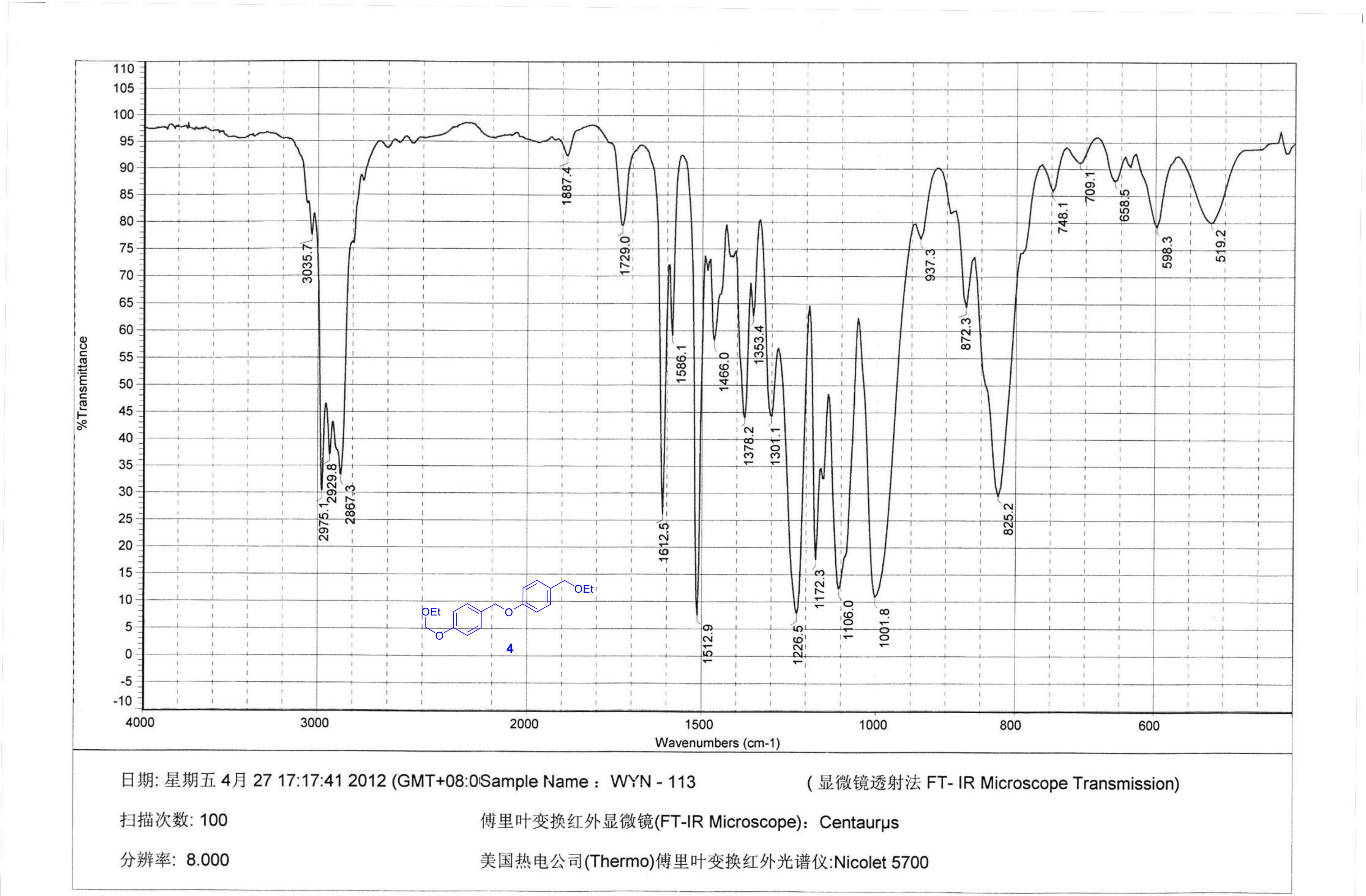

Fig. S38 The IR spectrum of compound **4**.

Single Mass Spectrum Deconvolution Report

Analysis Name: jingh019.d **Instrument:** LC-MSD-Trap-SL **Print Date:** 7/26/2011 5:24:01 AM
Method: TEST.MS **Operator:** Operator **Acq. Date:** 7/26/2011 5:17:44 AM
Sample Name: WYN-113
Analysis Info:

Acquisition Parameter:

Mass Range Mode	Std/Normal	Trap Drive	41.8	Scan Begin	100 m/z
Ion Polarity	Positive	Octopole RF Amplitude	142.5 Vpp	Scan End	400 m/z
Ion Source Type	ESI	Capillary Exit	-100.4 Volt	Averages	5 Spectra
Dry Temp (Set)	330 °C	Skimmer	-40.0 Volt	Max. Accu Time	200000 µs
Nebulizer (Set)	15.00 psi	Oct 1 DC	-12.00 Volt	ICC Target	20000
Dry Gas (Set)	5.00 l/min	Oct 2 DC	-1.70 Volt	Charge Control	on

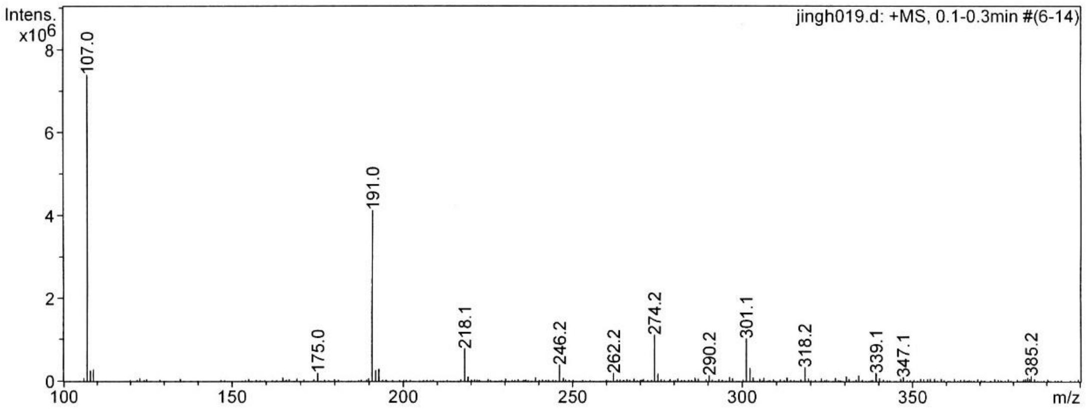

Component	Molecular Mass	Molecule	Absolute Abundance	Relative Abundance

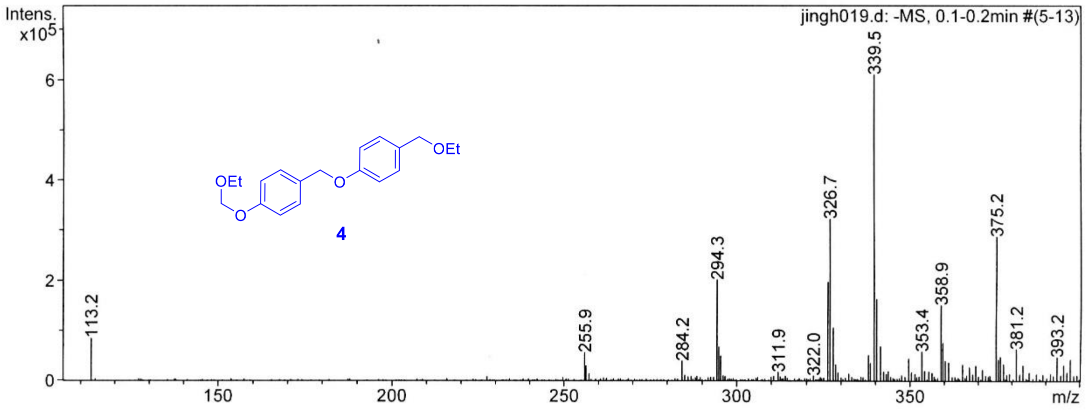

Component	Molecular Mass	Molecule	Absolute Abundance	Relative Abundance

Fig. S39 The ESI-MS of compound **4**.

Qualitative Analysis Report

**Data Filename**	2012041902.d	**Sample Name**	WYN-113
**Sample Type**	Sample	**Position**	P1-D6
**Instrument Name**	Instrument 1	**User Name**	
**Acq Method**		**IRM Calibration Status**	Success
**DA Method**	TEST LCMS.m	**Comment**	

User Chromatograms

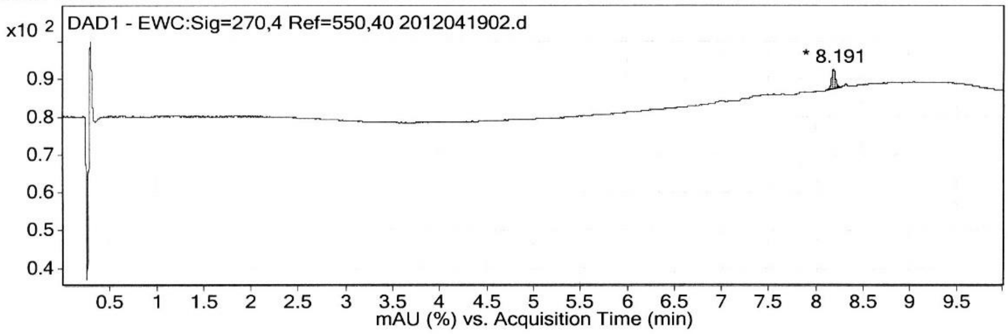

Integration Peak List

Peak	Start	RT	End	Height	Area	Area %
1	8.114	8.191	8.273	2.28	6.58	100

Fragmentor Voltage 135 **Collision Energy** 0 **Ionization Mode** ESI

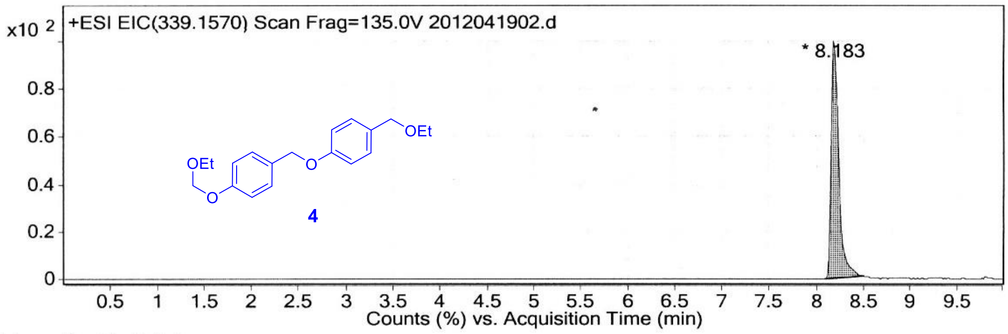

Integration Peak List

Peak	Start	RT	End	Height	Area	Area %
1	8.086	8.183	8.505	1086827	5914907	100

User Spectra

Fragmentor Voltage	Collision Energy	Ionization Mode
135	0	Esi

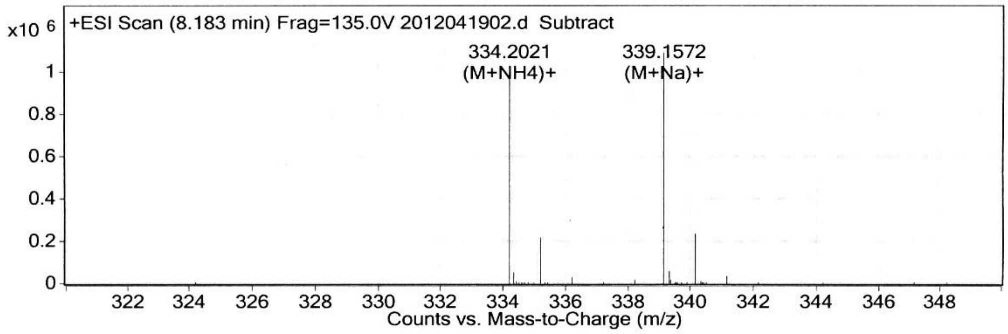

Fig. S40 The (+)-HR-ESI-MS report of compound **4**, page 1.

Qualitative Analysis Report

Peak List

m/z	z	Abund	Formula	Ion
165.0912	1	790140		
279.1593	1	1185354		
280.1627	1	213655		
301.142	1	768008		
302.1448	1	144202		
334.2021	1	976937	C19 H28 N O4	(M+NH4)+
335.2051	1	216853	C19 H28 N O4	(M+NH4)+
339.1572	1	1089143	C19 H24 Na O4	(M+Na)+
340.1608	1	237608	C19 H24 Na O4	(M+Na)+
579.2944	1	279295		

Formula Calculator Element Limits

Element	Min	Max
C	3	100
H	0	120
O	0	30
N	0	5
S	0	2
Cl	0	0

Formula Calculator Results

Formula	Best	Mass	Tgt Mass	Diff (ppm)	Ion Species	Score
C19 H24 O4	TRUE	316.168	316.1675	-1.74	C19 H24 Na O4	99.93
C20 H20 N4		316.168	316.1688	2.48	C20 H20 N4 Na	99.8
C16 H28 O4 S		316.168	316.1708	8.92	C16 H28 Na O4 S	97.73
C20 H20 N4		316.1683	316.1688	1.51	C20 H24 N5	99.88
C19 H24 O4	TRUE	316.1683	316.1675	-2.71	C19 H28 N O4	99.87
C16 H28 O4 S		316.1683	316.1708	7.94	C16 H32 N O4 S	97.85

--- End Of Report ---

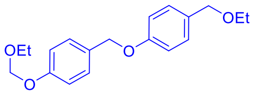

4

Fig. S41 The (+)-HR-ESI-MS report of compound **4**, page 2.

MS Formula Results: + Scan (8.183 min) Sub (2012041902.d)

m/z	Ion	Formula	Abundance
334.2021	(M+NH4)+	C19 H28 N O4	976936.7

Best	Formula (M)	Ion Formula	Calc m/z	Score	Cross S	Mass	Calc Mass	Diff (ppm)	Abs Diff (ppm)	Abund Match	Spacing Mat	Mass Match	m/z	DBE
☐	C20 H20 N4	C20 H24 N5	334.2026	99.88		316.1683	316.1688	1.51	1.51	99.68	100	99.94	334.2021	13
☑	C19 H24 O4	C19 H28 N O4	334.2013	99.87		316.1683	316.1675	-2.71	2.71	99.93	99.96	99.79	334.2021	8
☐	C16 H28 O4 S	C16 H32 N O4 S	334.2047	97.85		316.1683	316.1708	7.94	7.94	95.79	99.61	98.21	334.2021	3

m/z	Ion	Formula	Abundance
339.1572	(M+Na)+	C19 H24 Na O4	1089142.9

Best	Formula (M)	Ion Formula	Calc m/z	Score	Cross S	Mass	Calc Mass	Diff (ppm)	Abs Diff (ppm)	Abund Match	Spacing Mat	Mass Match	m/z	DBE
☑	C19 H24 O4	C19 H24 Na O4	339.1567	99.93		316.168	316.1675	-1.74	1.74	99.92	99.99	99.92	339.1572	8
☐	C20 H20 N4	C20 H20 N4 Na	339.158	99.8		316.168	316.1688	2.48	2.48	99.64	99.92	99.83	339.1572	13
☐	C16 H28 O4 S	C16 H28 Na O4 S	339.1601	97.73		316.168	316.1708	8.92	8.92	96.01	99.61	97.81	339.1572	3

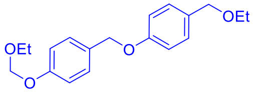

4

page 1

Fig. S42 The (+)-HR-ESI-MS report of compound **4**, page 3.

20120322-WYN-113/1
BRUKER AV-III-500 1H-NMR WYN-113 IN DMSO 2012.03.22
PROTON DMSO E:\\ shijiangong 22

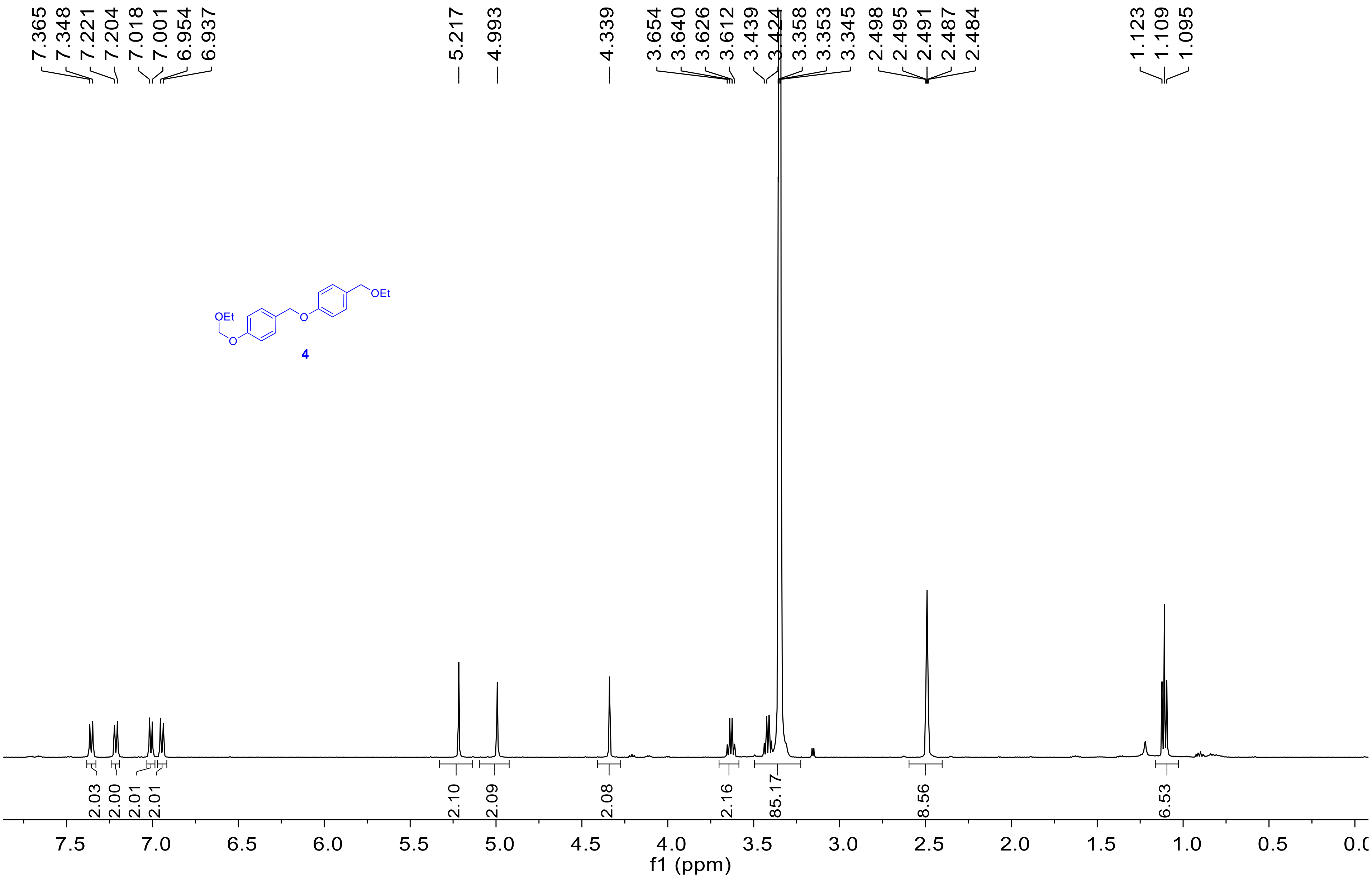

Fig. S43 The ^{1}H NMR spectrum of compound **4** in DMSO-d_6 (500 MHz).

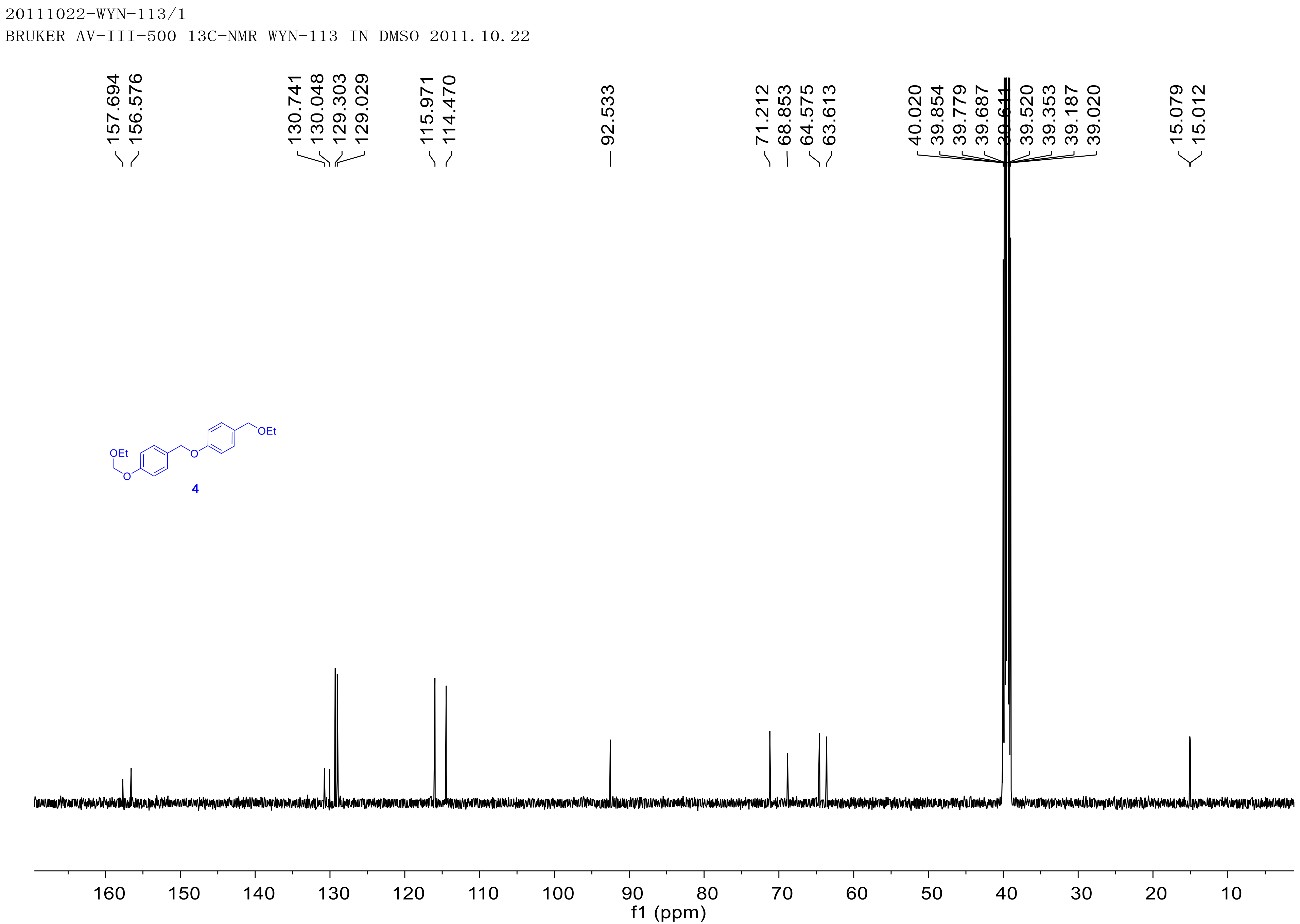

Fig. S44 The ^{13}C NMR spectrum of compound **4** in DMSO-d_6 (125 MHz).

BRUKER AV-III-500 DEPT-NMR WYN-113 IN DMSO 2011.12.01
C13DEPT135 DMSO E:\\ shijiangong 23

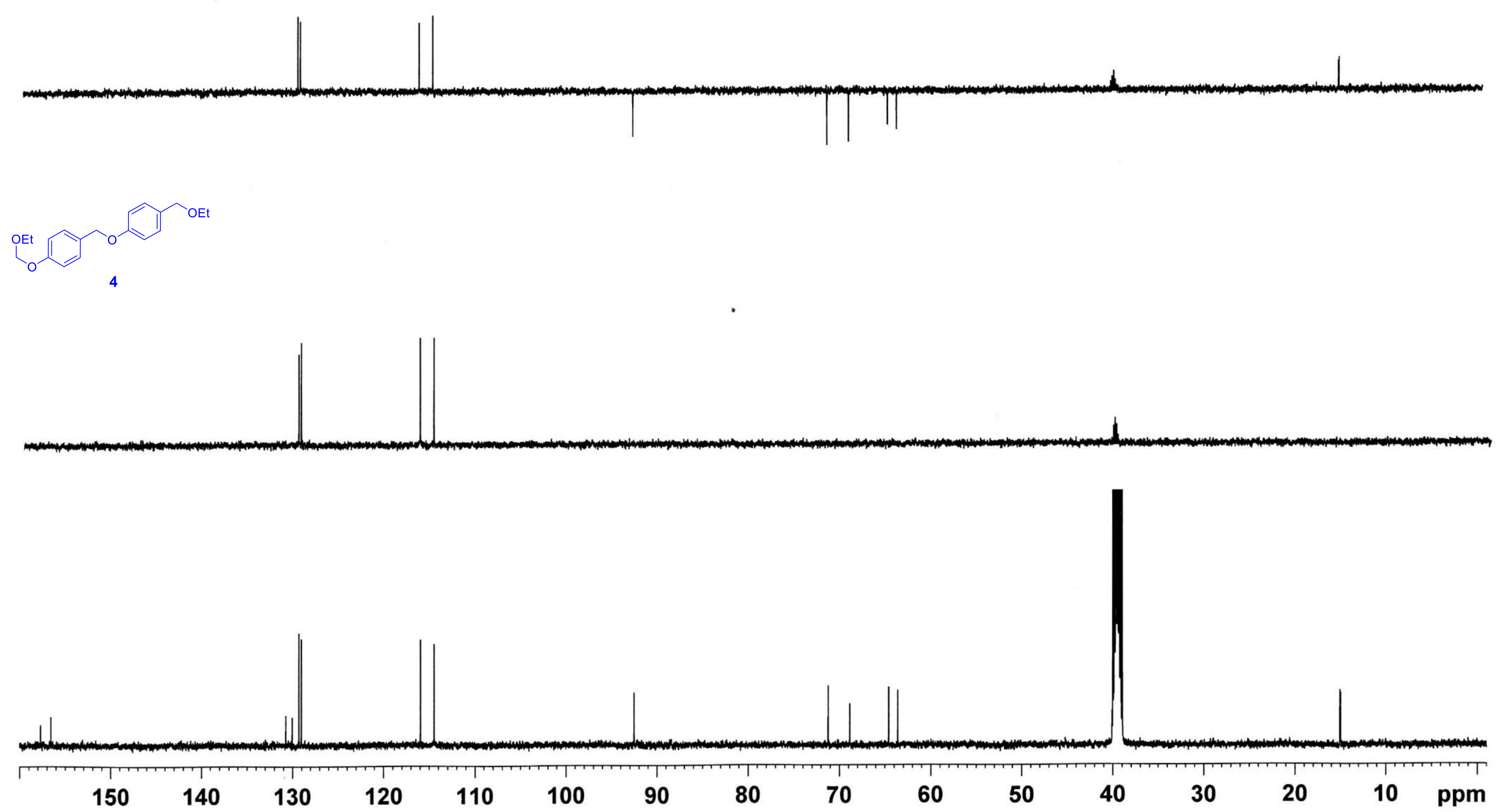

Fig. S45 The DEPT spectrum of compound **4** in DMSO-d_6 (125 MHz).

20120322-WYN-113/2
BRUKER AV-III-500 COSY-NMR WYN-113 IN DMSO 2012.03.22
COSYGPMFSW DMSO E:\\ shijiangong 22

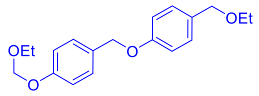

4

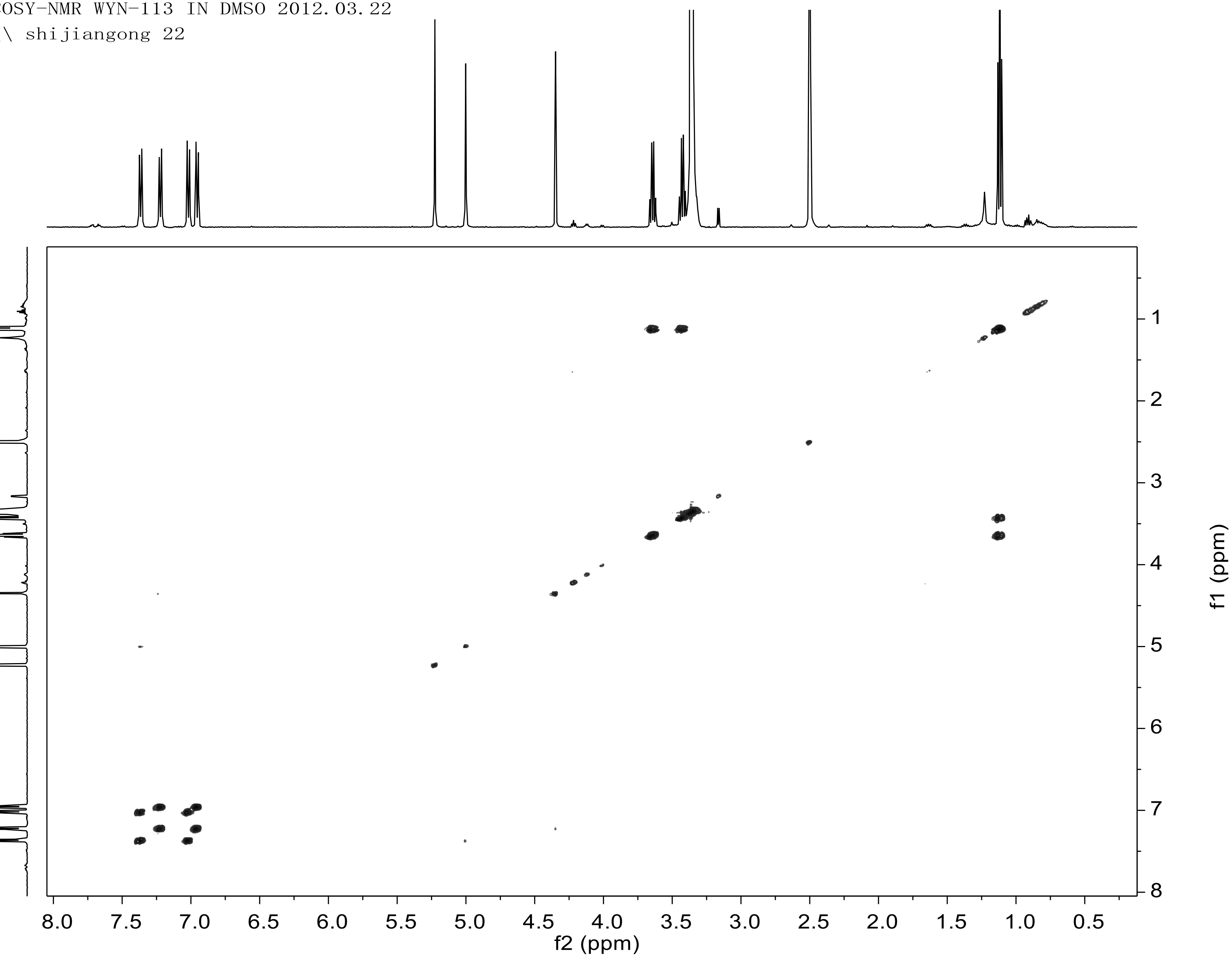

Fig. S46 The ^{1}H-^{1}H COSY spectrum of compound **4** in DMSO-d_6 (500 MHz).

20120322-WYN-113/3
BRUKER AV-III-500 HSQC-NMR WYN-113 IN DMSO 2012.03.22
HSQCETGPSI DMSO E:\\ shijiangong 22

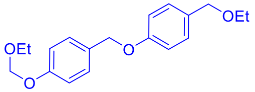

4

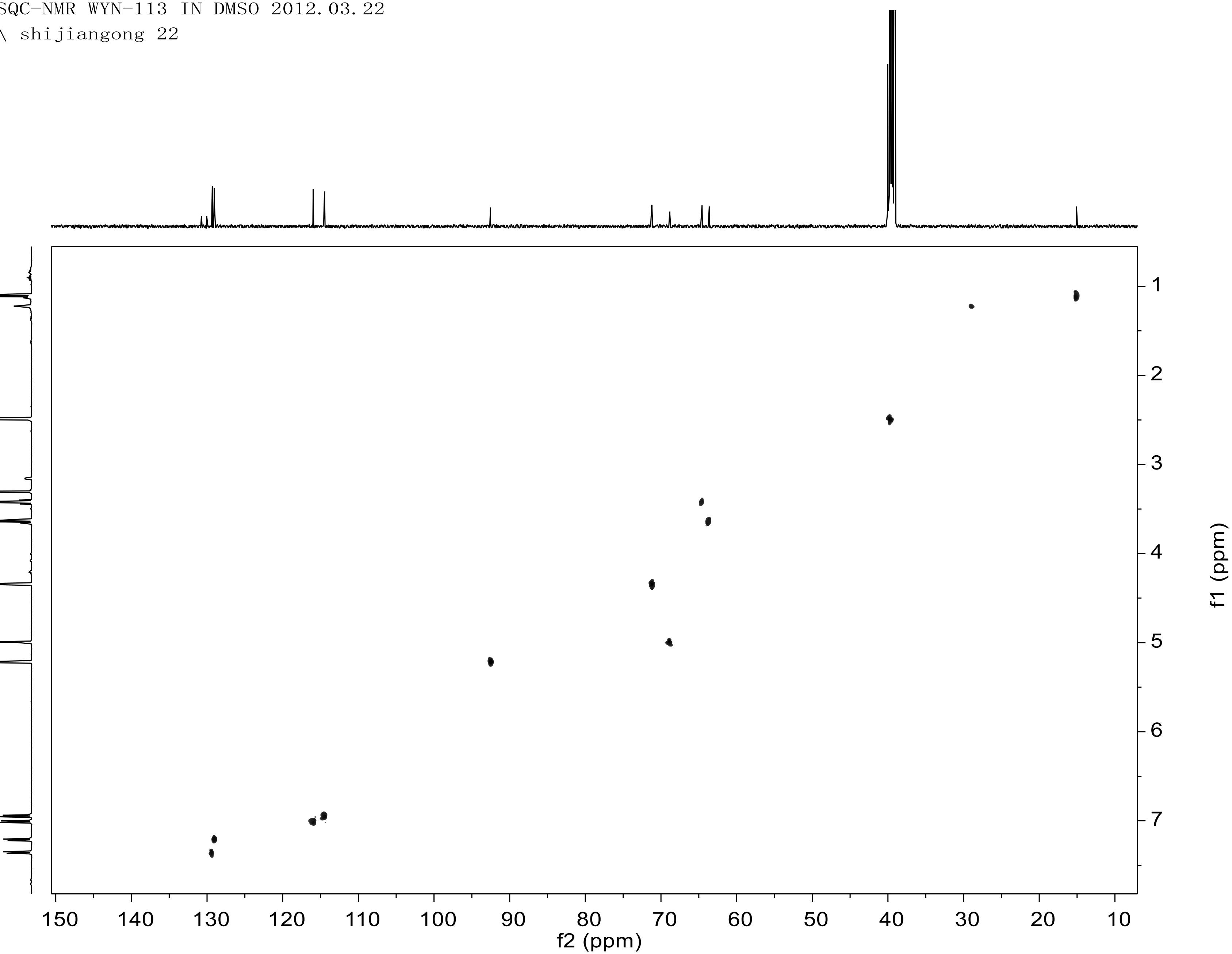

Fig. S47 The HSQC spectrum of compound **4** in DMSO-d_6 (500 MHz for ^{1}H).

20120322-WYN-113/4
BRUKER AV-III-500 HMBC-NMR WYN-113 IN DMSO 2012.03.22
HMBCGPND DMSO E:\\ shijiangong 22

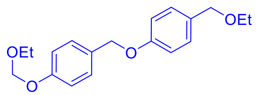

4

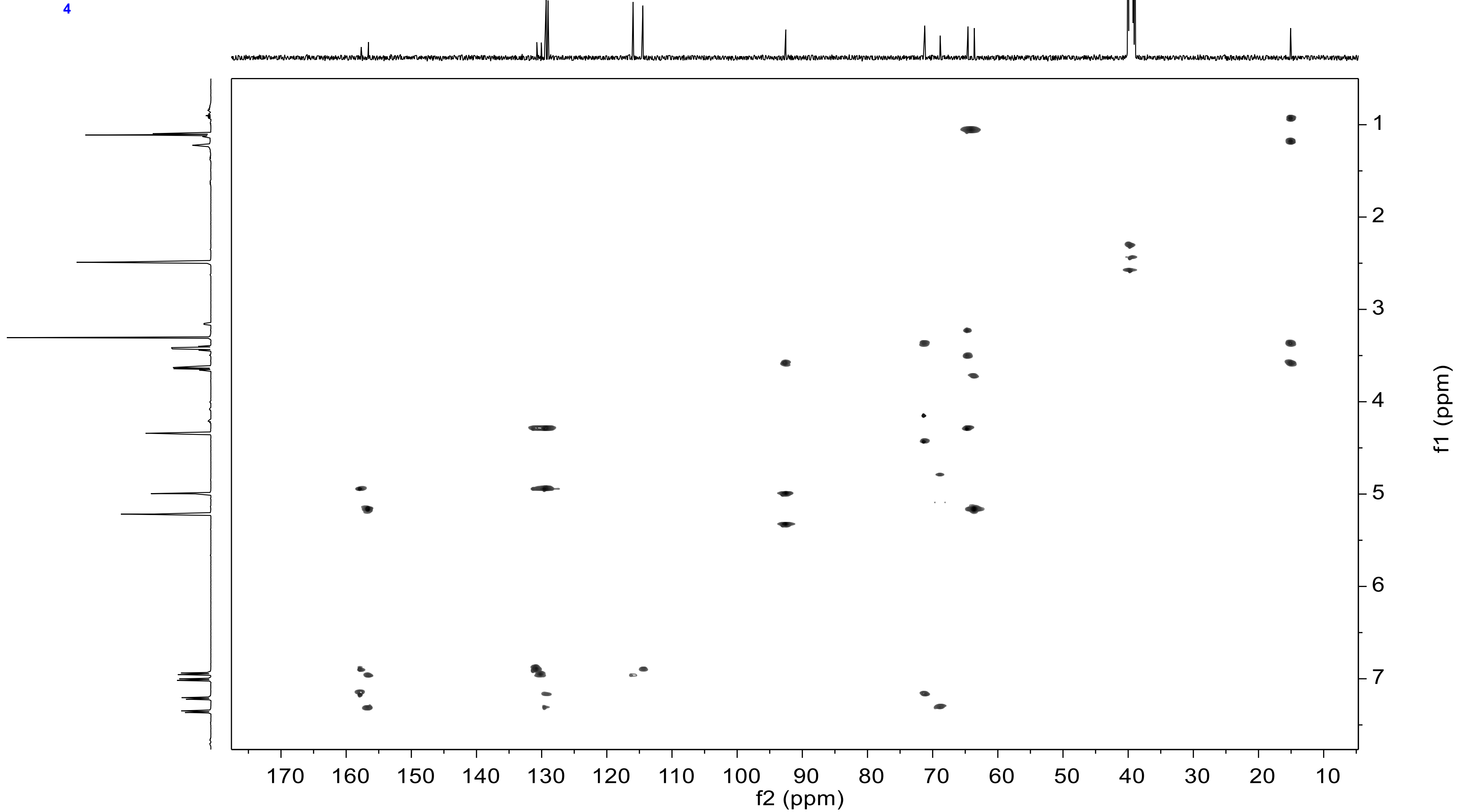

Fig. S48 The HMBC spectrum of compound **4** in DMSO-d_6 (500 MHz for ^{1}H).

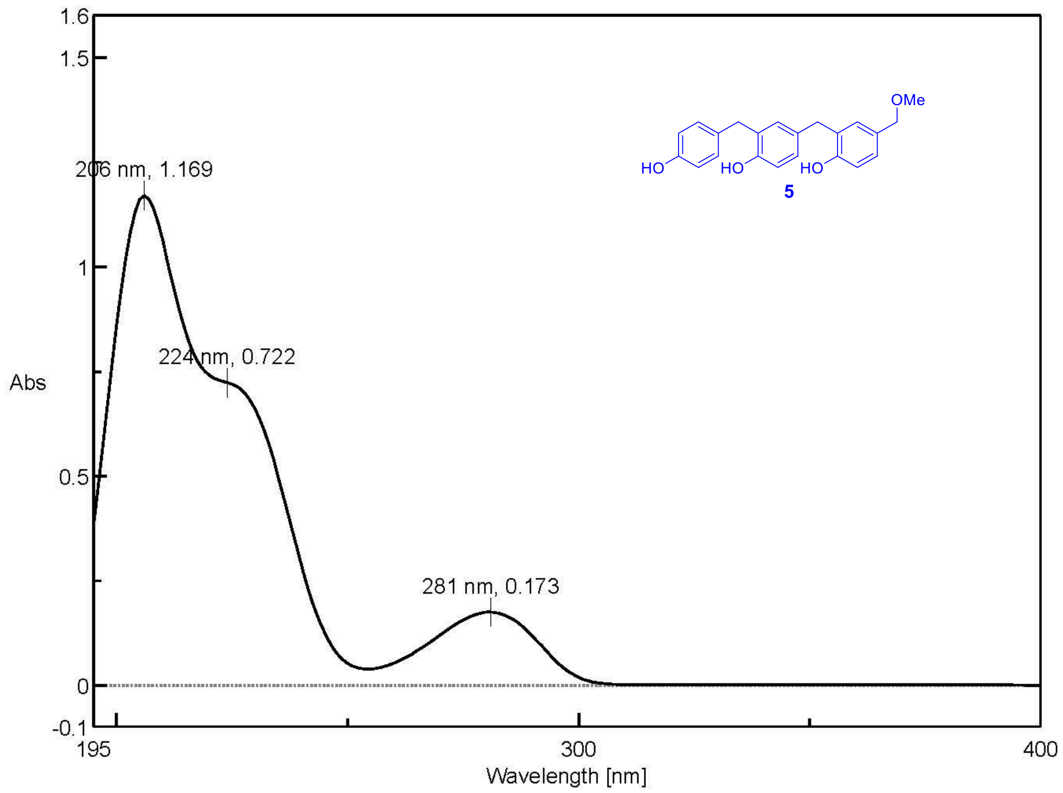

Memory-2

[Comment]

Sample Name	wyn-18C
Comment	
User	wyn
Division	
Company	324

[Measurement Information]

Instrument Name	V-650
Model Name	V-650
Serial No.	A034461150
Accessory	PSC-718
Accessory S/N	A001761114
Position	1
Cell Length	10 mm
Temperature	19.96 C
Control Sonsor	Holder
Monitor Sensor	Holder
Start Mode	Start immediately
Photometric Mode	Abs
Measurement range	400 - 195 nm
Data pitch	1 nm
Band width(UV/Vis)	1.0 nm
Response	Medium
Scanning speed	200 nm/min
Source Change	340 nm
Light Source	D2/WI
Filter Exchange	Step
Correction	Baseline

[Data Information]

Creation Date	2019-12-25 16:21
Data array type	Linear data array
Horizontal	Wavelength [nm]
Vertical	Abs
Start	400 nm
End	195 nm
Data pitch	1 nm
Data points	206

Fig. S49 The UV spectrum of compound **5**.

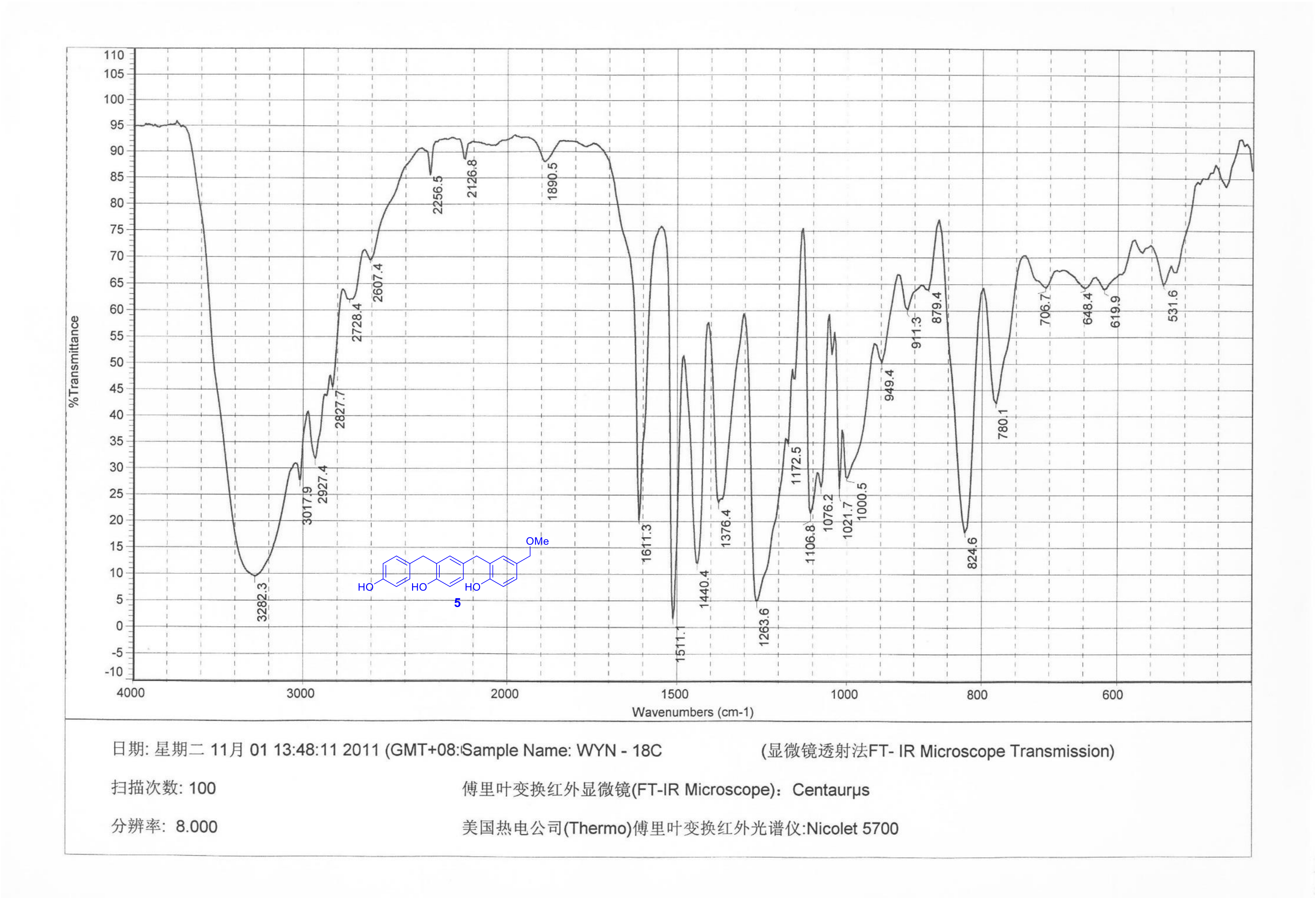

Fig. S50 The IR spectrum of compound **5**.

Single Mass Spectrum Deconvolution Report

Analysis Name: WANGY003.d **Instrument:** LC-MSD-Trap-SL **Print Date:** 7/9/2010 11:13:18 AM
Method: TEST.MS **Operator:** Operator **Acq. Date:** 7/9/2010 11:06:52 AM
Sample Name: WYN-18C
Analysis Info:

Acquisition Parameter:

Mass Range Mode	Std/Normal	Trap Drive	53.0	Scan Begin	100 m/z
Ion Polarity	Positive	Octopole RF Amplitude	171.0 Vpp	Scan End	600 m/z
Ion Source Type	ESI	Capillary Exit	-106.0 Volt	Averages	5 Spectra
Dry Temp (Set)	330 °C	Skimmer	-40.0 Volt	Max. Accu Time	200000 µs
Nebulizer (Set)	15.00 psi	Oct 1 DC	-12.00 Volt	ICC Target	20000
Dry Gas (Set)	5.00 l/min	Oct 2 DC	-1.70 Volt	Charge Control	on

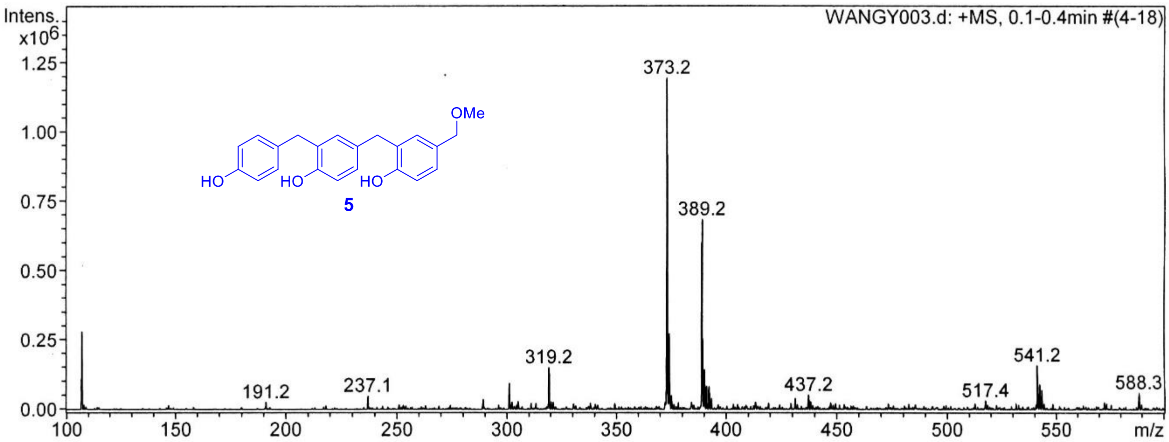

Component	Molecular Mass	Molecule	Absolute Abundance	Relative Abundance

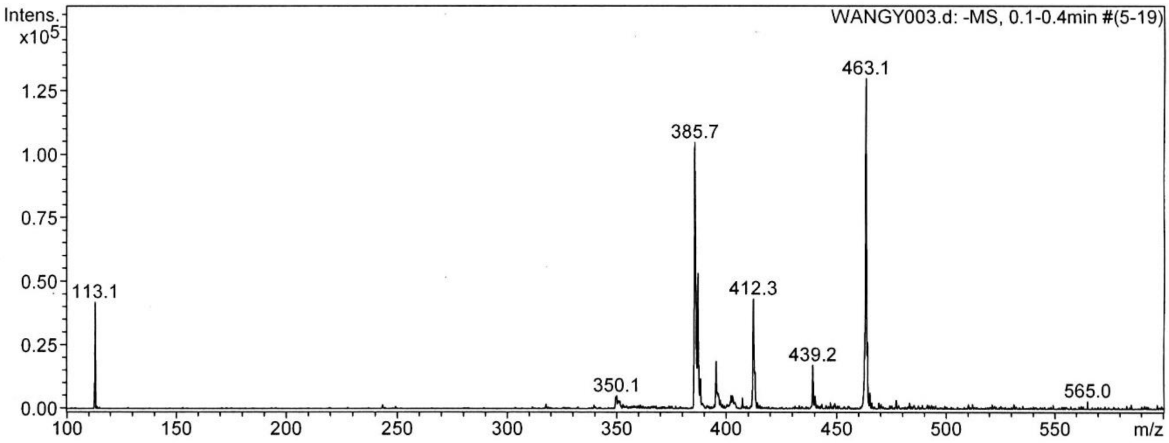

Component	Molecular Mass	Molecule	Absolute Abundance	Relative Abundance

Fig. S51 The ESI-MS of compound **5**.

Qualitative Analysis Report

**Data Filename**	201101175.d	**Sample Name**	WYN-18C
**Sample Type**	Sample	**Position**	P1-C5
**Instrument Name**	Instrument 1	**User Name**	
**Acq Method**		**IRM Calibration Status**	Success
**DA Method**	TEST LCMS.m	**Comment**	

User Chromatograms

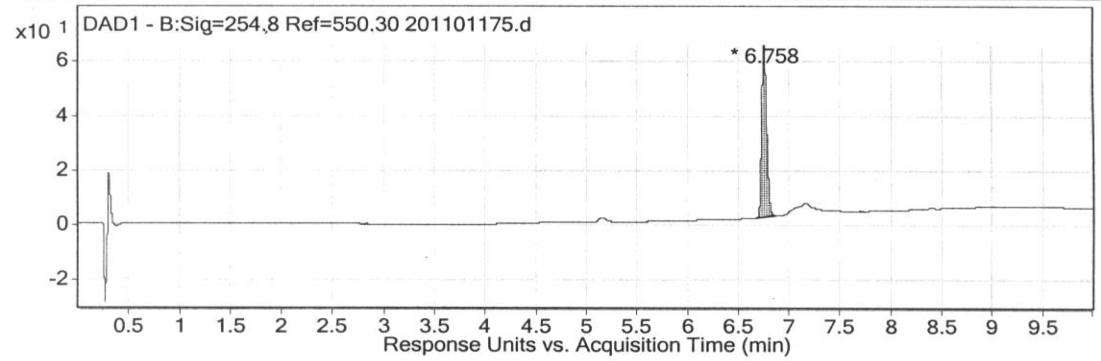

Integration Peak List

Peak	Start	RT	End	Height	Area	Area %
1	6.675	6.758	6.872	62.95	210.8	100

Fragmentor Voltage 135 **Collision Energy** 0 **Ionization Mode** ESI

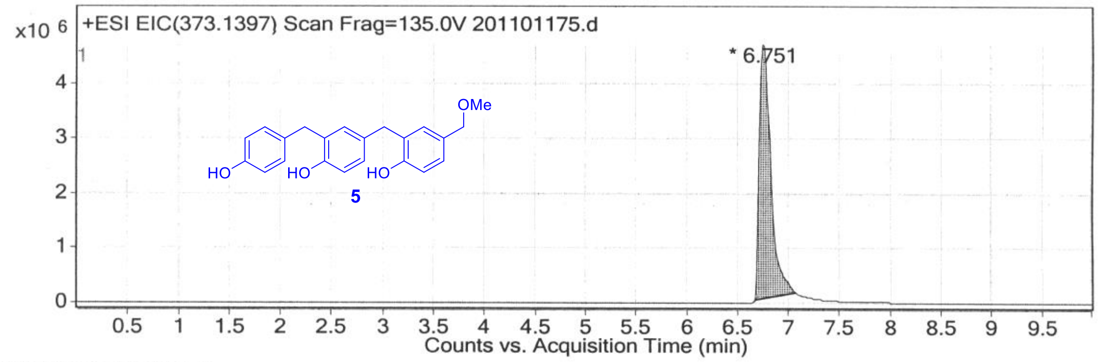

Integration Peak List

Peak	Start	RT	End	Height	Area	Area %
1	6.671	6.751	7.072	4630586	36835314	100

User Spectra

Fragmentor Voltage	Collision Energy	Ionization Mode
135	0	Esi

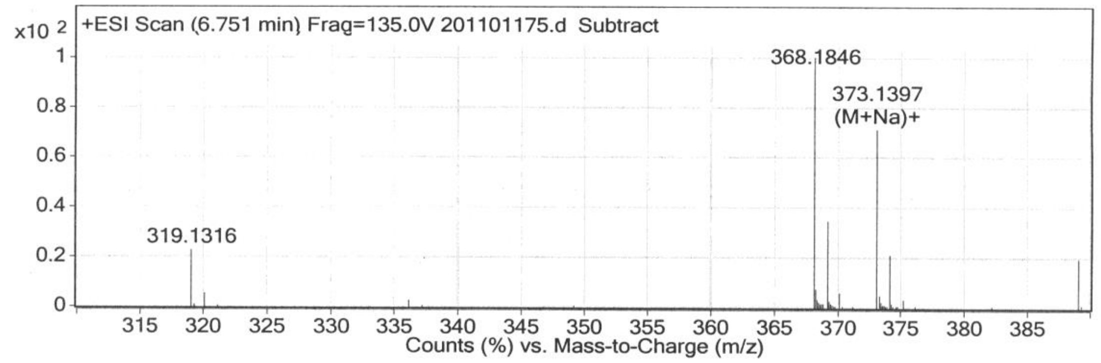

Agilent Technologies Page 1 of 2 Printed at: 10:37 AM on: 1/17/2011

Fig. S52 The (+)-HR-ESI-MS report of compound **5**, page 1.

Peak List

m/z	z	Abund	Formula	Ion
319.1316	1	1505606		
320.1349	1	371354		
368.1846	1	6571919		
368.3441		501385		
369.1872	1	2273060		
370.1905	1	392430		
373.1397	1	4706174	C22 H22 Na O4	(M+Na)+
374.1429	1	1382153	C22 H22 Na O4	(M+Na)+
389.1134		1267413		

Formula Calculator Element Limits

Element	Min	Max
C	3	100
H	0	120
O	0	30
N	0	3
S	0	0
Cl	0	0

Formula Calculator Results

Formula	Best	Mass	Tgt Mass	Diff (ppm)	Ion Species	Score
C22 H22 O4	TRUE	350.1504	350.1518	3.92	C22 H22 Na O4	99.1
C17 H22 N2 O6		350.1504	350.1478	-7.59	C17 H22 N2 Na O6	96.71

--- End Of Report ---

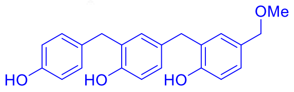

5

Fig. S53 The (+)-HR-ESI-MS report of compound **5**, page 2.

MS Formula Results: + Scan (6.751 min) Sub (201101175.d)

m/z	Ion	Formula	Abundance
373.1397	(M+Na)+	C22 H22 Na O4	4706174

Best	Formula (M)	Ion Formula	Calc m/z	Score	Cross S	Mass	Calc Mass	Diff (ppm)	Abs Diff (ppm)	Abund Match	Spacing Mat	Mass Match	m/z	DBE
☑	C22 H22 O4	C22 H22 Na O4	373.141	99.1		350.1504	350.1518	3.92	3.92	97.59	99.99	99.56	373.1397	12
☐	C17 H22 N2 O6	C17 H22 N2 Na O6	373.137	96.71		350.1504	350.1478	-7.59	7.59	91.22	99.98	98.36	373.1397	8

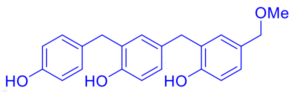

5

page 1

Fig. S54 The (+)-HR-ESI-MS report of compound **5**, page 3.

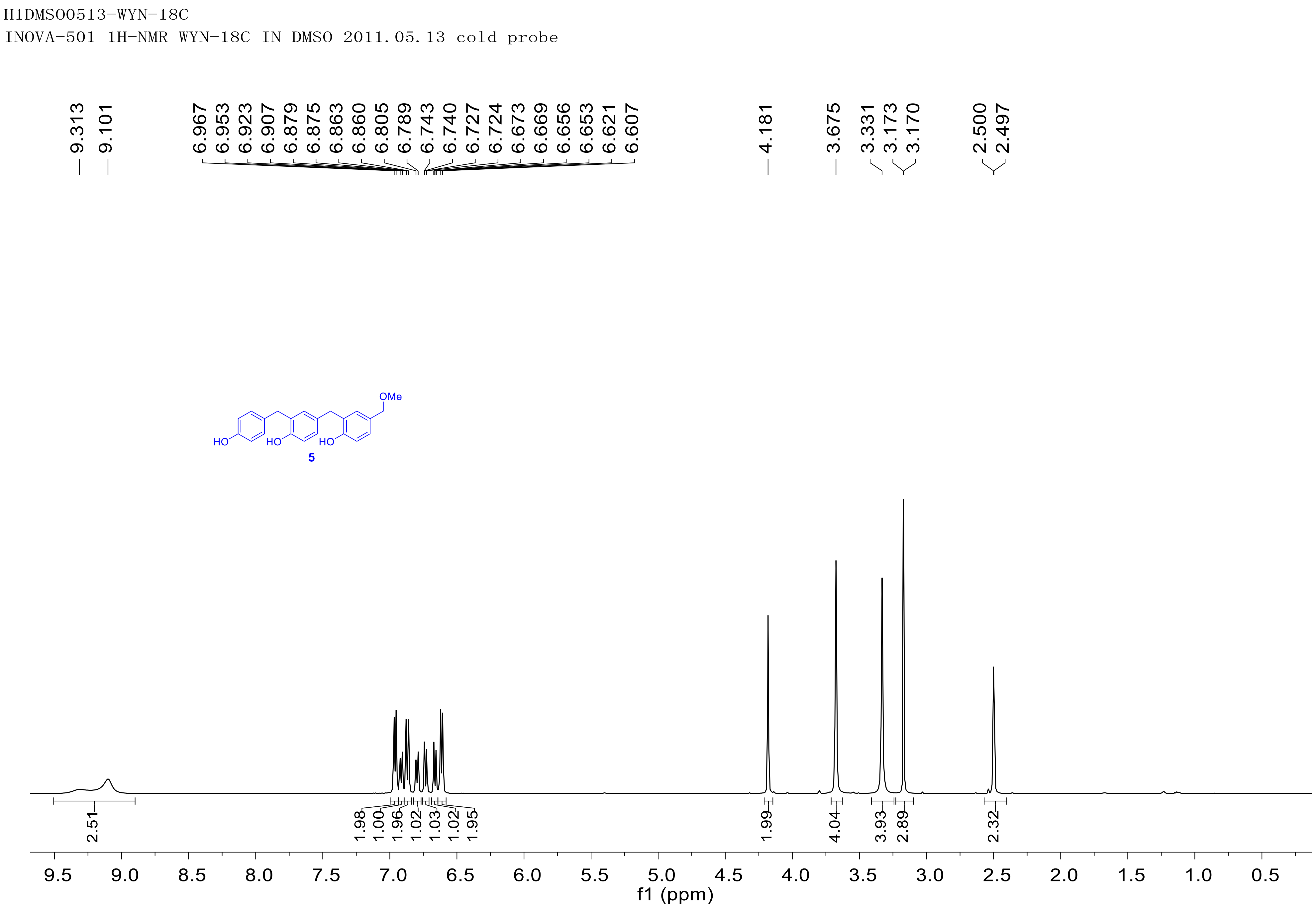

Fig. S55 The ^{1}H NMR spectrum of compound **5** in DMSO-d_6 (500 MHz).

C13DMSO1101-WYN-18C
INOVA-501 13C-NMR WYN-18C IN DMSO 2010.11.01

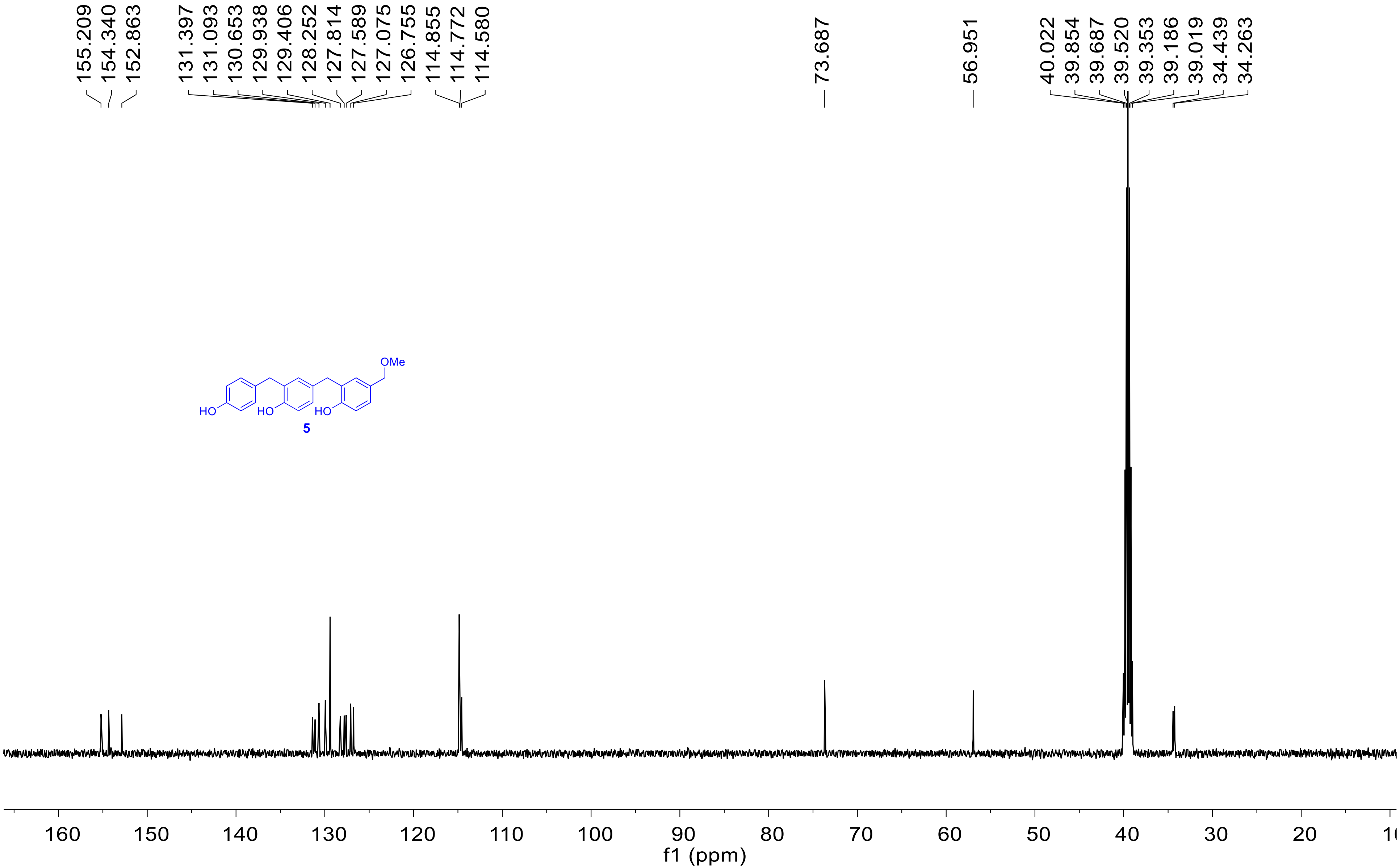

Fig. S56 The ^{13}C NMR spectrum of compound **5** in DMSO-d_6 (125 MHz).

DEPTDMSO0516-WYN-18C
INOVA-501 DEPT WYN-18C IN DMSO 2011.05.16 cold probe

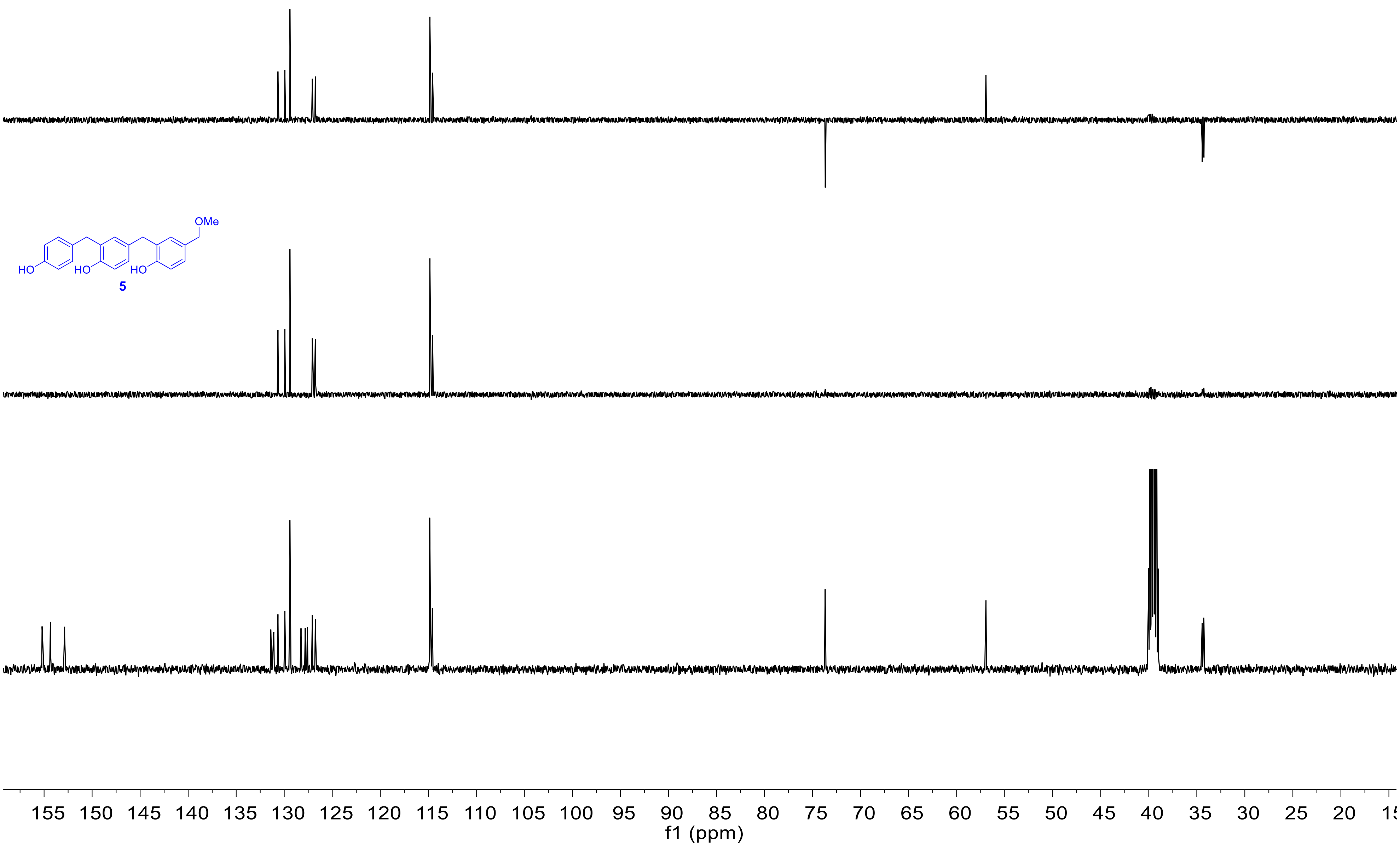

Fig. S57 The DEPT spectrum of compound **5** in DMSO-d_6 (125 MHz).

COSYDMSO0516-WYN-18C
INOVA-501 gCOSY WYN-18C IN DMSO 2011.05.16 cold probe

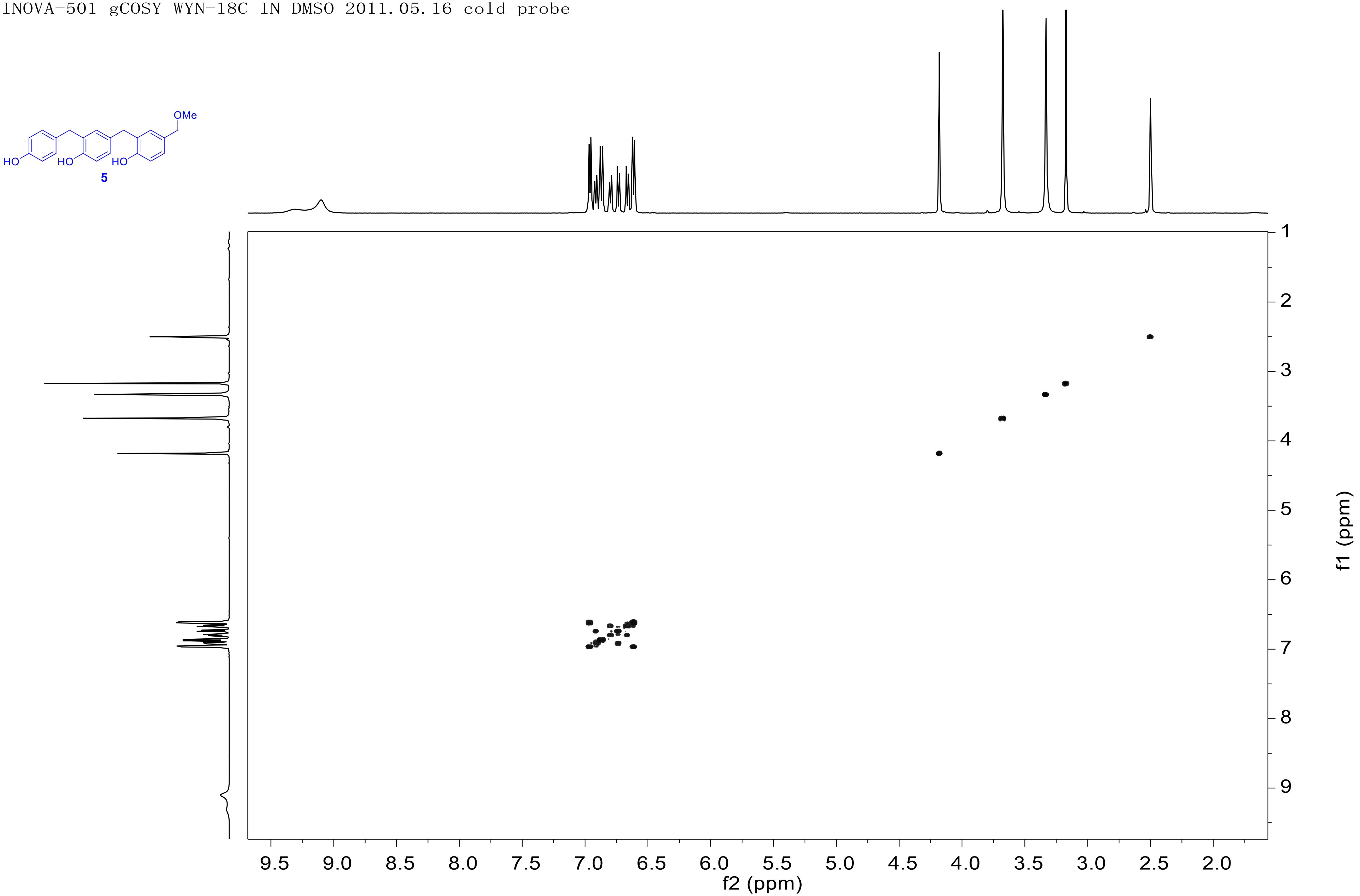

Fig. S58 The ^{1}H-^{1}H COSY spectrum of compound **5** in DMSO-d_6 (500 MHz).

HSQCDMSO0513-WYN-18C
INOVA-501 gHSQC WYN-18C IN DMSO 2011.05.13 cold probe

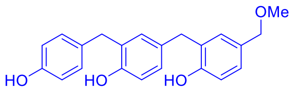

5

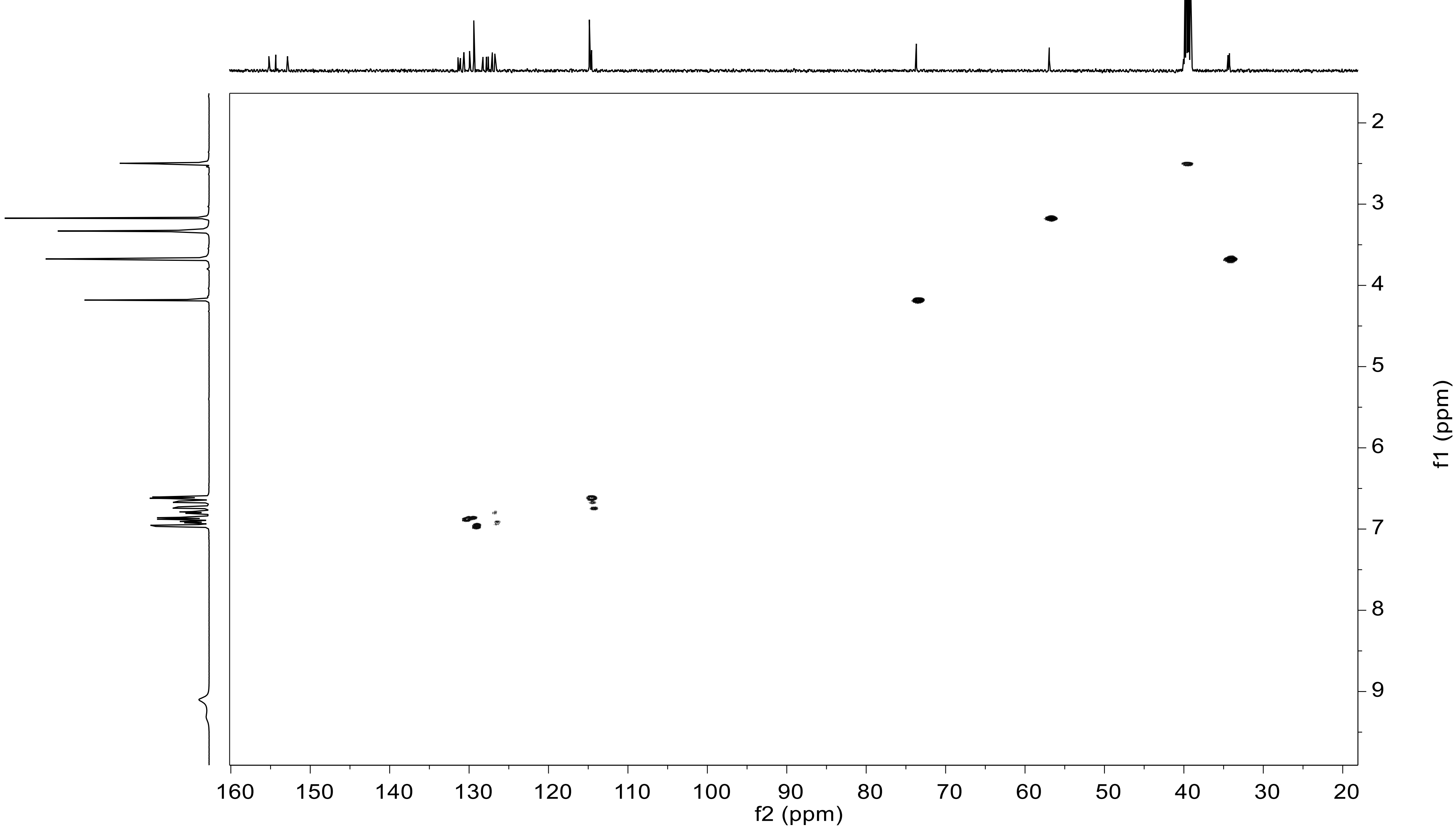

Fig. S59 The HSQC spectrum of compound **5** in DMSO-d_6 (500 MHz for ^{1}H).

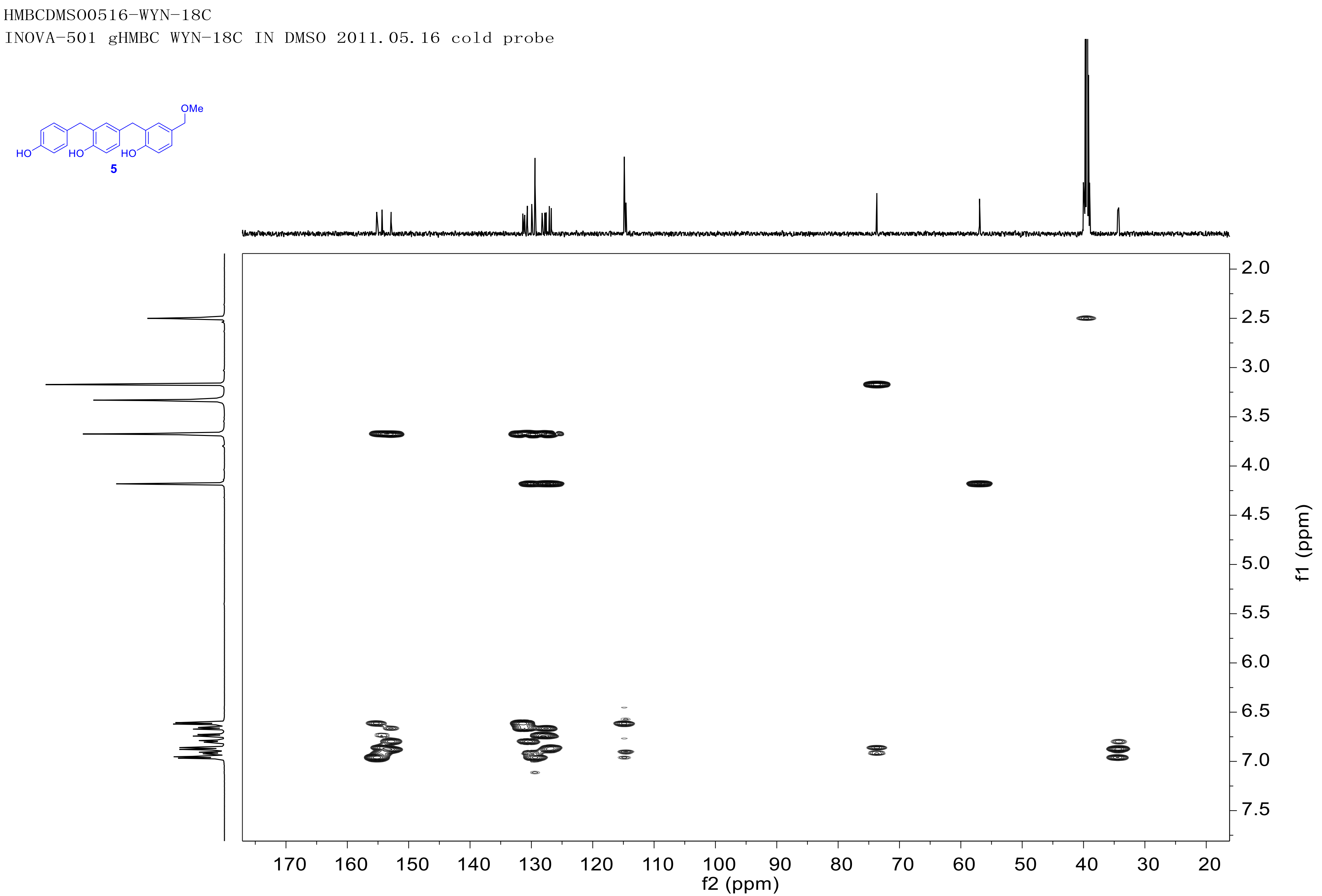

Fig. S60 The HMBC spectrum of compound **5** in DMSO-d_6 (500 MHz for ^{1}H).

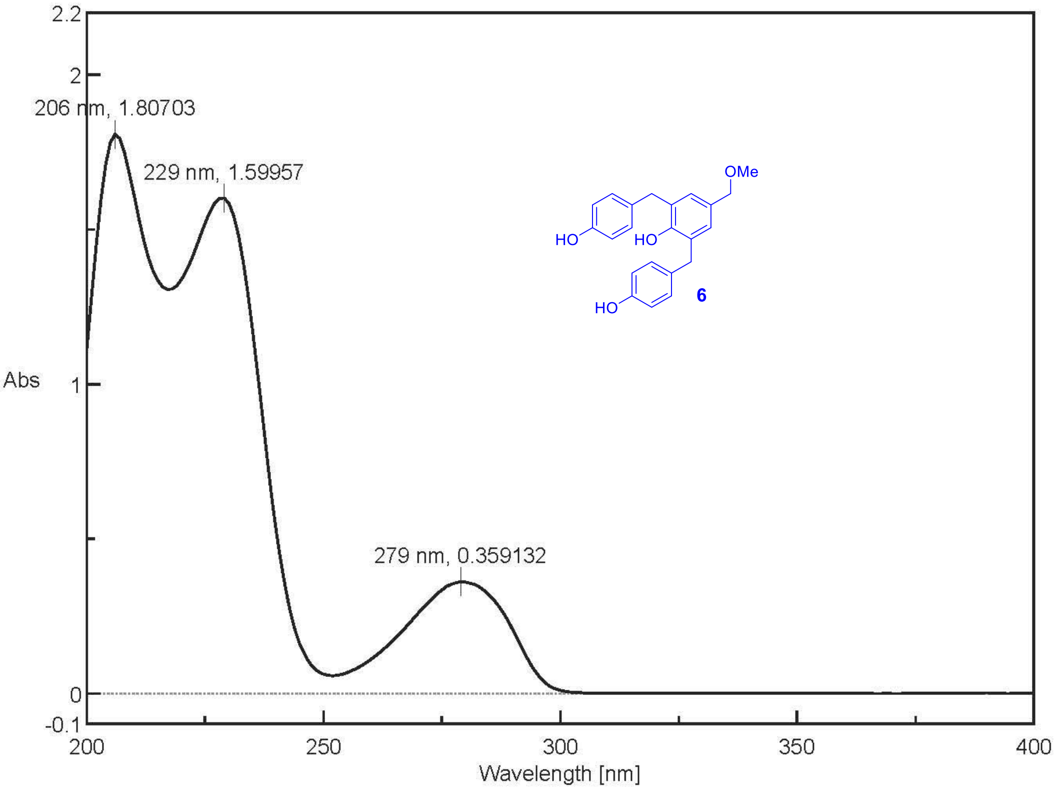

Memory-4

[Comment]
Sample Name wyn-29
Comment
User wyn
Division
Company 324

[Measurement Information]
Instrument Name V-650
Model Name V-650
Serial No. A034461150

Accessory	PSC-718
Accessory S/N	A001761114
Position	1
Cell Length	10 mm
Temperature	19.97 C
Control Sonsor	Holder
Monitor Sensor	Holder
Start Mode	Start immediately

Photometric Mode	Abs
Measurement range	400 - 195 nm
Data pitch	1 nm
Band width(UV/Vis)	1.0 nm
Response	Medium
Scanning speed	200 nm/min
Source Change	340 nm
Light Source	D2/WI
Filter Exchange	Step
Correction	Baseline

[Data Information]

Creation Date	2011-10-12 14:41
Data array type	Linear data array
Horizontal	Wavelength [nm]
Vertical	Abs
Start	400 nm
End	195 nm
Data pitch	1 nm
Data points	206

Fig. S61 The UV spectrum of compound **6**.

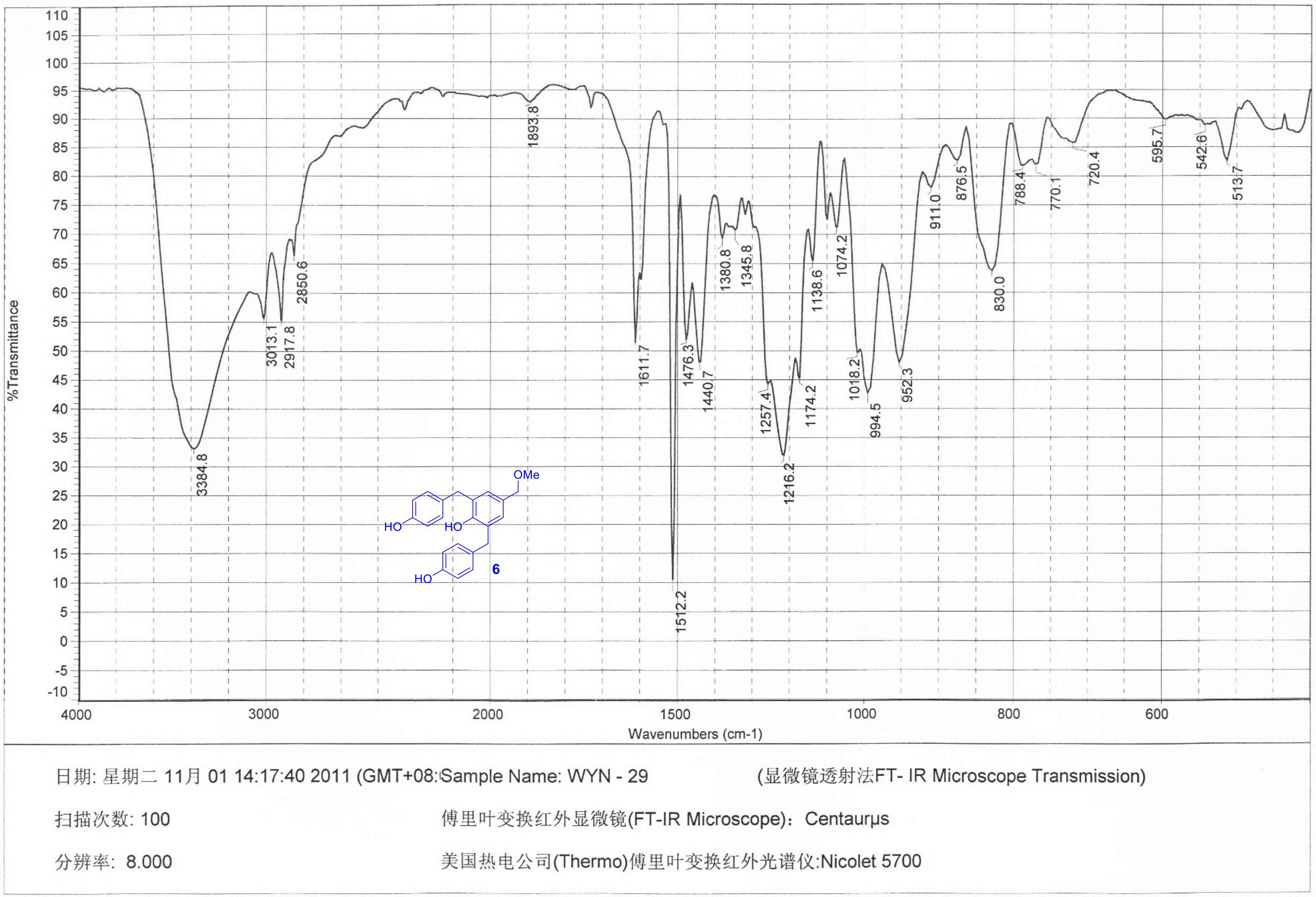

Fig. S62 The IR spectrum of compound **6**.

Single Mass Spectrum Deconvolution Report

Analysis Name: wngyn001.d **Instrument:** LC-MSD-Trap-SL **Print Date:** 6/25/2010 3:42:58 PM
Method: TEST.MS **Operator:** Operator **Acq. Date:** 6/25/2010 3:11:47 PM
Sample Name: WYN-2
Analysis Info:

Acquisition Parameter:

Mass Range Mode	Std/Normal	Trap Drive	45.5	Scan Begin	100 m/z
Ion Polarity	Positive	Octopole RF Amplitude	152.8 Vpp	Scan End	800 m/z
Ion Source Type	ESI	Capillary Exit	-102.3 Volt	Averages	5 Spectra
Dry Temp (Set)	330 °C	Skimmer	-40.0 Volt	Max. Accu Time	200000 µs
Nebulizer (Set)	15.00 psi	Oct 1 DC	-12.00 Volt	ICC Target	20000
Dry Gas (Set)	5.00 l/min	Oct 2 DC	-1.70 Volt	Charge Control	on

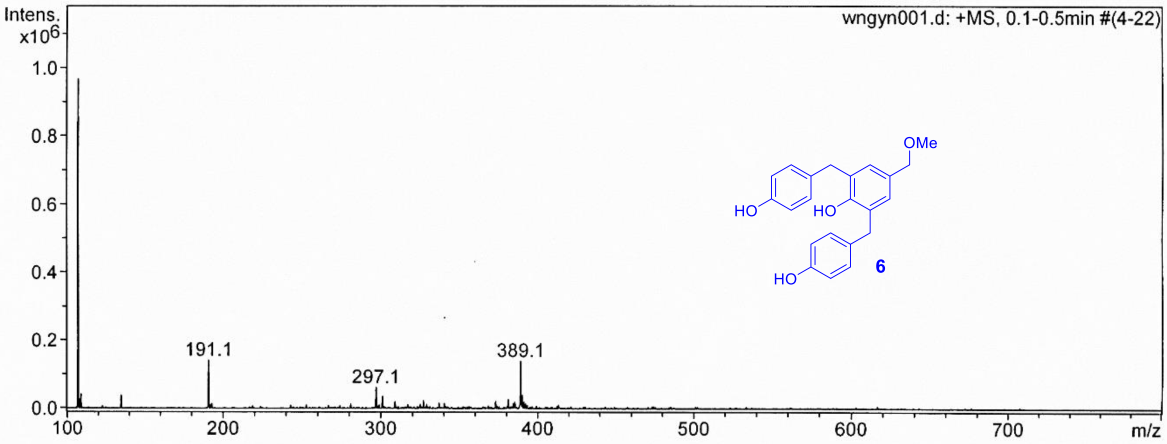

Component	Molecular Mass	Molecule	Absolute Abundance	Relative Abundance

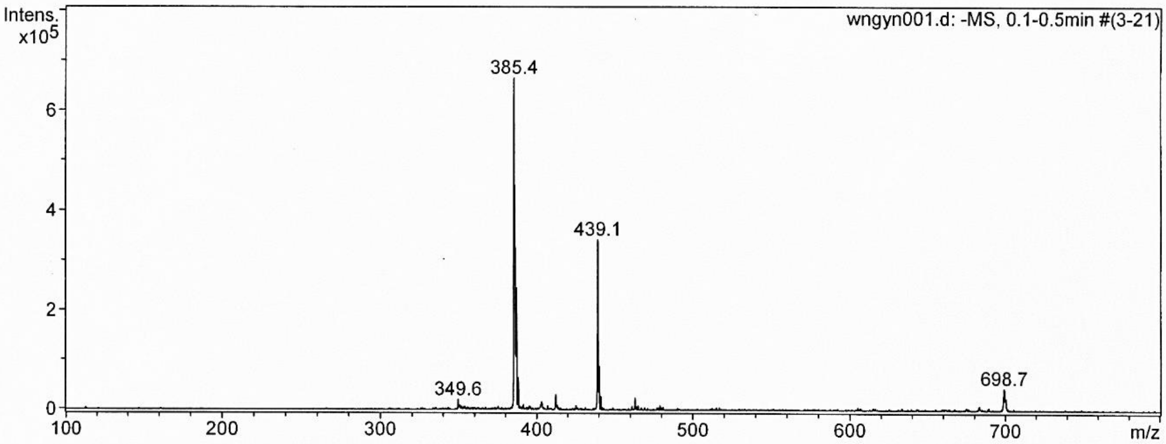

Component	Molecular Mass	Molecule	Absolute Abundance	Relative Abundance

MSD Trap Report v 4 (A4-Opt2) Page 1 of 1 Agilent Technologies

Fig. S63 The ESI-MS of compound **6**.

Qualitative Analysis Report

**Data Filename**	201105052.d	**Sample Name**	WYN-29
**Sample Type**	Sample	**Position**	P1-B2
**Instrument Name**	Instrument 1	**User Name**	
**Acq Method**		**IRM Calibration Status**	Success
**DA Method**	TEST LCMS.m	**Comment**	

User Chromatograms

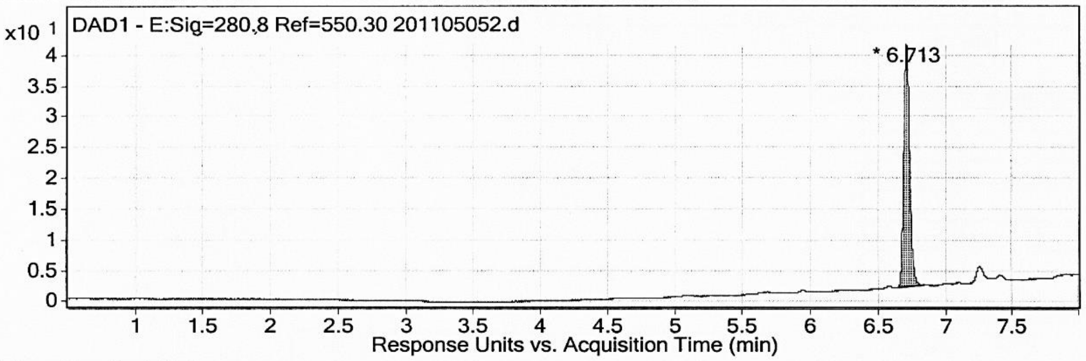

Integration Peak List

Peak	Start	RT	End	Height	Area	Area %
1	6.644	6.713	6.845	39.28	121.88	100

Fragmentor Voltage 135 **Collision Energy** 0 **Ionization Mode** ESI

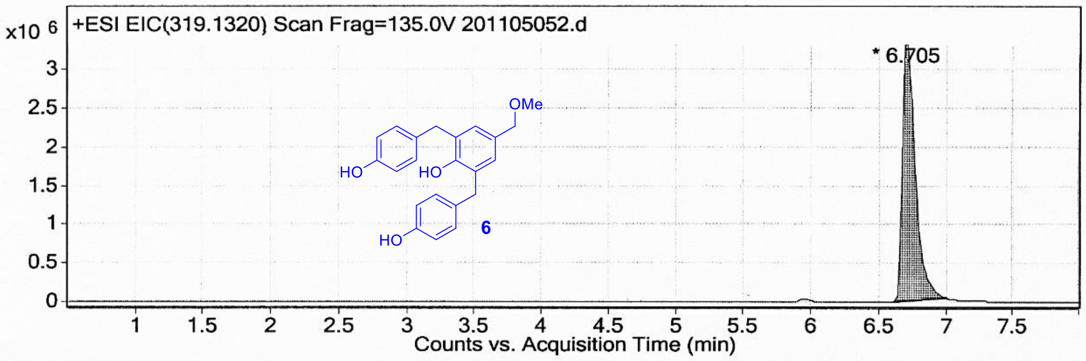

Integration Peak List

Peak	Start	RT	End	Height	Area	Area %
1	6.608	6.705	7.01	3300227	22759221	100

User Spectra

Fragmentor Voltage	Collision Energy	Ionization Mode
135	0	Esi

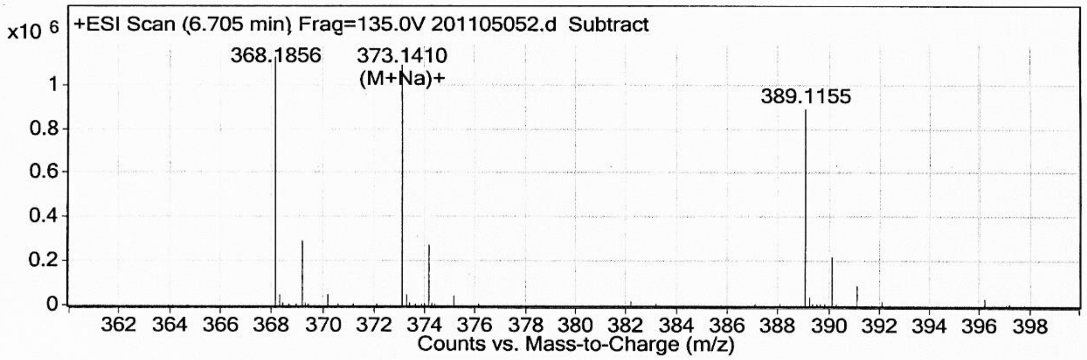

Fig. S64 The (+)-HR-ESI-MS report of compound **6**, page 1.

Qualitative Analysis Report

Peak List

m/z	z	Abund	Formula	Ion
319.1325	1	3311627		
319.2871		205209		
320.1366	1	853813		
368.1856	1	1125284		
369.1892	1	295013		
373.141	1	1091166	C22 H22 Na O4	(M+Na)+
374.1446	1	274193	C22 H22 Na O4	(M+Na)+
389.1155	1	888139		
390.1182	1	220836		

Formula Calculator Element Limits

Element	Min	Max
C	3	100
H	0	120
O	0	30
N	0	2
S	0	0
Cl	0	0

Formula Calculator Results

Formula	Best	Mass	Tgt Mass	Diff (ppm)	Ion Species	Score
C22 H22 O4	TRUE	350.1518	350.1518	-0.06	C22 H22 Na O4	99.96

--- End Of Report ---

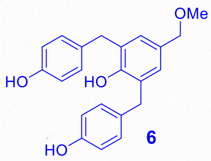

6

Fig. S65 The (+)-HR-ESI-MS report of compound **6**, page 2.

MS Formula Results: + Scan (6.705 min) Sub (201105052.d)

m/z	Ion	Formula	Abundance
373.141	(M+Na)+	C22 H22 Na O4	1091166.4

Best	Formula (M)	Ion Formula	Calc m/z	Score	Cross S	Mass	Calc Mass	Diff (ppm)	Abs Diff (ppm)	Abund Match	Spacing Mat	Mass Match	m/z	DBE
☑	C22 H22 O4	C22 H22 Na O4	373.141	99.96		350.1518	350.1518	-0.06	0.06	99.87	99.99	100	373.141	12

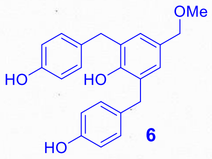

6

page 1

Fig. S66 The (+)-HR-ESI-MS report of compound **6**, page 3.

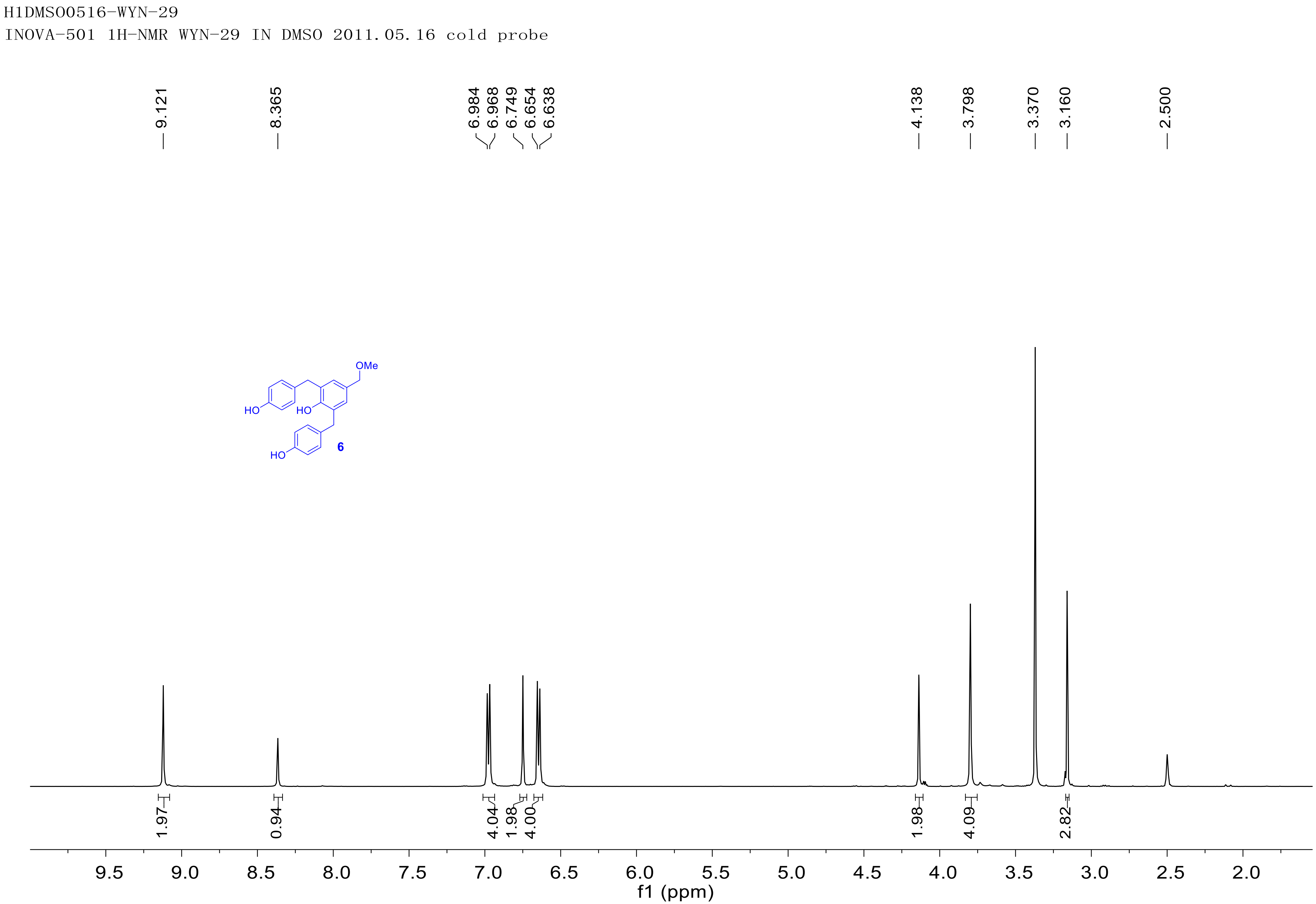

Fig. S67 The ^{1}H NMR spectrum of compound **6** in DMSO-d_6 (500 MHz).

C13DMSO1101-WYN-29
INOVA-501 13C-NMR WYN-29 IN DMSO 2010.11.01

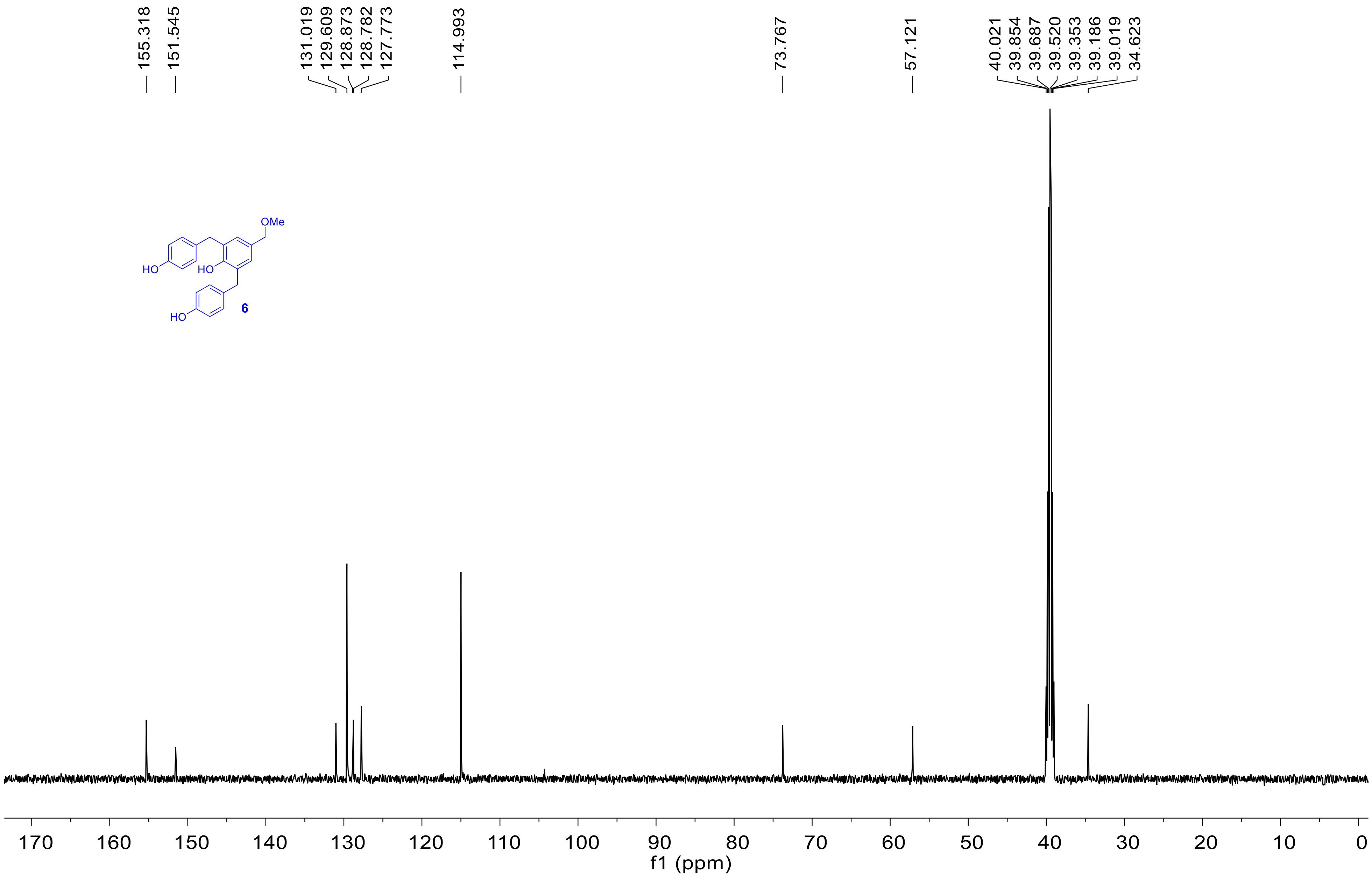

Fig. S68 The ^{13}C NMR spectrum of compound **6** in DMSO-d_6 (125 MHz).

DEPTDMSO0516-WYN-29
INOVA-501 DEPT WYN-29 IN DMSO 2011.05.16 cold probe

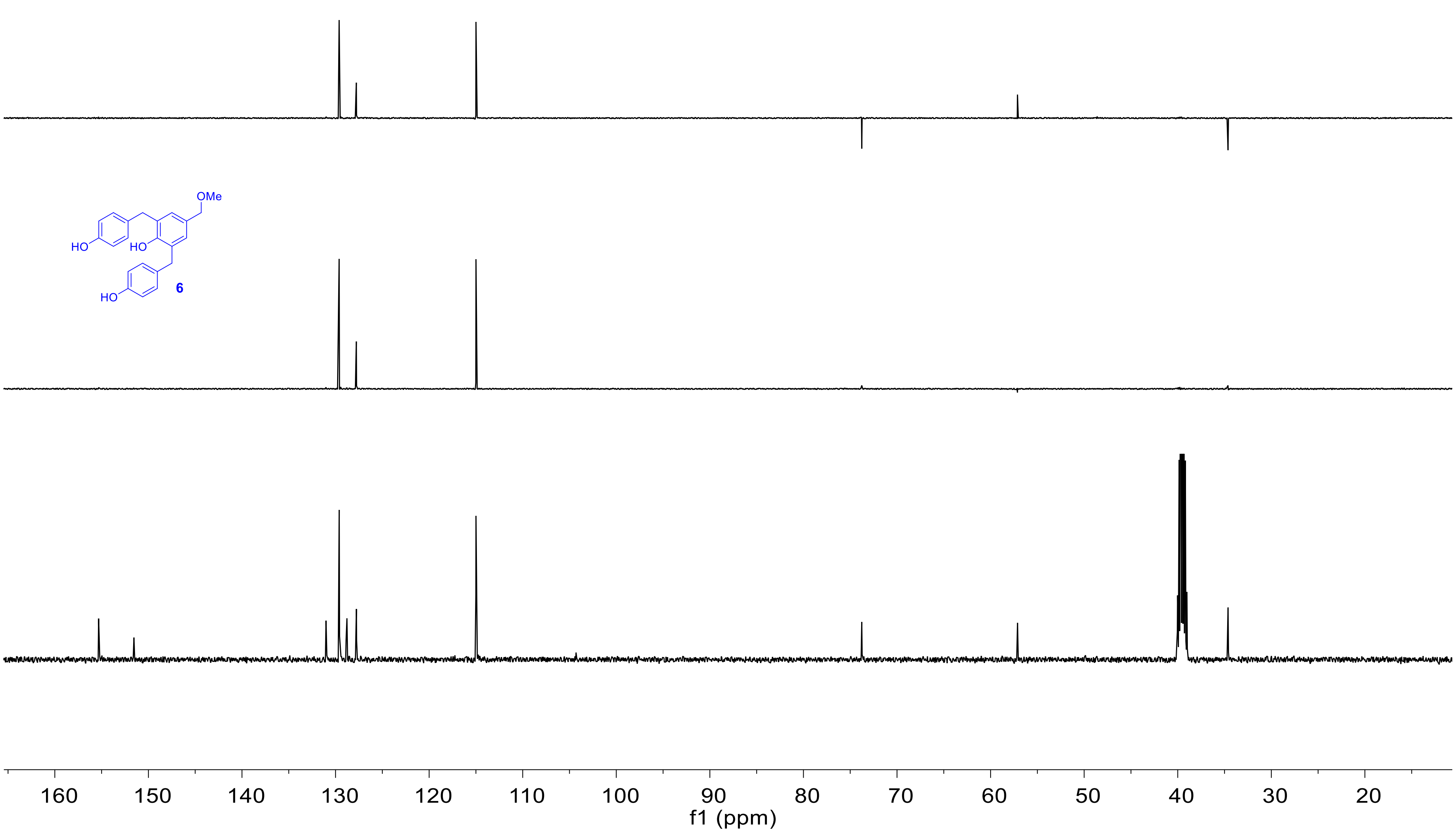

Fig. S69 The DEPT spectrum of compound **6** in DMSO-d_6 (125 MHz).

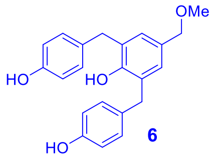

6

COSYDMSO0516-WYN-29
INOVA-501 gCOSY WYN-29 IN DMSO 2011.05.16 cold probe

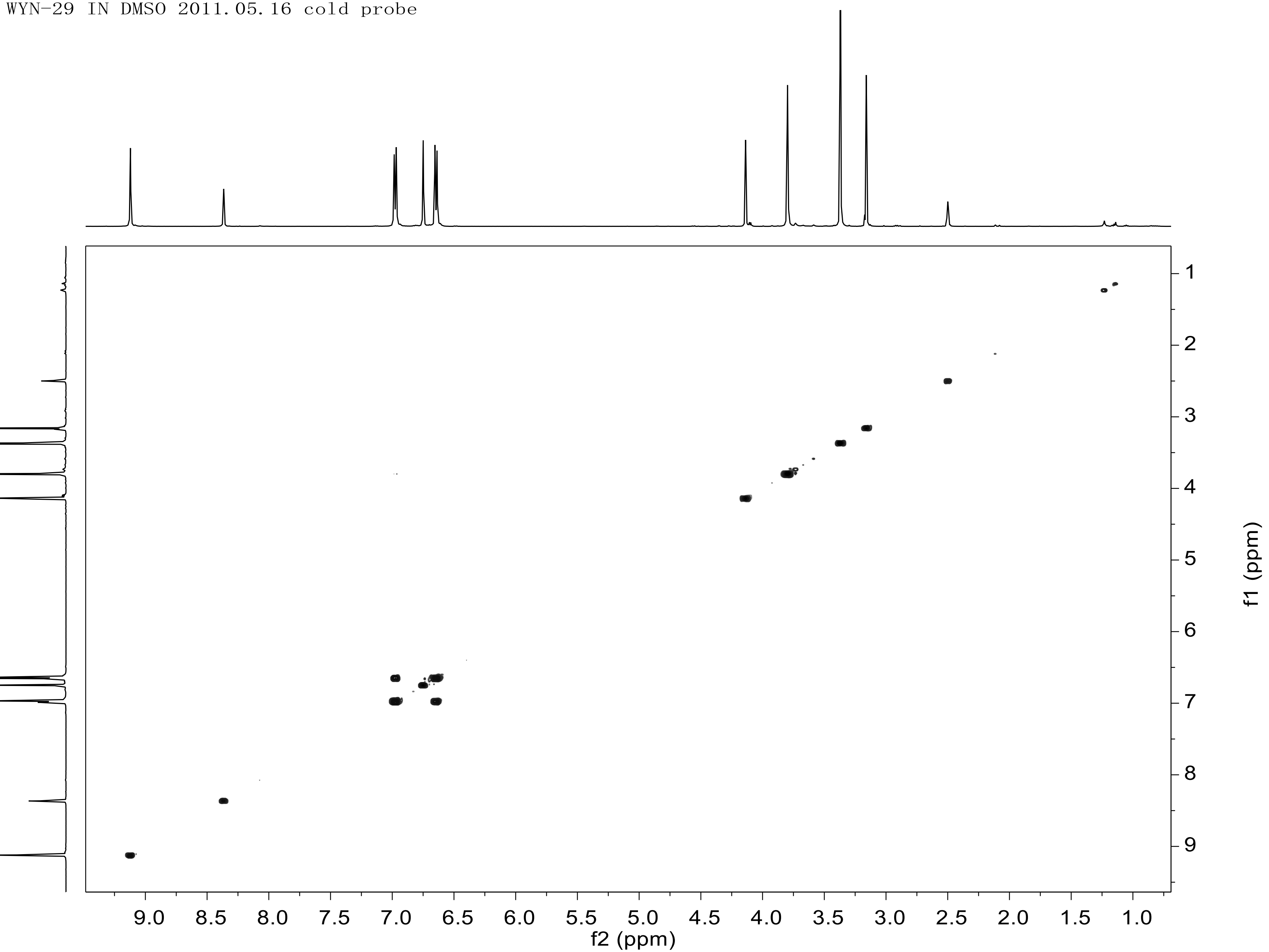

Fig. S70 The ^{1}H-^{1}H COSY spectrum of compound **6** in DMSO-d_6 (500 MHz).

HSQCDMSO0513-WYN-29
INOVA-501 gHSQC WYN-29 IN DMSO 2011.05.13 cold probe

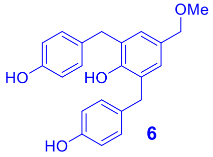

6

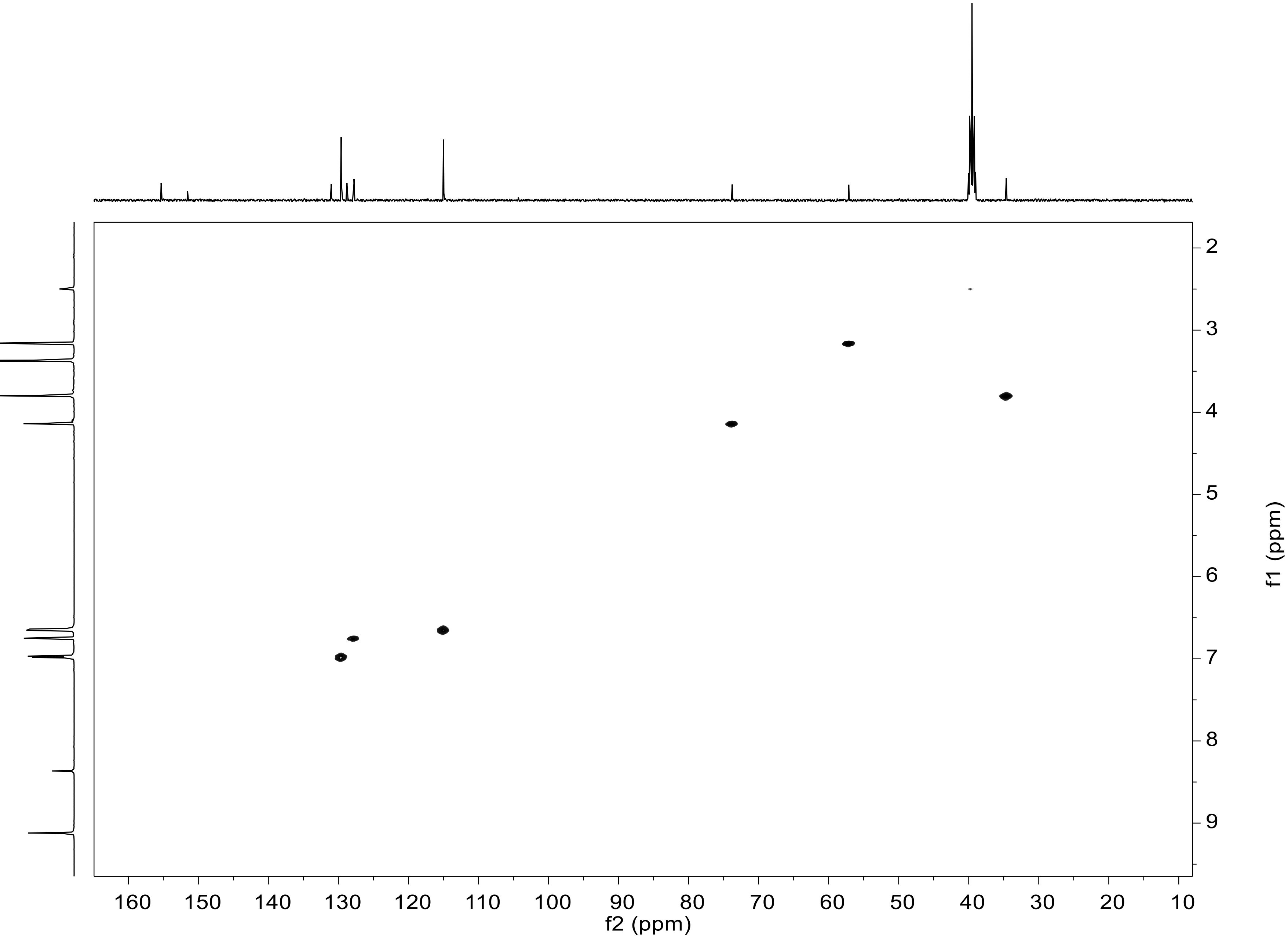

Fig. S71 The HSQC spectrum of compound **6** in DMSO-d_6 (500 MHz for ^{1}H).

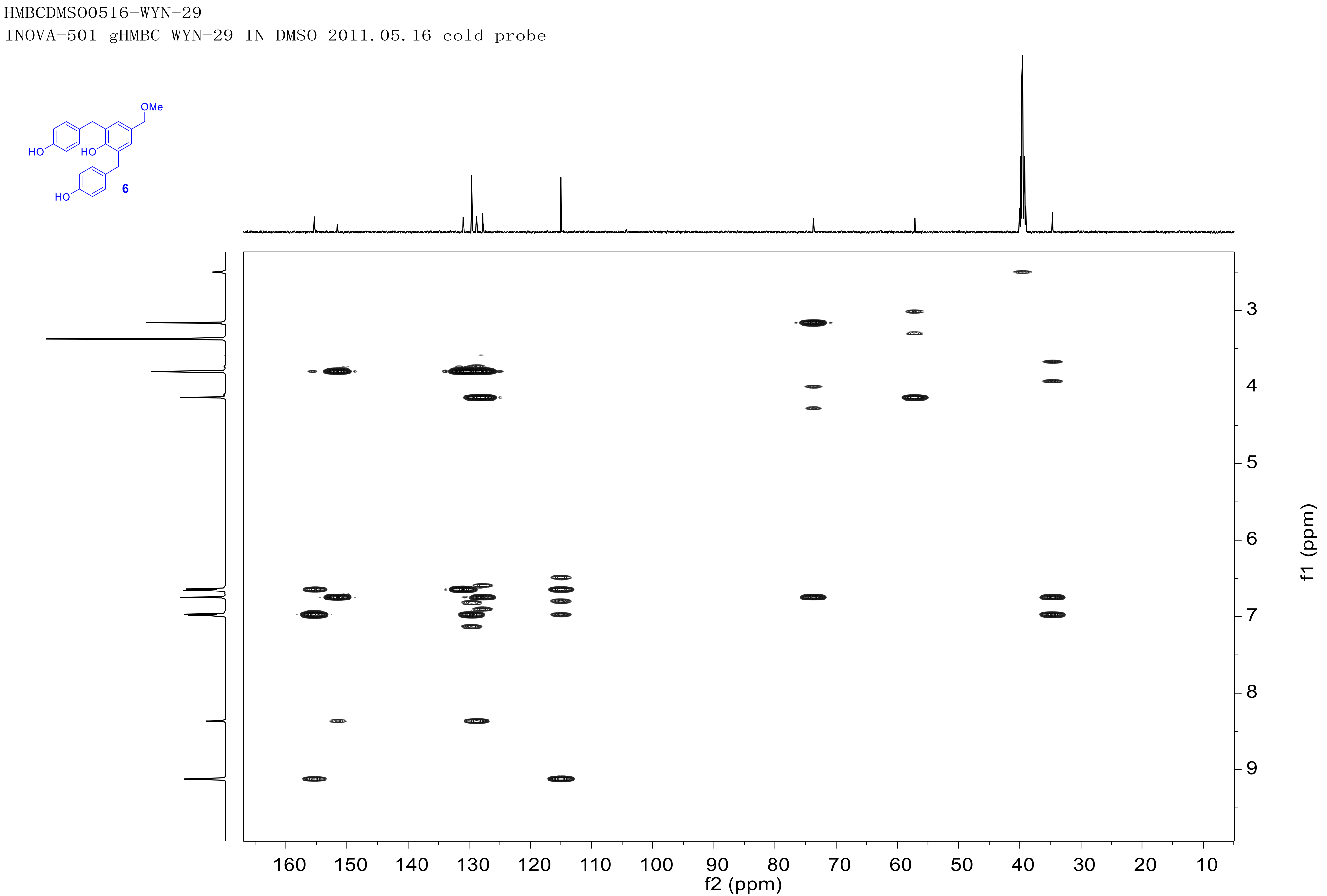

Fig. S72 The HMBC spectrum of compound **6** in DMSO-d_6 (500 MHz for ^{1}H).

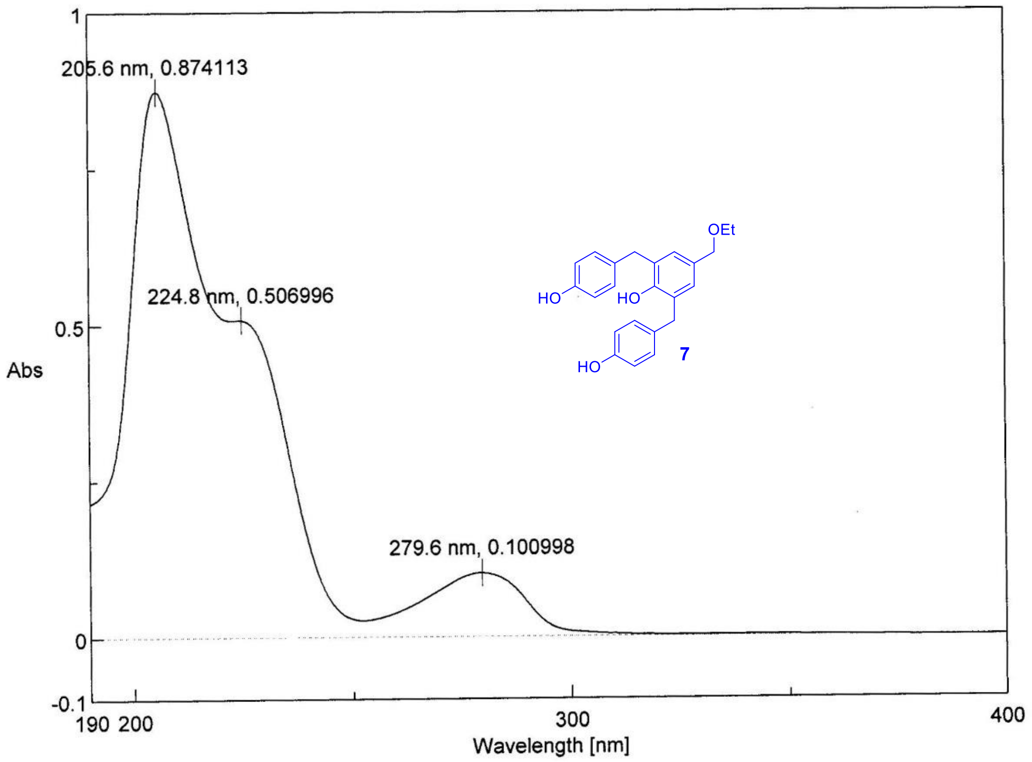

Memory-7

[Comment]
Sample Name TMG-26B
Comment 0.02
User
Division UV
Company 324

[Measurement Information]
Instrument Name V-650
Model Name V-650
Serial No. A034461150

Accessory PSC-718
Accessory S/N A001761114
Position 1
Cell Length 10 mm
Temperature 19.97 C
Control Sonsor Holder
Monitor Sensor Holder
Start Mode Start immediately

Photometric Mode Abs
Measurement range 400 - 190 nm
Data pitch 0.2 nm
Band width(UV/Vis) 2.0 nm
Response Medium
Scanning speed 200 nm/min
Source Change 340 nm
Light Source D2/WI
Filter Exchange Step
Correction Baseline

[Data Information]
Creation Date 2014-2-26 9:25

Data array type Linear data array
Horizontal Wavelength [nm]
Vertical Abs
Start 400 nm
End 190 nm
Data pitch 0.2 nm
Data points 1051

Fig. S73 The UV spectrum of compound **7**.

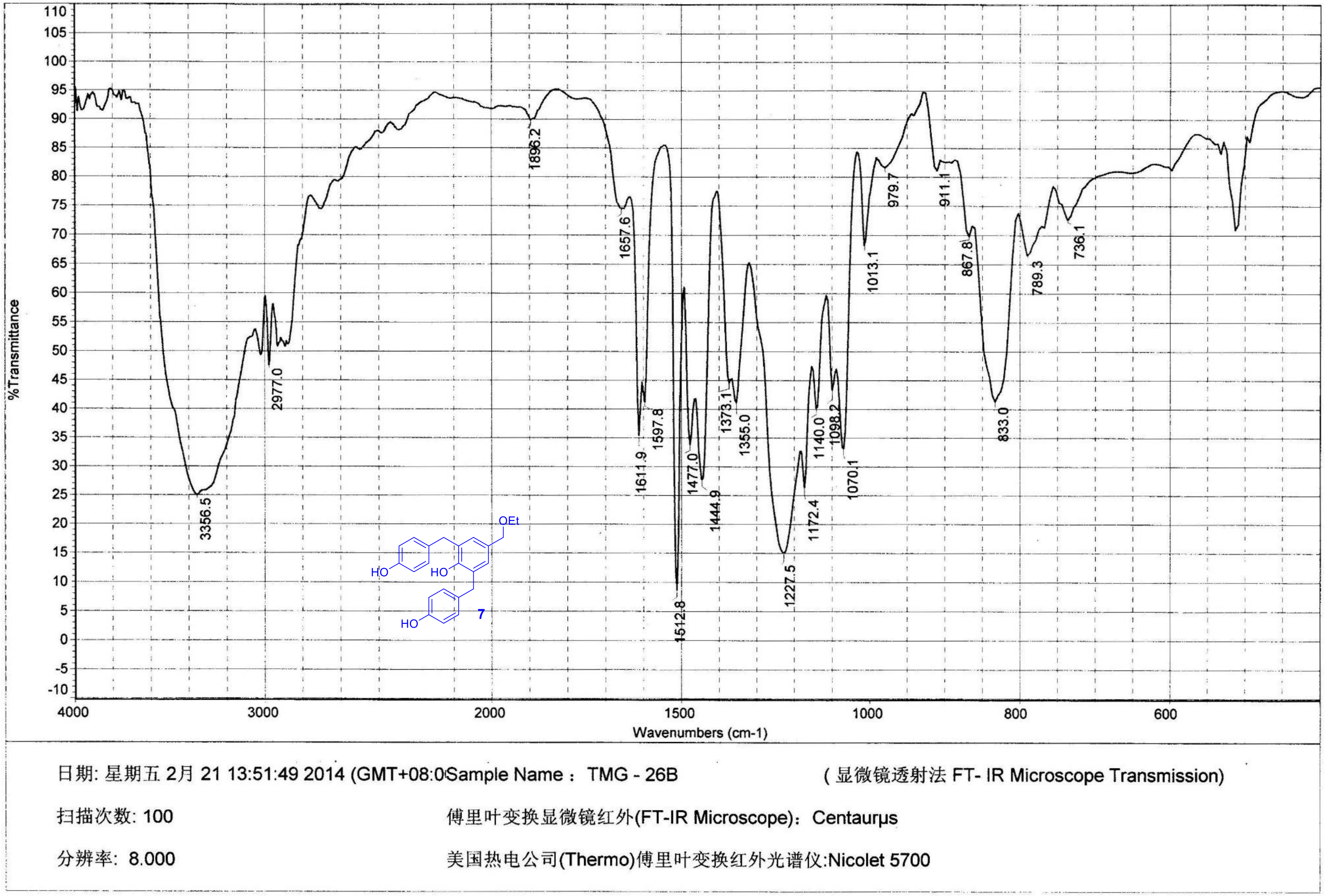

Fig. S74 The IR spectrum of compound **7**.

Single Mass Spectrum Deconvolution Report

**Analysis Name:**	guoql042.d	**Instrument:**	LC-MSD-Trap-SL	**Print Date:**	7/20/2011 9:54:18 AM
**Method:**	TEST.MS	**Operator:**	Operator	**Acq. Date:**	7/20/2011 9:48:35 AM
**Sample Name:**	TMG-26B				
**Analysis Info:**					

Acquisition Parameter:

Mass Range Mode	Std/Normal	Trap Drive	29.0	Scan Begin	100 m/z
Ion Polarity	Positive	Octopole RF Amplitude	152.8 Vpp	Scan End	800 m/z
Ion Source Type	ESI	Capillary Exit	102.3 Volt	Averages	5 Spectra
Dry Temp (Set)	330 °C	Skimmer	40.0 Volt	Max. Accu Time	200000 µs
Nebulizer (Set)	15.00 psi	Oct 1 DC	12.00 Volt	ICC Target	20000
Dry Gas (Set)	5.00 l/min	Oct 2 DC	1.70 Volt	Charge Control	on

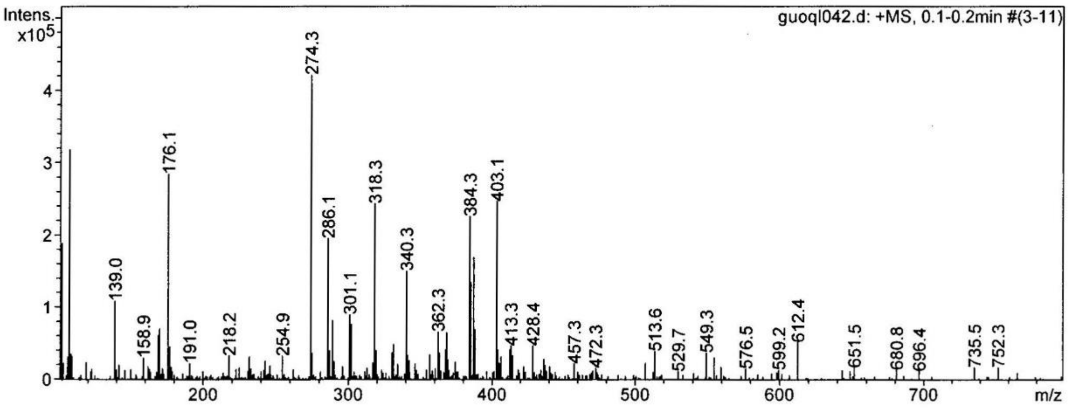

Component	Molecular Mass	Molecule	Absolute Abundance	Relative Abundance

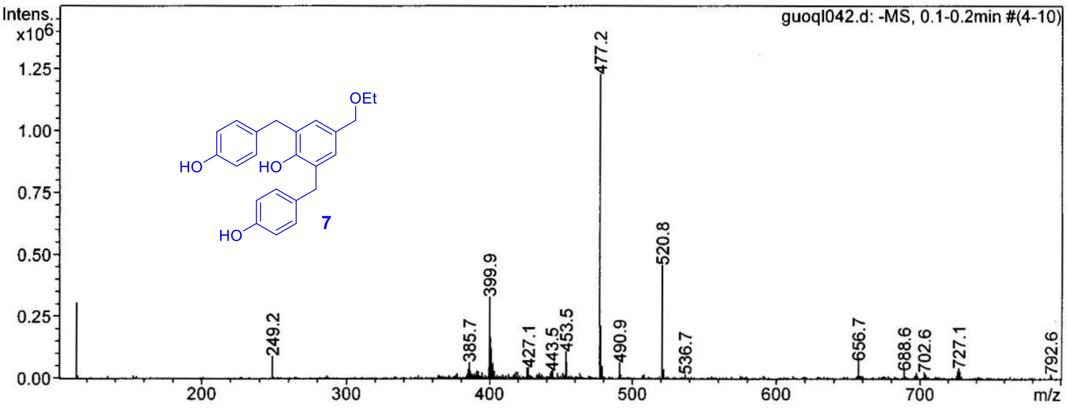

Component	Molecular Mass	Molecule	Absolute Abundance	Relative Abundance

Fig. S75 The ESI-MS of compound **7**.

Qualitative Analysis Report

**Data Filename**	2012112303.d	**Sample Name**	TMG-26B
**Sample Type**	Sample	**Position**	P1-D5
**Instrument Name**	Instrument 1	**User Name**	
**Acq Method**		**IRM Calibration Status**	Success
**DA Method**	TEST LCMS.m	**Comment**	

User Chromatograms

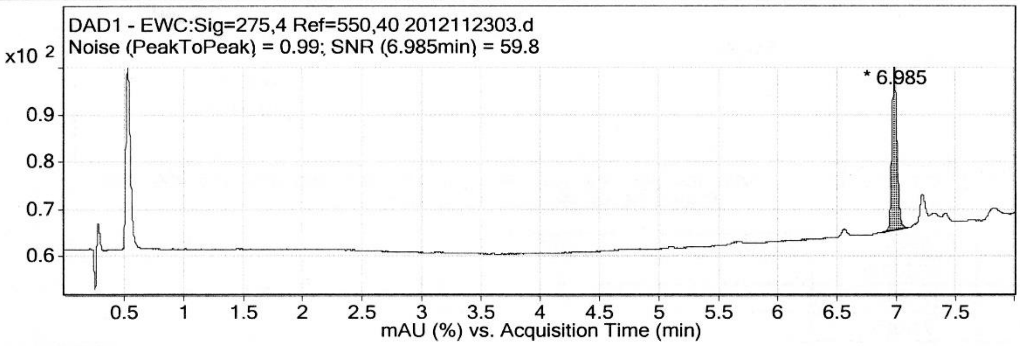

Integration Peak List

Peak	Start	RT	End	Height	Area	Area %	Signal To Noise
1	6.878	6.985	7.096	19.91	59.502	100	59.8

Noise Measurements

Noise Type	Signal Definition	Noise Multiplier	Noise Value
Peak-to-Peak	Area	1	0.994330406

Noise Regions

Start	End
1	2
4.5	5
8.6	9.4
9.8	11

Fragmentor Voltage 135 **Collision Energy** 0 **Ionization Mode** ESI

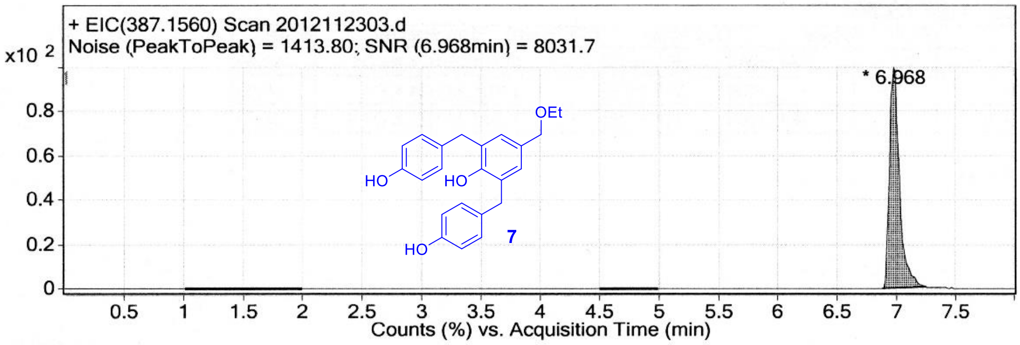

Integration Peak List

Peak	Start	RT	End	Height	Area	Area %	Signal To Noise
1	6.872	6.968	7.242	1959328	11355227	100	8031.7

Noise Measurements

Noise Type	Signal Definition	Noise Multiplier	Noise Value
Peak-to-Peak	Area	1	1413.798218

Noise Regions

Start	End
1	2
4.5	5
8.6	9.4
9.8	11

Fig. S76 The (+)-HR-ESI-MS report of compound **7**, page 1.

Qualitative Analysis Report

User Spectra

Fragmentor Voltage	Collision Energy	Ionization Mode
135	0	Esi

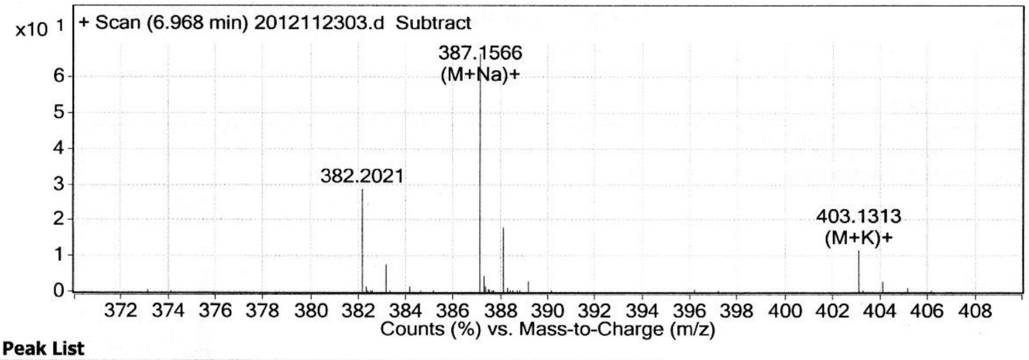

Peak List

m/z	z	Abund	Formula	Ion
319.1327	1	2957095		
319.2842		213167		
320.1368	1	747365		
382.2021	1	855406		
383.2049	1	220482		
387.1566	1	1963760	C23 H24 Na O4	(M+Na)+
388.1609	1	530508	C23 H24 Na O4	(M+Na)+
403.1313		345095	C23 H24 K O4	(M+K)+

Formula Calculator Element Limits

Element	Min	Max
C	3	100
H	0	500
O	0	90
N	0	2
S	0	2
Cl	0	0
Br	0	1

Formula Calculator Results

Formula	Best	Mass	Tgt Mass	Diff (ppm)	Ion Species	Score
C23 H24 O4	TRUE	364.1674	364.1675	0.22	C23 H24 Na O4	99.89
C23 H24 O4	TRUE	364.1682	364.1675	-1.91	C23 H24 K O4	99.89
C20 H28 O4 S		364.1682	364.1708	7.34	C20 H28 K O4 S	98.23

--- End Of Report ---

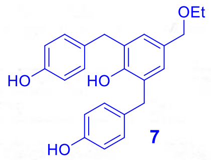

7

Fig. S77 The (+)-HR-ESI-MS report of compound **7**, page 2.

MS Formula Results: + Scan (6.968 min) Sub (2012112303.d)

m/z	Ion	Formula	Abundance
387.1566	(M+Na)+	C23 H24 Na O4	1963759.6

Best	Formula (M)	Ion Formula	Calc m/z	Score	Cross S	Mass	Calc Mass	Diff (ppm)	Abs Diff (ppm)	Abund Match	Spacing Mat	Mass Match	m/z	DBE
☑	C23 H24 O4	C23 H24 Na O4	387.1567	99.89		364.1674	364.1675	0.22	0.22	99.74	99.86	100	387.1566	12

m/z	Ion	Formula	Abundance
403.1313	(M+K)+	C23 H24 K O4	345094.9

Best	Formula (M)	Ion Formula	Calc m/z	Score	Cross S	Mass	Calc Mass	Diff (ppm)	Abs Diff (ppm)	Abund Match	Spacing Mat	Mass Match	m/z	DBE
☑	C23 H24 O4	C23 H24 K O4	403.1306	99.89		364.1682	364.1675	-1.91	1.91	99.92	99.85	99.9	403.1313	12
☐	C20 H28 O4 S	C20 H28 K O4 S	403.134	98.23		364.1682	364.1708	7.34	7.34	96.32	99.86	98.56	403.1313	7

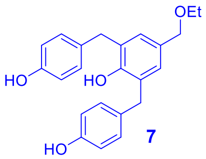

7

page 1

Fig. S78 The (+)-HR-ESI-MS report of compound **7**, page 3.

VNS-600 PROTON_01 TMG-26B IN acetone Nov 11 2012

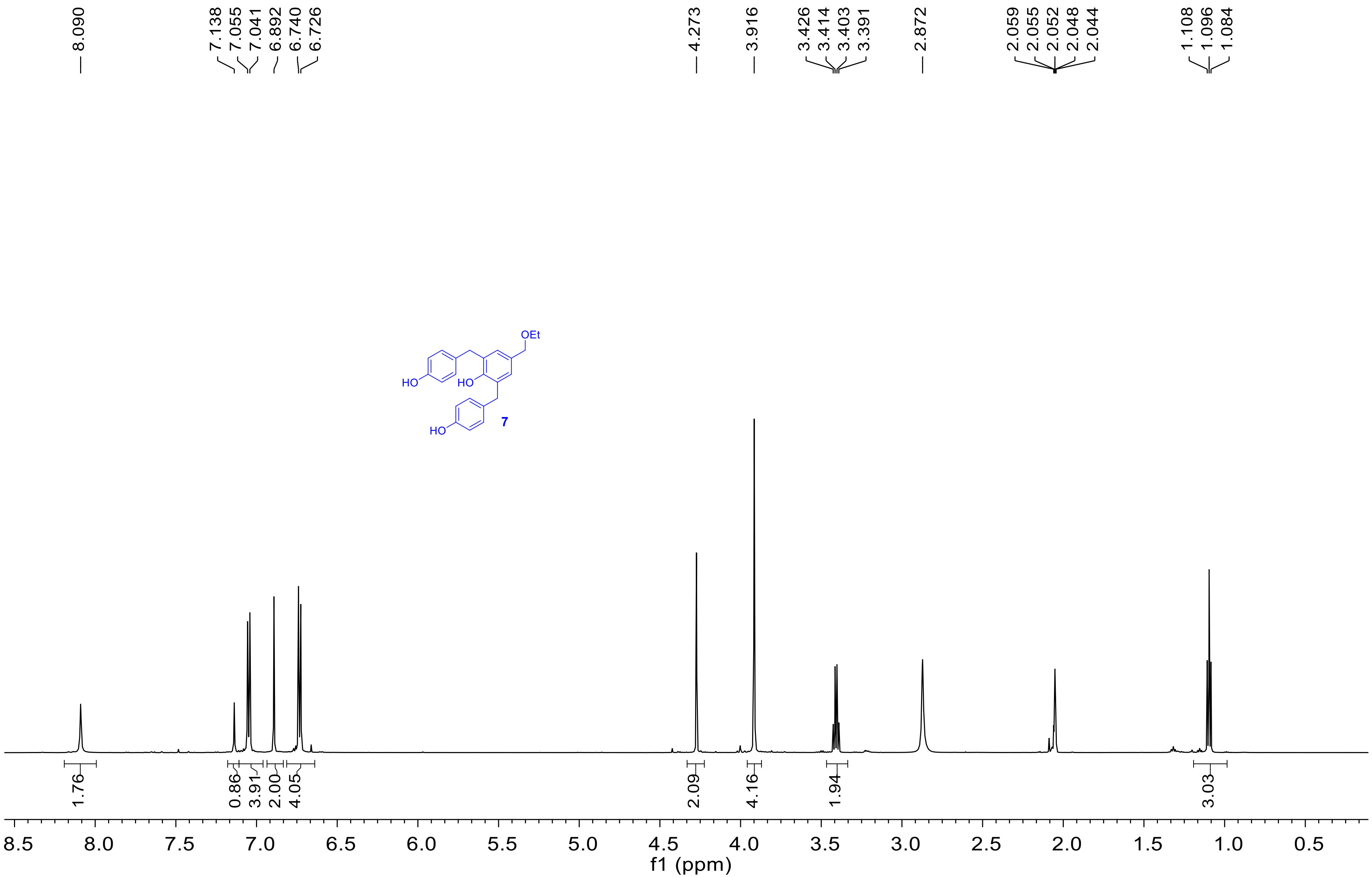

Fig. S79 The ^{1}H NMR spectrum of compound **7** in acetone-d_6 (600 MHz).

VNS-600 CARBON TMG-26B IN acetone Nov 11 2012

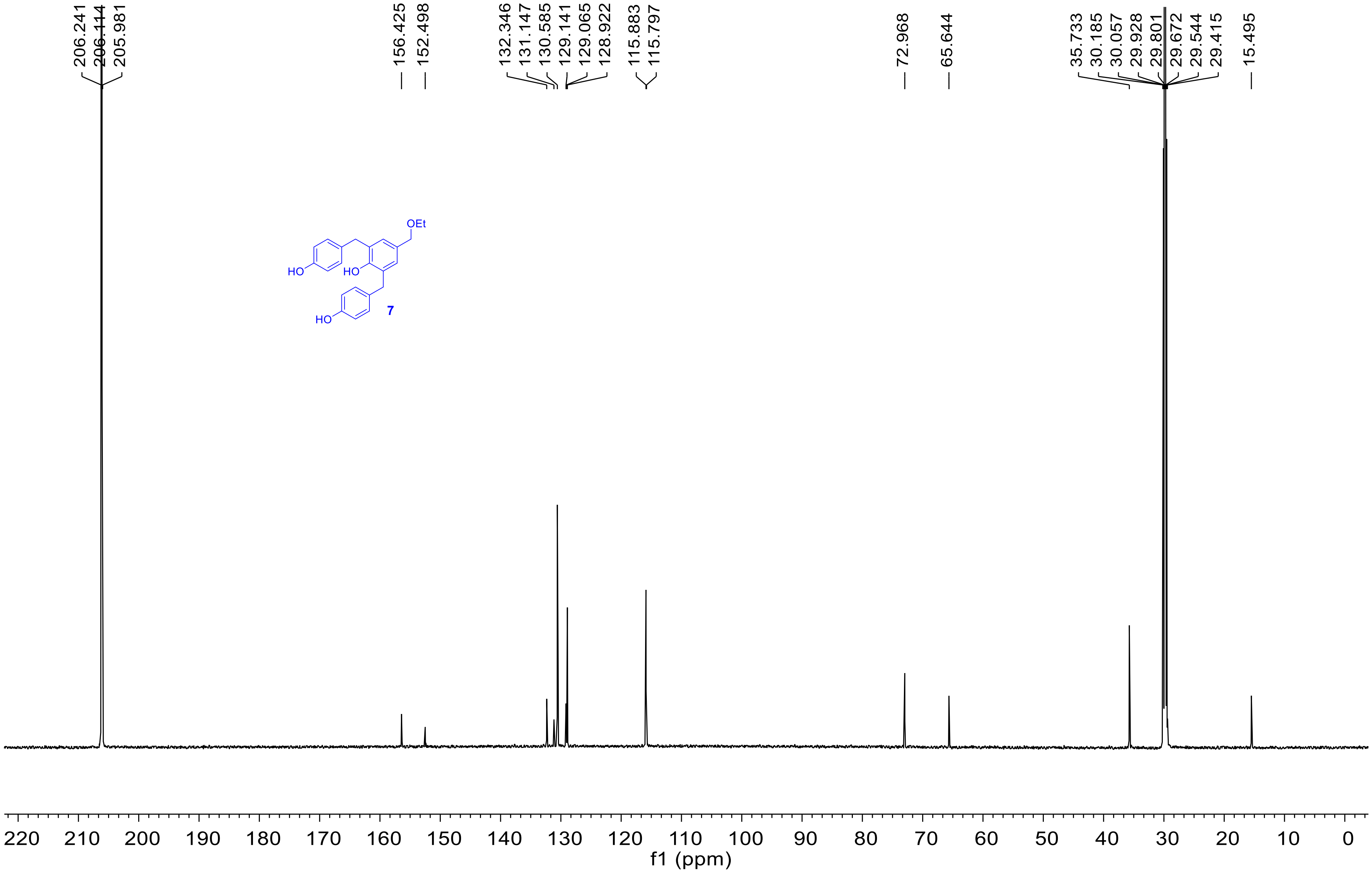

Fig. S80 The ^{13}C NMR spectrum of compound **7** in acetone-d_6 (150 MHz).

DEPT_01
DD2-500 DEPT TMG-26B IN acetone May 22 2012 sw-Probe

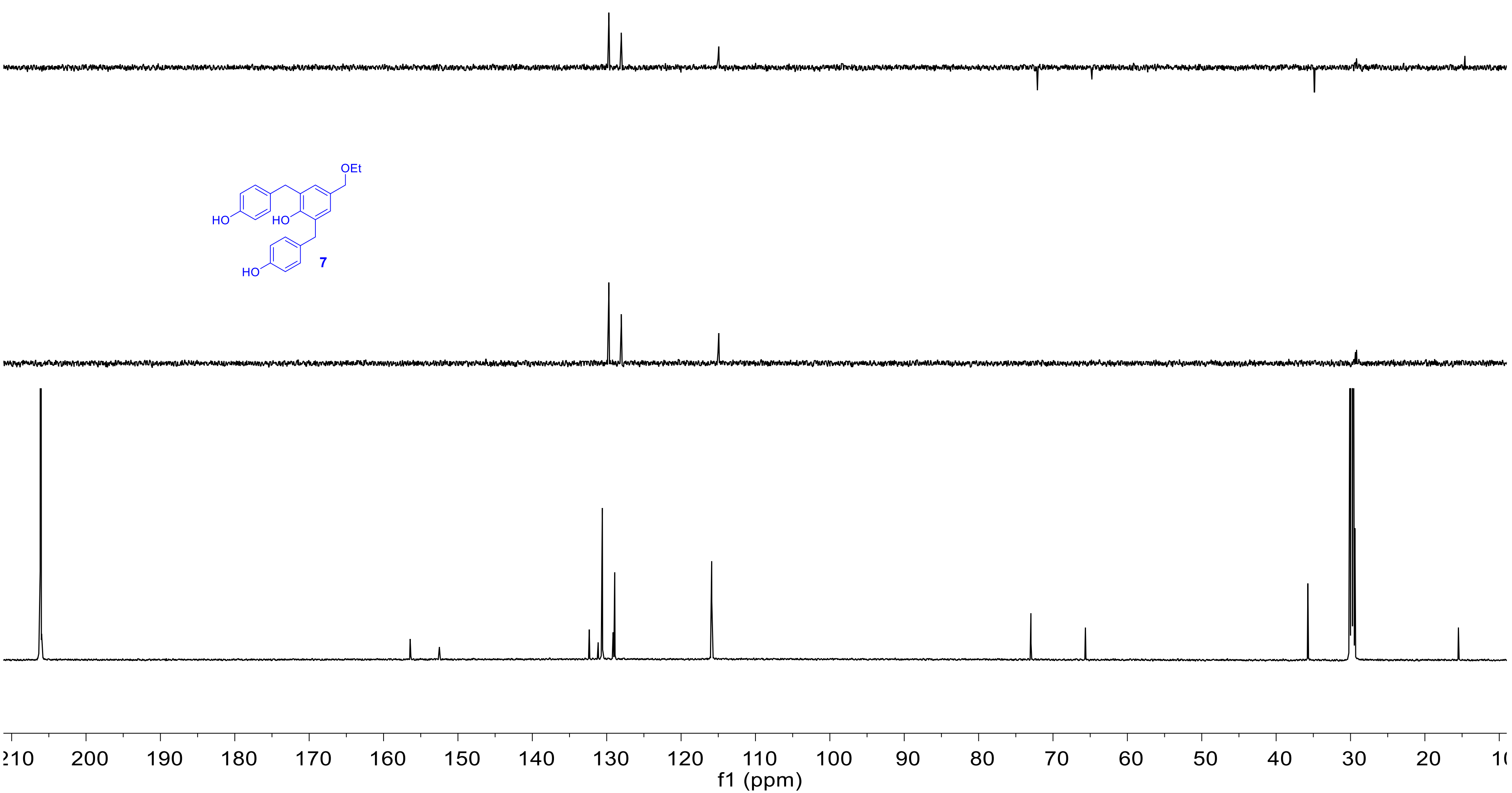

Fig. S81 The DEPT spectrum of compound **7** in acetone-d_6 (125 MHz).

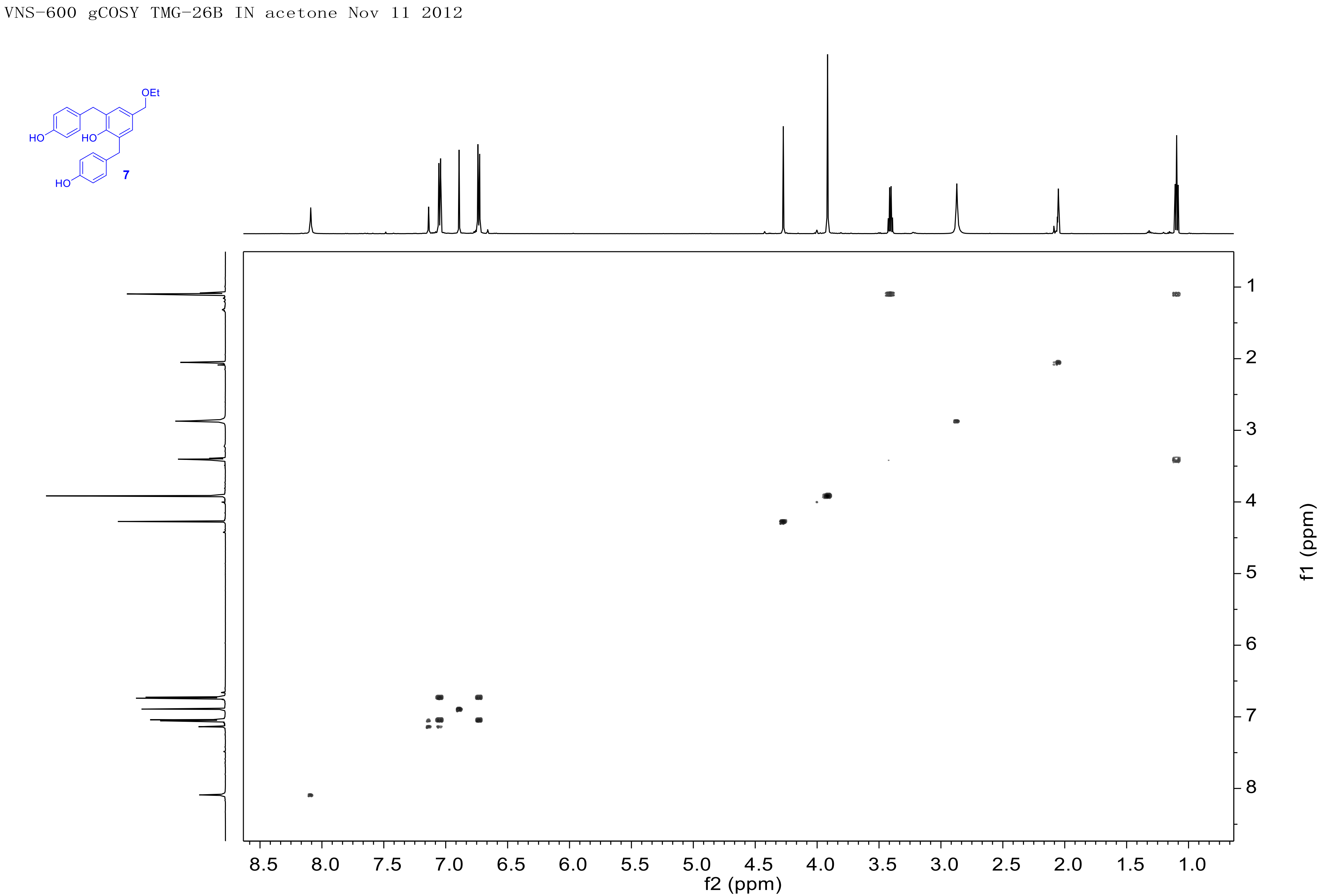

Fig. S82 The ^{1}H-^{1}H COSY spectrum of compound **7** in acetone-d_6 (600 MHz).

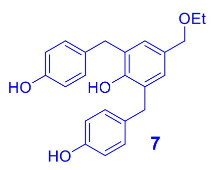

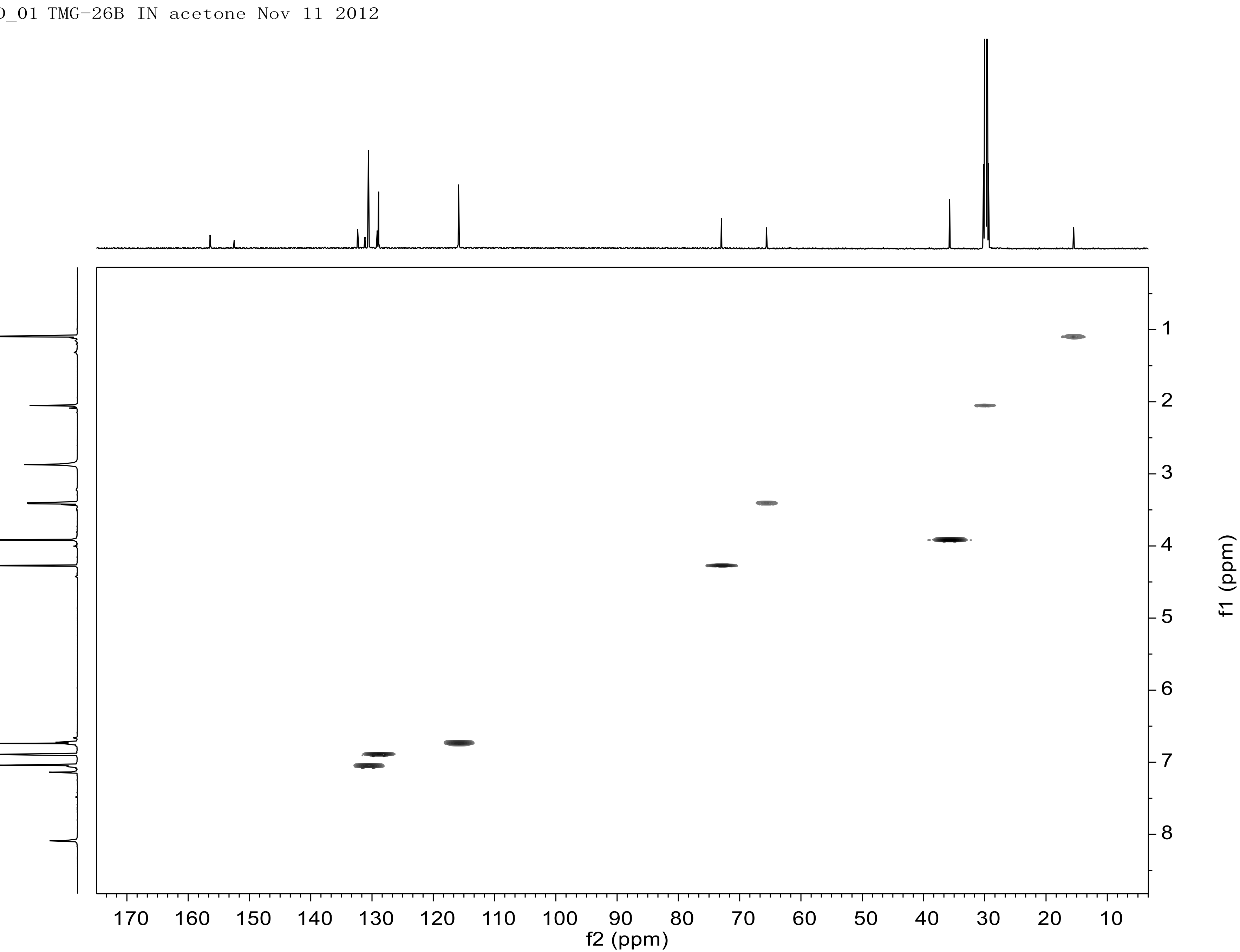

Fig. S83 The HSQC spectrum of compound **7** in acetone-d_6 (600 MHz for ^{1}H).

VNS-600 gHMBCAD_01 TMG-26B IN acetone Nov 11 2012

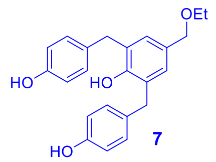

7

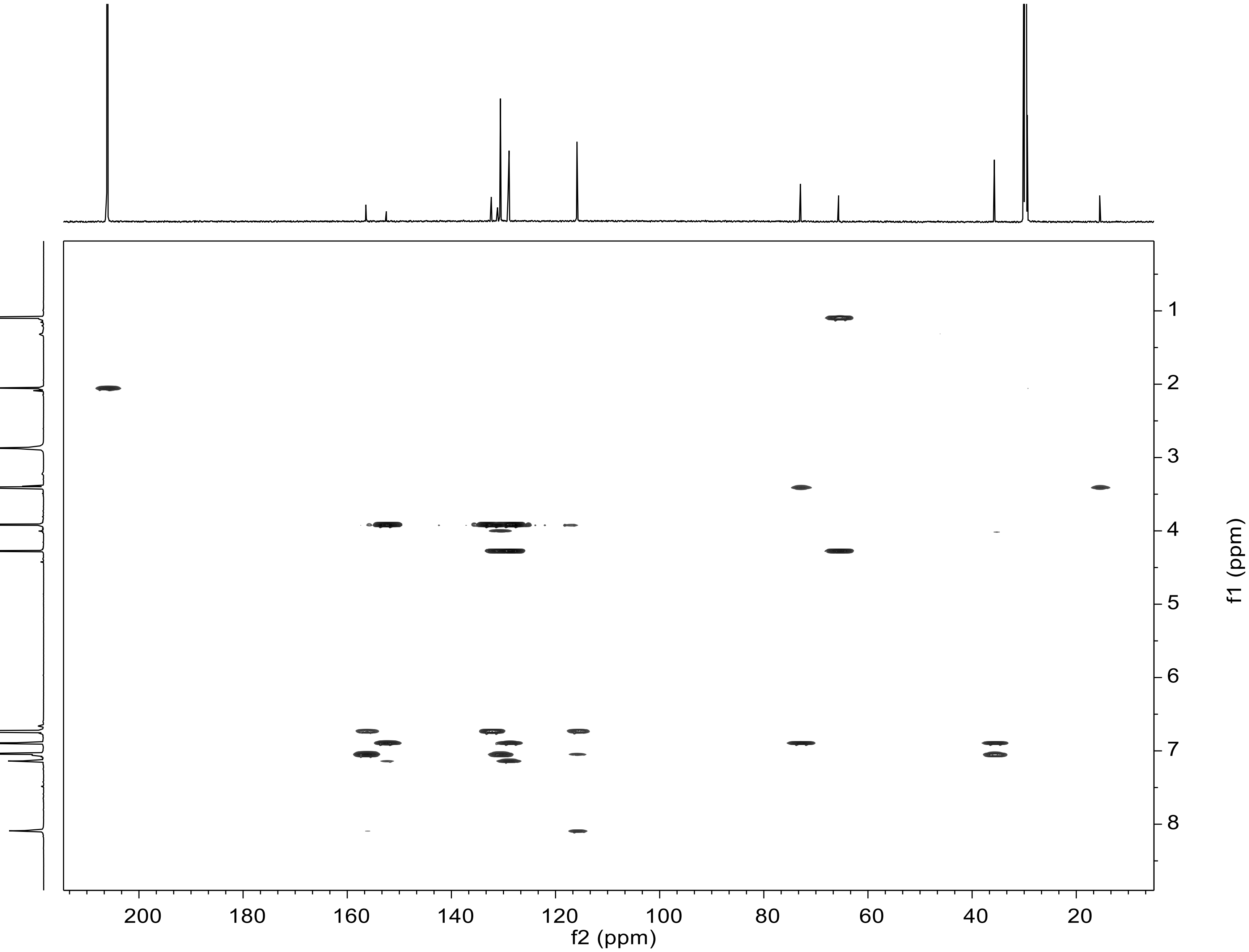

Fig. S84 The HMBC spectrum of compound **7** in acetone-d_6 (600 MHz for ^{1}H).

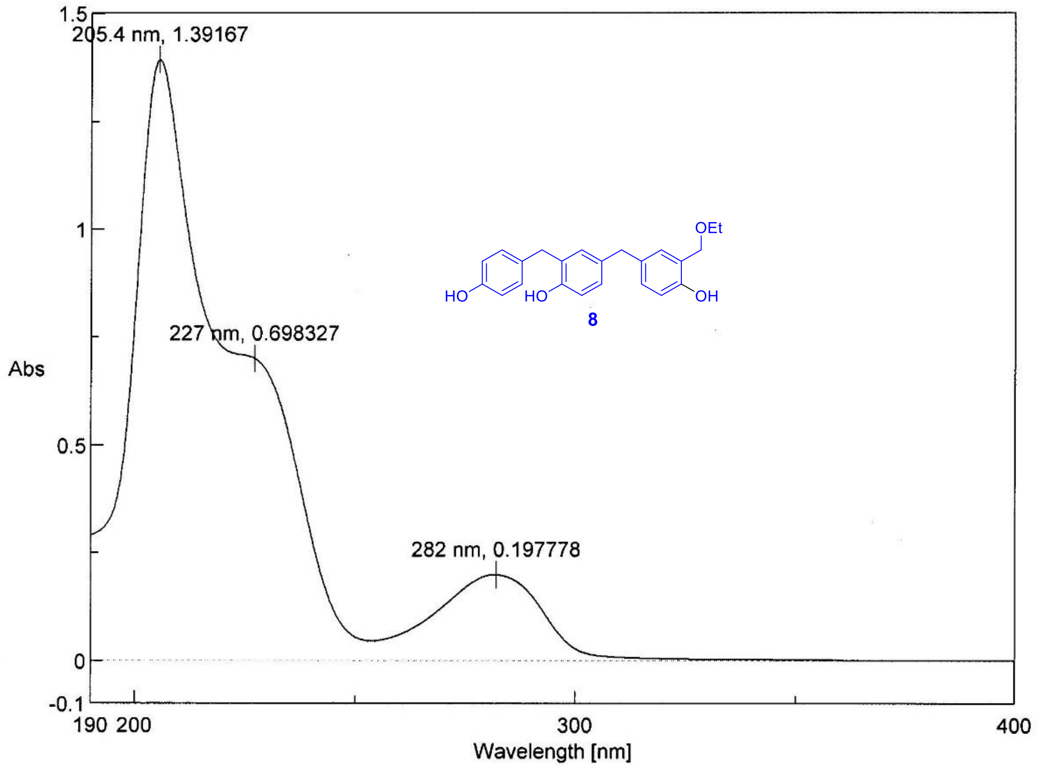

Memory-4

[Comment]

Sample Name	TMG-27a
Comment	0.02
User	
Division	UV
Company	324

[Measurement Information]

Instrument Name	V-650
Model Name	V-650
Serial No.	A034461150
Accessory	PSC-718
Accessory S/N	A001761114
Position	1
Cell Length	10 mm
Temperature	19.95 C
Control Sonsor	Holder
Monitor Sensor	Holder
Start Mode	Start immediately
Photometric Mode	Abs
Measurement range	400 - 190 nm
Data pitch	0.2 nm
Band width(UV/Vis)	2.0 nm
Response	Medium
Scanning speed	200 nm/min
Source Change	340 nm
Light Source	D2/WI
Filter Exchange	Step
Correction	Baseline

[Data Information]

Creation Date	2014-2-26 9:20
Data array type	Linear data array
Horizontal	Wavelength [nm]
Vertical	Abs
Start	400 nm
End	190 nm
Data pitch	0.2 nm
Data points	1051

Fig. S85 The UV spectrum of compound **8**.

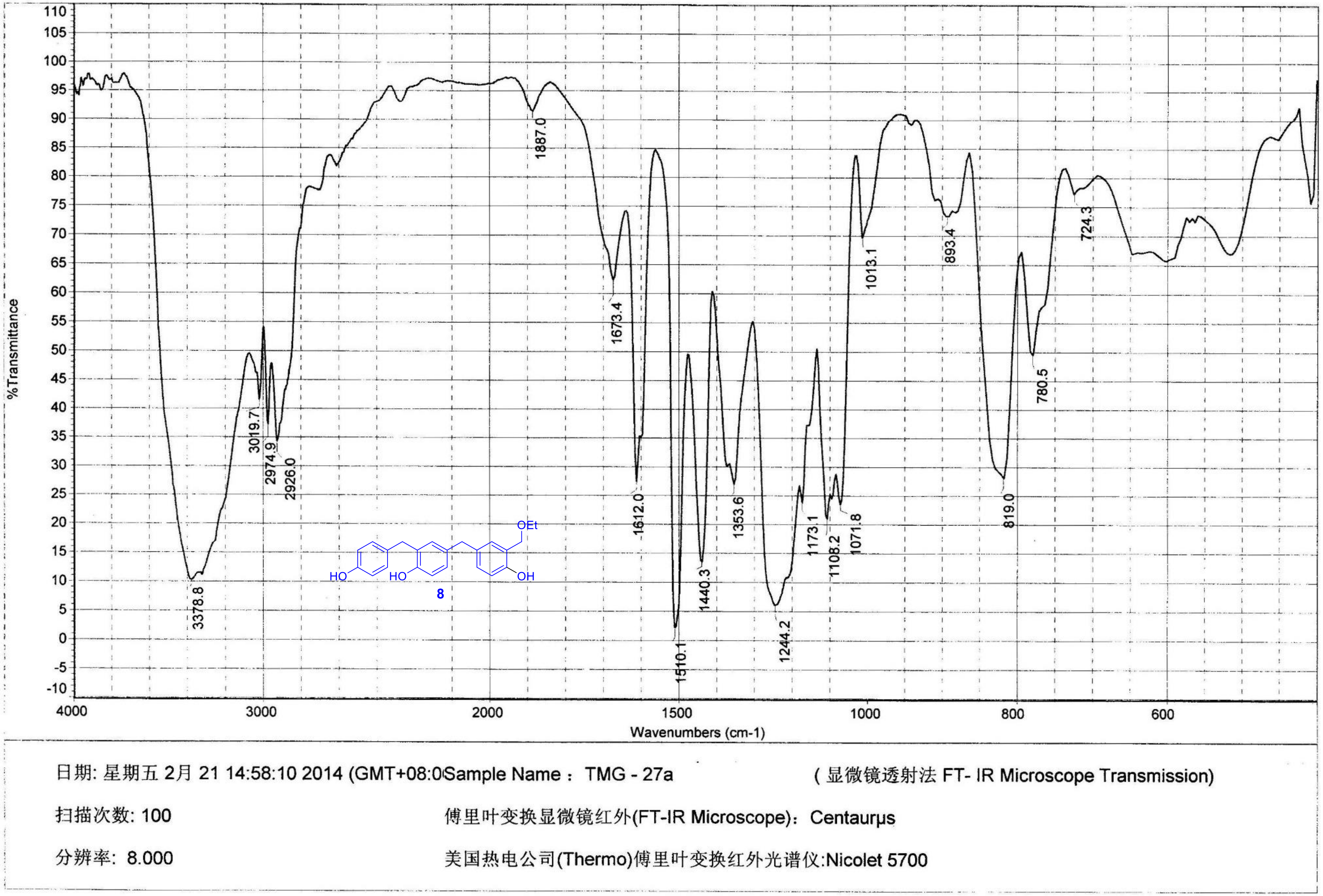

Fig. S86 The IR spectrum of compound **8**.

Single Mass Spectrum Deconvolution Report

Analysis Name: guoql041.d
Method: TEST.MS
Sample Name: TMG-27a
Analysis Info:

Instrument: LC-MSD-Trap-SL
Operator: Operator

Print Date: 7/20/2011 9:54:02 AM
Acq. Date: 7/20/2011 9:46:45 AM

Acquisition Parameter:

Mass Range Mode	Std/Normal	Trap Drive	45.5	Scan Begin	100 m/z
Ion Polarity	Positive	Octopole RF Amplitude	152.8 Vpp	Scan End	800 m/z
Ion Source Type	ESI	Capillary Exit	-102.3 Volt	Averages	5 Spectra
Dry Temp (Set)	330 °C	Skimmer	-40.0 Volt	Max. Accu Time	200000 µs
Nebulizer (Set)	15.00 psi	Oct 1 DC	-12.00 Volt	ICC Target	20000
Dry Gas (Set)	5.00 l/min	Oct 2 DC	-1.70 Volt	Charge Control	on

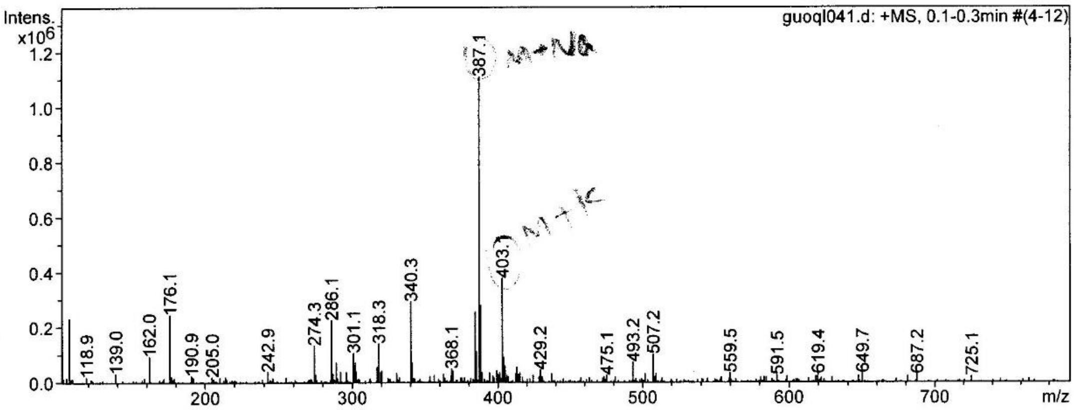

Component	Molecular Mass	Molecule	Absolute Abundance	Relative Abundance

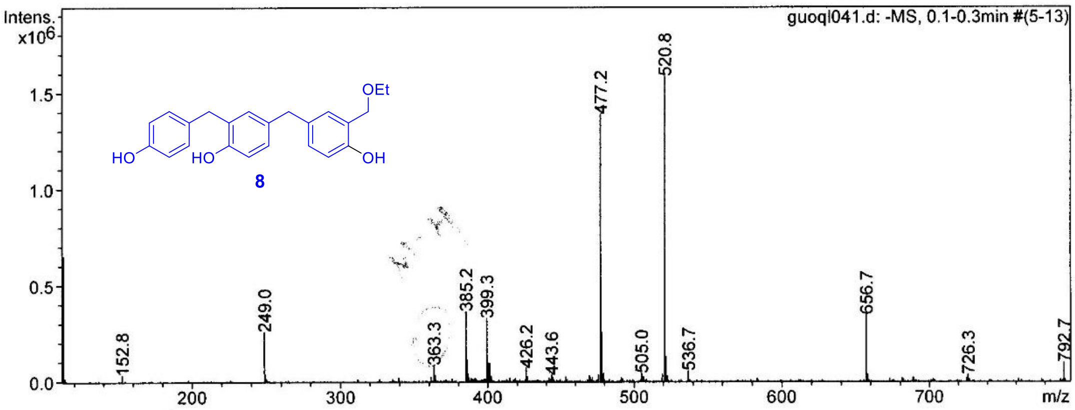

Component	Molecular Mass	Molecule	Absolute Abundance	Relative Abundance

Fig. S87 The ESI-MS of compound **8**.

Qualitative Analysis Report

**Data Filename**	2012112304.d	**Sample Name**	TMG-27a
**Sample Type**	Sample	**Position**	P1-D6
**Instrument Name**	Instrument 1	**User Name**	
**Acq Method**		**IRM Calibration Status**	Success
**DA Method**	TEST LCMS.m	**Comment**	

User Chromatograms

Fragmentor Voltage 135 **Collision Energy** 0 **Ionization Mode** ESI

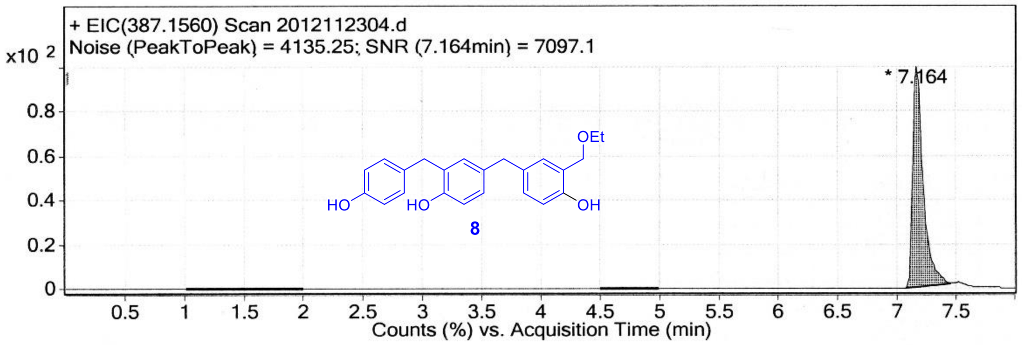

Integration Peak List

Peak	Start	RT	End	Height	Area	Area %	Signal To Noise
1	7.067	7.164	7.454	4590065	29348258	100	7097.1

Noise Measurements

Noise Type	Signal Definition	Noise Multiplier	Noise Value
Peak-to-Peak	Area	1	4135.248535

Noise Regions

Start	End
1	2
4.5	5
8.6	9.4
9.8	11

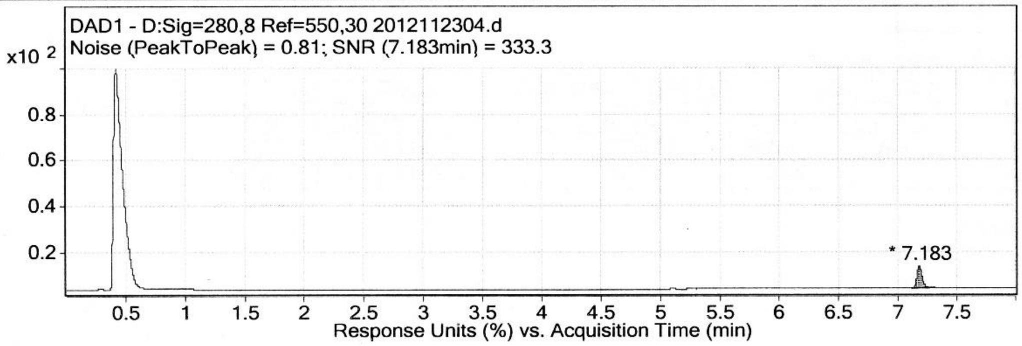

Integration Peak List

Peak	Start	RT	End	Height	Area	Area %	Signal To Noise
1	7.11	7.183	7.316	87.81	269.27	100	333.3

Noise Measurements

Noise Type	Signal Definition	Noise Multiplier	Noise Value
Peak-to-Peak	Area	1	0.807762146

Noise Regions

Start	End
1	2
4.5	5
8.6	9.4
9.8	11

Fig. S88 The (+)-HR-ESI-MS report of compound **8**, page 1.

Qualitative Analysis Report

User Spectra

Fragmentor Voltage	Collision Energy	Ionization Mode
135	0	Esi

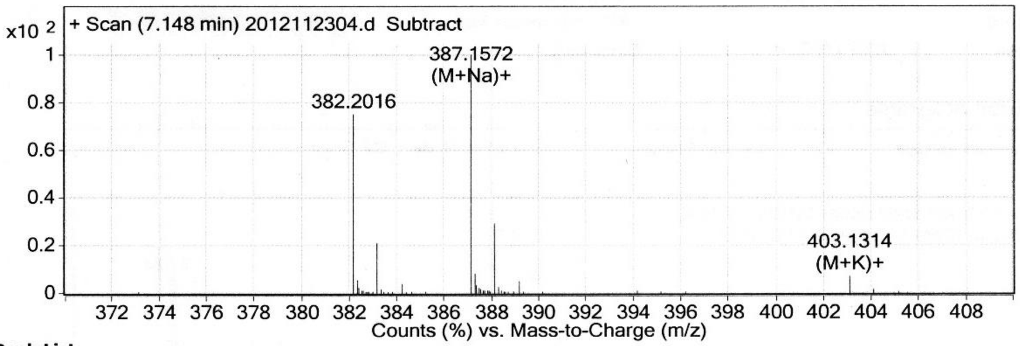

Peak List

m/z	z	Abund	Formula	Ion
237.1123		280871		
319.1329	1	1997481		
320.1366	1	488674		
382.2016	1	3162629		
383.2053	1	896802		
387.1572	1	4197111	C23 H24 Na O4	(M+Na)+
387.3239		335597		
388.1608	1	1232075	C23 H24 Na O4	(M+Na)+
403.1314		301373	C23 H24 K O4	(M+K)+
543.2177	1	626310		

Formula Calculator Element Limits

Element	Min	Max
C	3	100
H	0	500
O	0	90
N	0	2
S	0	2
Cl	0	0
Br	0	1

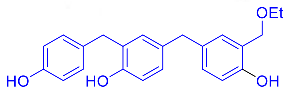

8

Formula Calculator Results

Formula	Best	Mass	Tgt Mass	Diff (ppm)	Ion Species	Score
C23 H24 O4	TRUE	364.168	364.1675	-1.46	C23 H24 Na O4	99.58
C23 H24 O4	TRUE	364.1682	364.1675	-2.03	C23 H24 K O4	99.93
C20 H28 O4 S		364.1682	364.1708	7.22	C20 H28 K O4 S	98.34

--- End Of Report ---

Fig. S89 The (+)-HR-ESI-MS report of compound **8**, page 2.

MS Formula Results: + Scan (7.148 min) Sub (2012112304.d)

m/z	Ion	Formula	Abundance
387.1572	(M+Na)+	C23 H24 Na O4	4197110.5

Best	Formula (M)	Ion Formula	Calc m/z	Score	Cross S	Mass	Calc Mass	Diff (ppm)	Abs Diff (ppm)	Abund Match	Spacing Mat	Mass Match	m/z	DBE
☑	C23 H24 O4	C23 H24 Na O4	387.1567	99.58		364.168	364.1675	-1.46	1.46	98.64	100	99.94	387.1572	12

m/z	Ion	Formula	Abundance
403.1314	(M+K)+	C23 H24 K O4	301372.6

Best	Formula (M)	Ion Formula	Calc m/z	Score	Cross S	Mass	Calc Mass	Diff (ppm)	Abs Diff (ppm)	Abund Match	Spacing Mat	Mass Match	m/z	DBE
☑	C23 H24 O4	C23 H24 K O4	403.1306	99.93		364.1682	364.1675	-2.03	2.03	99.95	99.99	99.89	403.1314	12
☐	C20 H28 O4 S	C20 H28 K O4 S	403.134	98.34		364.1682	364.1708	7.22	7.22	96.63	99.85	98.6	403.1314	7

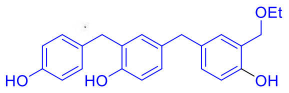

8

page 1

Fig. S90 The (+)-HR-ESI-MS report of compound **8**, page 3.

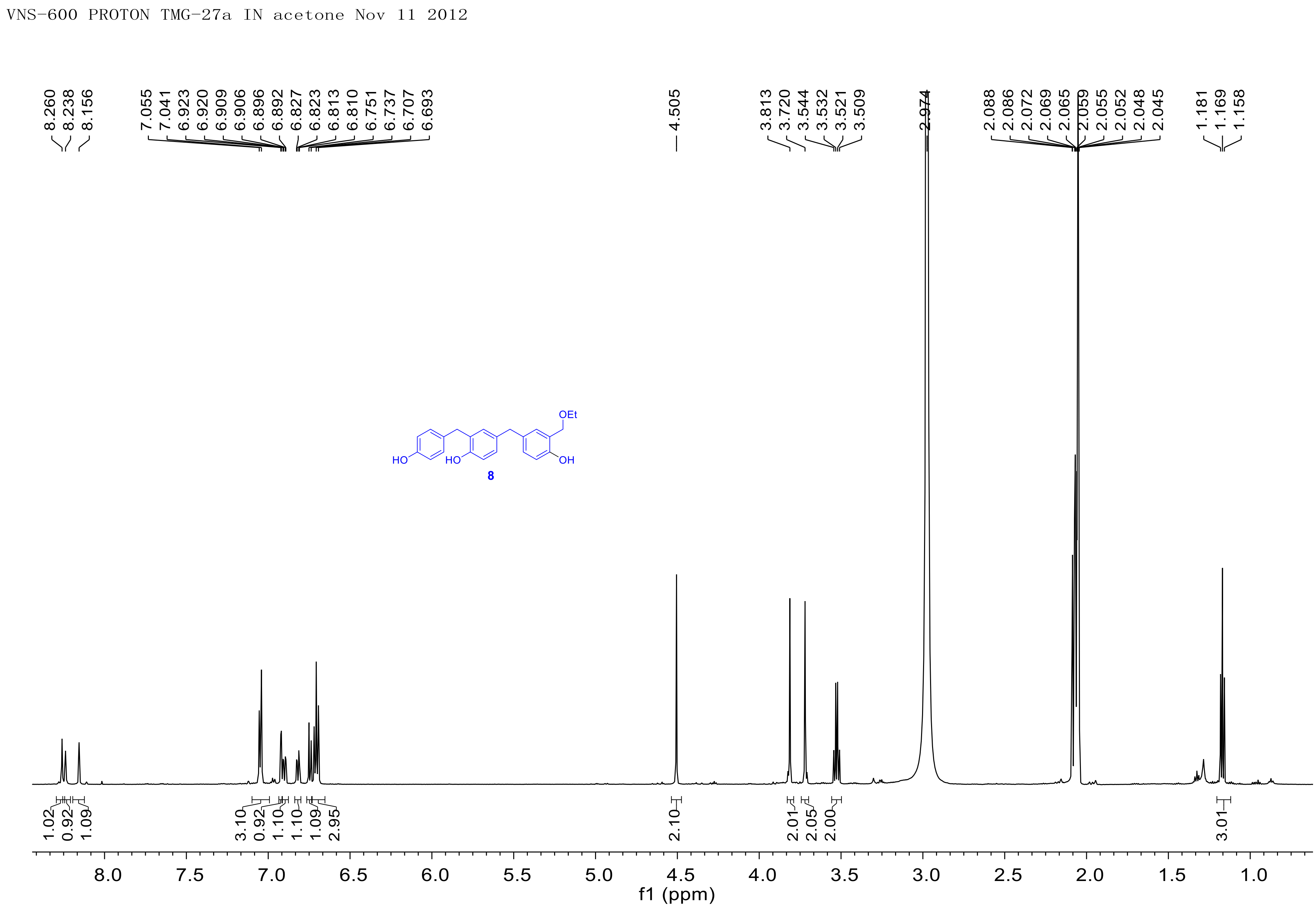

Fig. S91 The ^{1}H NMR spectrum of compound **8** in acetone-d_6 (600 MHz).

VNS-600 CARBON TMG-27a IN acetone Nov 11 2012

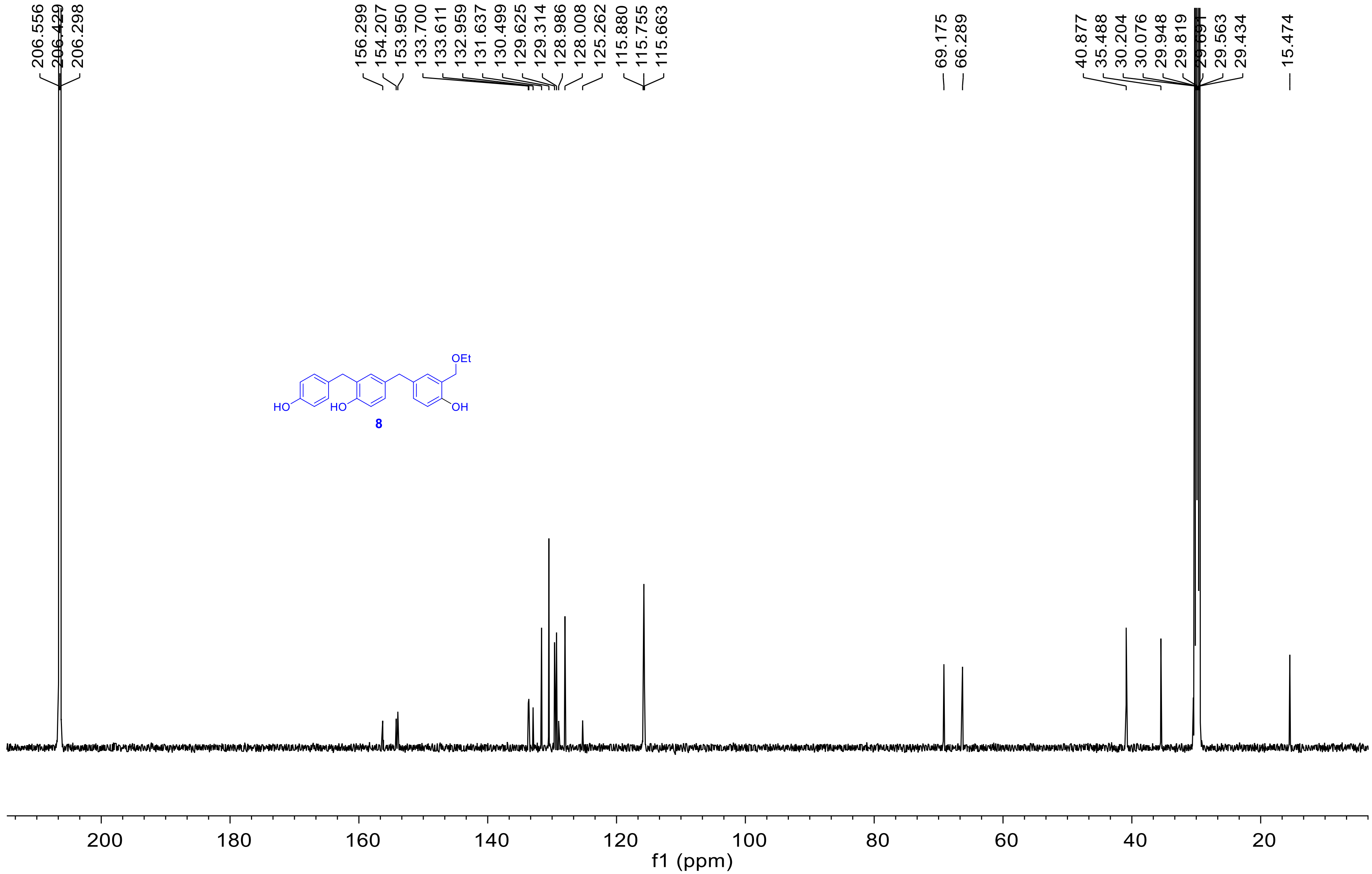

Fig. S92 The ^{13}C NMR spectrum of compound **8** in acetone-d_6 (150 MHz).

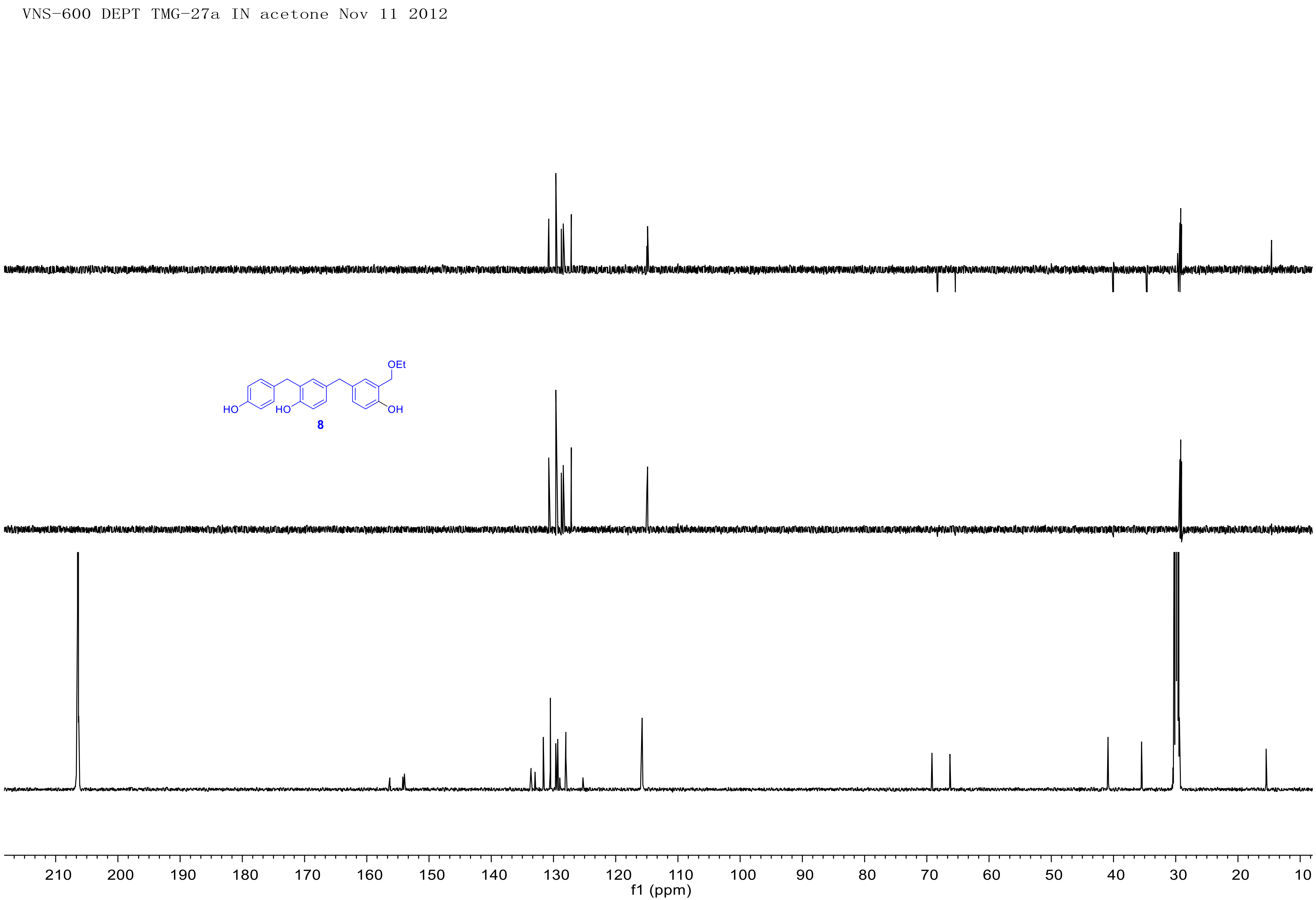

Fig. S93 The DEPT spectrum of compound **8** in acetone-d_6 (150 MHz).

VNS-600 gCOSY TMG-27a IN acetone Nov 11 2012

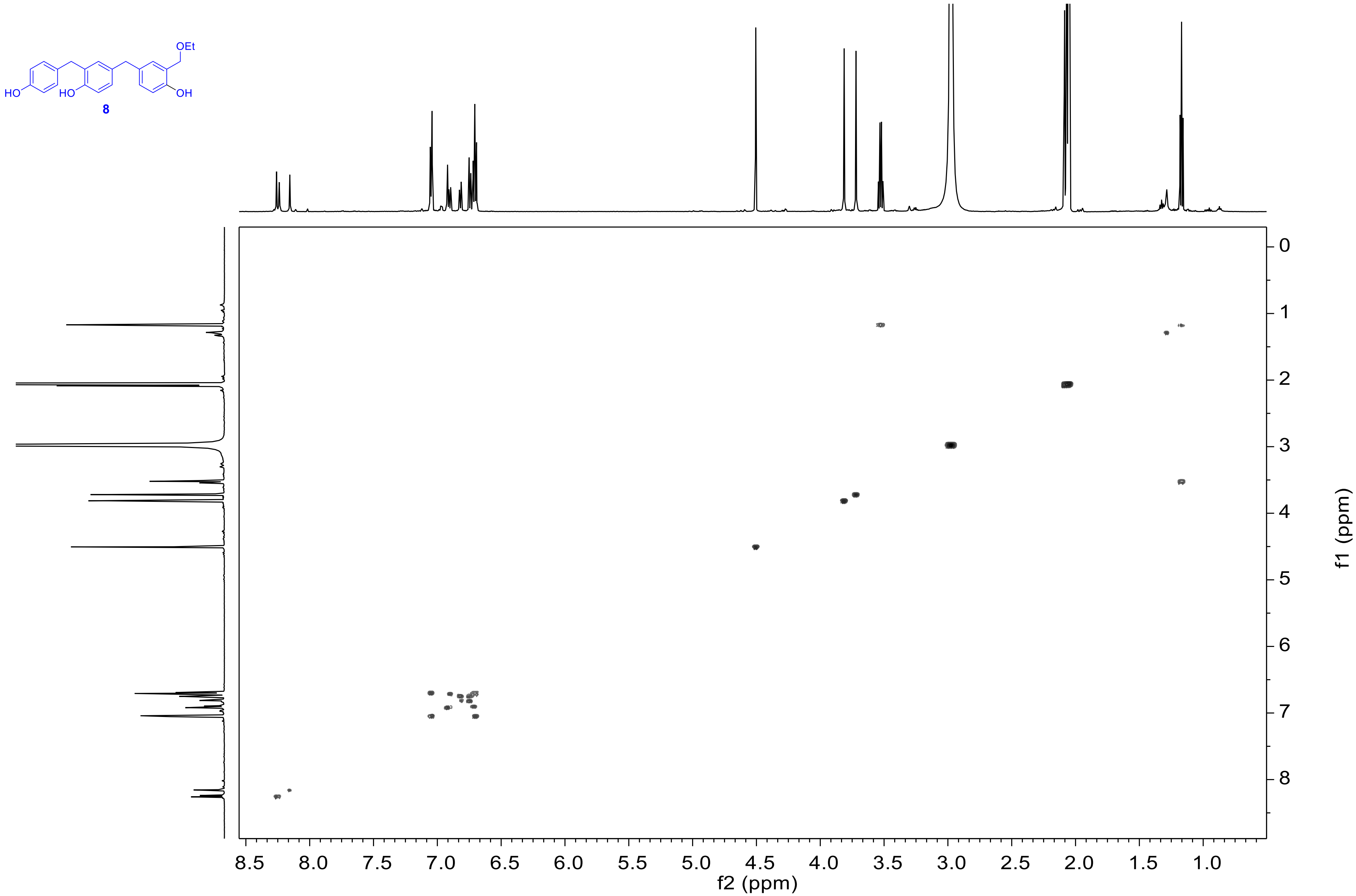

Fig. S94 The ^{1}H-^{1}H COSY spectrum of compound **8** in acetone-d_6 (600 MHz).

VNS-600 gHSQC TMG-27a IN acetone Nov 11 2012

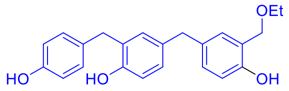

8

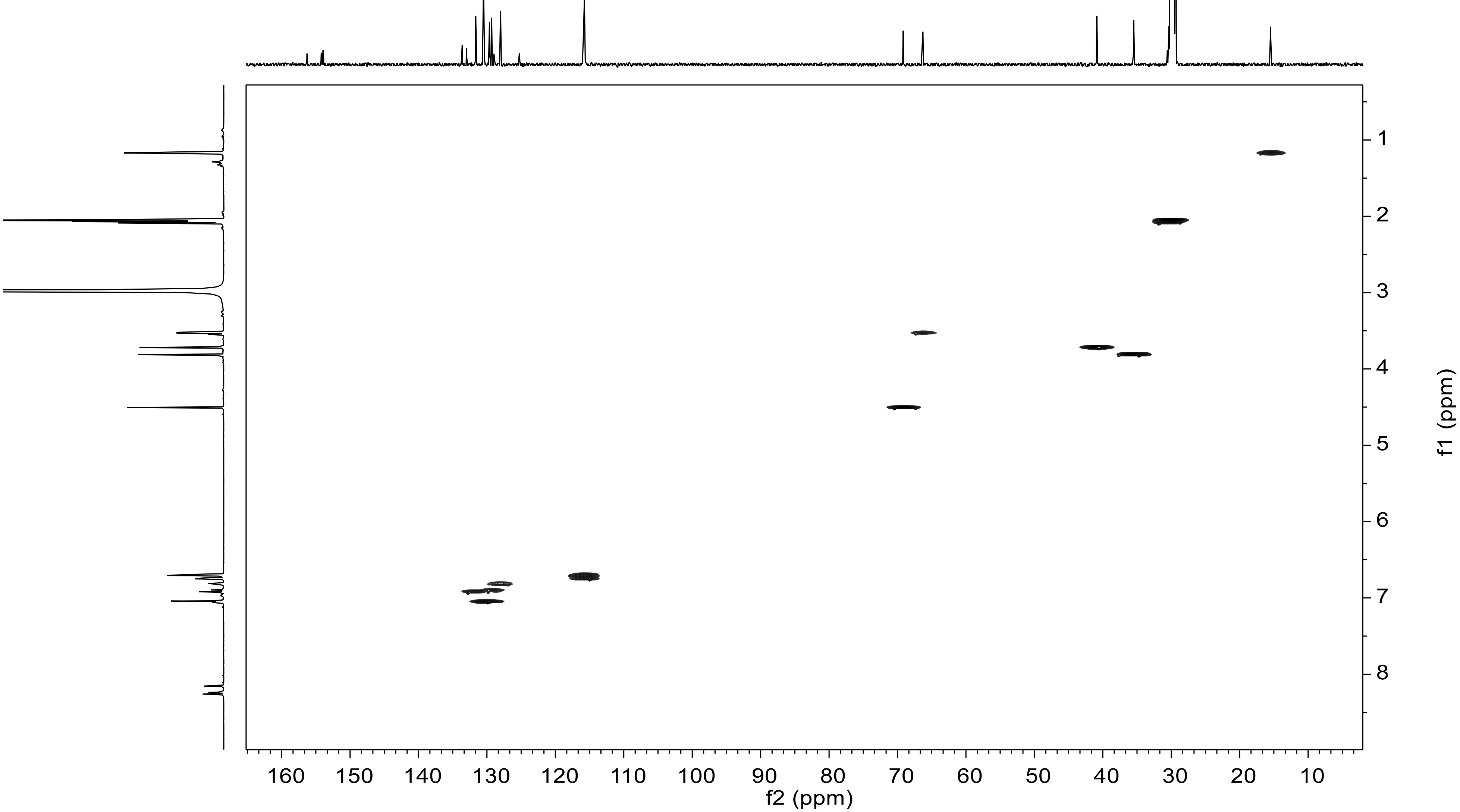

Fig. S95 The HSQC spectrum of compound **8** in acetone-d_6 (600 MHz for ^{1}H).

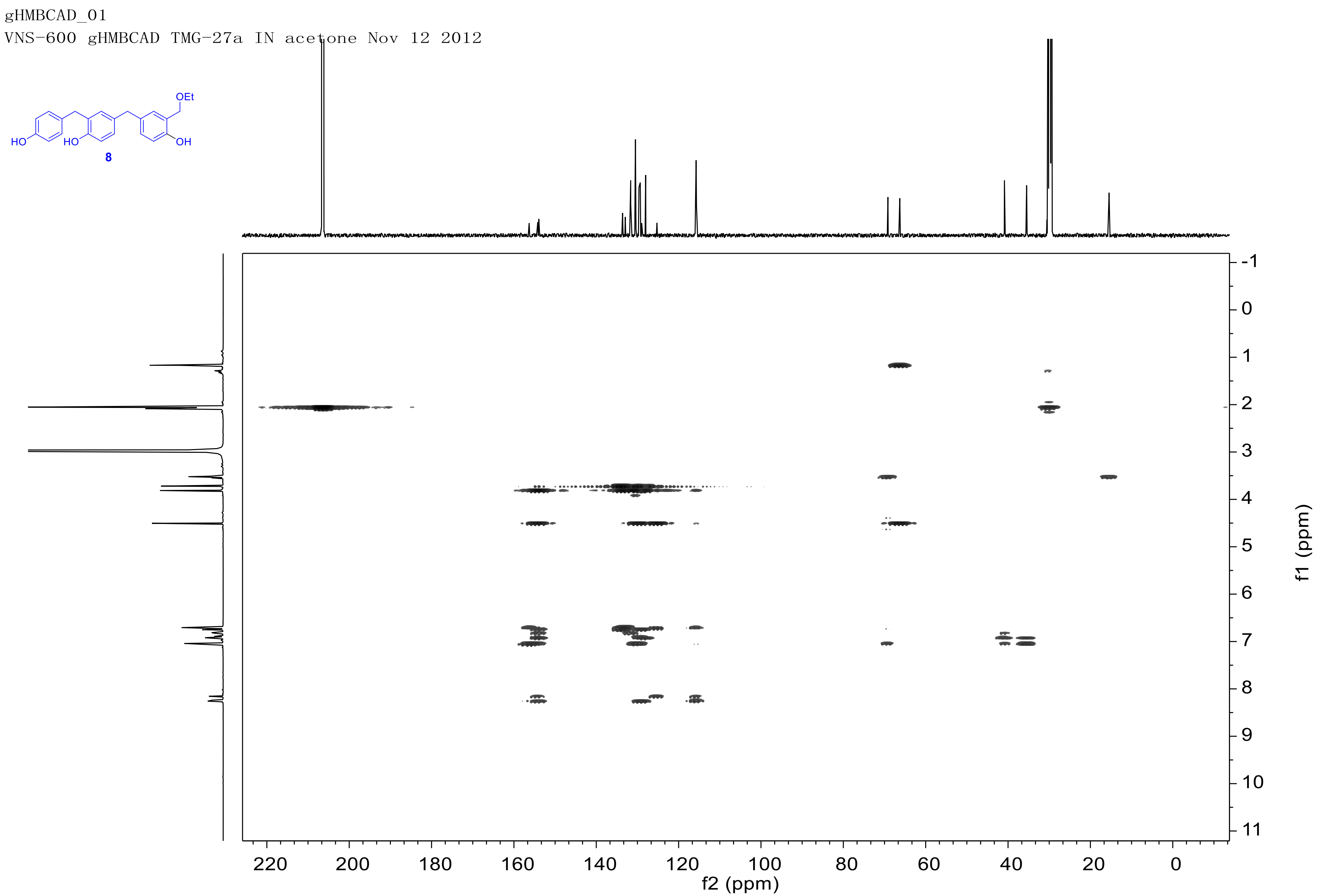

Fig. S96 The HMBC spectrum of compound **8** in acetone-d_6 (600 MHz for ^{1}H).

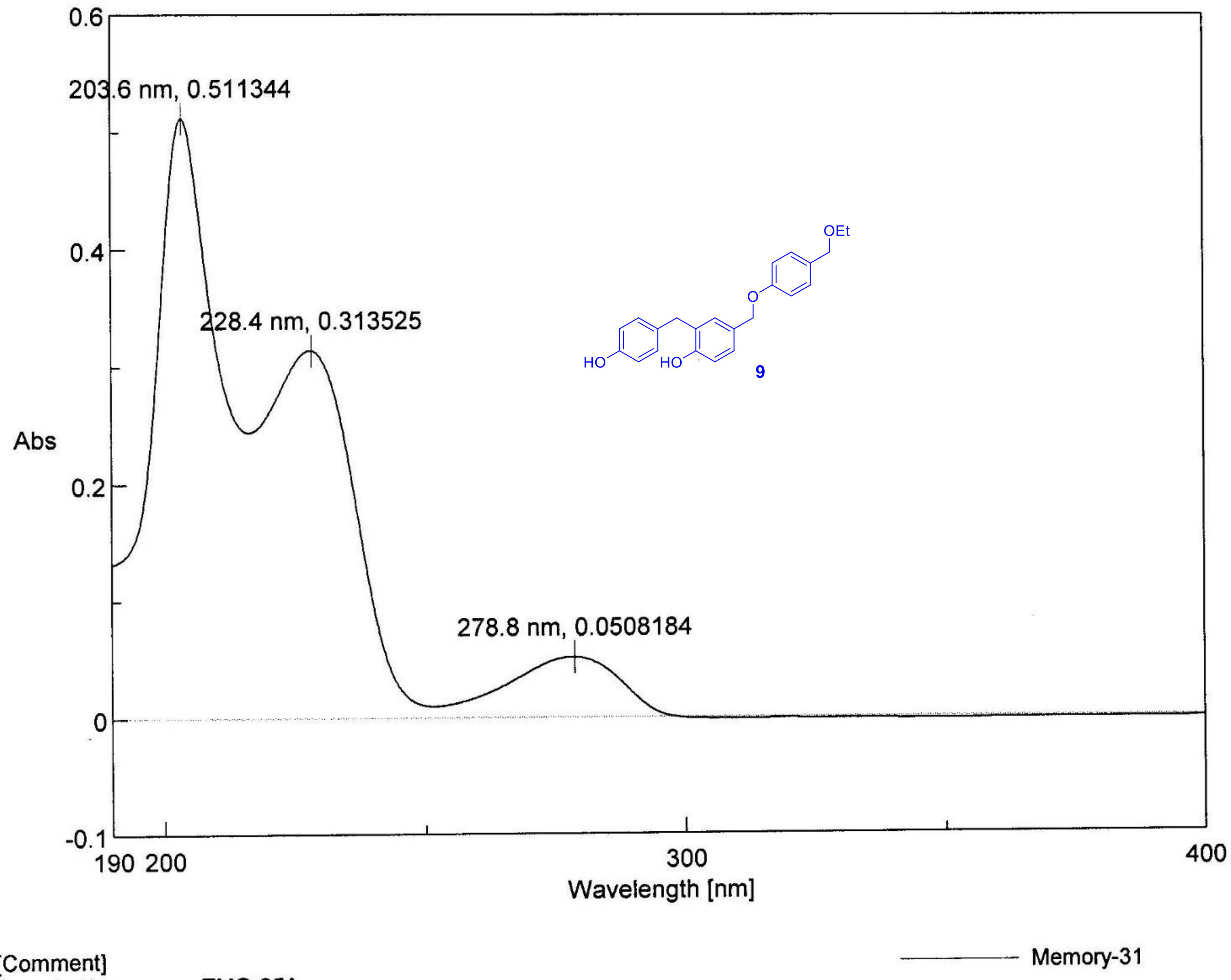

[Comment]
Sample Name TMG-25A
Comment 0.02
User
Division UV
Company 324

[Measurement Information]
Instrument Name V-650
Model Name V-650
Serial No. A034461150

Accessory PSC-718
Accessory S/N A001761114
Position 1
Cell Length 10 mm
Temperature 19.97 C
Control Sensor Holder
Monitor Sensor Holder
Start Mode Start immediately

Photometric Mode Abs
Measurement range 400 - 190 nm
Data pitch 0.2 nm
Band width(UV/Vis) 2.0 nm
Response Medium
Scanning speed 200 nm/min
Source Change 340 nm
Light Source D2/WI
Filter Exchange Step
Correction Baseline

[Data Information]
Creation Date 2014-2-26 10:05

Data array type Linear data array
Horizontal Wavelength [nm]
Vertical Abs
Start 400 nm
End 190 nm
Data pitch 0.2 nm
Data points 1051

Fig. S97 The UV spectrum of compound **9**.

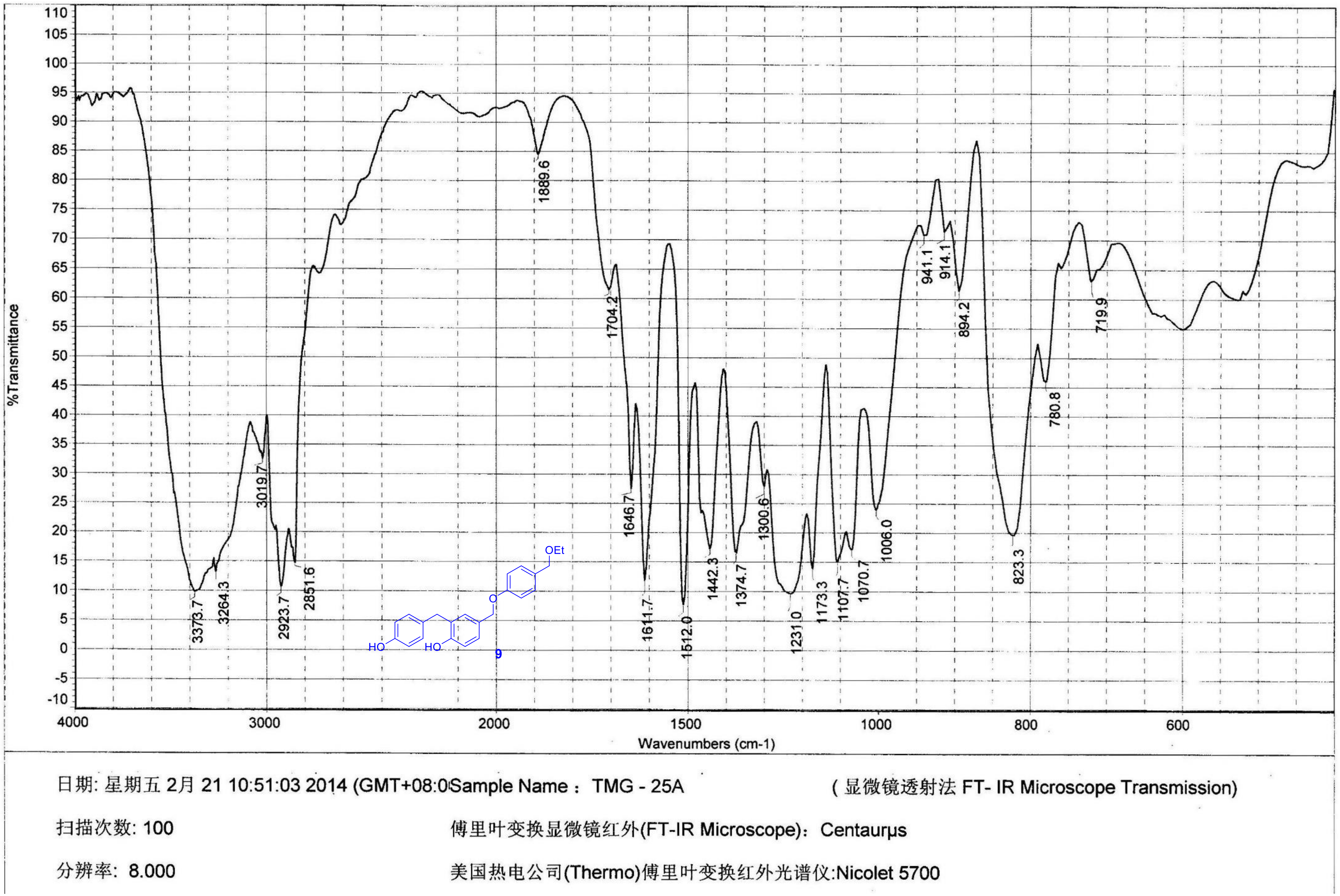

Fig. S98 The IR spectrum of compound **9**.

Single Mass Spectrum Deconvolution Report

Analysis Name: guoql148.d
Method: TEST.MS
Sample Name: TMG-25A
Analysis Info:

Instrument: LC-MSD-Trap-SL
Operator: Operator

Print Date: 3/25/2014 9:13:35 AM
Acq. Date: 3/25/2014 9:03:01 AM

Acquisition Parameter:

Mass Range Mode	Std/Normal	Trap Drive	36.3	Scan Begin	100 m/z
Ion Polarity	Positive	Octopole RF Amplitude	171.0 Vpp	Scan End	450 m/z
Ion Source Type	ESI	Capillary Exit	106.0 Volt	Averages	5 Spectra
Dry Temp (Set)	330 °C	Skimmer	40.0 Volt	Max. Accu Time	200000 µs
Nebulizer (Set)	15.00 psi	Oct 1 DC	12.00 Volt	ICC Target	20000
Dry Gas (Set)	6.00 l/min	Oct 2 DC	1.70 Volt	Charge Control	on

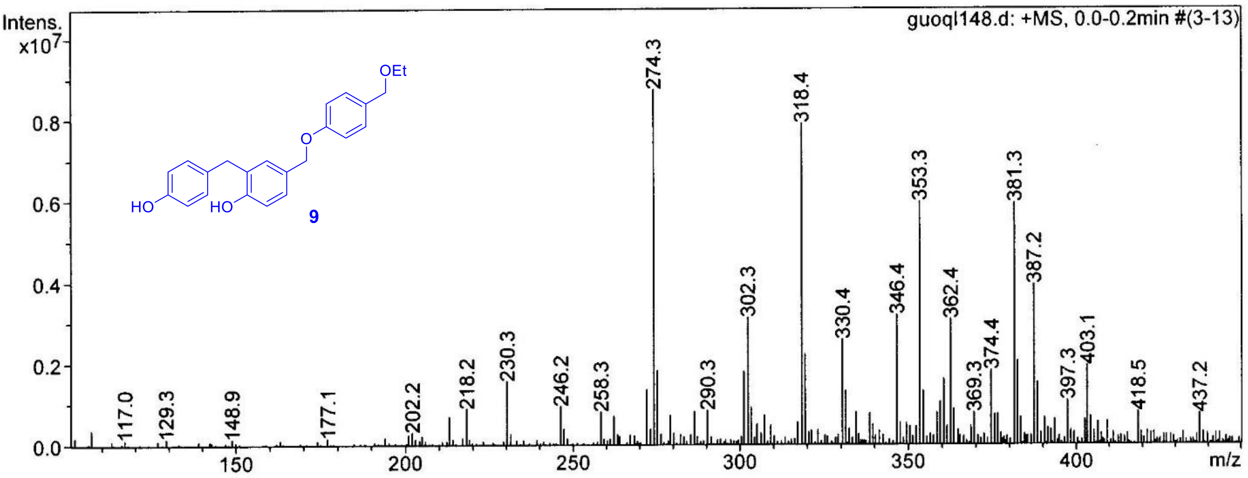

Component Molecular Mass Molecule Absolute Abundance Relative Abundance

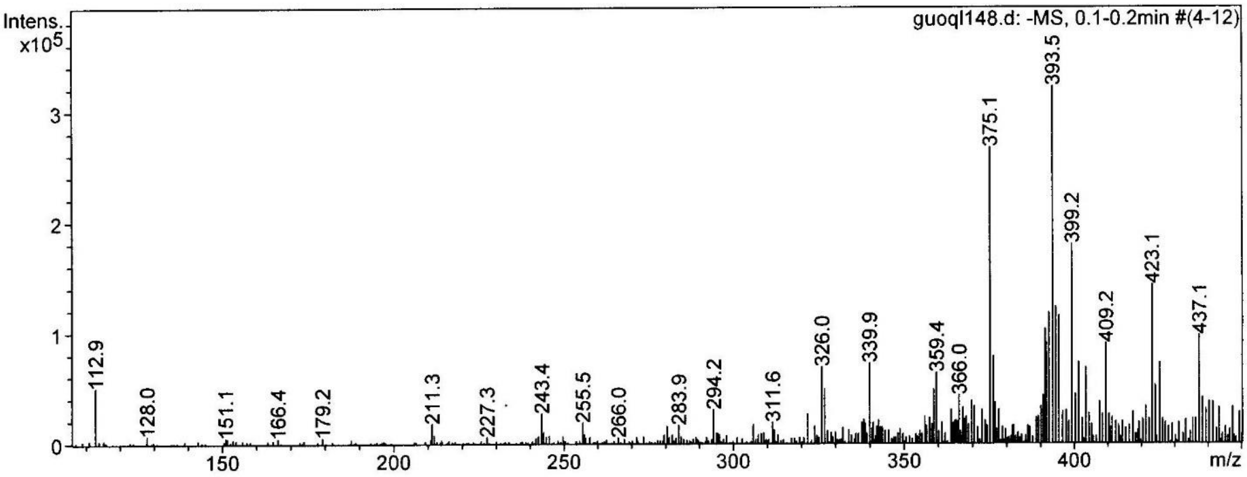

Component Molecular Mass Molecule Absolute Abundance Relative Abundance

Fig. S99 The ESI-MS of compound **9**.

Qualitative Analysis Report

**Data Filename**	2014022602.d	**Sample Name**	TMG-25A
**Sample Type**	Sample	**Position**	P1-C2
**Instrument Name**	Instrument 1	**User Name**	
**Acq Method**		**IRM Calibration Status**	Success
**DA Method**	TEST LCMS.m	**Comment**	

User Chromatograms

Fragmentor Voltage 135 **Collision Energy** 0 **Ionization Mode** ESI

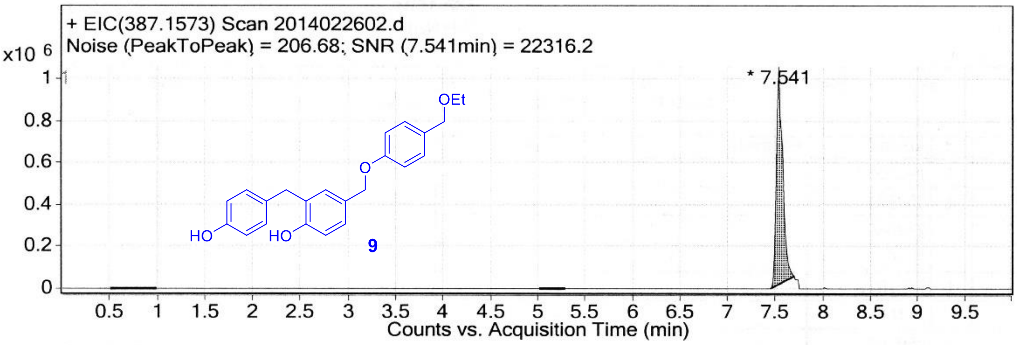

Integration Peak List

Peak	Start	RT	End	Height	Area	Area %	Signal To Noise
1	7.444	7.541	7.702	1036189	4612284	100	22316.2

Noise Measurements

Noise Type	Signal Definition	Noise Multiplier	Noise Value
Peak-to-Peak	Area	1	206.6786194

Noise Regions

Start	End
0.5	1
5	5.3
9.99	11

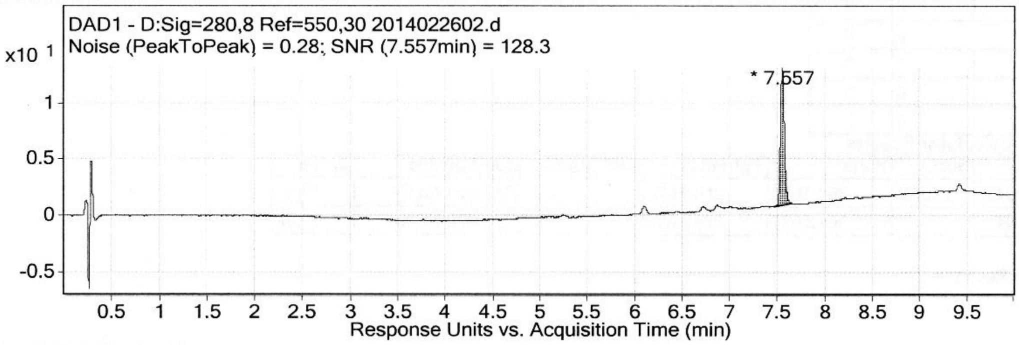

Integration Peak List

Peak	Start	RT	End	Height	Area	Area %	Signal To Noise
1	7.463	7.557	7.651	12.21	35.56	100	128.3

Noise Measurements

Noise Type	Signal Definition	Noise Multiplier	Noise Value
Peak-to-Peak	Area	1	0.277042389

Noise Regions

Start	End
0.5	1
5	5.3
9.99	11

User Spectra

Fig. S100 The (+)-HR-ESI-MS report of compound **9**, Page1.

Qualitative Analysis Report

Fragmentor Voltage	Collision Energy	Ionization Mode
135	0	Esi

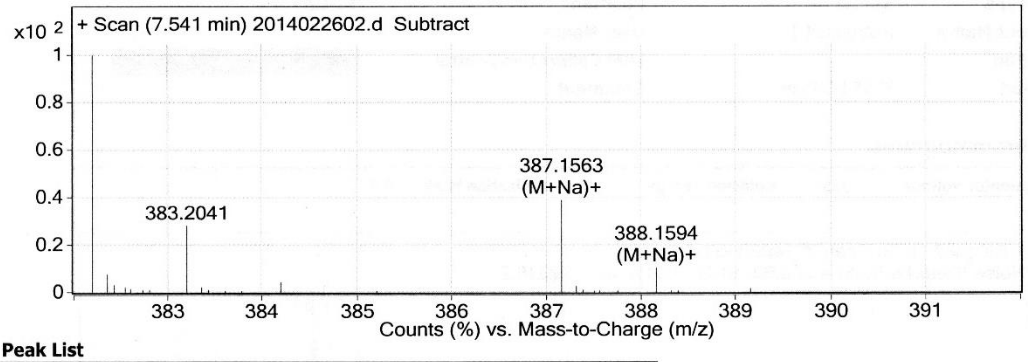

Peak List

m/z	z	Abund	Formula	Ion
213.09	1	1937087		
214.0938	1	309974		
230.247		238660		
319.1324		239551		
382.2001	1	2727817		
382.3661		211665		
383.2041	1	769706		
387.1563	1	1058013	C23 H24 Na O4	(M+Na)+
388.1594	1	285566	C23 H24 Na O4	(M+Na)+
396.2163		512742		

Formula Calculator Element Limits

Element	Min	Max
C	3	100
H	0	500
O	0	90
N	0	5
S	0	5
Cl	0	2
Br	0	0
Si	0	0
F	0	0
P	0	0

Formula Calculator Results

Formula	Best	Mass	Tgt Mass	Diff (ppm)	Ion Species	Score
C23 H24 O4	TRUE	364.1671	364.1675	0.98	C23 H24 Na O4	99.91
C24 H20 N4		364.1671	364.1688	4.63	C24 H20 N4 Na	99.68

--- End Of Report ---

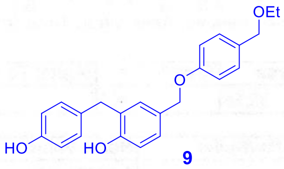

9

Fig. S101 The (+)-HR-ESI-MS report of compound **9**, Page2.

MS Formula Results: + Scan (7.541 min) Sub (2014022602.d)

m/z	Ion	Formula	Abundance
387.1563	(M+Na)+	C23 H24 Na O4	1058013.4

Best	Formula (M)	Ion Formula	Calc m/z	Score	Cross S	Mass	Calc Mass	Diff (ppm)	Abs Diff (ppm)	Abund Match	Spacing Mat	Mass Match	m/z	DBE
☑	C23 H24 O4	C23 H24 Na O4	387.1567	99.91		364.1671	364.1675	0.98	0.98	99.75	99.96	99.97	387.1563	12
☐	C24 H20 N4	C24 H20 N4 Na	387.158	99.68		364.1671	364.1688	4.63	4.63	99.92	99.99	99.38	387.1563	17

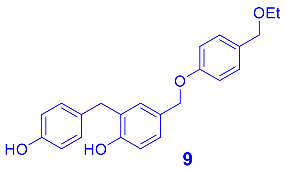

9

page 1

Fig. S102 The (+)-HR-ESI-MS report of compound **9**, page 3.

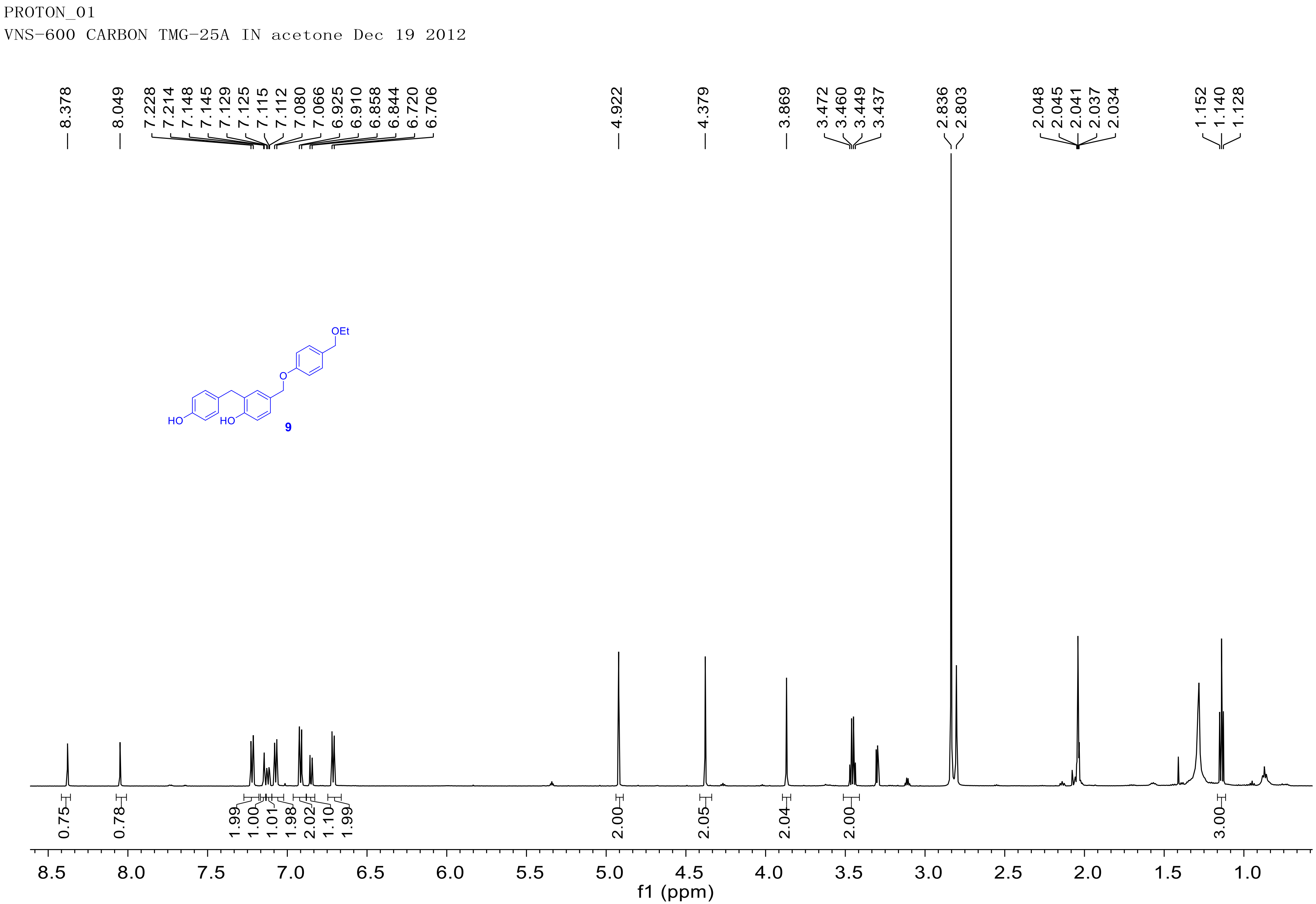

Fig. S103 The ^{1}H NMR spectrum of compound **9** in acetone-d_6 (600 MHz).

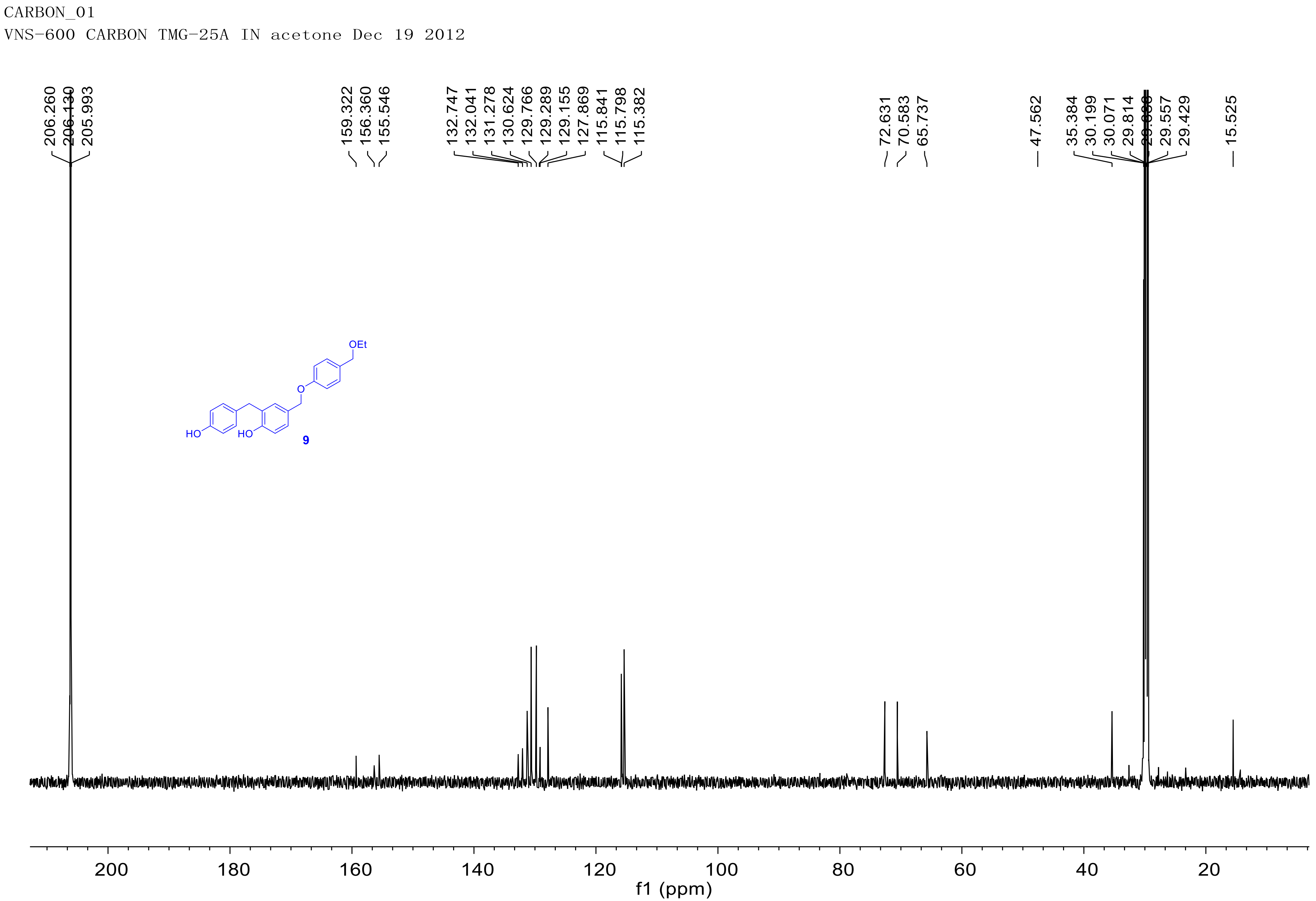

Fig. S104 The ^{13}C NMR spectrum of compound **9** in acetone-d_6 (150 MHz).

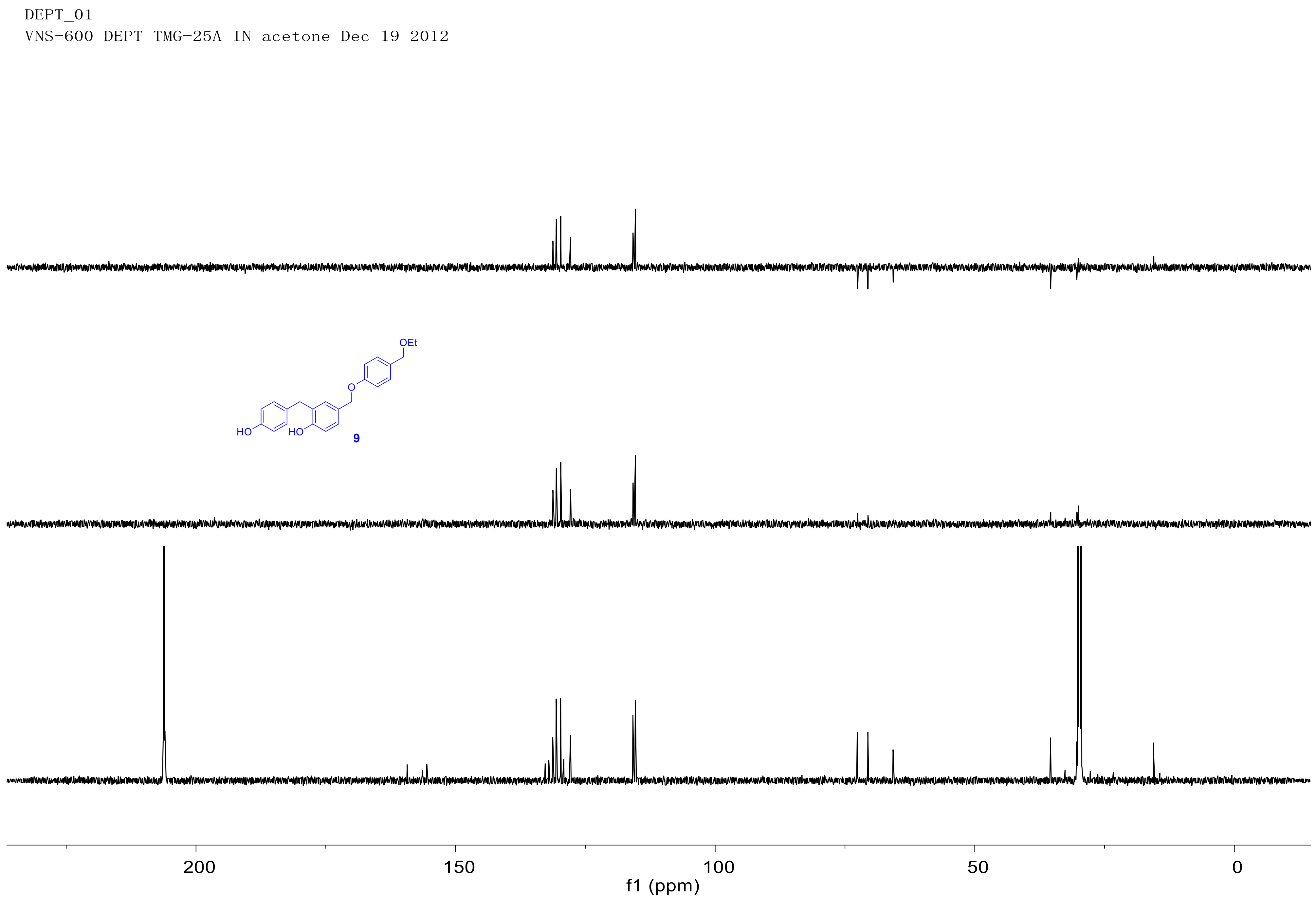

Fig. S105 The DEPT spectrum of compound **9** in acetone-d_6 (150 MHz).

gCOSY_01
VNS-600 CARBON TMG-25A IN acetone Dec 19 2012

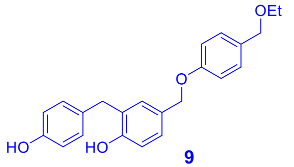

9

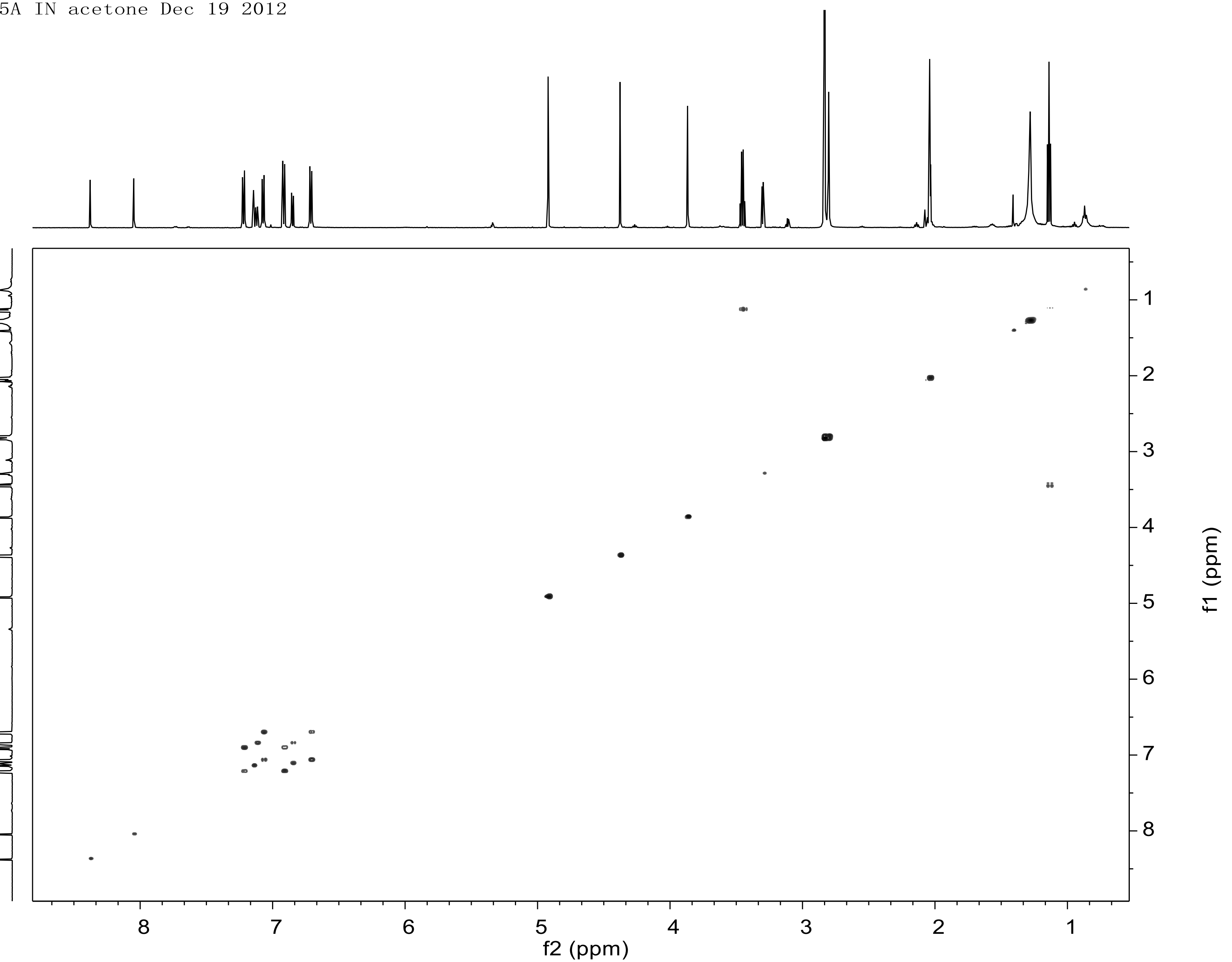

Fig. S106 The ^{1}H-^{1}H COSY spectrum of compound **9** in acetone-d_6 (600 MHz).

gHSQCAD_01
VNS-600 CARBON TMG-25A IN acetone Dec 19 2012

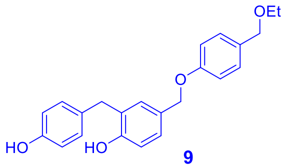

9

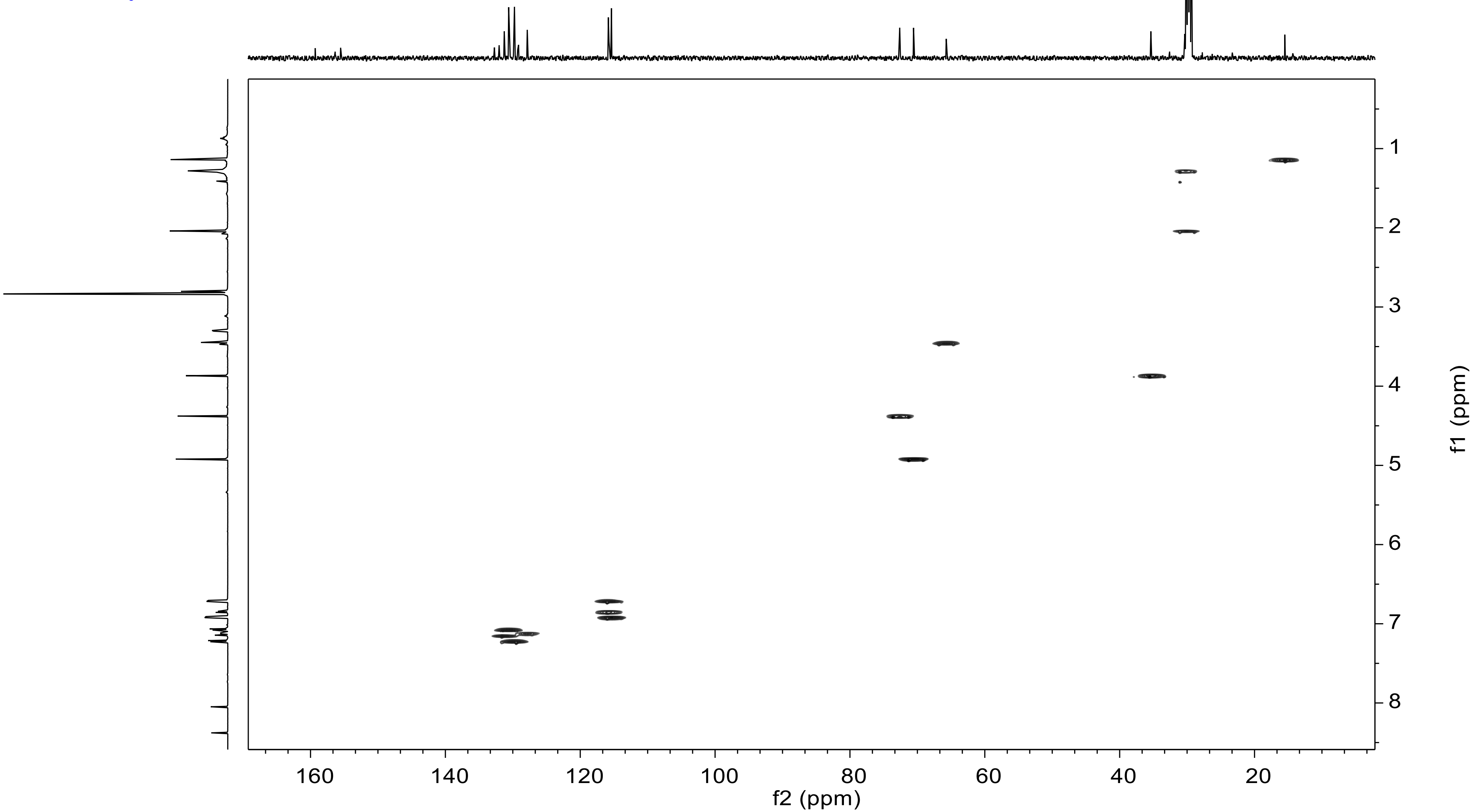

Fig. S107 The HSQC spectrum of compound **9** in acetone-d_6 (600 MHz for ^{1}H).

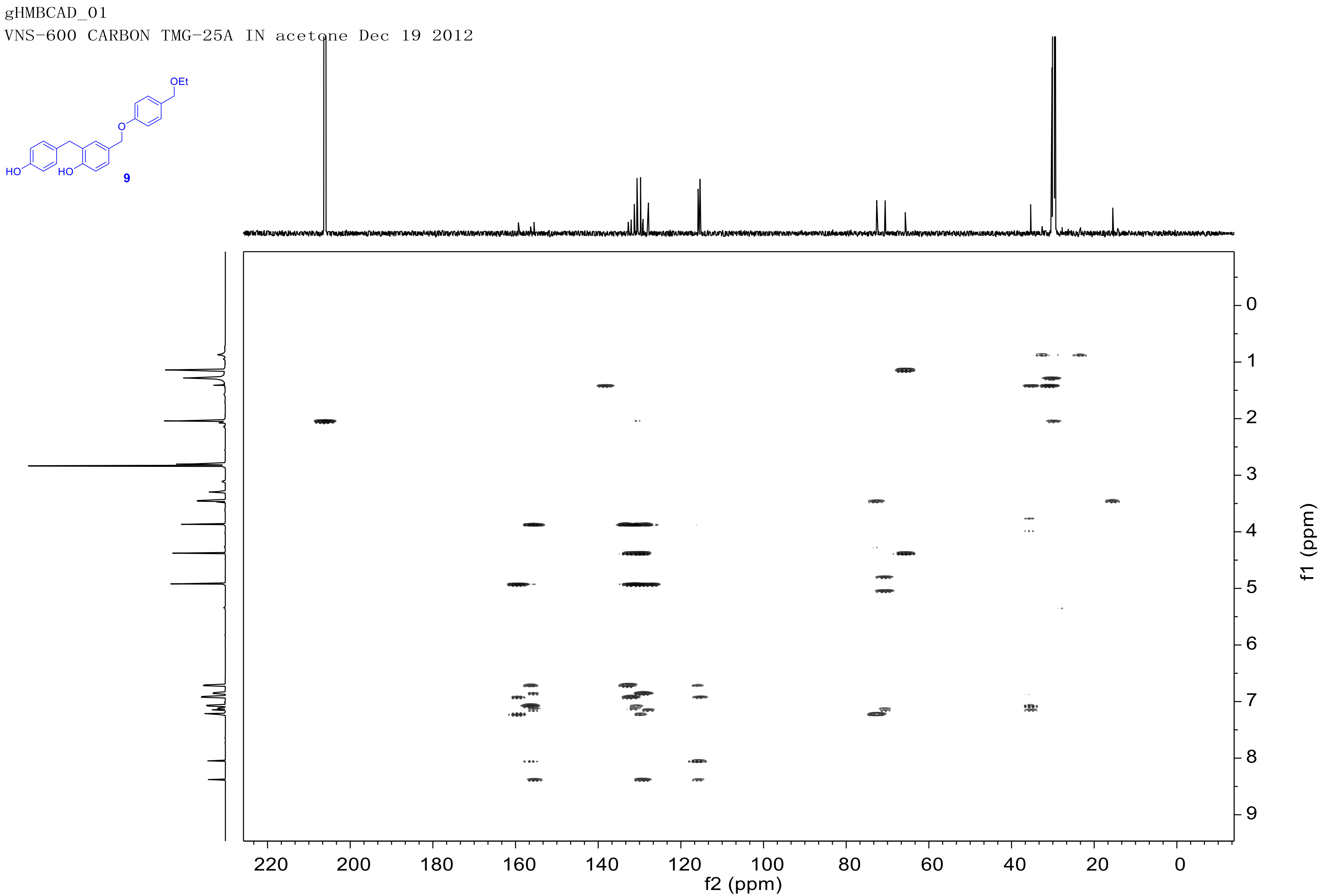

Fig. S108 The HMBC spectrum of compound **9** in acetone-d_6 (600 MHz for ^{1}H).

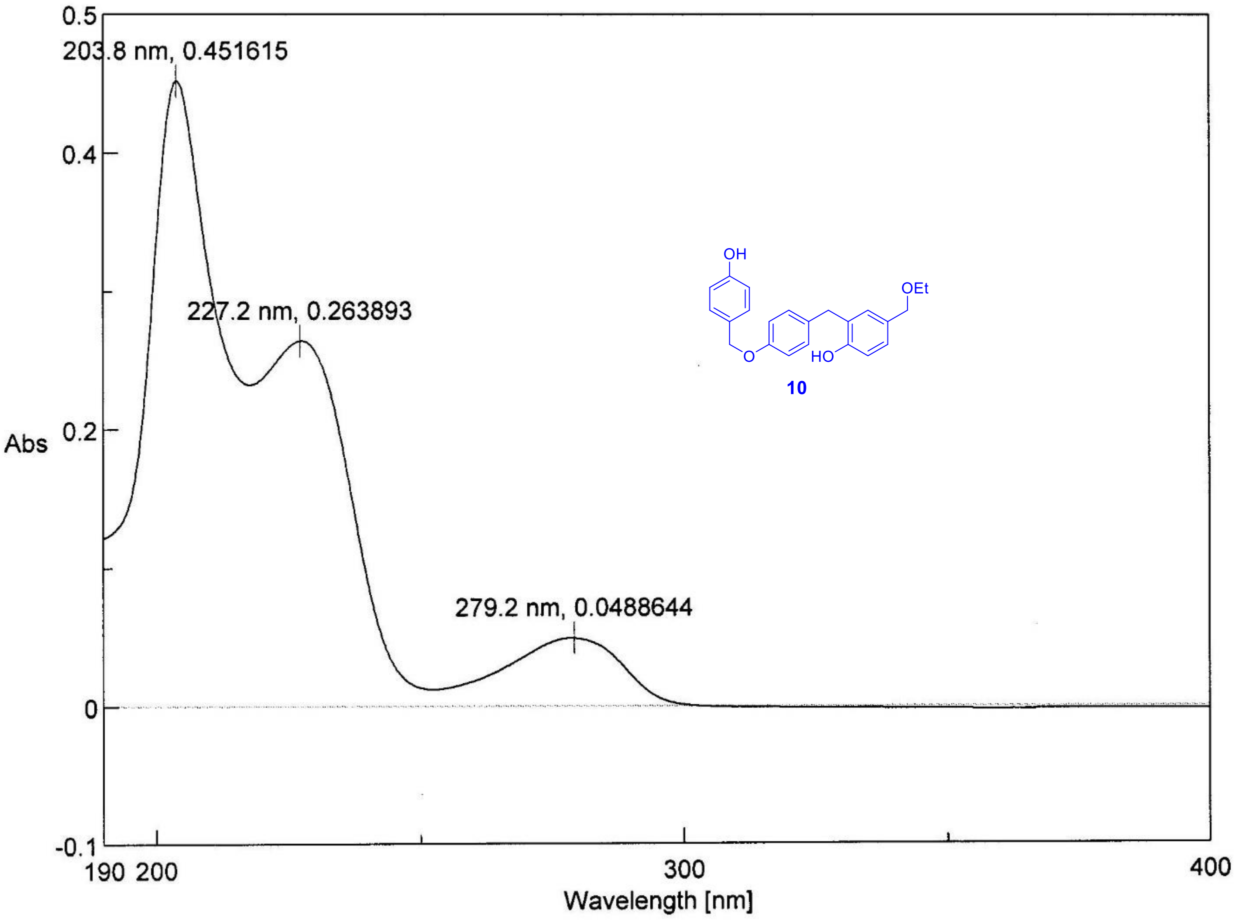

[Comment]

TMG-25B

Sample Name	TMG-25B
Comment	0.02
User	
Division	UV
Company	324

[Measurement Information]

Instrument Name	V-650
Model Name	V-650
Serial No.	A034461150
Accessory	PSC-718
Accessory S/N	A001761114
Position	1
Cell Length	10 mm
Temperature	19.98 C
Control Sonsor	Holder
Monitor Sensor	Holder
Start Mode	Start immediately
Photometric Mode	Abs
Measurement range	400 - 190 nm
Data pitch	0.2 nm
Band width(UV/Vis)	2.0 nm
Response	Medium
Scanning speed	200 nm/min
Source Change	340 nm
Light Source	D2/WI
Filter Exchange	Step
Correction	Baseline

[Data Information]

Creation Date	2014-2-26 10:11
Data array type	Linear data array
Horizontal	Wavelength [nm]
Vertical	Abs
Start	400 nm
End	190 nm
Data pitch	0.2 nm
Data points	1051

Fig. S109 The UV spectrum of compound **10**.

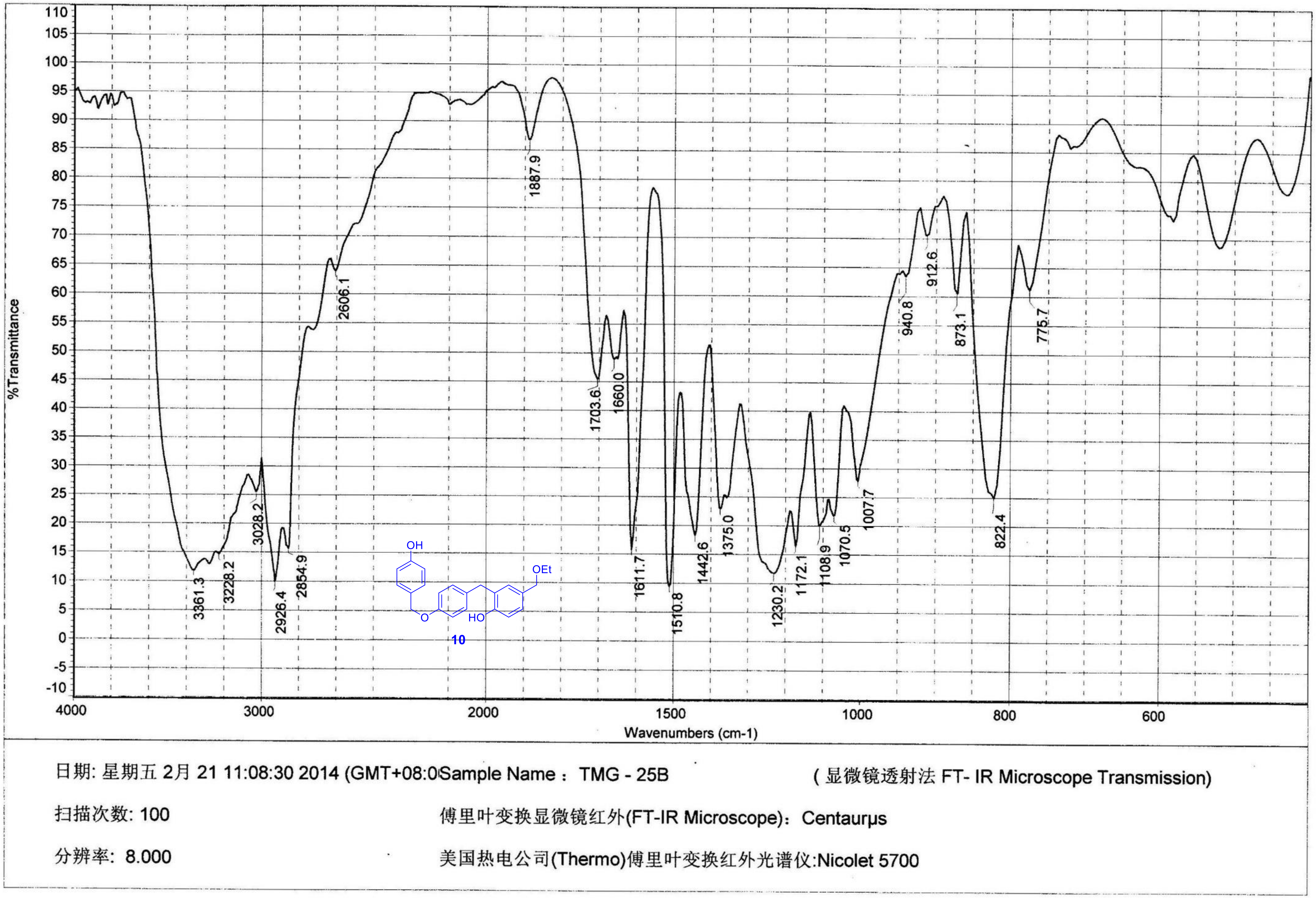

Fig. S110 The IR spectrum of compound **10**.

Single Mass Spectrum Deconvolution Report

Analysis Name: guoql149.d **Instrument:** LC-MSD-Trap-SL **Print Date:** 3/25/2014 9:15:04 AM
Method: TEST.MS **Operator:** Operator **Acq. Date:** 3/25/2014 9:05:21 AM
Sample Name: TMG-25B
Analysis Info:

Acquisition Parameter:

Mass Range Mode	Std/Normal	Trap Drive	36.3	Scan Begin	100 m/z
Ion Polarity	Positive	Octopole RF Amplitude	171.0 Vpp	Scan End	450 m/z
Ion Source Type	ESI	Capillary Exit	106.0 Volt	Averages	5 Spectra
Dry Temp (Set)	330 °C	Skimmer	40.0 Volt	Max. Accu Time	200000 µs
Nebulizer (Set)	15.00 psi	Oct 1 DC	12.00 Volt	ICC Target	20000
Dry Gas (Set)	6.00 l/min	Oct 2 DC	1.70 Volt	Charge Control	on

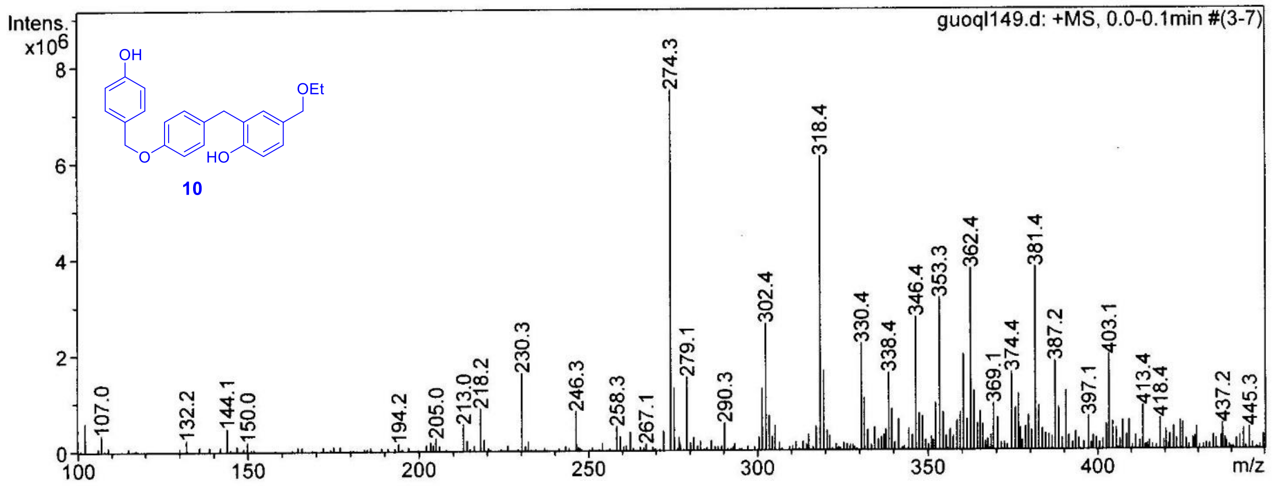

Component | Molecular Mass | Molecule | Absolute Abundance | Relative Abundance

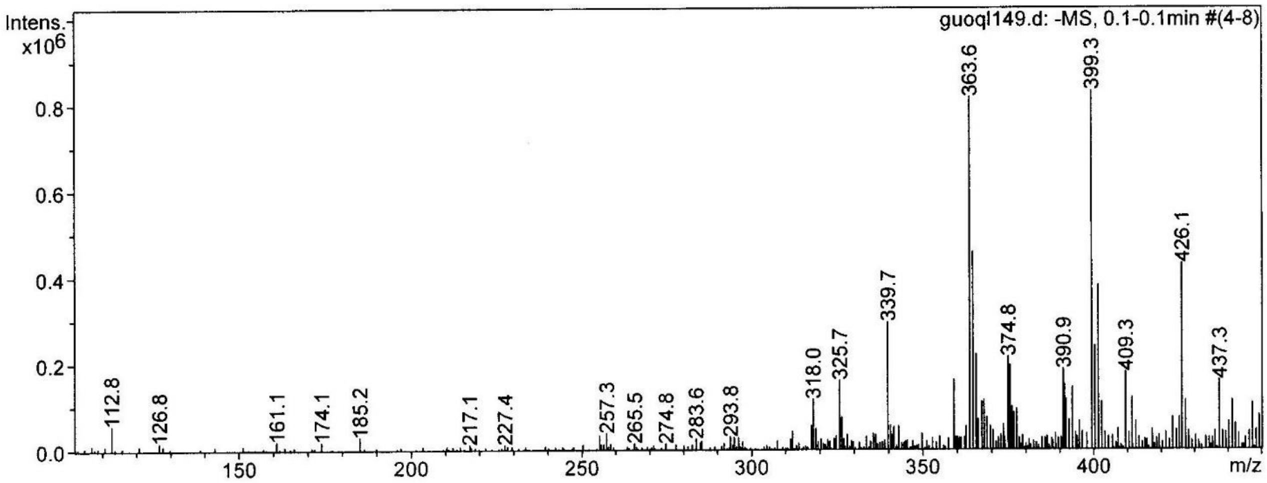

Component | Molecular Mass | Molecule | Absolute Abundance | Relative Abundance

Fig. S111 The ESI-MS of compound **10**.

Qualitative Analysis Report

**Data Filename**	2014022603.d	**Sample Name**	TMG-25B
**Sample Type**	Sample	**Position**	P1-C3
**Instrument Name**	Instrument 1	**User Name**	
**Acq Method**		**IRM Calibration Status**	Success
**DA Method**	TEST LCMS.m	**Comment**	

User Chromatograms

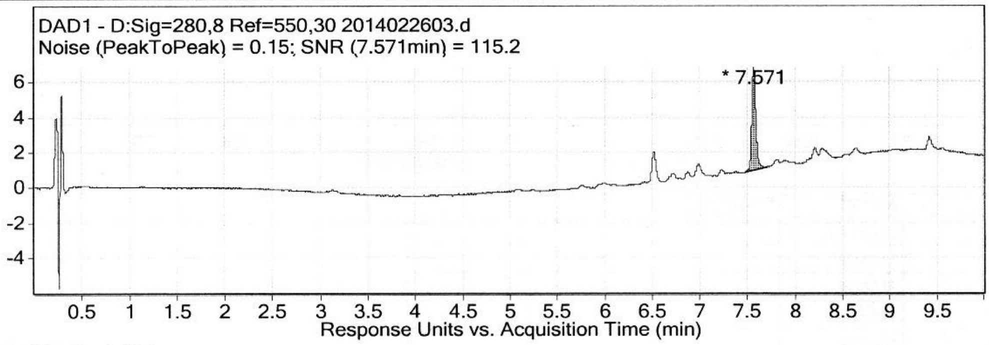

Integration Peak List

Peak	Start	RT	End	Height	Area	Area %	Signal To Noise
1	7.482	7.571	7.713	5.85	17.69	100	115.2

Noise Measurements

Noise Type	Signal Definition	Noise Multiplier	Noise Value
Peak-to-Peak	Area	1	0.153541565

Noise Regions

Start	End
0.5	1
5	5.3
9.99	11

Fragmentor Voltage 135 **Collision Energy** 0 **Ionization Mode** ESI

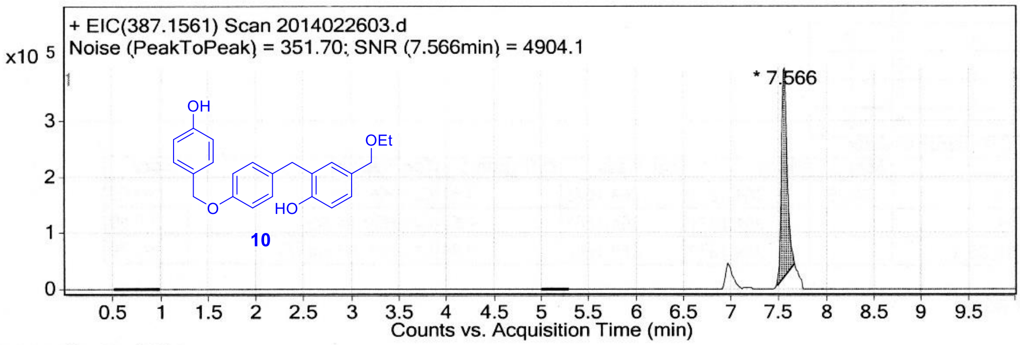

Integration Peak List

Peak	Start	RT	End	Height	Area	Area %	Signal To Noise
1	7.485	7.566	7.662	371567	1724778	100	4904.1

Noise Measurements

Noise Type	Signal Definition	Noise Multiplier	Noise Value
Peak-to-Peak	Area	1	351.7009888

Noise Regions

Start	End
0.5	1
5	5.3
9.99	11

User Spectra

Agilent Technologies   Page 1 of 2   Printed at: 8:56 AM on: 2/26/2014

Fig. S112 The (+)-HR-ESI-MS report of compound **10**, Page1.

Qualitative Analysis Report

Fragmentor Voltage	Collision Energy	Ionization Mode
135	0	Esi

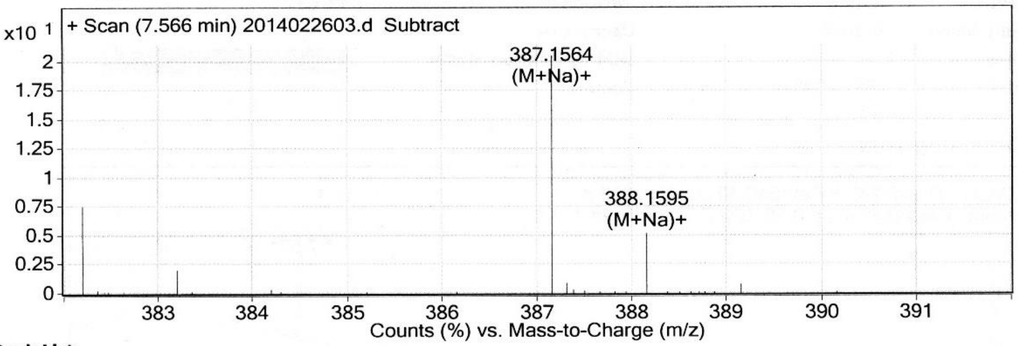

Peak List

m/z	z	Abund	Formula	Ion
213.0902	1	1920478		
213.2151		132508		
214.0938	1	316654		
230.2472		105284		
274.2733		144339		
319.1319		246041		
382.2		144314		
387.1564	1	394118	C23 H24 Na O4	(M+Na)+
396.216	1	474116		
397.2192	1	132201		

Formula Calculator Element Limits

Element	Min	Max
C	3	100
H	0	500
O	0	90
N	0	5
S	0	5
Cl	0	2
Br	0	0
Si	0	0
F	0	0
P	0	0

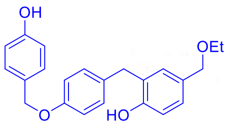

10

Formula Calculator Results

Formula	Best	Mass	Tgt Mass	Diff (ppm)	Ion Species	Score
C23 H24 O4	TRUE	364.1671	364.1675	0.87	C23 H24 Na O4	99.98
C24 H20 N4		364.1671	364.1688	4.52	C24 H20 N4 Na	99.59
C15 H28 N2 O6 S		364.1672	364.1668	-0.94	C15 H28 N2 Na O6 S	97.39

--- End Of Report ---

Fig. S113 The (+)-HR-ESI-MS report of compound **10**, Page2.

MS Formula Results: + Scan (7.566 min) Sub (2014022603.d)

m/z	Ion	Formula	Abundance
387.1564	(M+Na)+	C23 H24 Na O4	394117.9

Best	Formula (M)	Ion Formula	Calc m/z	Score	Cross S	Mass	Calc Mass	Diff (ppm)	Abs Diff (ppm)	Abund Match	Spacing Mat	Mass Match	m/z	DBE
☑	C23 H24 O4	C23 H24 Na O4	387.1567	99.98		364.1671	364.1675	0.87	0.87	99.99	99.98	99.98	387.1564	12
☐	C24 H20 N4	C24 H20 N4 Na	387.158	99.59		364.1671	364.1688	4.52	4.52	99.56	99.99	99.41	387.1564	17
☐	C15 H28 N2 O6 S	C15 H28 N2 Na O6 S	387.156	97.39		364.1672	364.1668	-0.94	0.94	91.47	99.32	99.97	387.1564	3

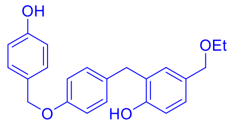

10

page 1

Fig. S114 The (+)-HR-ESI-MS report of compound **10**, page 3.

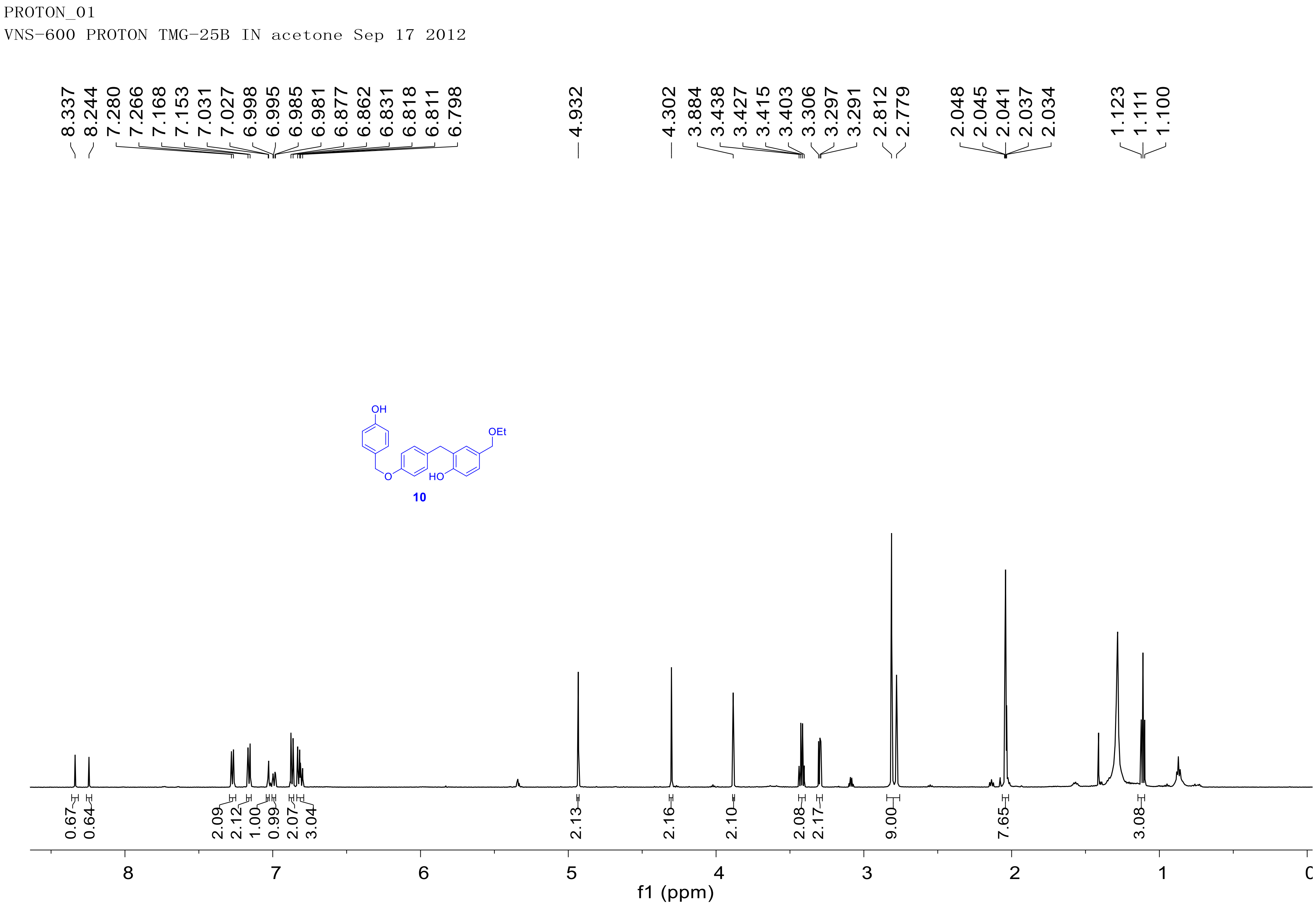

Fig. S115 The ^{1}H NMR spectrum of compound **10** in acetone-d_6 (600 MHz).

CARBON_01
VNS-600 CARBON TMG-25B IN acetone Dec 19 2012

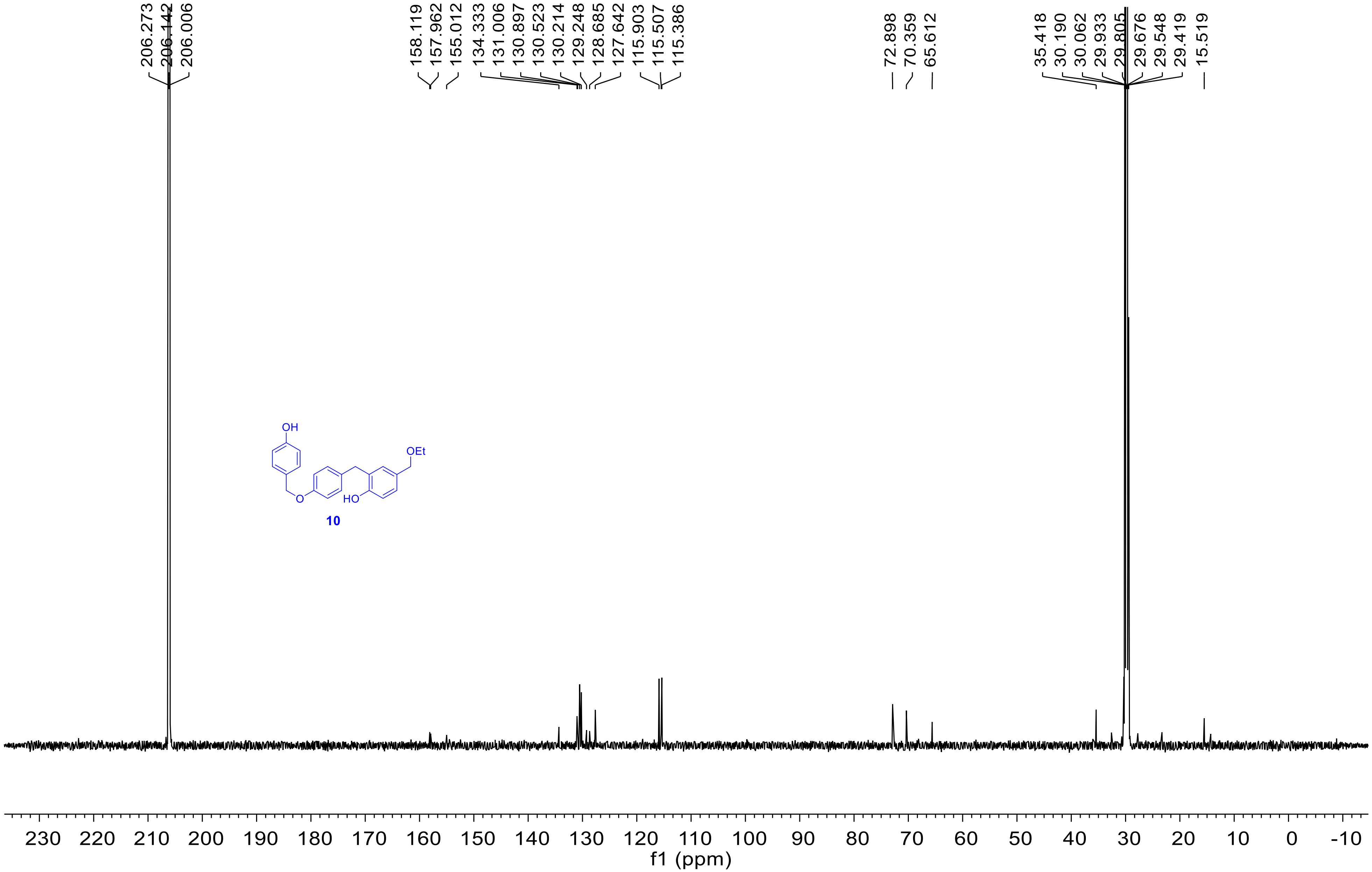

10

Fig. S116 The ^{13}C NMR spectrum of compound **10** in acetone-d_6 (150 MHz).

DEPT_01
VNS-600 DEPT TMG-25B IN acetone Dec 19 2012

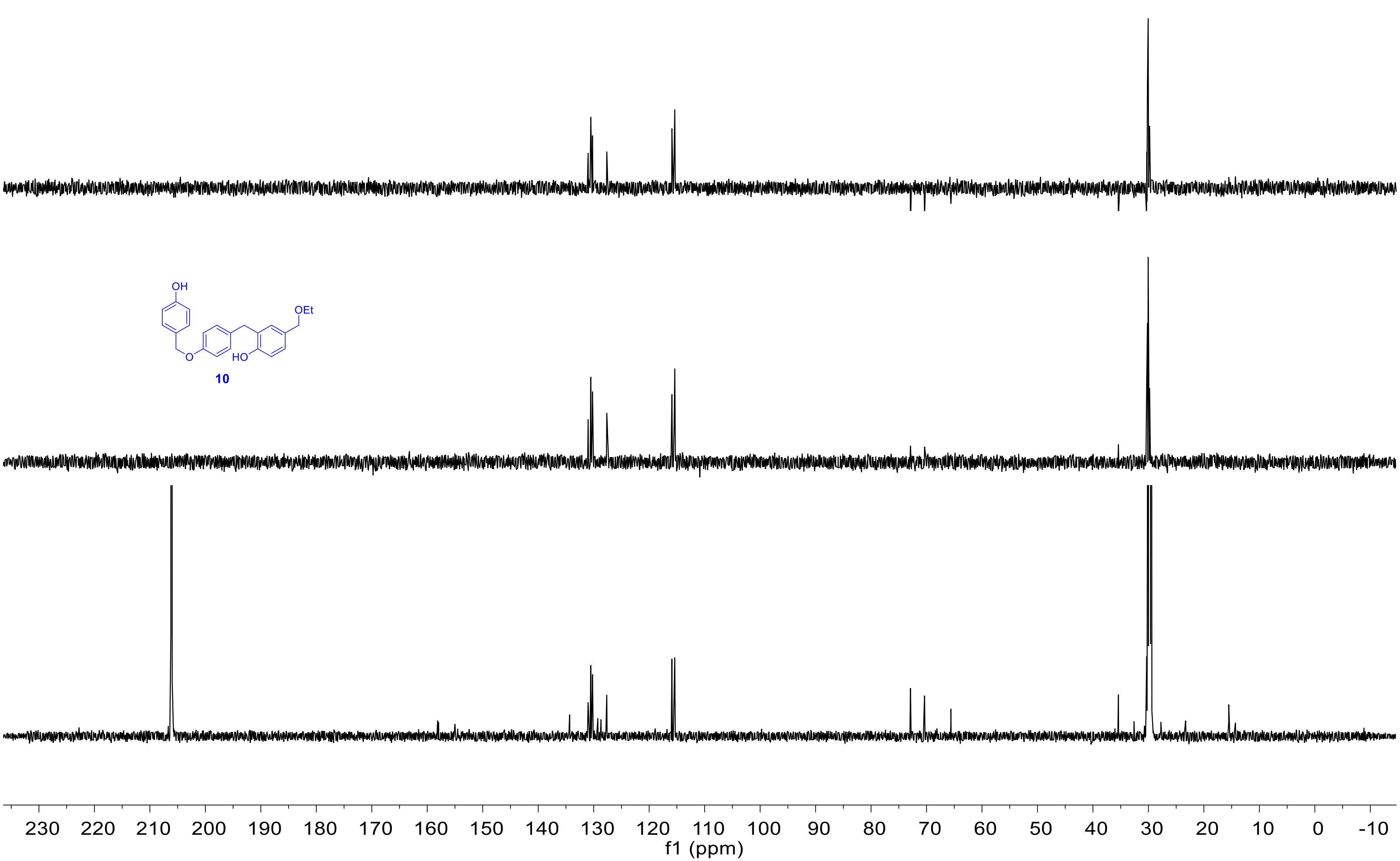

Fig. S117 The DEPT spectrum of compound **10** in acetone-d_6 (150 MHz).

gCOSY_01
VNS-600 gCOSY TMG-25B IN acetone Dec 19 2012

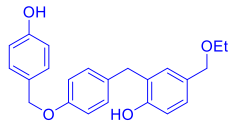

10

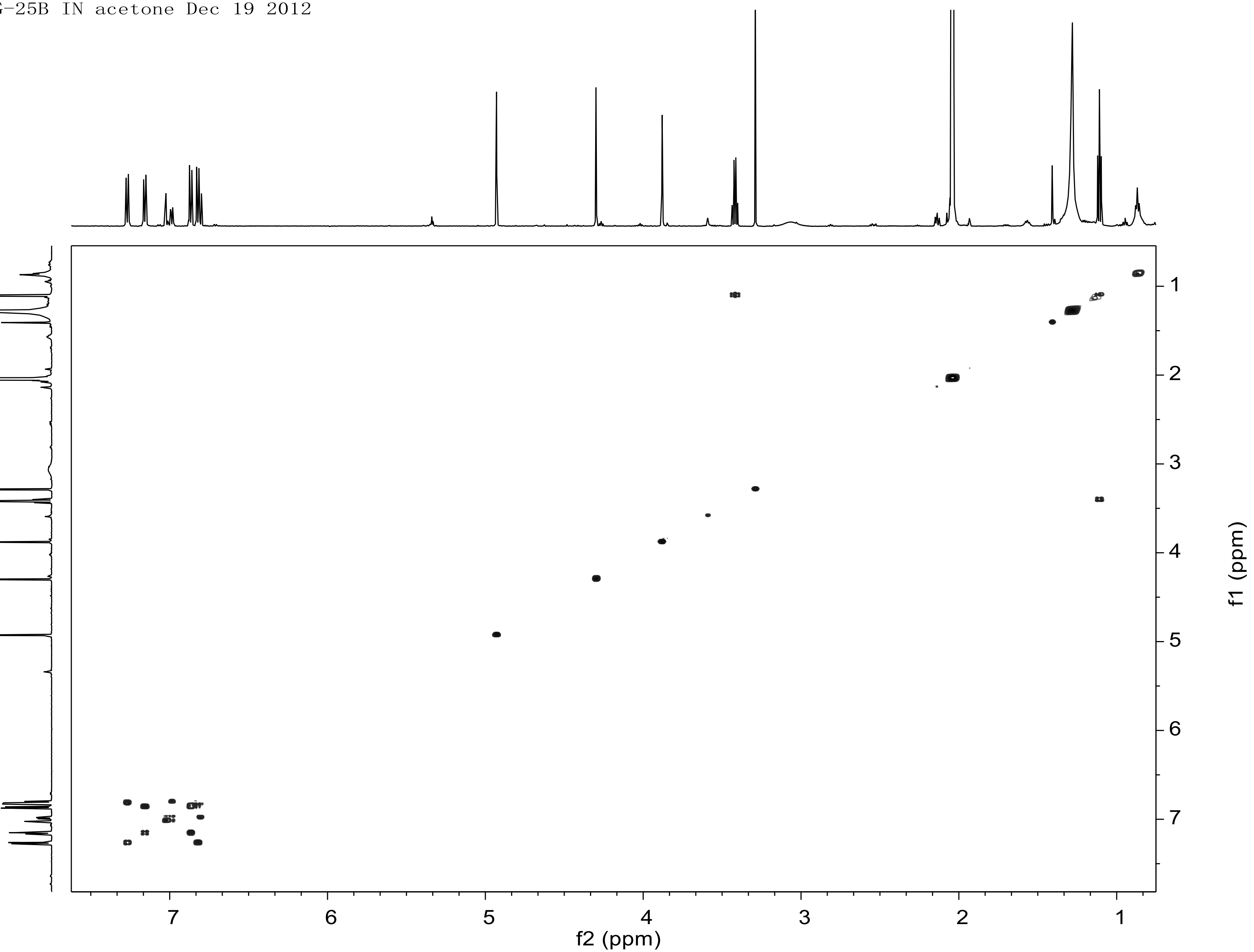

Fig. S118 The ^{1}H-^{1}H COSY spectrum of compound **10** in acetone-d_6 (600 MHz).

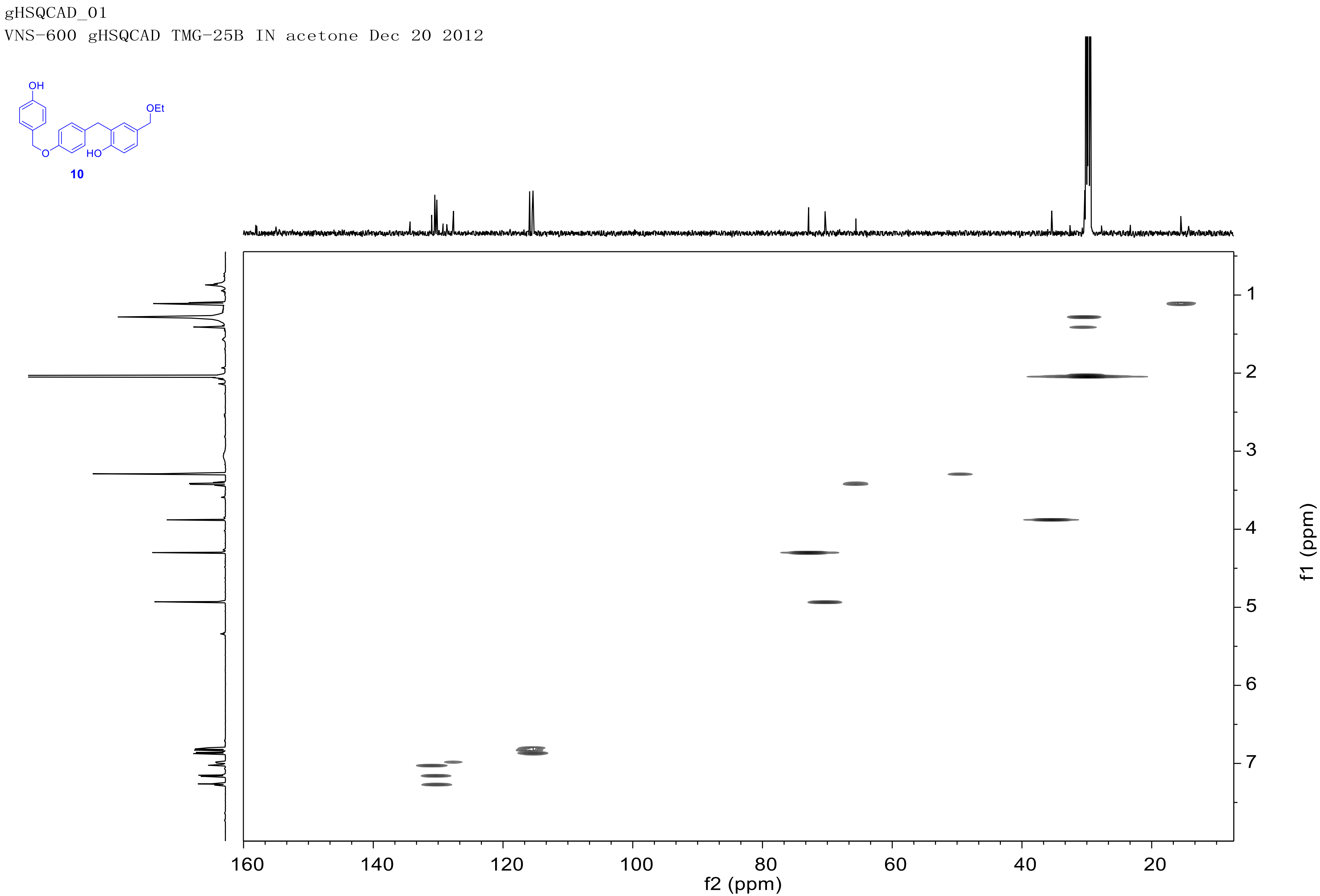

Fig. S119 The HSQC spectrum of compound **10** in acetone-d_6 (600 MHz for ^{1}H).

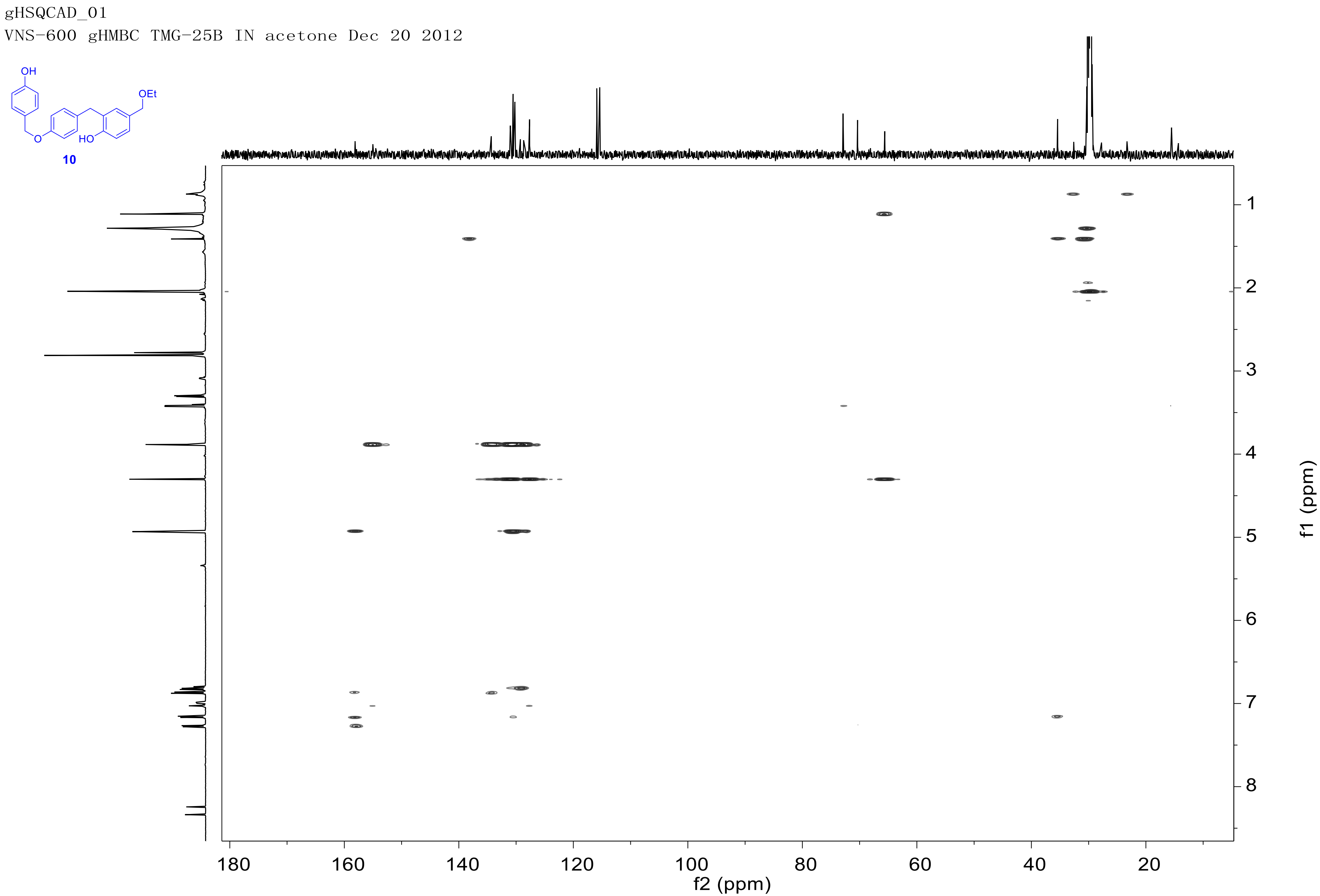

Fig. S120 The HMBC spectrum of compound **10** in acetone-d_6 (600 MHz for ^{1}H).

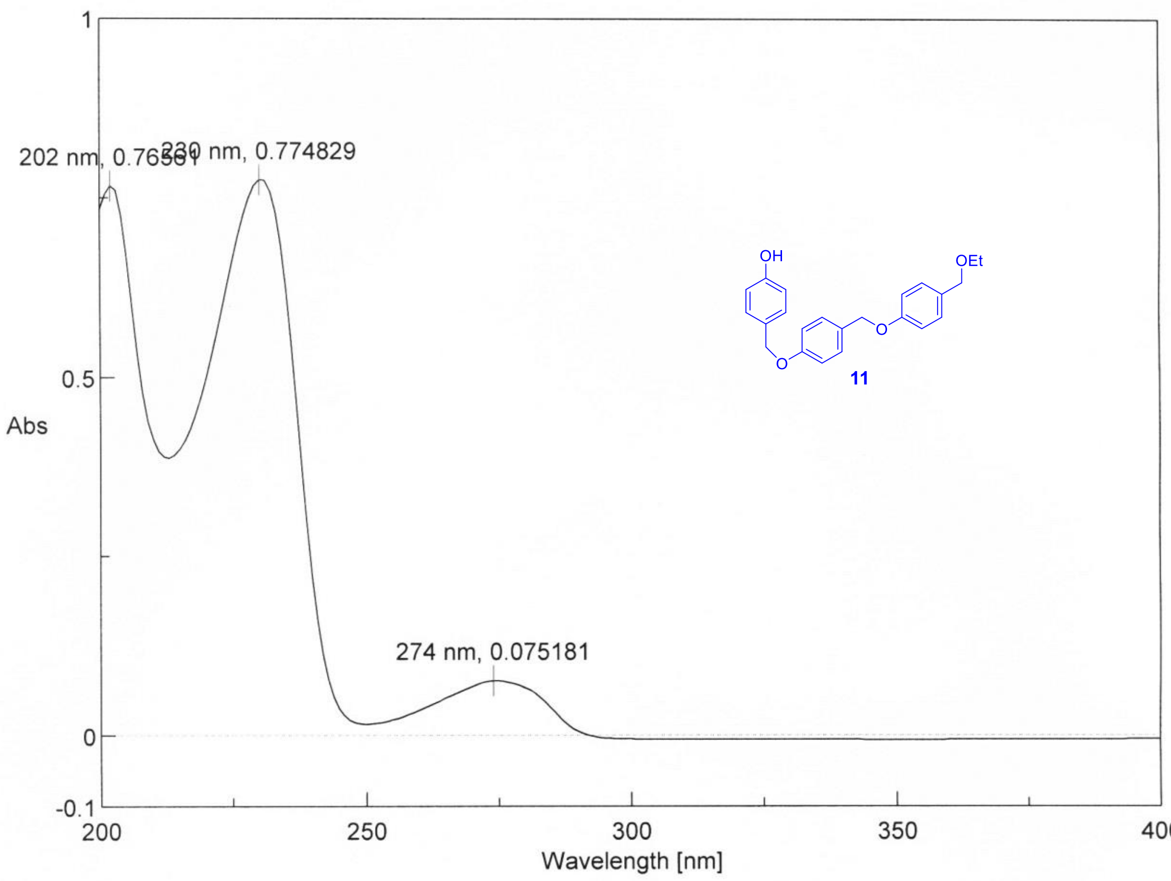

wyn-110-MeOH-2-SV

[Comment]
Sample Name wyn-110
Comment MeOH
User 王亚男
Division UV
Company 324

[Measurement Information]
Instrument Name V-650
Model Name V-650
Serial No. A034461150

Accessory PSC-718
Accessory S/N A001761114
Position 1
Cell Length 10 mm
Temperature 19.99 C
Control Sonsor Holder
Monitor Sensor Holder
Start Mode Start immediately

Photometric Mode Abs
Measurement range 400 - 200 nm
Data pitch 1 nm
Band width(UV/Vis) 1.0 nm
Response Medium
Scanning speed 200 nm/min
Source Change 340 nm
Light Source D2/WI
Filter Exchange Step
Correction Baseline

[Data Information]
Creation Date 2012-5-16 8:45

Data array type Linear data array
Horizontal Wavelength [nm]
Vertical Abs
Start 400 nm
End 200 nm
Data pitch 1 nm
Data points 201

Fig. S121 The UV spectrum of compound **11**.

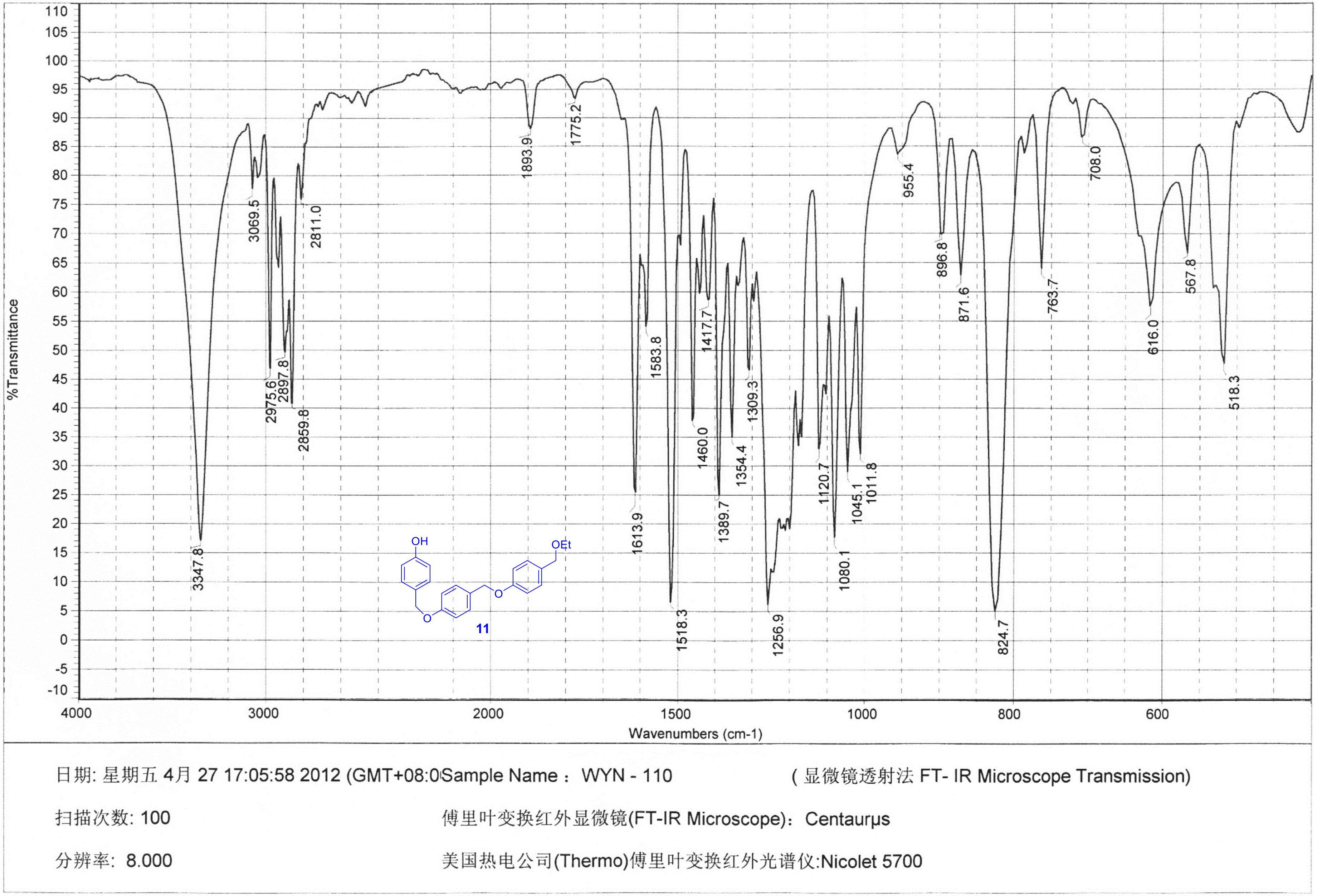

Fig. S122 The IR spectrum of compound **11**.

Single Mass Spectrum Deconvolution Report

Analysis Name: wangy104.d **Instrument:** LC-MSD-Trap-SL **Print Date:** 7/20/2011 7:59:44 AM
Method: TEST.MS **Operator:** Operator **Acq. Date:** 7/20/2011 7:41:13 AM
Sample Name: WYN-110
Analysis Info:

Acquisition Parameter:

Mass Range Mode	Std/Normal	Trap Drive	29.0	Scan Begin	100 m/z
Ion Polarity	Positive	Octopole RF Amplitude	152.8 Vpp	Scan End	500 m/z
Ion Source Type	ESI	Capillary Exit	102.3 Volt	Averages	5 Spectra
Dry Temp (Set)	330 °C	Skimmer	40.0 Volt	Max. Accu Time	200000 µs
Nebulizer (Set)	15.00 psi	Oct 1 DC	12.00 Volt	ICC Target	20000
Dry Gas (Set)	5.00 l/min	Oct 2 DC	1.70 Volt	Charge Control	on

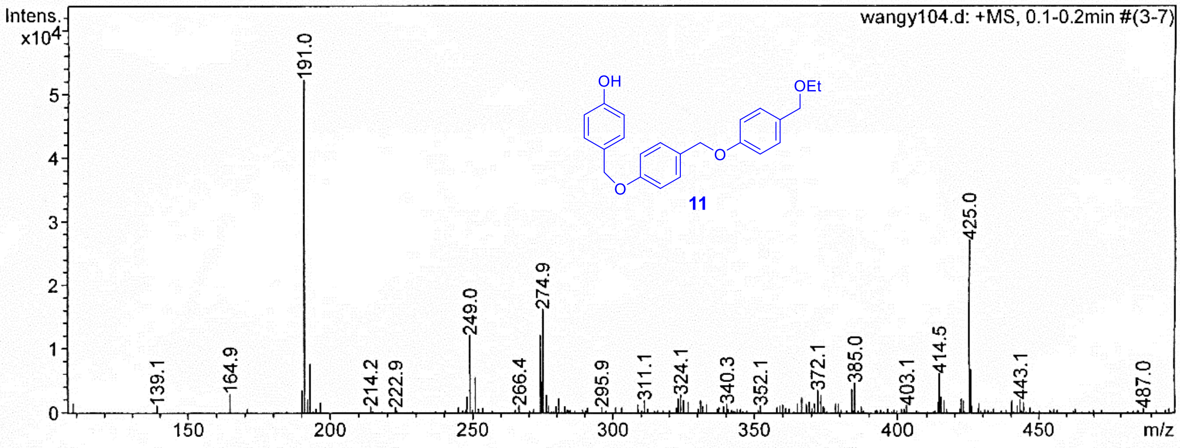

Component	Molecular Mass	Molecule	Absolute Abundance	Relative Abundance

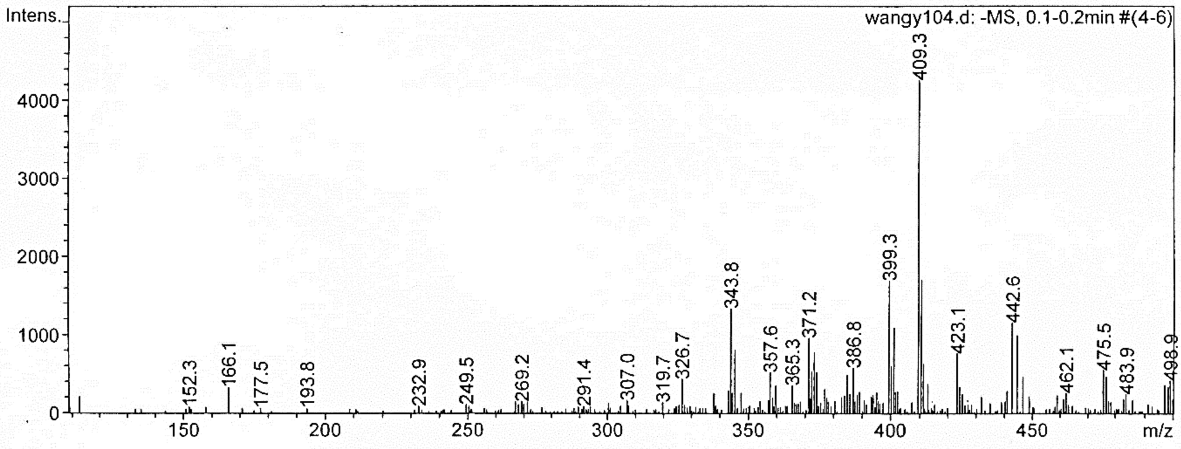

Component	Molecular Mass	Molecule	Absolute Abundance	Relative Abundance

Fig. S123 The ESI-MS of compound **11**.

Qualitative Analysis Report

**Data Filename**	2012041901.d	**Sample Name**	WYN-110
**Sample Type**	Sample	**Position**	P1-D5
**Instrument Name**	Instrument 1	**User Name**	
**Acq Method**		**IRM Calibration Status**	Success
**DA Method**	TEST LCMS.m	**Comment**	

User Chromatograms

Fragmentor Voltage 135 **Collision Energy** 0 **Ionization Mode** ESI

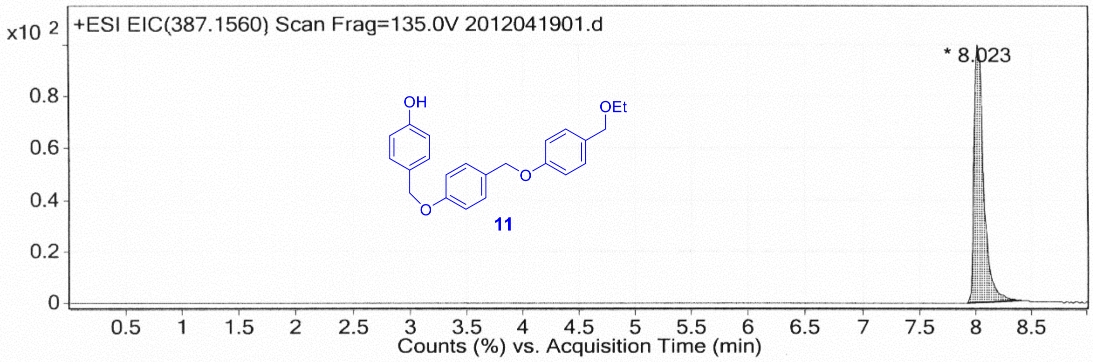

Integration Peak List

Peak	Start	RT	End	Height	Area	Area %
1	7.926	8.023	8.409	1654364	10264210	100

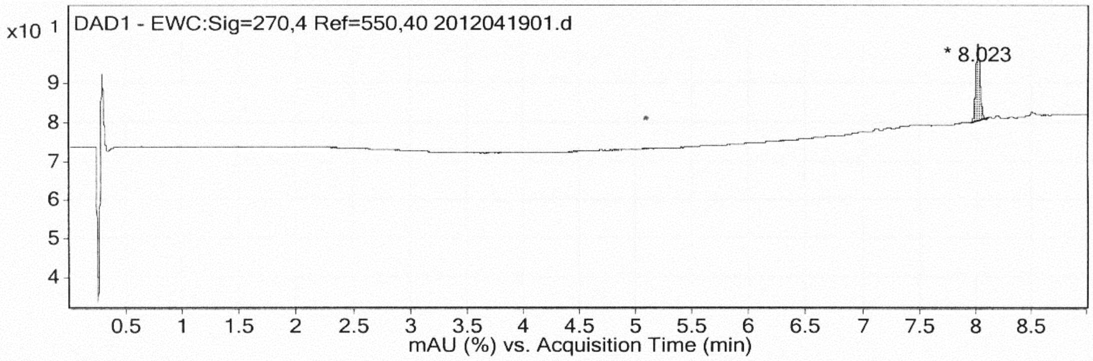

Integration Peak List

Peak	Start	RT	End	Height	Area	Area %
1	7.957	8.023	8.127	9.37	25.966	100

User Spectra

**Fragmentor Voltage**	**Collision Energy**	**Ionization Mode**
135	0	Esi

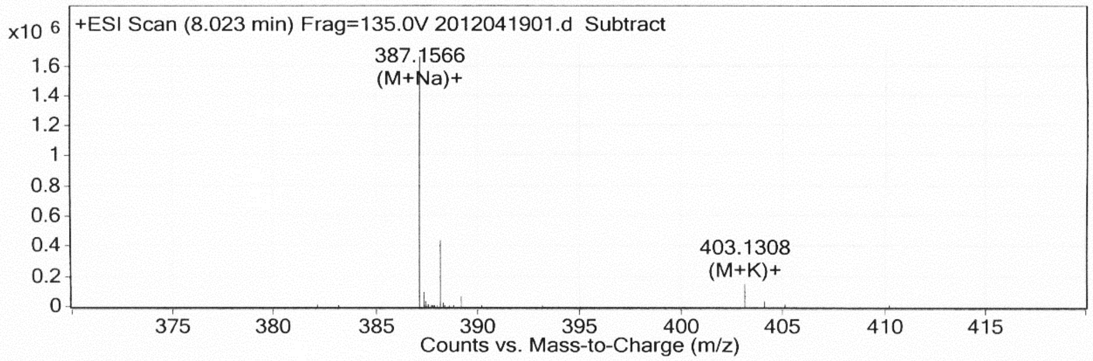

Fig. S124 The (+)-HR-ESI-MS report of compound **11**, page 1.

Qualitative Analysis Report

Peak List

m/z	z	Abund	Formula	Ion
107.0452		839087		
213.0906		110234		
387.1566	1	1656432	C23 H24 Na O4	(M+Na)+
387.3234		100571		
388.1606	1	439581	C23 H24 Na O4	(M+Na)+
403.1308		156212	C23 H24 K O4	(M+K)+

Formula Calculator Element Limits

Element	Min	Max
C	3	100
H	0	120
O	0	30
N	0	5
S	0	2
Cl	0	0

Formula Calculator Results

Formula	Best	Mass	Tgt Mass	Diff (ppm)	Ion Species	Score
C23 H24 O4	TRUE	364.1674	364.1675	0.13	C23 H24 Na O4	99.94
C24 H20 N4		364.1674	364.1688	3.78	C24 H20 N4 Na	99.71
C20 H28 O4 S		364.1674	364.1708	9.37	C20 H28 Na O4 S	97.49
C15 H28 N2 O6 S		364.1674	364.1668	-1.69	C15 H28 N2 Na O6 S	96.99
C19 H28 N2 O S2		364.1674	364.1643	-8.56	C19 H28 N2 Na O S2	95.87
C23 H24 O4	TRUE	364.1677	364.1675	-0.53	C23 H24 K O4	99.96
C24 H20 N4		364.1677	364.1688	3.13	C24 H20 K N4	99.68
C20 H28 O4 S		364.1677	364.1708	8.71	C20 H28 K O4 S	98.27
C15 H28 N2 O6 S		364.1677	364.1668	-2.35	C15 H28 K N2 O6 S	97.93
C19 H28 N2 O S2		364.1677	364.1643	-9.22	C19 H28 K N2 O S2	96.79

--- End Of Report ---

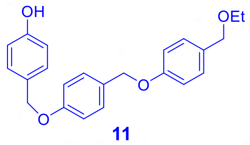

11

Fig. S125 The (+)-HR-ESI-MS report of compound **11**, page 2.

MS Formula Results: + Scan (8.023 min) Sub (2012041901.d)

m/z	Ion	Formula	Abundance
387.1566	(M+Na)+	C23 H24 Na O4	1656431.6

Best	Formula (M)	Ion Formula	Calc m/z	Score	Cross S	Mass	Calc Mass	Diff (ppm)	Abs Diff (ppm)	Abund Match	Spacing Mat	Mass Match	m/z	DBE
☑	C23 H24 O4	C23 H24 Na O4	387.1567	99.94		364.1674	364.1675	0.13	0.13	99.86	99.93	100	387.1566	12
☐	C24 H20 N4	C24 H20 N4 Na	387.158	99.71		364.1674	364.1688	3.78	3.78	99.83	99.83	99.59	387.1566	17
☐	C20 H28 O4 S	C20 H28 Na O4 S	387.1601	97.49		364.1674	364.1708	9.37	9.37	95.89	99.41	97.48	387.1566	7
☐	C15 H28 N2 O6 S	C15 H28 N2 Na O6 S	387.156	96.99		364.1674	364.1668	-1.69	1.69	90.24	99.21	99.92	387.1566	3
☐	C19 H28 N2 O S2	C19 H28 N2 Na O S2	387.1535	95.87		364.1674	364.1643	-8.56	8.56	89.95	98.94	97.89	387.1566	7

m/z	Ion	Formula	Abundance
403.1308	(M+K)+	C23 H24 K O4	156211.9

Best	Formula (M)	Ion Formula	Calc m/z	Score	Cross S	Mass	Calc Mass	Diff (ppm)	Abs Diff (ppm)	Abund Match	Spacing Mat	Mass Match	m/z	DBE
☑	C23 H24 O4	C23 H24 K O4	403.1306	99.96		364.1677	364.1675	-0.53	0.53	99.97	99.87	99.99	403.1308	12
☐	C24 H20 N4	C24 H20 K N4	403.132	99.68		364.1677	364.1688	3.13	3.13	99.39	99.94	99.74	403.1308	17
☐	C20 H28 O4 S	C20 H28 K O4 S	403.134	98.27		364.1677	364.1708	8.71	8.71	97.38	99.93	97.97	403.1308	7
☐	C15 H28 N2 O6 S	C15 H28 K N2 O6 S	403.13	97.93		364.1677	364.1668	-2.35	2.35	93.1	99.9	99.85	403.1308	3
☐	C19 H28 N2 O S2	C19 H28 K N2 O S2	403.1275	96.79		364.1677	364.1643	-9.22	9.22	92.76	99.77	97.73	403.1308	7

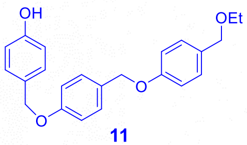

11

page 1

Fig. S126 The (+)-HR-ESI-MS report of compound **11**, page 3.

20200323 WYN-110/1
Bruker AVIII HD 600
PROTON DMSO D:\\ DATA2020 20

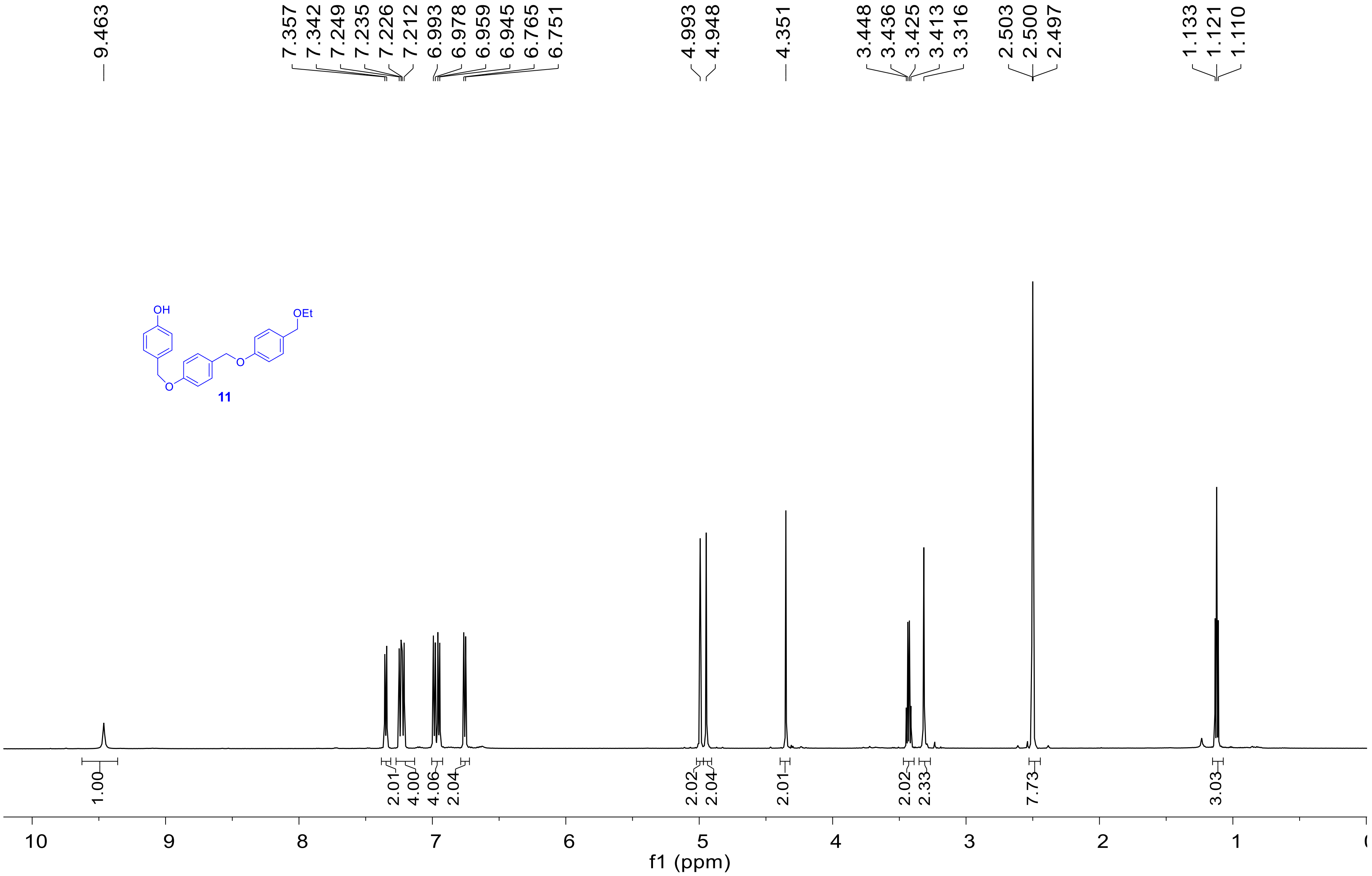

Fig. S127 The ^{1}H NMR spectrum of compound **11** in DMSO-d_6 (600 MHz).

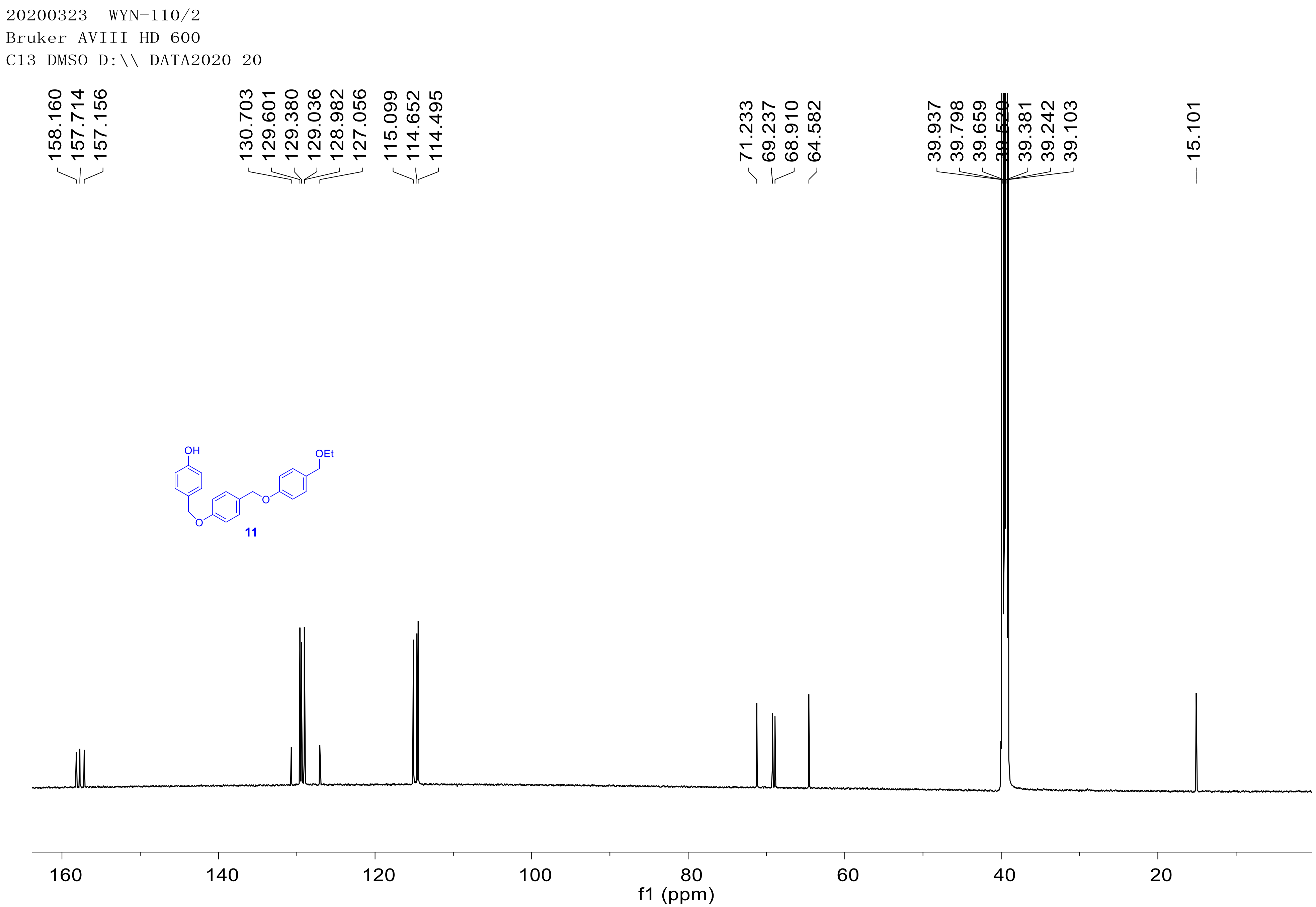

Fig. S128 The ^{13}C NMR spectrum of compound **11** in DMSO-d_6 (150 MHz).

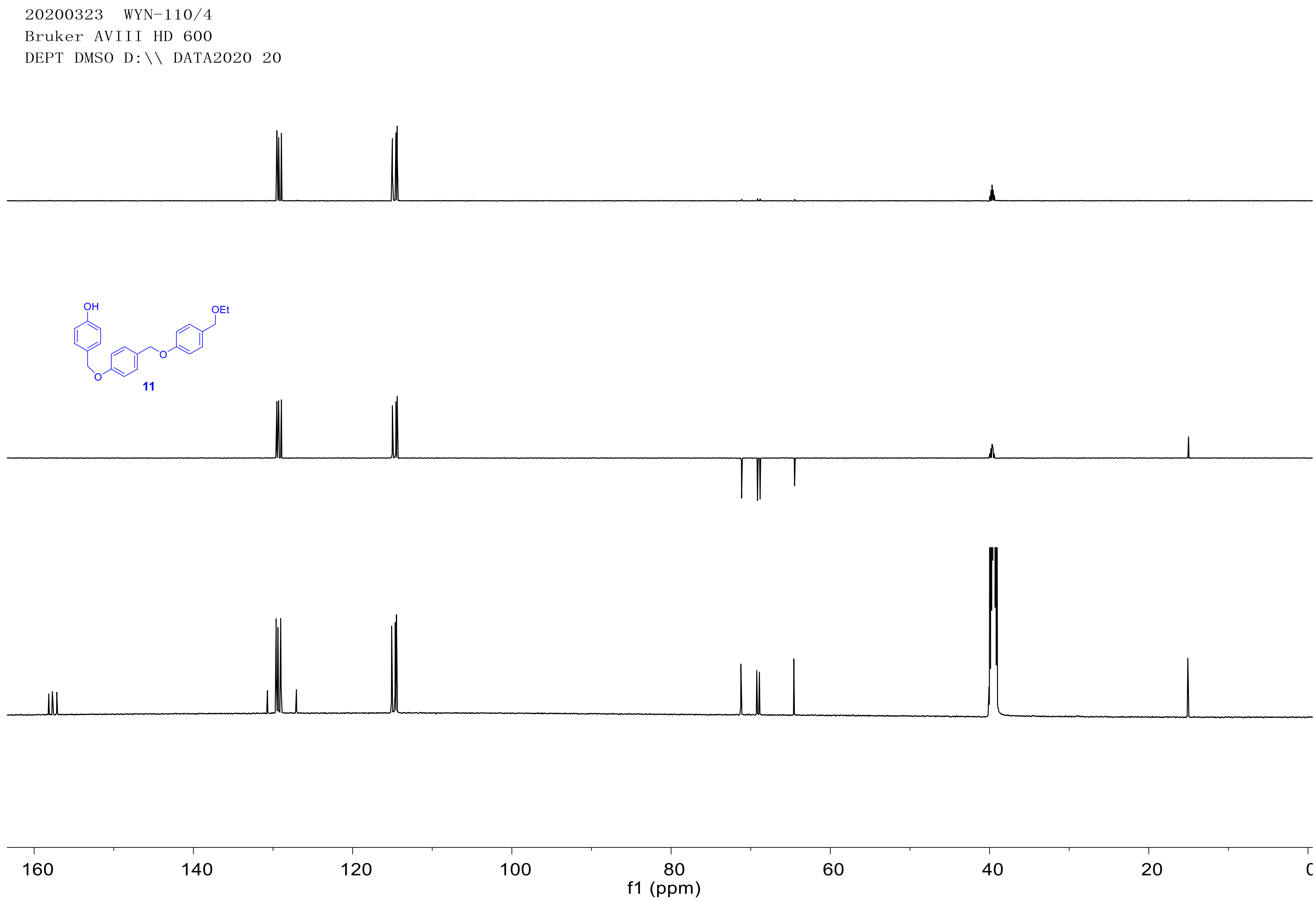

Fig. S129 The DEPT spectrum of compound **11** in DMSO-d_6 (150 MHz).

20200323 WYN-110/5
Bruker AVIII HD 600
COSY_MQF DMSO D:\\ DATA2020 20

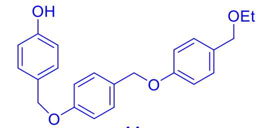

11

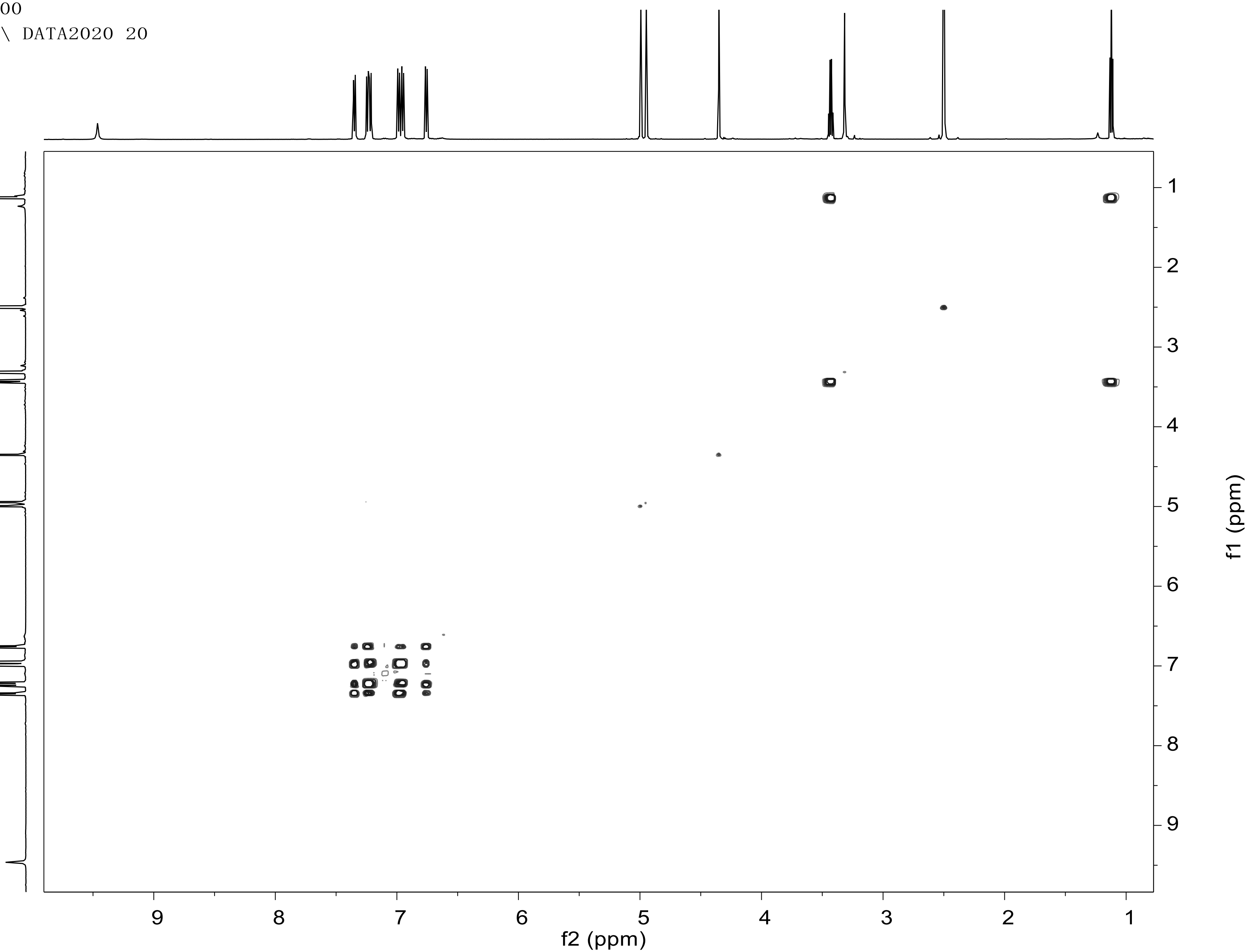

Fig. S130 The ^{1}H-^{1}H COSY spectrum of compound **11** in DMSO-d_6 at 600 MHz.

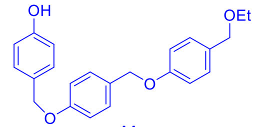

11

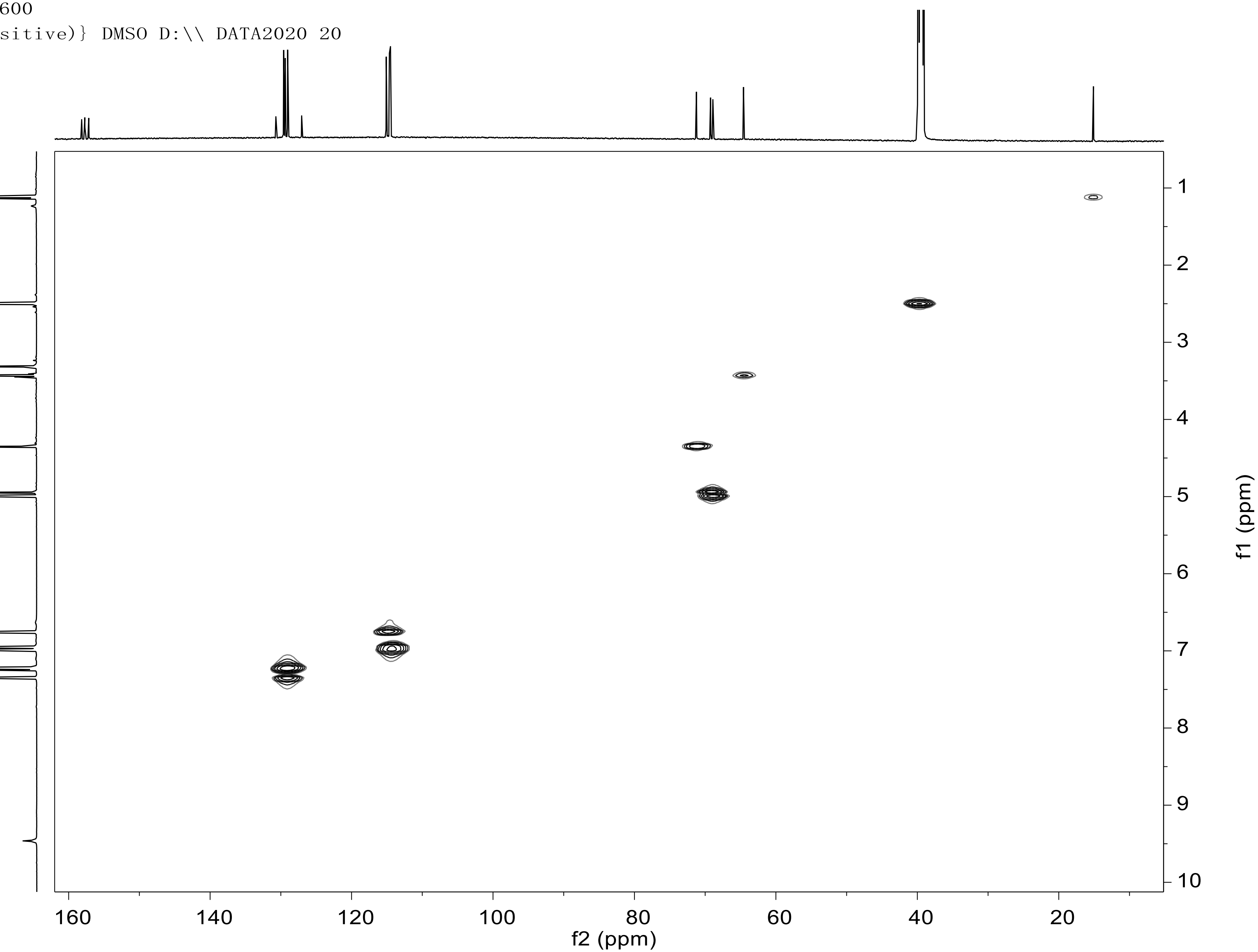

Fig. S131 The HSQC spectrum of compound **11** in DMSO-d_6 (500 MHz for ^{1}H).

20200323 WYN-110/7
Bruker AVIII HD 600
HMBC DMSO D:\\ DATA2020 20

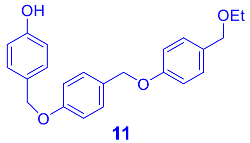

11

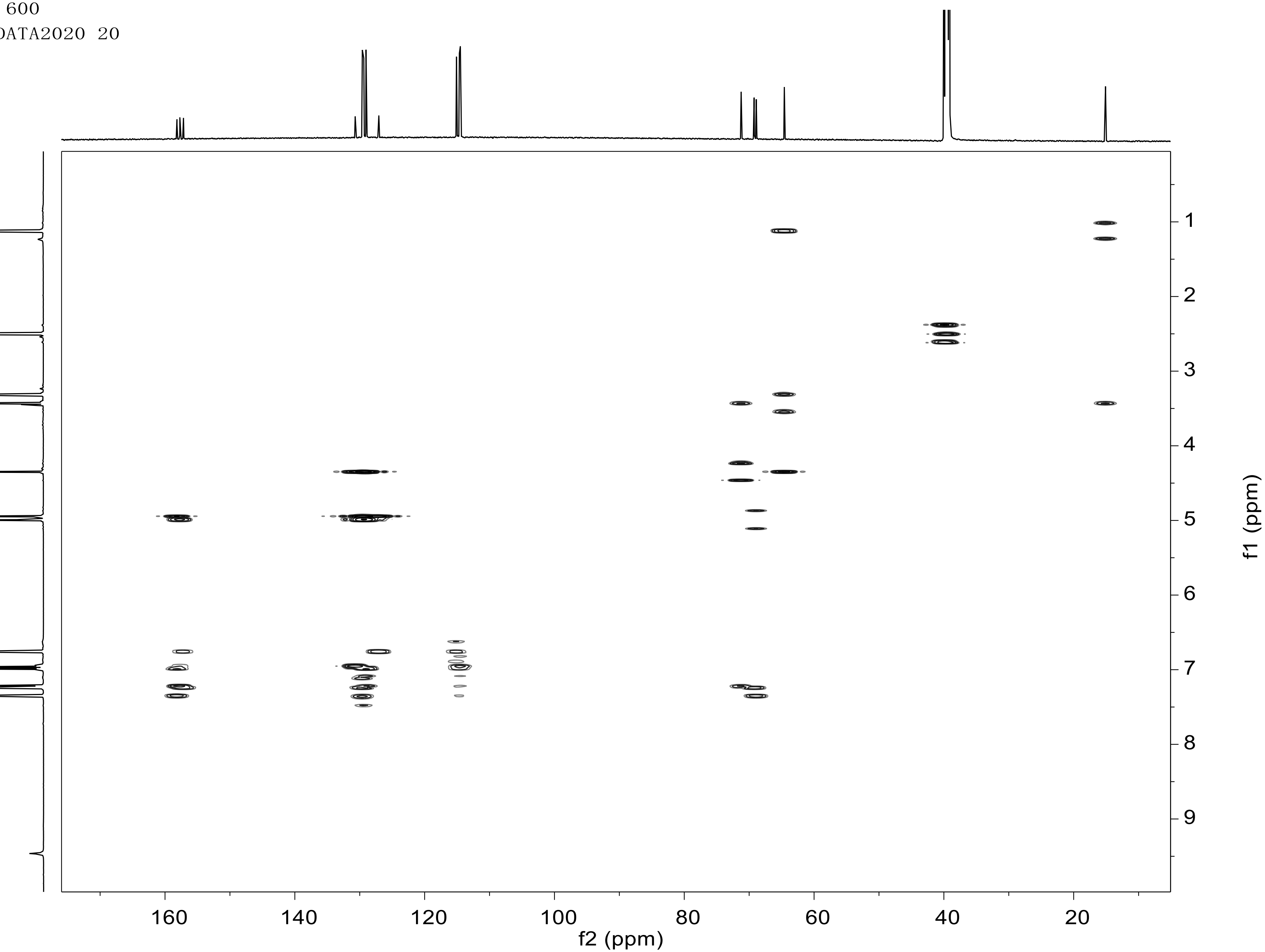

Fig. S132 The HMBC spectrum of compound **11** in DMSO-d_6 (600 MHz for ^{1}H).

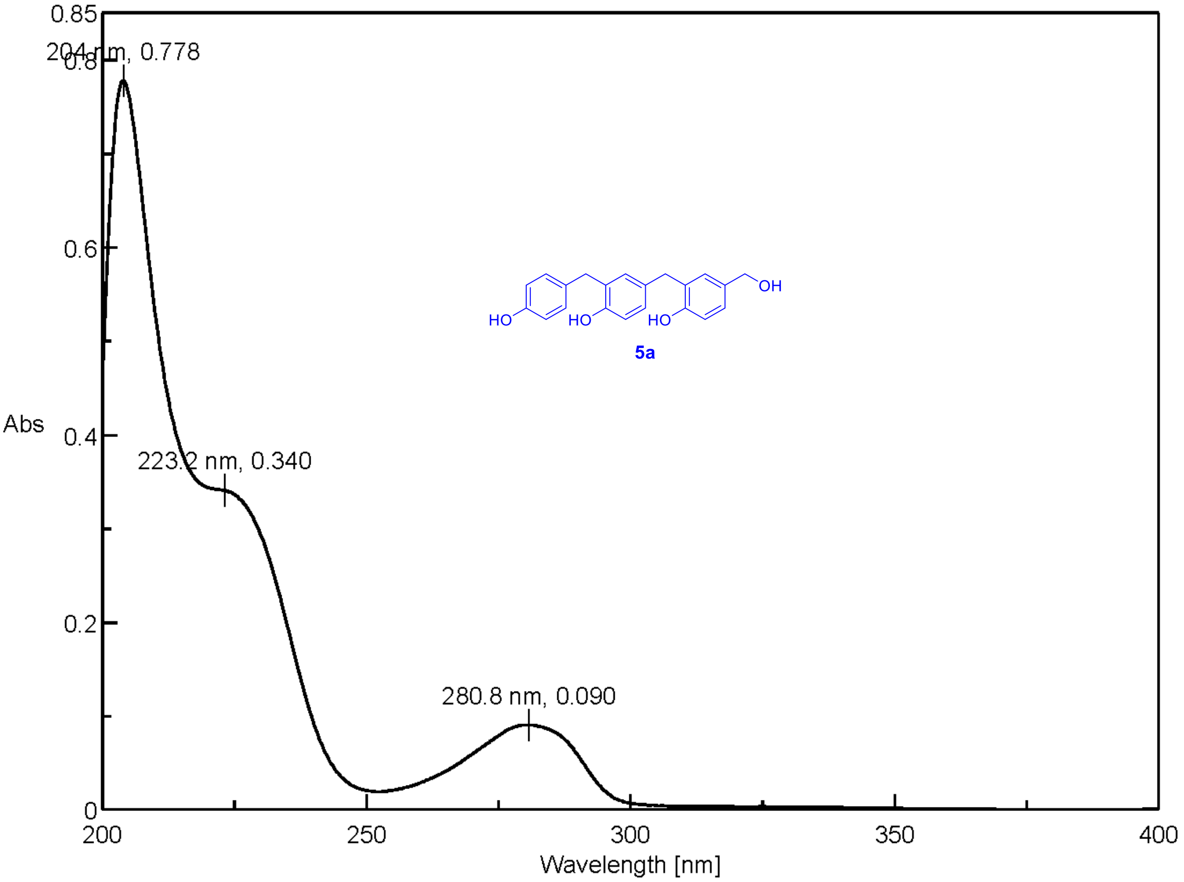

[Comment]
Sample Name ZM37-4D
Comment 0.02
User GQL
Division UV
Company 324

Memory-1

[Measurement Information]
Instrument Name V-650
Model Name V-650
Serial No. A034461150

Accessory PSC-718
Accessory S/N A001761114
Position 1
Cell Length 10 mm
Temperature 19.95 C
Control Sonsor Holder
Monitor Sensor Holder
Start Mode Start immediately

Photometric Mode Abs
Measurement range 400 - 190 nm
Data pitch 0.2 nm
Band width(UV/Vis) 2.0 nm
Response Medium
Scanning speed 200 nm/min
Source Change 340 nm
Light Source D2/WI
Filter Exchange Step
Correction Baseline

[Data Information]
Creation Date 2020-3-16 11:19

Data array type Linear data array
Horizontal Wavelength [nm]
Vertical Abs
Start 400 nm
End 190 nm
Data pitch 0.2 nm
Data points 1051

Fig. S133 The UV spectrum of compound **5a**.

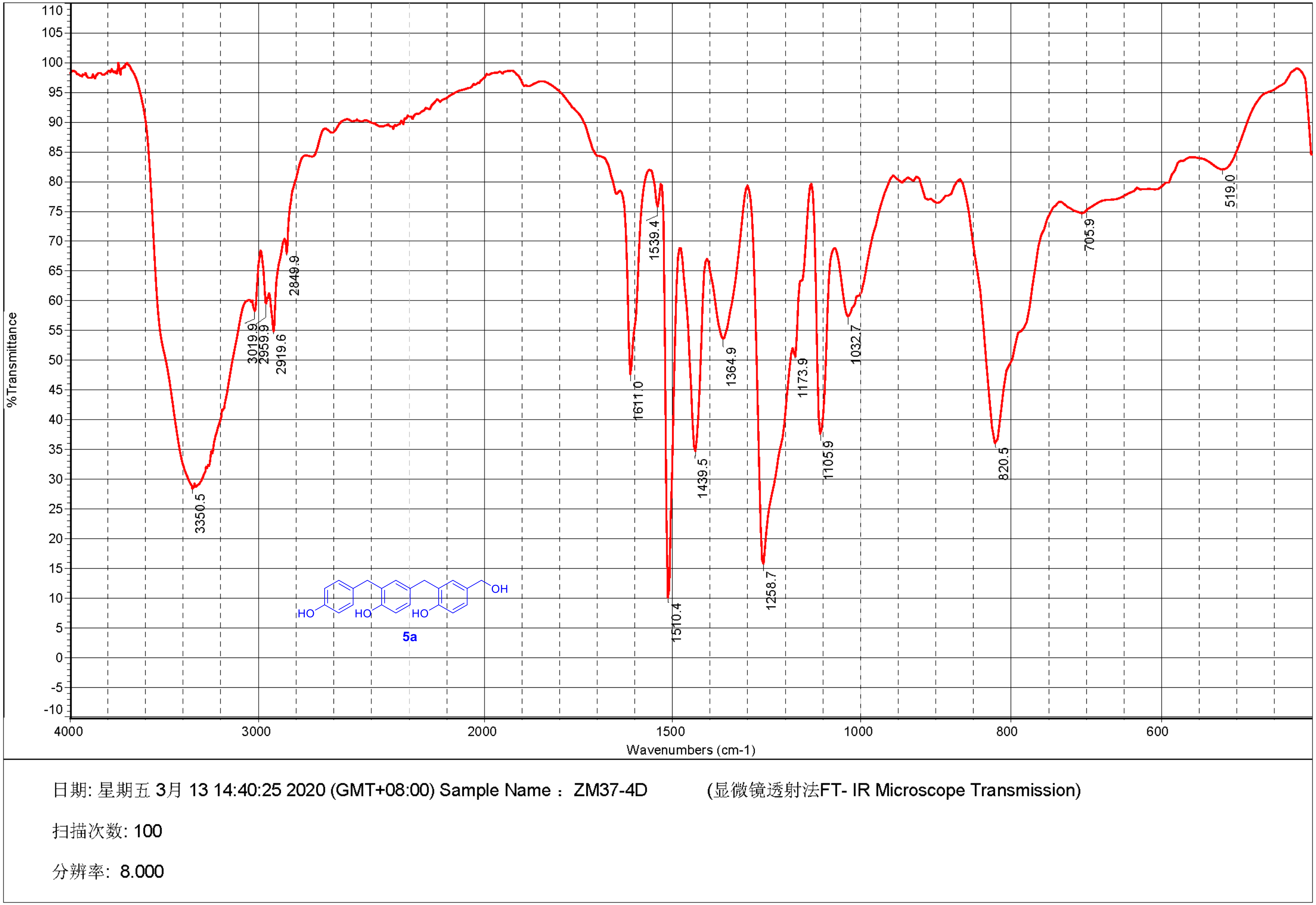

Fig. S134 The IR spectrum of compound **5a**.

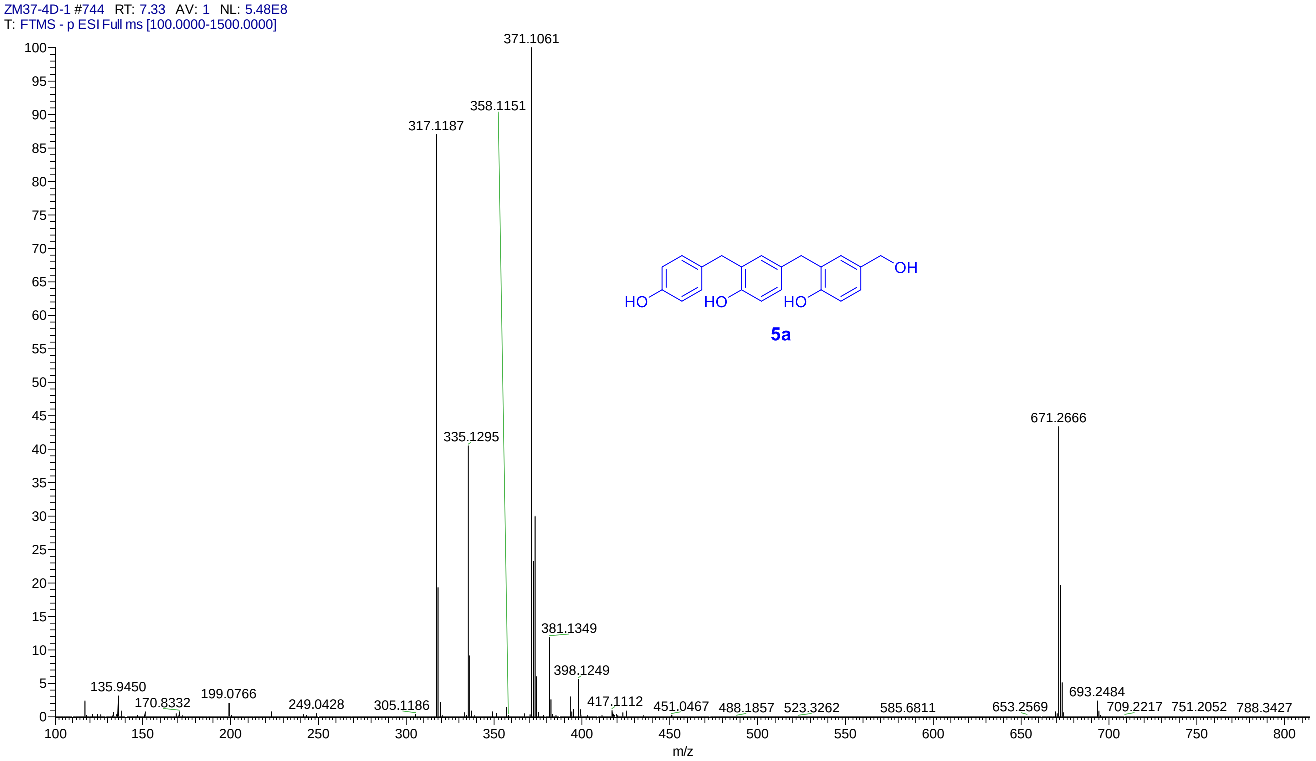

m/z	Theo. Mass	Delta (mmu)	RDB equiv.	Composition
335.1295	335.1278	1.73	12.5	C21 H19 O4
	335.1256	3.93	3.5	C15 H24 O6 Cl
	335.1337	-4.14	3.5	C14 H23 O9
	335.1197	9.8	12.5	C22 H20 O Cl
	335.1184	11.12	-0.5	C10 H23 O12
	335.1408	-11.33	7.5	C19 H24 O3 Cl
	335.1125	16.99	8.5	C17 H19 O7
	335.1103	19.18	-0.5	C11 H24 O9 Cl
	335.1489	-19.39	7.5	C18 H23 O6
	335.1045	25.06	8.5	C18 H20 O4 Cl
m/z	Theo. Mass	Delta (mmu)	RDB equiv.	Composition
371.1061	371.1045	1.62	11.5	C21 H20 O4 Cl
	371.1103	-4.26	2.5	C14 H24 O9 Cl
	371.1125	-6.45	11.5	C20 H19 O7
	371.0973	8.81	7.5	C16 H19 O10
	371.1184	-12.32	2.5	C13 H23 O12
	371.0892	16.87	7.5	C17 H20 O7 Cl
	371.1256	-19.51	6.5	C18 H24 O6 Cl
	371.082	24.06	3.5	C12 H19 O13
	371.1337	-27.58	6.5	C17 H23 O9
	371.0761	29.94	12.5	C19 H15 O8

Fig. S135 The (–)-HR-ESI-MS report of compound **5a**.

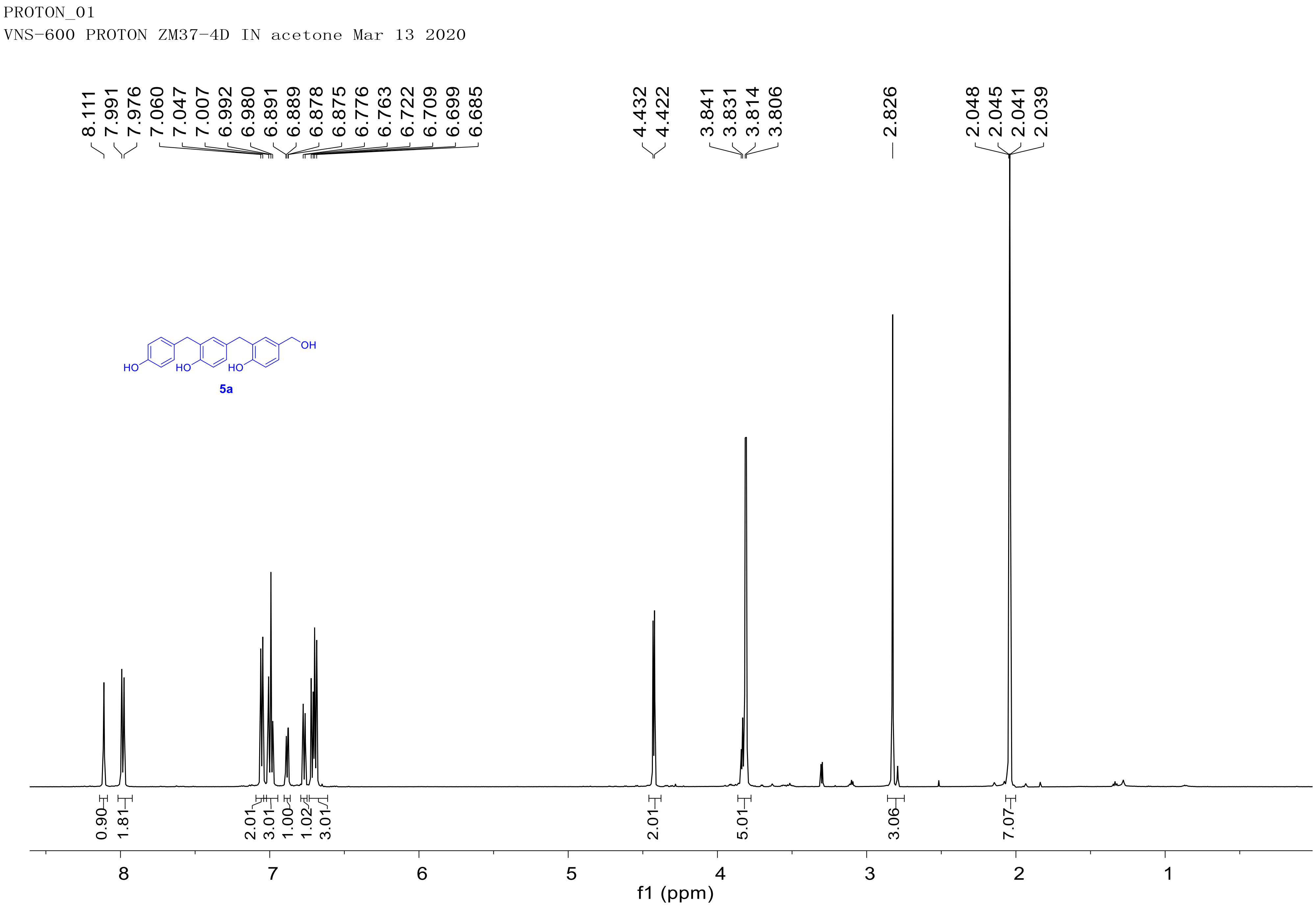

Fig. S136 The ^{1}H NMR spectrum of compound **5a** in acetone-d_6 (600 MHz).

CARBON_01
VNS-600 CARBON ZM37-4D IN acetone Mar 13 2020

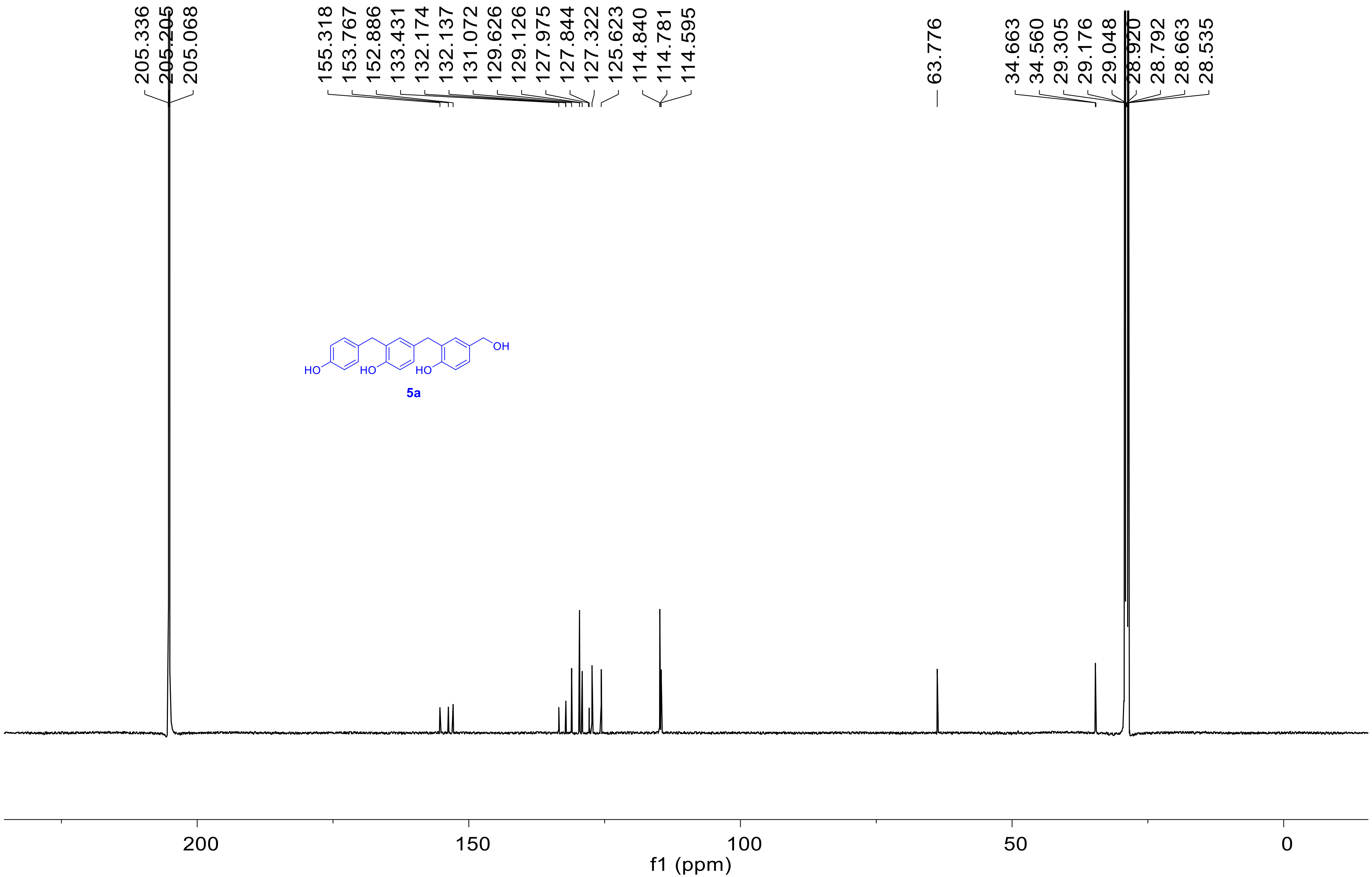

Fig. S137 The ^{13}C NMR spectrum of compound **5a** in acetone-d_6 (150 MHz).

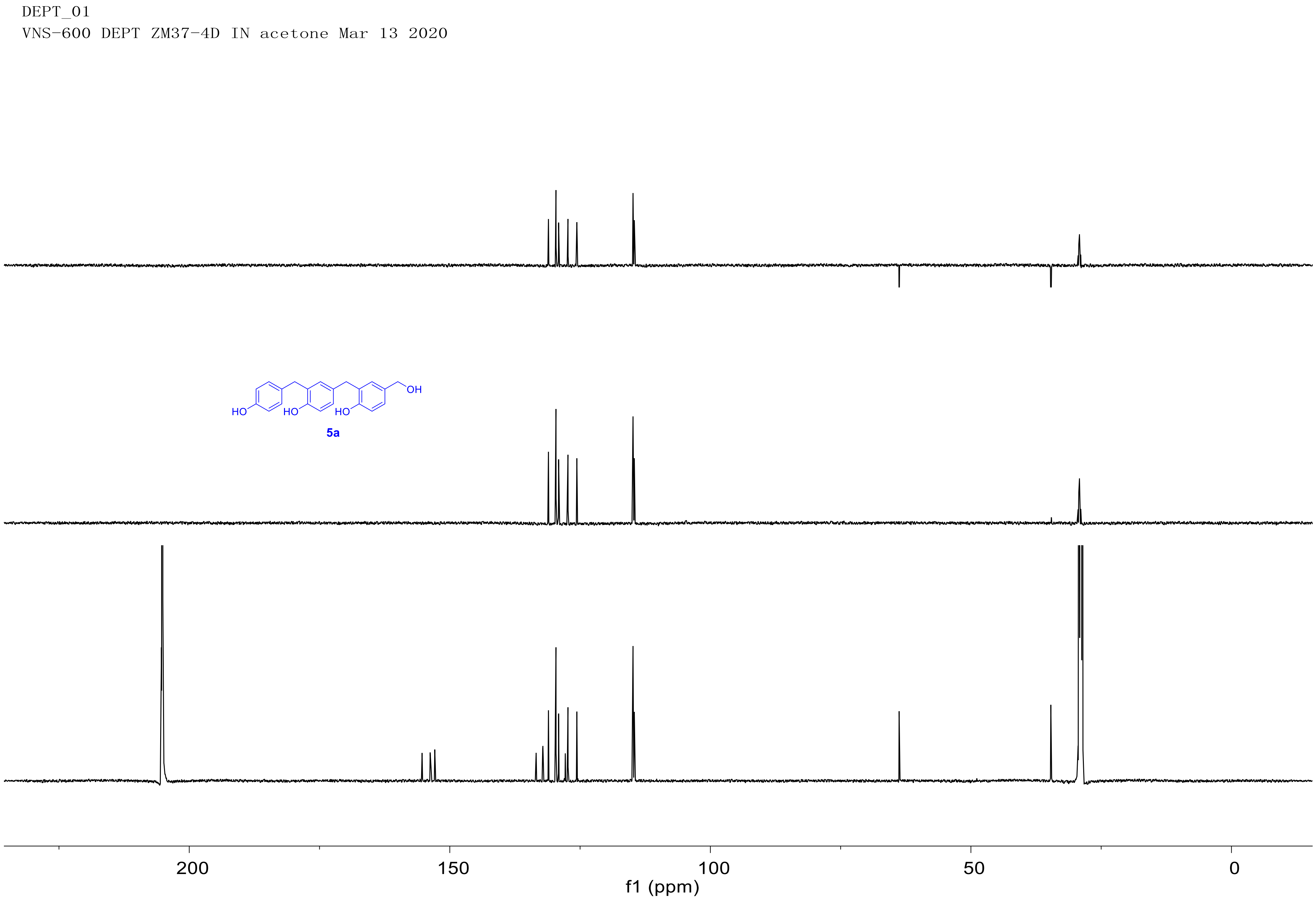

Fig. S138 The DEPT spectrum of compound **5a** in acetone-d_6 (150 MHz).

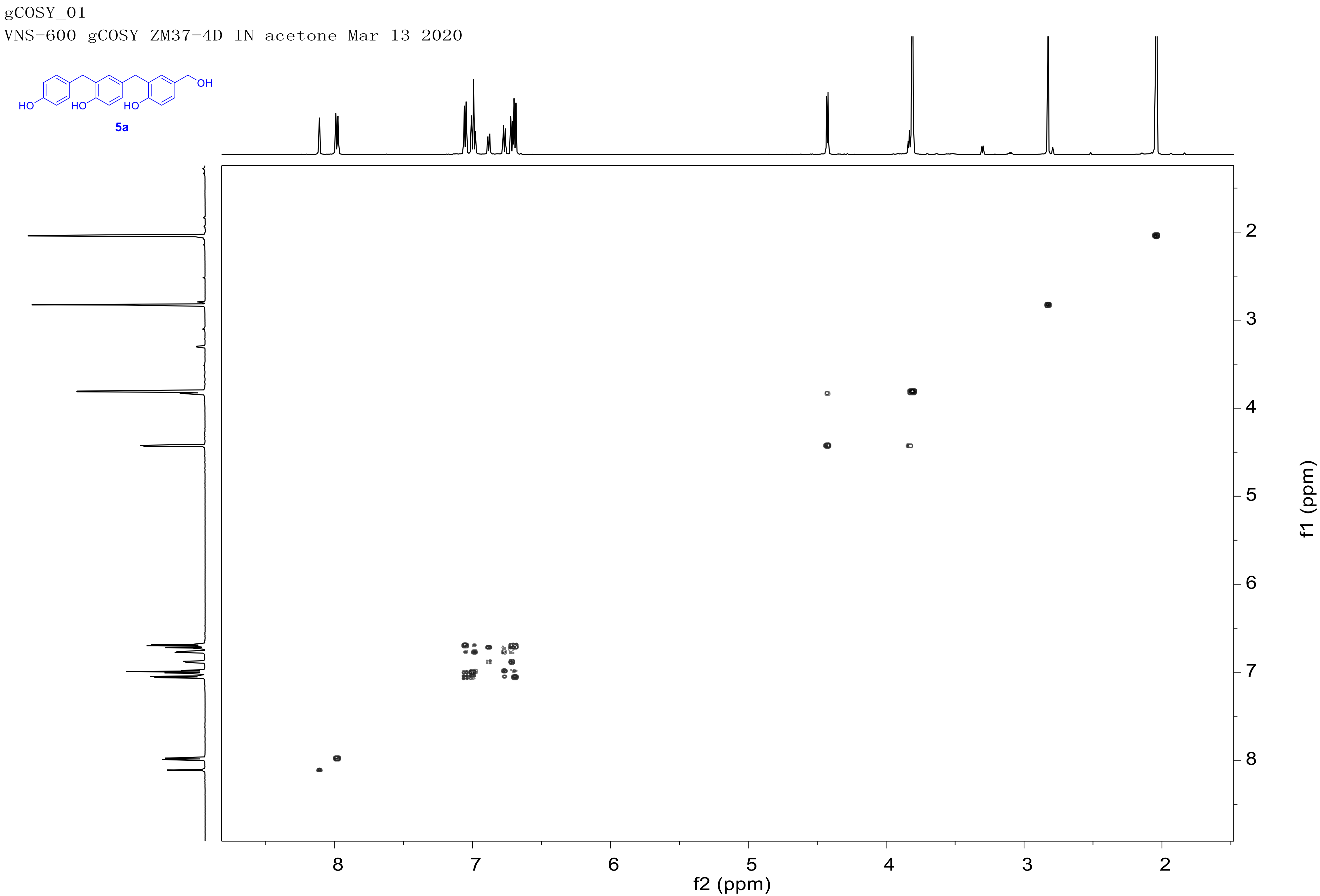

Fig. S139 The ^{1}H-^{1}H COSY spectrum of compound **5a** in acetone-d_6 (600 MHz).

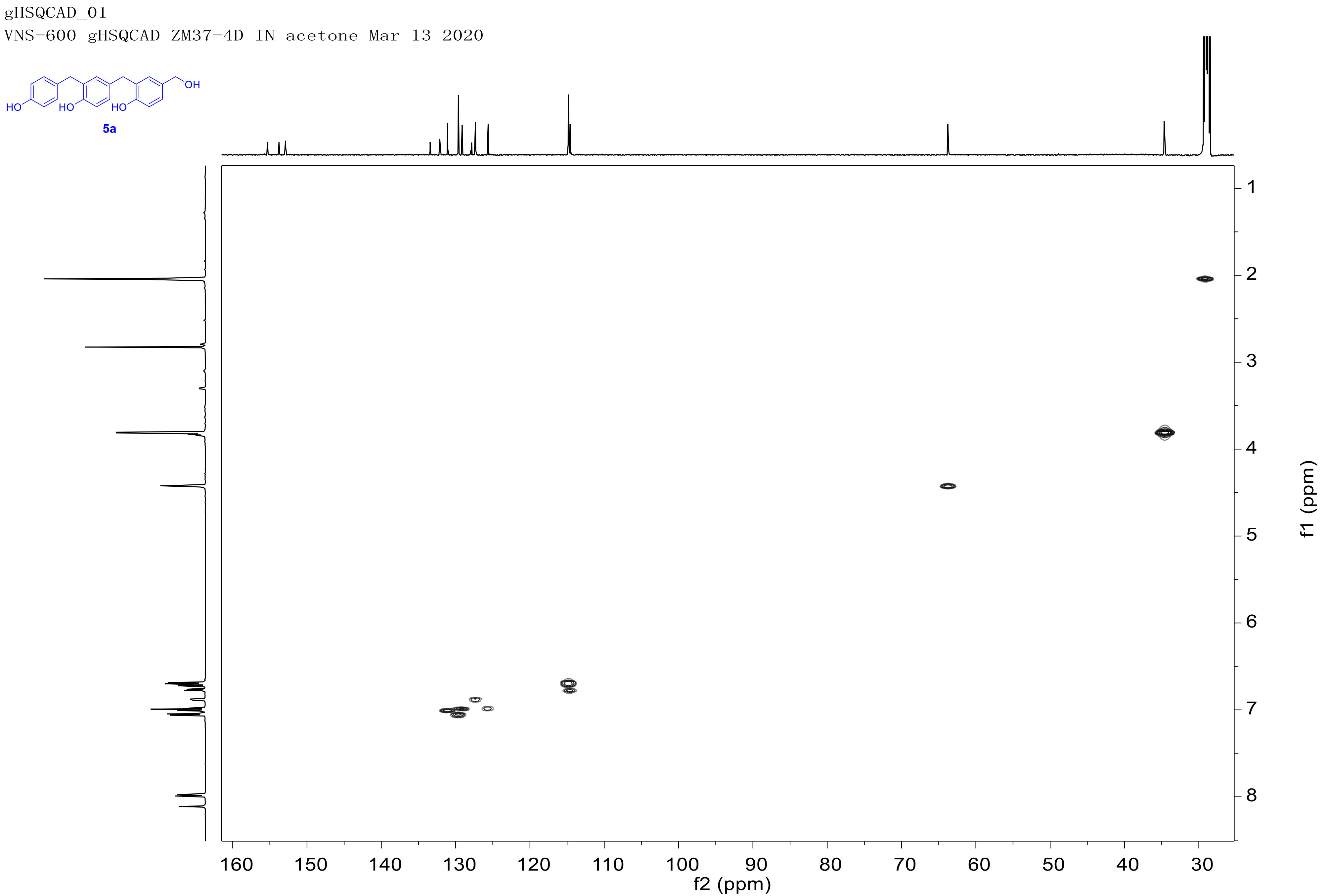

Fig. S140 The HSQC spectrum of compound **5a** in acetone-d_6 (600 MHz for ^{1}H).

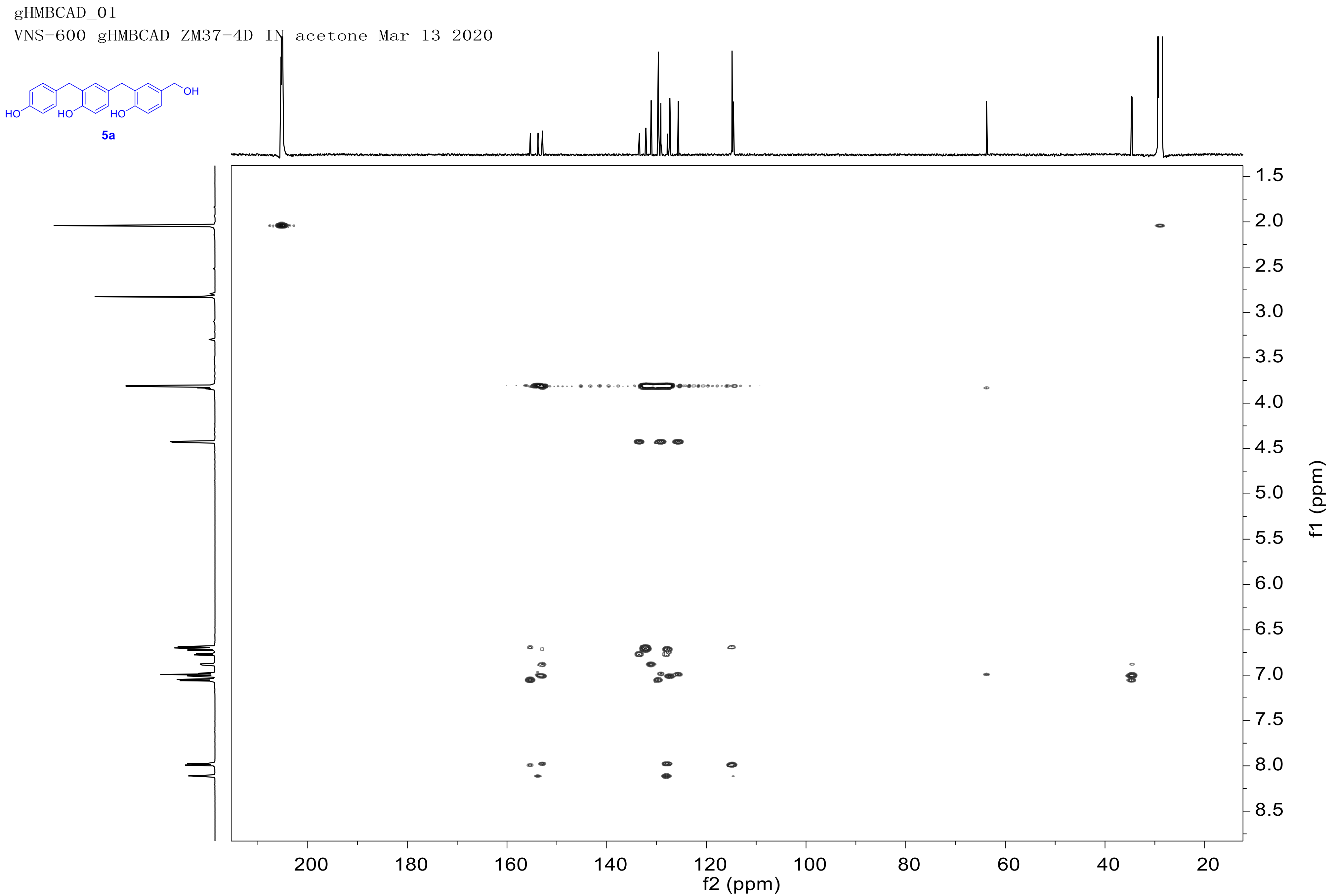

Fig. S141 The HMBC spectrum of compound **5a** in acetone-d_6 (600 MHz for ^{1}H) .

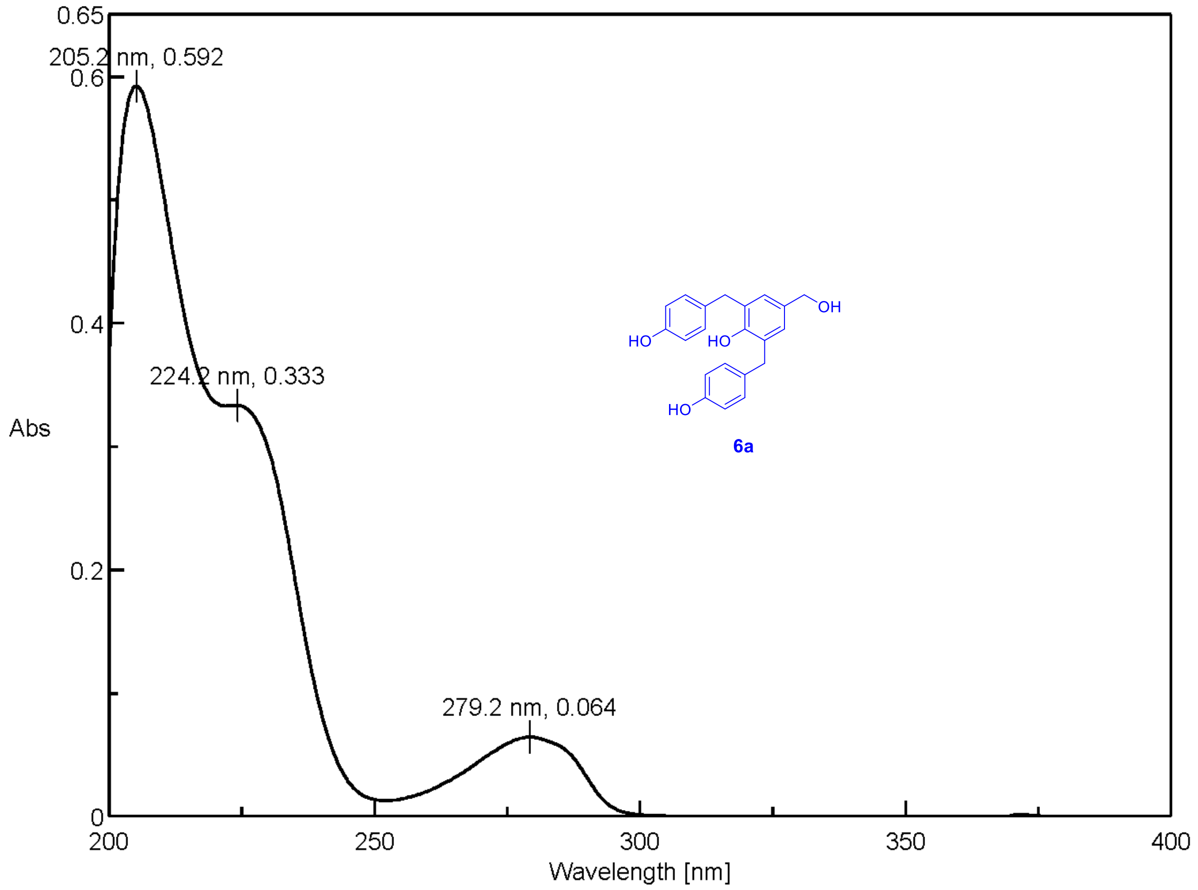

[Comment]
Sample Name ZM37-2b
Comment 0.02
User GQL
Division UV
Company 324

[Measurement Information]
Instrument Name V-650
Model Name V-650
Serial No. A034461150

Accessory PSC-718
Accessory S/N A001761114
Position 1
Cell Length 10 mm
Temperature 19.98 C
Control Sonsor Holder
Monitor Sensor Holder
Start Mode Start immediately

Photometric Mode Abs
Measurement range 400 - 190 nm
Data pitch 0.2 nm
Band width(UV/Vis) 2.0 nm
Response Medium
Scanning speed 200 nm/min
Source Change 340 nm
Light Source D2/WI
Filter Exchange Step
Correction Baseline

——— Memory-1

[Data Information]
Creation Date 2020-3-16 11:25

Data array type Linear data array
Horizontal Wavelength [nm]
Vertical Abs
Start 400 nm
End 190 nm
Data pitch 0.2 nm
Data points 1051

Fig. S142 The UV spectrum of compound **6a.**

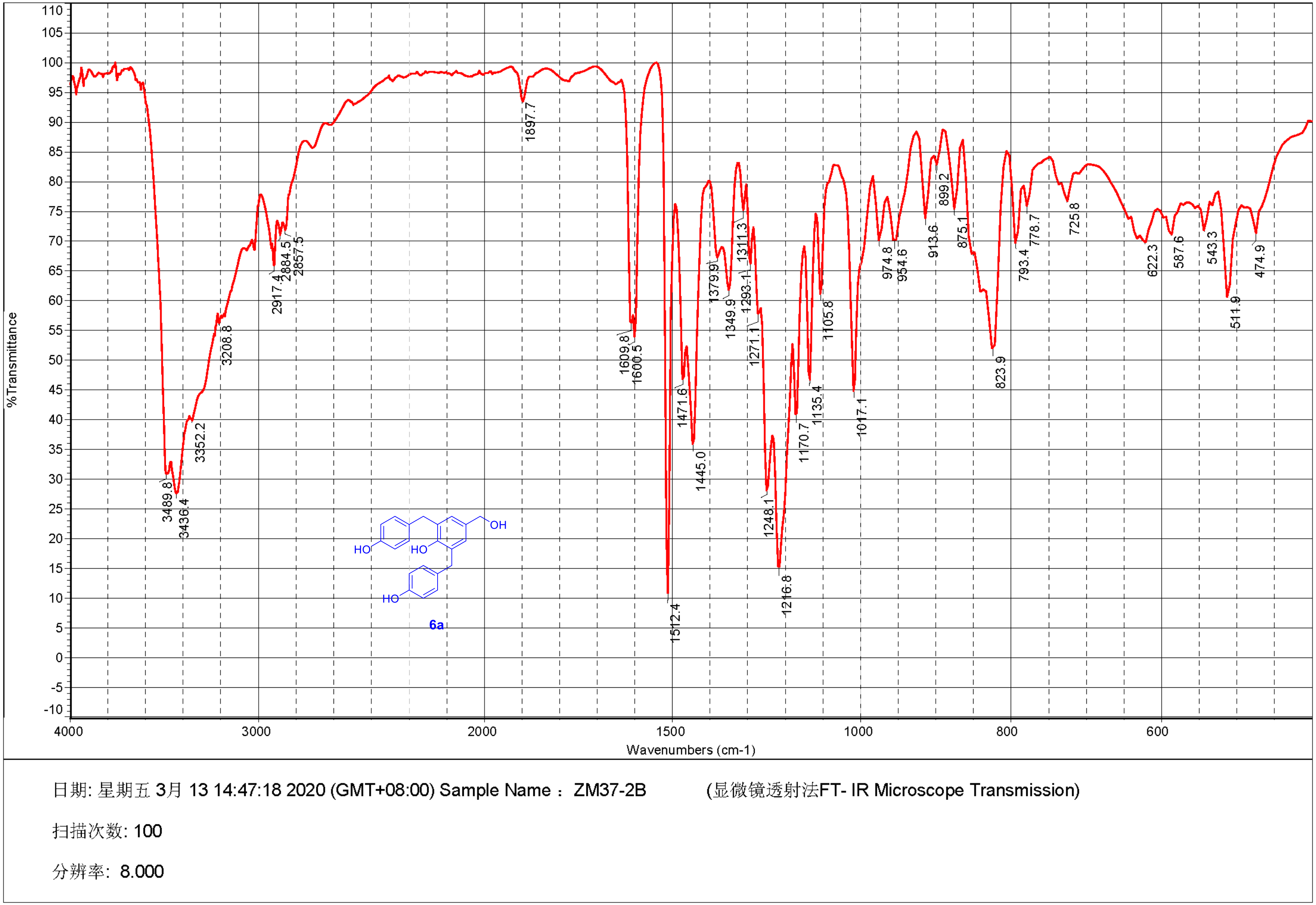

Fig. S143 The IR spectrum of compound **6a**.

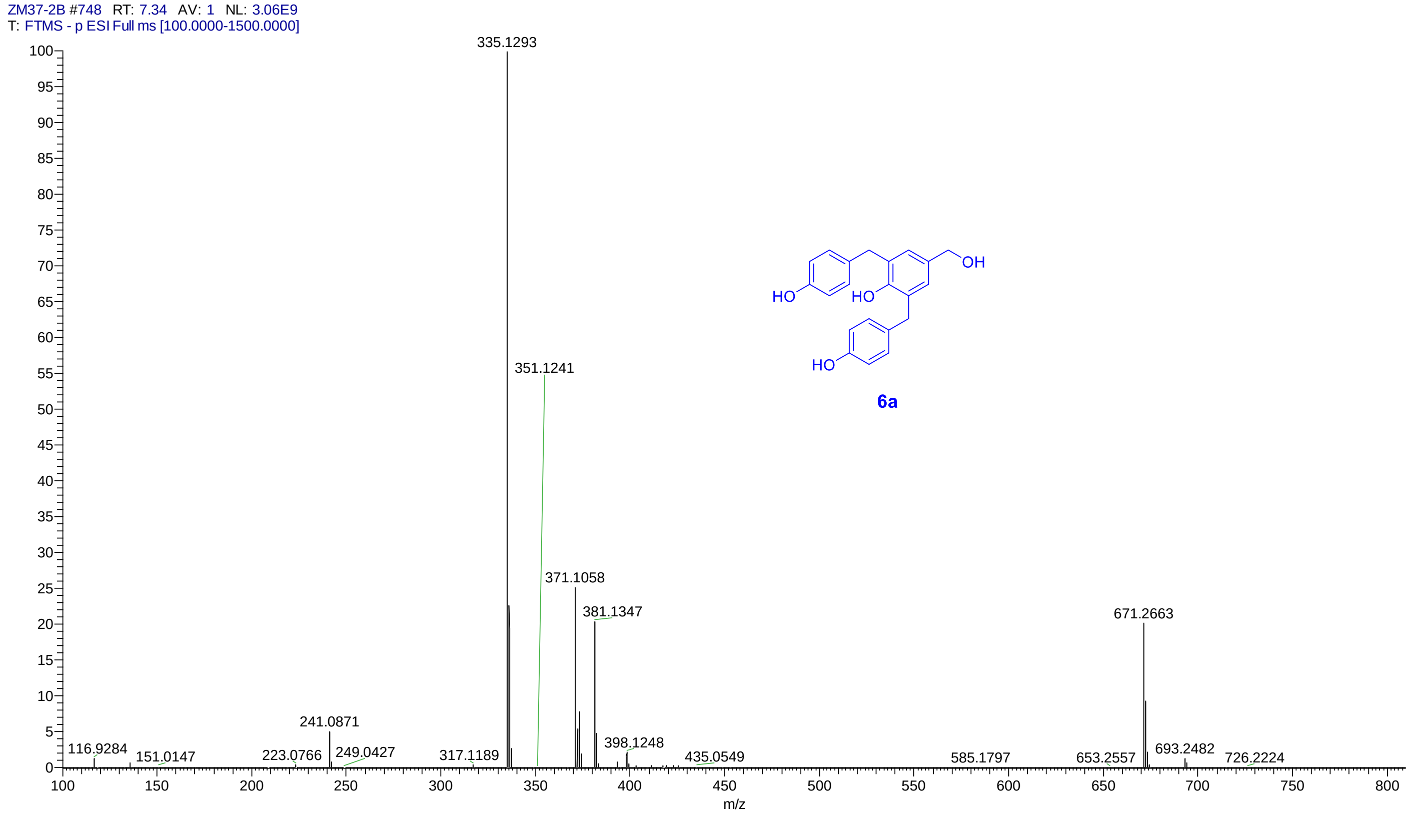

m/z	Theo. Mass	Delta (mmu)	RDB equiv.	Composition
335.1293	335.1278	1.51	12.5	C21 H19 O4
	335.1256	3.71	3.5	C15 H24 O6 Cl
	335.1337	-4.36	3.5	C14 H23 O9
	335.1197	9.58	12.5	C22 H20 O Cl
	335.1184	10.9	-0.5	C10 H23 O12
	335.1408	-11.55	7.5	C19 H24 O3 Cl
	335.1125	16.77	8.5	C17 H19 O7
	335.1103	18.96	-0.5	C11 H24 O9 Cl
	335.1489	-19.61	7.5	C18 H23 O6
	335.1045	24.84	8.5	C18 H20 O4 Cl
**m/z**	**Theo. Mass**	**Delta (mmu)**	**RDB equiv.**	**Composition**
371.1058	371.1045	1.37	11.5	C21 H20 O4 Cl
	371.1103	-4.51	2.5	C14 H24 O9 Cl
	371.1125	-6.7	11.5	C20 H19 O7
	371.0973	8.56	7.5	C16 H19 O10
	371.1184	-12.57	2.5	C13 H23 O12
	371.0892	16.62	7.5	C17 H20 O7 Cl
	371.1256	-19.76	6.5	C18 H24 O6 Cl
	371.082	23.81	3.5	C12 H19 O13
	371.1337	-27.83	6.5	C17 H23 O9
	371.0761	29.69	12.5	C19 H15 O8

Fig. S144 The (–)-HR-ESI-MS report of compound **6a**.

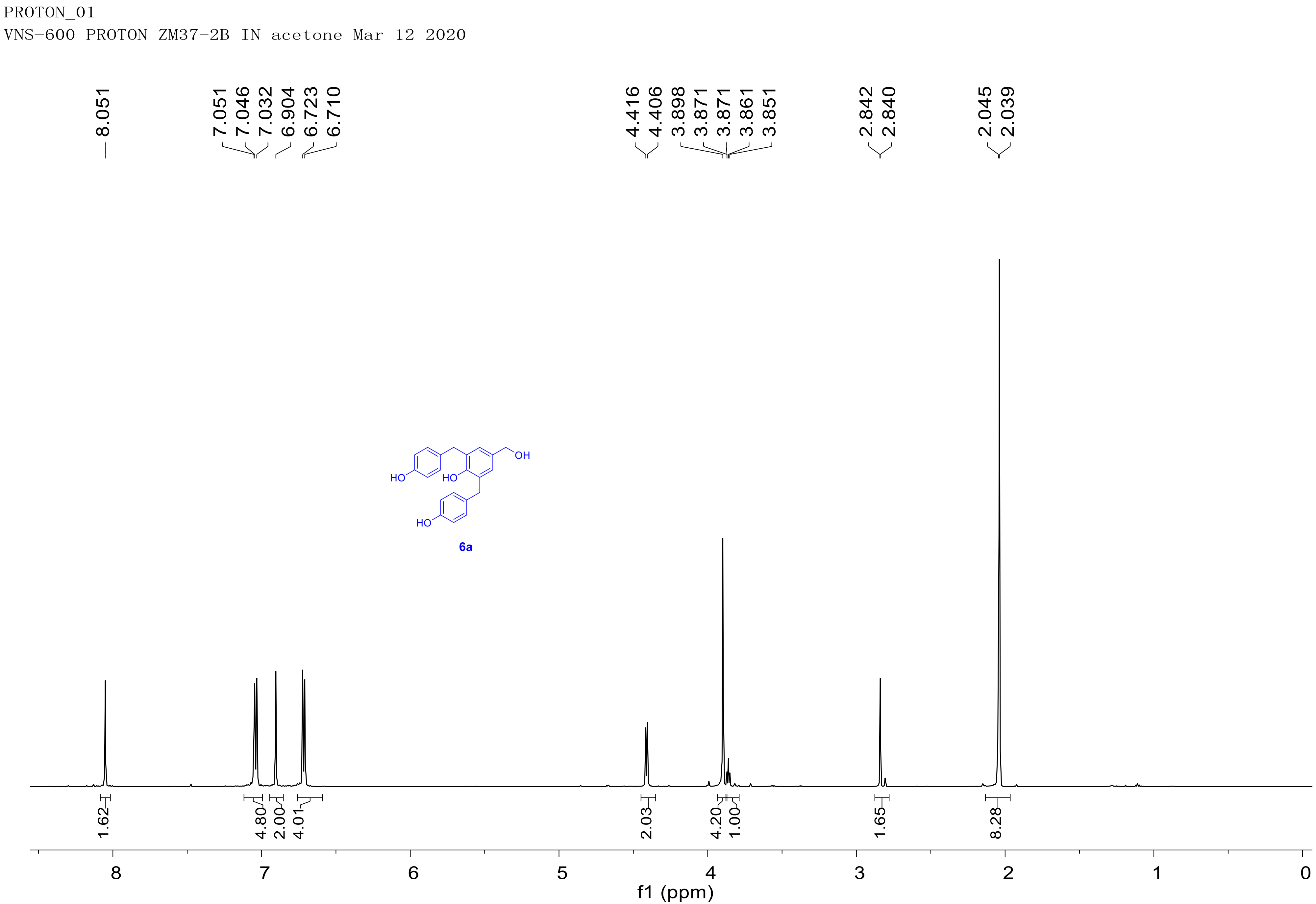

Fig. S145 The ^{1}H NMR spectrum of compound **6a** in acetone-d_6 (600 MHz).

CARBON_01
VNS-600 CARBON ZM37-2B IN acetone Mar 12 2020

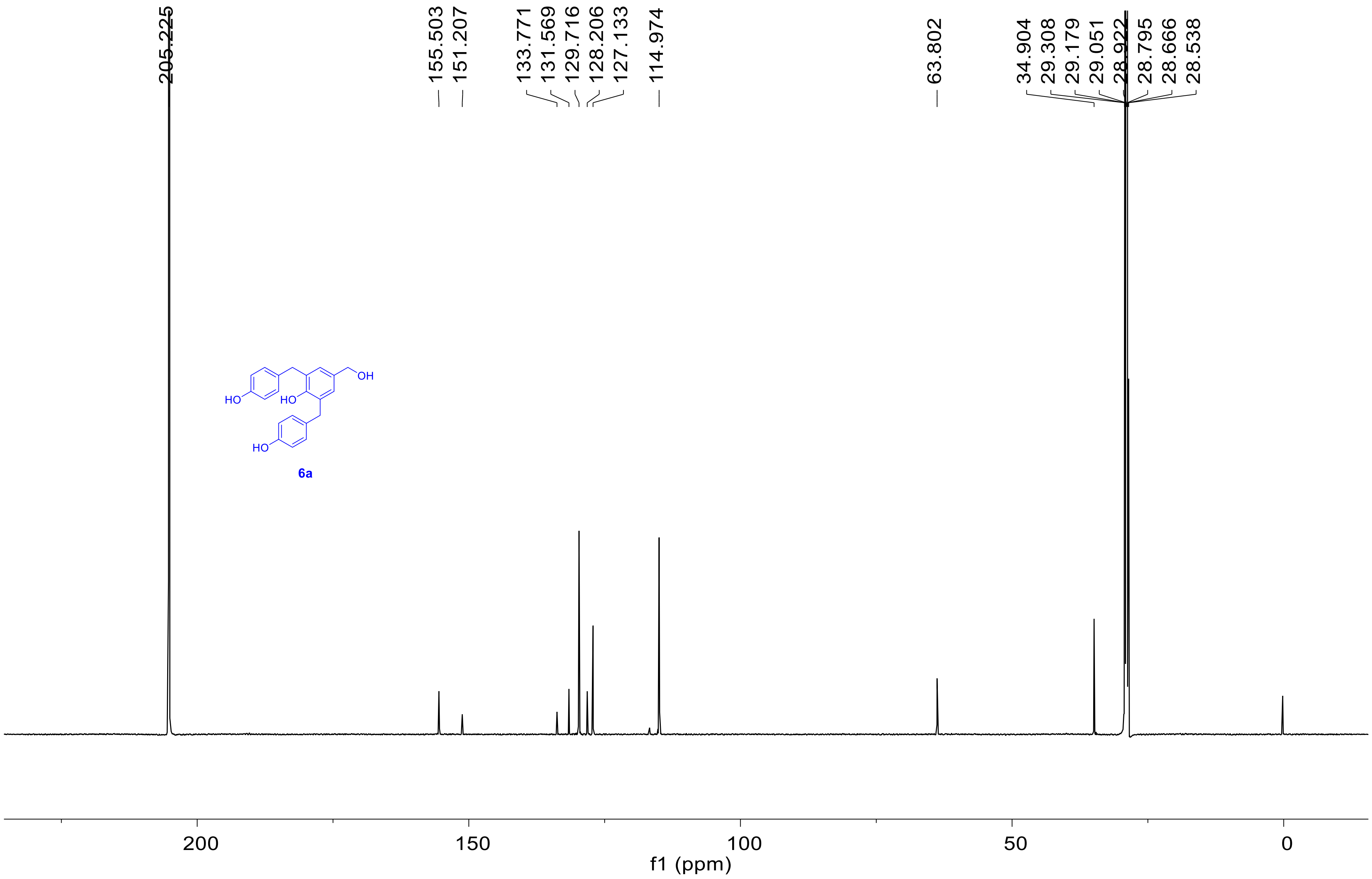

Fig. S146 The ^{13}C NMR spectrum of compound **6a** in acetone-d_6 (150 MHz).

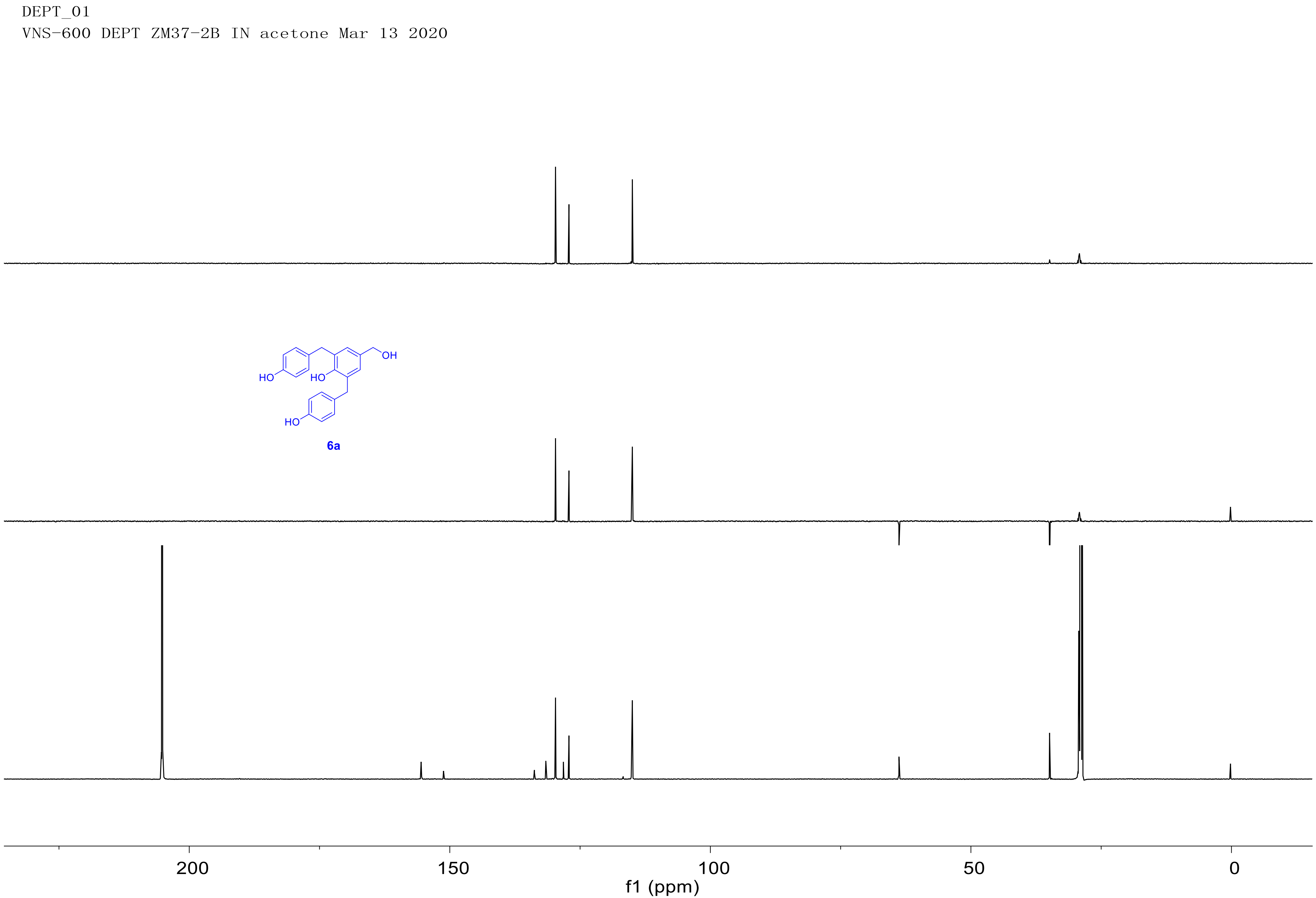

Fig. S147 The DEPT spectrum of compound **6a** in acetone-d_6 (150 MHz).

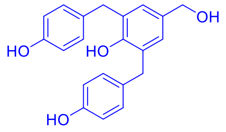

6a

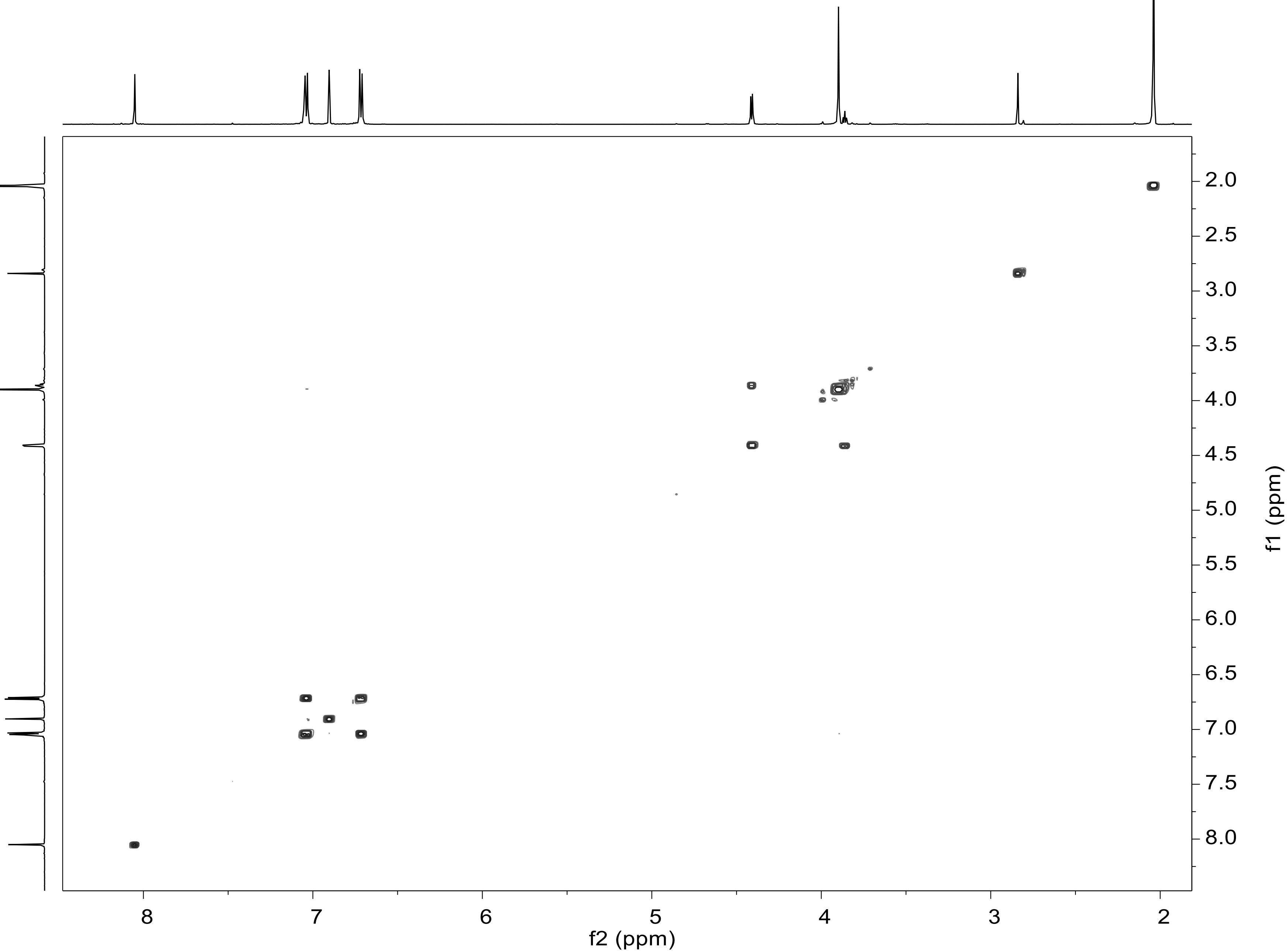

Fig. S148 The ^{1}H-^{1}H COSY spectrum of compound **6a** in acetone-d_6 (600 MHz).

gHSQCAD_01
VNS-600 gHSQCAD ZM37-2B IN acetone Mar 13 2020

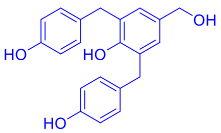

6a

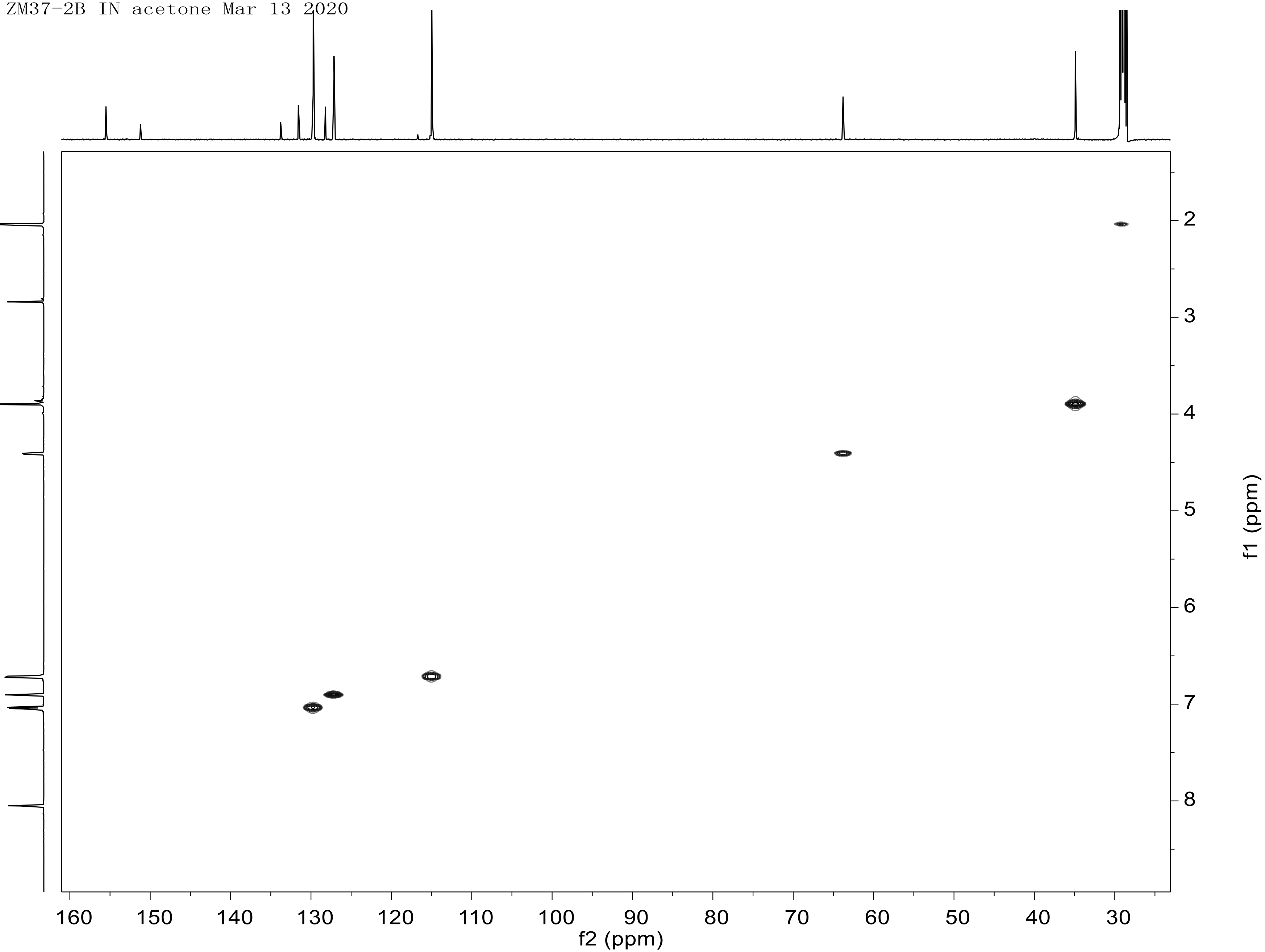

Fig. S149 The HSQC spectrum of compound **6a** in acetone-d_6 (600 MHz for ^{1}H).

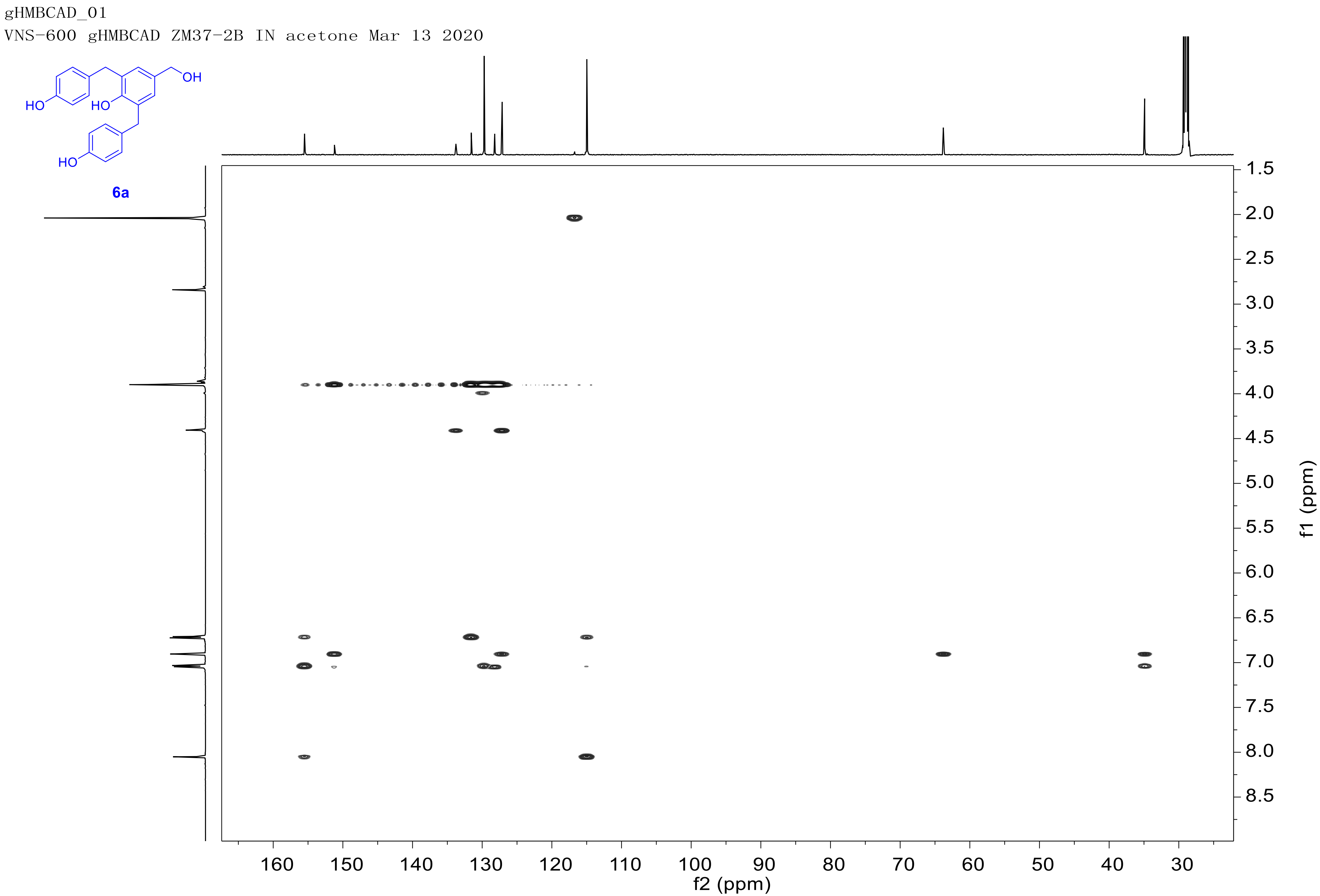

Fig. S150 The HMBC spectrum of compound **6a** in acetone-d_6 (600 MHz for ^{1}H).

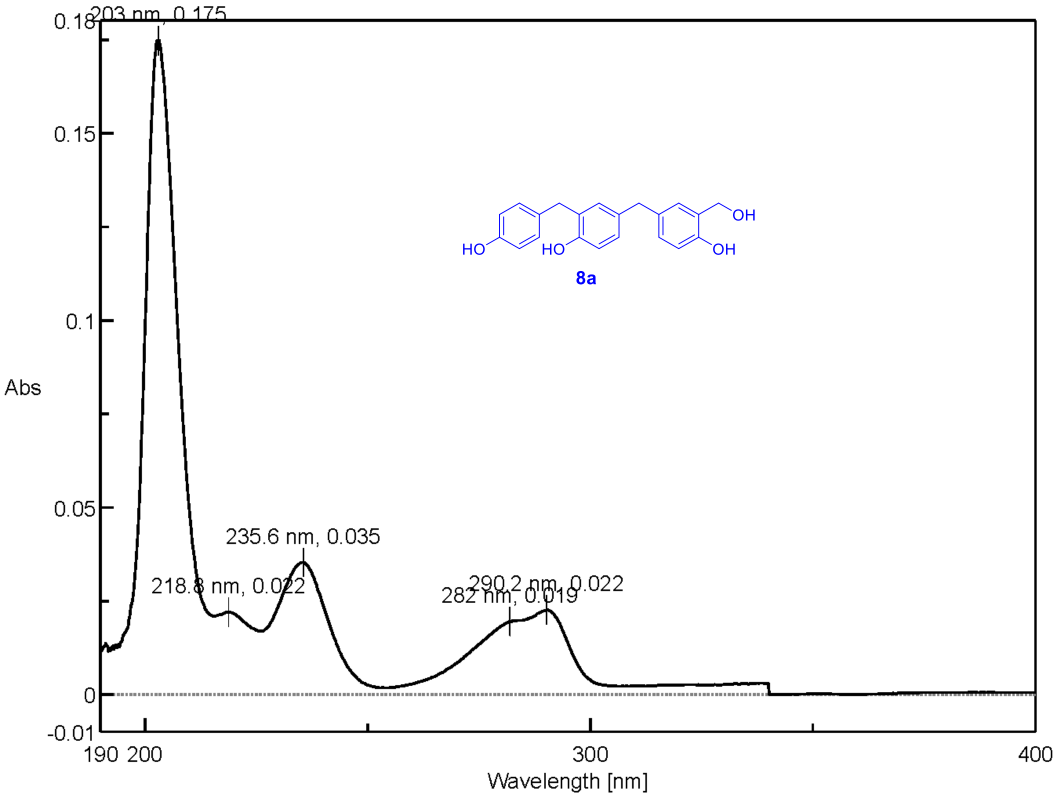

[Comment]
Sample Name H-6
Comment CH3CN
User HJW
Division UV
Company 324

b2b

[Measurement Information]
Instrument Name V-650
Model Name V-650
Serial No. A034461150

Accessory PSC-718
Accessory S/N A001761114
Position 1
Cell Length 10 mm
Temperature 19.96 C
Control Sonsor Holder
Monitor Sensor Holder
Start Mode Start immediately

Photometric Mode Abs
Measurement range 400 - 190 nm
Data pitch 0.2 nm
Band width(UV/Vis) 2.0 nm
Response Medium
Scanning speed 200 nm/min
Source Change 340 nm
Light Source D2/WI
Filter Exchange Step
Correction Baseline

[Data Information]
Creation Date 2019-11-13 11:30

Data array type Linear data array
Horizontal Wavelength [nm]
Vertical Abs
Start 400 nm
End 190 nm
Data pitch 0.2 nm
Data points 1051

Fig. S151 The UV spectrum of compound **8a**.

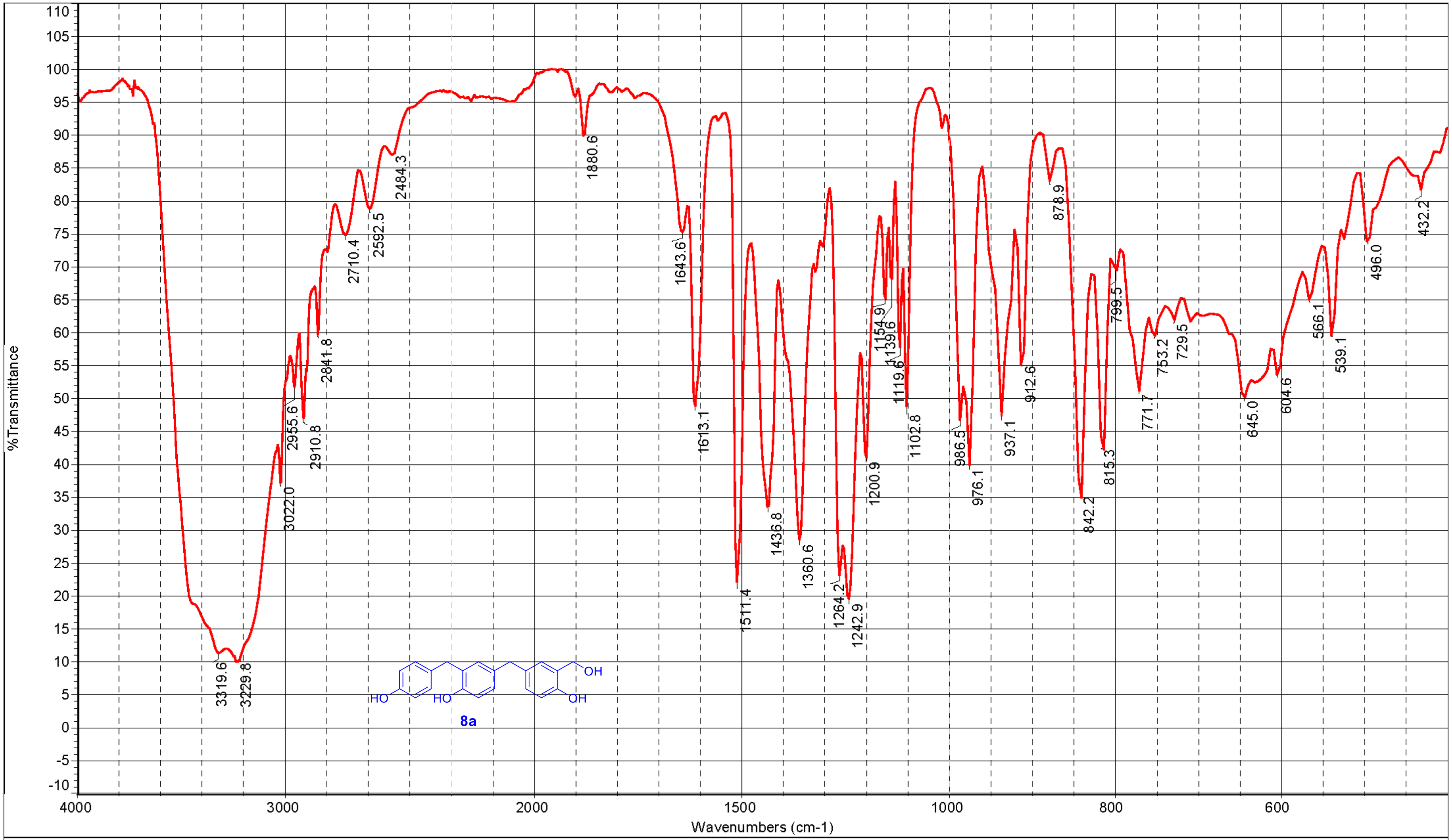

日期: 星期一 12月 09 14:20:19 2019 (GMT+08:00Sample Name： b2b　　(显微镜透射法 FT- IR Microscope Transmission)

扫描次数: 100

分辨率: 8.000

Fig. S152 The IR spectrum of compound **8a**.

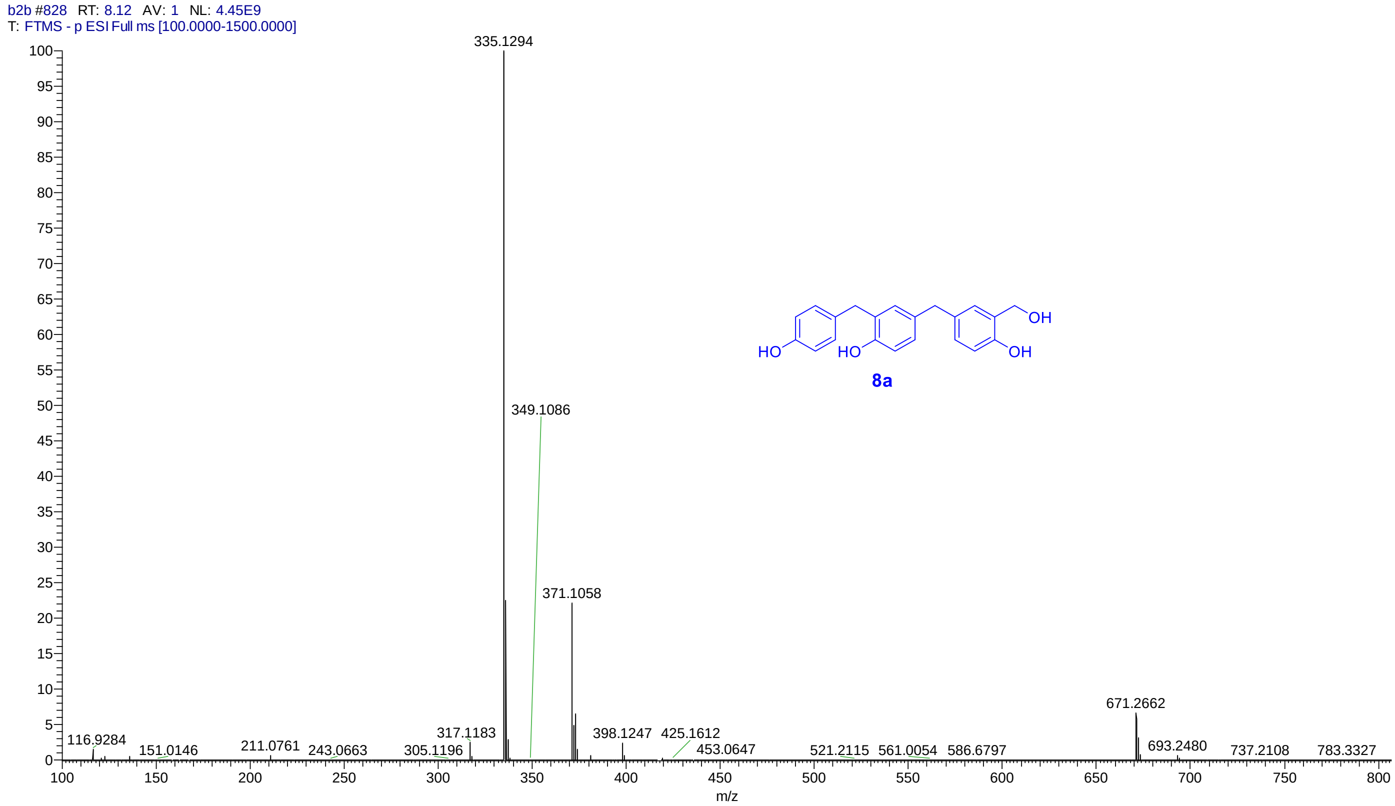

m/z	Theo. Mass	Delta (mmu)	RDB equiv.	Composition
335.1294	335.1278	1.57	12.5	C21 H19 O4
	335.1256	3.77	3.5	C15 H24 O6 Cl
	335.1337	-4.3	3.5	C14 H23 O9
	335.1197	9.64	12.5	C22 H20 O Cl
	335.1184	10.96	-0.5	C10 H23 O12
	335.1408	-11.49	7.5	C19 H24 O3 Cl
	335.1125	16.83	8.5	C17 H19 O7
	335.1103	19.02	-0.5	C11 H24 O9 Cl
	335.1489	-19.55	7.5	C18 H23 O6
	335.1045	24.9	8.5	C18 H20 O4 Cl

m/z	Theo. Mass	Delta (mmu)	RDB equiv.	Composition
371.1058	371.1045	1.34	11.5	C21 H20 O4 Cl
	371.1103	-4.54	2.5	C14 H24 O9 Cl
	371.1125	-6.73	11.5	C20 H19 O7
	371.0973	8.53	7.5	C16 H19 O10
	371.1184	-12.6	2.5	C13 H23 O12
	371.0892	16.59	7.5	C17 H20 O7 Cl
	371.1256	-19.79	6.5	C18 H24 O6 Cl
	371.082	23.78	3.5	C12 H19 O13
	371.1337	-27.86	6.5	C17 H23 O9
	371.0761	29.66	12.5	C19 H15 O8

Fig. S153 The (–)-HR-ESI-MS report of compound **8a**.

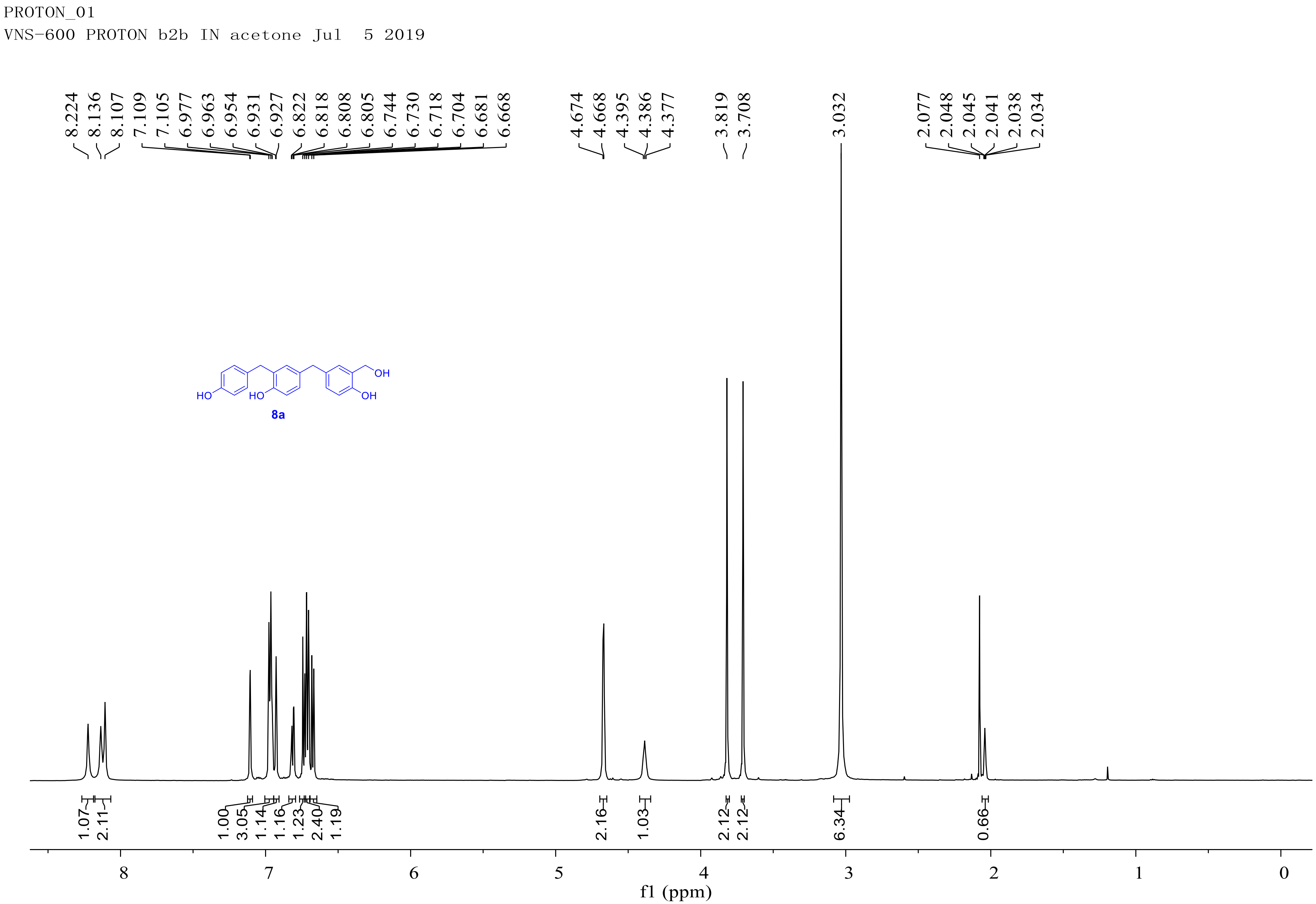

Fig. S154 The ^{1}H NMR spectrum of compound **8a** in acetone-d_6 (600 MHz).

CARBON_01
VNS-600 CARBON b2b IN acetone Jul 5 2019

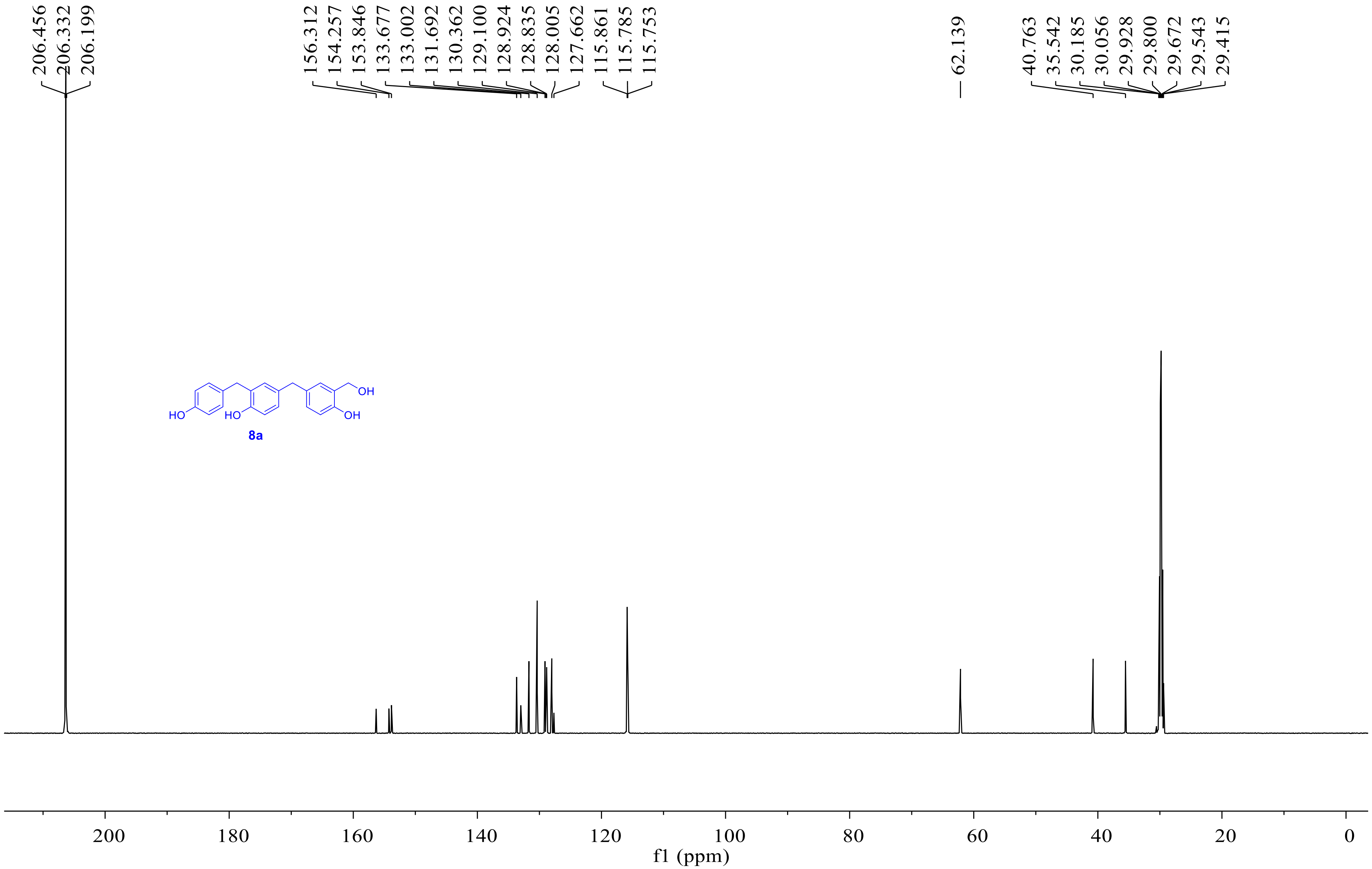

Fig. S155 The ^{13}C NMR spectrum of compound **8a** in acetone-d_6 (150 MHz).

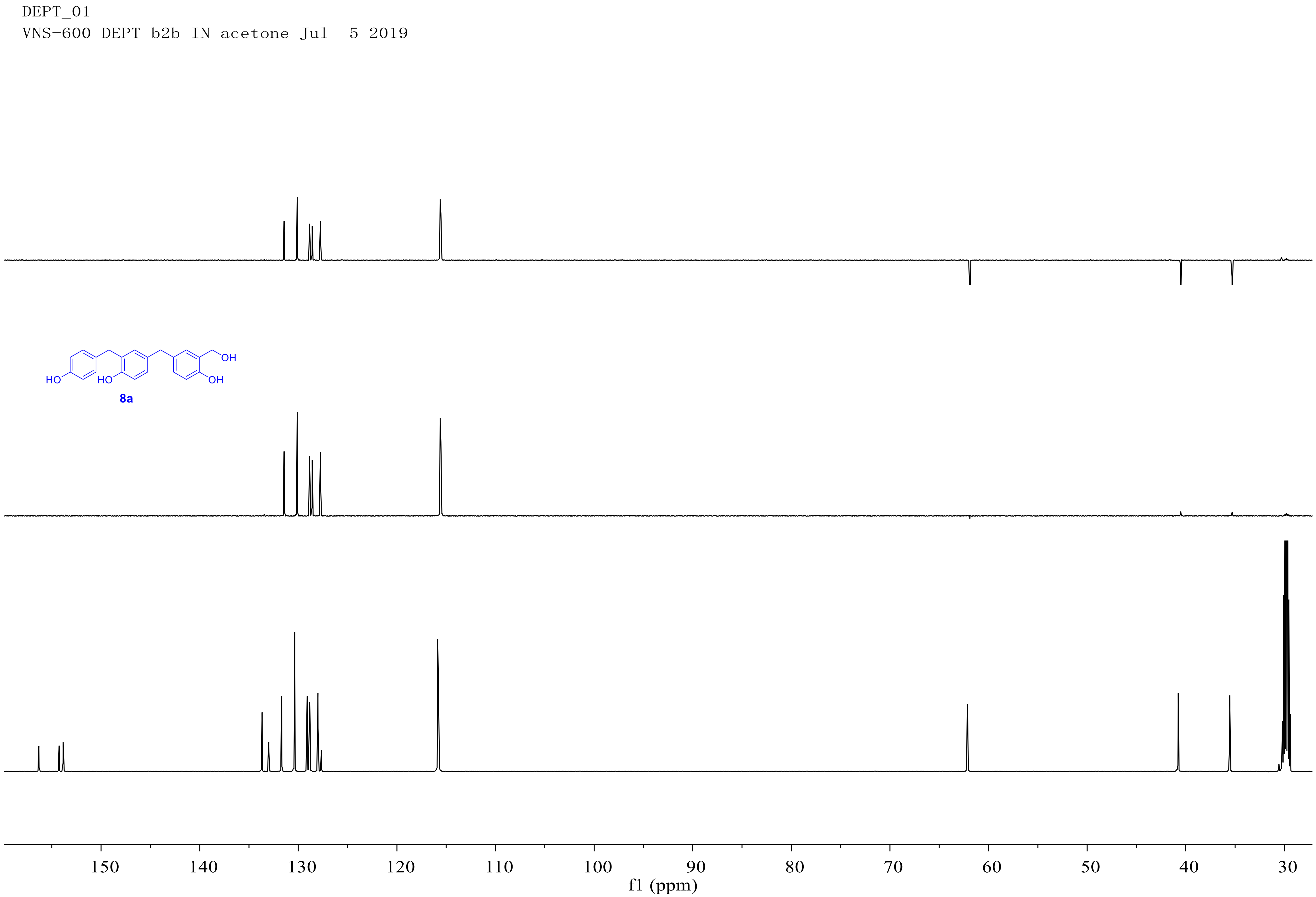

Fig. S156 The DEPT spectrum of compound **8a** in acetone-d_6 (150 MHz).

gCOSY_01
VNS-600 gCOSY b2b IN acetone Sep 16 2019

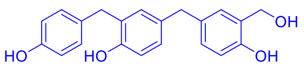

8a

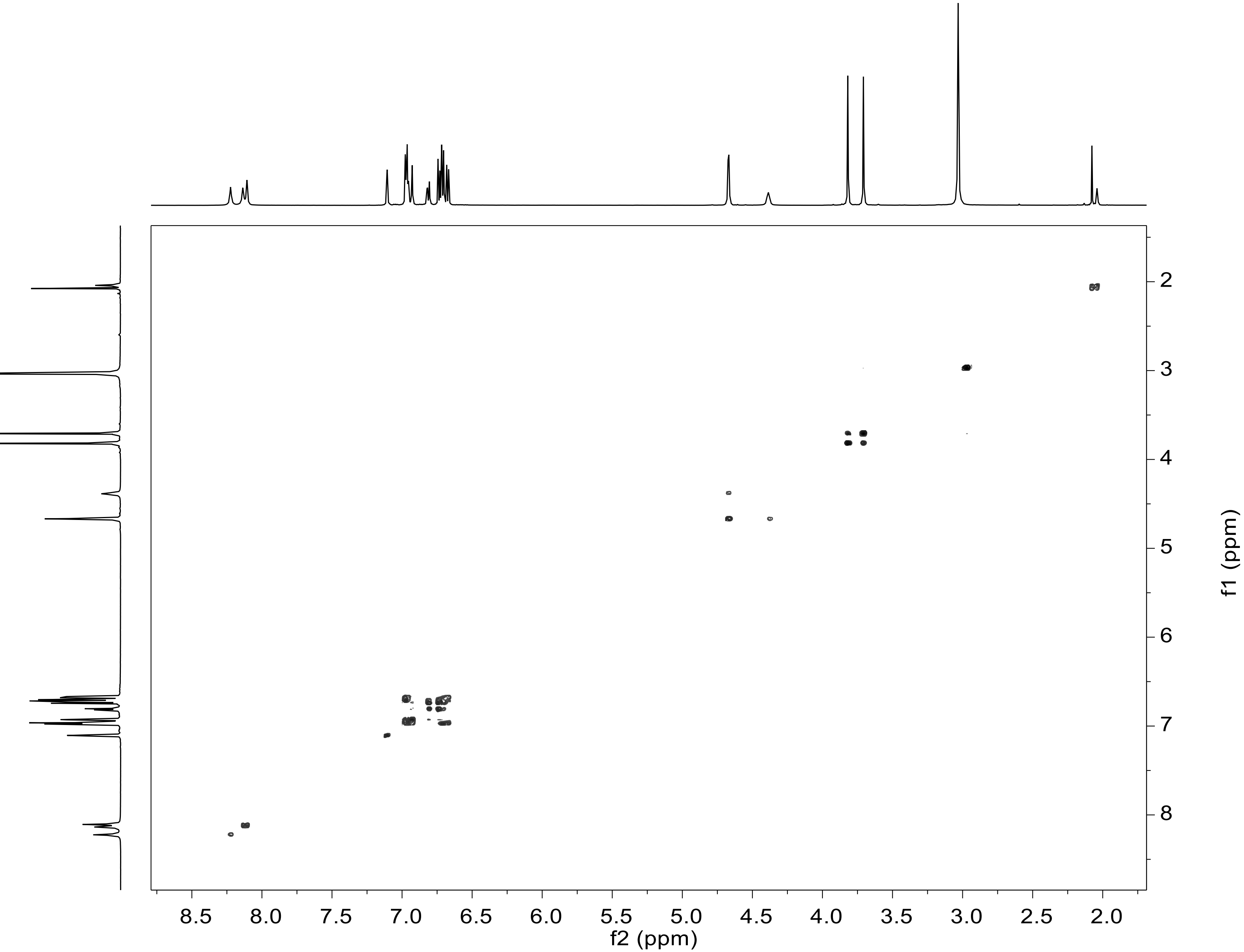

Fig. S157 The ^{1}H-^{1}H COSY spectrum of compound **8a** in acetone-d_6 (600 MHz).

gHSQCAD_01
VNS-600 gHSQCAD b2b IN acetone Oct 22 2019

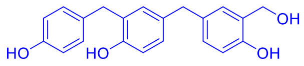

8a

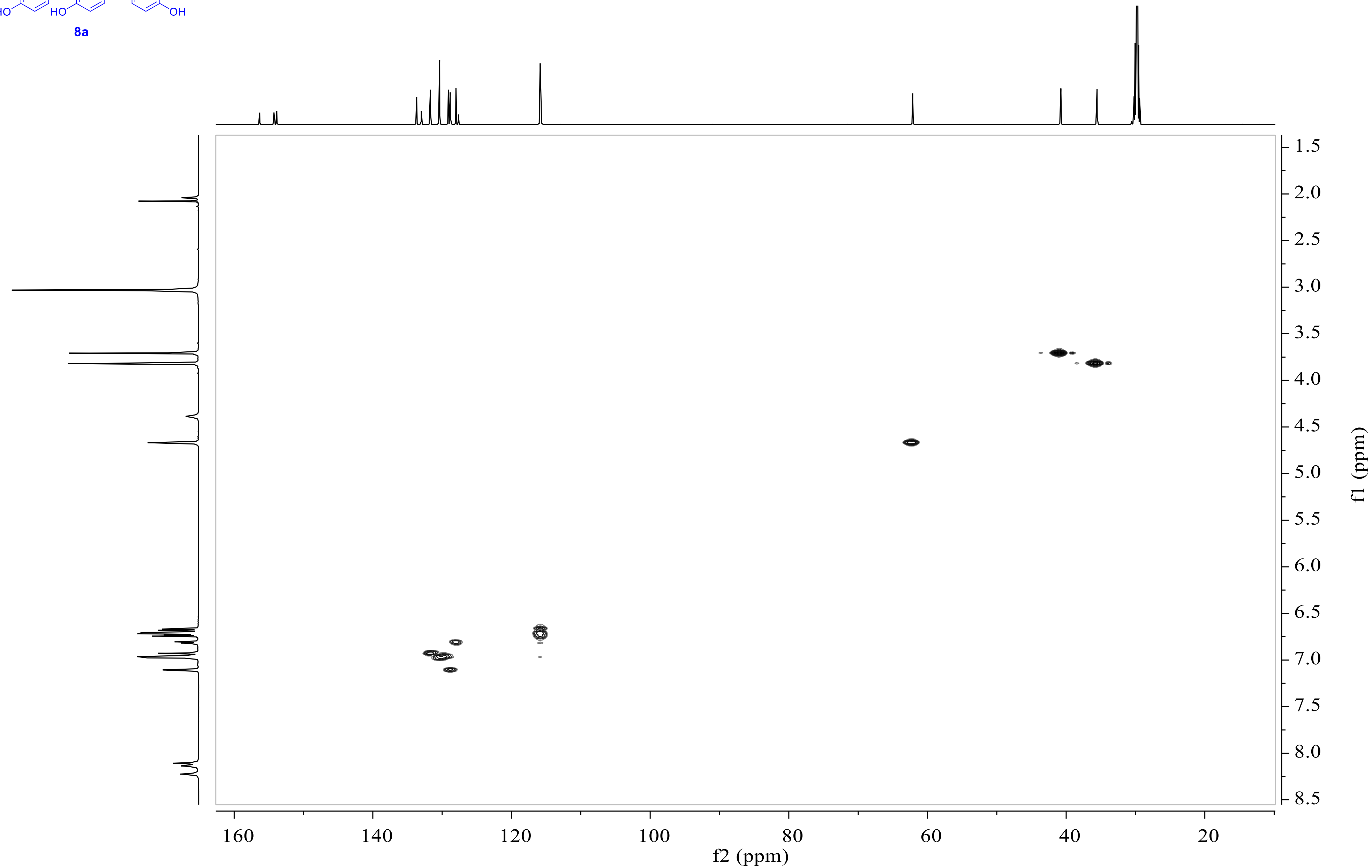

Fig. S158 The HSQC spectrum of compound **8a** in acetone-d_6 (600 MHz for ^{1}H).

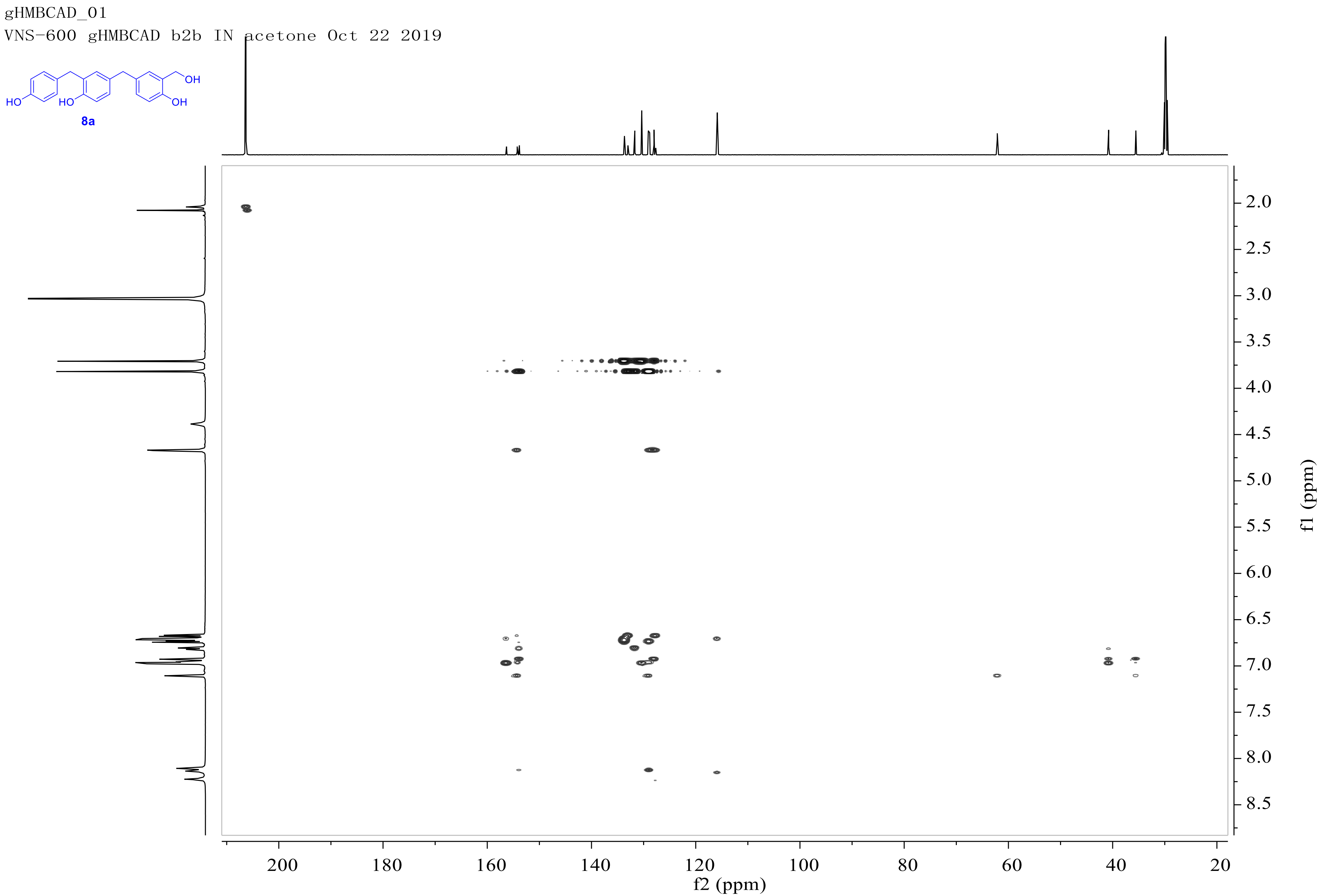

Fig. S159 The HMBC spectrum of compound **8a** in acetone-d_6 (600 MHz for ^{1}H).

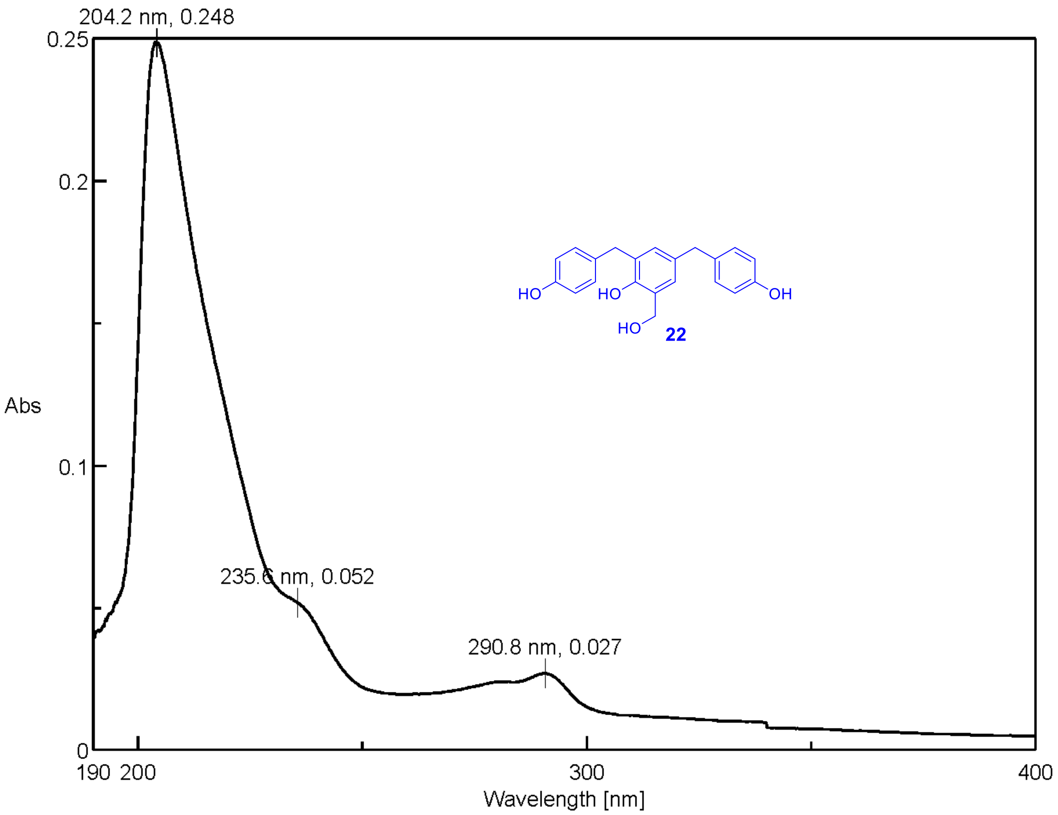

[Comment] — d3a
Sample Name H-6
Comment CH3CN
User HJW
Division UV
Company 324
[Measurement Information]
Instrument Name V-650
Model Name V-650
Serial No. A034461150

Accessory PSC-718
Accessory S/N A001761114
Position 1
Cell Length 10 mm
Temperature 19.95 C
Control Sonsor Holder
Monitor Sensor Holder
Start Mode Start immediately

Photometric Mode Abs
Measurement range 400 - 190 nm
Data pitch 0.2 nm
Band width(UV/Vis) 2.0 nm
Response Medium
Scanning speed 200 nm/min
Source Change 340 nm
Light Source D2/WI
Filter Exchange Step
Correction Baseline

[Data Information]
Creation Date 2019-11-13 11:18

Data array type Linear data array
Horizontal Wavelength [nm]
Vertical Abs
Start 400 nm
End 190 nm
Data pitch 0.2 nm
Data points 1051

Fig. S160 The UV spectrum of compound **22**.

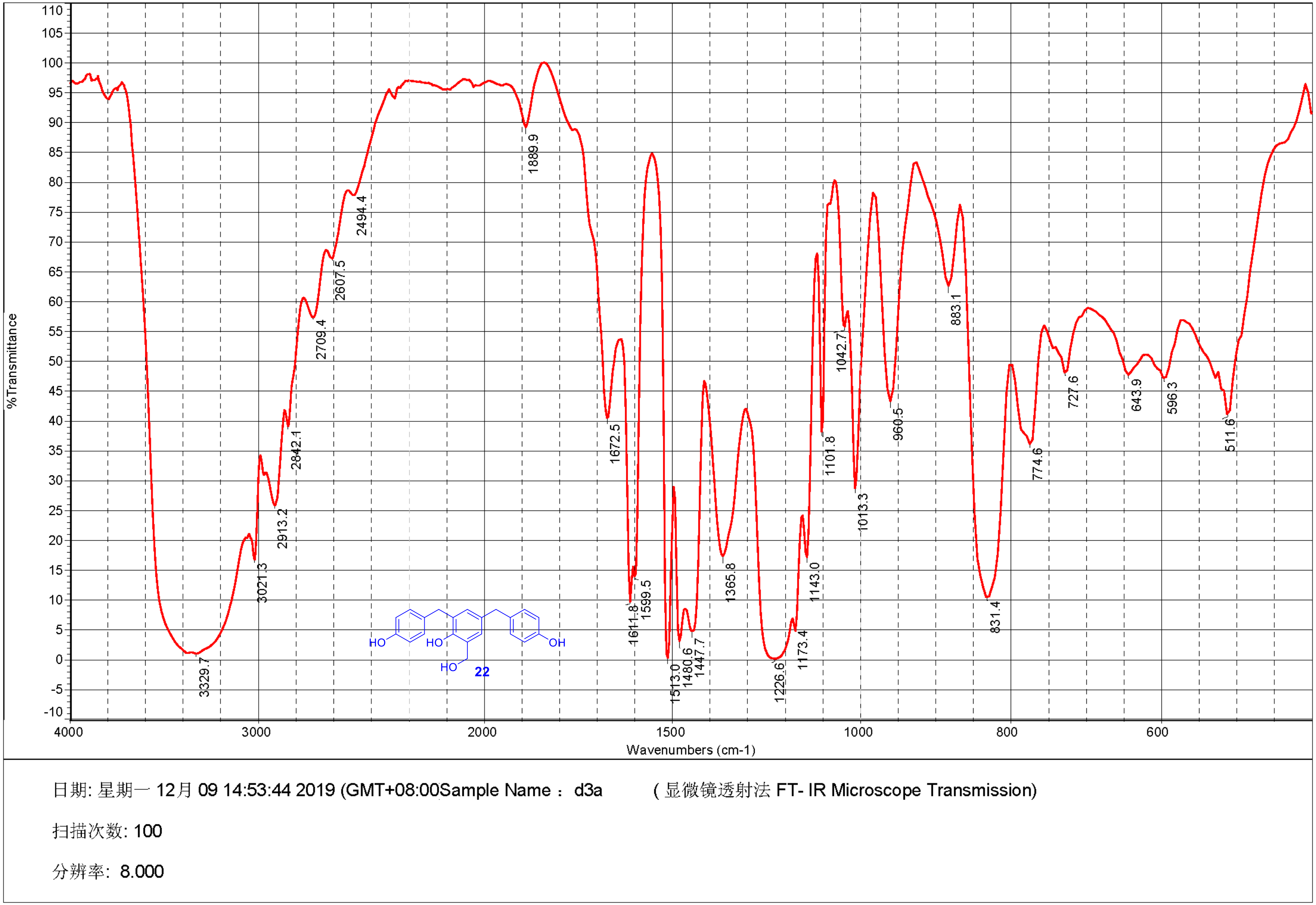

Fig. S161 The IR spectrum of compound **22**.

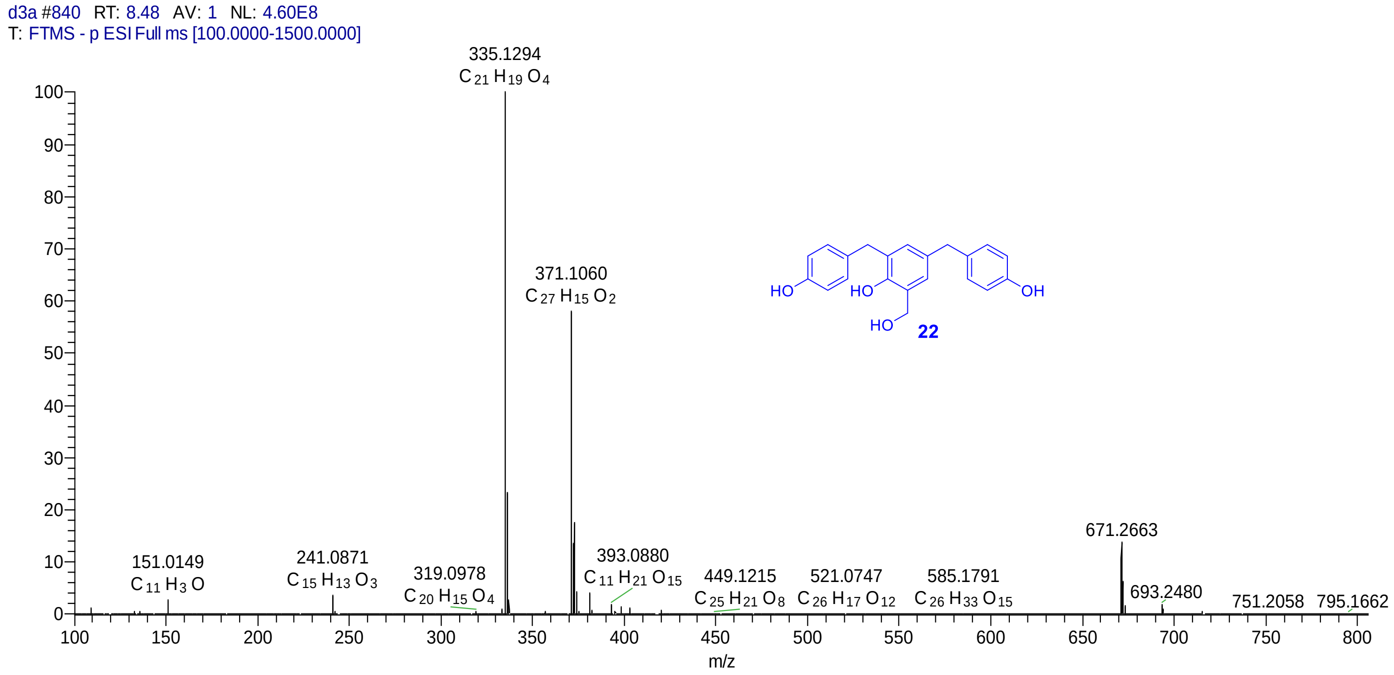

m/z	Theo. Mass	Delta (mmu)	RDB equiv.	Composition
335.1294	335.1278	1.64	12.5	C21 H19 O4
	335.1256	3.84	3.5	C15 H24 O6 Cl
	335.1337	-4.23	3.5	C14 H23 O9
	335.1197	9.71	12.5	C22 H20 O Cl
	335.1184	11.03	-0.5	C10 H23 O12
	335.1408	-11.42	7.5	C19 H24 O3 Cl
	335.1125	16.9	8.5	C17 H19 O7
	335.1103	19.09	-0.5	C11 H24 O9 Cl
	335.1489	-19.48	7.5	C18 H23 O6
	335.1045	24.97	8.5	C18 H20 O4 Cl
m/z	Theo. Mass	Delta (mmu)	RDB equiv.	Composition
371.1060	371.1045	1.53	11.5	C21 H20 O4 Cl
	371.1103	-4.35	2.5	C14 H24 O9 Cl
	371.1125	-6.54	11.5	C20 H19 O7
	371.0973	8.72	7.5	C16 H19 O10
	371.1184	-12.41	2.5	C13 H23 O12
	371.0892	16.78	7.5	C17 H20 O7 Cl
	371.1256	-19.6	6.5	C18 H24 O6 Cl
	371.0820	23.97	3.5	C12 H19 O13
	371.1337	-27.67	6.5	C17 H23 O9
	371.0761	29.85	12.5	C19 H15 O8

Fig. S162 The (–)-HR-ESI-MS report of compound **22**.

PROTON_01
VNS-600 PROTON d3a IN acetone Sep 17 2019

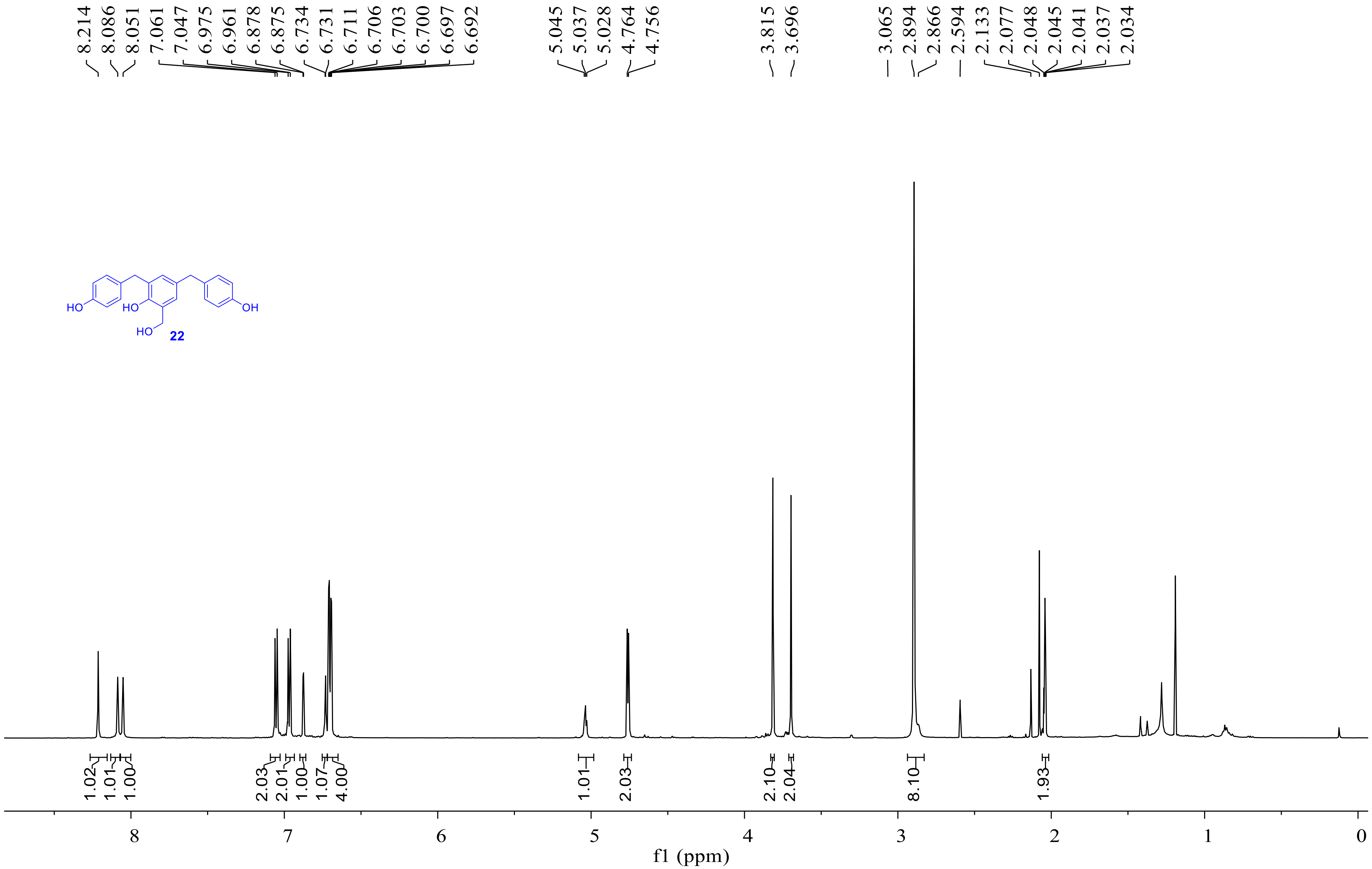

Fig. S163 The ^{1}H NMR spectrum of compound **22** in acetone-d_6 (600 MHz).

CARBON_01
VNS-600 CARBON d3a IN acetone Oct 17 2019

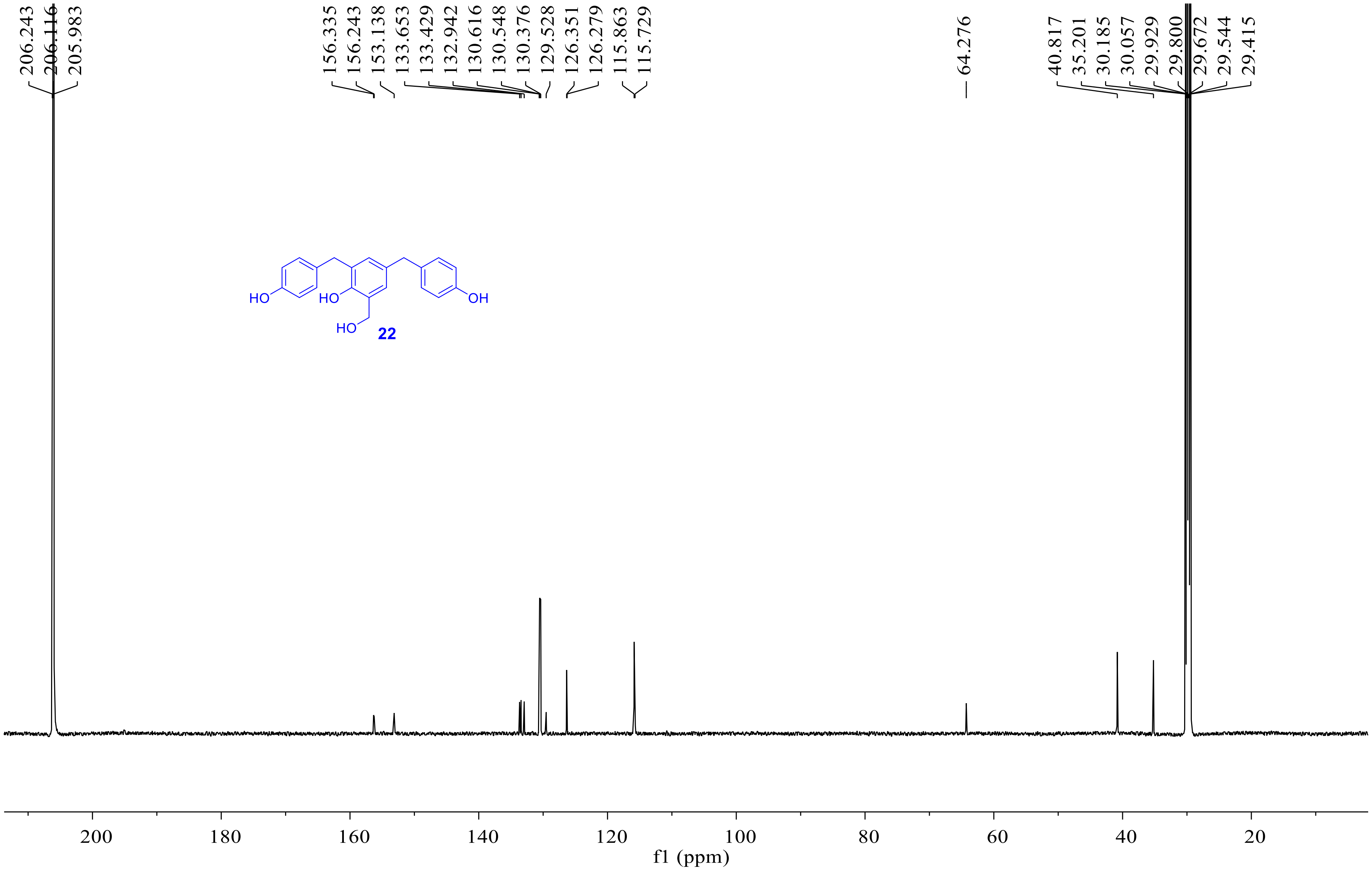

Fig. S164 The ^{13}C NMR spectrum of compound **22** in acetone-d_6 (150 MHz).

DEPT_01
VNS-600 DEPT d3a IN acetone Oct 17 2019

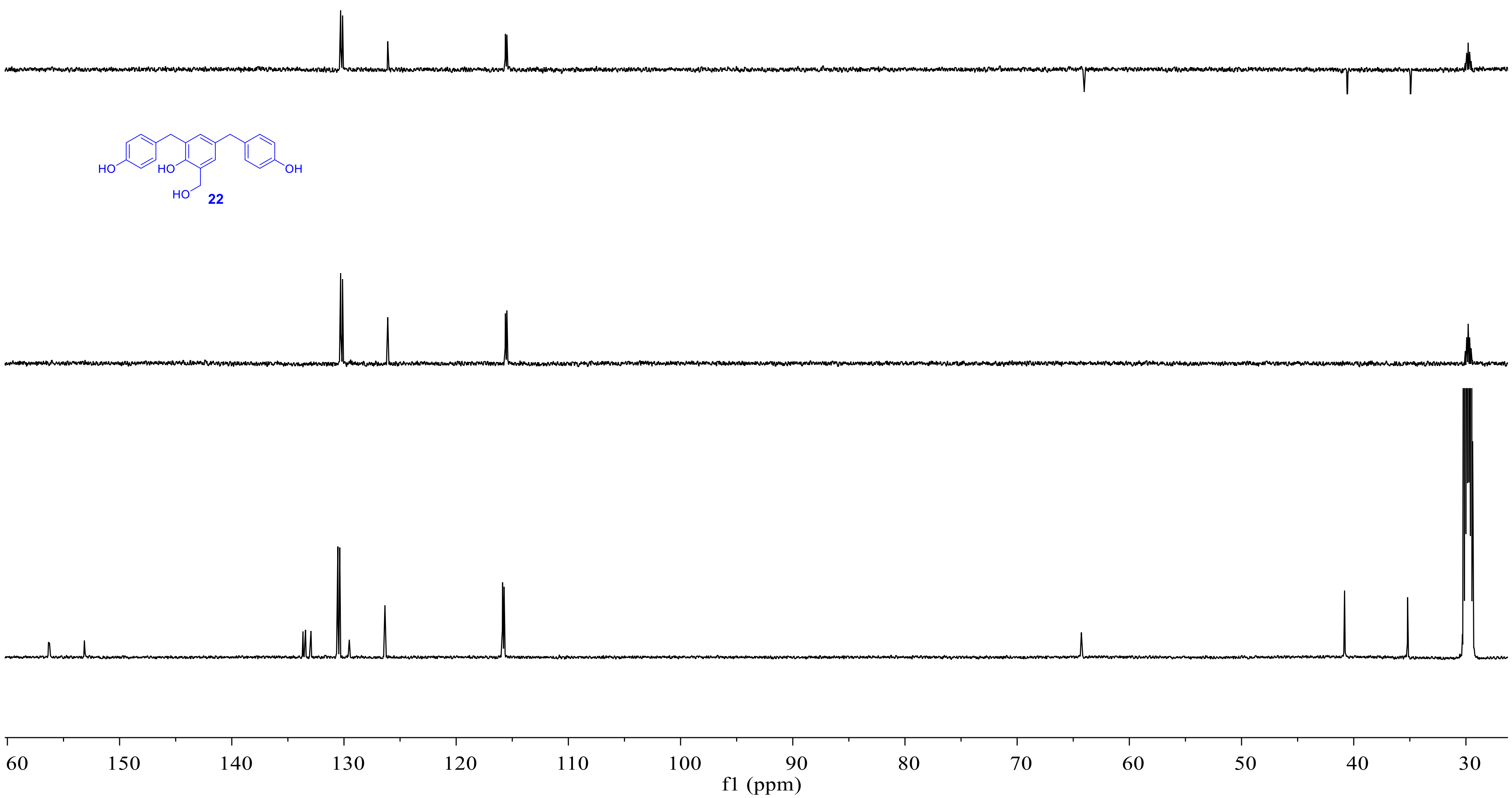

Fig. S165 The DEPT spectrum of compound **22** in acetone-d_6 (150 MHz).

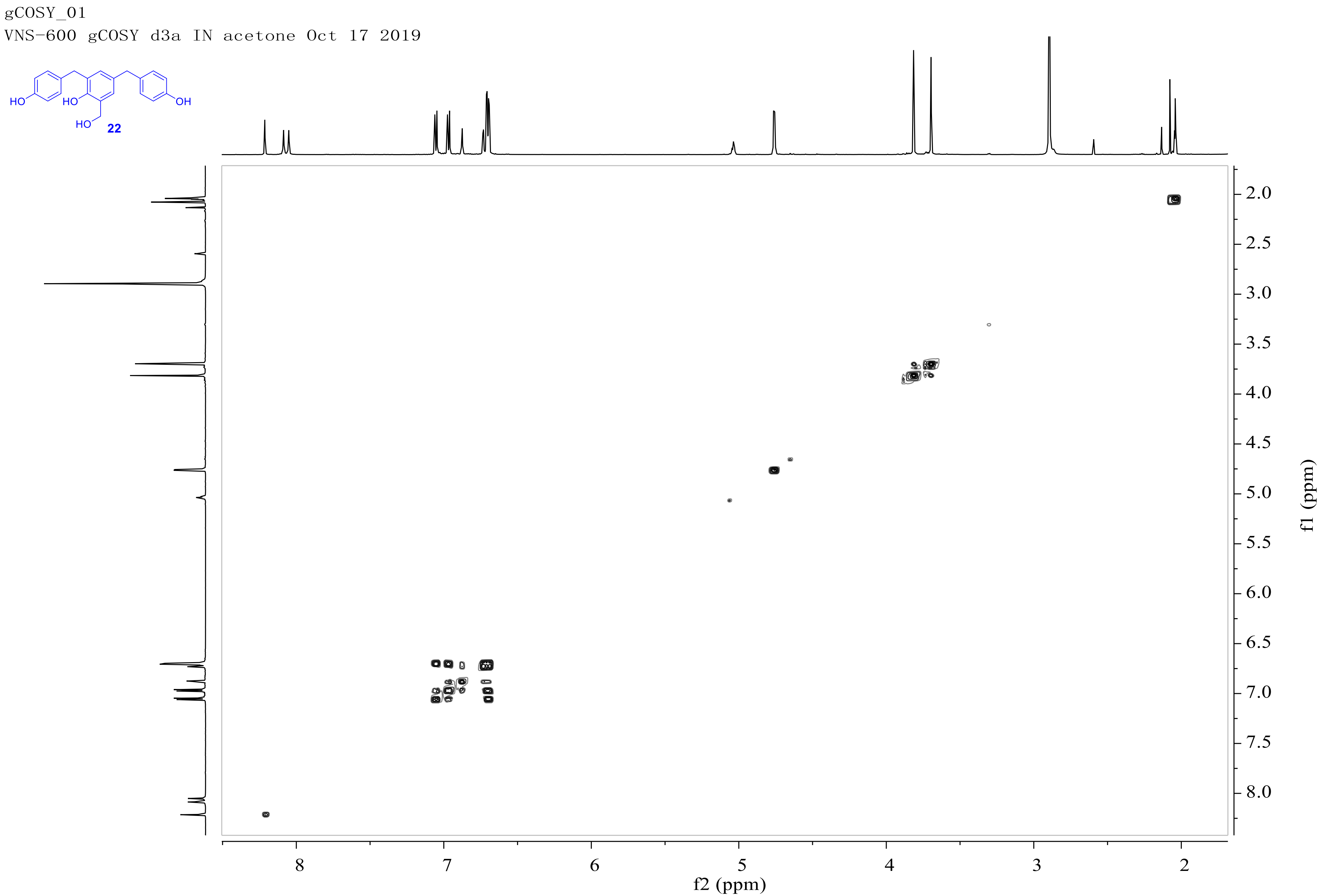

Fig. S166 The ^{1}H-^{1}H COSY spectrum of compound **22** in acetone-d_6 (600 MHz).

gHSQCAD_01
VNS-600 gHSQCAD d3a IN acetone Oct 17 2019

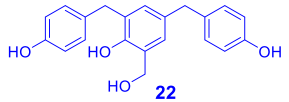

22

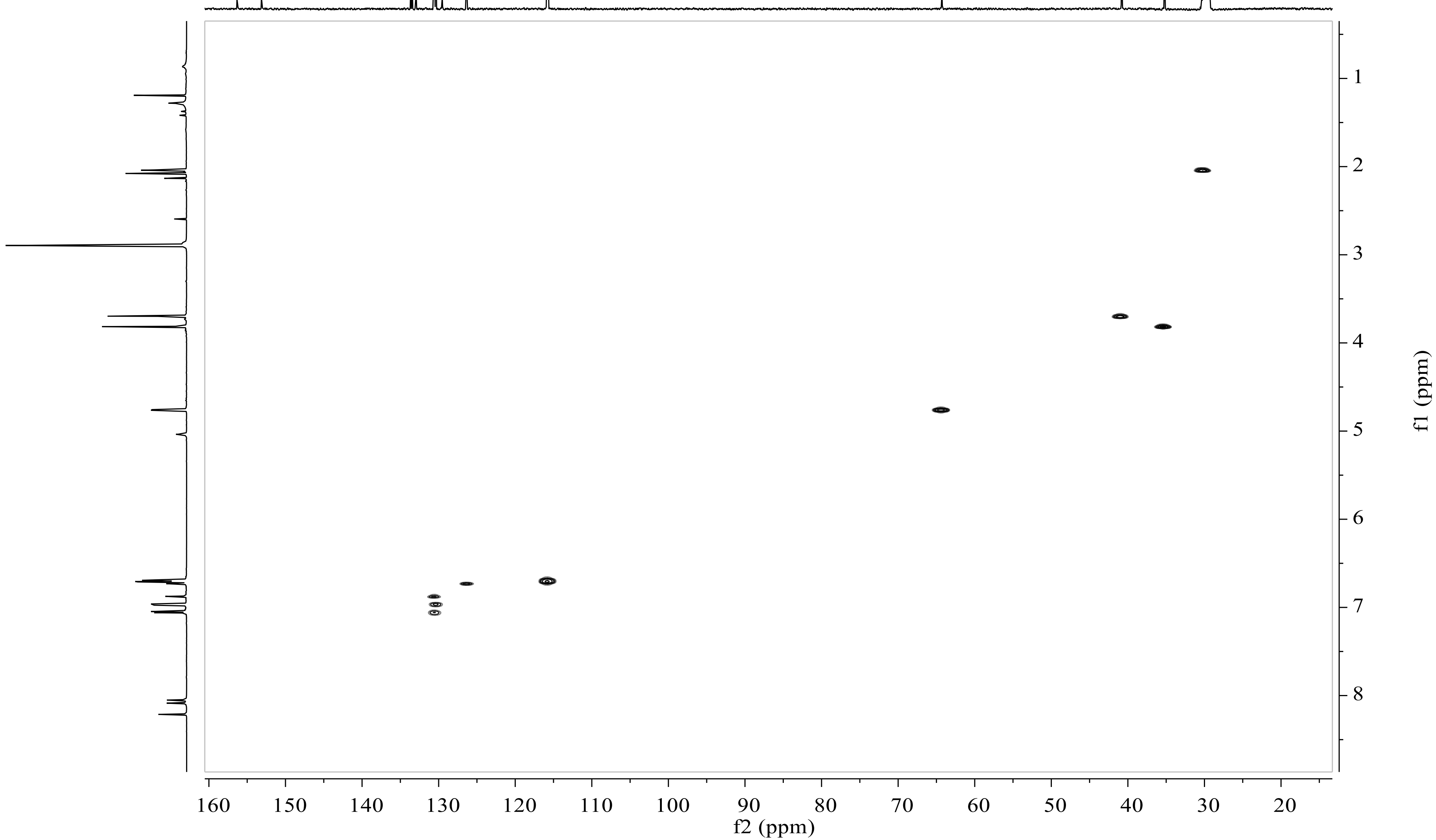

Fig. S167 The HSQC spectrum of compound **22** in acetone-d_6 (600 MHz for ^{1}H).

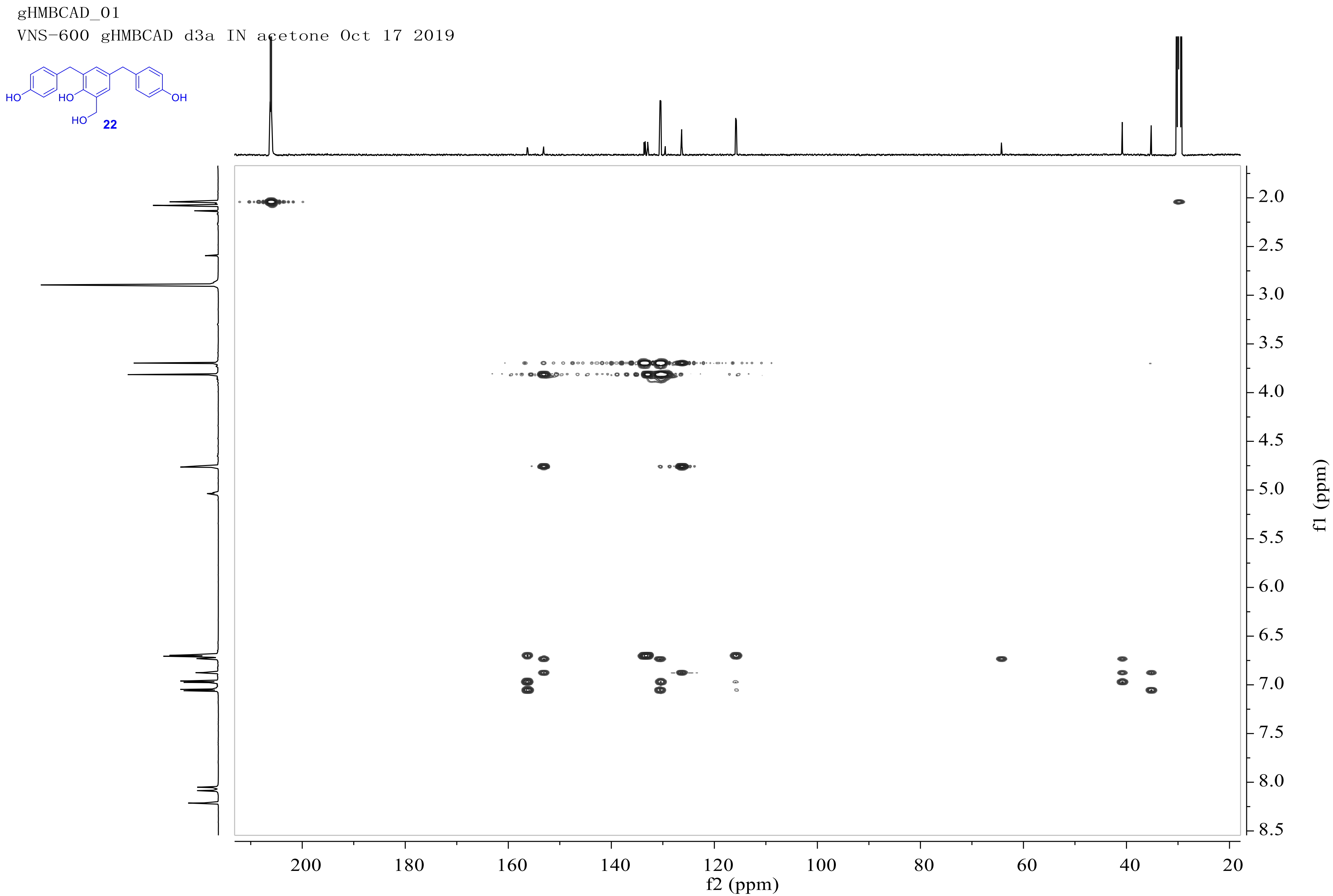

Fig. S168 The HMBC spectrum of compound **22** in acetone-d_6 (600 MHz for ^{1}H).

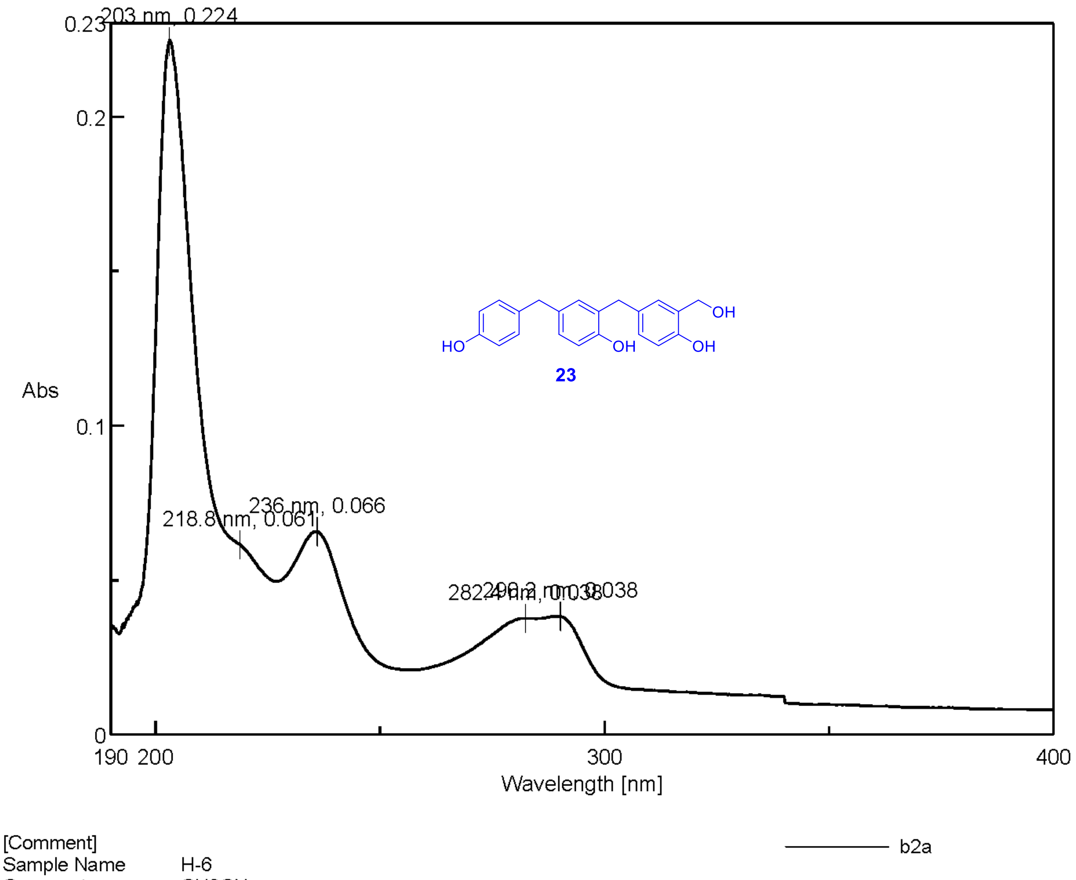

[Comment]
Sample Name H-6
Comment CH3CN
User HJW
Division UV
Company 324
[Measurement Information]
Instrument Name V-650
Model Name V-650
Serial No. A034461150

Accessory PSC-718
Accessory S/N A001761114
Position 1
Cell Length 10 mm
Temperature 19.95 C
Control Sonsor Holder
Monitor Sensor Holder
Start Mode Start immediately

Photometric Mode Abs
Measurement range 400 - 190 nm
Data pitch 0.2 nm
Band width(UV/Vis) 2.0 nm
Response Medium
Scanning speed 200 nm/min
Source Change 340 nm
Light Source D2/WI
Filter Exchange Step
Correction Baseline

[Data Information]
Creation Date 2019-11-13 11:25

Data array type Linear data array
Horizontal Wavelength [nm]
Vertical Abs
Start 400 nm
End 190 nm
Data pitch 0.2 nm
Data points 1051

Fig. S169 The UV spectrum of compound **23**.

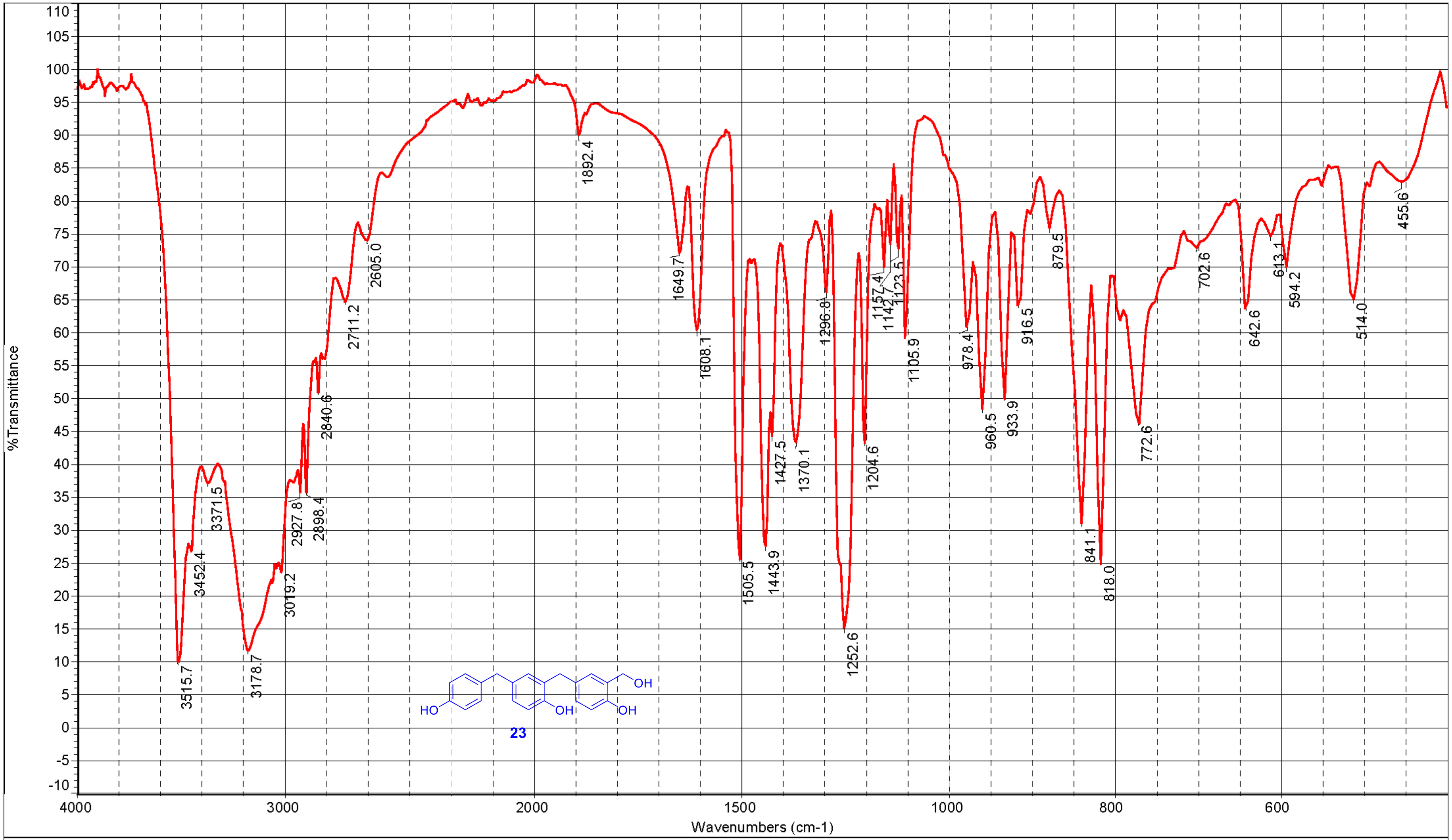

日期: 星期一 12月 09 14:28:19 2019 (GMT+08:00Sample Name： b2a (显微镜透射法 FT- IR Microscope Transmission)

扫描次数: 100

分辨率: 8.000

Fig. S170 The IR spectrum of compound **23**.

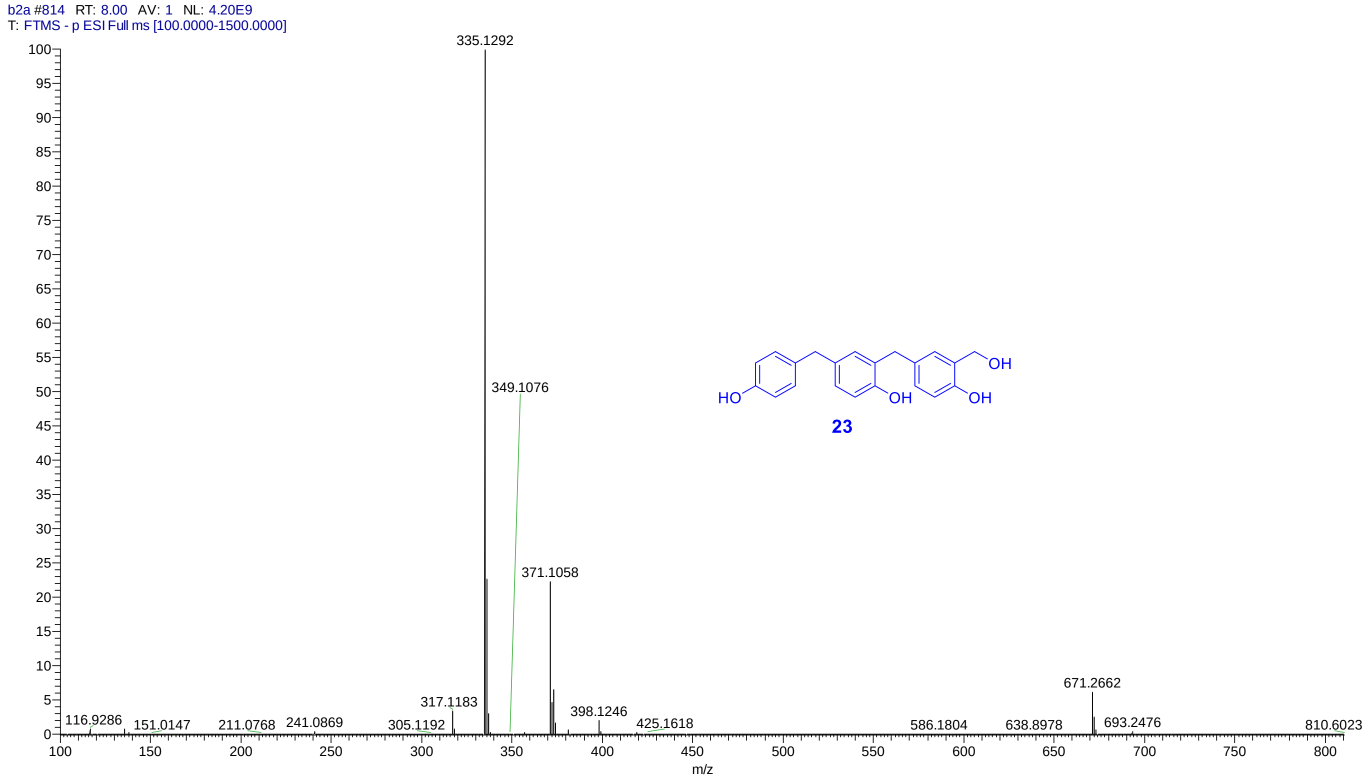

m/z	Theo. Mass	Delta (mmu)	RDB equiv.	Composition
335.1293	335.1278	1.51	12.5	C21 H19 O4
	335.1256	3.71	3.5	C15 H24 O6 Cl
	335.1337	-4.36	3.5	C14 H23 O9
	335.1197	9.58	12.5	C22 H20 O Cl
	335.1184	10.9	-0.5	C10 H23 O12
	335.1408	-11.55	7.5	C19 H24 O3 Cl
	335.1125	16.77	8.5	C17 H19 O7
	335.1103	18.96	-0.5	C11 H24 O9 Cl
	335.1489	-19.61	7.5	C18 H23 O6
	335.1045	24.84	8.5	C18 H20 O4 Cl
**m/z**	**Theo. Mass**	**Delta (mmu)**	**RDB equiv.**	**Composition**
371.1059	371.1045	1.41	11.5	C21 H20 O4 Cl
	371.1103	-4.47	2.5	C14 H24 O9 Cl
	371.1125	-6.66	11.5	C20 H19 O7
	371.0973	8.6	7.5	C16 H19 O10
	371.1184	-12.53	2.5	C13 H23 O12
	371.0892	16.66	7.5	C17 H20 O7 Cl
	371.1256	-19.72	6.5	C18 H24 O6 Cl
	371.0820	23.85	3.5	C12 H19 O13
	371.1337	-27.79	6.5	C17 H23 O9
	371.0761	29.73	12.5	C19 H15 O8

Fig. S171 The (–)-HR-ESI-MS report of compound **23**.

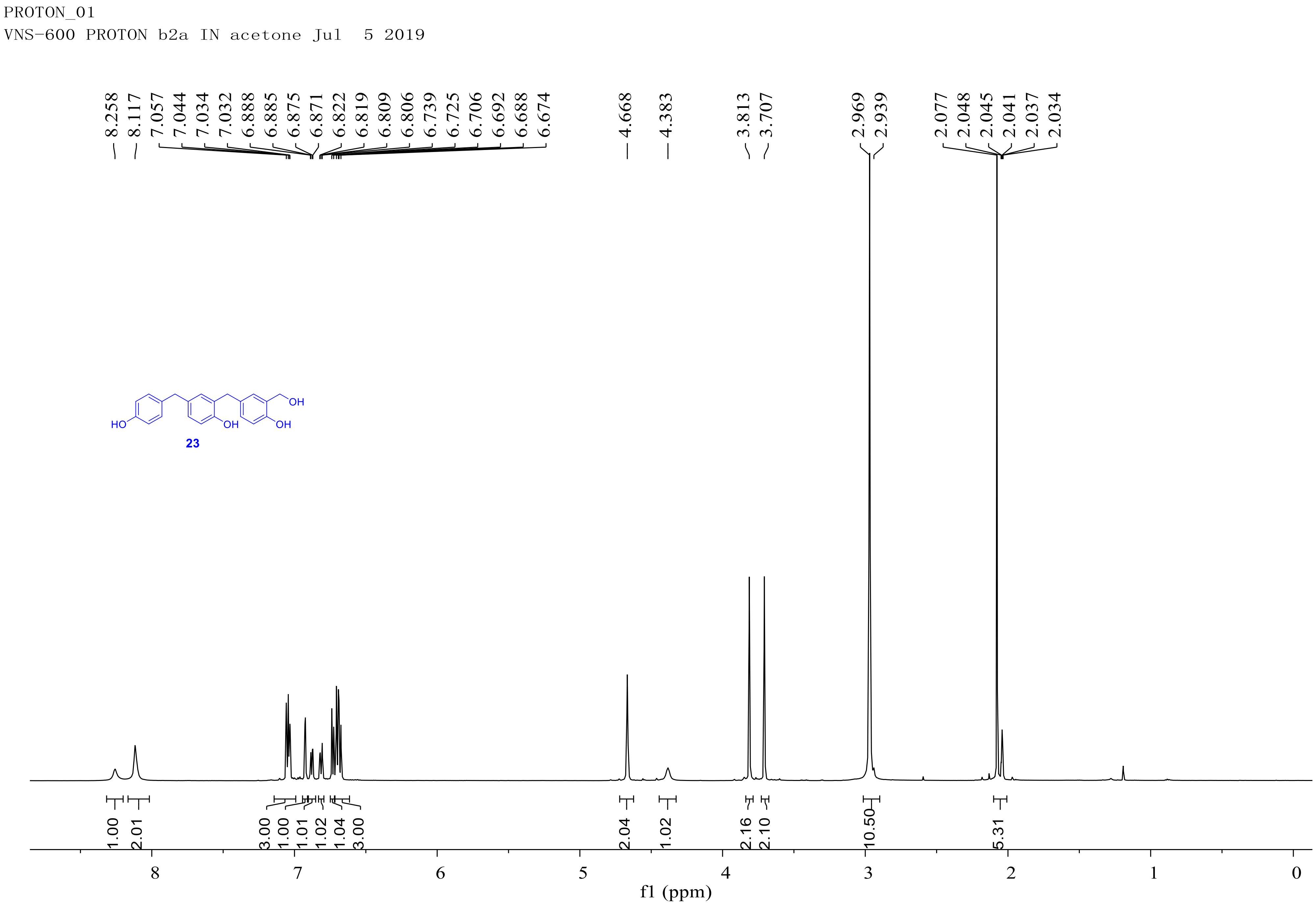

Fig. S172 The ^{1}H NMR spectrum of compound **23** in acetone-d_6 (600 MHz).

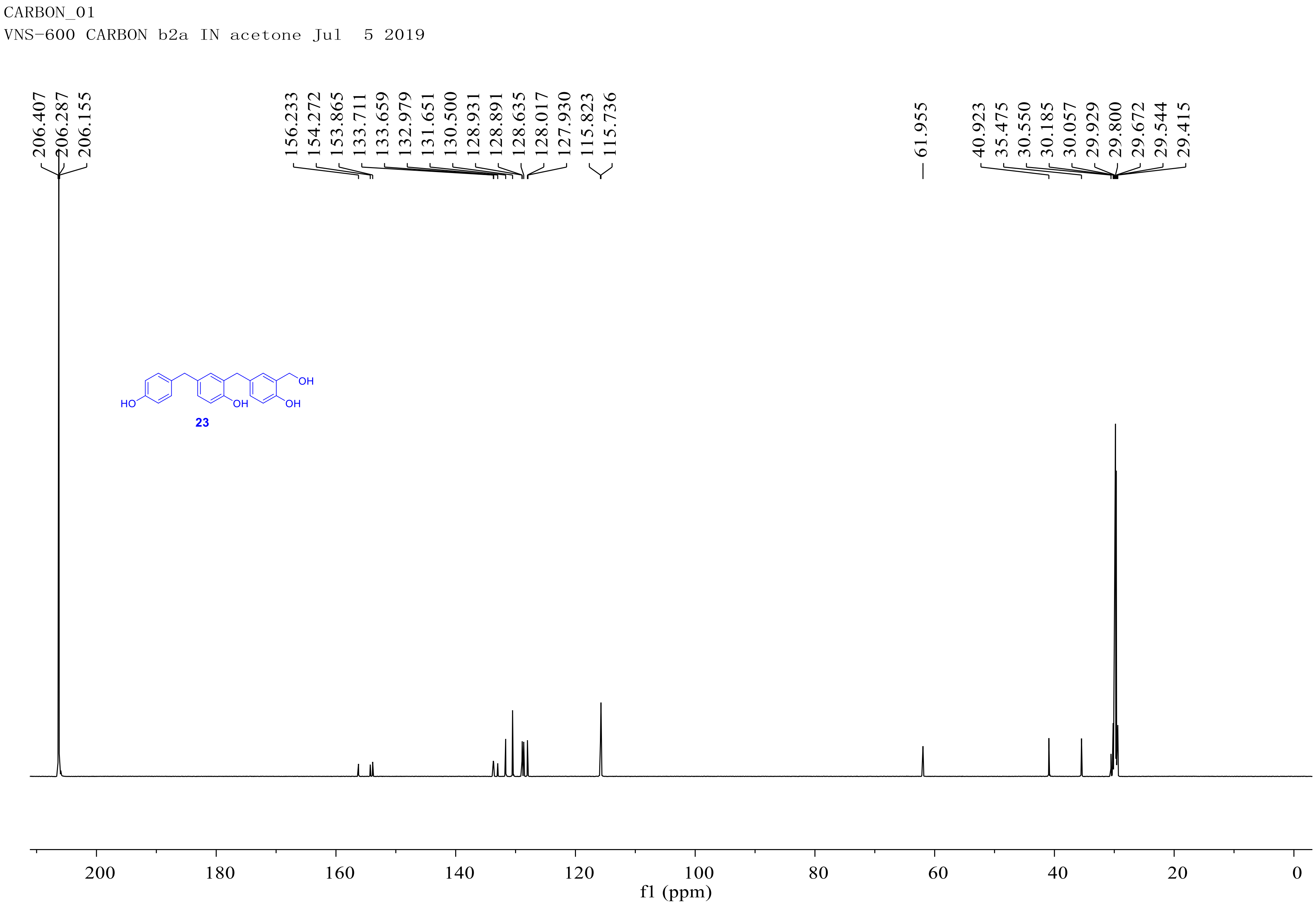

Fig. S173 The ^{13}C NMR spectrum of compound **23** in acetone-d_6 (150 MHz).

CARBON_01
VNS-600 CARBON b2a IN acetone Jul 5 2019

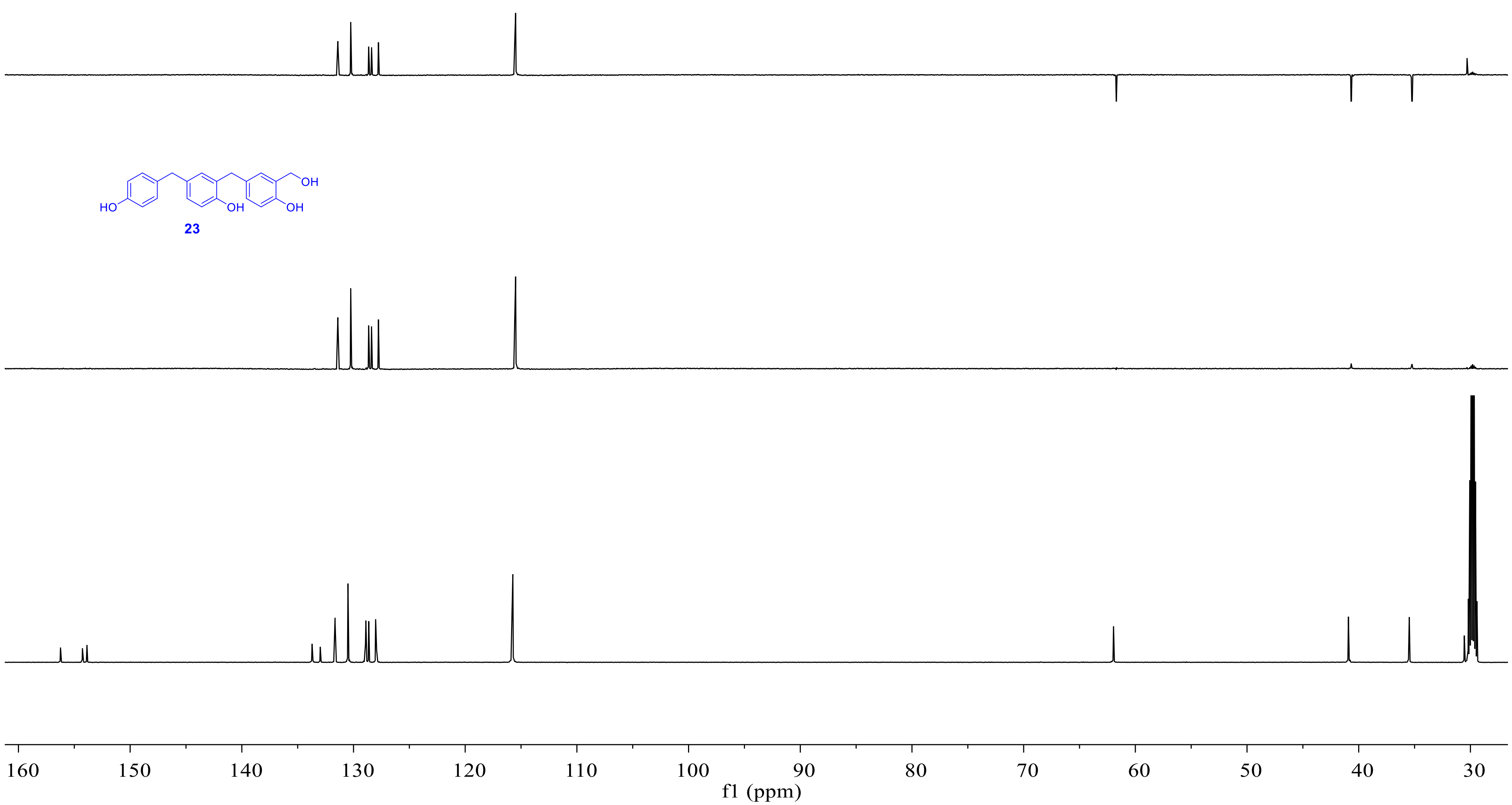

Fig. S174 The DEPT spectrum of compound **23** in acetone-d_6 (150 MHz).

gCOSY_01
VNS-600 gCOSY b2a IN acetone Sep 16 2019

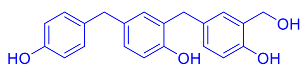
23

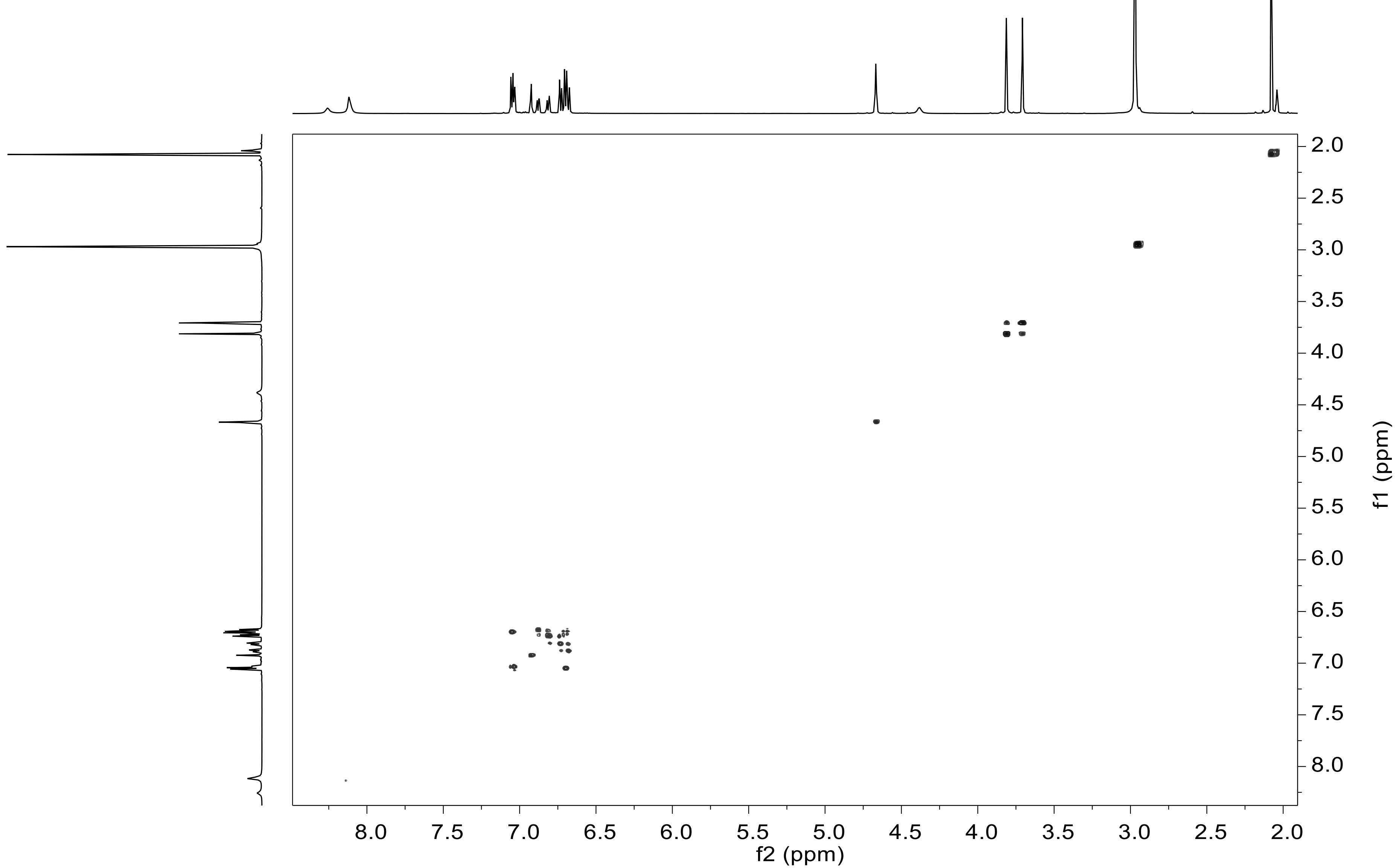

Fig. S175 The ^{1}H-^{1}H COSY spectrum of compound **23** in acetone-d_6 (600 MHz).

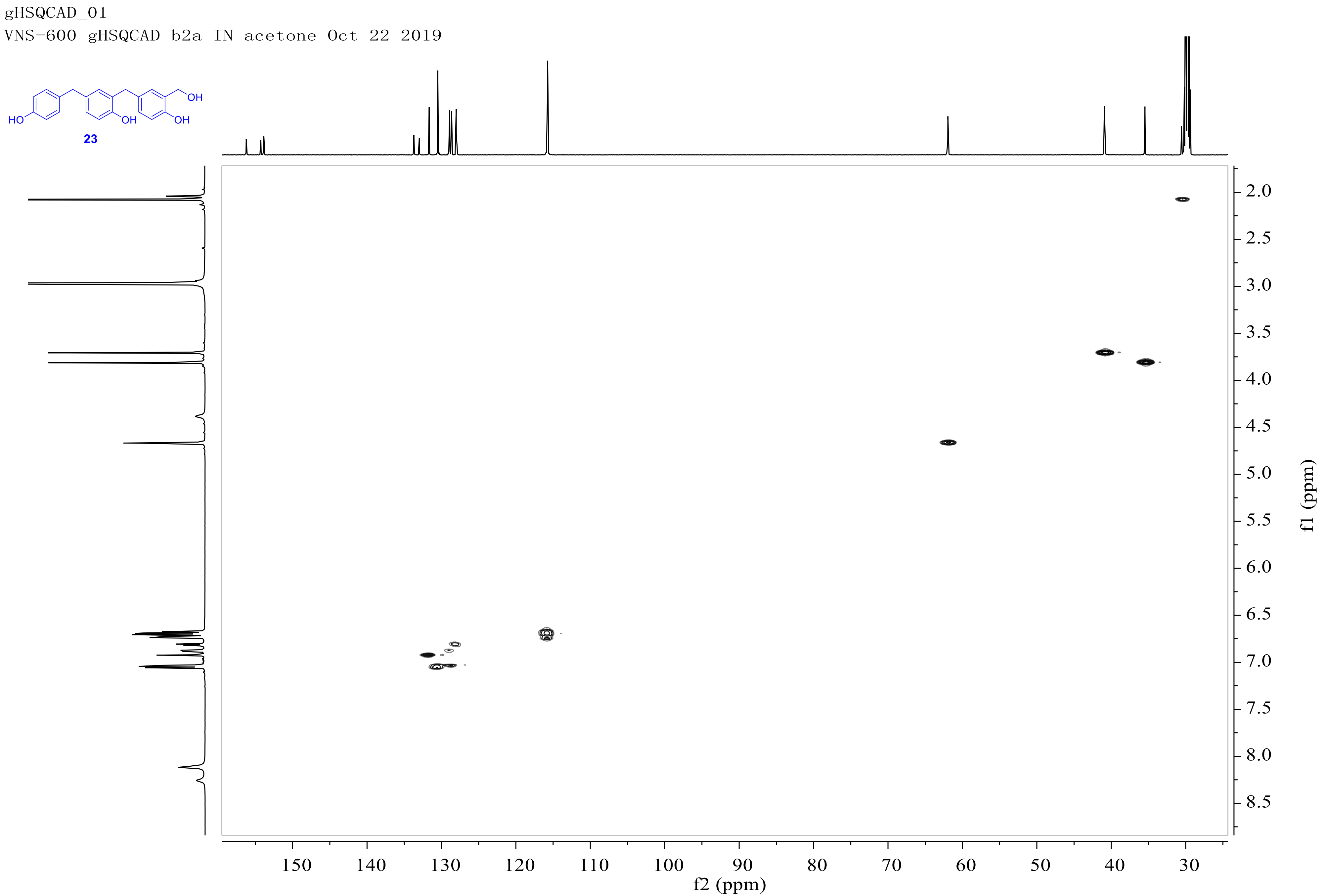

Fig. S176 The HSQC spectrum of compound **23** in acetone-d_6 (600 MHz for ^{1}H).

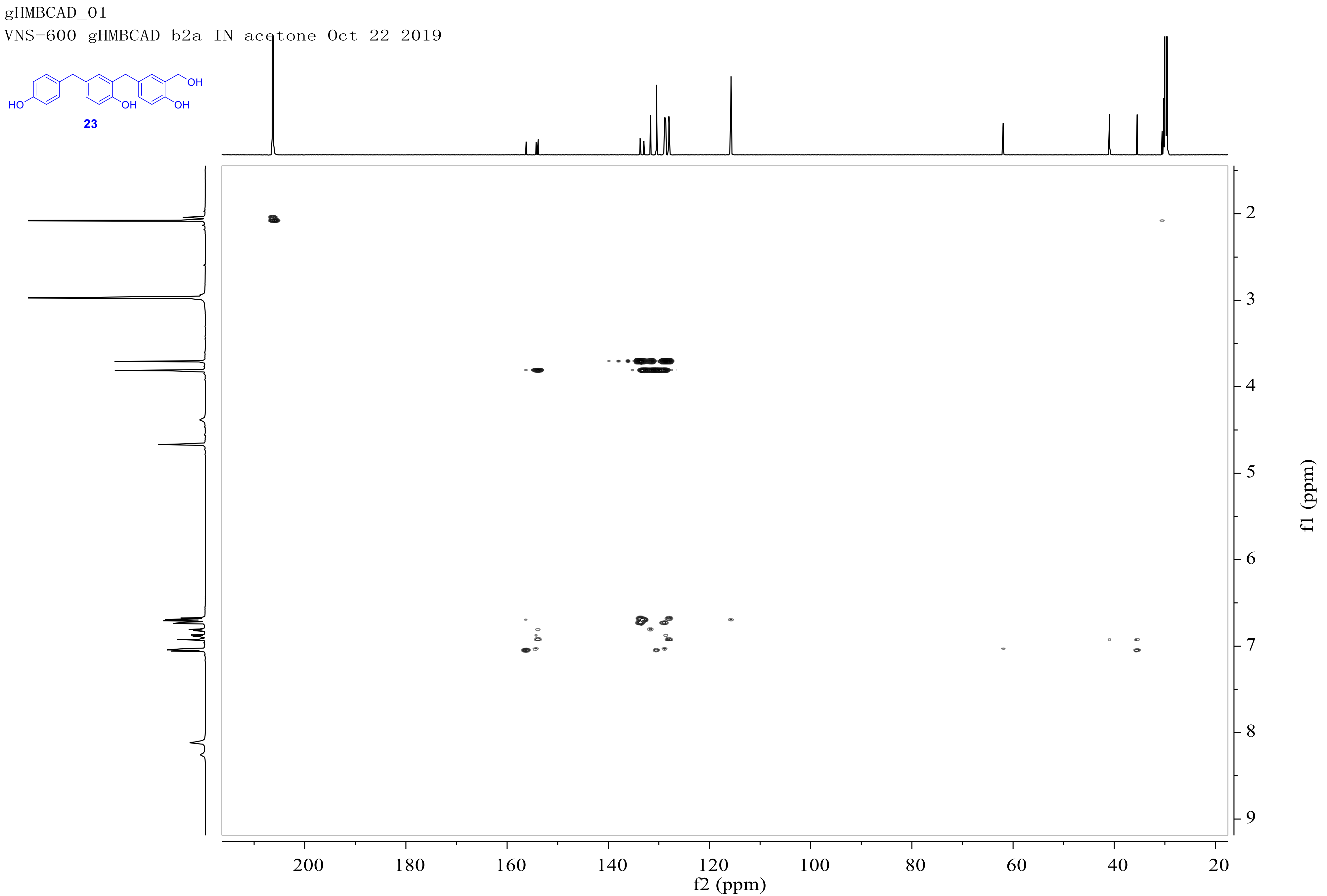

Fig. S177 The HMBC spectrum of compound **23** in acetone-d_6 (600 MHz for ^{1}H).

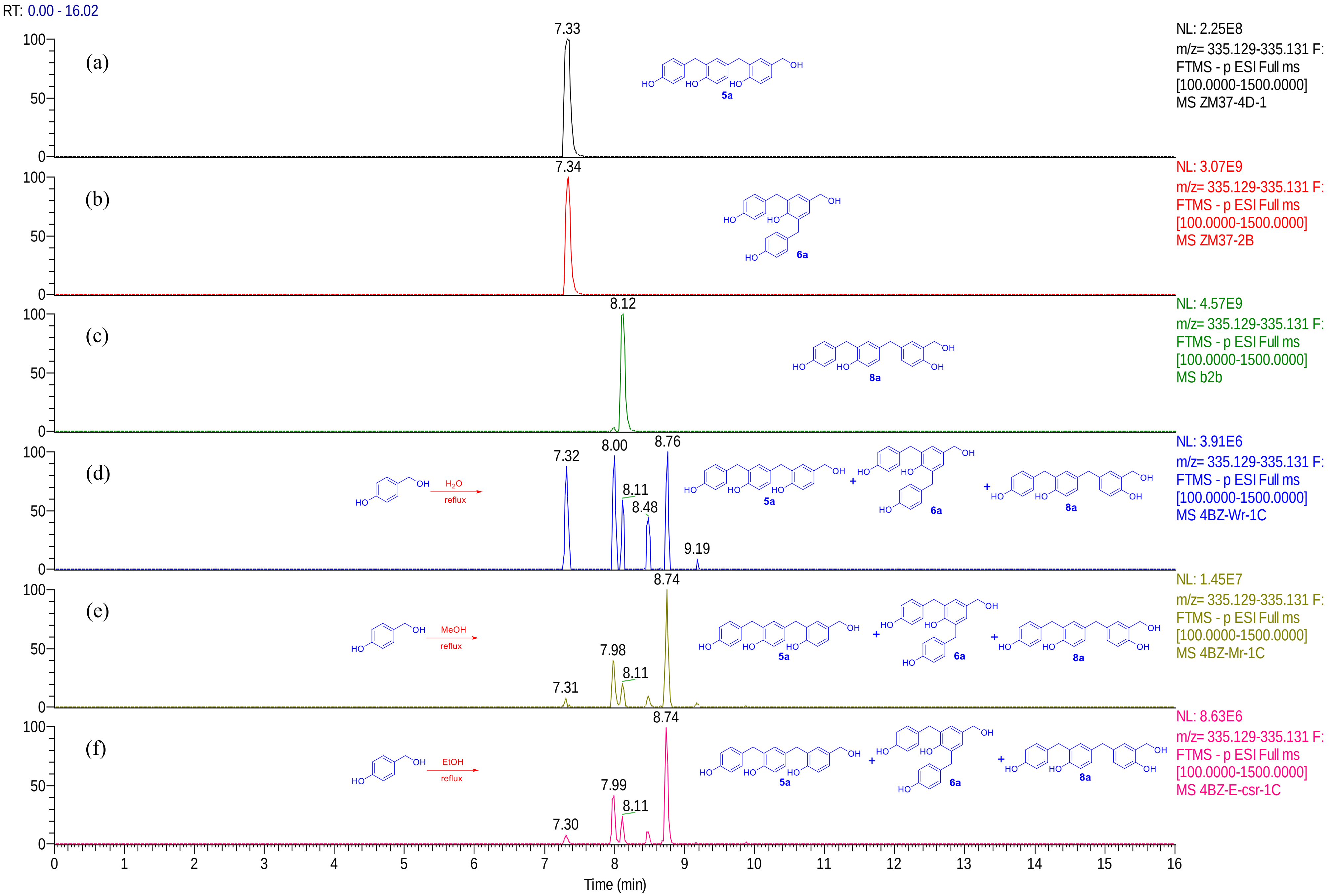

Fig. S178 Overlaid chromatograms of the extracted negative ion at *m/z* 335.130 $[M{-}H]^-$: (a)–(c) compounds **5a**, **6a**, and **8a** in CH_3CN, respectively; (d)–(f) H_2O, MeOH, and EtOH solutions of *p*-hydroxybenzyl alcohol were sonicated for 0.5 h then refluxed for 1.0 h, respectively.

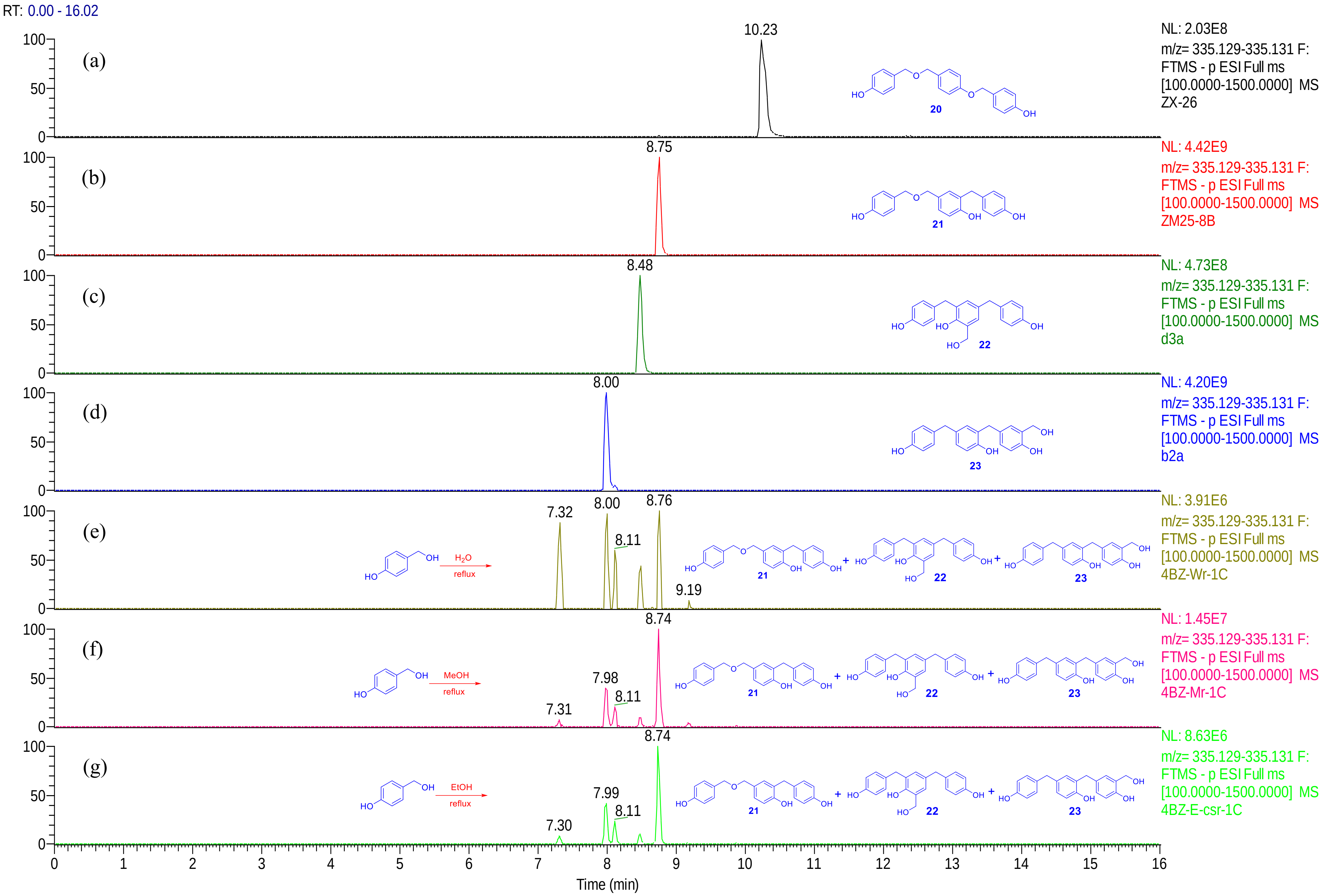

Fig. S179 Overlaid chromatograms of the extracted negative ion at *m/z* 335.130 [M–H]$^-$: (a)–(d) compounds **20**, **21**, **22**, and **23** in CH_3CN, respectively; (e)–(g) H_2O, MeOH, and EtOH solutions of *p*-hydroxybenzyl alcohol were sonicated for 0.5 h then refluxed for 1.0 h, respectively.

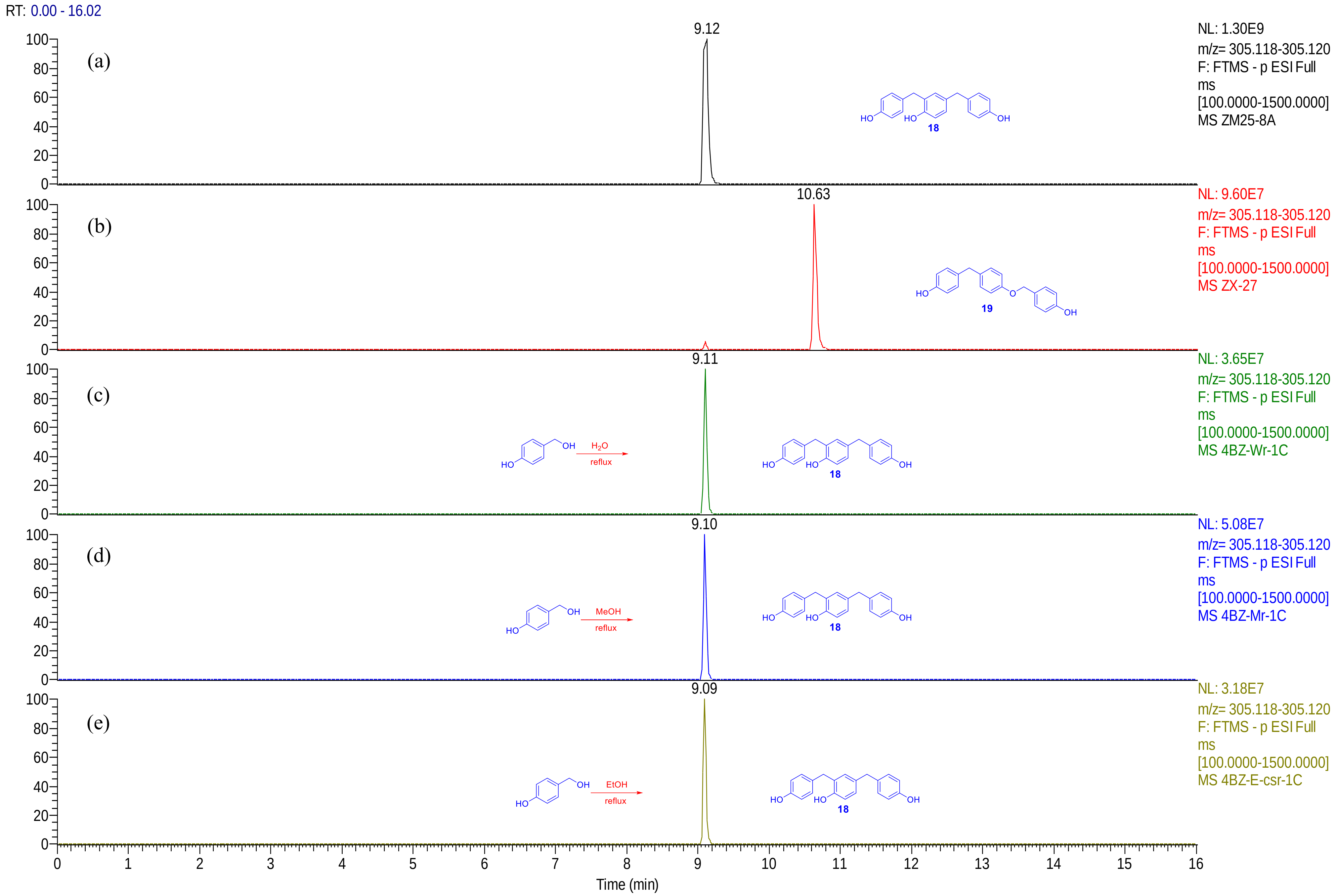

Fig. S180 Overlaid chromatograms of the extracted negative ion at *m/z* 305.119 [M–H]⁻: (a) and (b) compounds **18** and **19** in CH_3CN, respectively; (c)–(e) H_2O, MeOH, and EtOH solutions of *p*-hydroxybenzyl alcohol were sonicated for 0.5 h then refluxed for 1.0 h, respectively.

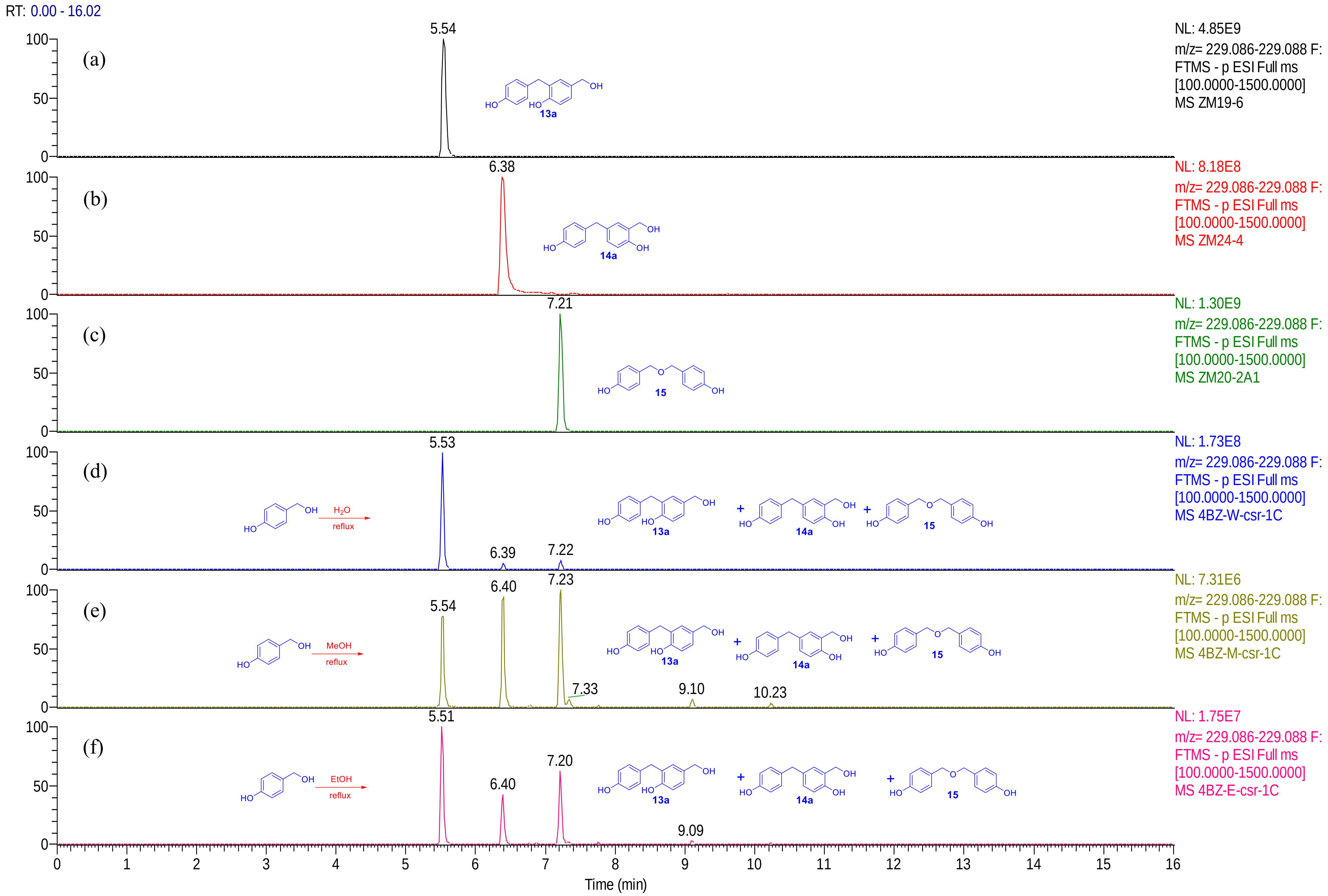

Fig. S181 Overlaid chromatograms of the extracted negative ion at *m/z* 229.087 $[M–H]^-$: (a)–(c) compounds **13a**, **14a**, and **15** in CH_3CN, respectively; (d)–(f) H_2O, MeOH, and EtOH solutions of *p*-hydroxybenzyl alcohol were sonicated for 0.5 h then refluxed for 1.0 h, respectively.

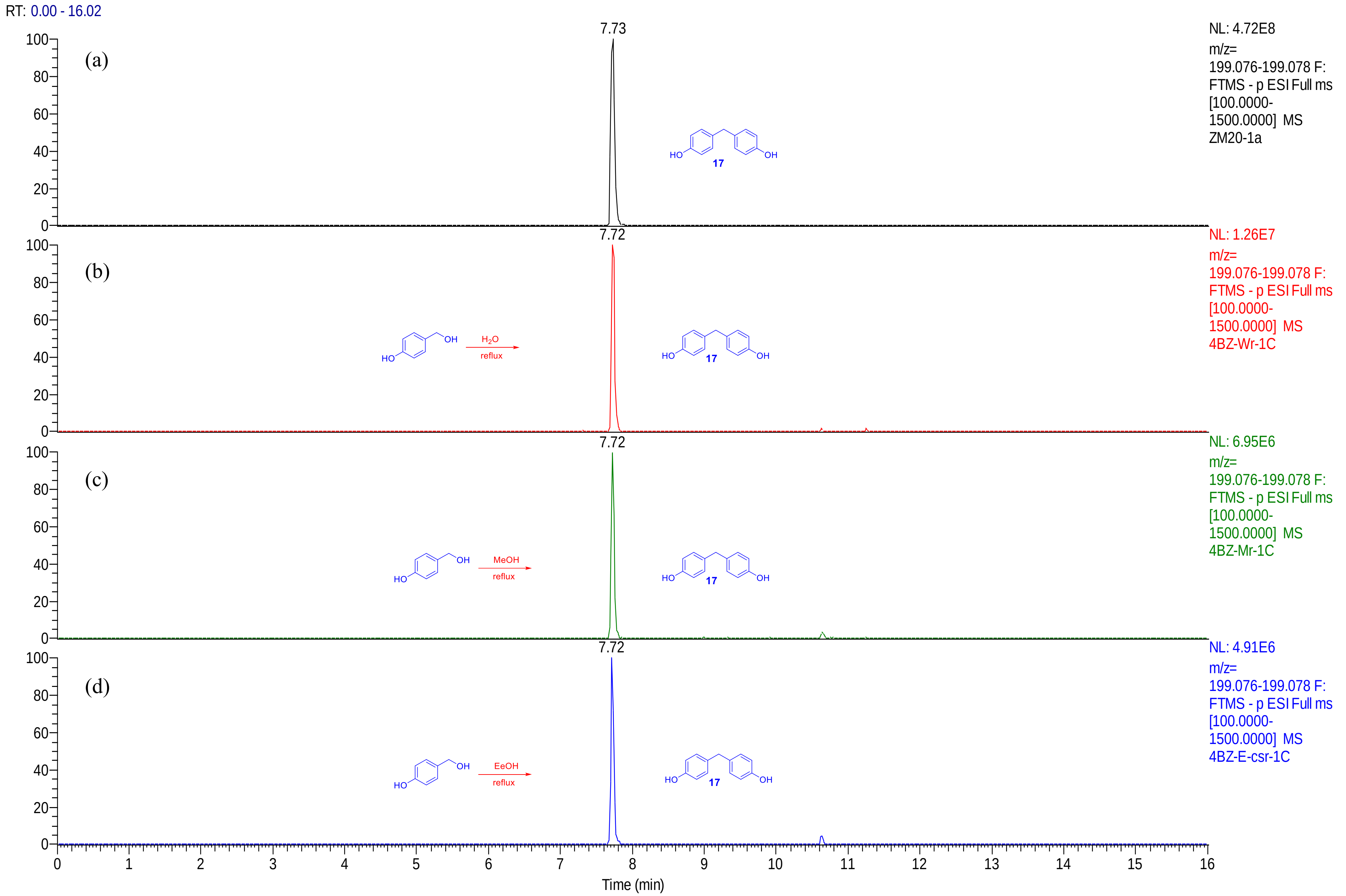

Fig. S182 Overlaid chromatograms of the extracted negative ion at *m/z* 199.077 $[M–H]^-$: (a) compound **17** in CH_3CN; (b)–(d) H_2O, MeOH, and EtOH solutions of *p*-hydroxybenzyl alcohol were sonicated for 0.5 h then refluxed for 1.0 h, respectively.

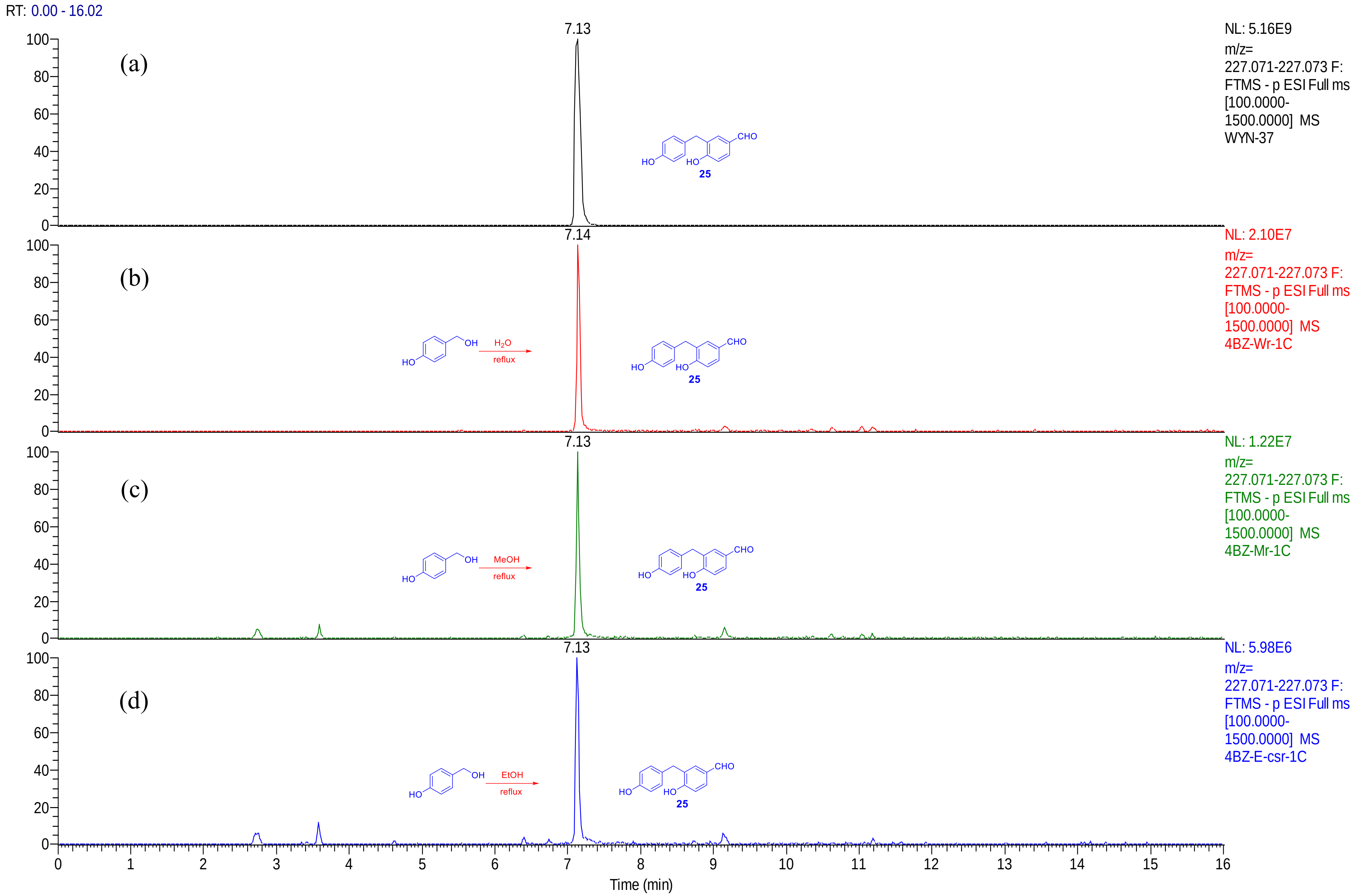

Fig. S183 Overlaid chromatograms of the extracted negative ion at *m/z* 227.072 $[M-H]^-$: (a) compound **25**; (b)–(d) H_2O, MeOH, and EtOH solutions of *p*-hydroxybenzyl alcohol were sonicated for 0.5 h then refluxed for 1.0 h, respectively.

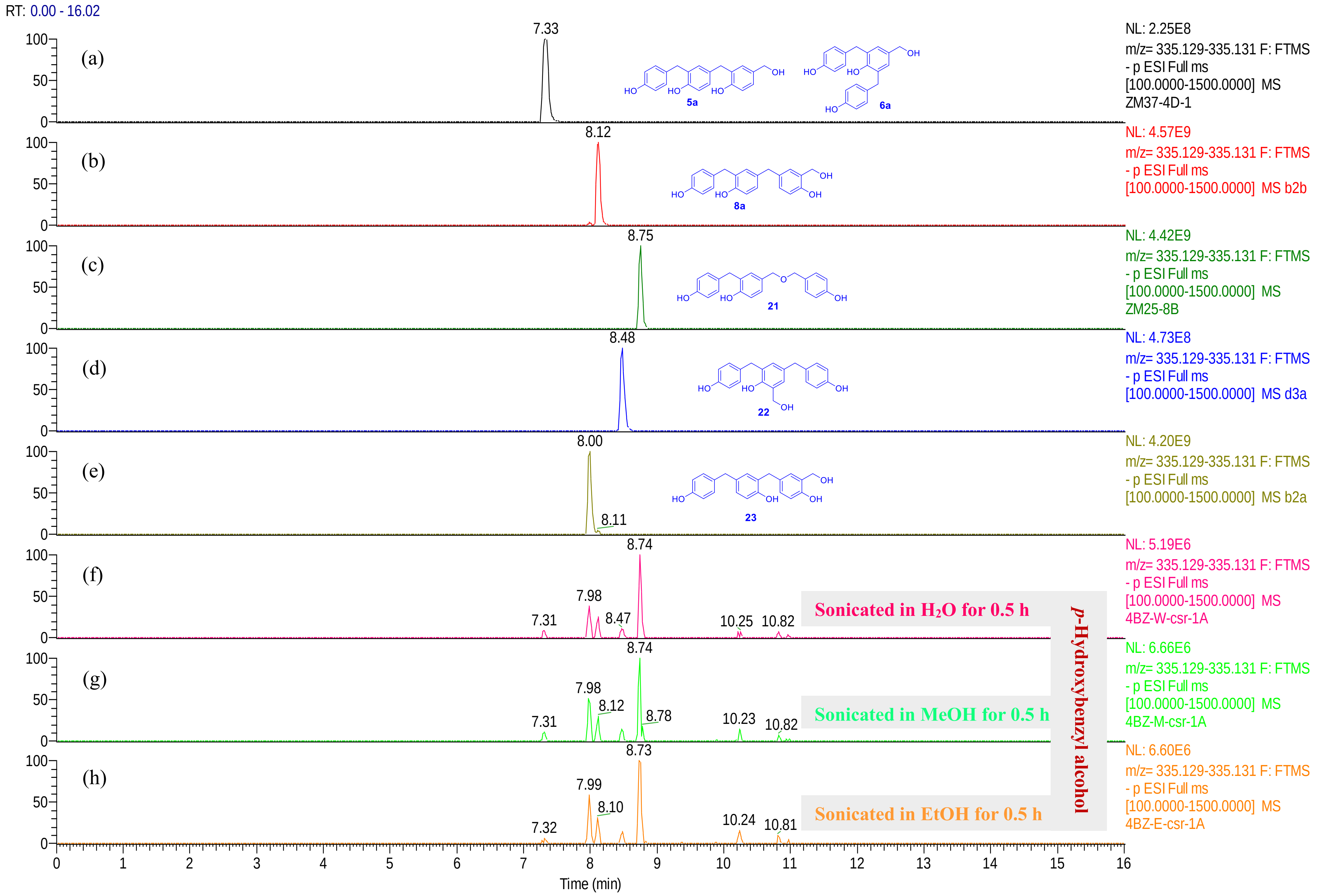

Fig. S184 Overlaid chromatograms of the extracted negative ion at *m/z* 335.130 $[M-H]^-$: (a)–(e) compounds **5a**/**6a**, **8a**, **21**, **22**, and **23** in CH_3CN, respectively; (f)–(h) H_2O, MeOH, and EtOH solutions of *p*-hydroxybenzyl alcohol were sonicated for 0.5 h, respectively.

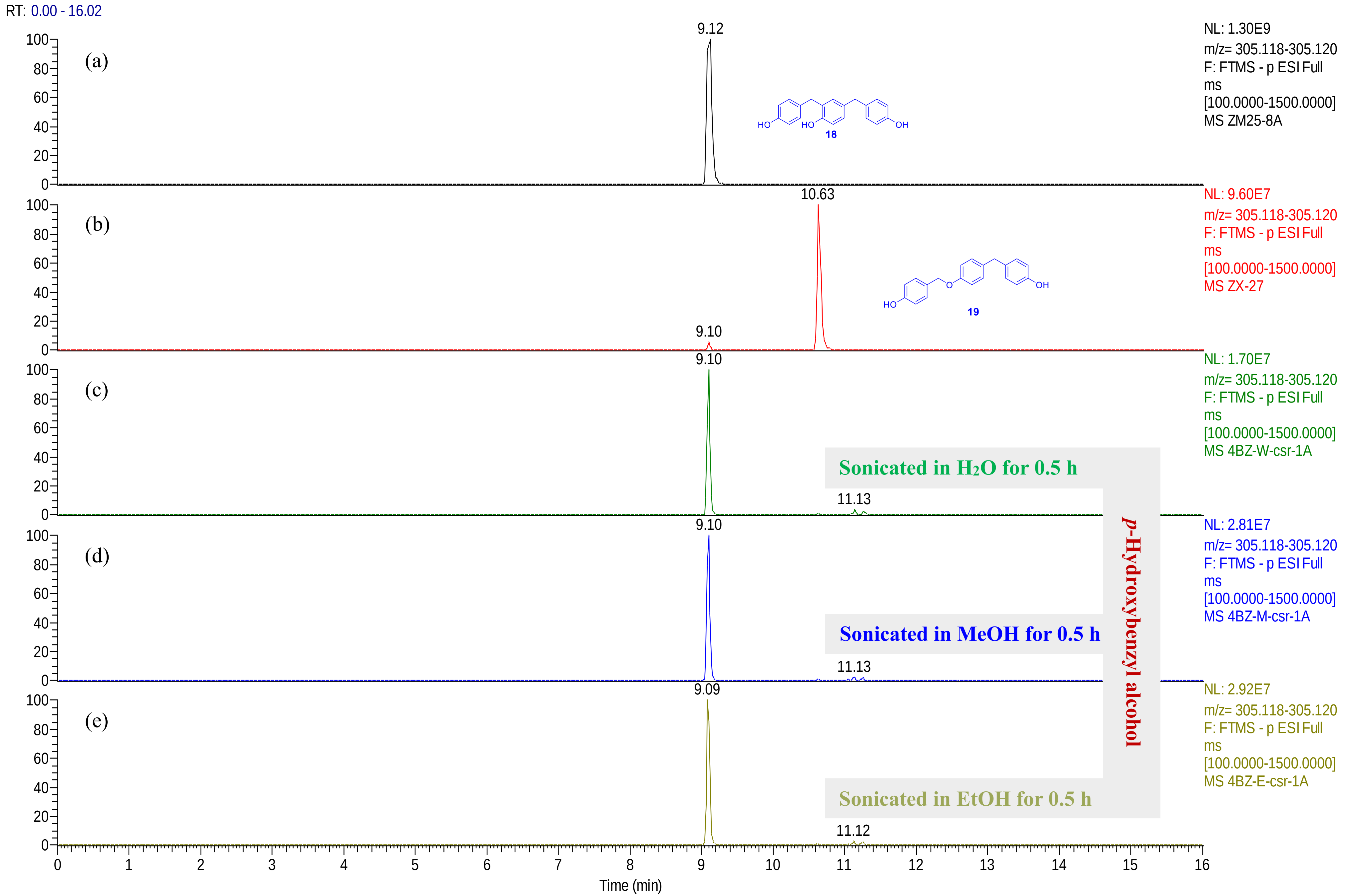

Fig. S185 Overlaid chromatograms of the extracted negative ion at *m/z* 305.119 $[M–H]^-$: (a) and (b) compound **18** and **19** in CH_3CN, respectively; (c)–(e) H_2O, MeOH, and EtOH solutions of *p*-hydroxybenzyl alcohol were sonicated for 0.5 h, respectively.

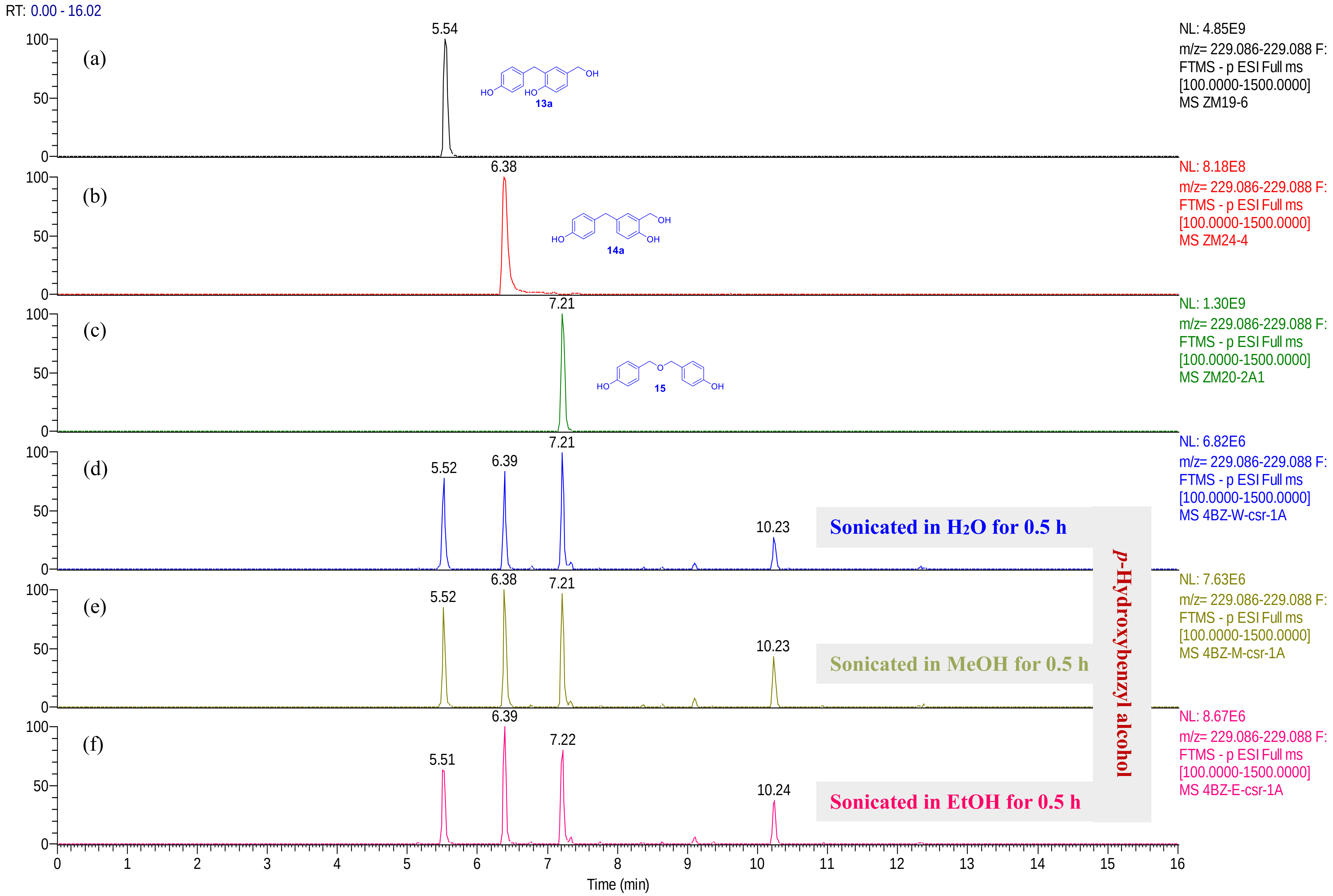

Fig. S186 Overlaid chromatograms of the extracted negative ion at *m/z* 229.087 [M–H]$^-$: (a)–(c) compounds **13a**, **14a**, and **15** in CH_3CN, respectively; (d)–(f) H_2O, MeOH, and EtOH solutions of *p*-hydroxybenzyl alcohol were sonicated for 0.5 h, respectively.

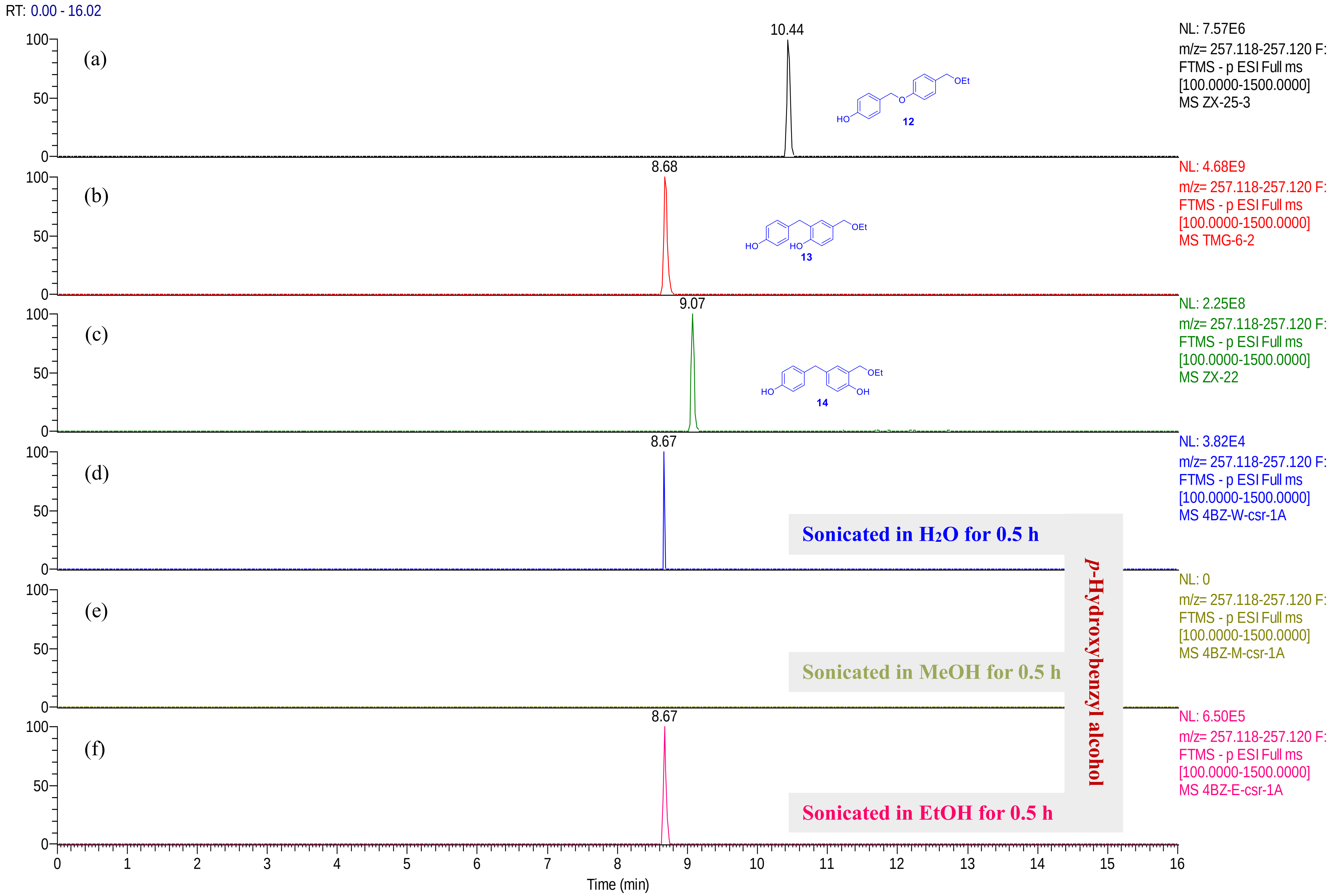

Fig. S187 Overlaid chromatograms of the extracted negative ion at *m/z* 257.119 $[M-H]^-$: (a)–(c) compounds **12**, **13**, and **14** in CH_3CN, respectively; (d)–(f) H_2O, MeOH, and EtOH solutions of *p*-hydroxybenzyl alcohol were sonicated for 0.5 h, respectively.

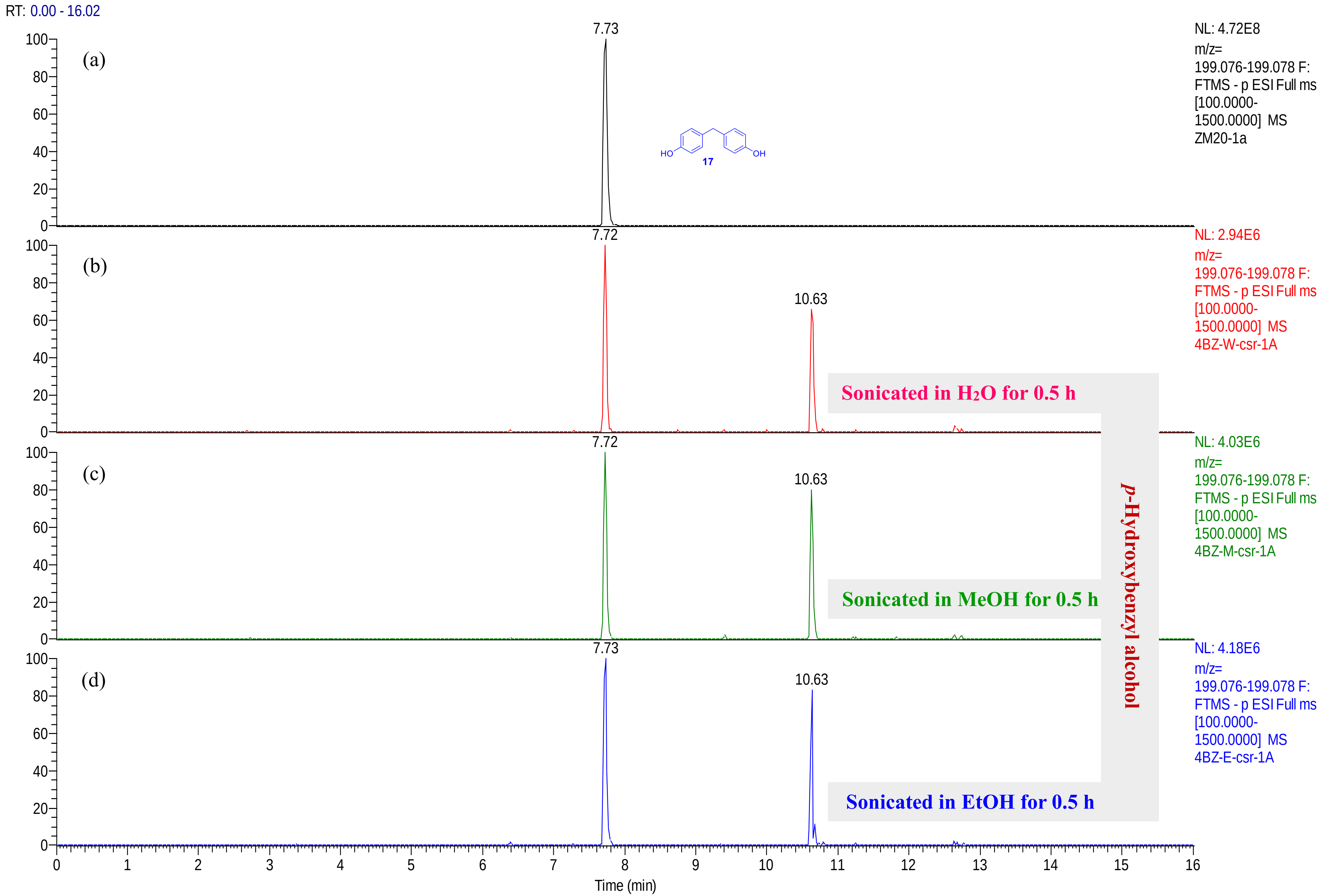

Fig. S188 Overlaid chromatograms of the extracted negative ion at *m/z* 199.077 $[M{-}H]^-$: (a) compound **17** in CH_3CN; (b)–(d) H_2O, MeOH, and EtOH solutions of *p*-hydroxybenzyl alcohol were sonicated for 0.5 h, respectively.

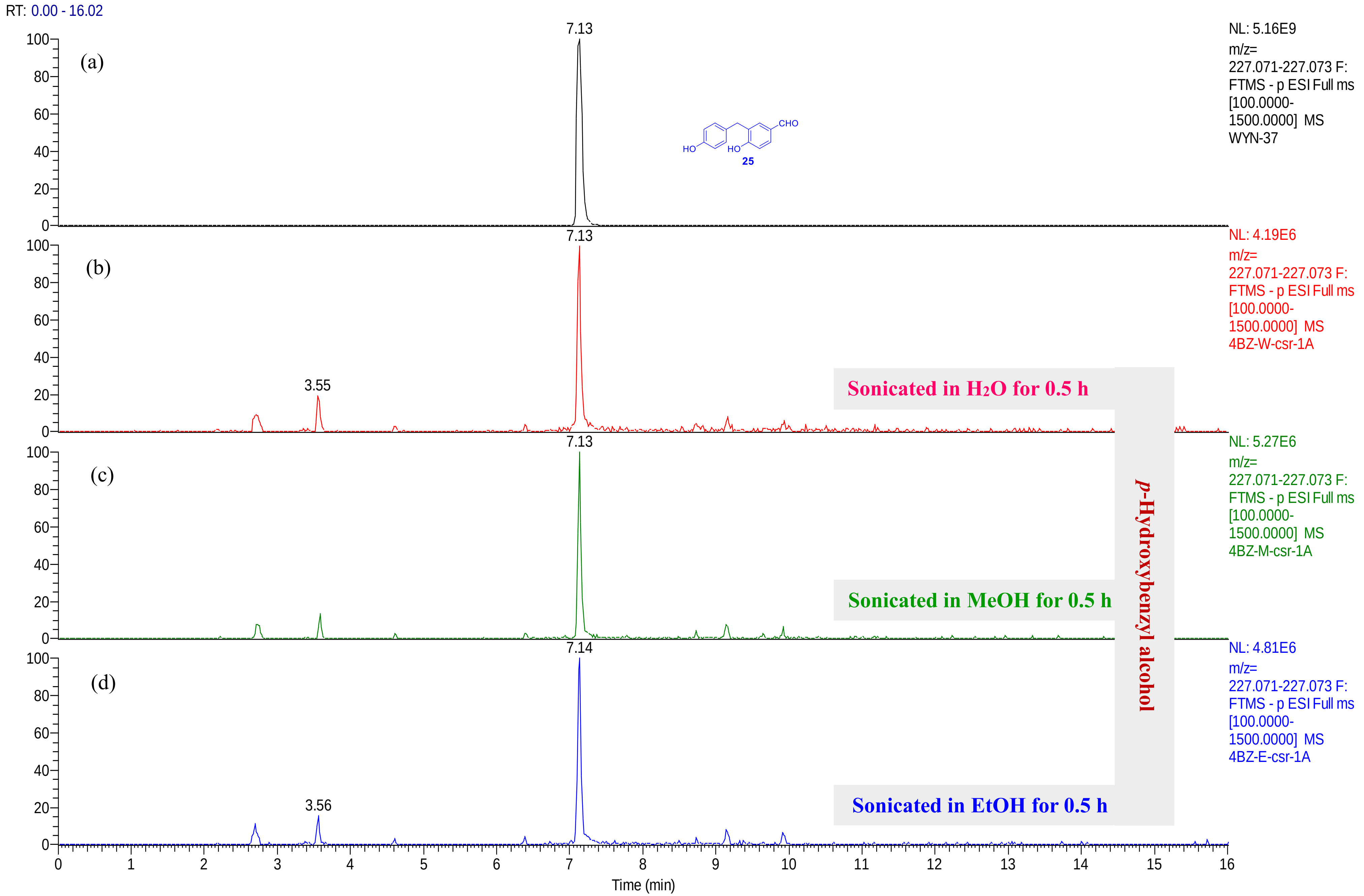

Fig. S189 Overlaid chromatograms of the extracted negative ion at *m/z* 227.072 [M–H]$^-$: (a) compound **25** in CH_3CN; (b)–(d) H_2O, MeOH, and EtOH solutions of *p*-hydroxybenzyl alcohol were sonicated for 0.5 h, respectively.

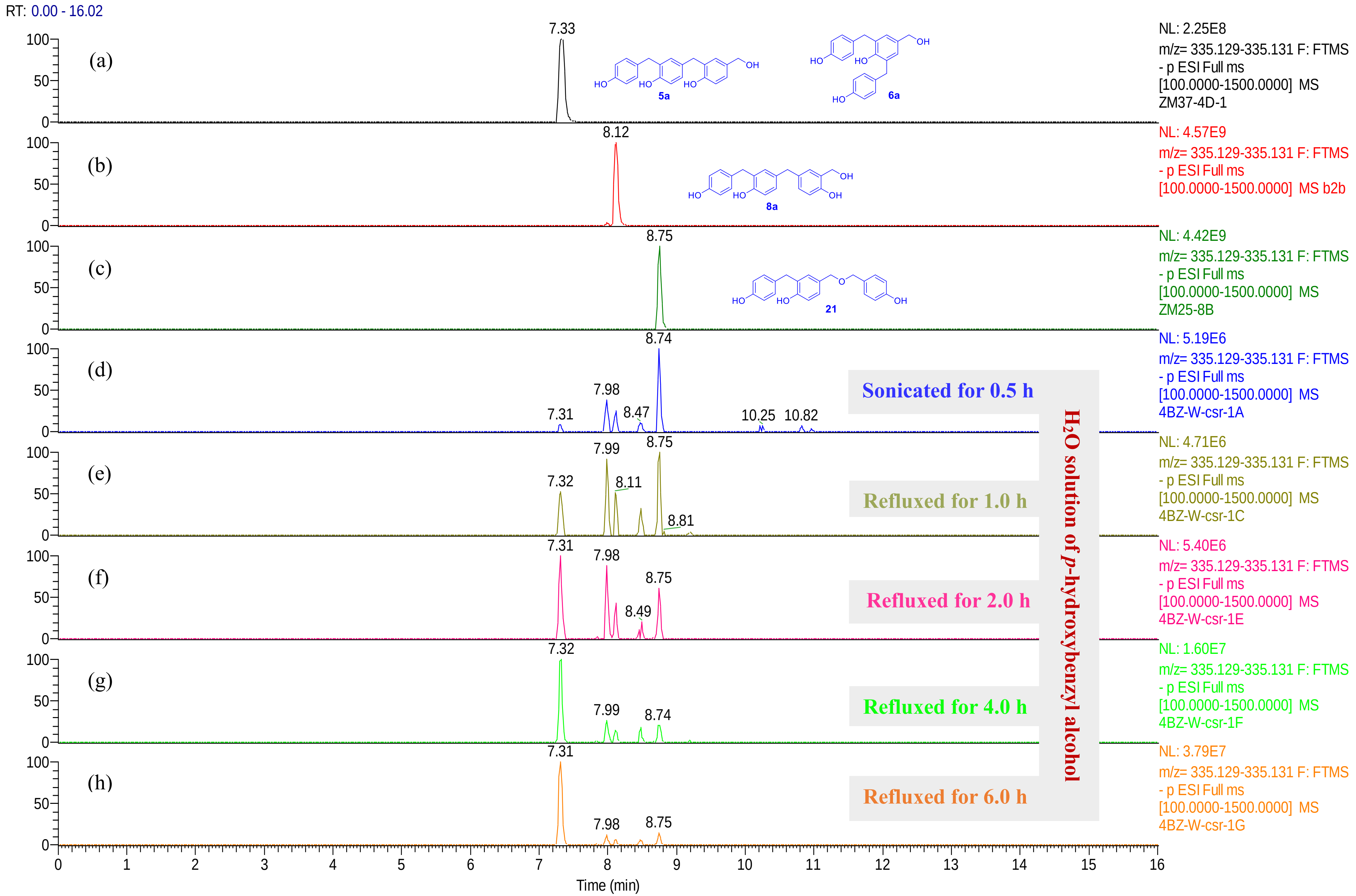

Fig. S190 Overlaid chromatograms of the extracted negative ion at *m/z* 335.130 [M–H]⁻: (a)–(c) compounds **5a**/**6a**, **8a**, and **21** in CH_3CN, respectively; (d)–(h) H_2O solution of *p*-hydroxybenzyl alcohol was sonicated for 0.5 h then refluxed for 1.0 h, 2.0 h, 4.0 h, and 6.0 h, respectively.

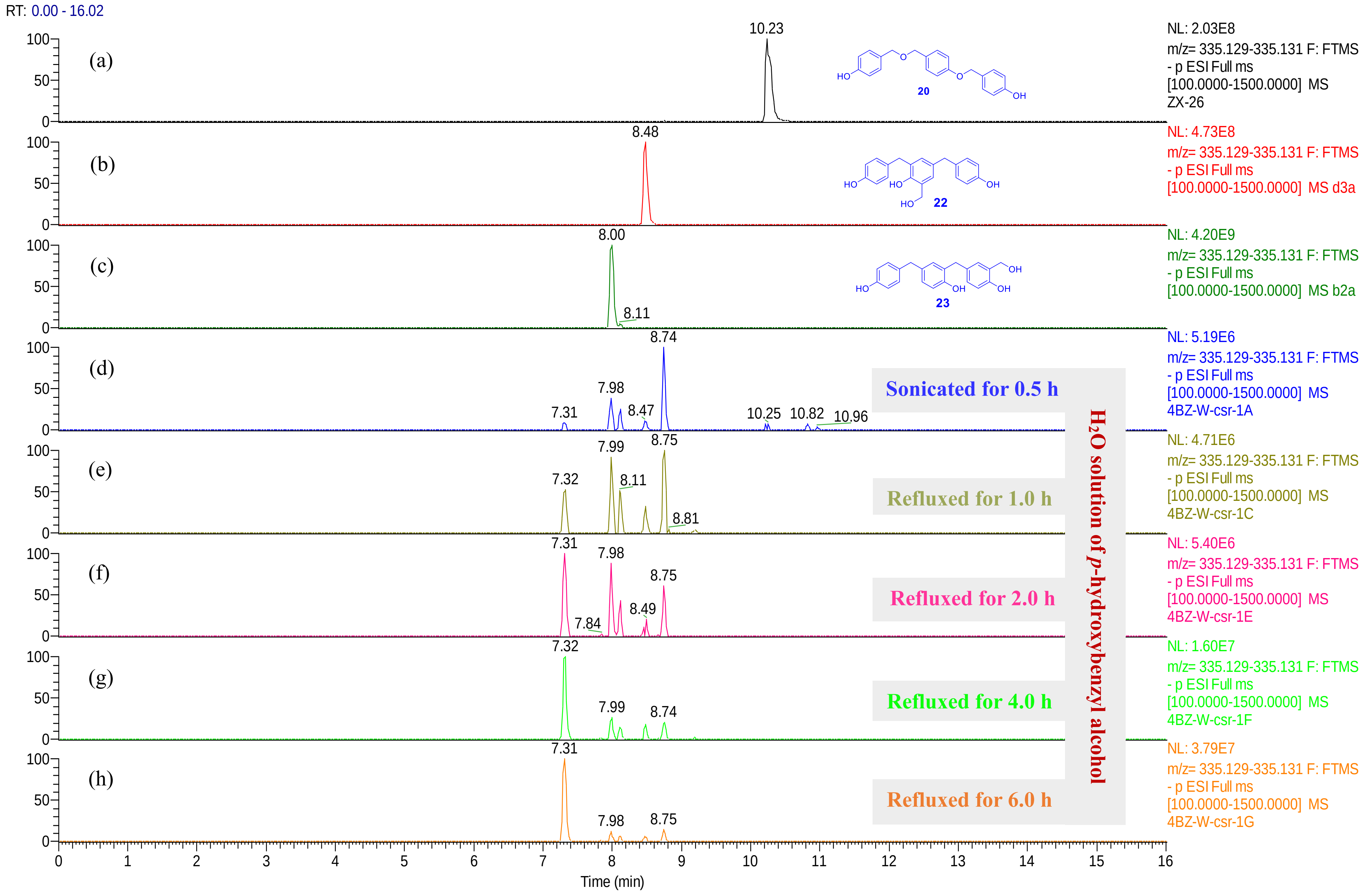

Fig. S191 Overlaid chromatograms of the extracted negative ion at *m/z* 335.130 $[M{-}H]^-$: (a) – (c) compounds **20**, **22**, and **23** in CH_3CN, respectively; (d)–(h) H_2O solution of *p*-hydroxybenzyl alcohol was sonicated for 0.5 h then refluxed for 1.0 h, 2.0 h, 4.0 h, and 6.0 h, respectively.

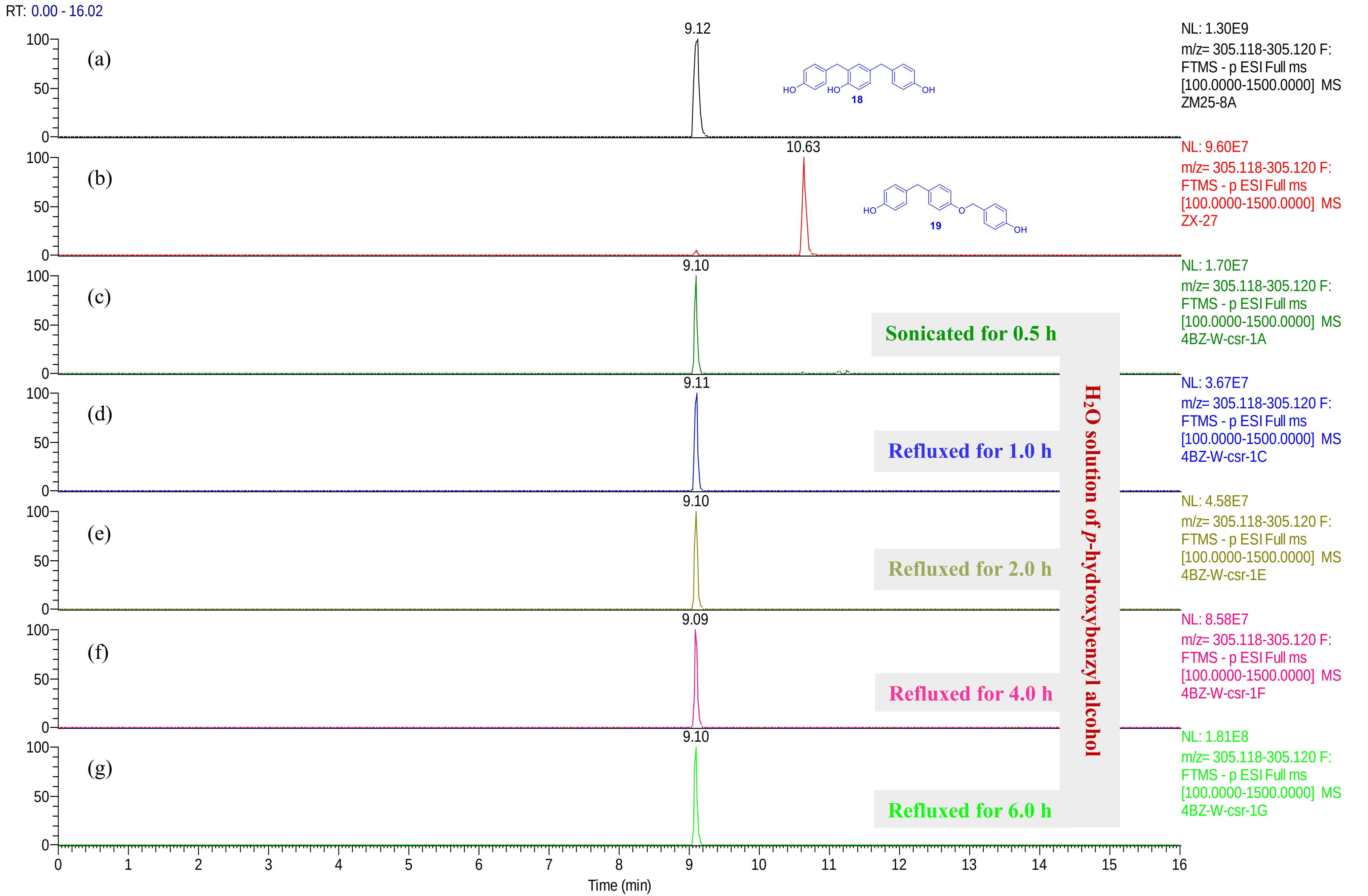

Fig. S192 Overlaid chromatograms of the extracted negative ion at *m/z* 305.119 $[M-H]^-$: (a) and (b) compounds **18** and **19** in CH_3CN, respectively; (c)–(g) H_2O solution of *p*-hydroxybenzyl alcohol was sonicated for 0.5 h then refluxed for 1.0 h, 2.0 h, 4.0 h, and 6.0 h, respectively.

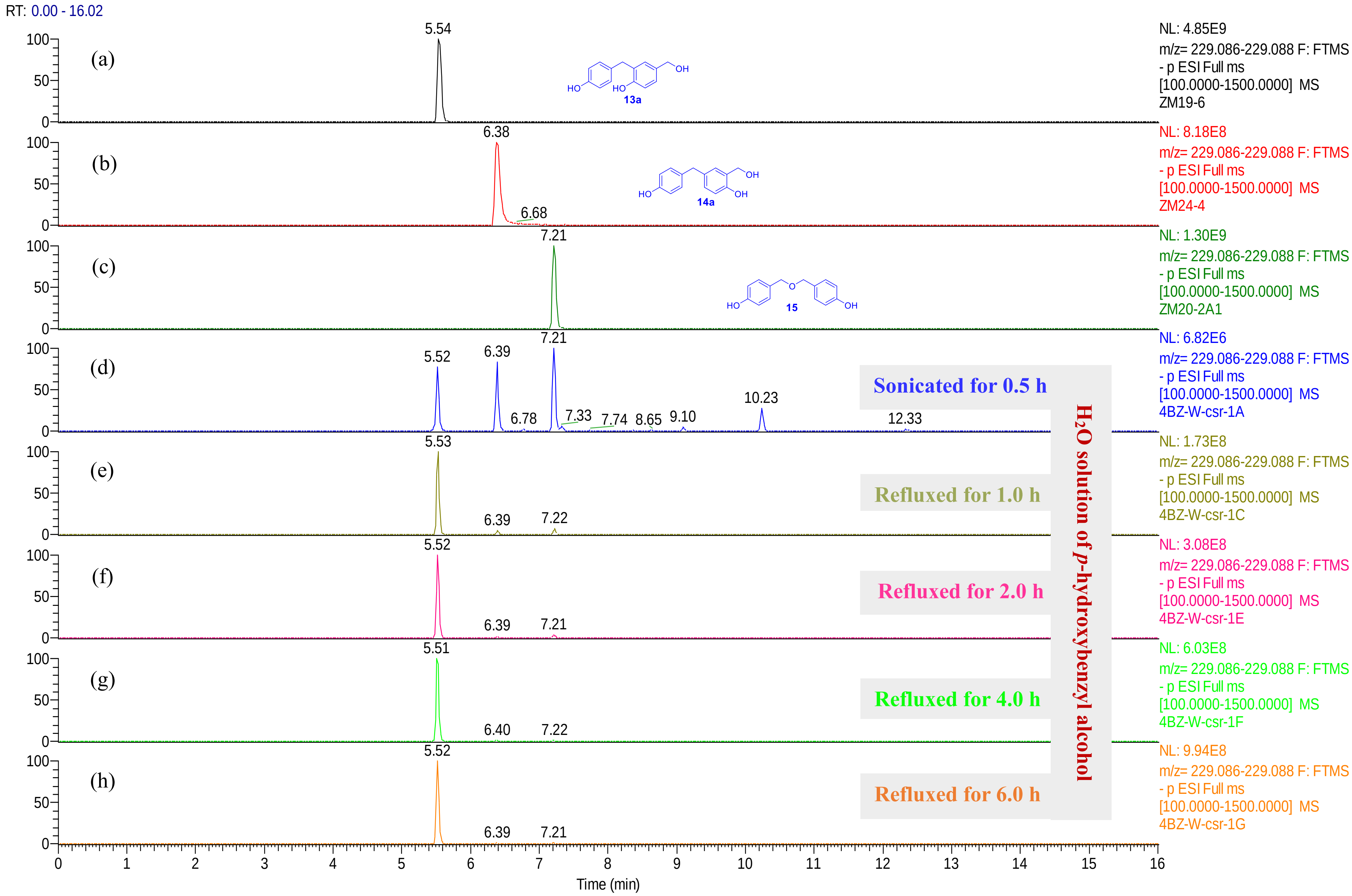

Fig. S193 Overlaid chromatograms of the extracted negative ion at *m/z* 229.087 [M–H]⁻: (a)–(c) compounds **13a**, **14a**, and **15** in CH_3CN, respectively; (d)–(h) H_2O solution of *p*-hydroxybenzyl alcohol was sonicated for 0.5 h then refluxed for 1.0 h, 2.0 h, 4.0 h, and 6.0 h, respectively.

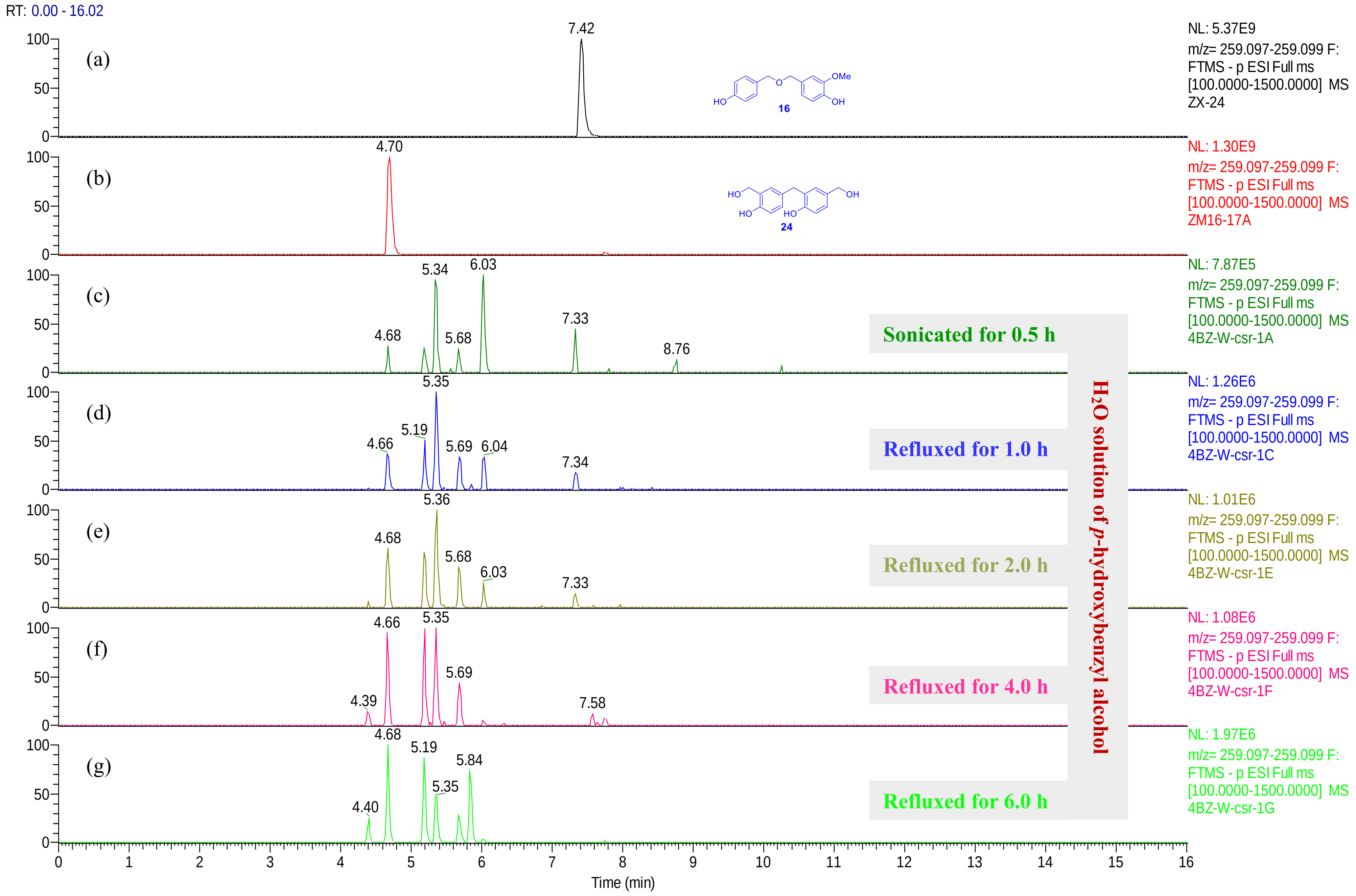

Fig. S194 Overlaid chromatograms of the extracted negative ion at *m/z* 259.098 $[M{-}H]^-$: (a) and (b) compounds **16** and **24** in CH_3CN, respectively; (c)–(g) H_2O solution of *p*-hydroxybenzyl alcohol was sonicated for 0.5 h then refluxed for 1.0 h, 2.0 h, 4.0 h, and 6.0 h, respectively.

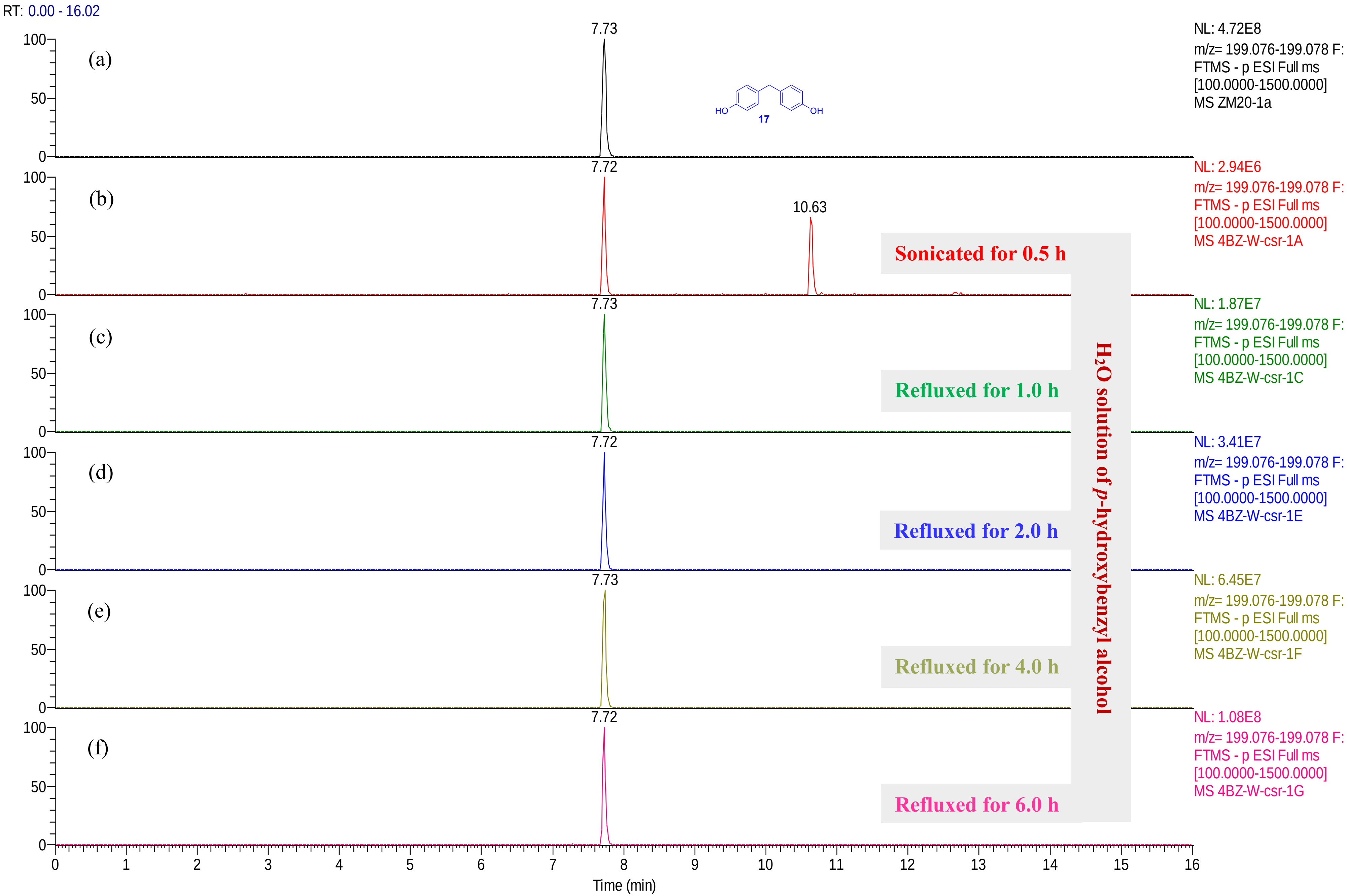

Fig. S195 Overlaid chromatograms of the extracted negative ion at *m/z* 199.077 [M–H]$^-$: (a) compound **17** in CH_3CN; (b)–(f) H_2O solution of *p*-hydroxybenzyl alcohol was sonicated for 0.5 h then refluxed for 1.0 h, 2.0 h, 4.0 h, and 6.0 h, respectively.

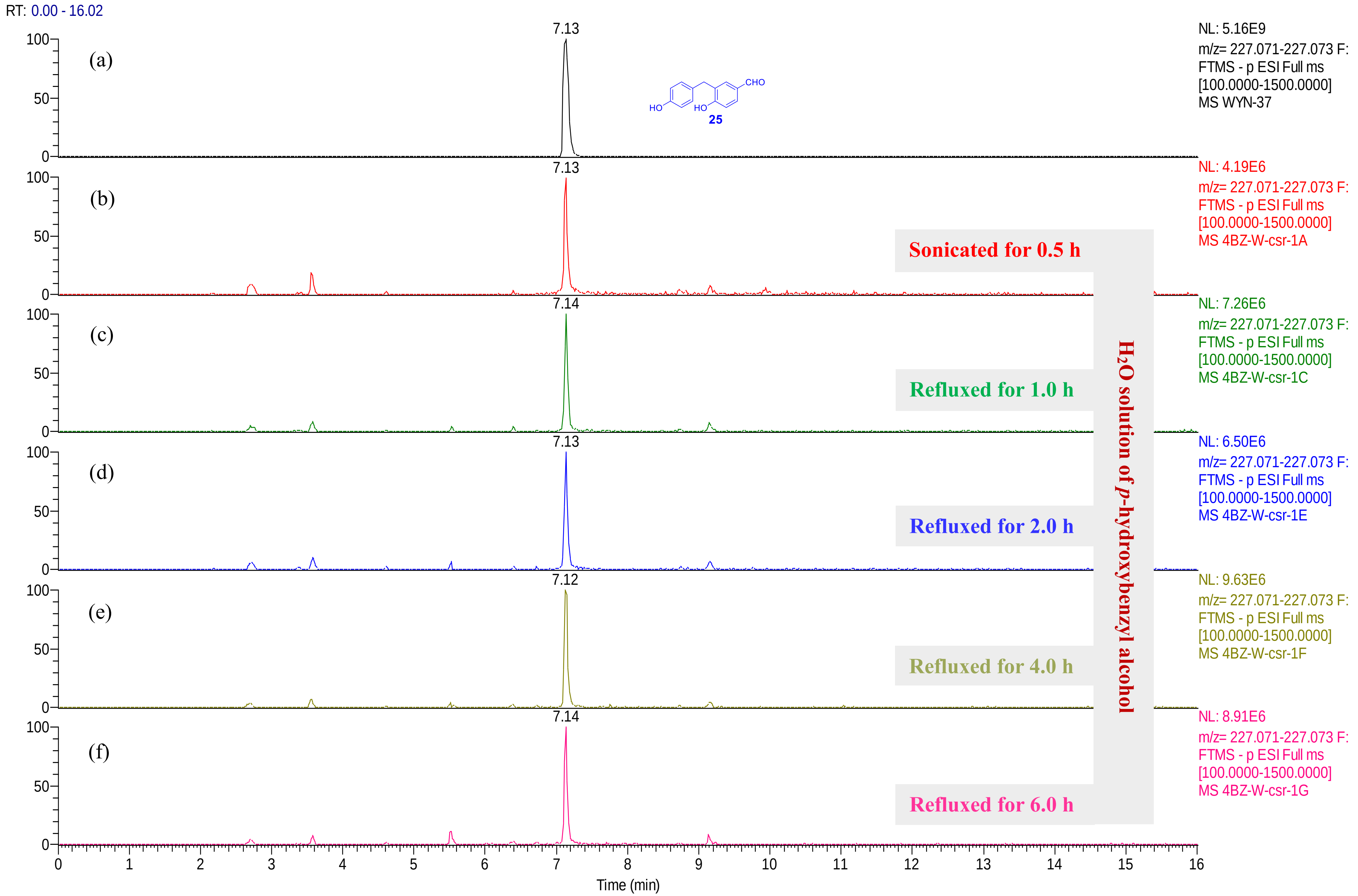

Fig. S196 Overlaid chromatograms of the extracted negative ion at *m/z* 227.072 $[M-H]^-$: (a) compound **25** in CH_3CN; (b)–(f) H_2O solution of *p*-hydroxybenzyl alcohol was sonicated for 0.5 h then refluxed for 1.0 h, 2.0 h, 4.0 h, and 6.0 h, respectively.

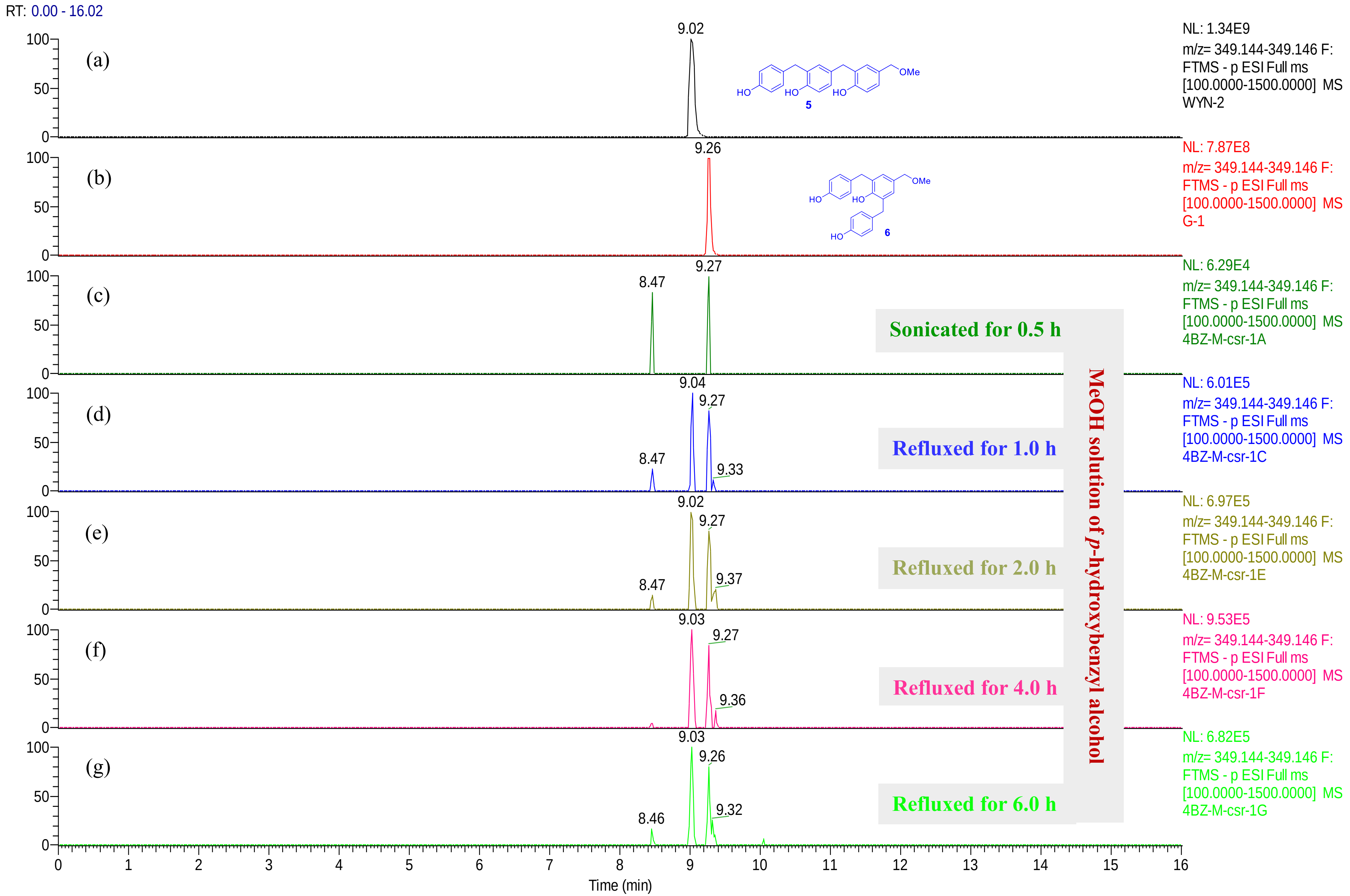

Fig. S197 Overlaid chromatograms of the extracted negative ion at *m/z* 349.145 $[M{-}H]^-$: (a)–(c) compounds **5** and **6** in CH_3CN, respectively; (d)–(g) MeOH solution of *p*-hydroxybenzyl alcohol was sonicated for 0.5 h then refluxed for 1.0 h, 2.0 h, 4.0 h, and 6.0 h, respectively.

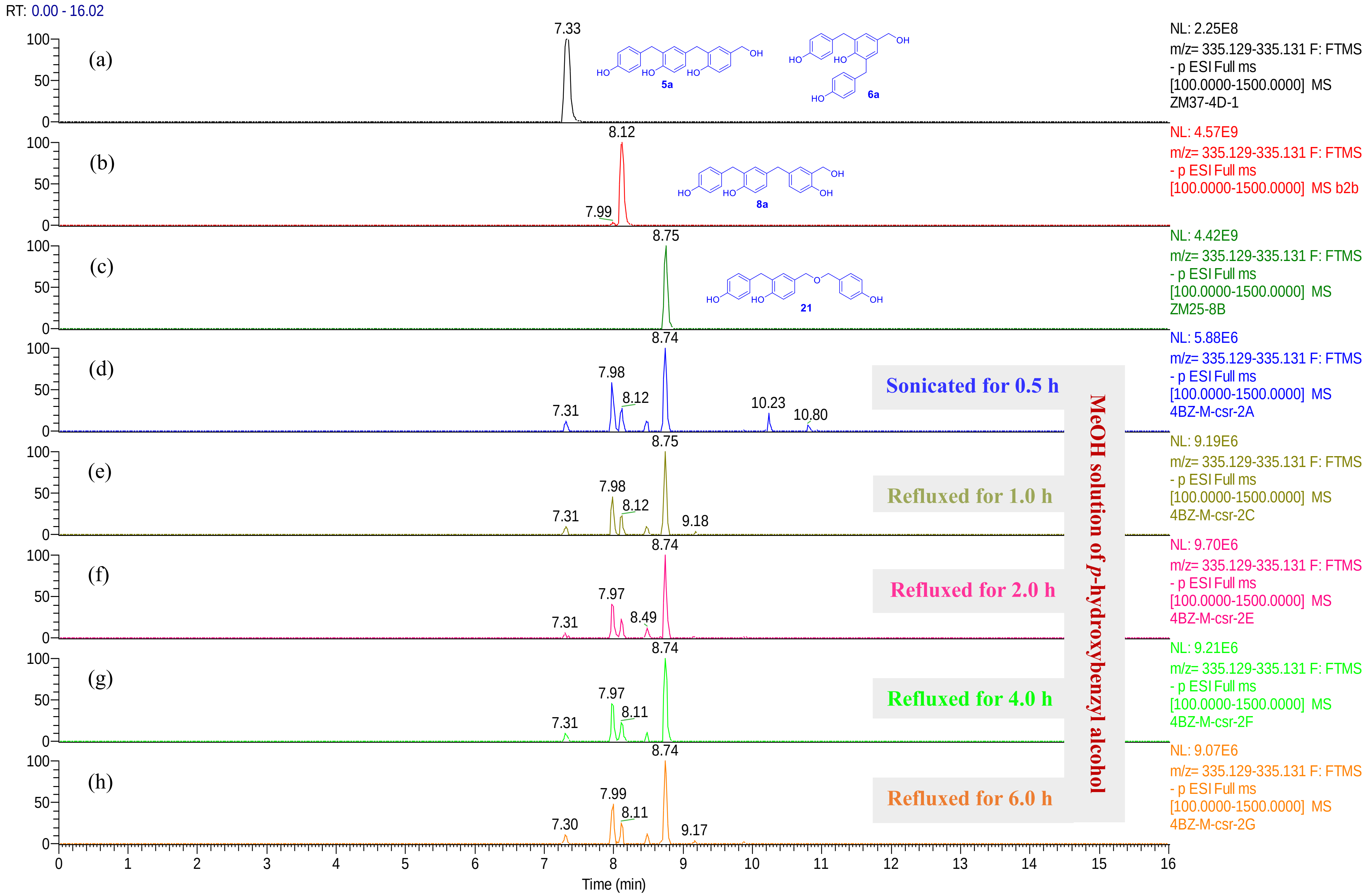

Fig. S198 Overlaid chromatograms of the extracted negative ion at *m/z* 335.130 $[M–H]^-$: (a)–(c) compounds **5a**/**6a**, **8a**, and **21** in CH_3CN, respectively; (d)–(h) MeOH solution of *p*-hydroxybenzyl alcohol was sonicated for 0.5 h then refluxed for 1.0 h, 2.0 h, 4.0 h, and 6.0 h, respectively.

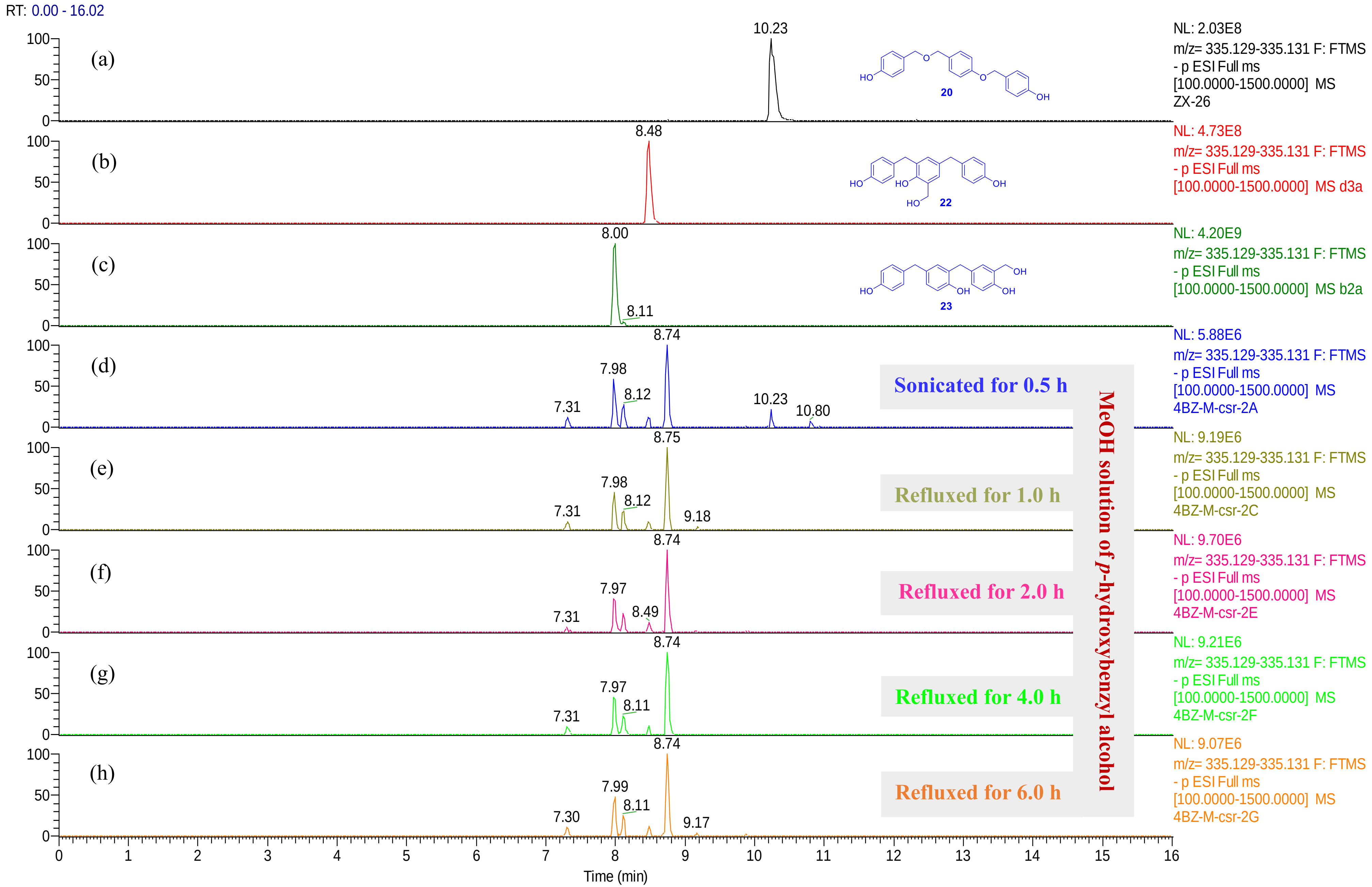

Fig. S199 Overlaid chromatograms of the extracted negative ion at *m/z* 335.130 $[M-H]^-$: (a)–(c) compounds **20**, **22**, and **23** in CH_3CN, respectively; (d)–(h) MeOH solution of *p*-hydroxybenzyl alcohol was sonicated for 0.5 h then refluxed for 1.0 h, 2.0 h, 4.0 h, and 6.0 h, respectively.

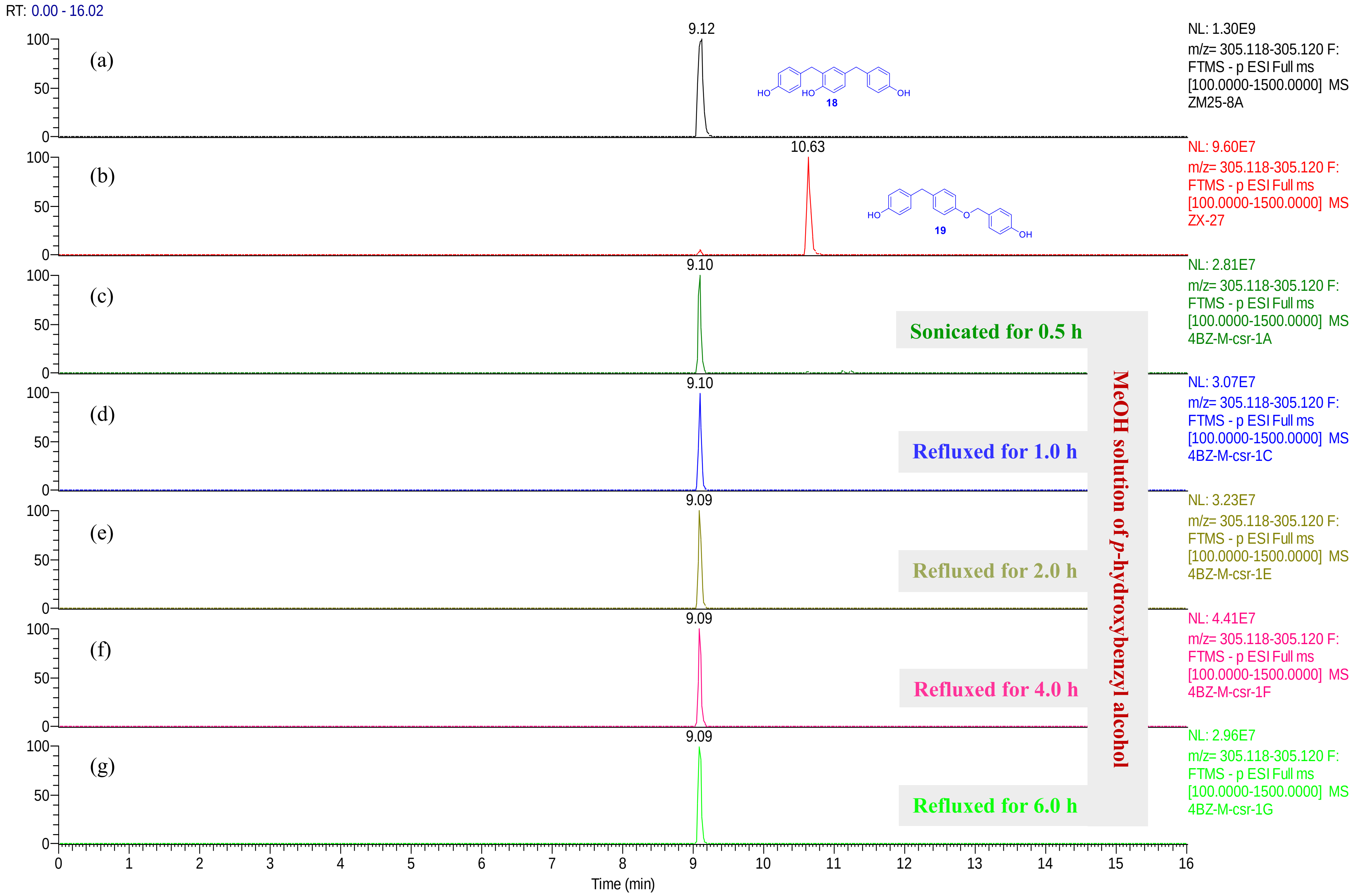

Fig. S200 Overlaid chromatograms of the extracted negative ion at *m/z* 305.119 $[M-H]^-$: (a) and (b) compounds **18** and **19** in CH_3CN, respectively; (c)–(g) MeOH solution of *p*-hydroxybenzyl alcohol was sonicated for 0.5 h then refluxed for 1.0 h, 2.0 h, 4.0 h, and 6.0 h, respectively.

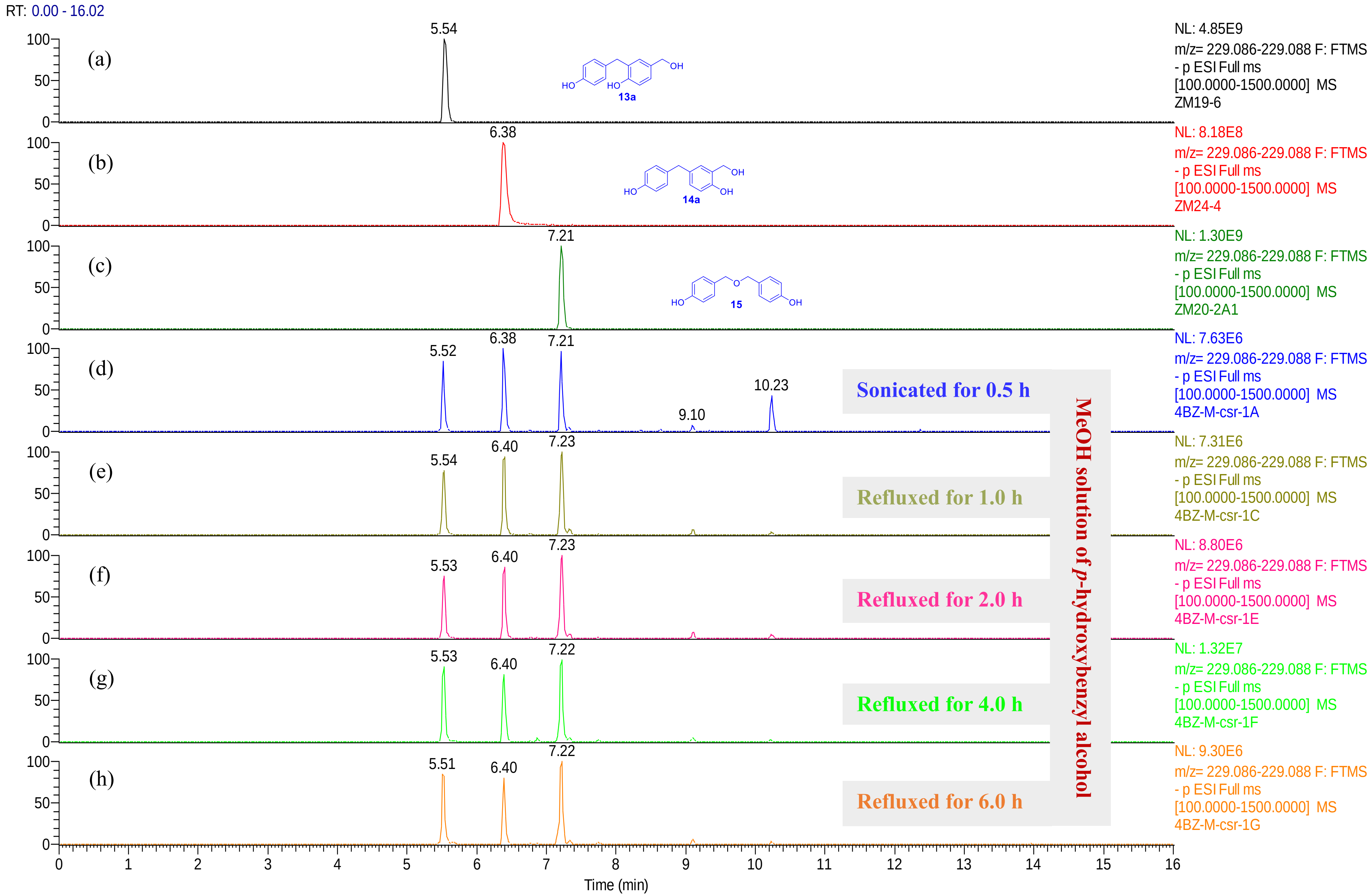

Fig. S201 Overlaid chromatograms of the extracted negative ion at *m/z* 229.087 $[M{-}H]^-$: (a)–(c) compounds **13a**, **14a**, and **15** in CH_3CN, respectively; (d)–(h) MeOH solution of *p*-hydroxybenzyl alcohol was sonicated for 0.5 h then refluxed for 1.0 h, 2.0 h, 4.0 h, and 6.0 h, respectively.

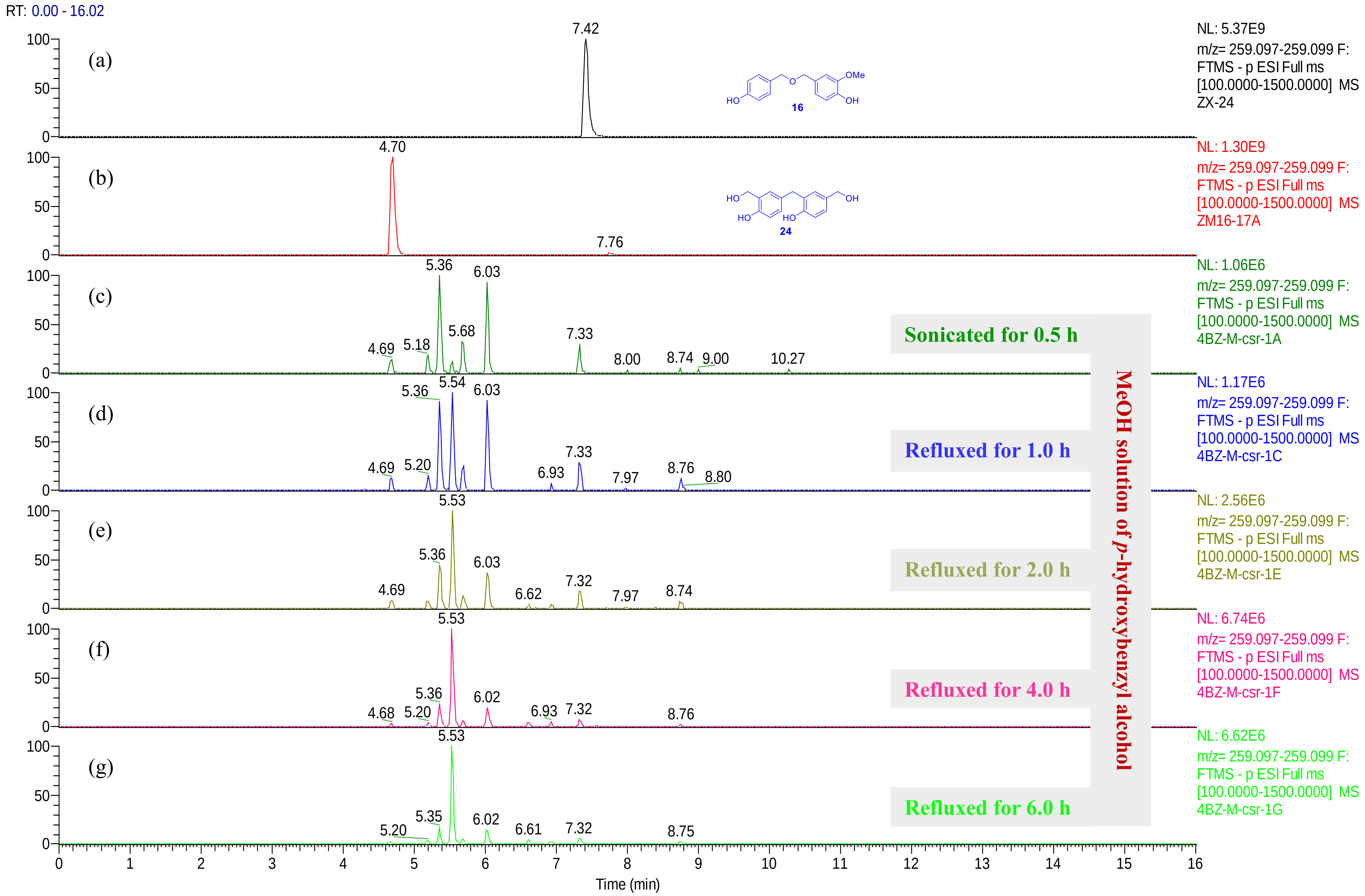

Fig. S202 Overlaid chromatograms of the extracted negative ion at *m/z* 259.098 [M–H]$^-$: (a) and (b) compounds **16** and **24** in CH_3CN, respectively; (c)–(g) MeOH solution of *p*-hydroxybenzyl alcohol was sonicated for 0.5 h then refluxed for 1.0 h, 2.0 h, 4.0 h, and 6.0 h, respectively.

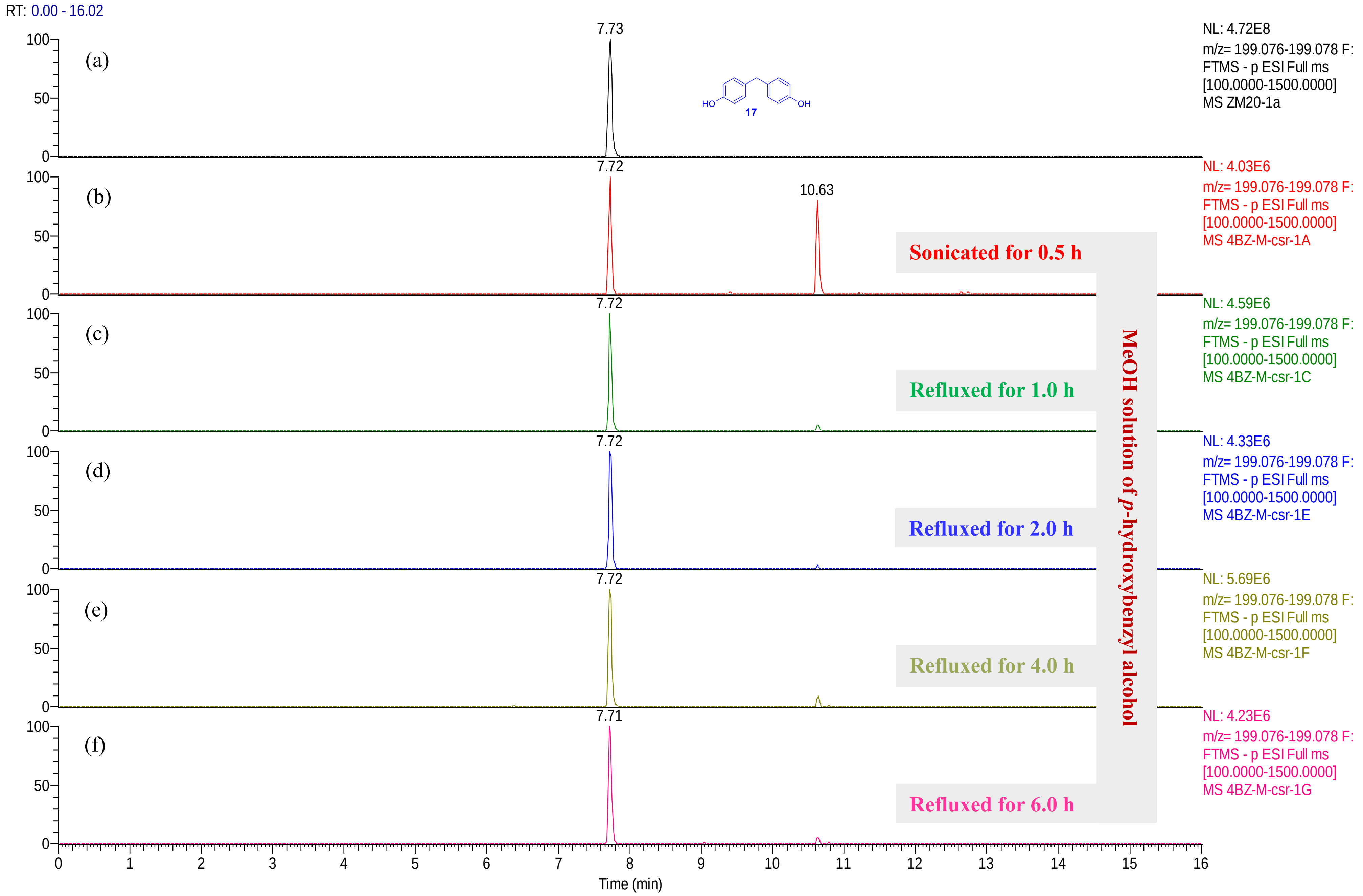

Fig. S203 Overlaid chromatograms of the extracted negative ion at *m/z* 199.077 [M–H]⁻: (a) compound **17** in CH_3CN; (b)–(f) MeOH solution of *p*-hydroxybenzyl alcohol was sonicated for 0.5 h then refluxed for 1.0 h, 2.0 h, 4.0 h, and 6.0 h, respectively.

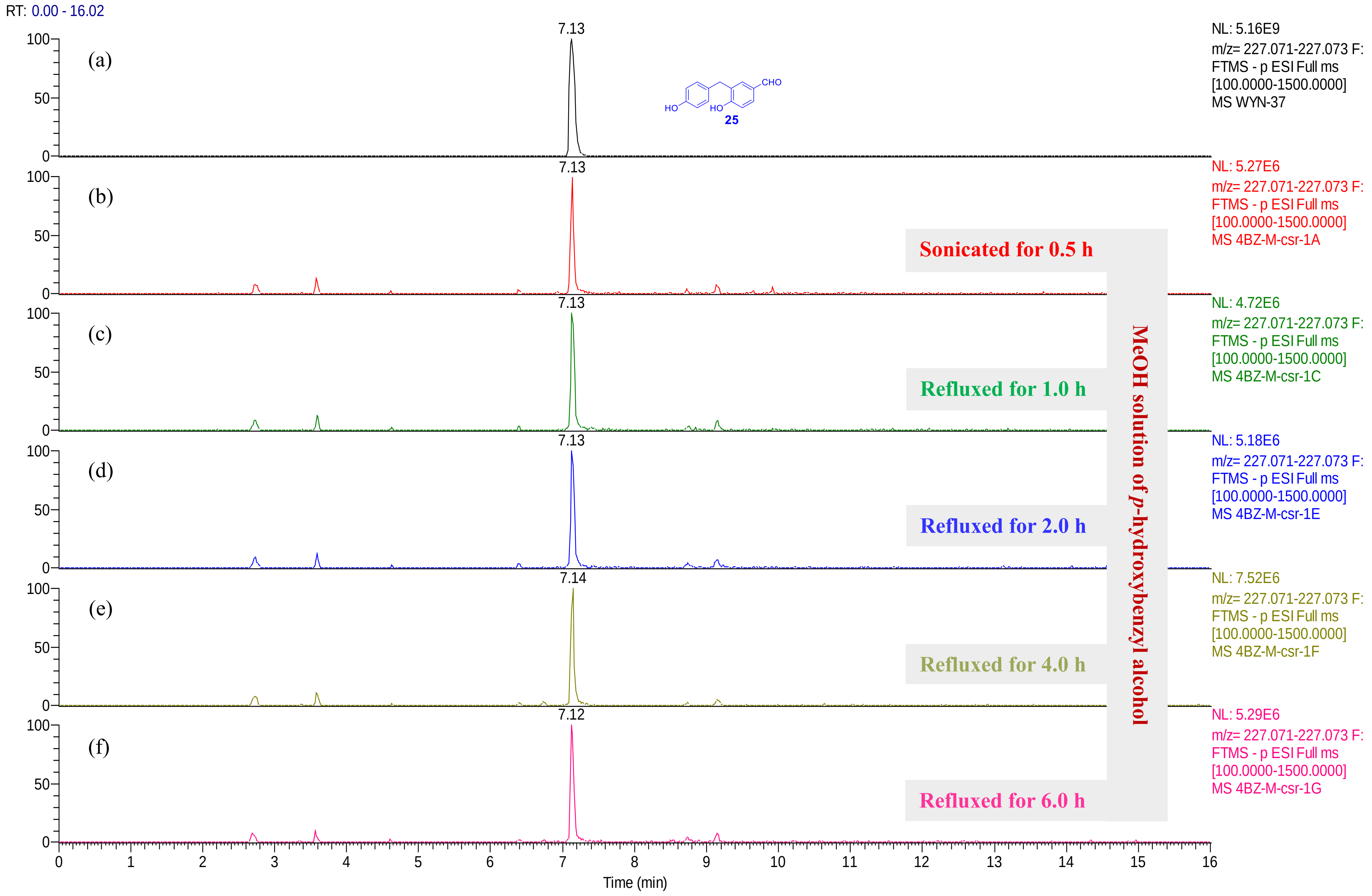

Fig. S204 Overlaid chromatograms of the extracted negative ion at *m/z* 227.072 [M–H]⁻: (a) compound **25** in CH_3CN; (b)–(f) MeOH solution of *p*-hydroxybenzyl alcohol was sonicated for 0.5 h then refluxed for 1.0 h, 2.0 h, 4.0 h, and 6.0 h, respectively.

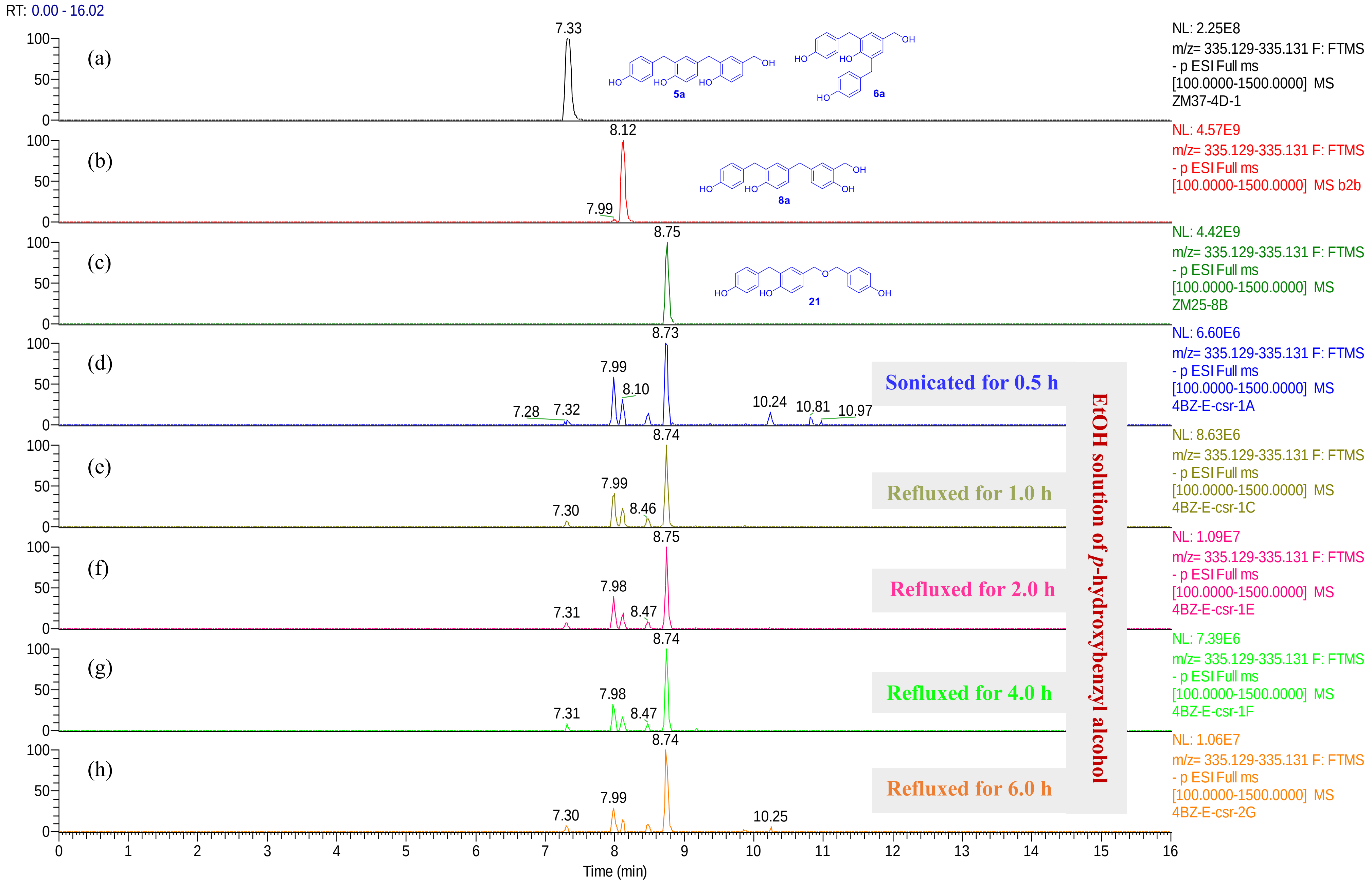

Fig. S205 Overlaid chromatograms of the extracted negative ion at m/z 335.130 $[M-H]^-$: (a)–(c) compounds **5a**/**6a**, **8a**, and **21** in CH_3CN, respectively; (d)–(h) EtOH solution of *p*-hydroxybenzyl alcohol was sonicated for 0.5 h then refluxed for 1.0 h, 2.0 h, 4.0 h, and 6.0 h, respectively.

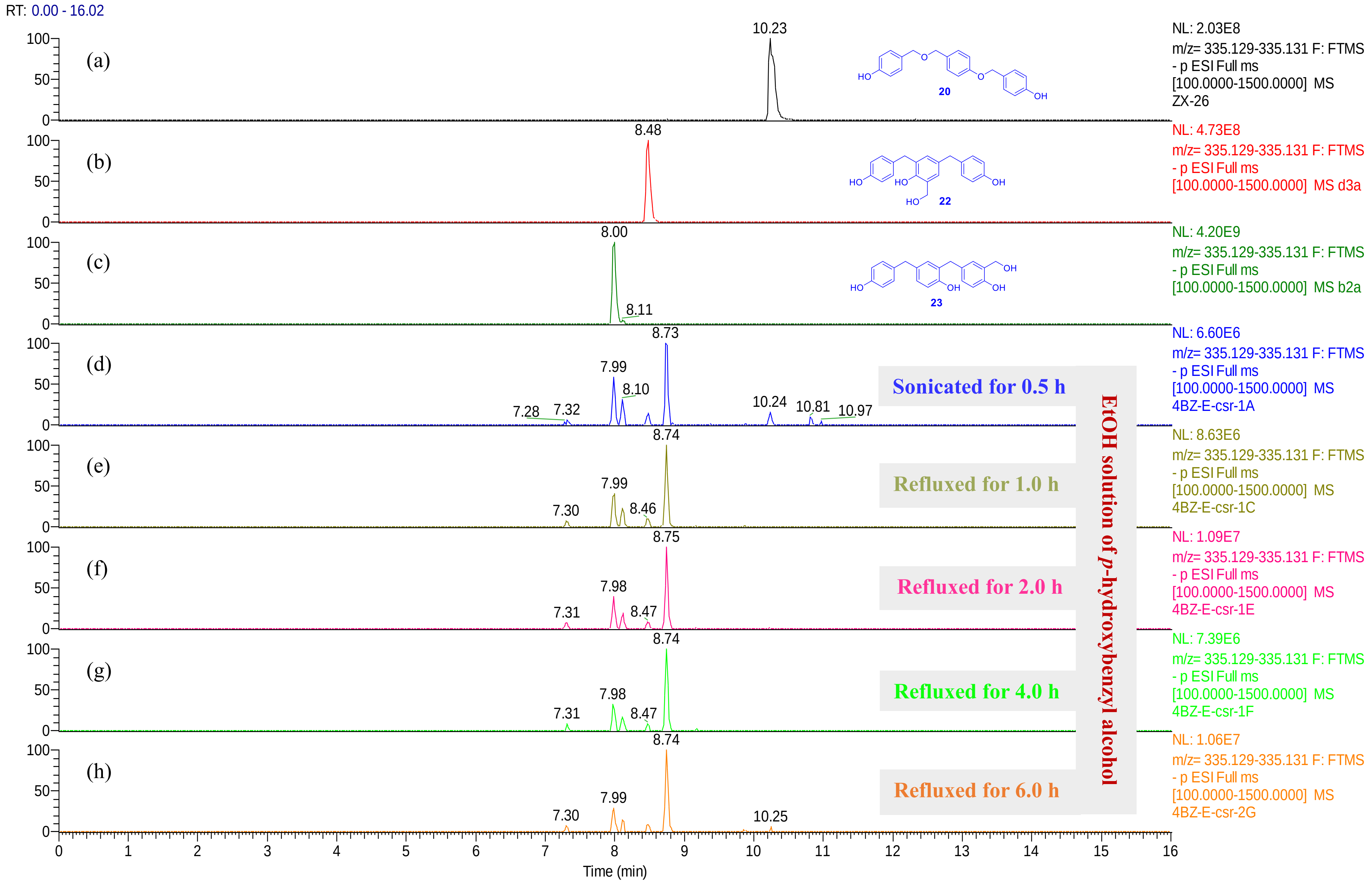

Fig. S206 Overlaid chromatograms of the extracted negative ion at *m/z* 335.130 [M−H]⁻: (a)–(c) compounds **20**, **22**, and **23** in CH_3CN, respectively; (d)–(h) EtOH solution of *p*-hydroxybenzyl alcohol was sonicated for 0.5 h then refluxed for 1.0 h, 2.0 h, 4.0 h, and 6.0 h, respectively.

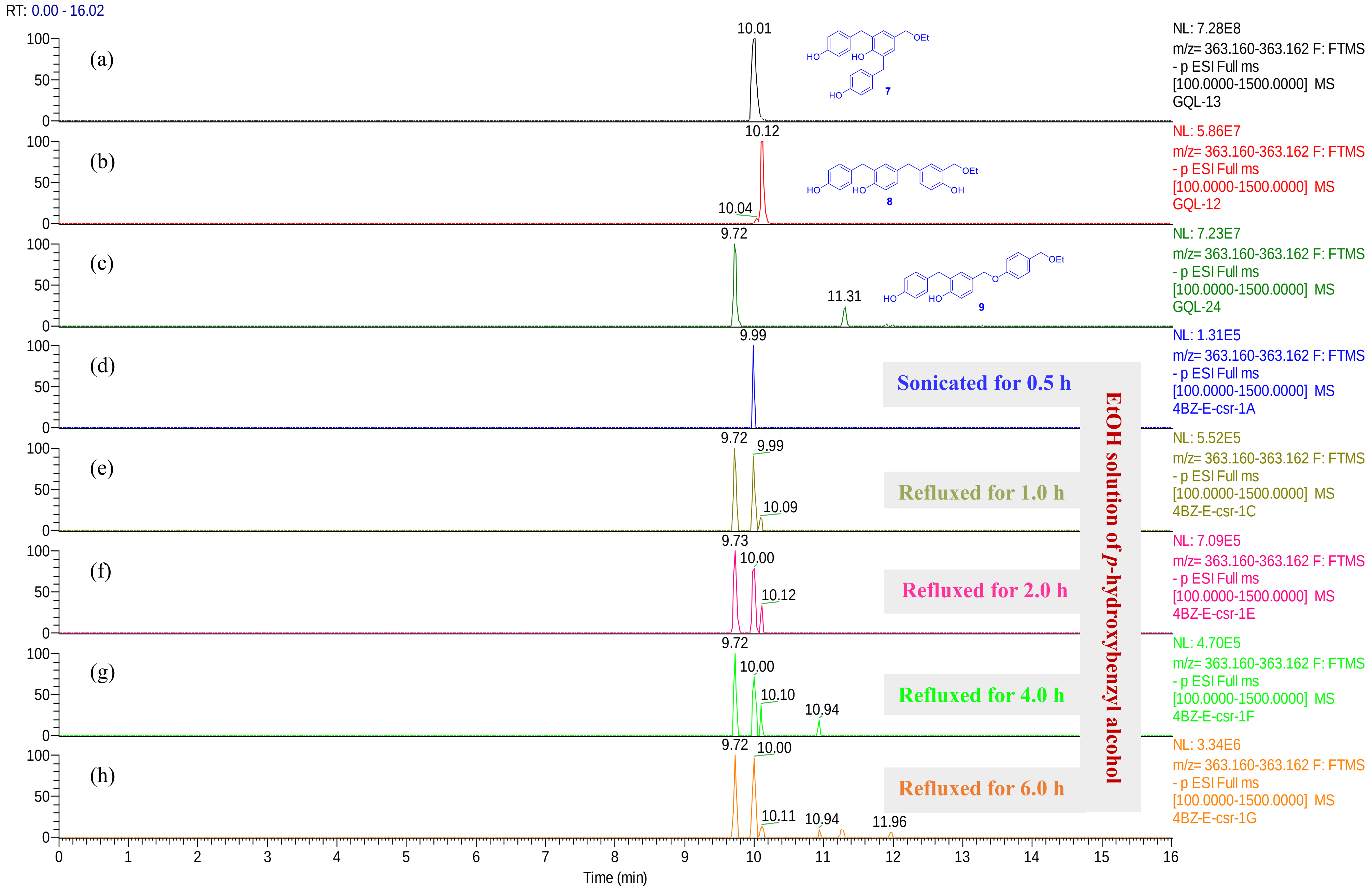

Fig. S207 Overlaid chromatograms of the extracted negative ion at *m/z* 363.161 [M–H]⁻: (a)–(c) compounds **7**, **8**, and **9** in CH_3CN, respectively; (d)–(h) EtOH solution of *p*-hydroxybenzyl alcohol was sonicated for 0.5 h then refluxed for 1.0 h, 2.0 h, 4.0 h, and 6.0 h, respectively.

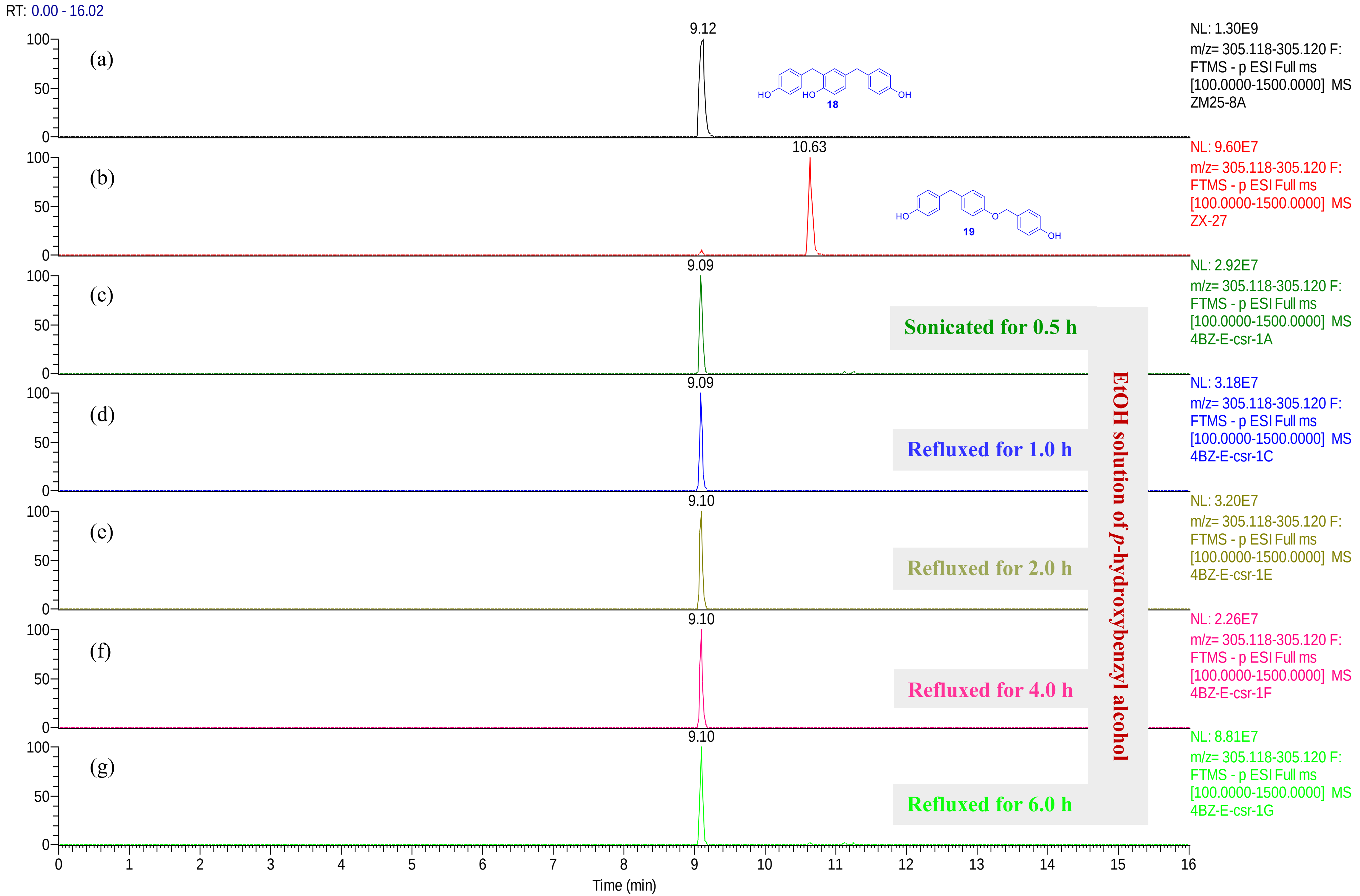

Fig. S208 Overlaid chromatograms of the extracted negative ion at *m/z* 305.119 $[M-H]^-$: (a) and (b) compounds **18** and **19** in CH_3CN, respectively; (c)–(g) EtOH solution of *p*-hydroxybenzyl alcohol was sonicated for 0.5 h then refluxed for 1.0 h, 2.0 h, 4.0 h, and 6.0 h, respectively.

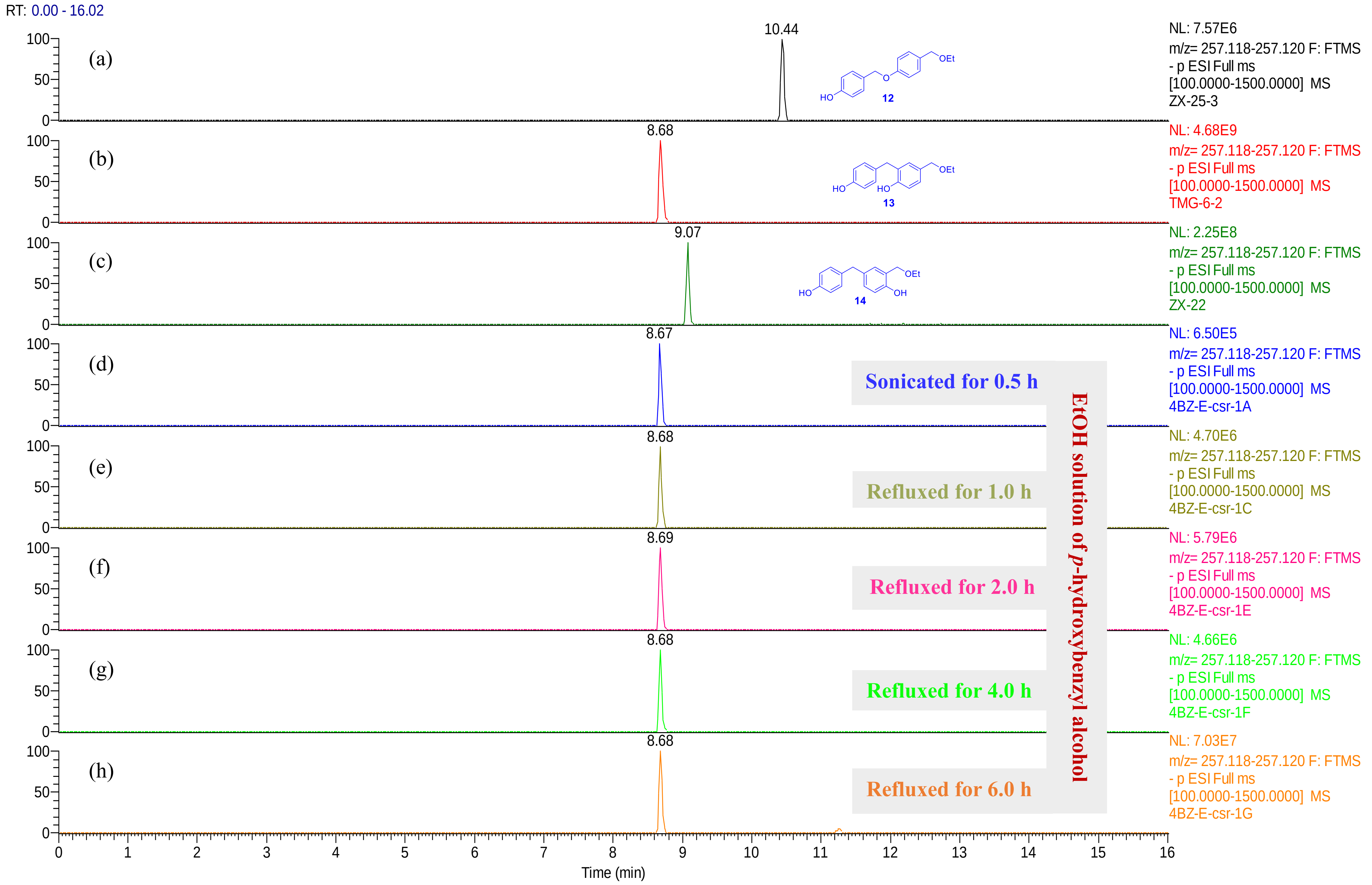

Fig. S209 Overlaid chromatograms of the extracted negative ion at *m/z* 257.119 [M–H]⁻: (a)–(c) compounds **12**, **13**, and **14** in CH_3CN, respectively; (d)–(h) EtOH solution of *p*-hydroxybenzyl alcohol was sonicated for 0.5 h then refluxed for 1.0 h, 2.0 h, 4.0 h, and 6.0 h, respectively.

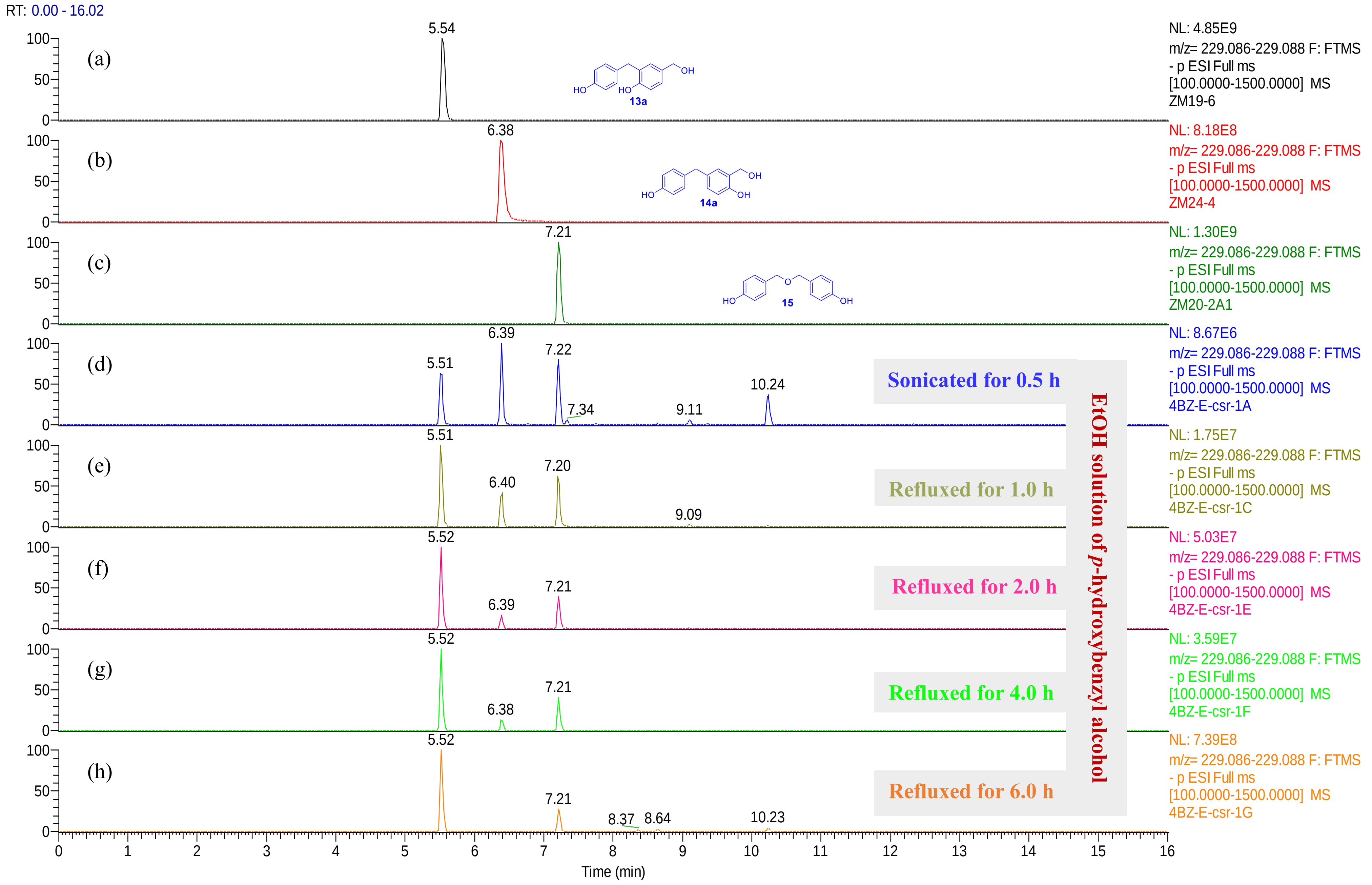

Fig. S210 Overlaid chromatograms of the extracted negative ion at *m/z* 229.087 [M–H]⁻: (a)–(c) compounds **13a**, **14a**, and **15** in CH_3CN, respectively; (d)–(h) EtOH solution of *p*-hydroxybenzyl alcohol was sonicated for 0.5 h then refluxed for 1.0 h, 2.0 h, 4.0 h, and 6.0 h, respectively.

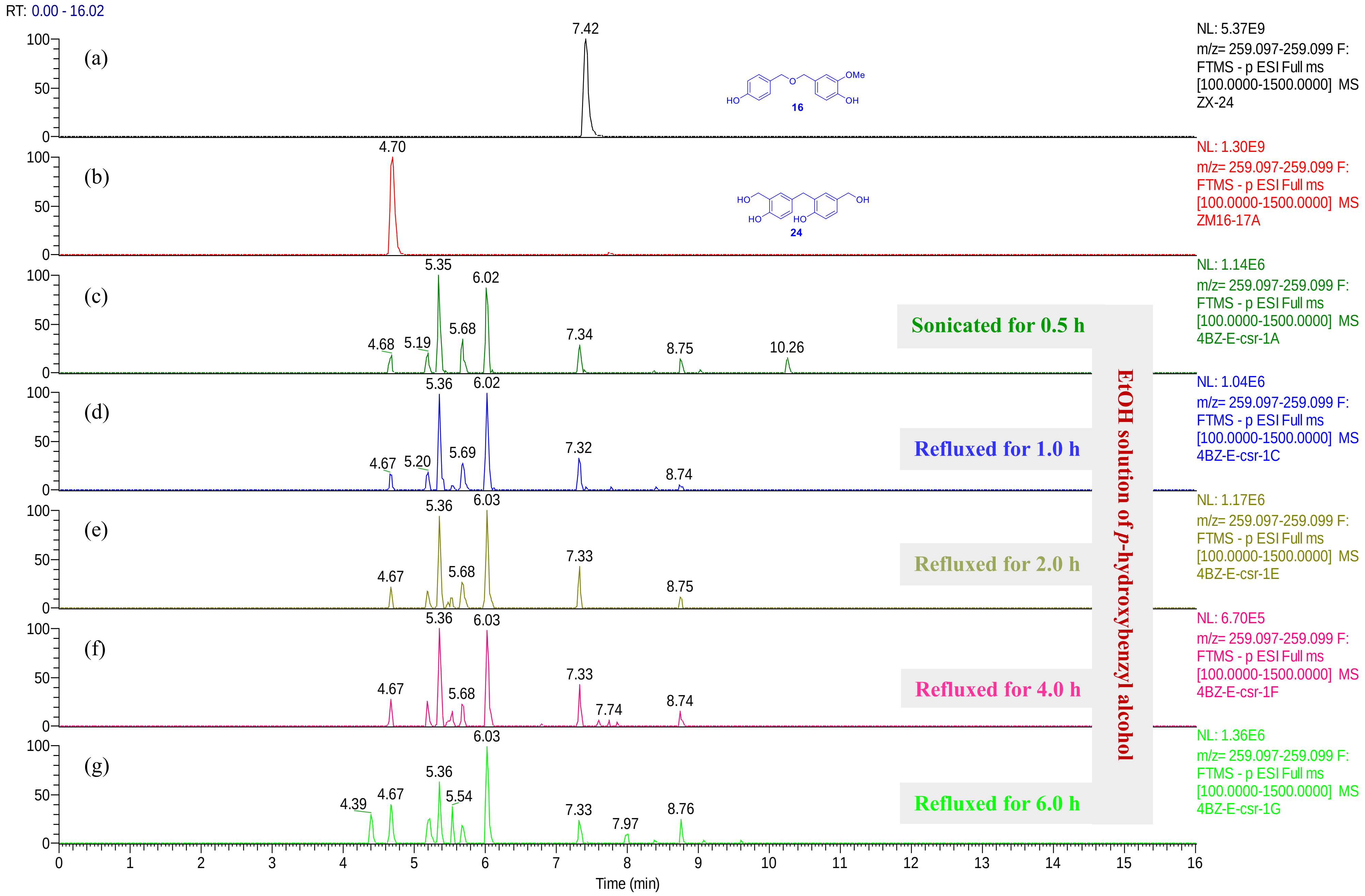

Fig. S211 Overlaid chromatograms of the extracted negative ion at *m/z* 259.098 [M–H]$^-$: (a) and (b) compounds **16** and **24** in CH_3CN, respectively; (c)–(g) EtOH solution of *p*-hydroxybenzyl alcohol was sonicated for 0.5 h then refluxed for 1.0 h, 2.0 h, 4.0 h, and 6.0 h, respectively.

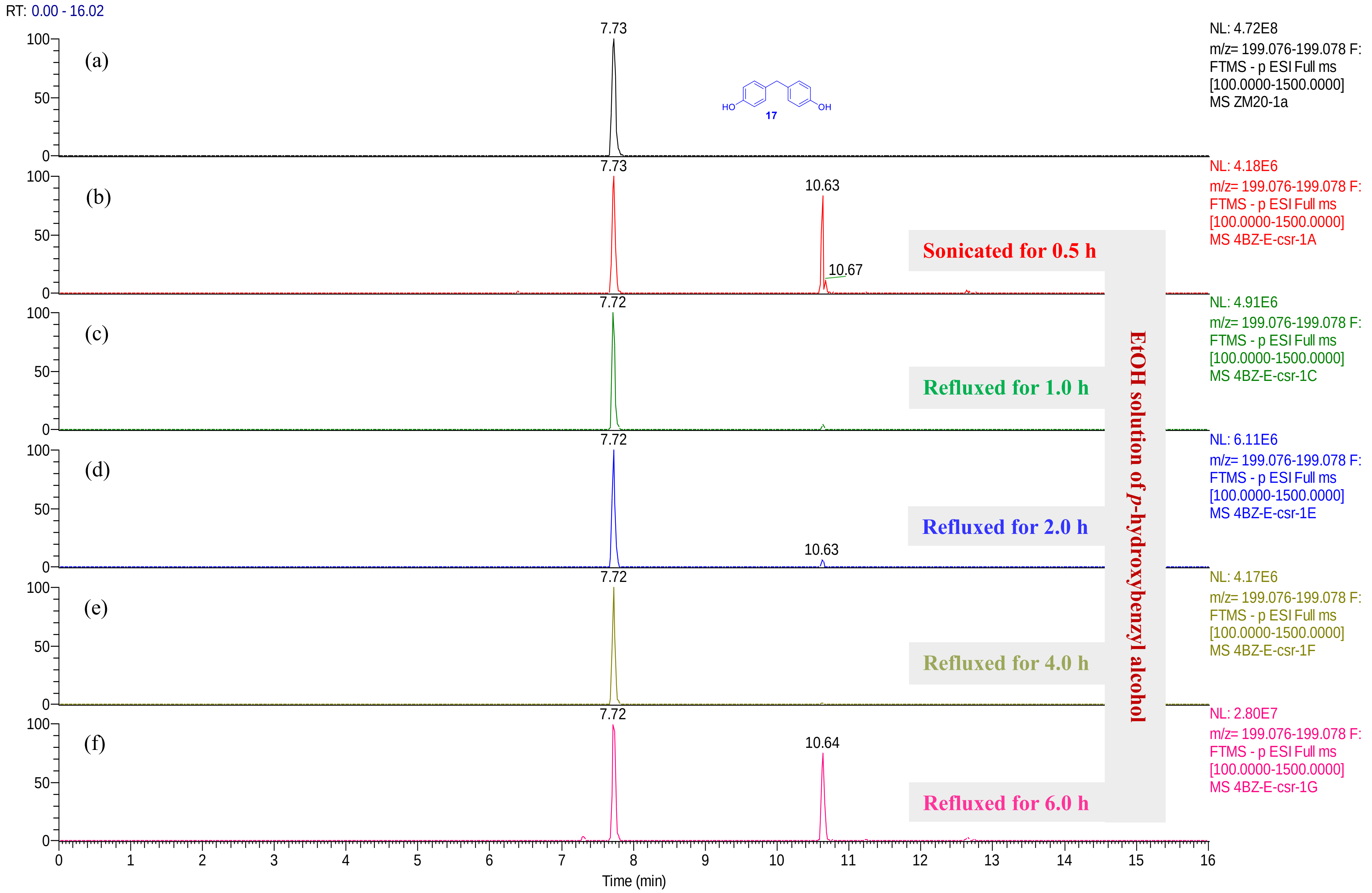

Fig. S212 Overlaid chromatograms of the extracted negative ion at *m/z* 199.077 [M–H]$^-$: (a) compound **17** in CH_3CN; (b)–(f) EtOH solution of *p*-hydroxybenzyl alcohol was sonicated for 0.5 h then refluxed for 1.0 h, 2.0 h, 4.0 h, and 6.0 h, respectively.

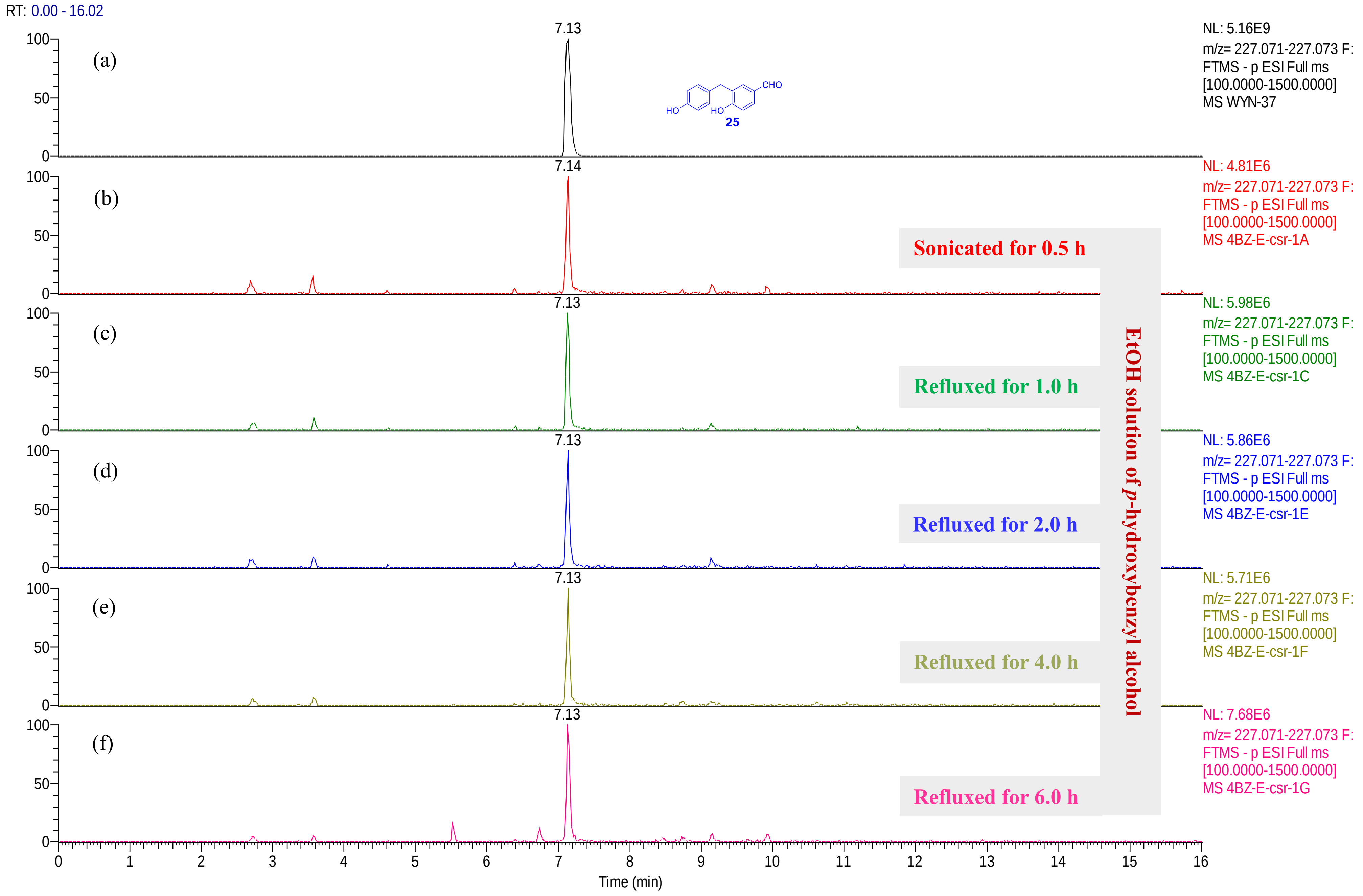

Fig. S213 Overlaid chromatograms of the extracted negative ion at *m/z* 227.072 $[M-H]^-$: (a) compound **25** in CH_3CN; (b)–(f) EtOH solution of *p*-hydroxybenzyl alcohol was sonicated for 0.5 h then refluxed for 1.0 h, 2.0 h, 4.0 h, and 6.0 h, respectively.

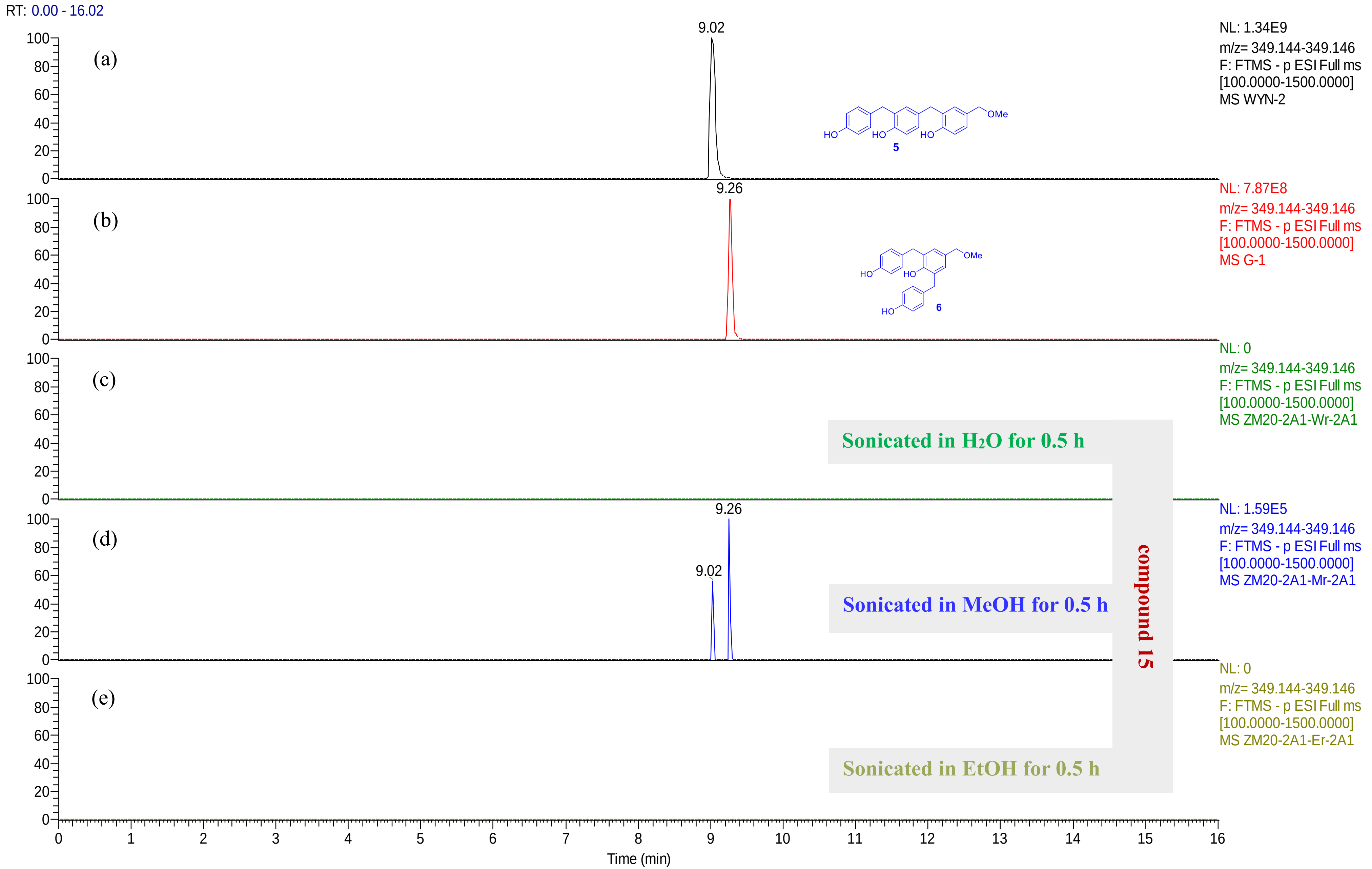

Fig. S214 Overlaid chromatograms of the extracted negative ion at *m/z* 349.145 $[M-H]^-$: (a) and (b) compounds **5** and **6** in CH_3CN, respectively; (c)–(e) H_2O, MeOH, and EtOH solutions of compound **15** were sonicated for 0.5 h, respectively.

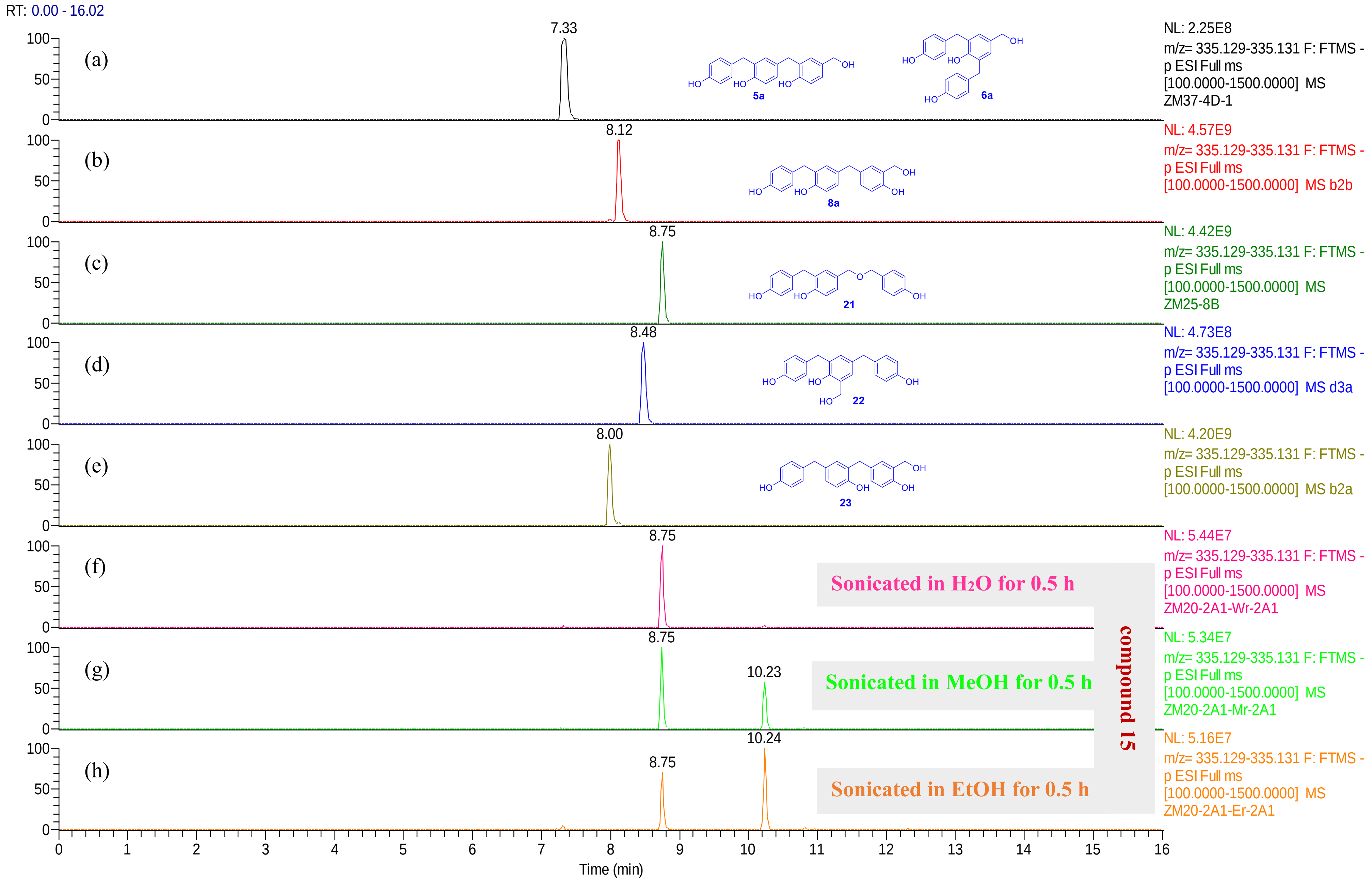

Fig. S215 Overlaid chromatograms of the extracted negative ion at *m/z* 335.130 $[M-H]^-$: (a)–(e) compounds **5a**/**6a**, **8a**, and **21**–**23** in CH_3CN, respectively; (f)–(h) H_2O, MeOH, and EtOH solutions of compound **15** were sonicated for 0.5 h, respectively.

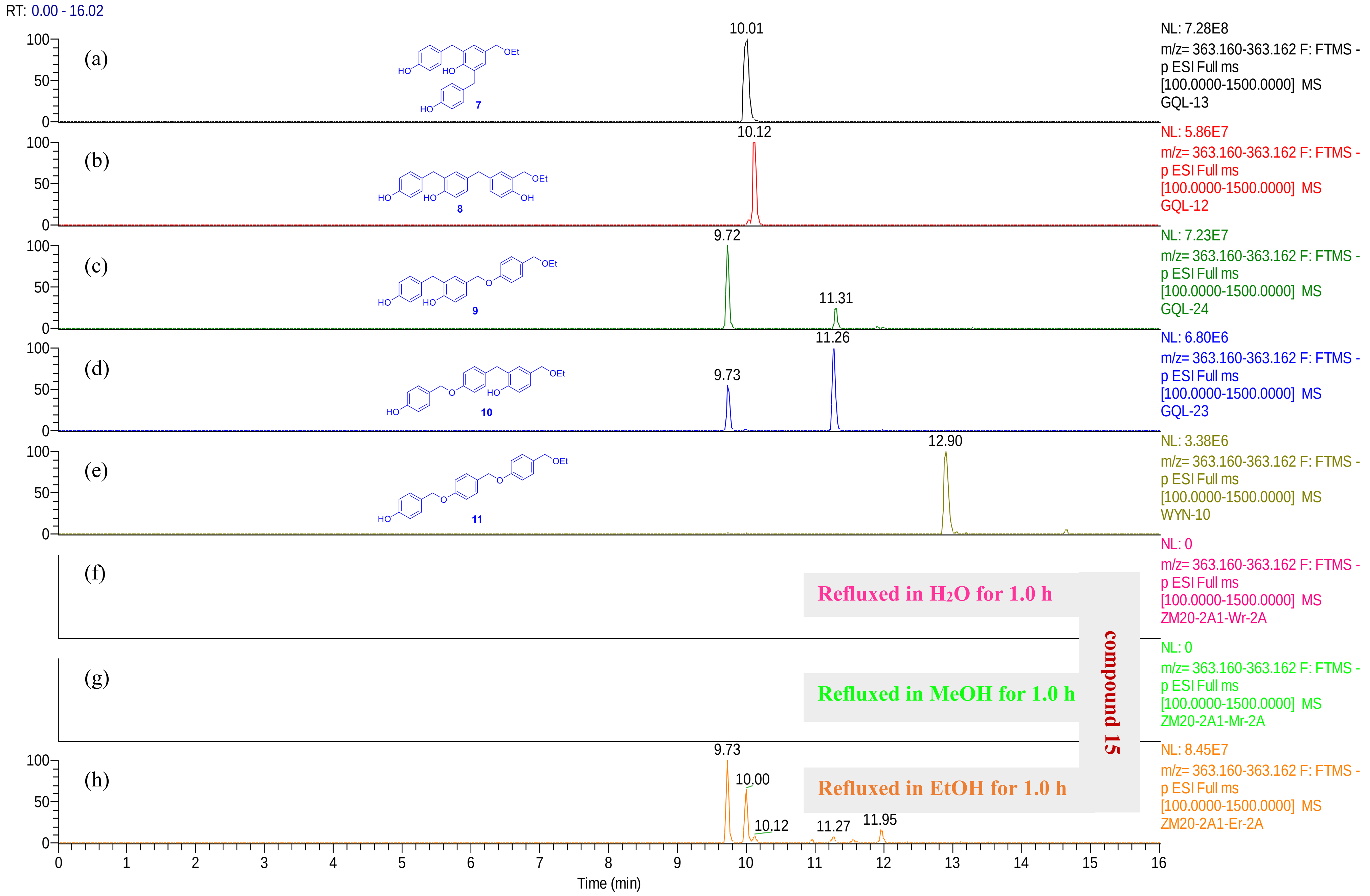

Fig. S216 Overlaid chromatograms of the extracted negative ion at *m/z* 363.161 $[M-H]^-$: (a)–(e) compounds **7–11** in CH_3CN, respectively; (f)–(h) H_2O, MeOH, and EtOH solutions of compound **15** were sonicated for 0.5 h then refluxed for 1.0 h, respectively.

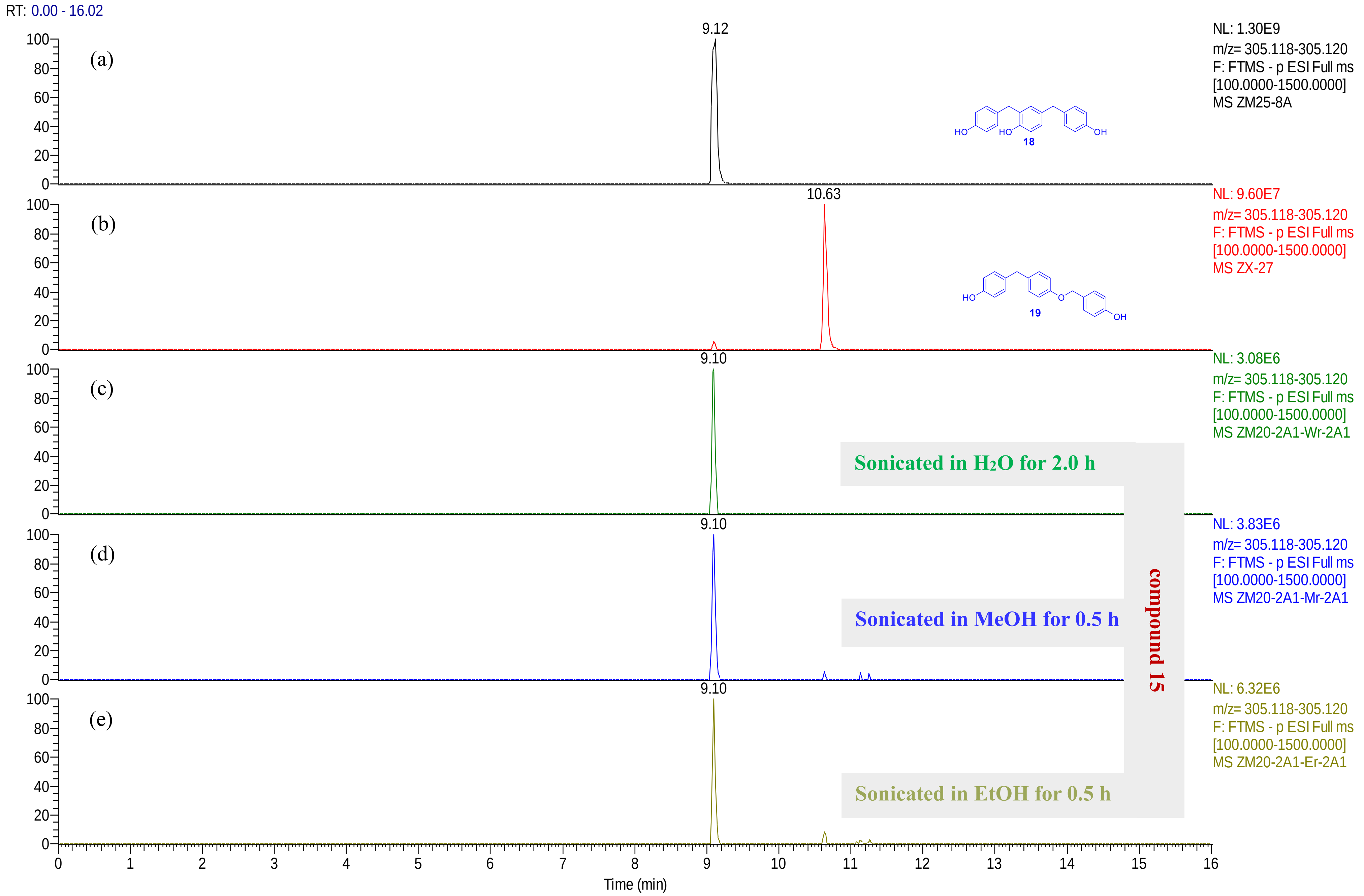

Fig. S217 Overlaid chromatograms of the extracted negative ion at *m/z* 305.119 [M–H]$^-$: (a) and (b) compounds **18** and **19** in CH_3CN, respectively; (c)–(e) H_2O, MeOH, and EtOH solutions of compound **15** were sonicated for 0.5 h, respectively.

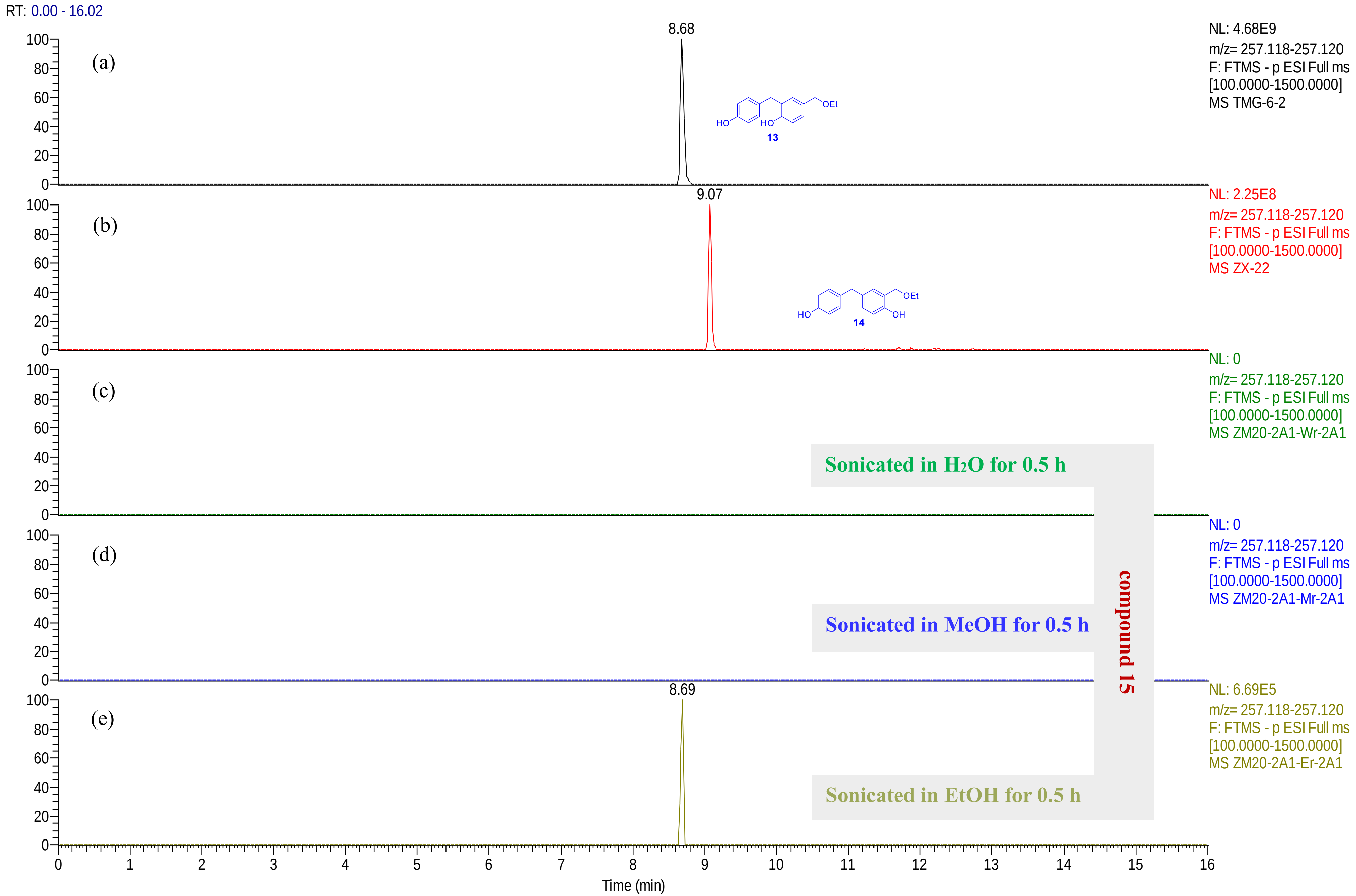

Fig. S218 Overlaid chromatograms of the extracted negative ion at *m/z* 257.119 $[M-H]^-$: (a) and (b) compounds **13** and **14** in CH_3CN, respectively; (c)–(d) H_2O, MeOH, and EtOH solutions of compound **15** were sonicated for 0.5 h, respectively.

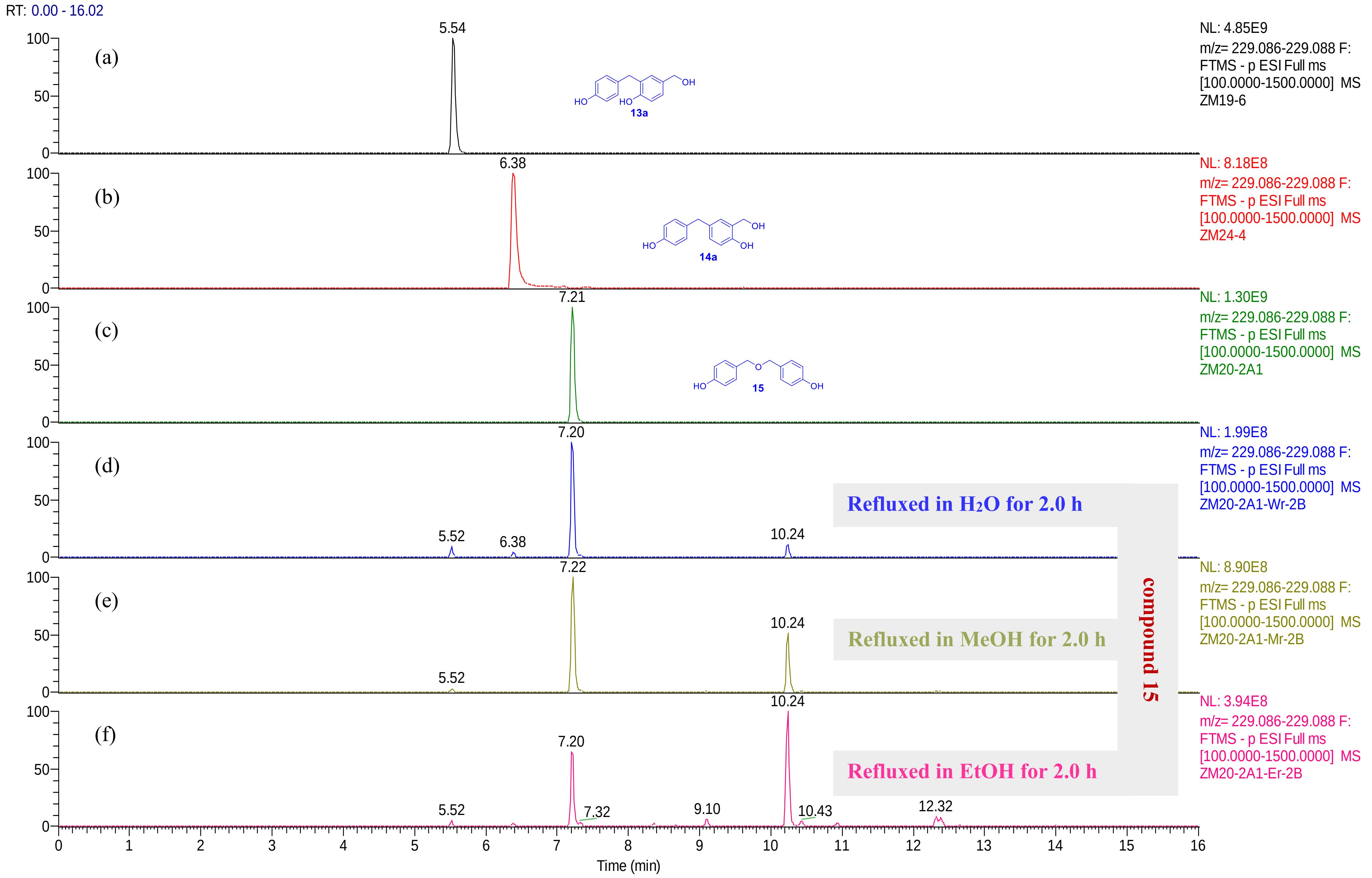

Fig. S219 Overlaid chromatograms of the extracted negative ion at *m/z* 229.087 [M–H]$^-$: (a)–(c) compounds **13a**, **14a**, and **15** in CH_3CN, respectively; (d)–(g) H_2O, MeOH, and EtOH solutions of compound **15** were sonicated for 0.5 h then refluxed for 2.0 h, respectively.

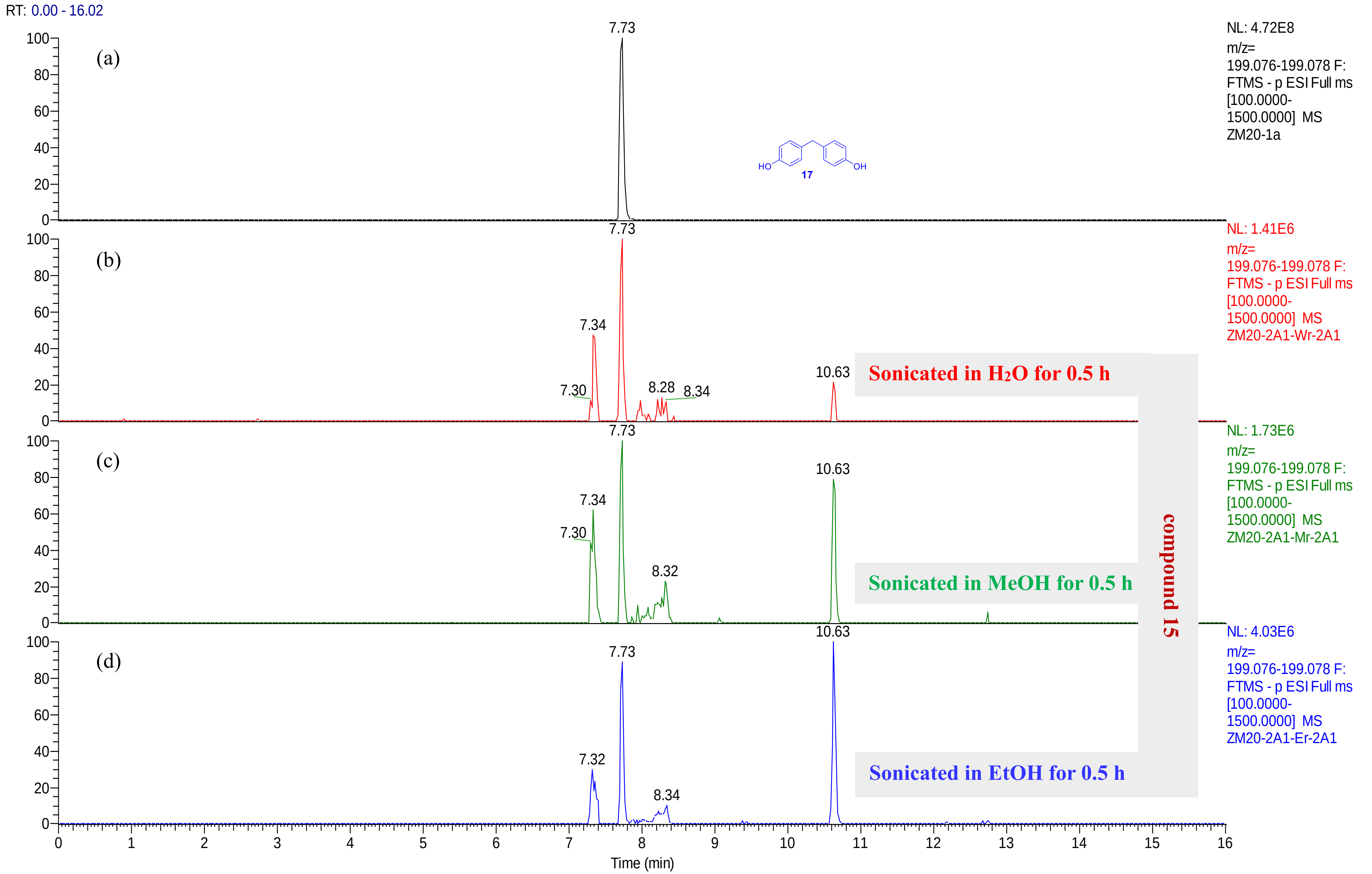

Fig. S220 Overlaid chromatograms of the extracted negative ion at *m/z* 199.077 [M–H]⁻: (a) compound **17** in CH_3CN; (b)–(d) H_2O, MeOH, and EtOH solutions of compound **15** were sonicated for 0.5 h, respectively.

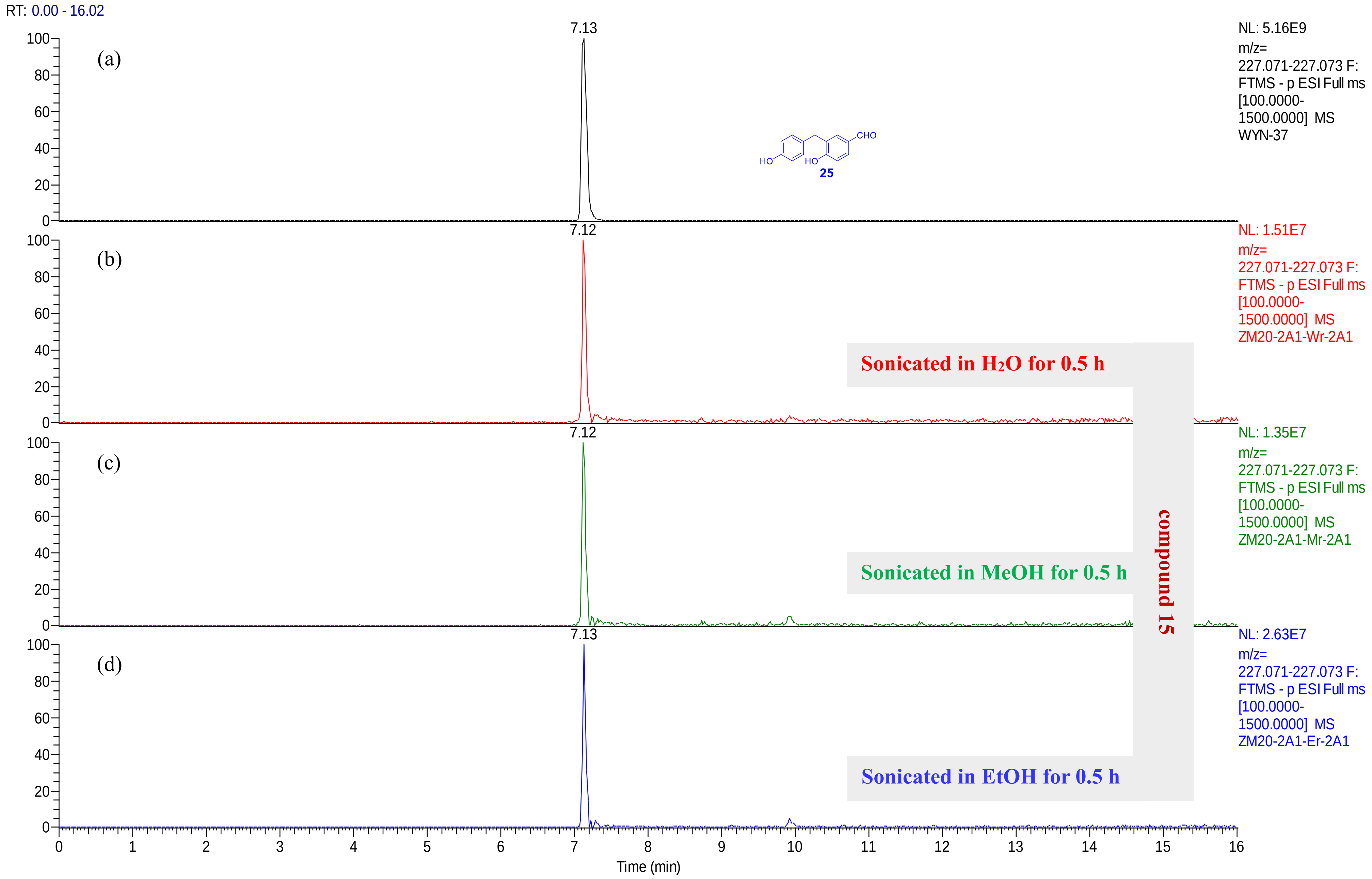

Fig. S221 Overlaid chromatograms of the extracted negative ion at *m/z* 227.072 $[M-H]^-$: (a) compound **25** in CH_3CN; (b)–(d) H_2O, MeOH, and EtOH solutions of compound **15** were sonicated for 0.5 h, respectively.

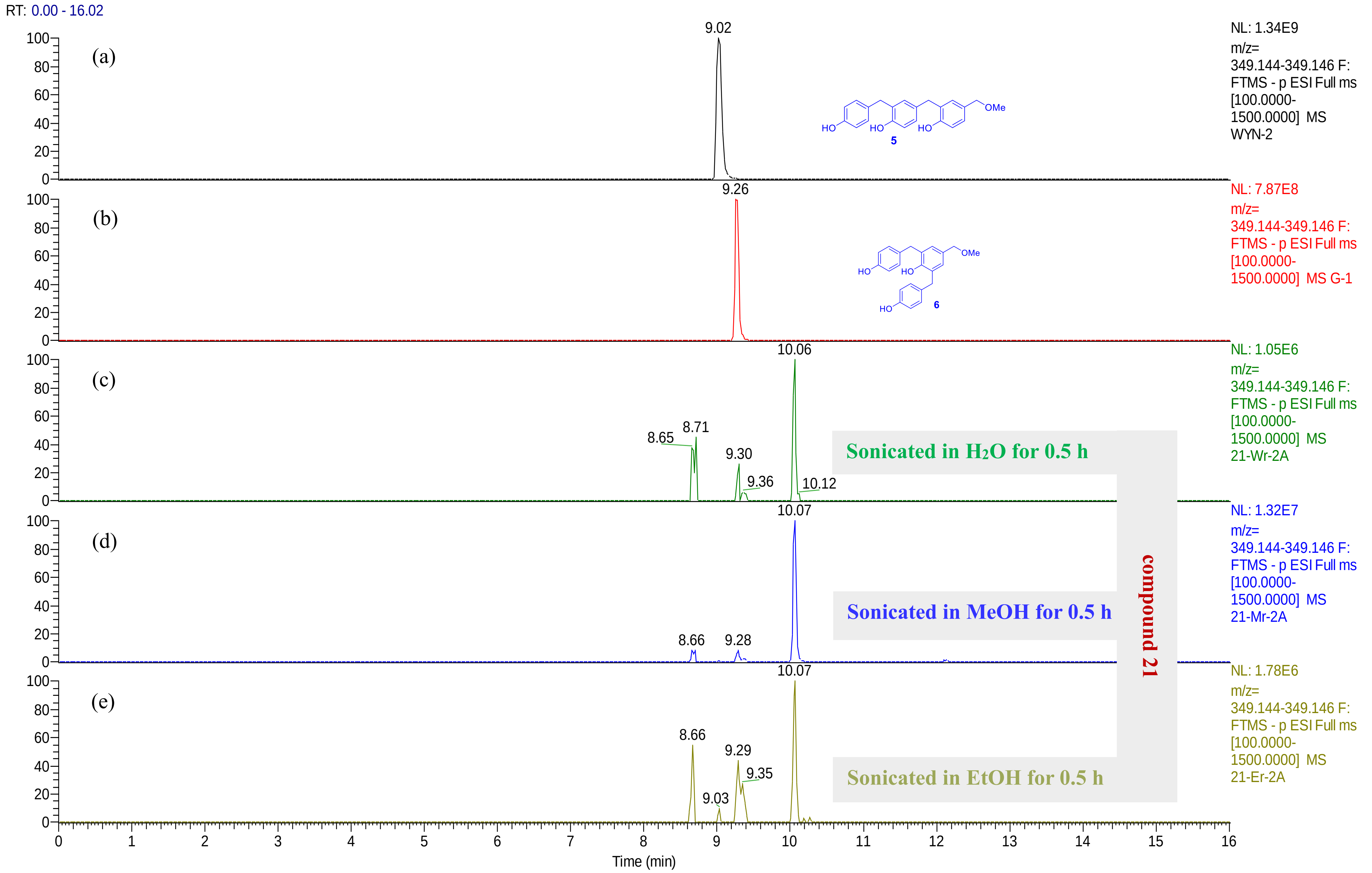

Fig. S222 Overlaid chromatograms of the extracted negative ion at *m/z* 349.145 $[M-H]^-$: (a) and (b) compounds **5** and **6** in CH_3CN, respectively; (c)–(e) H_2O, MeOH, and EtOH solutions of compound **21** were sonicated for 0.5 h, respectively.

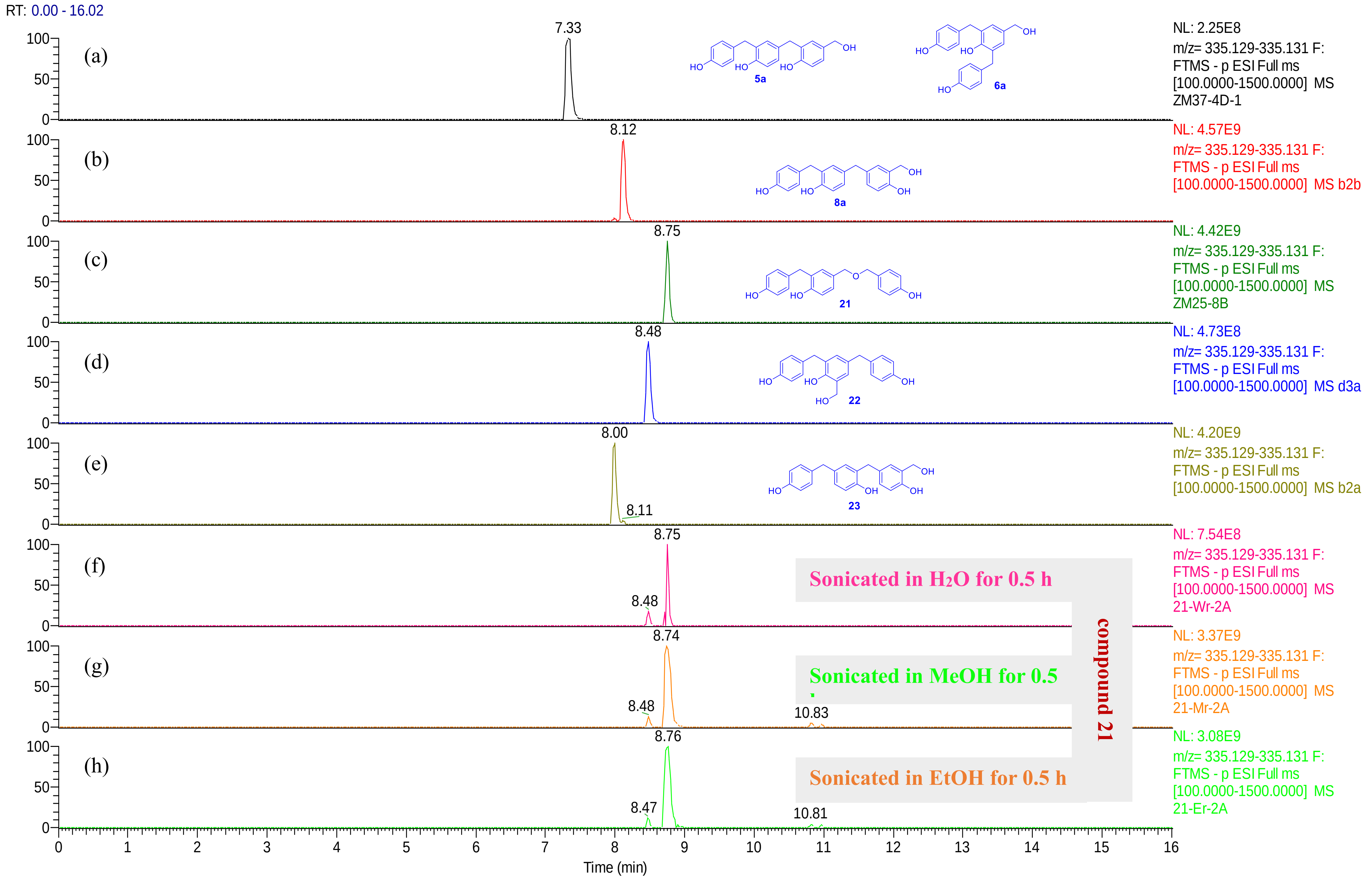

Fig. S223 Overlaid chromatograms of the extracted negative ion at *m/z* 335.130 $[M-H]^-$: (a)–(e) compounds **5a**/**6a**, **8a**, and **21**–**23** in CH_3CN, respectively; (f)–(h) H_2O, MeOH, and EtOH solutions of compound **21** were sonicated for 0.5 h, respectively.

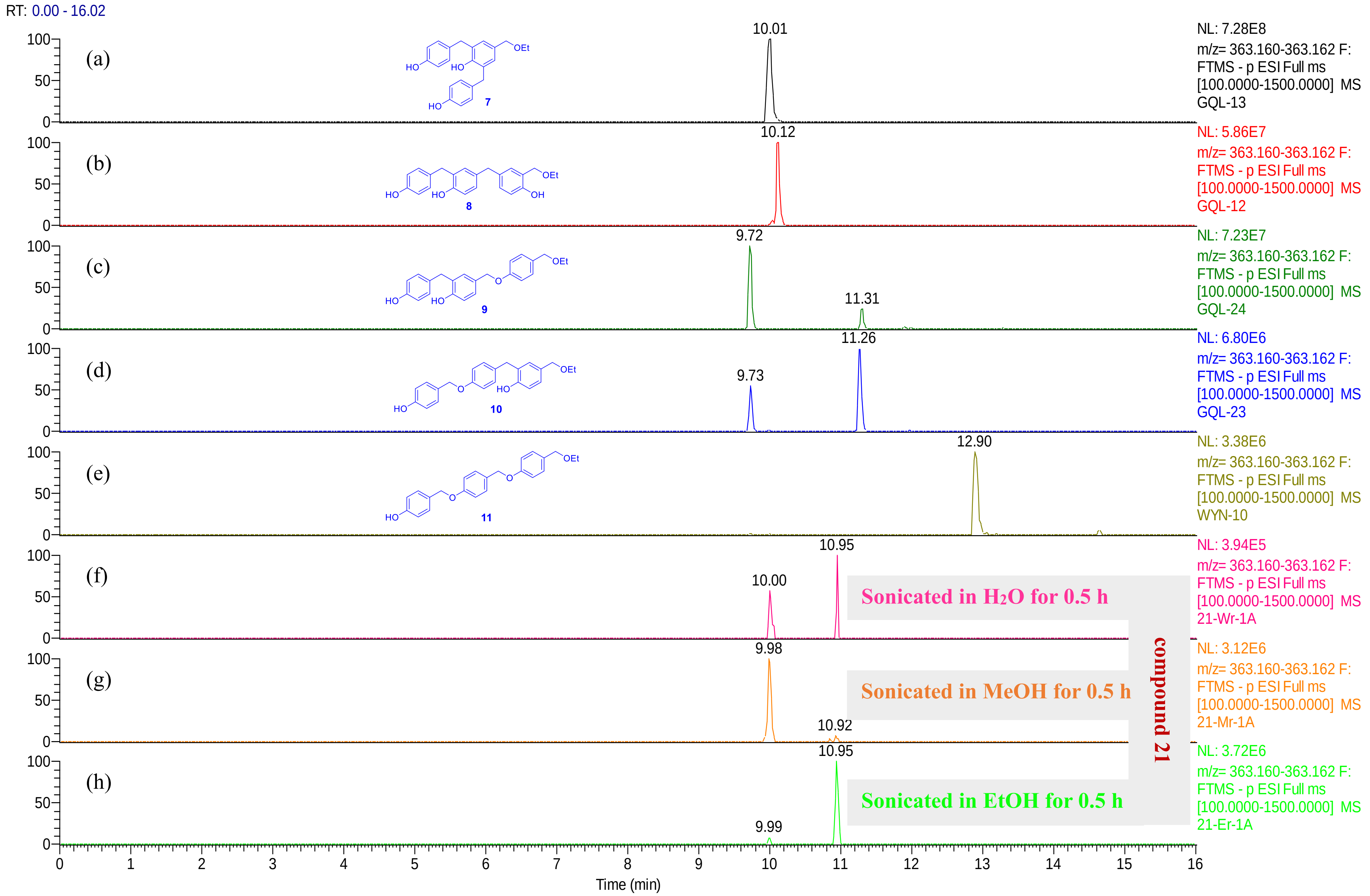

Fig. S224 Overlaid chromatograms of the extracted negative ion at *m/z* 363.161 $[M-H]^-$: (a)–(e) compounds **7–11** in CH_3CN, respectively; (f)–(h) H_2O, MeOH, and EtOH solutions of compound **21** were sonicated for 0.5 h, respectively.

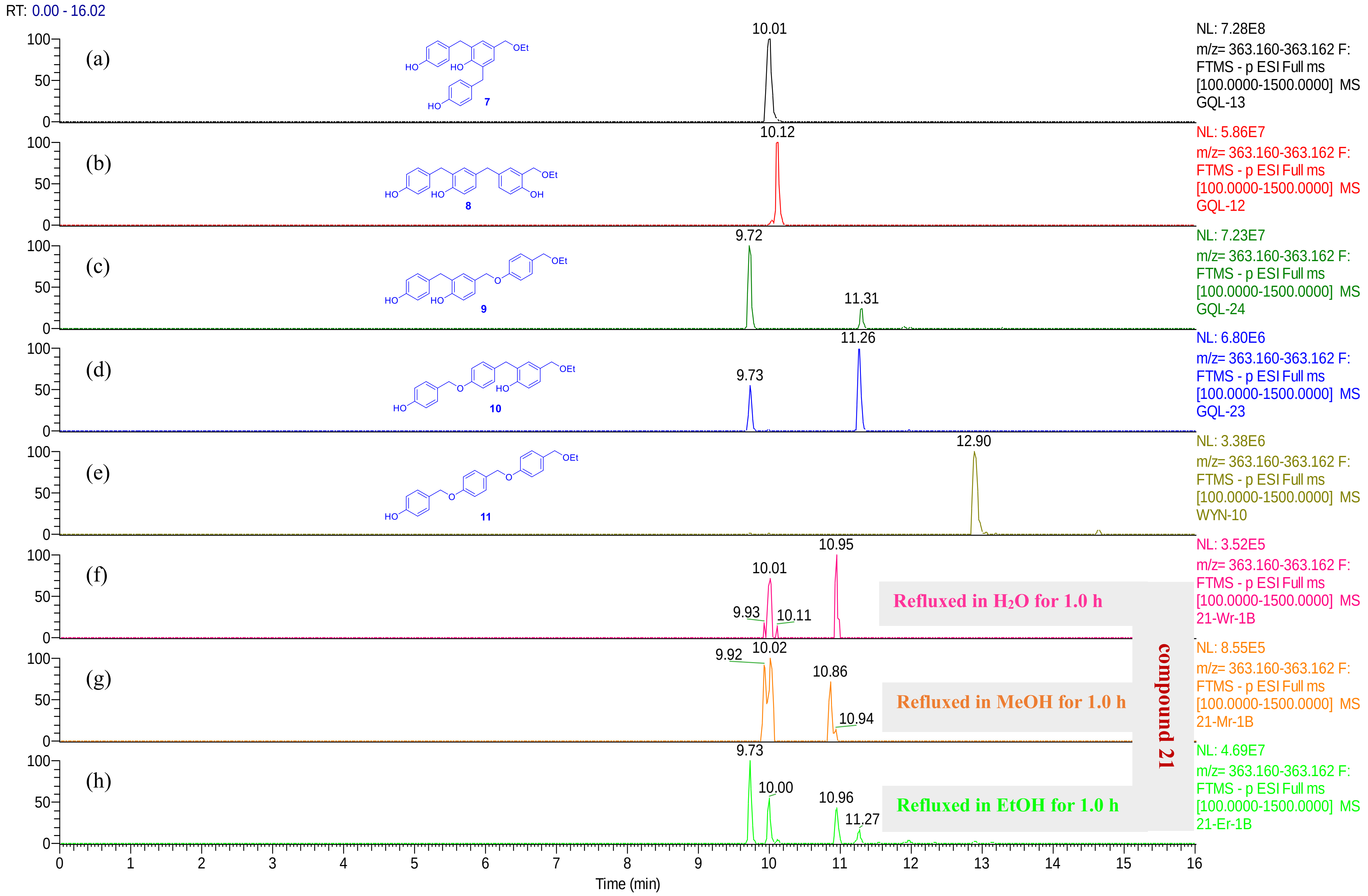

Fig. S225 Overlaid chromatograms of the extracted negative ion at *m/z* 363.161 $[M-H]^-$: (a)–(e) compounds **7**–**11** in CH_3CN, respectively; (f)–(h) H_2O, MeOH, and EtOH solutions of compound **21** were sonicated for 0.5 h then refluxed for 1.0 h, respectively.

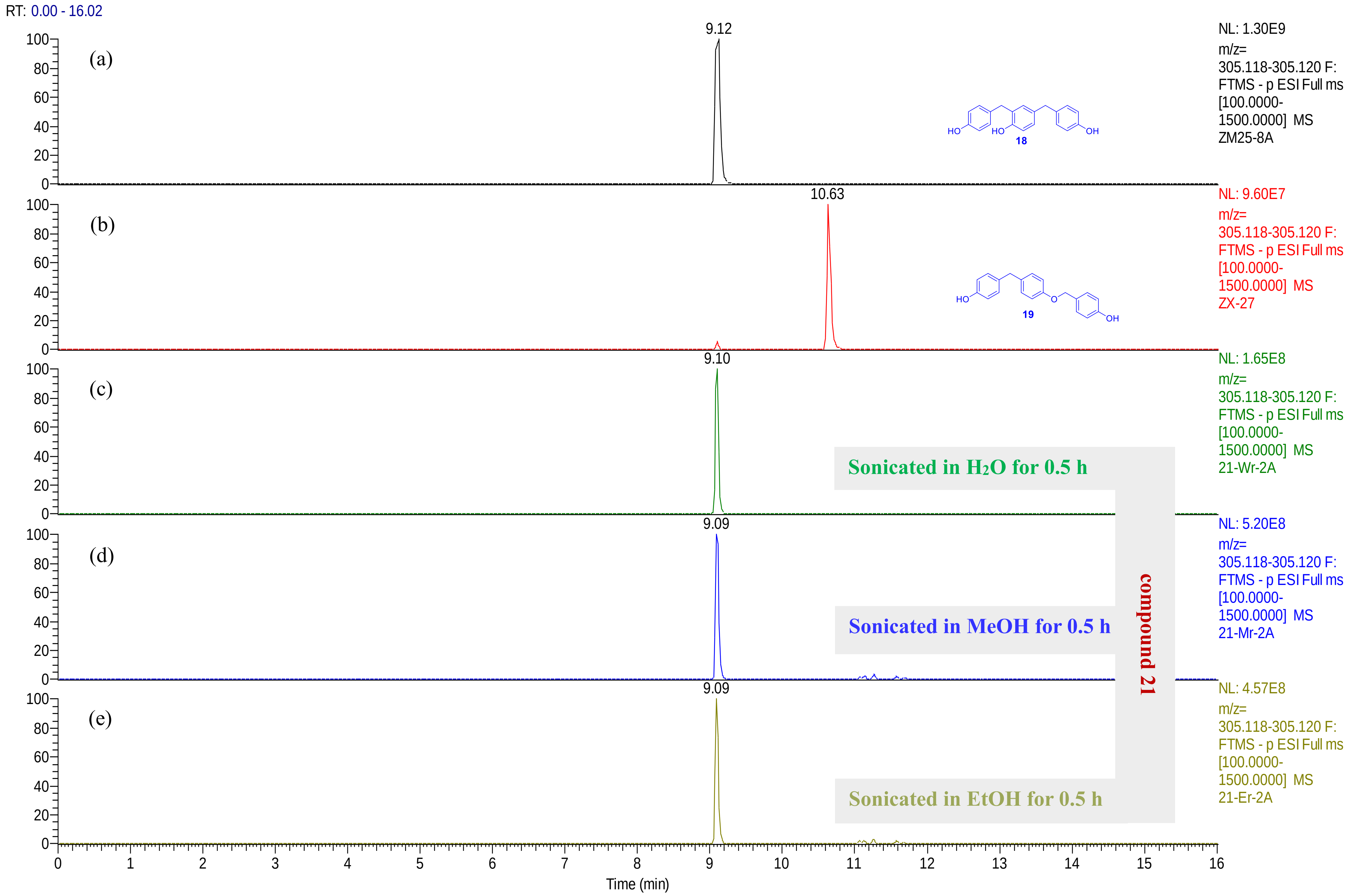

Fig. S226 Overlaid chromatograms of the extracted negative ion at *m/z* 305.119 $[M{-}H]^-$: (a) and (b) compounds **18** and **19** in CH_3CN, respectively; (c)–(e) H_2O, MeOH, and EtOH solutions of compound **21** were sonicated for 0.5 h, respectively.

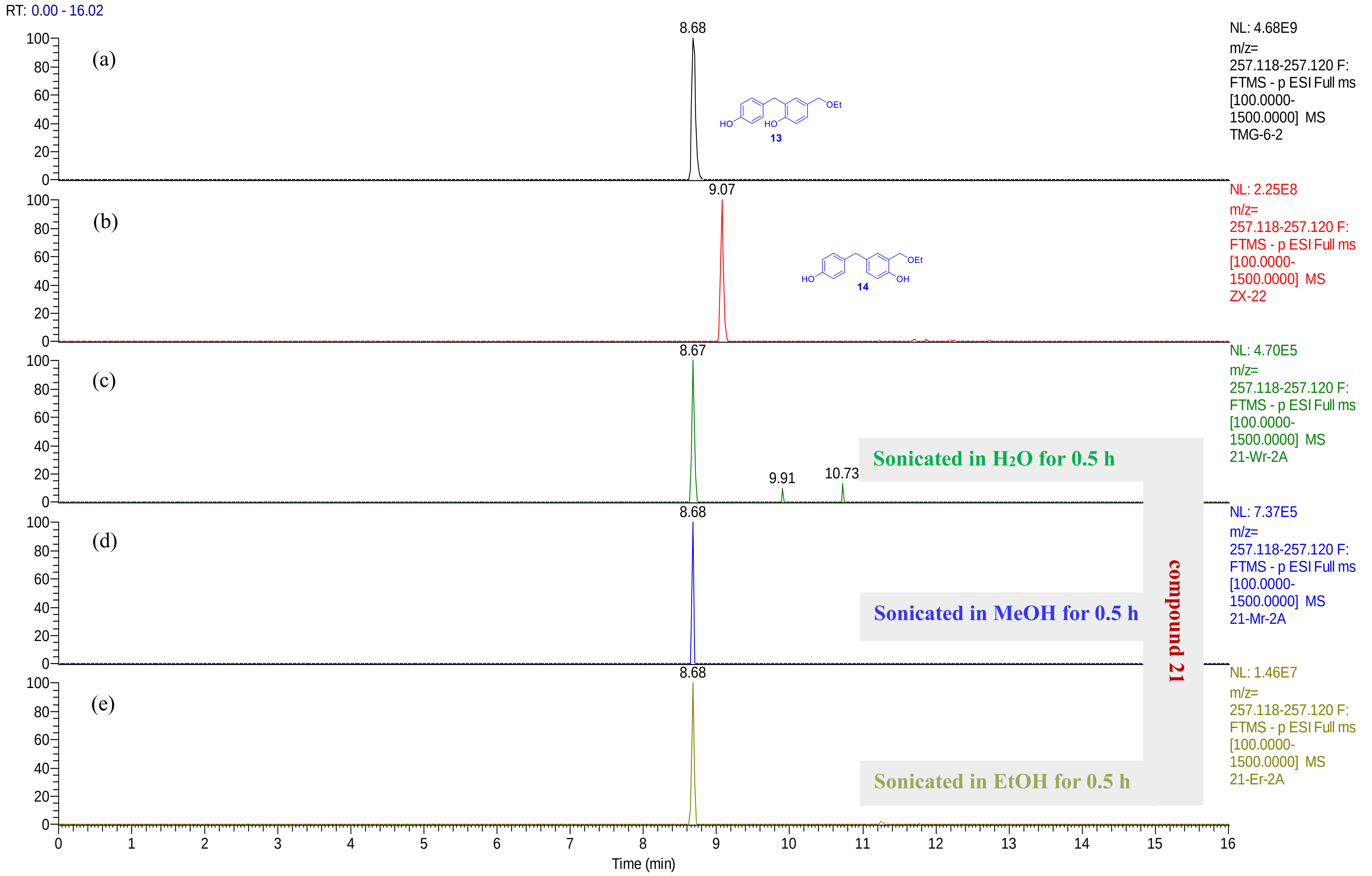

Fig. S227 Overlaid chromatograms of the extracted negative ion at *m/z* 257.119 $[M-H]^-$: (a) and (b) compounds **13** and **14** in CH_3CN, respectively; (c)–(d) H_2O, MeOH, and EtOH solutions of compound **21** were sonicated for 0.5 h, respectively.

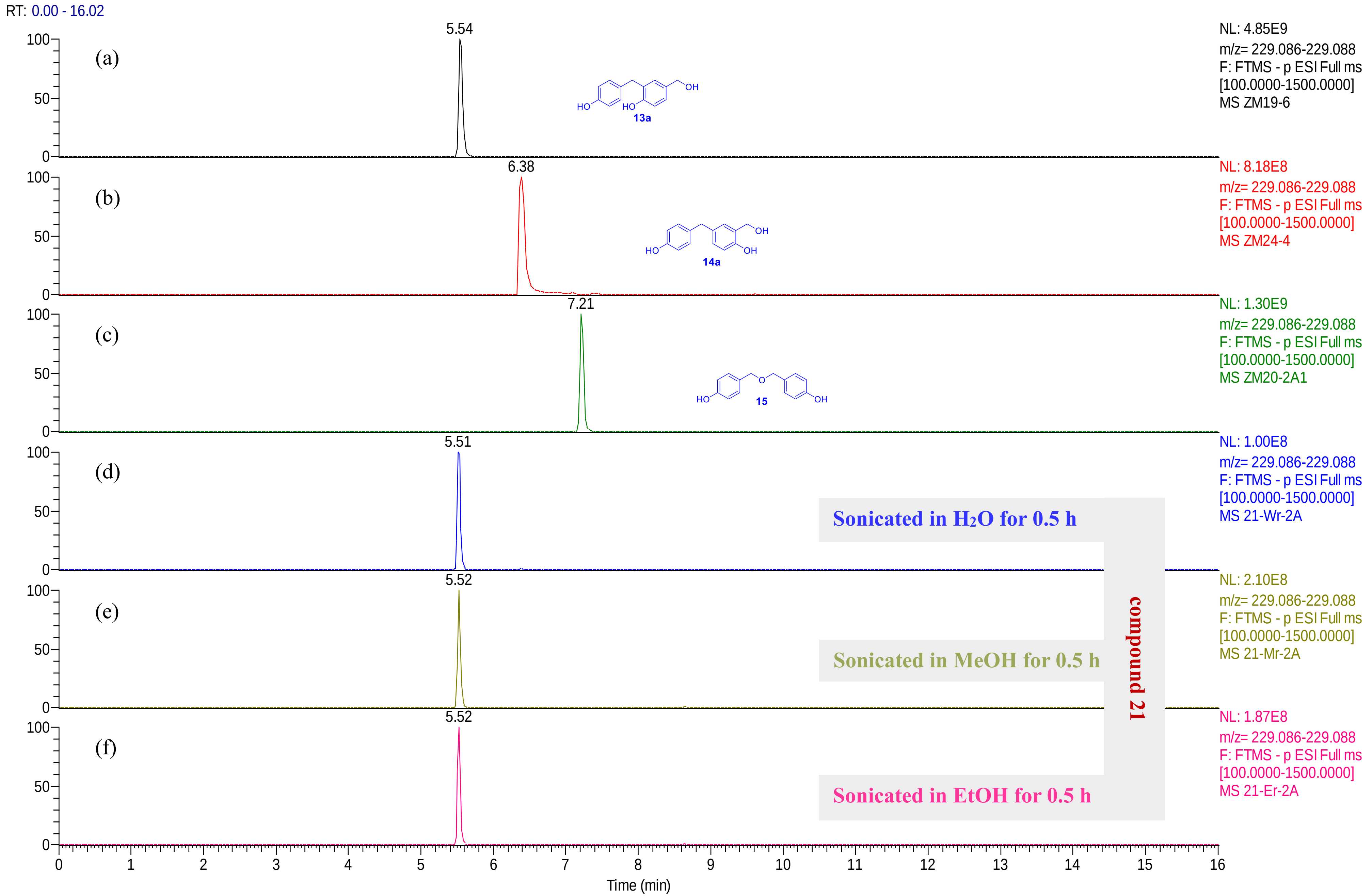

Fig. S228 Overlaid chromatograms of the extracted negative ion at *m/z* 229.087 $[M-H]^-$: (a)–(c) compounds **13a**, **14a**, and **15** in CH_3CN, respectively; (d)–(g) H_2O, MeOH, and EtOH solutions of compound **21** were sonicated for 0.5 h, respectively.

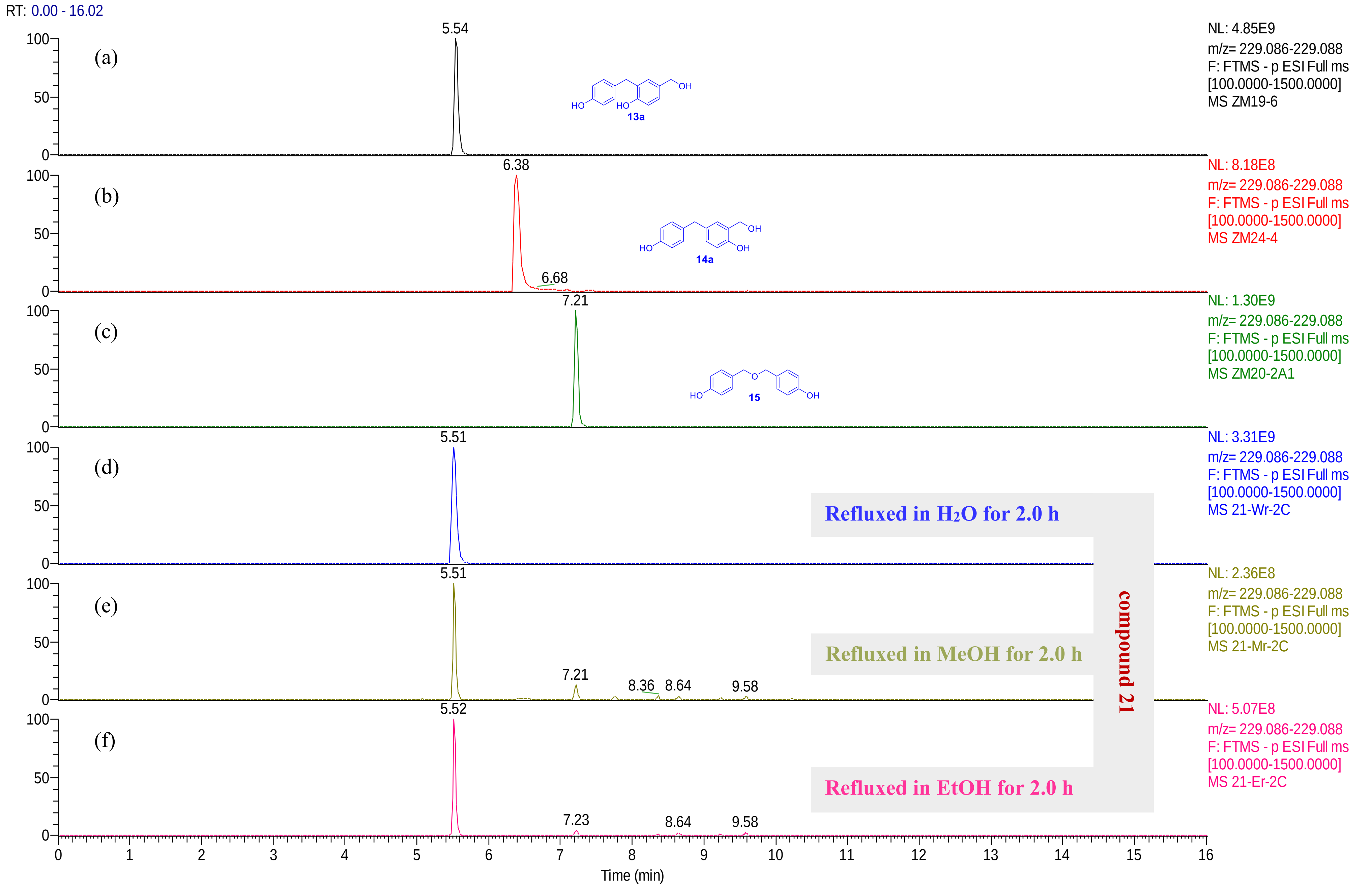

Fig. S229 Overlaid chromatograms of the extracted negative ion at *m/z* 229.087 $[M-H]^-$: (a)–(c) compounds **13a**, **14a**, and **15** in CH_3CN, respectively; (d)–(g) H_2O, MeOH, and EtOH solutions of compound **21** were sonicated for 0.5 h then refluxed for 2.0 h, respectively.

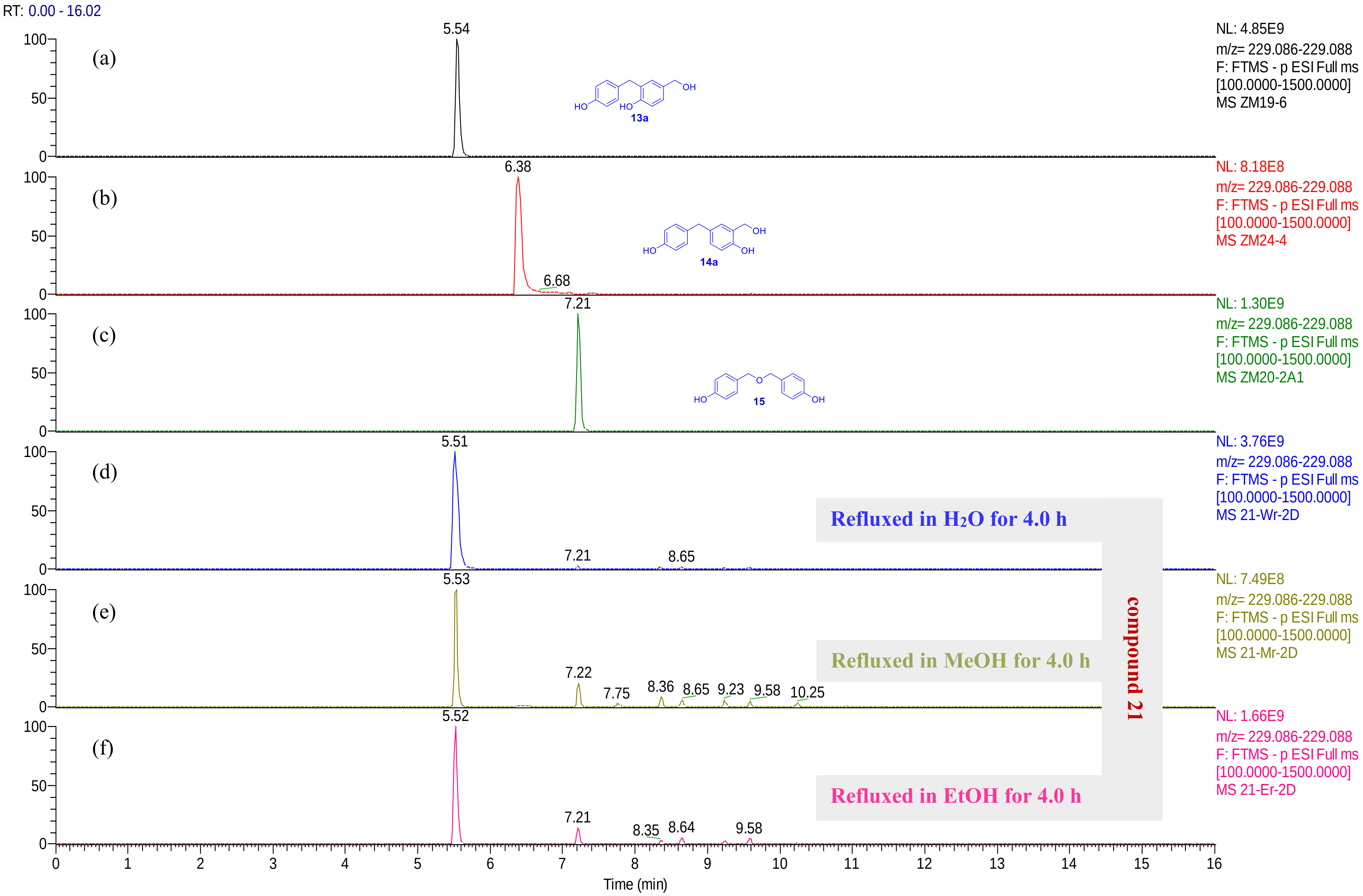

Fig. S230 Overlaid chromatograms of the extracted negative ion at *m/z* 229.087 $[M-H]^-$: (a)–(c) compounds **13a**, **14a**, and **15** in CH_3CN, respectively; (d)–(g) H_2O, MeOH, and EtOH solutions of compound **21** were sonicated for 0.5 h then refluxed for 4 h, respectively.

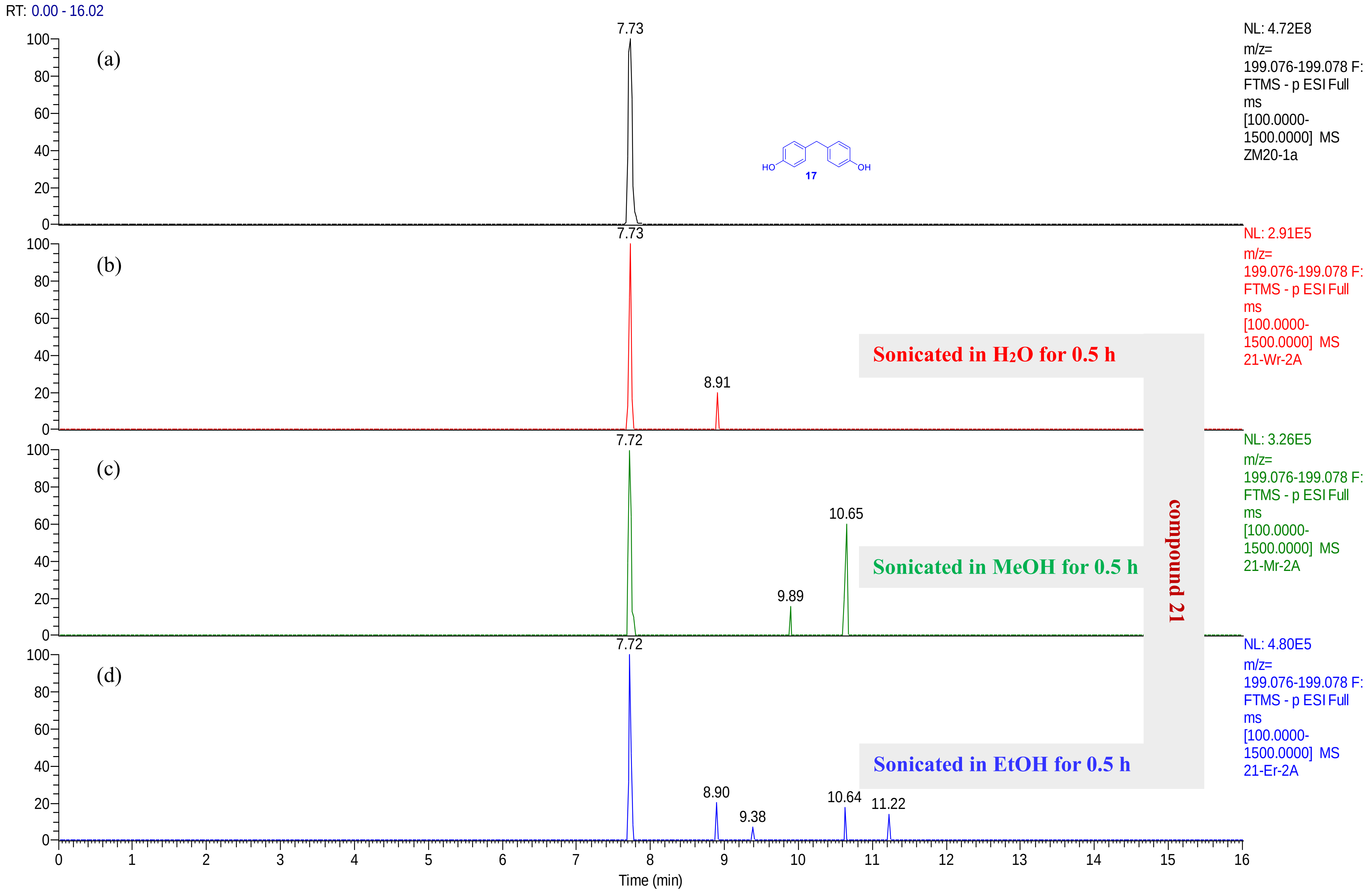

Fig. S231 Overlaid chromatograms of the extracted negative ion at *m/z* 199.077 $[M-H]^-$: (a) compound **17** in CH_3CN; (b)–(d) H_2O, MeOH, and EtOH solutions of compound **21** were sonicated for 0.5 h, respectively.

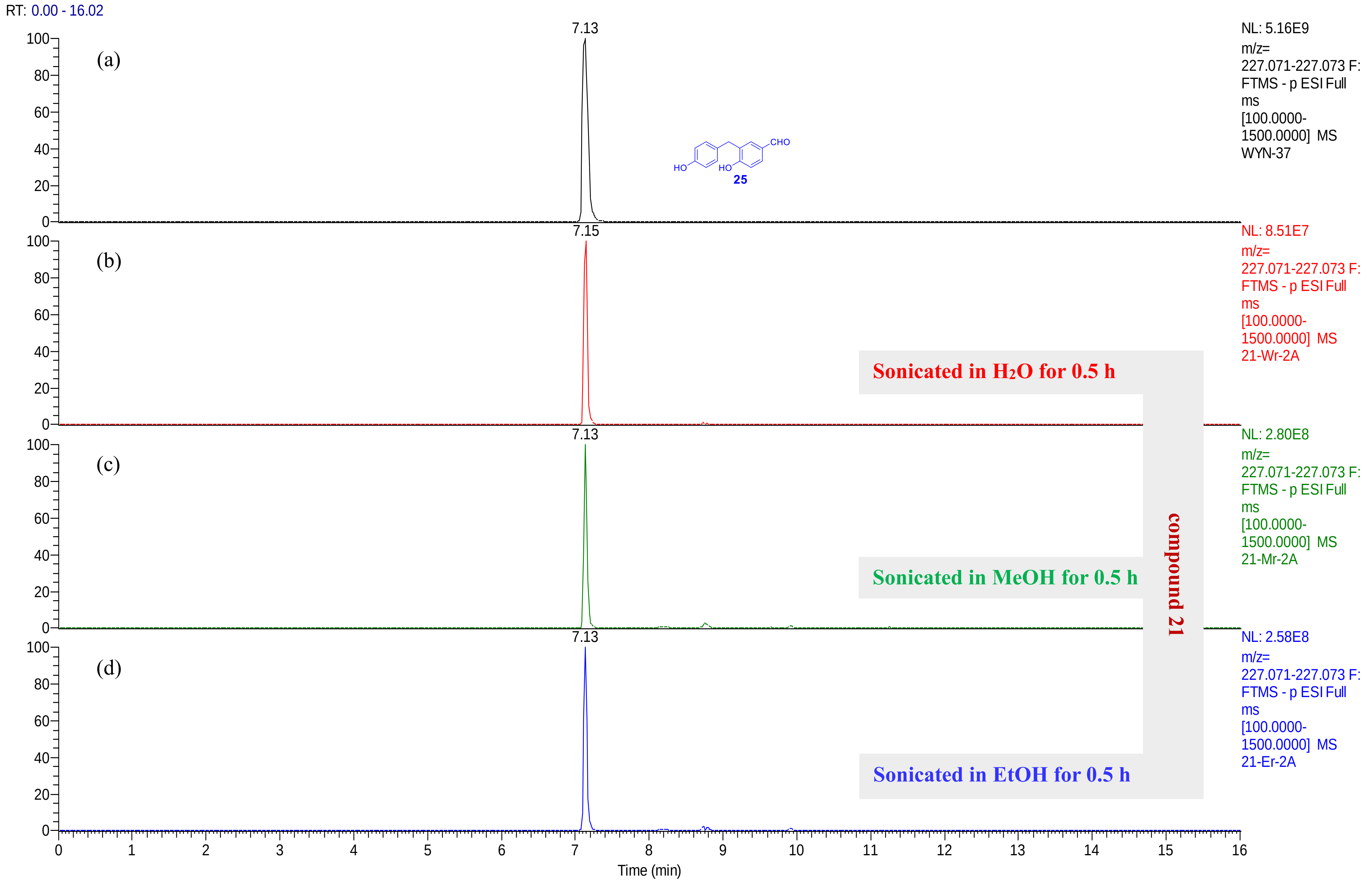

Fig. S232 Overlaid chromatograms of the extracted negative ion at *m/z* 227.072 $[M{-}H]^-$: (a) compound **25** in CH_3CN; (b)–(d) H_2O, MeOH, and EtOH solutions of compound **21** were sonicated for 0.5 h, respectively.

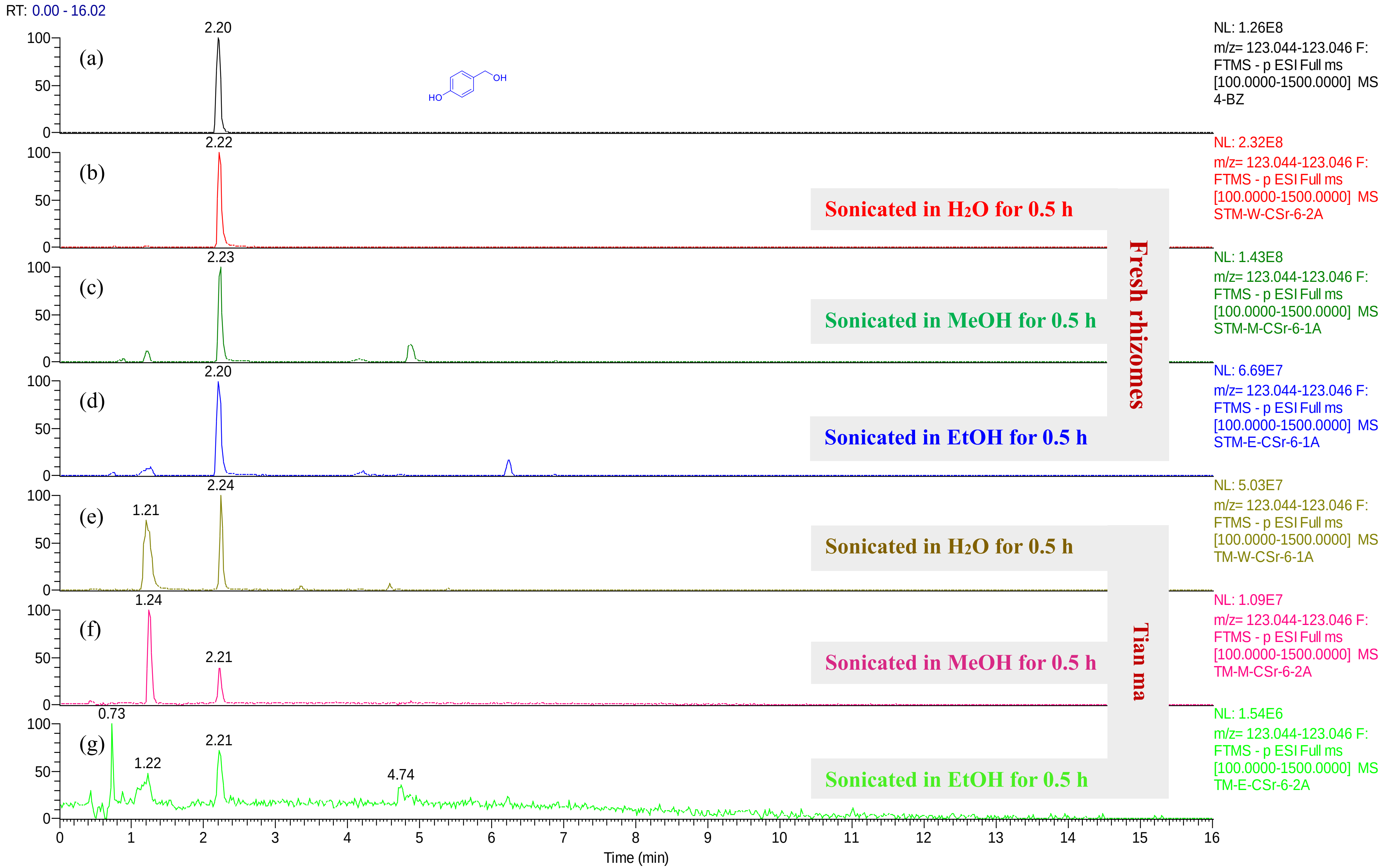

Fig. S233 Overlaid chromatograms of the extracted negative ion at *m/z* 123.045 $[M-H]^-$: (a) *p*-hydroxybenzyl alcohol in CH_3CN; (b)–(d) extracts obtained by sonicating of fresh *G. elata* rhizomes with H_2O, MeOH, and EtOH, respectively; (e)–(g) extracts obtained by sonicating of "tian ma" (the steamed and dried *G. elata* rhizomes) with H_2O, MeOH, and EtOH, respectively.

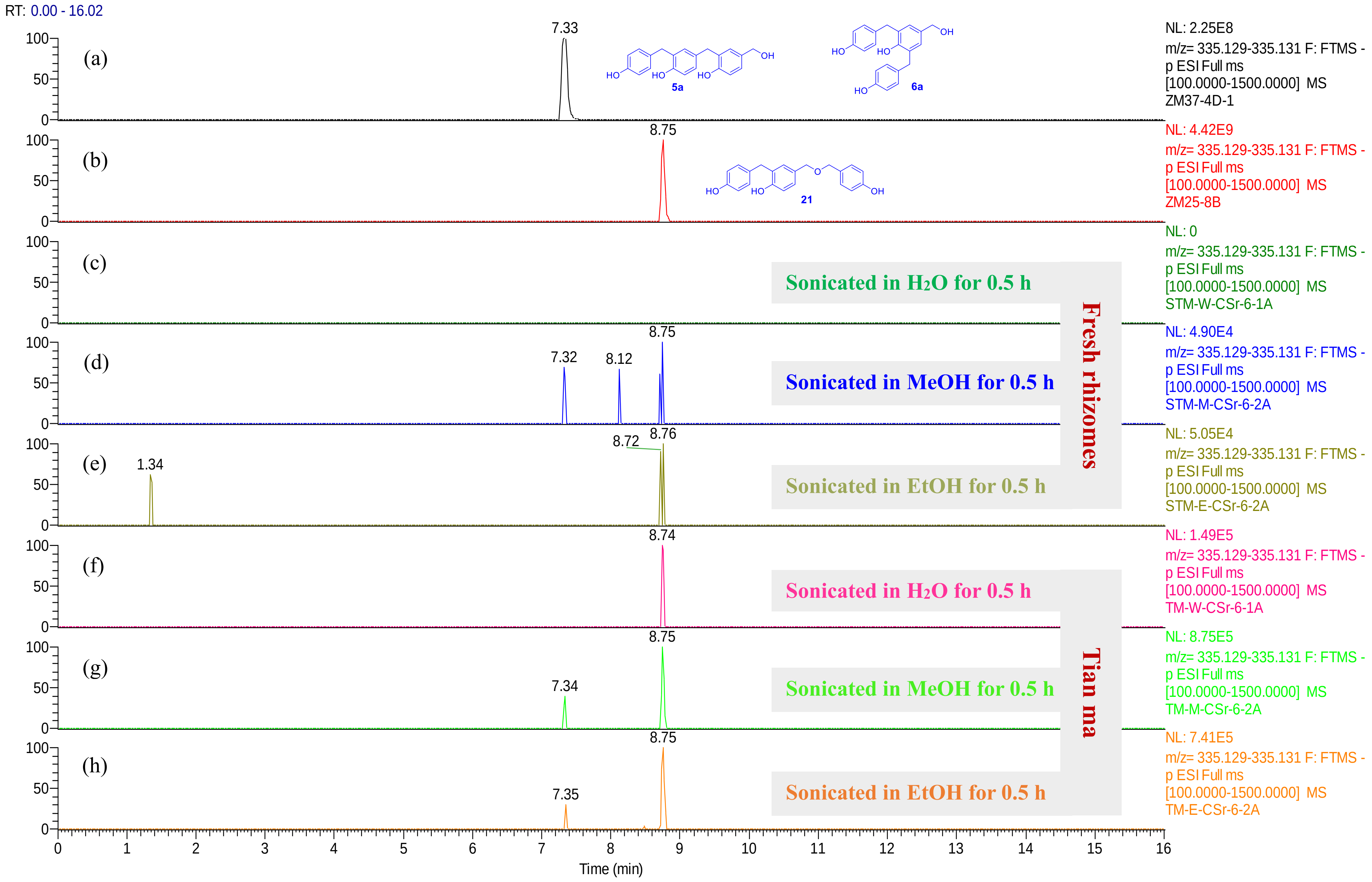

Fig. S234 Overlaid chromatograms of the extracted negative ion at *m/z* 335.130 $[M-H]^-$: (a) and (b) compounds **5a**/**6a** and **21** in CH_3CN, respectively; (c)–(e) extracts obtained by sonicating of fresh *G. elata* rhizomes with H_2O, MeOH, and EtOH, respectively; (f)–(h) extracts obtained by sonicating of "tian ma" (the steamed and dried *G. elata* rhizomes) with H_2O, MeOH, and EtOH, respectively.

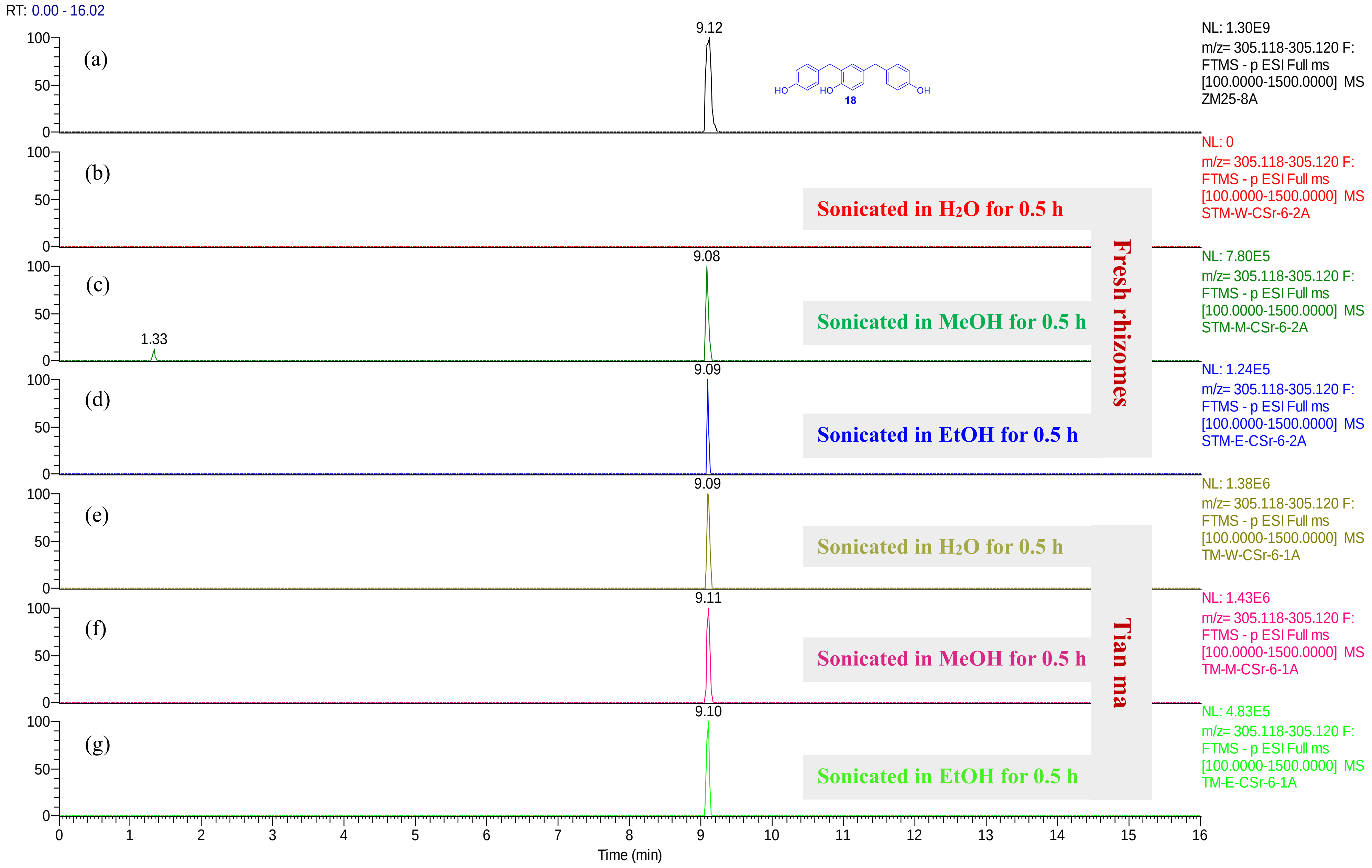

Fig. S235 Overlaid chromatograms of the extracted negative ion at *m/z* 305.119 $[M–H]^-$: (a) compound **18** in CH_3CN; (b)–(d) extracts obtained by sonicating of fresh *G. elata* rhizomes with H_2O, MeOH, and EtOH, respectively; (e)–(g) extracts obtained by sonicating of "tian ma" (the steamed and dried *G. elata* rhizomes) with H_2O, MeOH, and EtOH, respectively.

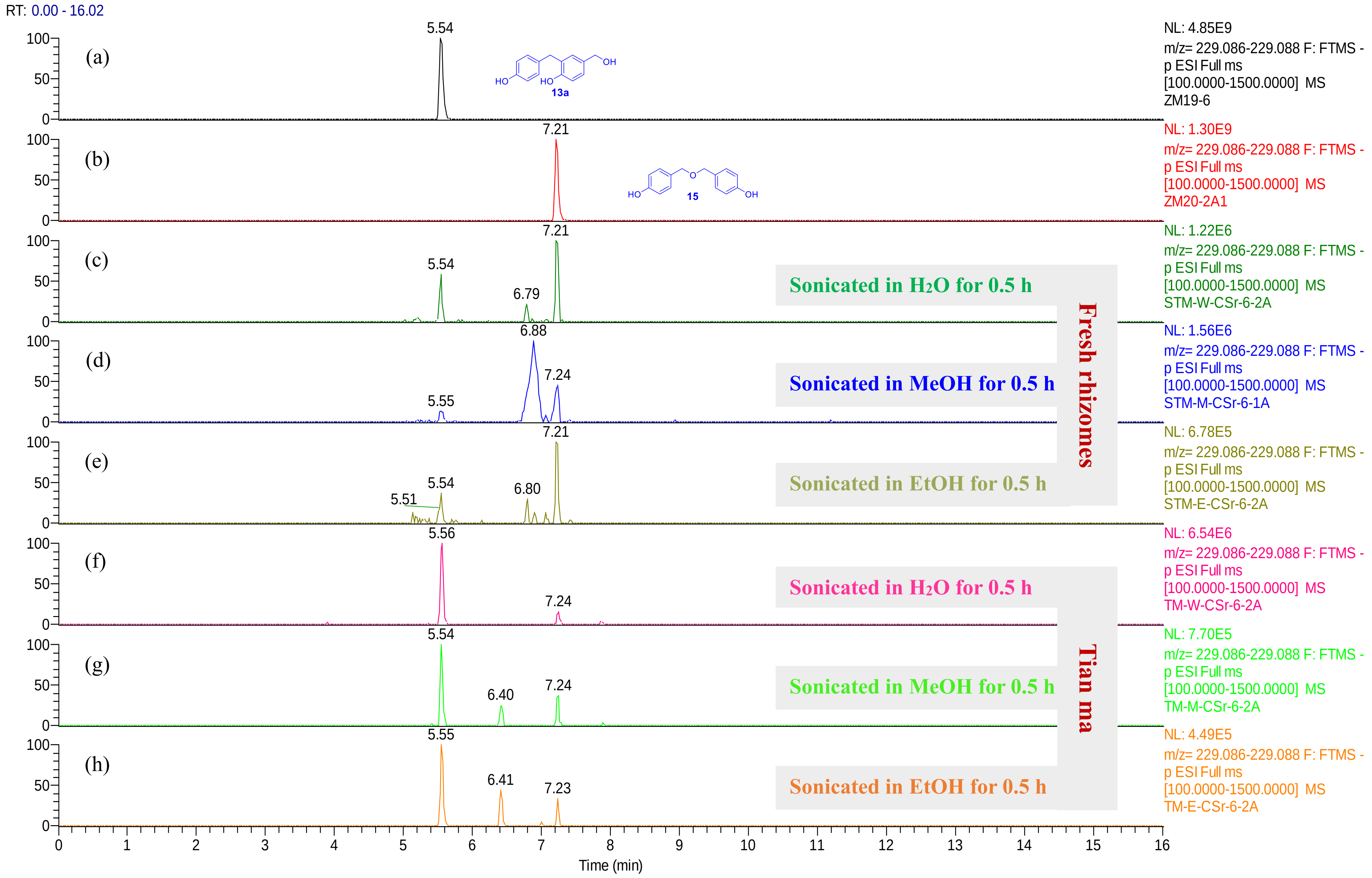

Fig. S236 Overlaid chromatograms of the extracted negative ion at *m/z* 229.087 $[M{-}H]^-$: (a) and (b) compounds **13a** and **15** in CH_3CN, respectively; (c)–(e) extracts obtained by sonicating of fresh *G. elata* rhizomes with H_2O, MeOH, and EtOH, respectively; (f)–(h) extracts obtained by sonicating of "tian ma" (the steamed and dried *G. elata* rhizomes) with H_2O, MeOH, and EtOH, respectively.

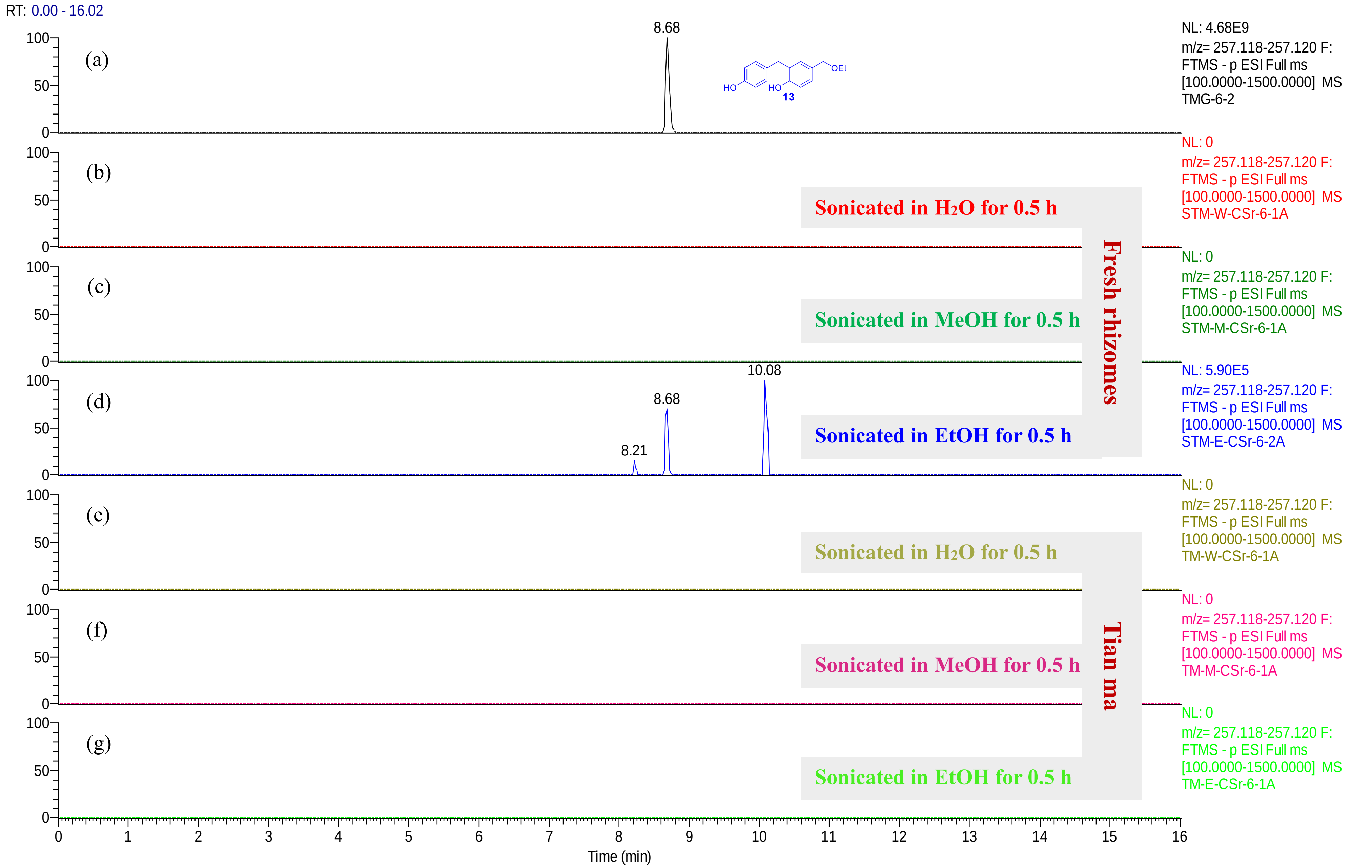

Fig. S237 Overlaid chromatograms of the extracted negative ion at *m/z* 257.119 $[M-H]^-$: (a) compound **13** in CH_3CN; (b)–(d) extracts obtained by sonicating of fresh *G. elata* rhizomes with H_2O, MeOH, and EtOH, respectively; (e)–(g) extracts obtained by sonicating of "tian ma" (the steamed and dried *G. elata* rhizomes) with H_2O, MeOH, and EtOH, respectively.

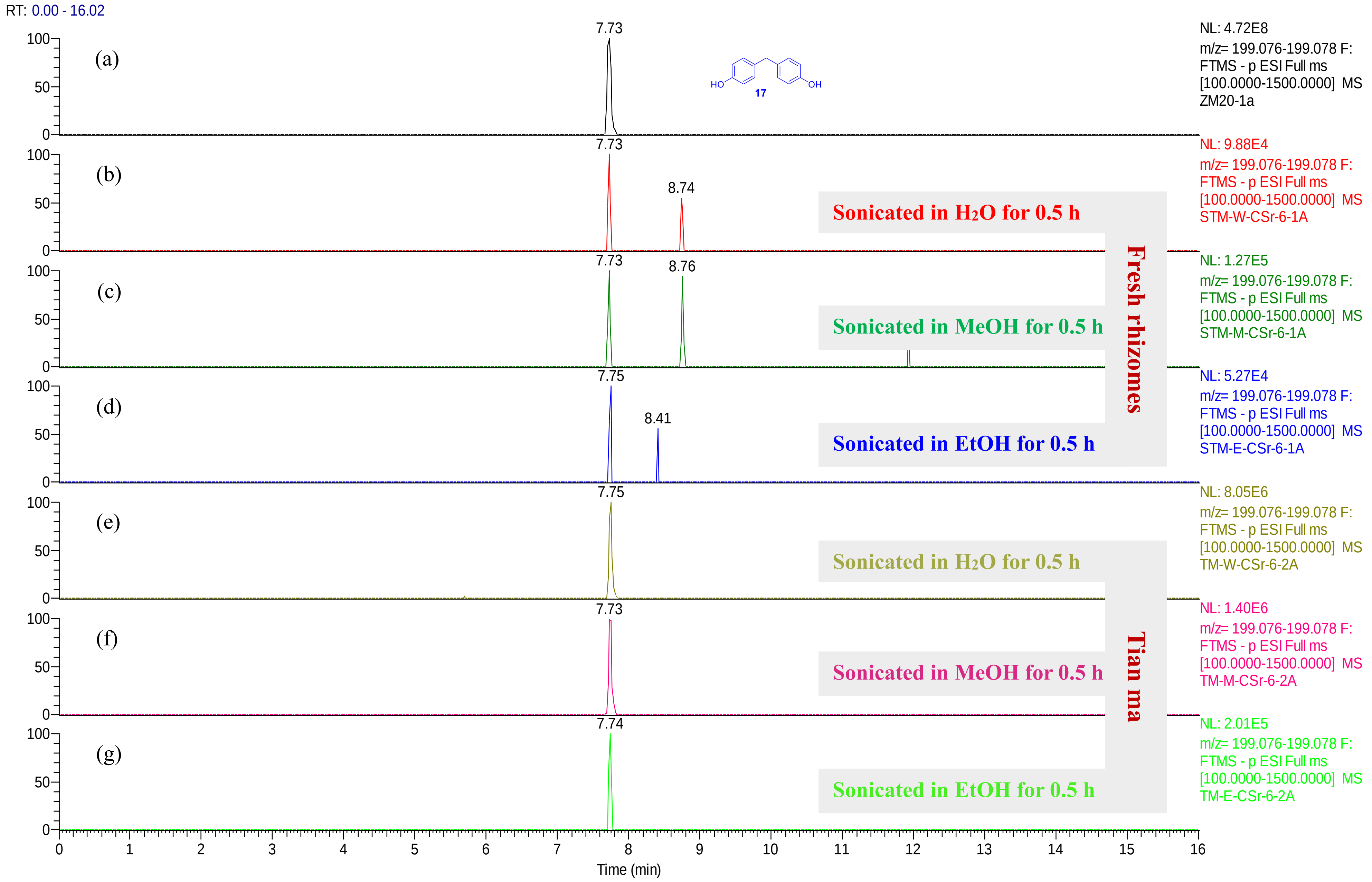

Fig. S238 Overlaid chromatograms of the extracted negative ion at *m/z* 199.077 $[M-H]^-$: (a) compound **17** in CH_3CN; (b)–(d) extracts obtained by sonicating of fresh *G. elata* rhizomes with H_2O, MeOH, and EtOH, respectively; (e)–(g) extracts obtained by sonicating of "tian ma" (the steamed and dried *G. elata* rhizomes) with H_2O, MeOH, and EtOH, respectively.

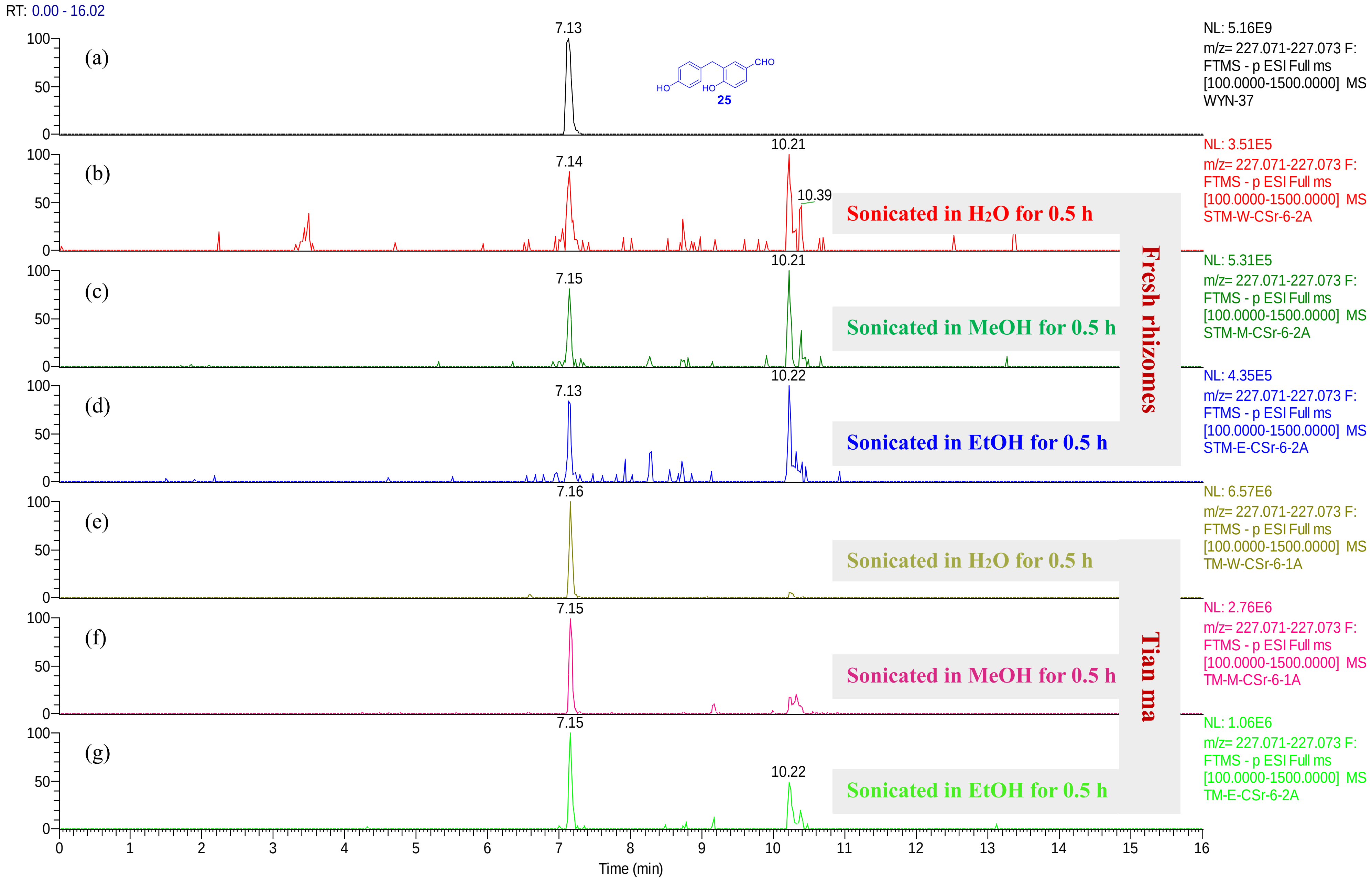

Fig. S239 Overlaid chromatograms of the extracted negative ion at *m/z* 227.072 $[M-H]^-$: (a) compound **25** in CH_3CN; (b)–(d) extracts obtained by sonicating of fresh *G. elata* rhizomes with H_2O, MeOH, and EtOH, respectively; (e)–(g) extracts obtained by sonicating of "tian ma" (the steamed and dried *G. elata* rhizomes) with H_2O, MeOH, and EtOH, respectively.

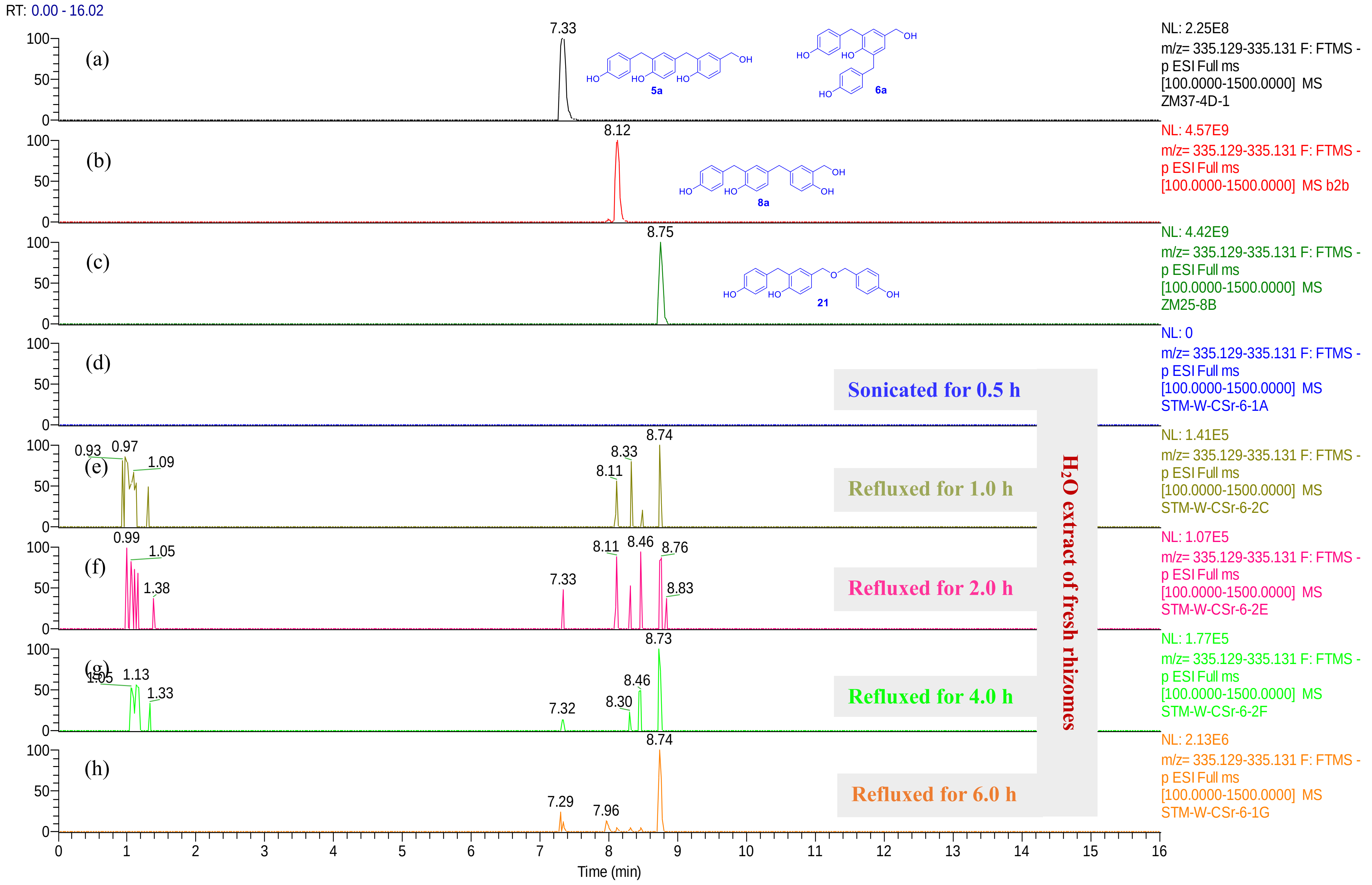

Fig. S240 Overlaid chromatograms of the extracted negative ion at *m/z* 335.130 [M−H]⁻: (a)–(c) compounds **5a**/**6a**, **8a**, and **21** in CH_3CN, respectively; (d)–(h) extracts obtained by sonicating of fresh *G. elata* rhizomes with H_2O for 0.5 h then refluxed for 1.0 h, 2. 0 h, 4.0 h, and 6.0 h, respectively.

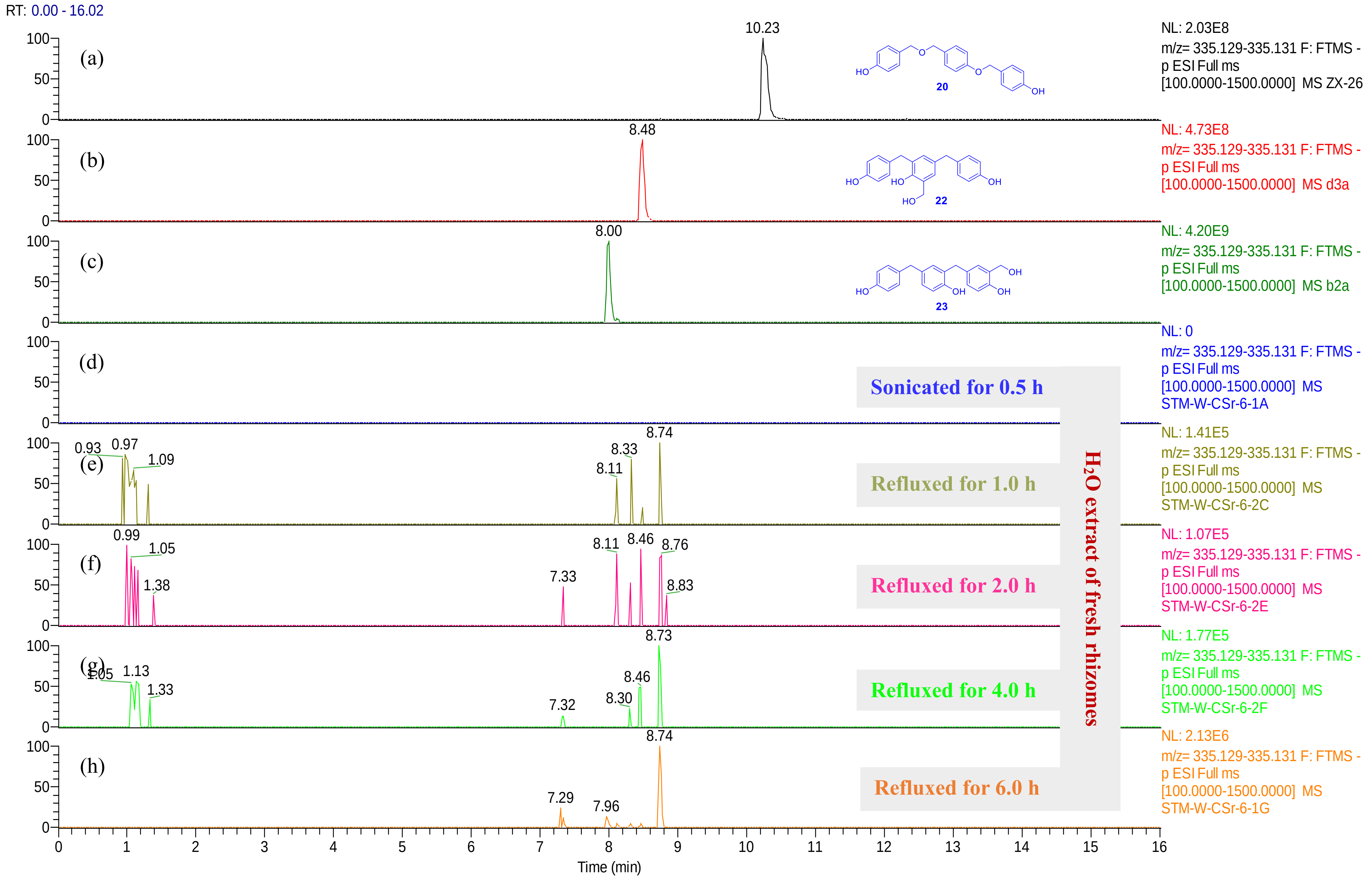

Fig. S241 Overlaid chromatograms of the extracted negative ion at *m/z* 335.130 [M–H]$^-$: (a)–(c) compounds **20**, **22**, and **23** in CH_3CN, respectively; (d)–(h) extracts obtained by sonicating of fresh *G. elata* rhizomes with H_2O for 0.5 h then refluxed for 1.0 h, 2.0 h, 4.0 h, and 6.0 h, respectively.

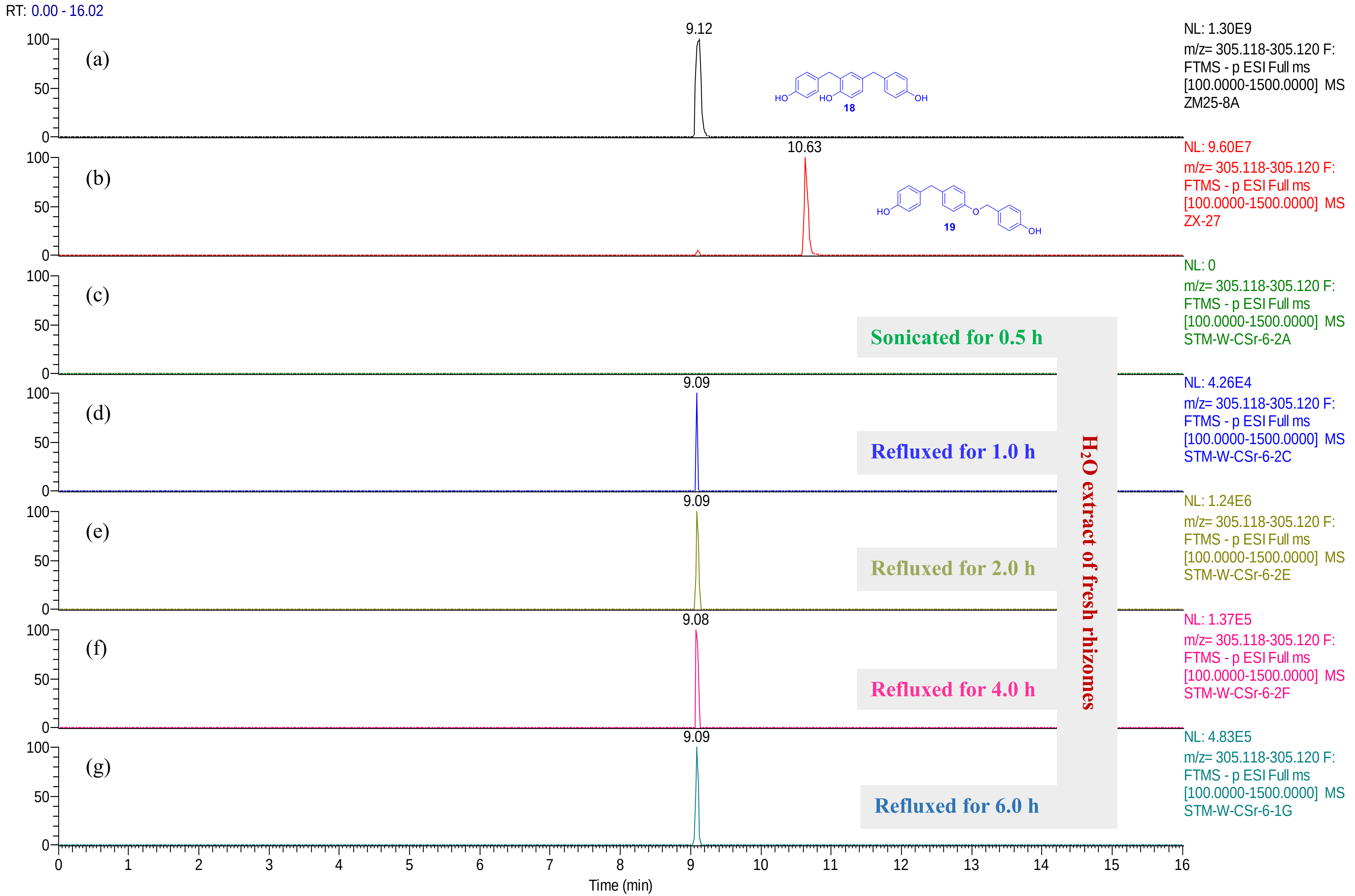

Fig. S242 Overlaid chromatograms of the extracted negative ion at *m/z* 305.119 $[M{-}H]^-$: (a) and (b) compounds **18** and **19** in CH_3CN, respectively; (c)–(g) extracts obtained by sonicating of fresh *G. elata* rhizomes with H_2O for 0.5 h then refluxed for 1.0 h, 2.0 h, 4.0 h, and 6.0 h, respectively.

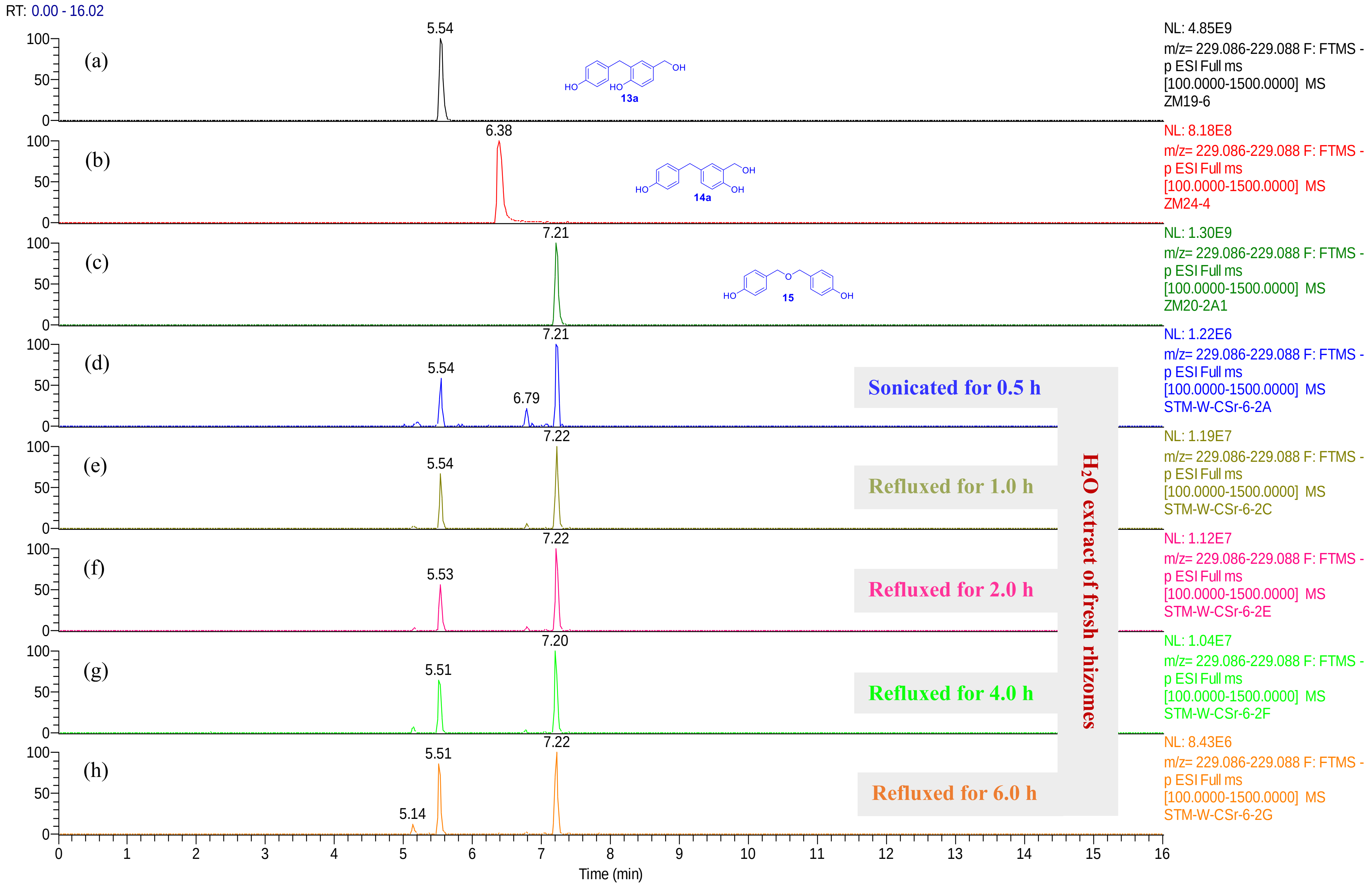

Fig. S243 Overlaid chromatograms of the extracted negative ion at *m/z* 229.087 $[M-H]^-$: (a)–(c) compounds **13a**, **14a**, and **15** in CH_3CN, respectively; (d)–(h) extracts obtained by sonicating of fresh *G. elata* rhizomes with H_2O for 0.5 h then refluxed for 1.0 h, 2.0 h, 4.0 h, and 6.0 h, respectively.

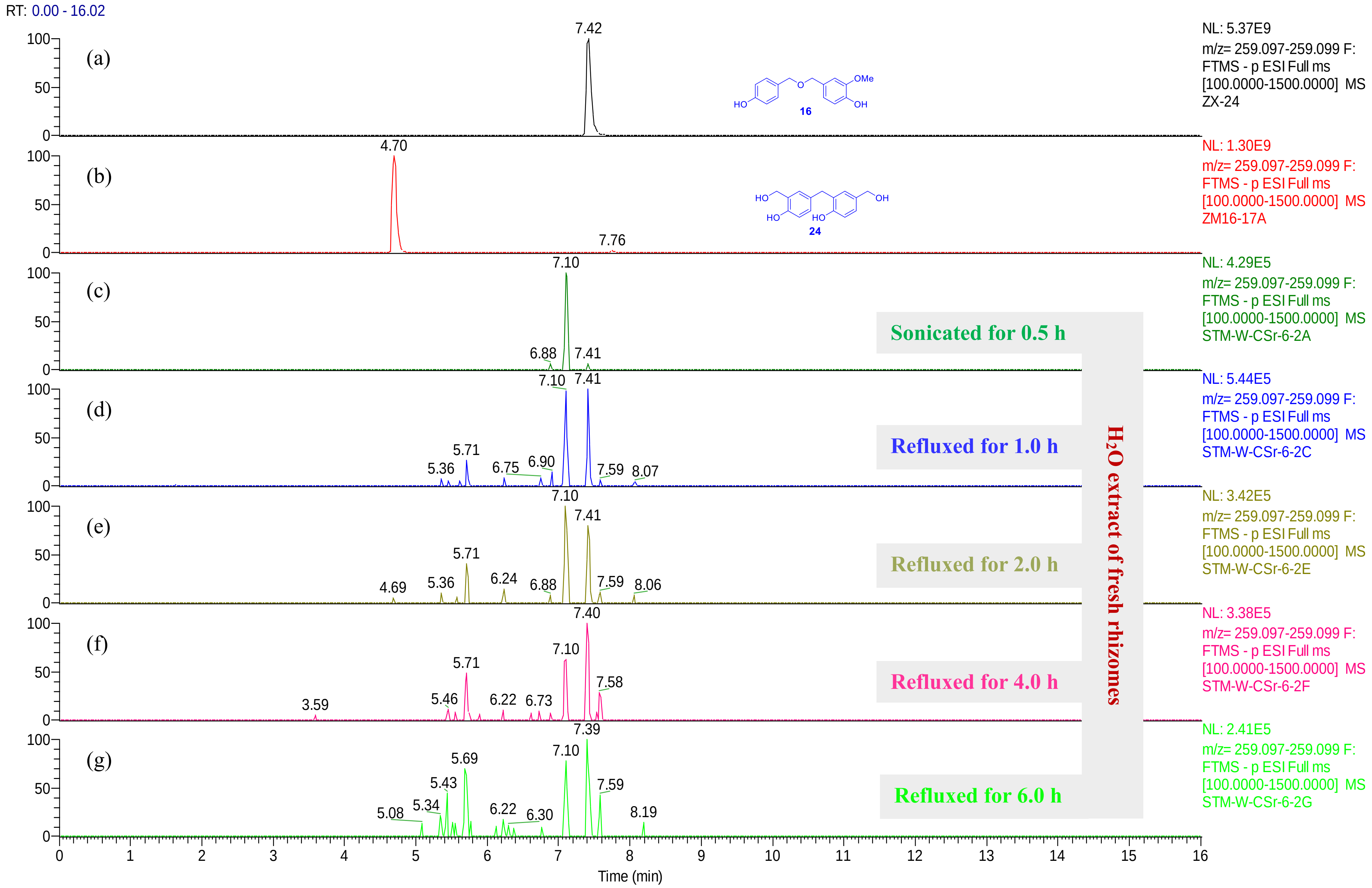

Fig. S244 Overlaid chromatograms of the extracted negative ion at *m/z* 259.098 $[M-H]^-$: (a) and (b) compounds **16** and **24** in CH_3CN, respectively; (c)–(g) extracts obtained by sonicating of fresh *G. elata* rhizomes with H_2O for 0.5 h then refluxed for 1.0 h, 2.0 h, 4.0 h, and 6.0 h, respectively.

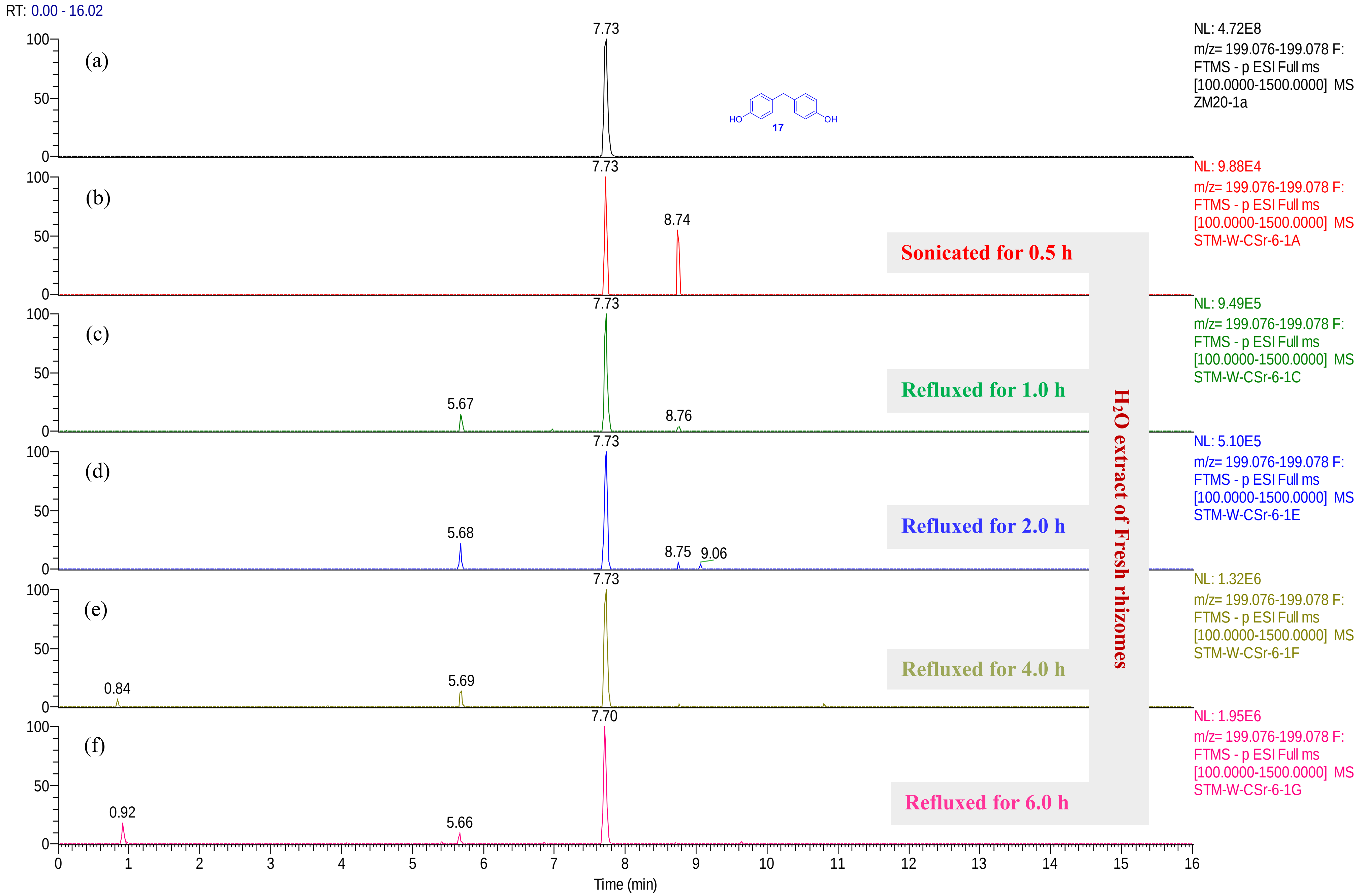

Fig. S245 Overlaid chromatograms of the extracted negative ion at *m/z* 199.077 [M–H]⁻: (a) compound **17** in CH_3CN; (b)–(f) extracts obtained by sonicating of fresh *G. elata* rhizomes with H_2O for 0.5 h then refluxed for 1.0 h, 2.0 h, 4.0 h, and 6.0 h, respectively.

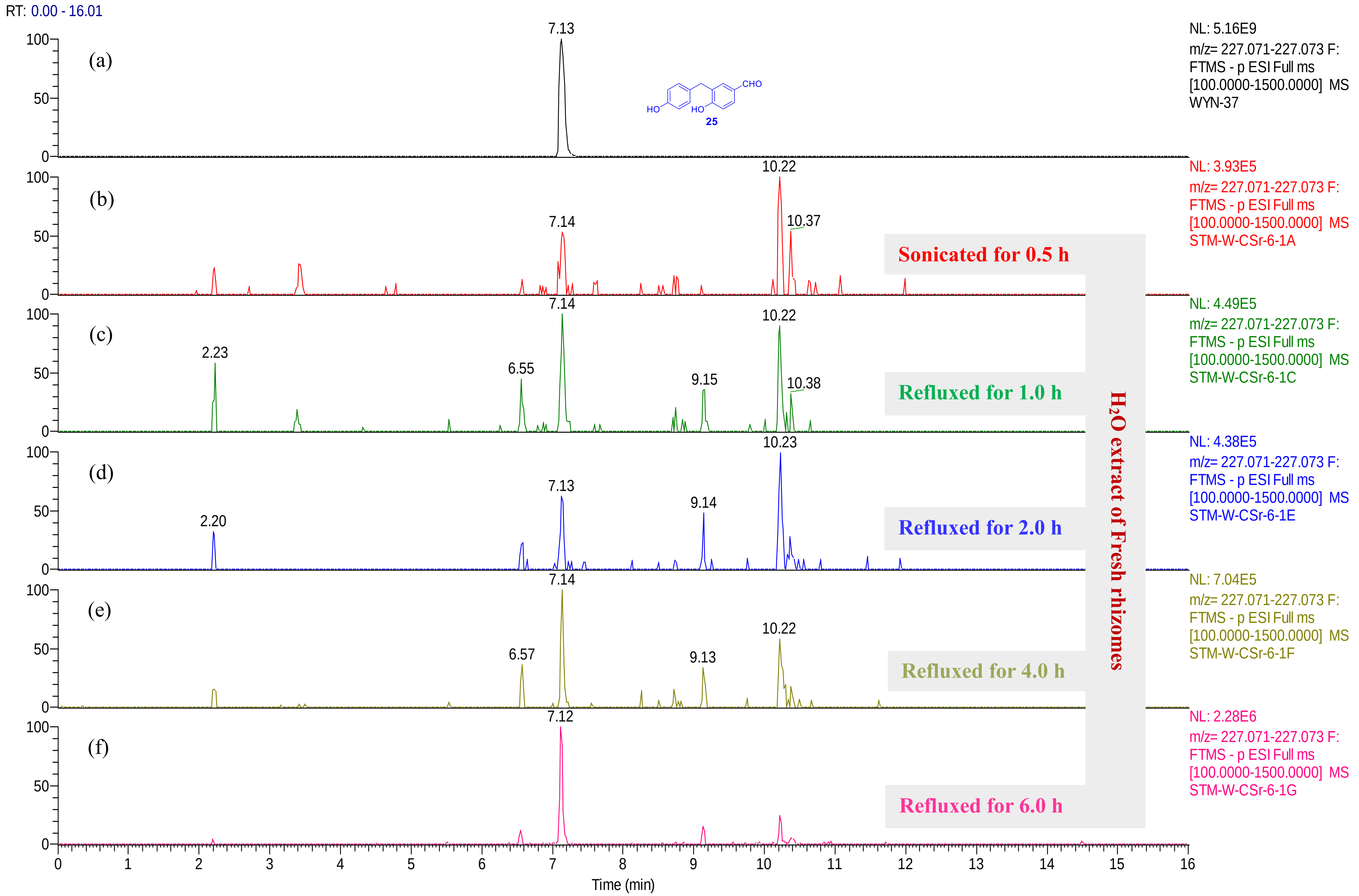

Fig. S246 Overlaid chromatograms of the extracted negative ion at *m/z* 227.072 [M–H]⁻: (a) compound **25** in CH_3CN; (b)–(f) extracts obtained by sonicating of fresh *G. elata* rhizomes with H_2O for 0.5 h then refluxed for 1.0 h, 2.0 h, 4.0 h, and 6.0 h, respectively.

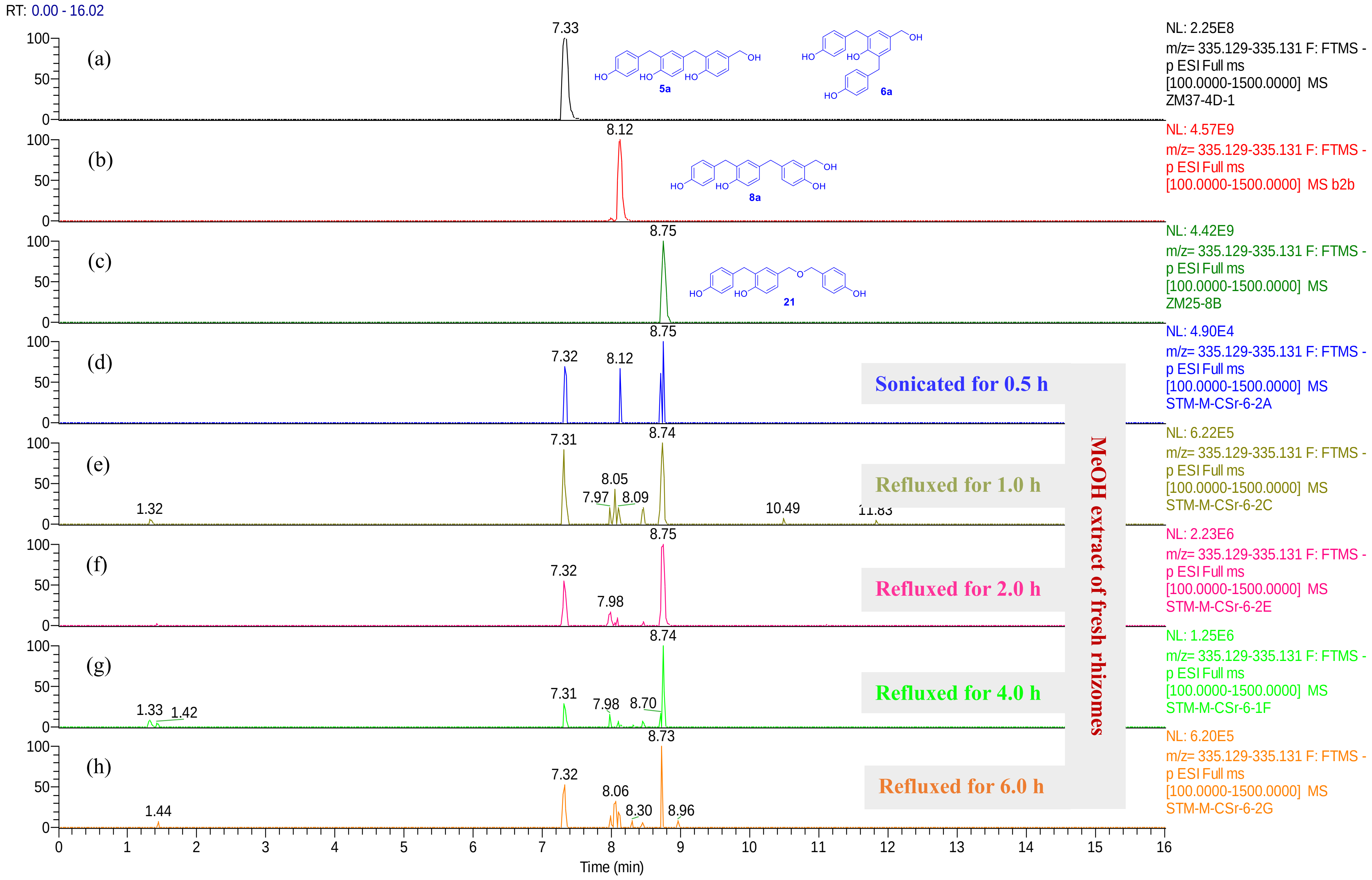

Fig. S247 Overlaid chromatograms of the extracted negative ion at *m/z* 335.130 $[M-H]^-$: (a)–(c) compounds **5a**/**6a**, **8a**, and **21** in CH_3CN, respectively; (d)–(h) extracts obtained by sonicating of fresh *G. elata* rhizomes with MeOH for 0.5 h then refluxed for 1.0 h, 2.0 h, 4.0 h, and 6.0 h, respectively.

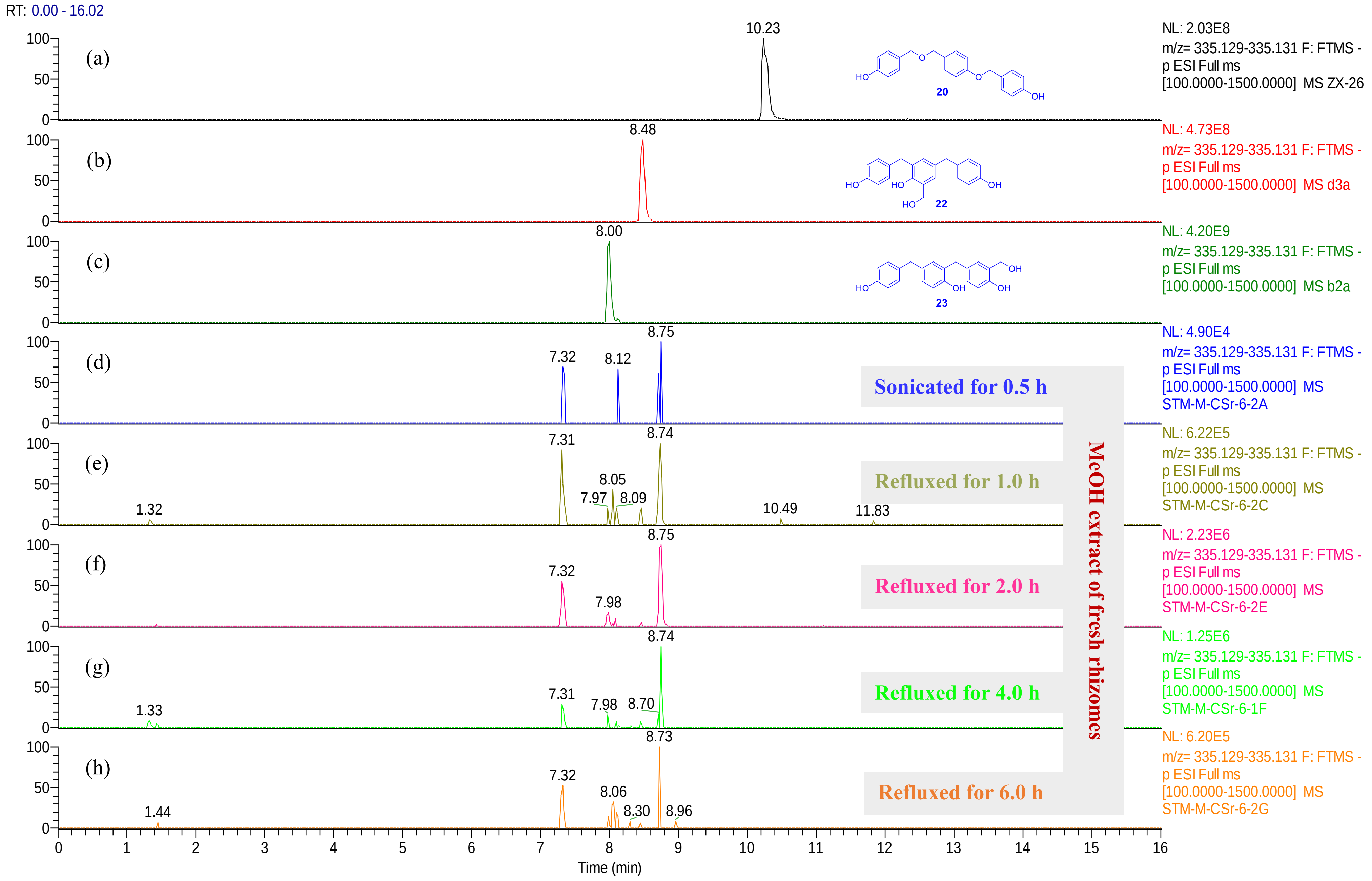

Fig. S248 Overlaid chromatograms of the extracted negative ion at *m/z* 335.130 $[M-H]^-$: (a)–(c) compounds **20**, **22**, and **23** in CH_3CN, respectively; (d)–(h) extracts obtained by sonicating of fresh *G. elata* rhizomes with MeOH for 0.5 h then refluxed for 1.0 h, 2.0 h, 4.0 h, and 6.0 h, respectively.

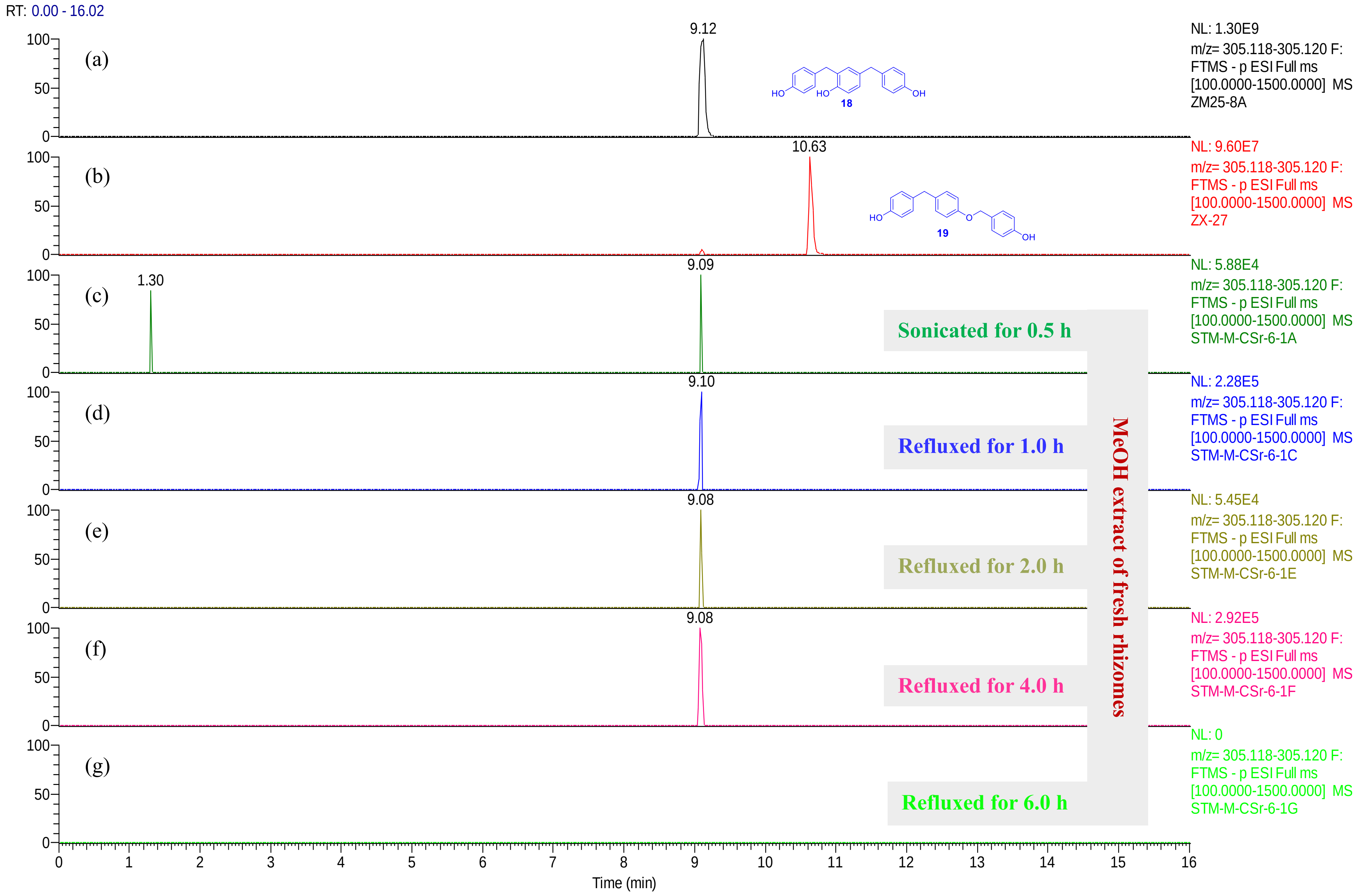

Fig. S249 Overlaid chromatograms of the extracted negative ion at *m/z* 305.119 [M–H]$^-$: (a) and (b) compounds **18** and **19** in CH_3CN, respectively; (c)–(g) extracts obtained by sonicating of fresh *G. elata* rhizomes with MeOH for 0.5 h then refluxed for 1.0 h, 2.0 h, 4.0 h, and 6.0 h, respectively.

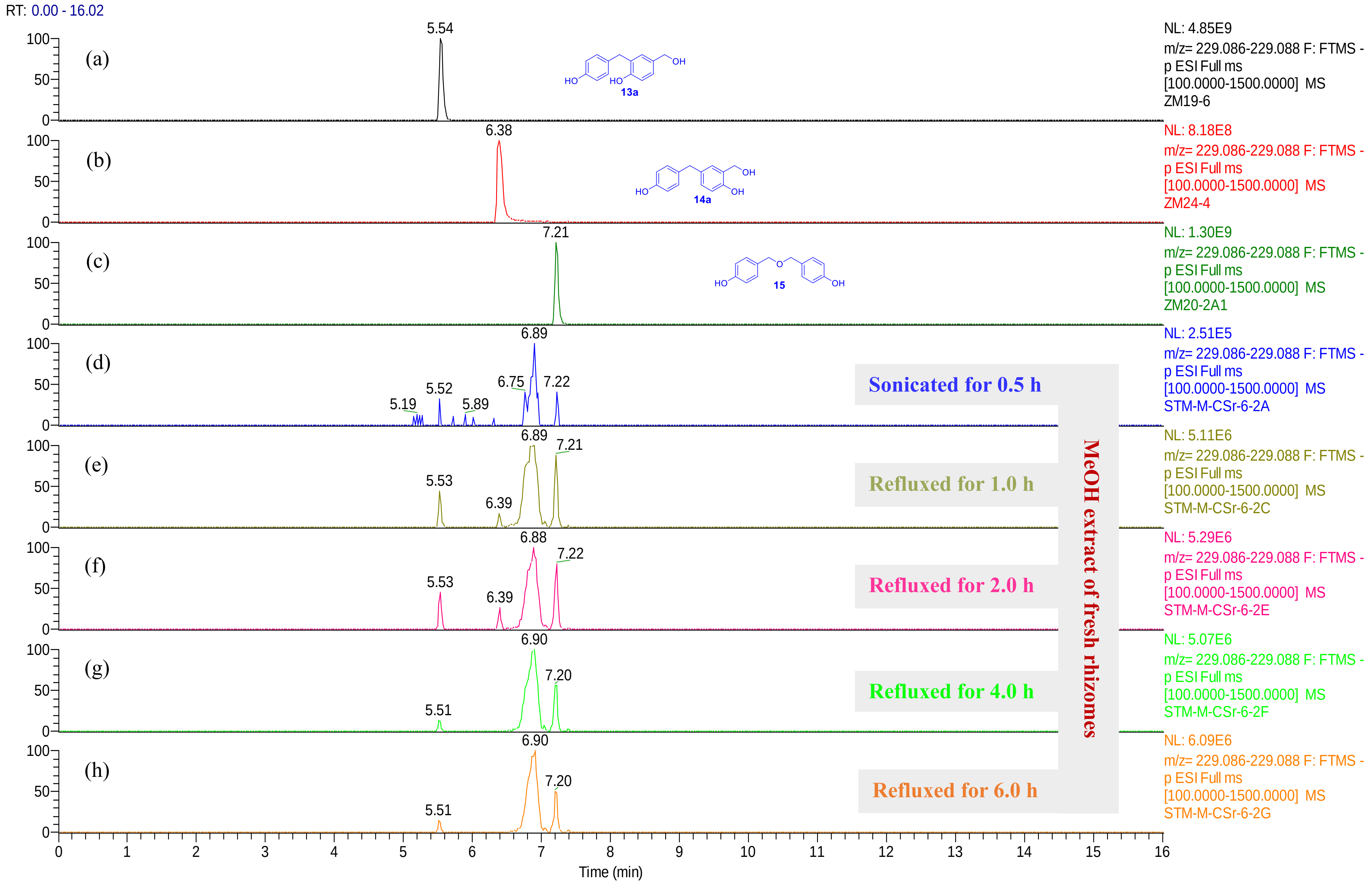

Fig. S250 Overlaid chromatograms of the extracted negative ion at *m/z* 229.087 $[M-H]^-$: (a)–(c) compounds **13a**, **14a**, and **15** in CH_3CN, respectively; (d)–(h) extracts obtained by sonicating of fresh *G. elata* rhizomes with MeOH for 0.5 h then refluxed for 1.0 h, 2.0 h, 4.0 h, and 6.0 h, respectively.

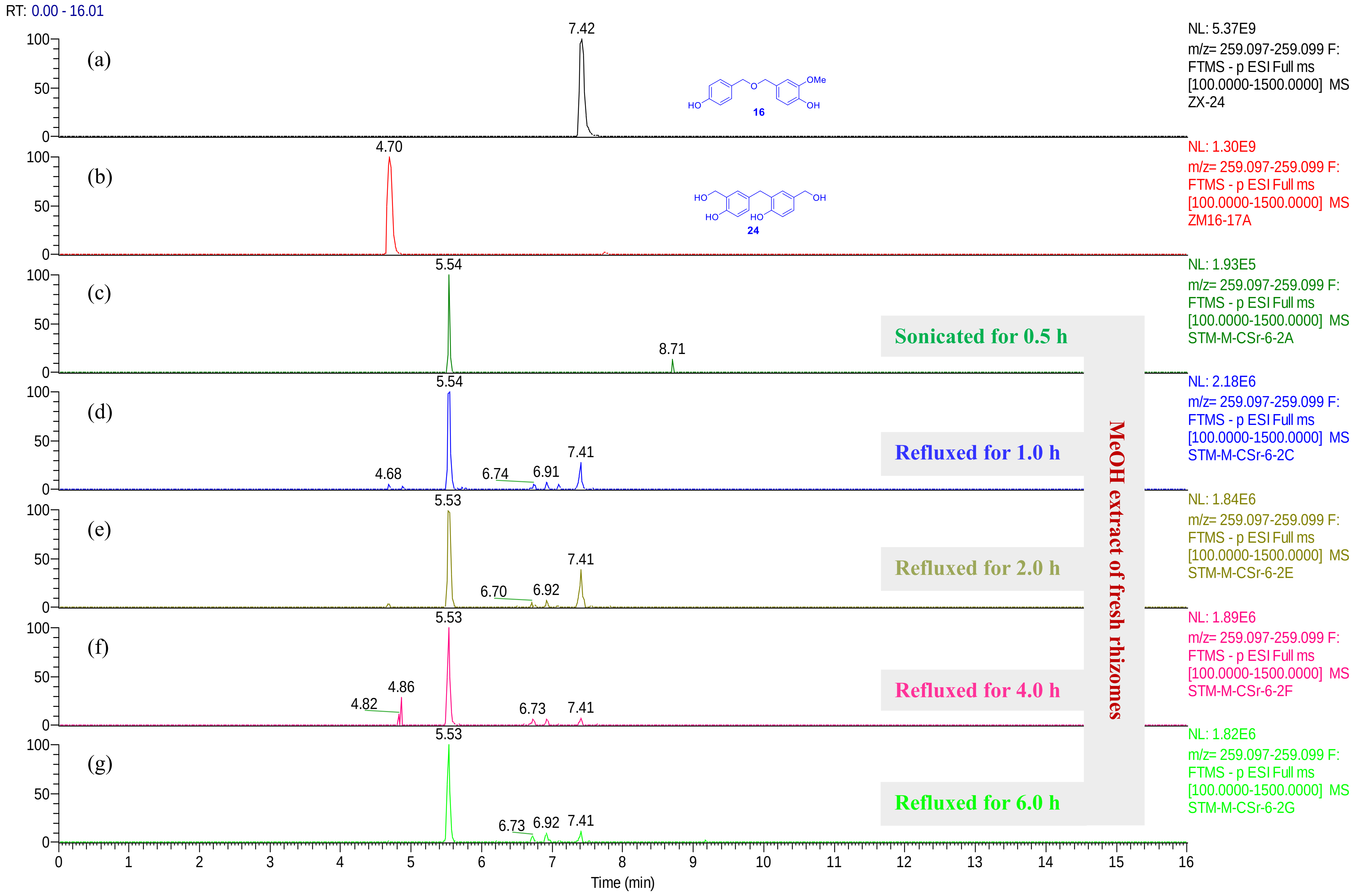

Fig. S251 Overlaid chromatograms of the extracted negative ion at *m/z* 259.098 [M–H]⁻: (a) and (b) compounds **16** and **24** in CH_3CN, respectively; (c)–(g) extracts obtained by sonicating of fresh *G. elata* rhizomes with MeOH for 0.5 h then refluxed for 1.0 h, 2.0 h, 4.0 h, and 6.0 h, respectively.

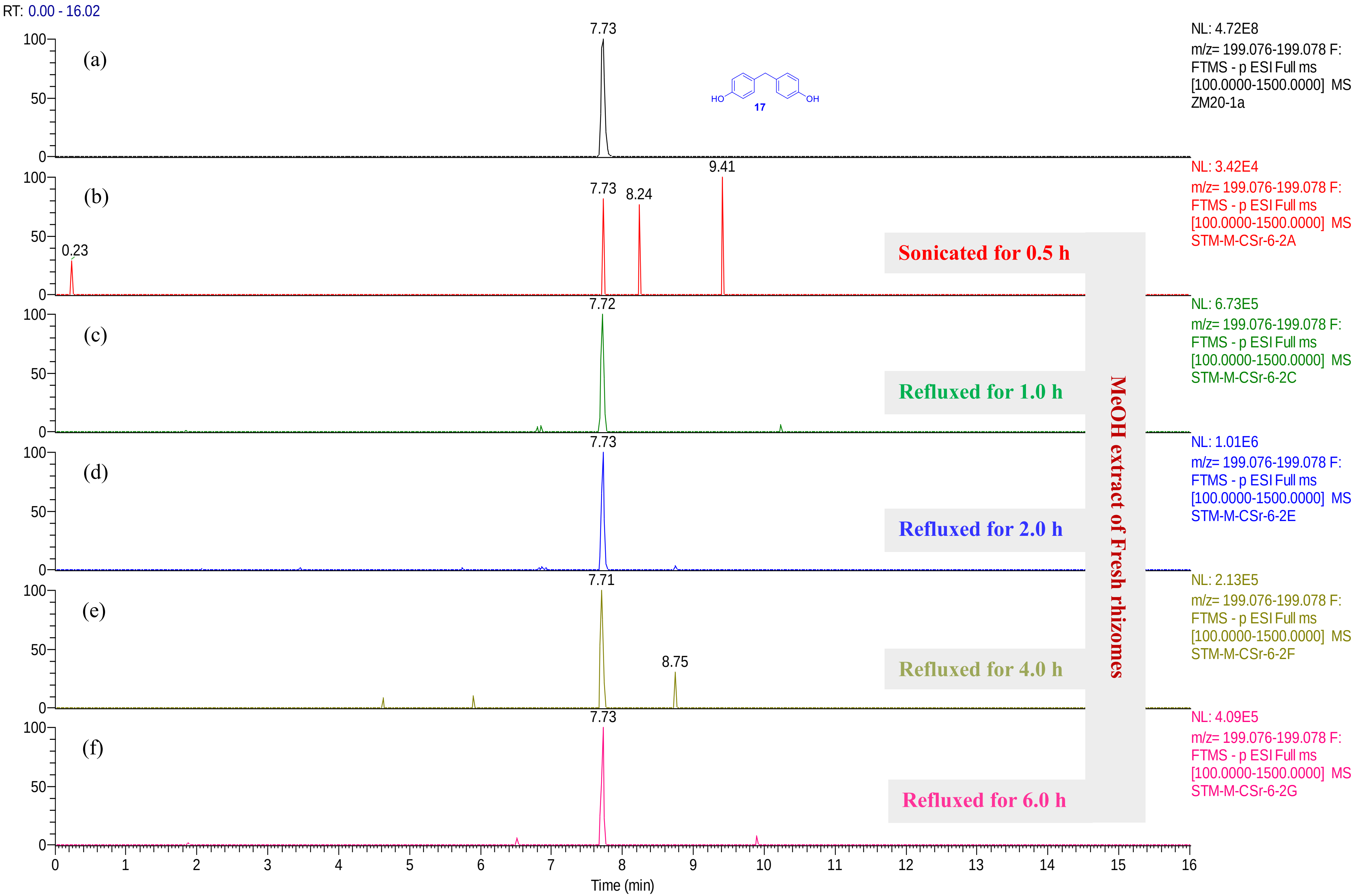

Fig. S252 Overlaid chromatograms of the extracted negative ion at *m/z* 199.077 [M−H]⁻: (a) compound **17** in CH_3CN; (b)–(f) extracts obtained by sonicating of fresh *G. elata* rhizomes with MeOH for 0.5 h then refluxed for 1.0 h, 2.0 h, 4.0 h, and 6.0 h, respectively.

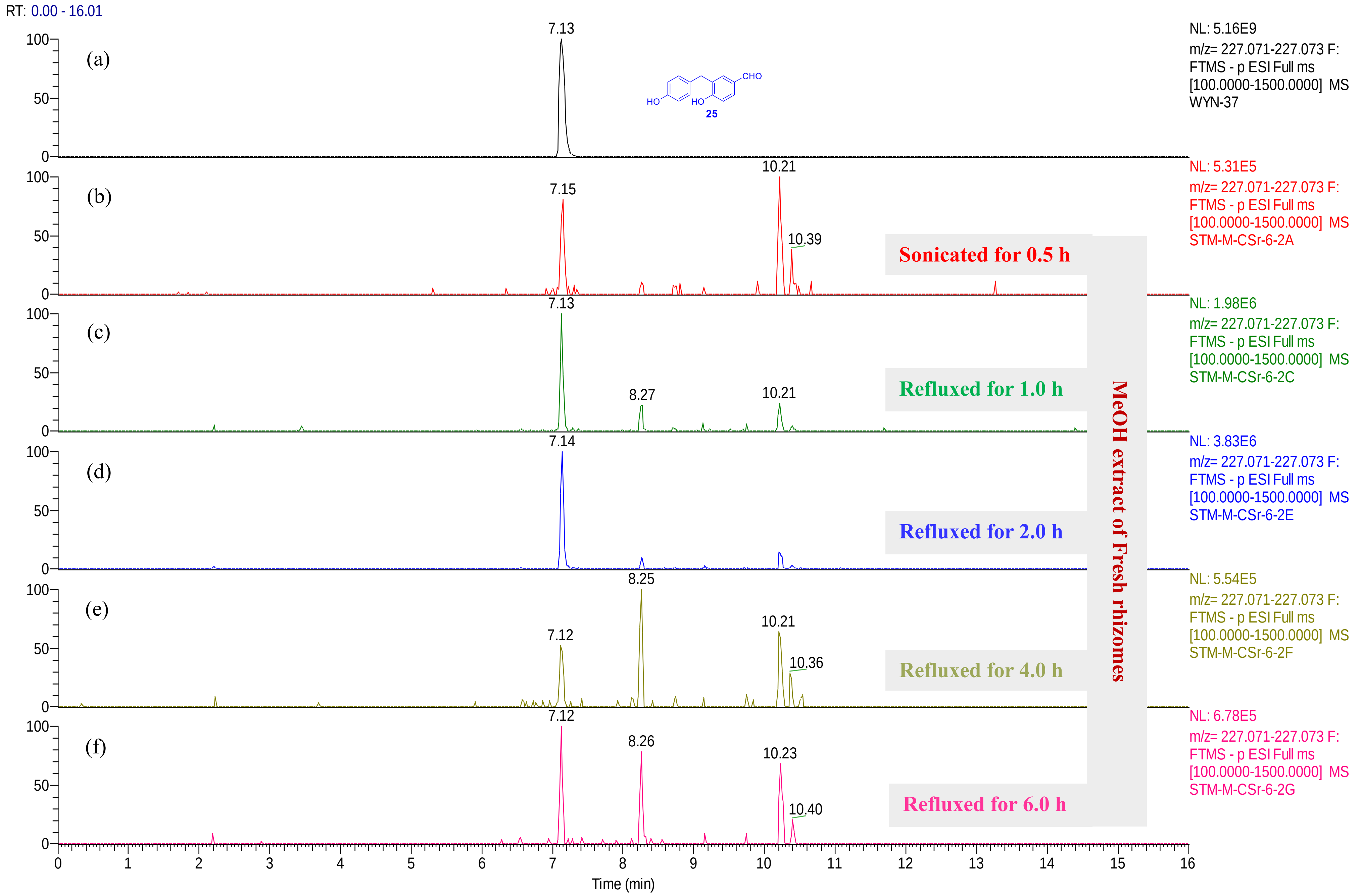

Fig. S253 Overlaid chromatograms of the extracted negative ion at *m/z* 227.072 [M−H]⁻: (a) compound **25** in CH_3CN; (b)–(f) extracts obtained by sonicating of fresh *G. elata* rhizomes with MeOH for 0.5 h then refluxed for 1.0 h, 2.0 h, 4.0 h, and 6.0 h, respectively.

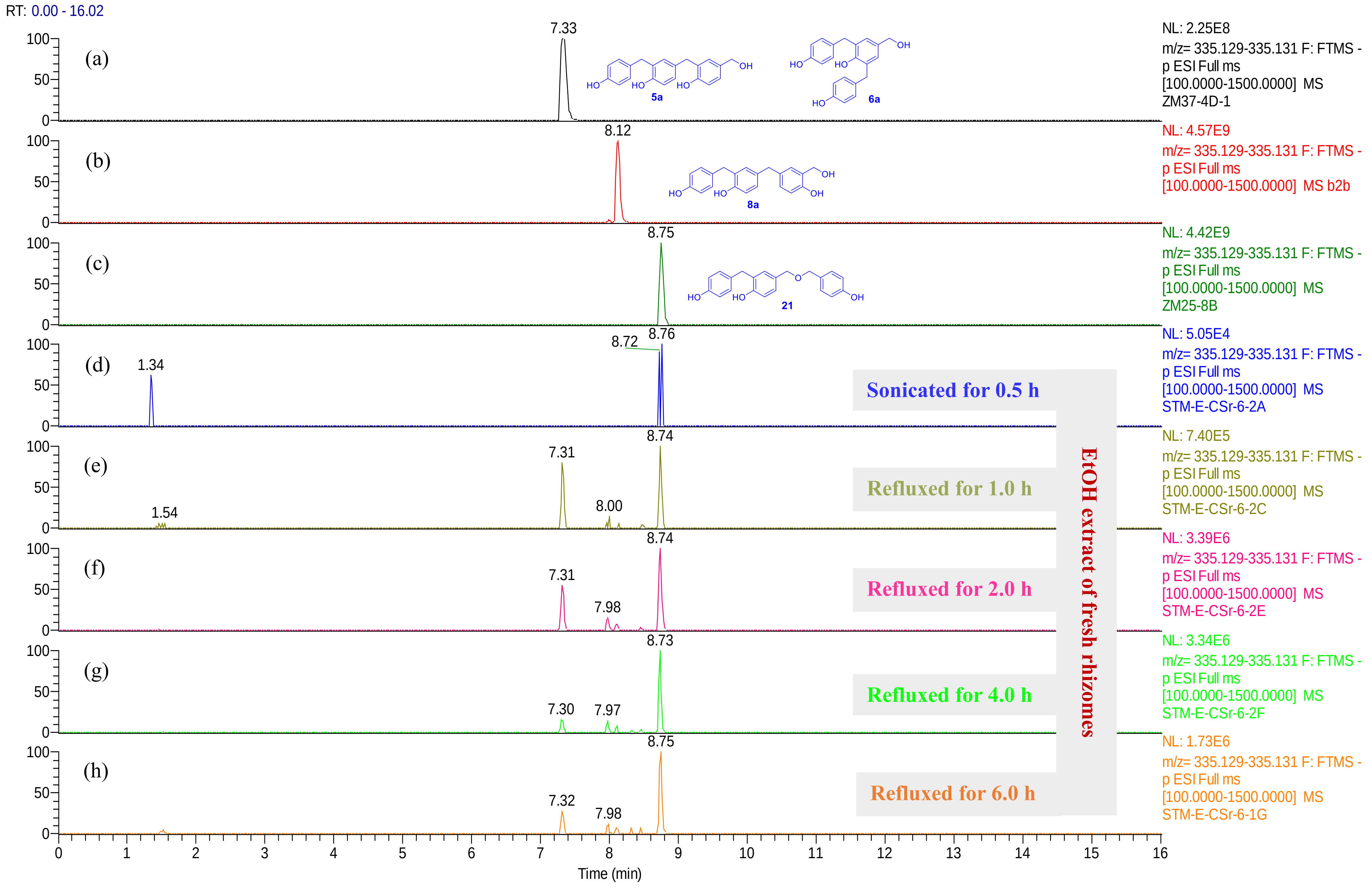

Fig. S254 Overlaid chromatograms of the extracted negative ion at *m/z* 335.130 $[M-H]^-$: (a)–(c) compounds **5a**/**6a**, **8a**, and **21** in CH_3CN, respectively; (d)–(h) extracts obtained by sonicating of fresh *G. elata* rhizomes with EtOH for 0.5 h then refluxed for 1.0 h, 2.0 h, 4.0 h, and 6.0 h, respectively.

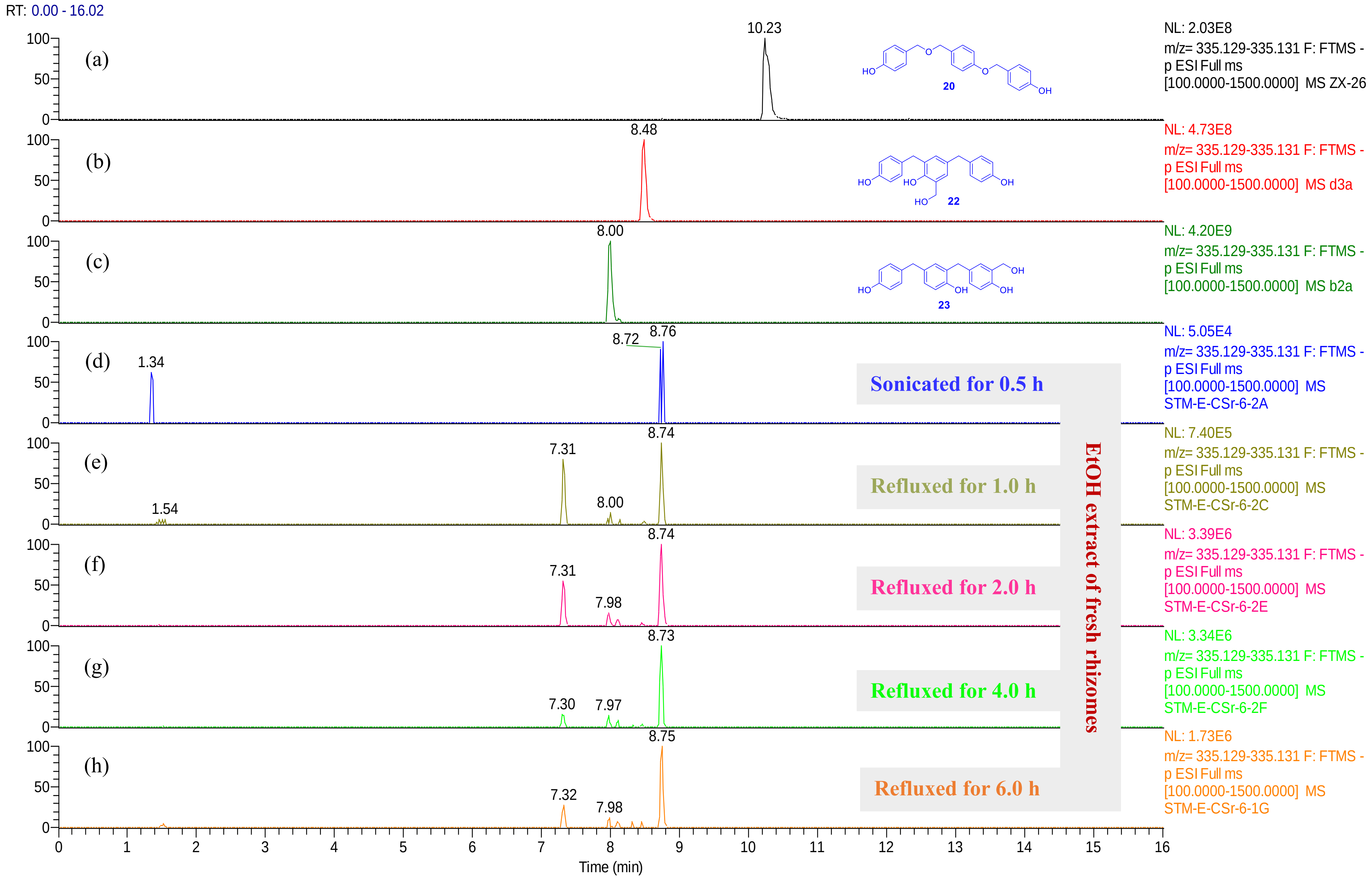

Fig. S255 Overlaid chromatograms of the extracted negative ion at *m/z* 335.130 $[M–H]^-$: (a)–(c) compounds **20**, **22**, and **23** in CH_3CN, respectively; (d)–(h) extracts obtained by sonicating of fresh *G. elata* rhizomes with EtOH for 0.5 h then refluxed for 1.0 h, 2.0 h, 4.0 h, and 6.0 h, respectively.

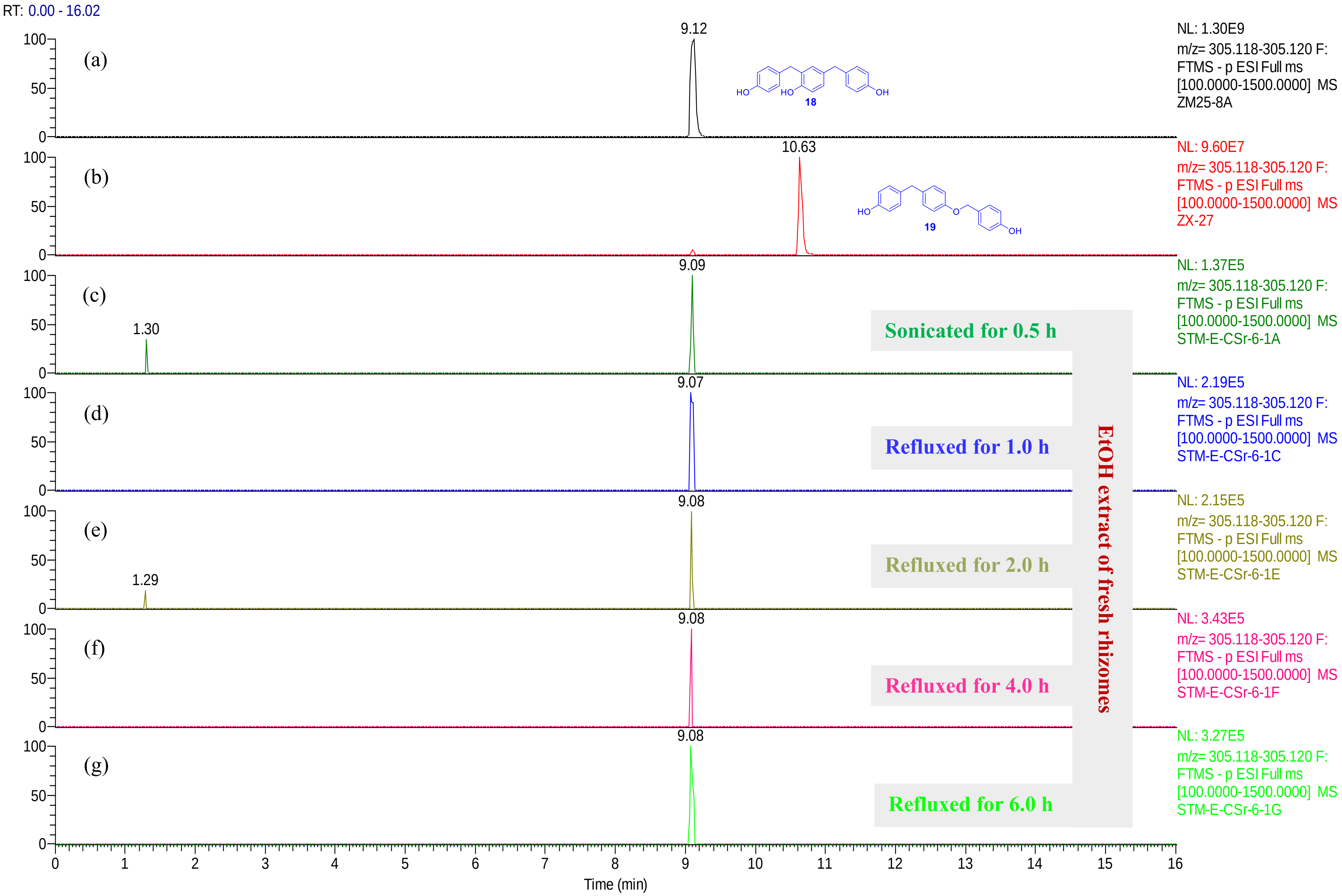

Fig. S256 Overlaid chromatograms of the extracted negative ion at *m/z* 305.119 $[M-H]^-$: (a) and (b) compounds **18** and **19** in CH_3CN, respectively; (c)–(g) extracts obtained by sonicating of fresh *G. elata* rhizomes with EtOH for 0.5 h then refluxed for 1.0 h, 2.0 h, 4.0 h, and 6.0 h, respectively.

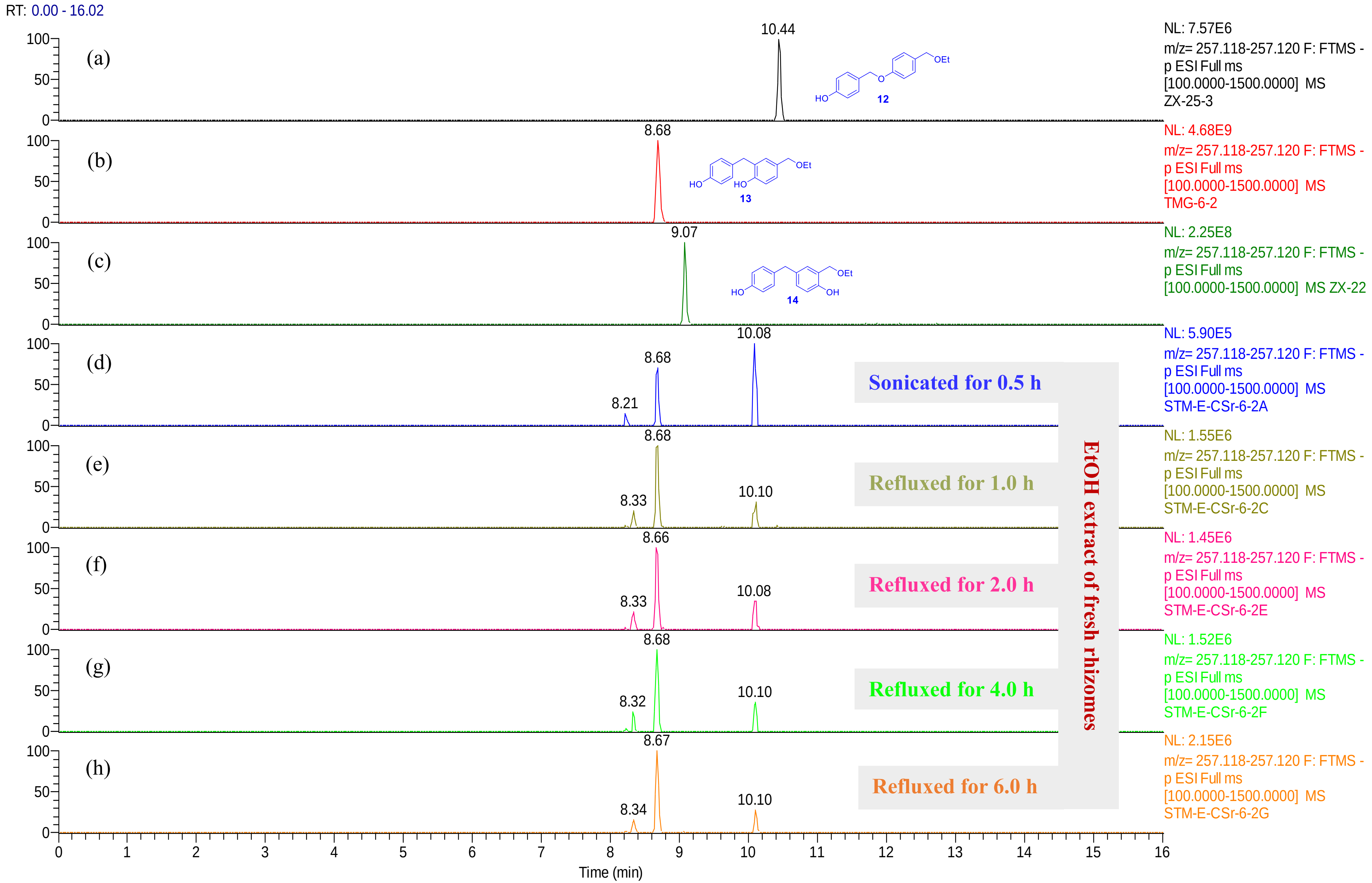

Fig. S257 Overlaid chromatograms of the extracted negative ion at *m/z* 257.119 [M−H]⁻: (a)−(c) compounds **12**, **13**, and **14** in CH_3CN, respectively; (d)−(h) extracts obtained by sonicating of fresh *G. elata* rhizomes with EtOH for 0.5 h then refluxed for 1.0 h, 2.0 h, 4.0 h, and 6.0 h, respectively.

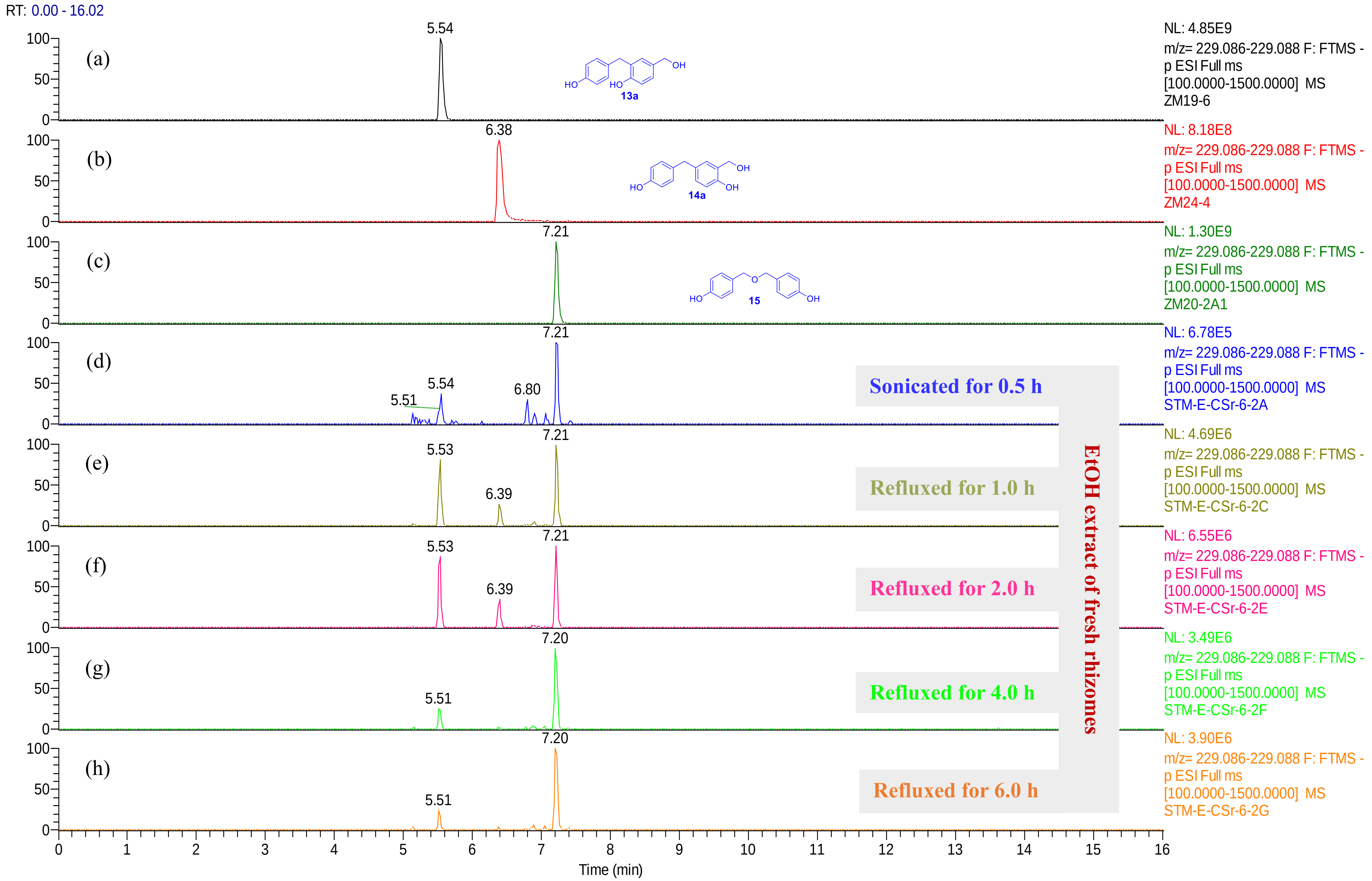

Fig. S258 Overlaid chromatograms of the extracted negative ion at *m/z* 229.087 $[M-H]^-$: (a)–(c) compounds **13a**, **14a**, and **15** in CH_3CN, respectively; (d)–(h) extracts obtained by sonicating of fresh *G. elata* rhizomes with EtOH for 0.5 h then refluxed for 1.0 h, 2.0 h, 4.0 h, and 6.0 h, respectively.

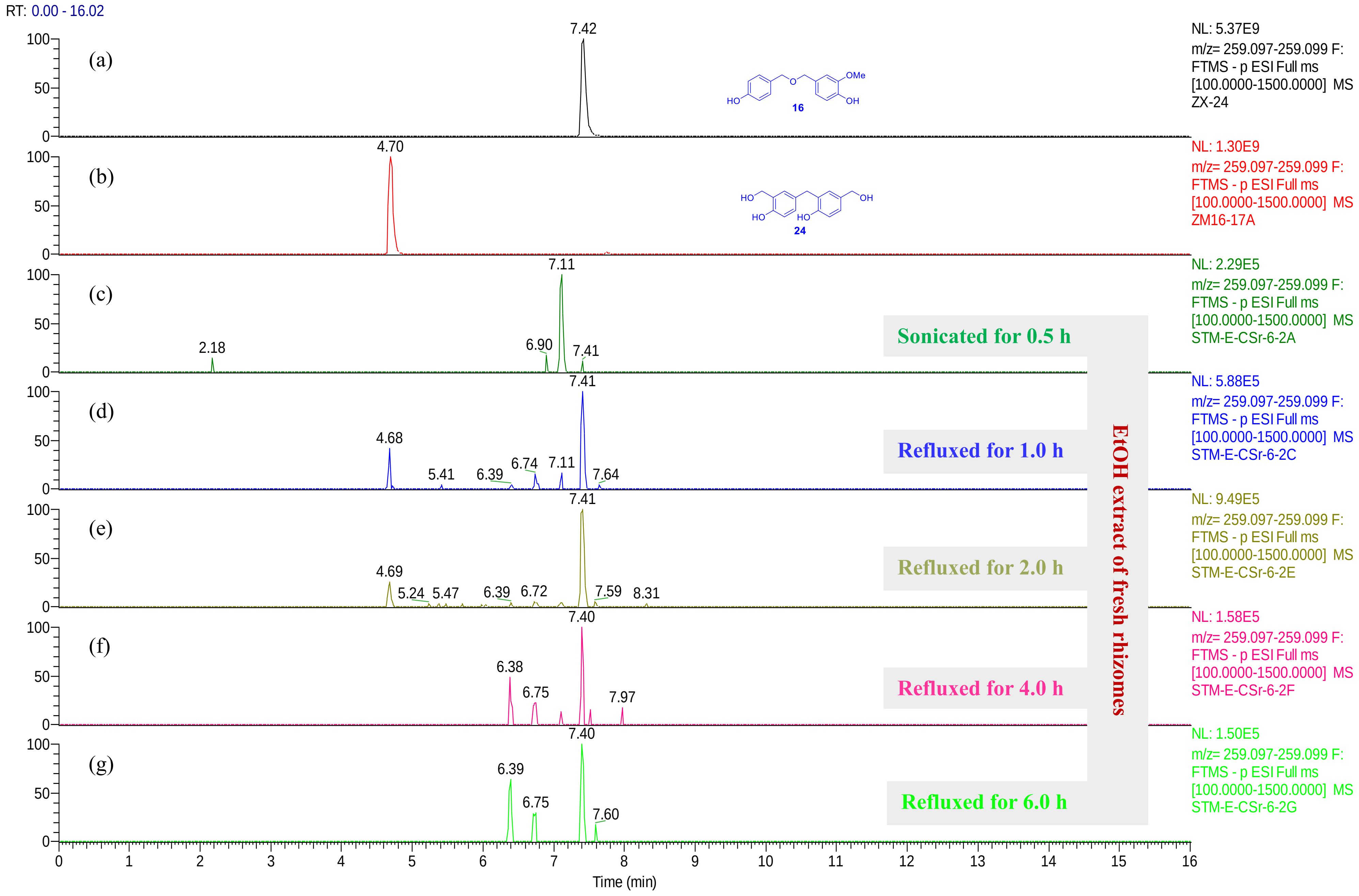

Fig. S259 Overlaid chromatograms of the extracted negative ion at *m/z* 259.098 [M–H]⁻: (a) and (b) compounds **16** and **24** in CH_3CN, respectively; (c)–(g) extracts obtained by sonicating of fresh *G. elata* rhizomes with EtOH for 0.5 h then refluxed for 1.0 h, 2.0 h, 4.0 h, and 6.0 h, respectively.

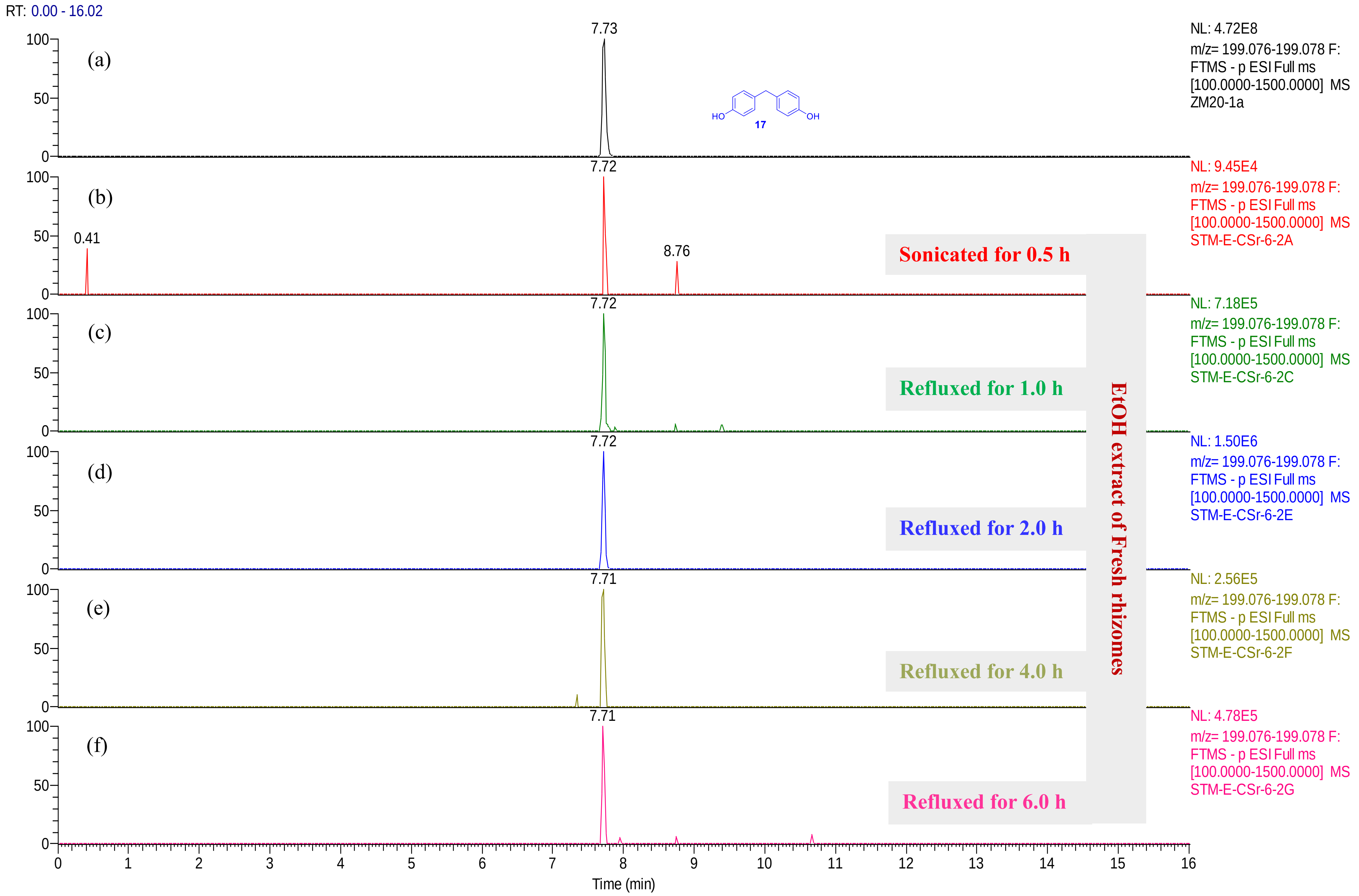

Fig. S260 Overlaid chromatograms of the extracted negative ion at *m/z* 199.077 $[M-H]^-$: (a) compound **17** in CH_3CN; (b)–(f) extracts obtained by sonicating of fresh *G. elata* rhizomes with EtOH for 0.5 h then refluxed for 1.0 h, 2.0 h, 4.0 h, and 6.0 h, respectively.

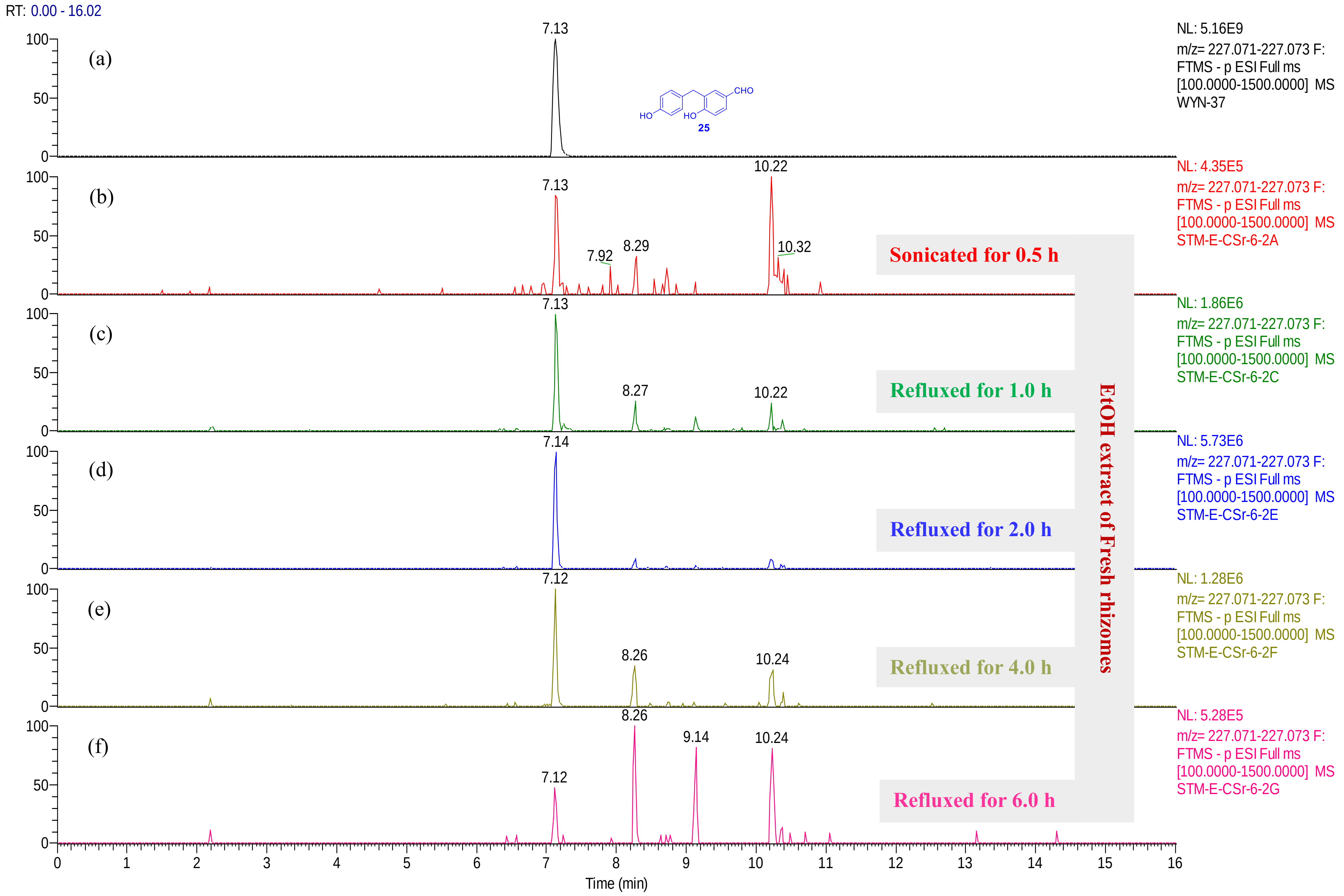

Fig. S261 Overlaid chromatograms of the extracted negative ion at *m/z* 227.072 [M–H]⁻: (a) compound **25** in CH_3CN; (b)–(f) extracts obtained by sonicating of fresh *G. elata* rhizomes with EtOH for 0.5 h then refluxed for 1.0 h, 2.0 h, 4.0 h, and 6.0 h, respectively.

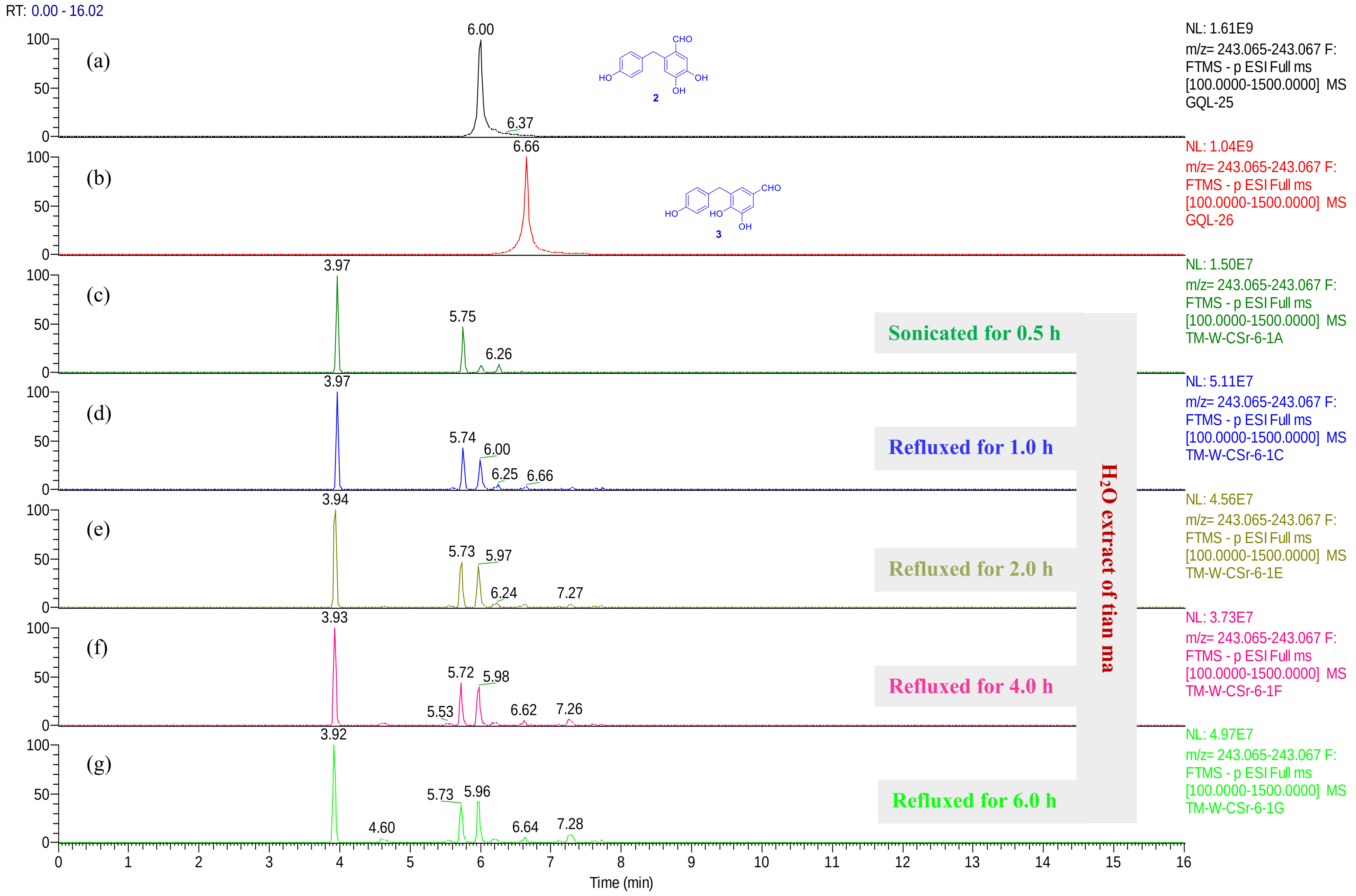

Fig. S262 Overlaid chromatograms of the extracted negative ion at *m/z* 243.066 $[M-H]^-$: (a) and (b) compounds **2** and **3** in CH_3CN, respectively; (c)–(g) extracts obtained by sonicating of "tian ma" (the steamed and dried *G. elata* rhizomes) with H_2O for 0.5 h then refluxed for 1.0 h, 2.0 h, 4.0 h, and 6.0 h, respectively.

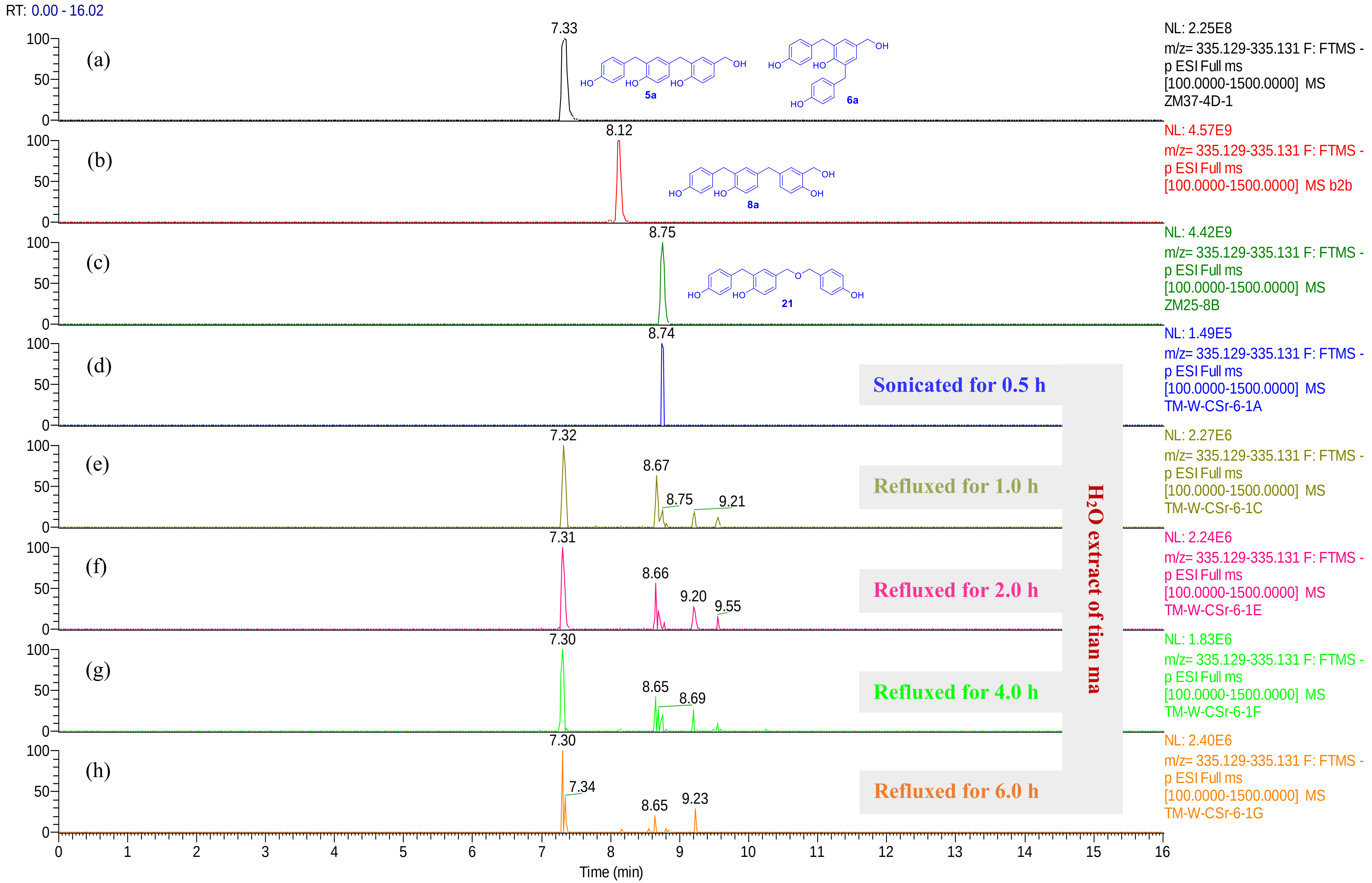

Fig. S263 Overlaid chromatograms of the extracted negative ion at *m/z* 335.130 [M−H]⁻: (a)–(c) compounds **5a**/**6a**, **8a**, and **21** in CH_3CN, respectively; (d)–(h) extracts obtained by sonicating of "tian ma" (the steamed and dried *G. elata* rhizomes) with H_2O for 0.5 h then refluxed for 1.0 h, 2.0 h, 4.0 h, and 6.0 h, respectively.

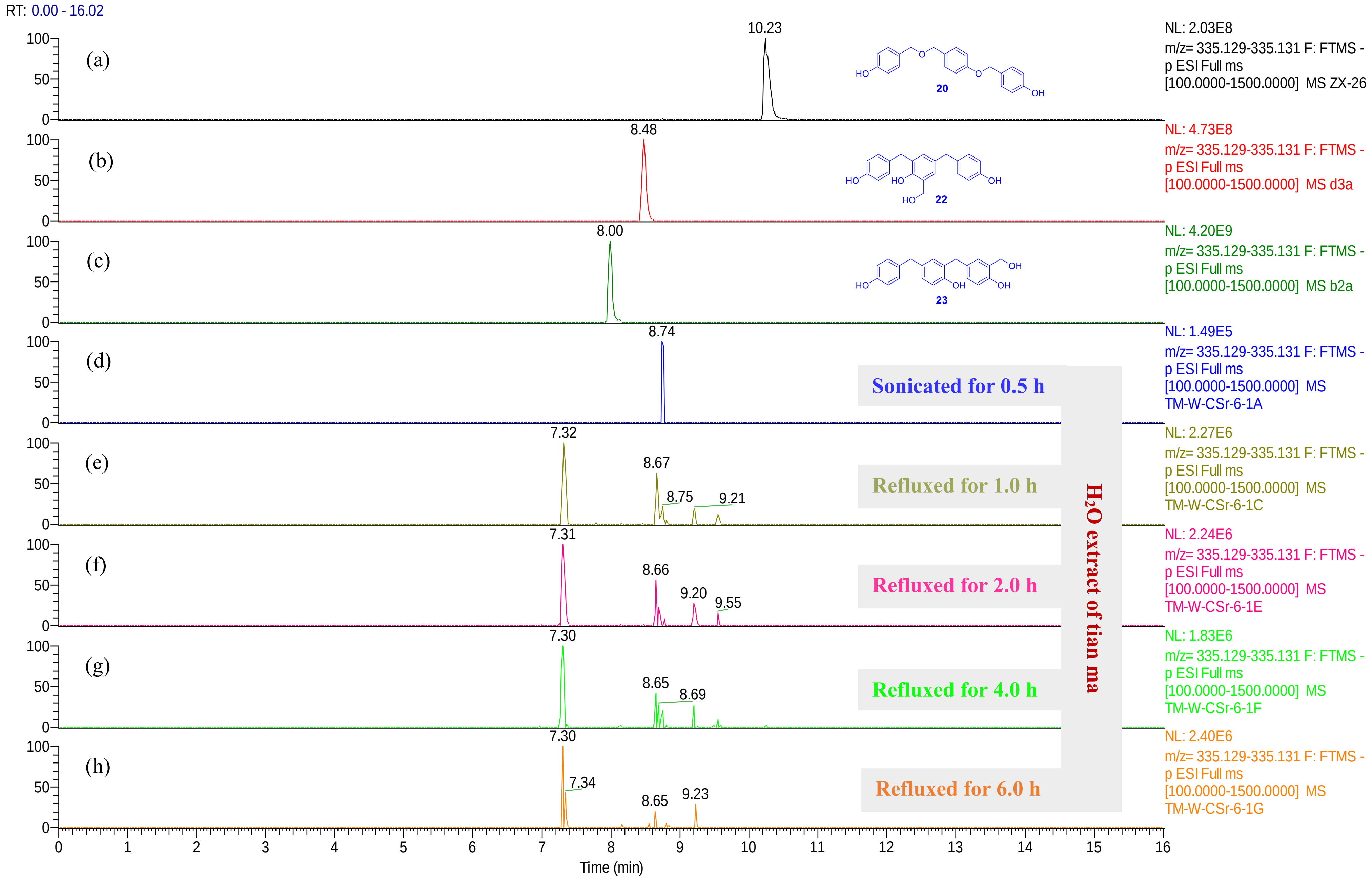

Fig. S264 Overlaid chromatograms of the extracted negative ion at *m/z* 335.130 $[M-H]^-$: (a)–(c) compounds **20**, **22**, and **23** in CH_3CN, respectively; (d)–(h) extracts obtained by sonicating of "tian ma" (the steamed and dried *G. elata* rhizomes) with H_2O for 0.5 h then refluxed for 1.0 h, 2.0 h, 4.0 h, and 6.0 h, respectively.

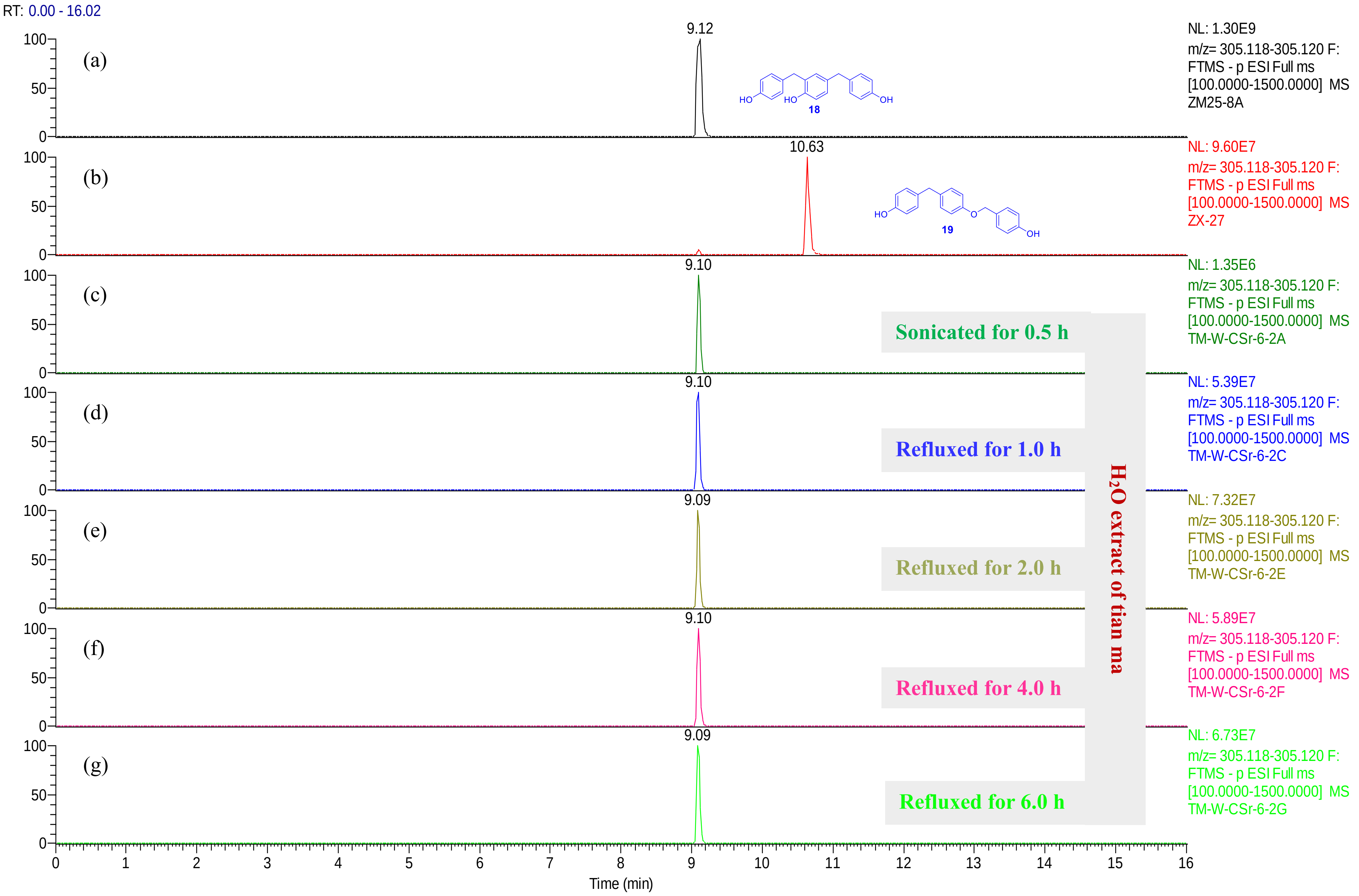

Fig. S265 Overlaid chromatograms of the extracted negative ion at *m/z* 305.119 $[M-H]^-$: (a) and (b) compounds **18** and **19** in CH_3CN, respectively; (c)–(g) extracts obtained by sonicating of "tian ma" (the steamed and dried *G. elata* rhizomes) with H_2O for 0. 5 h then refluxed for 1.0 h, 2.0 h, 4.0 h, and 6.0 h, respectively.

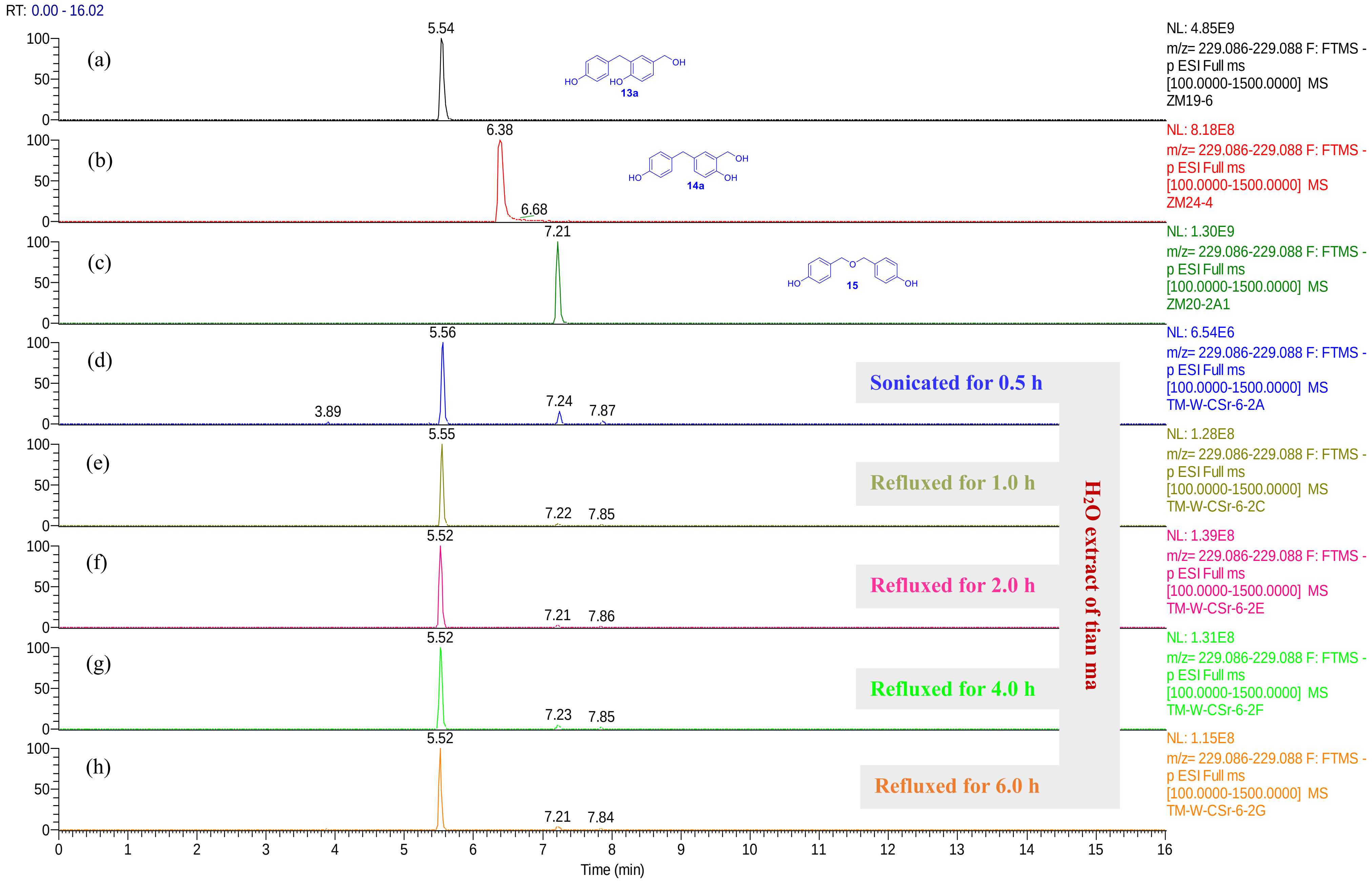

Fig. S266 Overlaid chromatograms of the extracted negative ion at *m/z* 229.087 $[M-H]^-$: (a)–(c) compounds **13a**, **14a**, and **15** in CH_3CN, respectively; (d)–(h) extracts obtained by sonicating of "tian ma" (the steamed and dried *G. elata* rhizomes) with H_2O for 0.5 h then refluxed for 1.0 h, 2.0 h, 4.0 h, and 6.0 h, respectively.

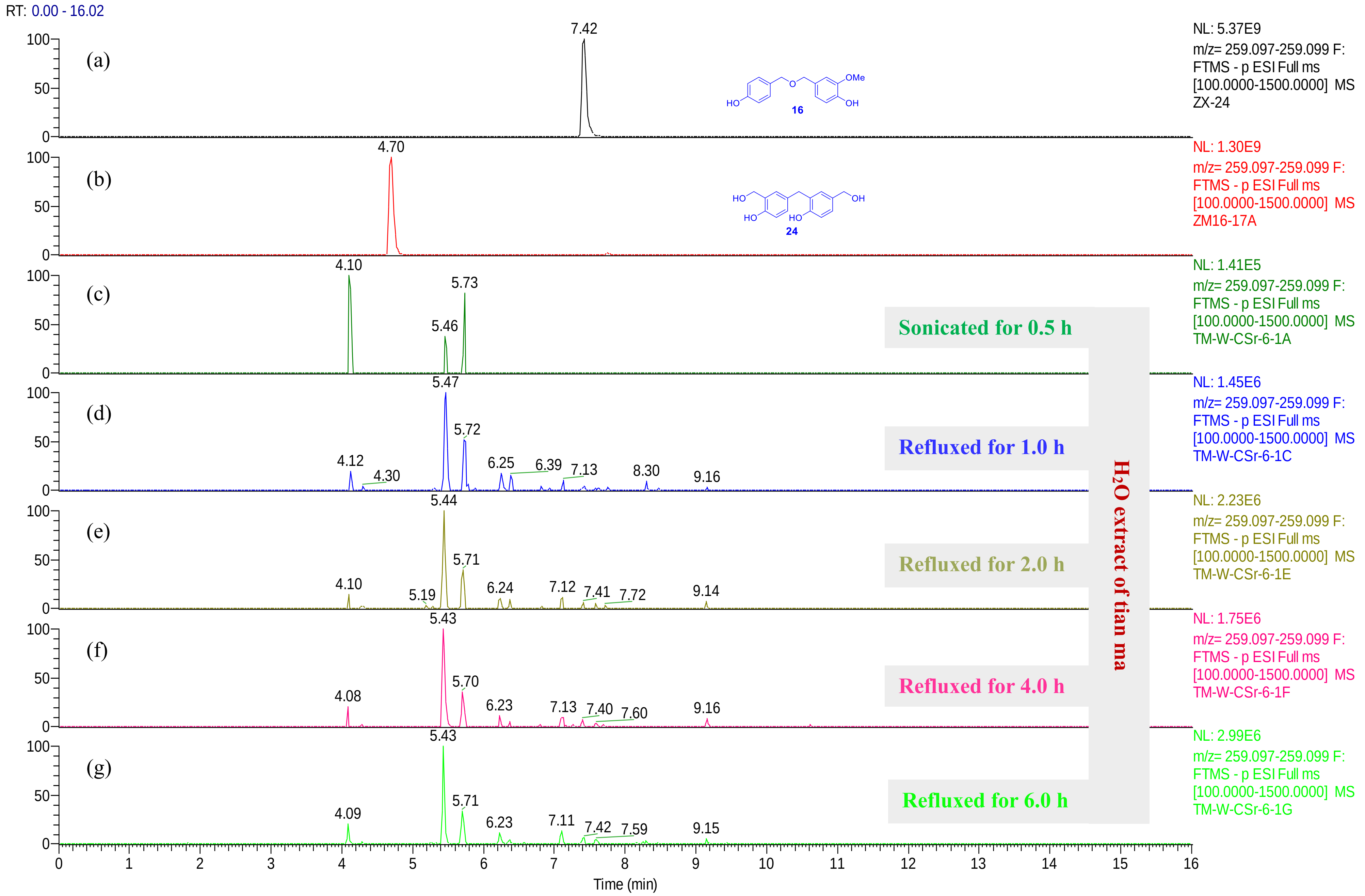

Fig. S267 Overlaid chromatograms of the extracted negative ion at *m/z* 259.098 [M–H]$^-$: (a) and (b) compounds **16** and **24** in CH_3CN, respectively; (c)–(g) extracts obtained by sonicating of "tian ma" (the steamed and dried *G. elata* rhizomes) with H_2O for 0.5 h then refluxed for 1.0 h, 2.0 h, 4.0 h, and 6.0 h, respectively.

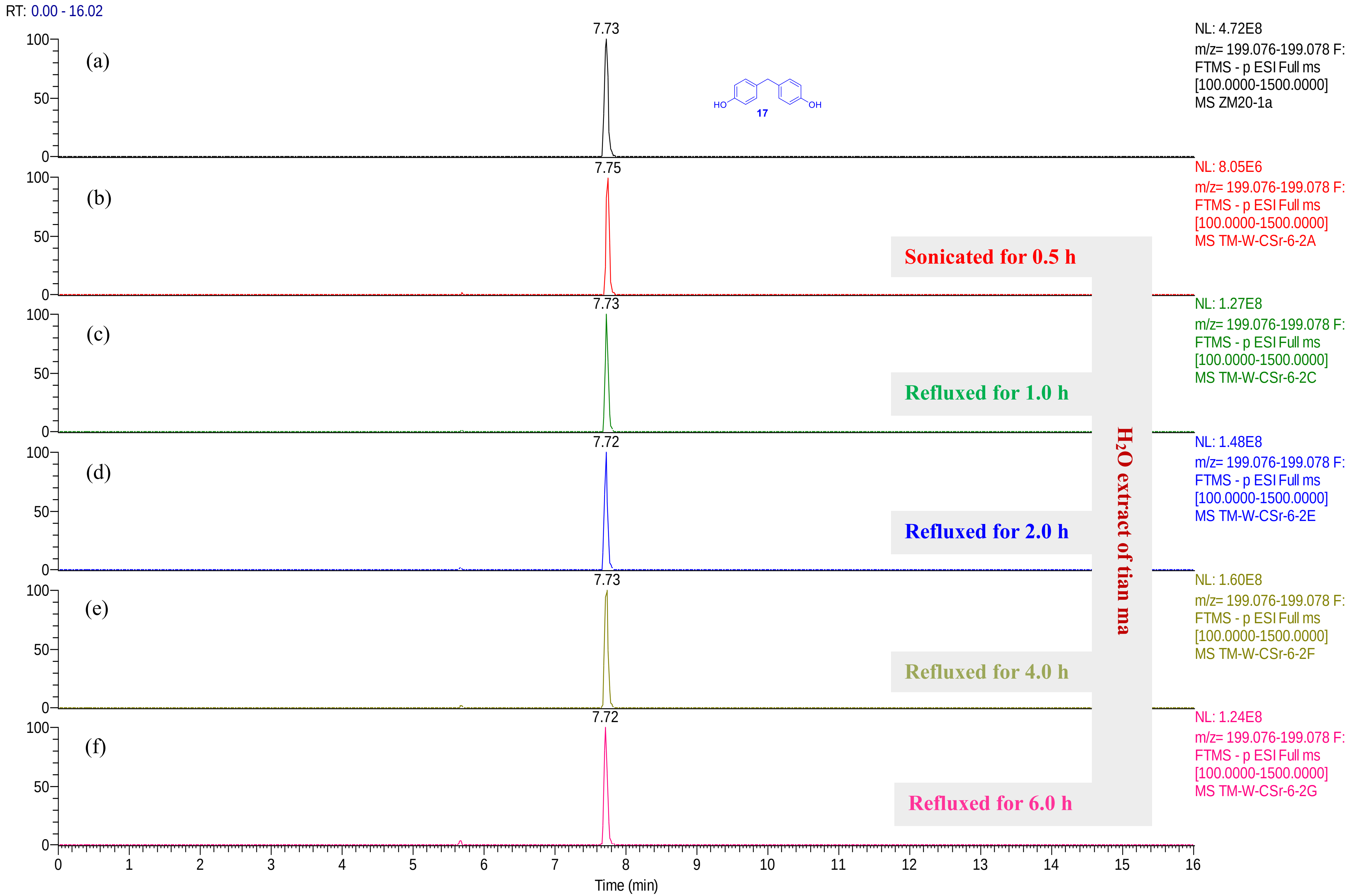

Fig. S268 Overlaid chromatograms of the extracted negative ion at *m/z* 199.077 [M–H]⁻: (a) compound **17** in CH_3CN; (b)–(f) extracts obtained by sonicating of "tian ma" (the steamed and dried *G. elata* rhizomes) with H_2O for 0. 5 h then refluxed for 1.0 h, 2.0 h, 4.0 h, and 6.0 h, respectively.

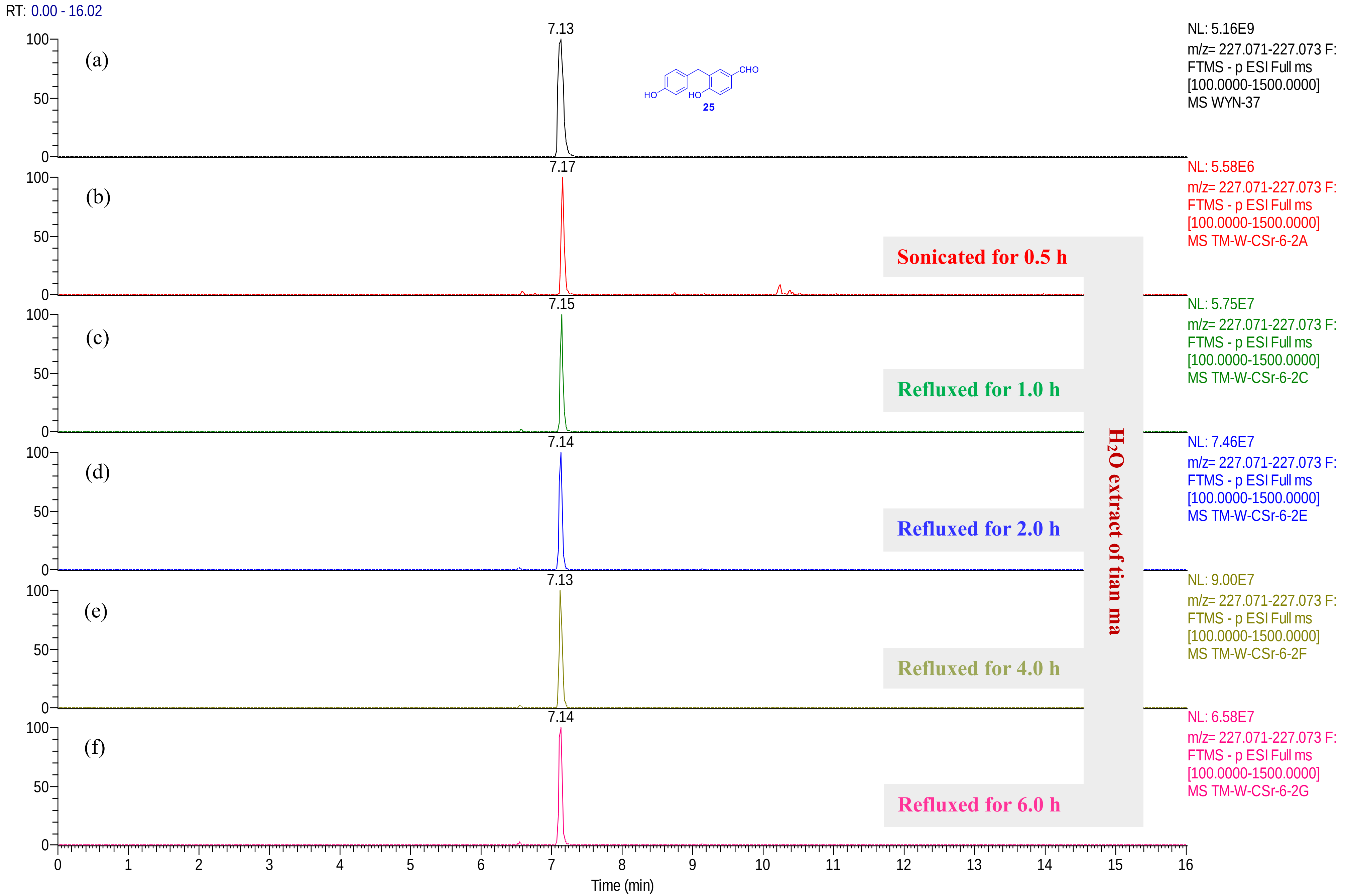

Fig. S269 Overlaid chromatograms of the extracted negative ion at *m/z* 227.072 $[M-H]^-$: (a) compound **25** in CH_3CN; (b)–(f) extracts obtained by sonicating of "tian ma" (the steamed and dried *G. elata* rhizomes) with H_2O for 0.5 h then refluxed for 1.0 h, 2.0 h, 4.0 h, and 6.0 h, respectively.

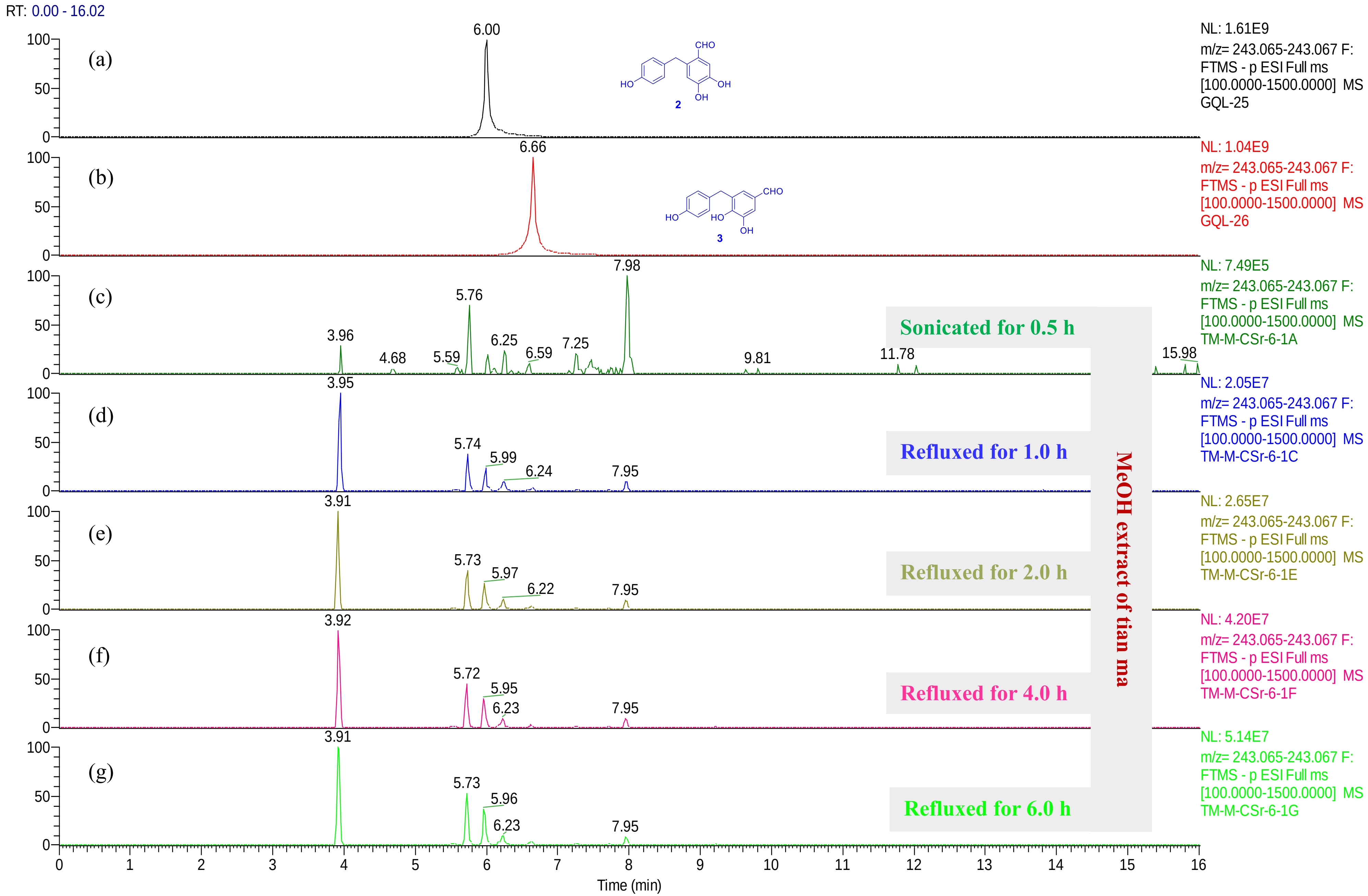

Fig. S270 Overlaid chromatograms of the extracted negative ion at *m/z* 243.066 $[M-H]^-$: (a) and (b) compounds **2** and **3** in CH_3CN, respectively; (c)–(g) extracts obtained by sonicating of "tian ma" (the steamed and dried *G. elata* rhizomes) with MeOH for 0.5 h then refluxed for 1.0 h, 2.0 h, 4.0 h, and 6.0 h, respectively.

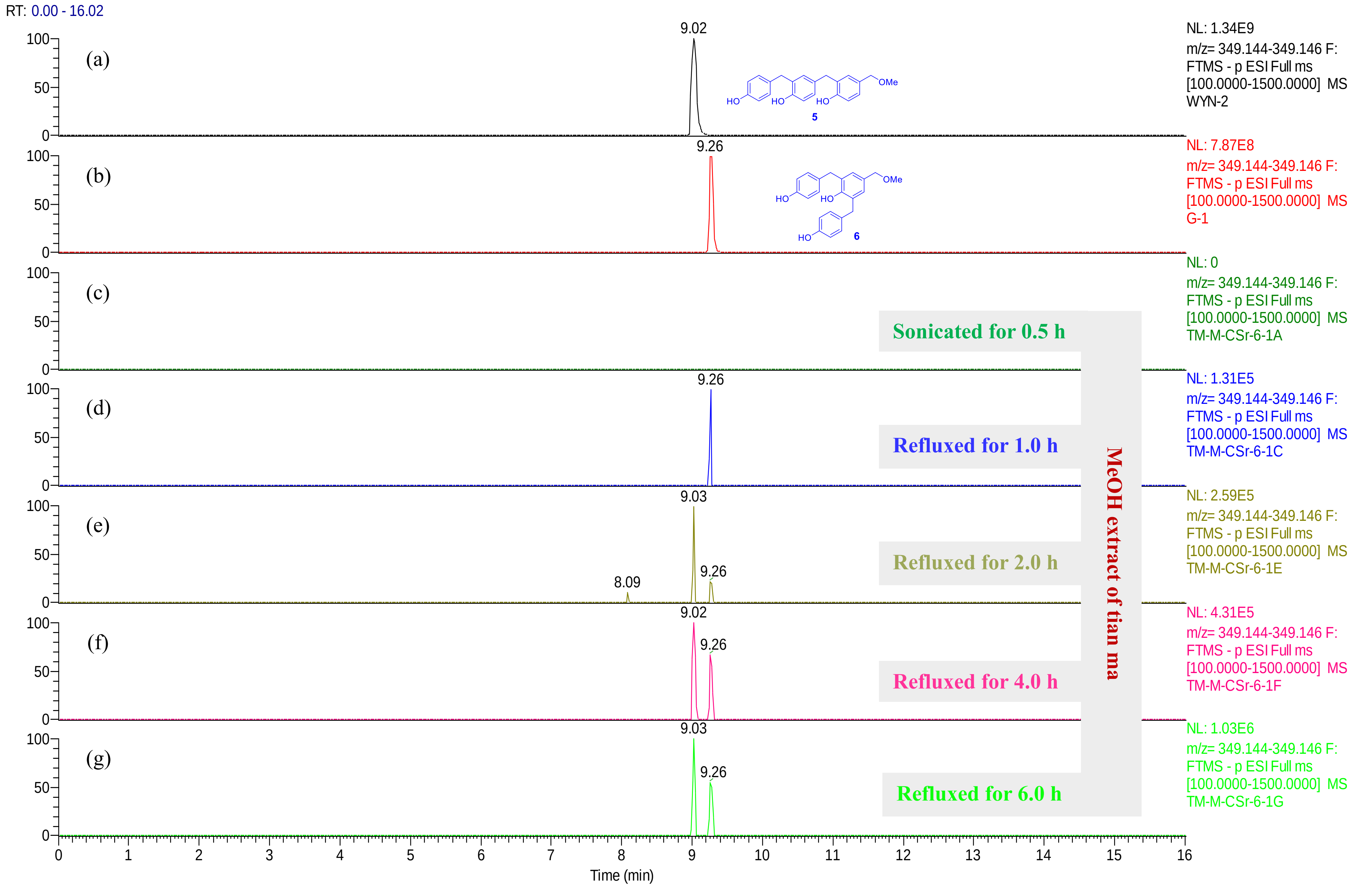

Fig. S271 Overlaid chromatograms of the extracted negative ion at *m/z* 349.145 $[M-H]^-$: (a) and (b) compounds **5** and **6** in CH_3CN, respectively; (c)–(g) extracts obtained by sonicating of "tian ma" (the steamed and dried *G. elata* rhizomes) with MeOH for 0.5 h then refluxed for 1.0 h, 2.0 h, 4.0 h, and 6.0 h, respectively.

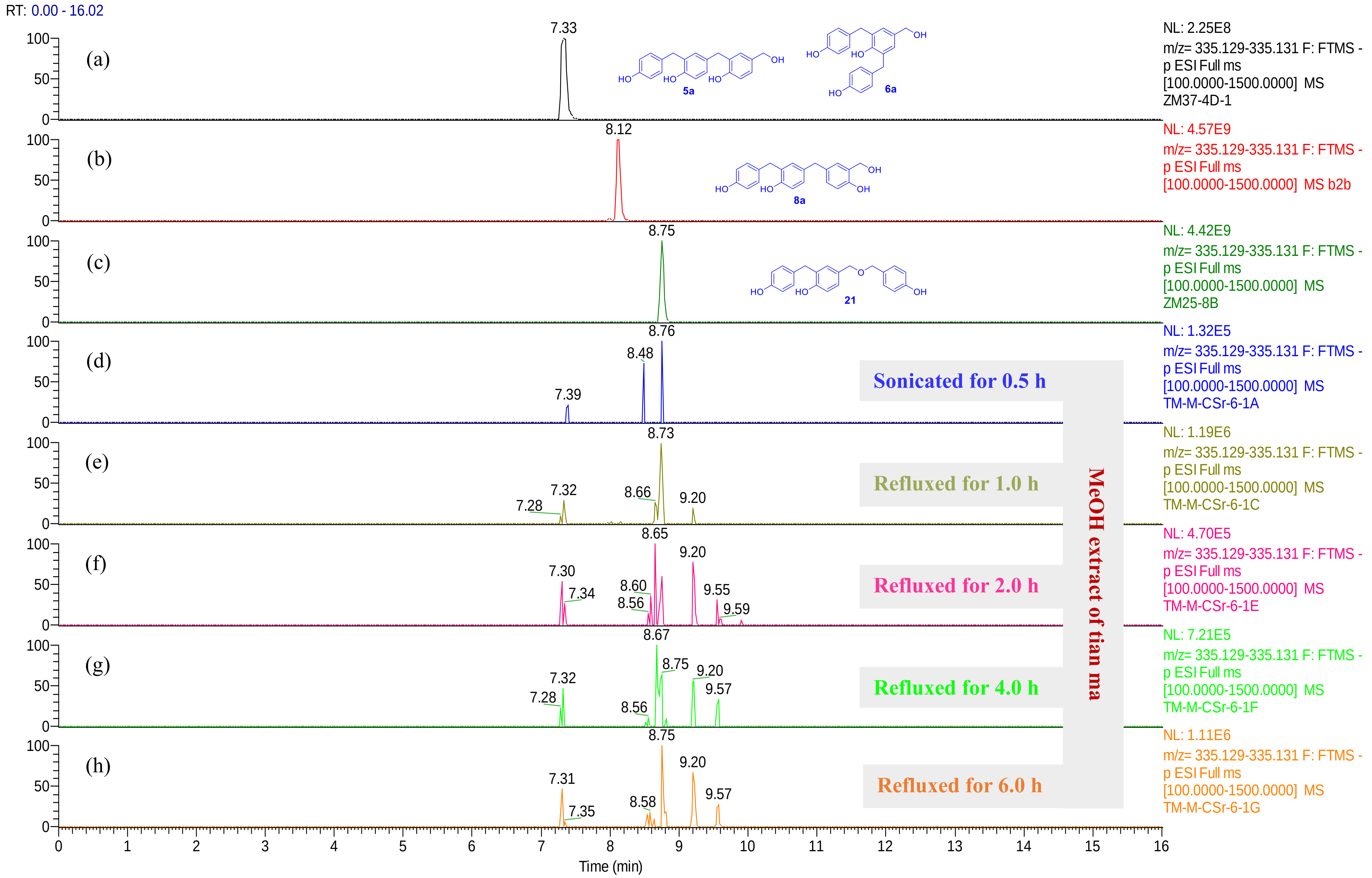

Fig. S272 Overlaid chromatograms of the extracted negative ion at *m/z* 335.130 $[M-H]^-$: (a)–(c) compounds **5a**/**6a**, **8a**, and **21** in CH_3CN, respectively; (d)–(h) extracts obtained by sonicating of “tian ma” (the steamed and dried *G. elata* rhizomes) with MeOH for 0.5 h then refluxed for 1.0 h, 2.0 h, 4.0 h, and 6.0 h, respectively.

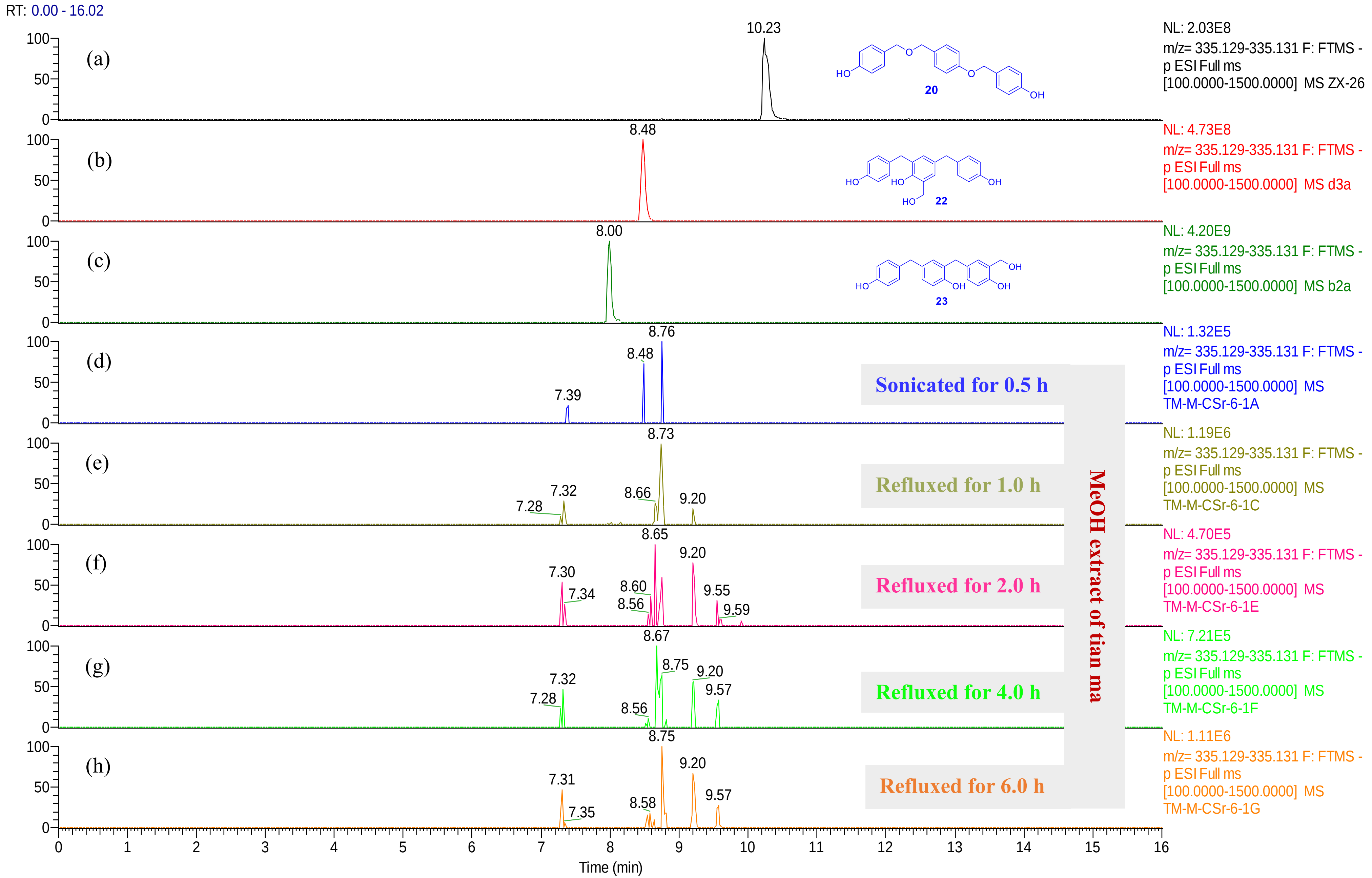

Fig. S273 Overlaid chromatograms of the extracted negative ion at *m/z* 335.130 [M–H]$^-$: (a)–(c) compounds **20**, **22**, and **23** in CH_3CN, respectively; (d)–(h) extracts obtained by sonicating of "tian ma" (the steamed and dried *G. elata* rhizomes) with MeOH for 0.5 h then refluxed for 1.0 h, 2.0 h, 4.0 h, and 6.0 h, respectively.

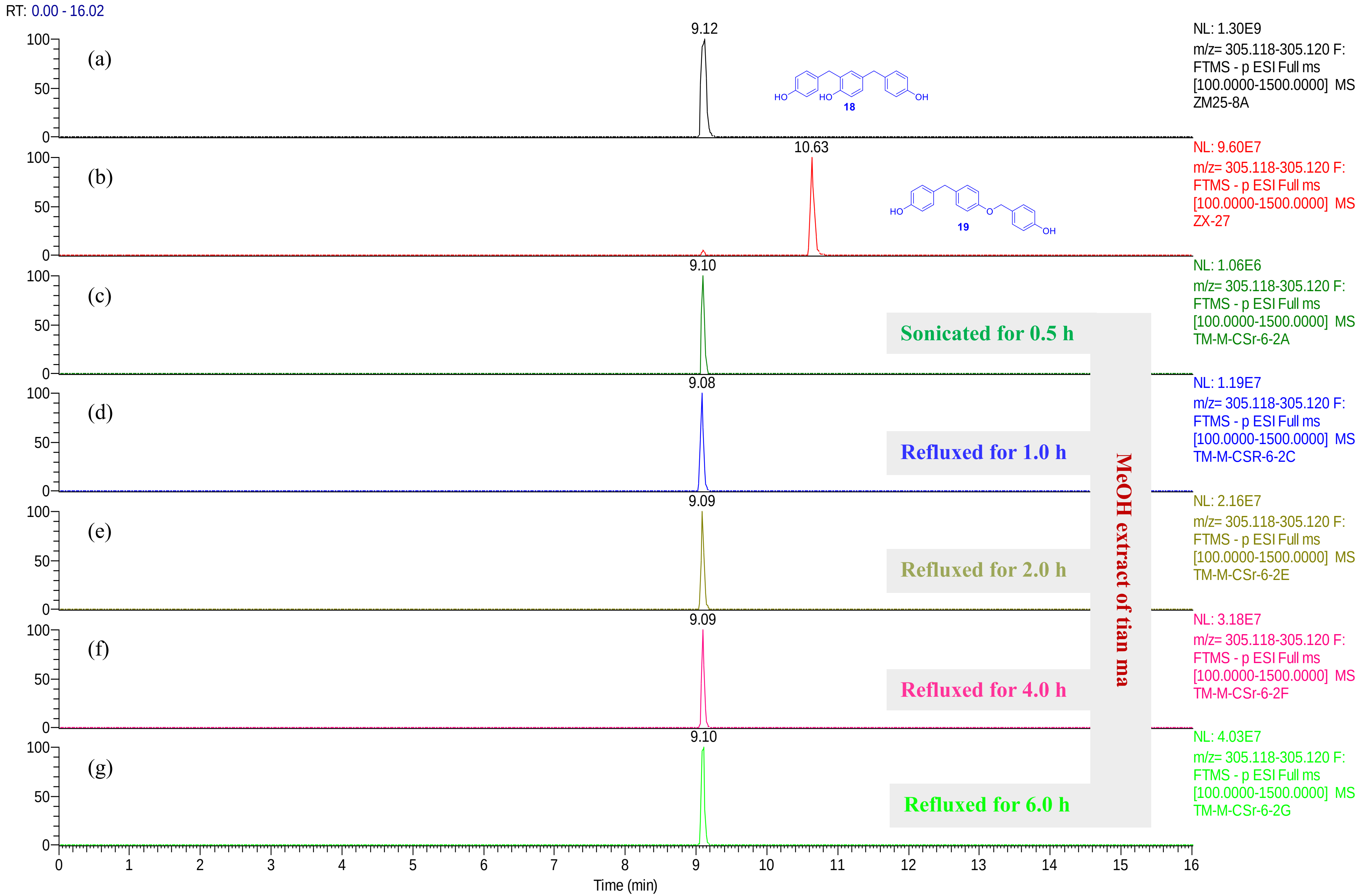

Fig. S274 Overlaid chromatograms of the extracted negative ion at *m/z* 305.119 $[M–H]^-$: (a) and (b) compounds **18** and **19** in CH_3CN, respectively; (c)–(g) extracts obtained by sonicating of "tian ma" (the steamed and dried *G. elata* rhizomes) with MeOH for 0.5 h then refluxed for 1.0 h, 2.0 h, 4.0 h, and 6.0 h, respectively.

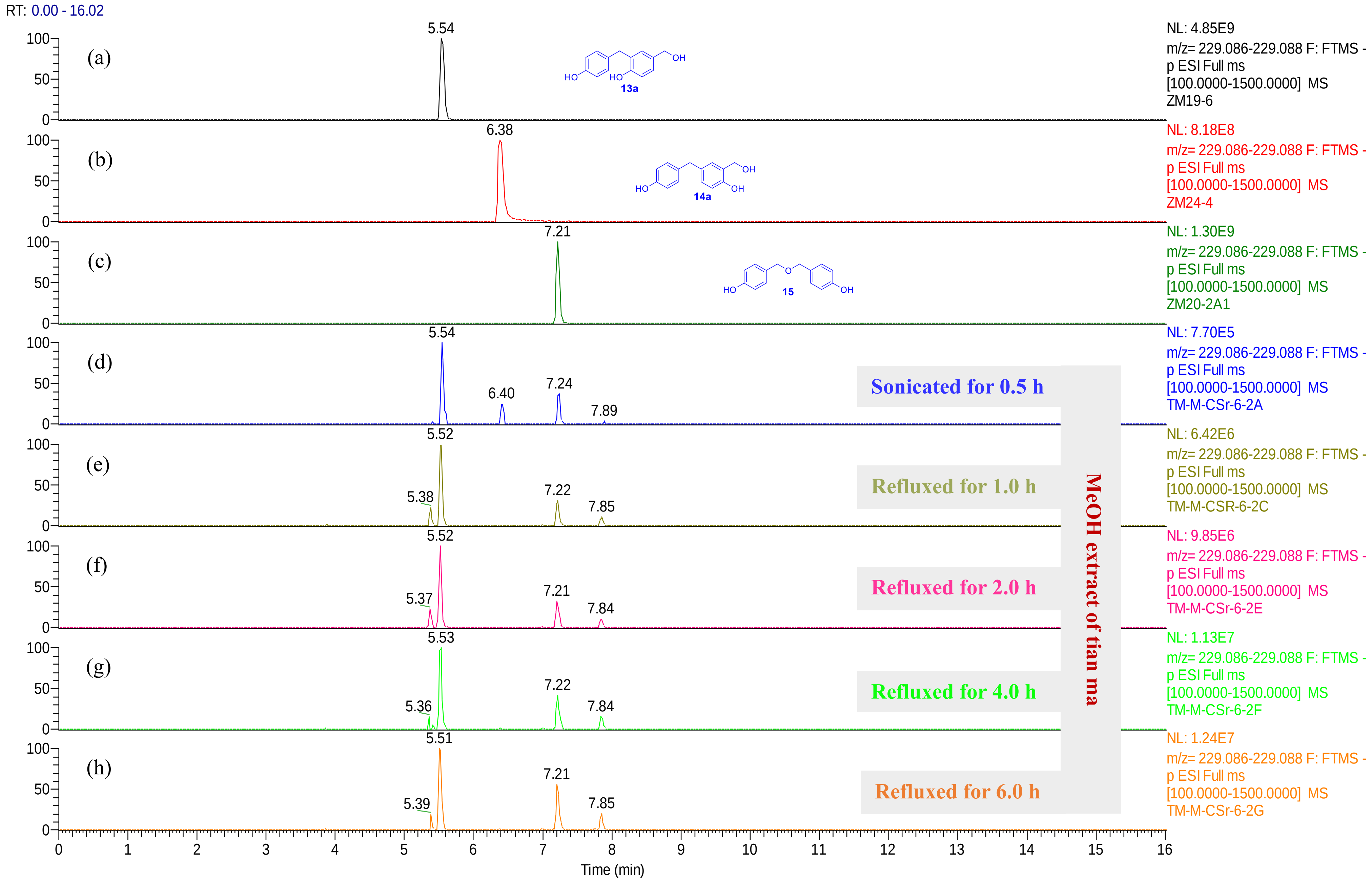

Fig. S275 Overlaid chromatograms of the extracted negative ion at *m/z* 229.087 [M−H]$^-$: (a)–(c) compounds **13a**, **14a**, and **15** in CH_3CN, respectively; (d)–(h) extracts obtained by sonicating of "tian ma" (the steamed and dried *G. elata* rhizomes) with MeOH for 0.5 h then refluxed for 1.0 h, 2.0 h, 4.0 h, and 6.0 h, respectively.

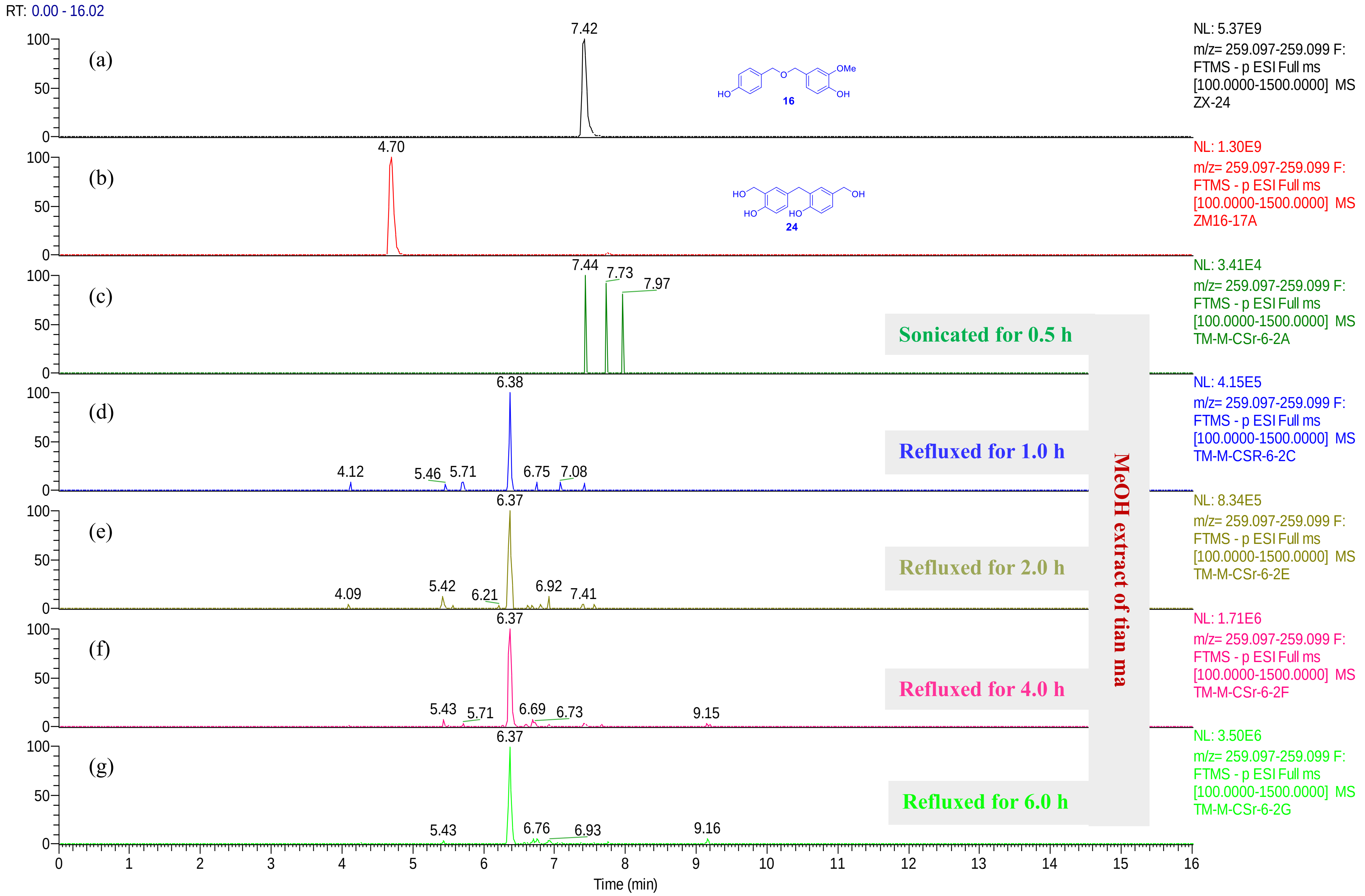

Fig. S276 Overlaid chromatograms of the extracted negative ion at *m/z* 259.098 [M–H]$^-$: (a) and (b) compounds **16** and **24** in CH_3CN, respectively; (c)–(g) extracts obtained by sonicating of "tian ma" (the steamed and dried *G. elata* rhizomes) with MeOH for 0.5 h then refluxed for 1.0 h, 2.0 h, 4.0 h, and 6.0 h, respectively.

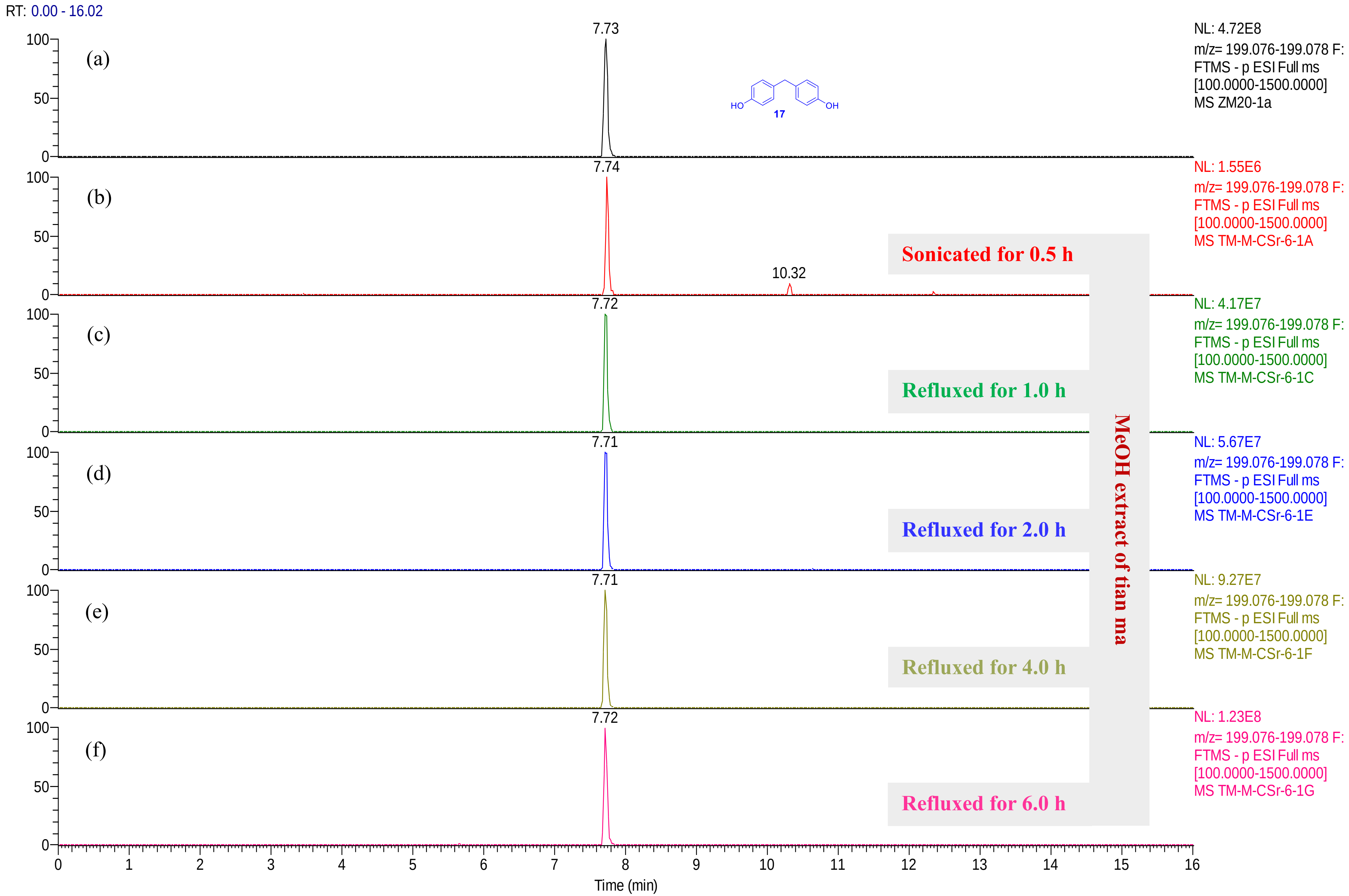

Fig. S277 Overlaid chromatograms of the extracted negative ion at *m/z* 199.077 $[M{-}H]^-$: (a) compound **17** in CH_3CN; (b)–(f) extracts obtained by sonicating of “tian ma” (the steamed and dried *G. elata* rhizomes) with MeOH for 0.5 h then refluxed for 1.0 h, 2.0 h, 4.0 h, and 6.0 h, respectively.

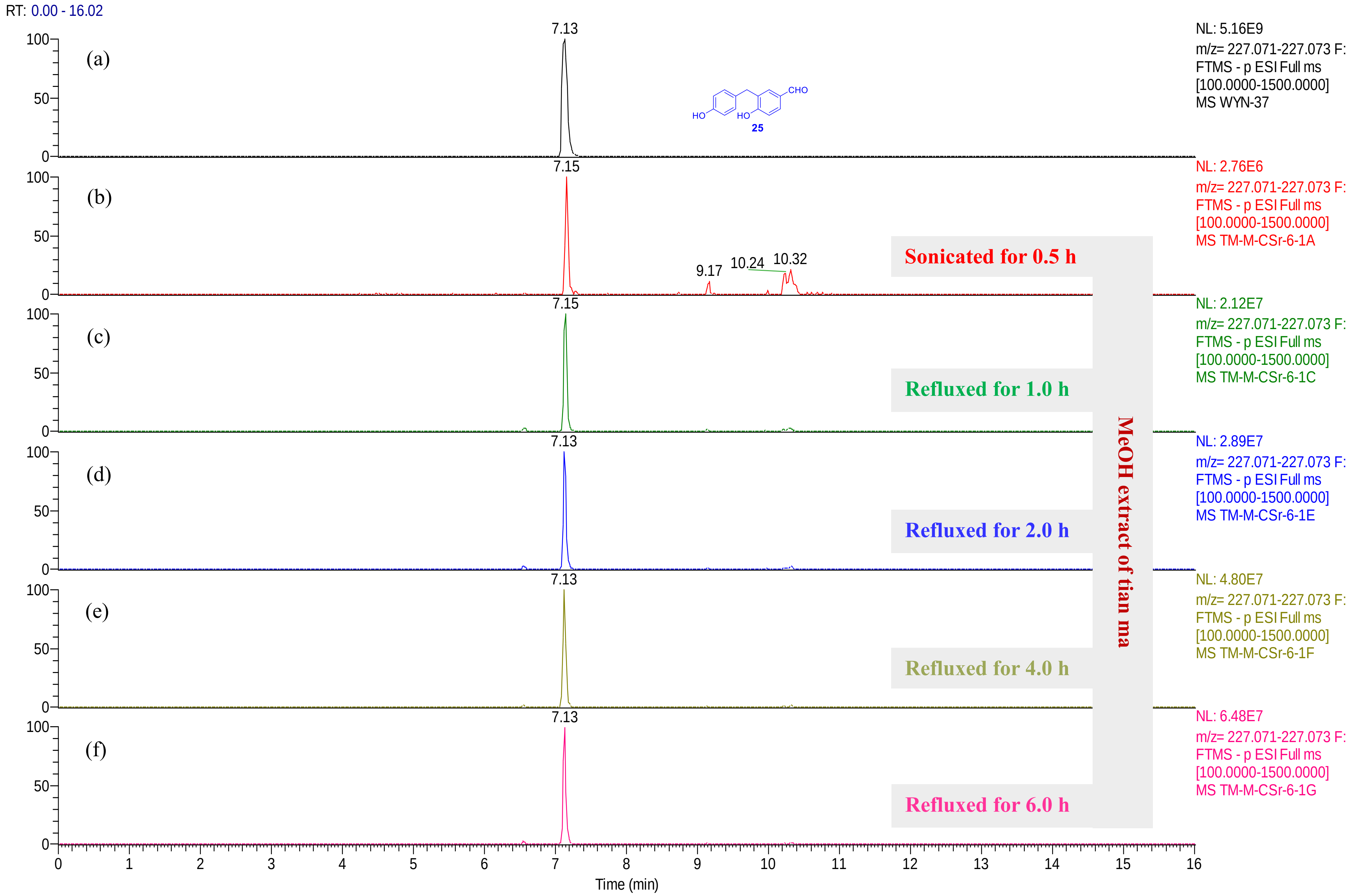

Fig. S278 Overlaid chromatograms of the extracted negative ion at *m/z* 227.072 $[M-H]^-$: (a) compound **25** in CH_3CN; (b)–(f) extracts obtained by sonicating of "tian ma" (the steamed and dried *G. elata* rhizomes) with MeOH for 0.5 h then refluxed for 1.0 h, 2.0 h, 4.0 h, and 6.0 h, respectively.

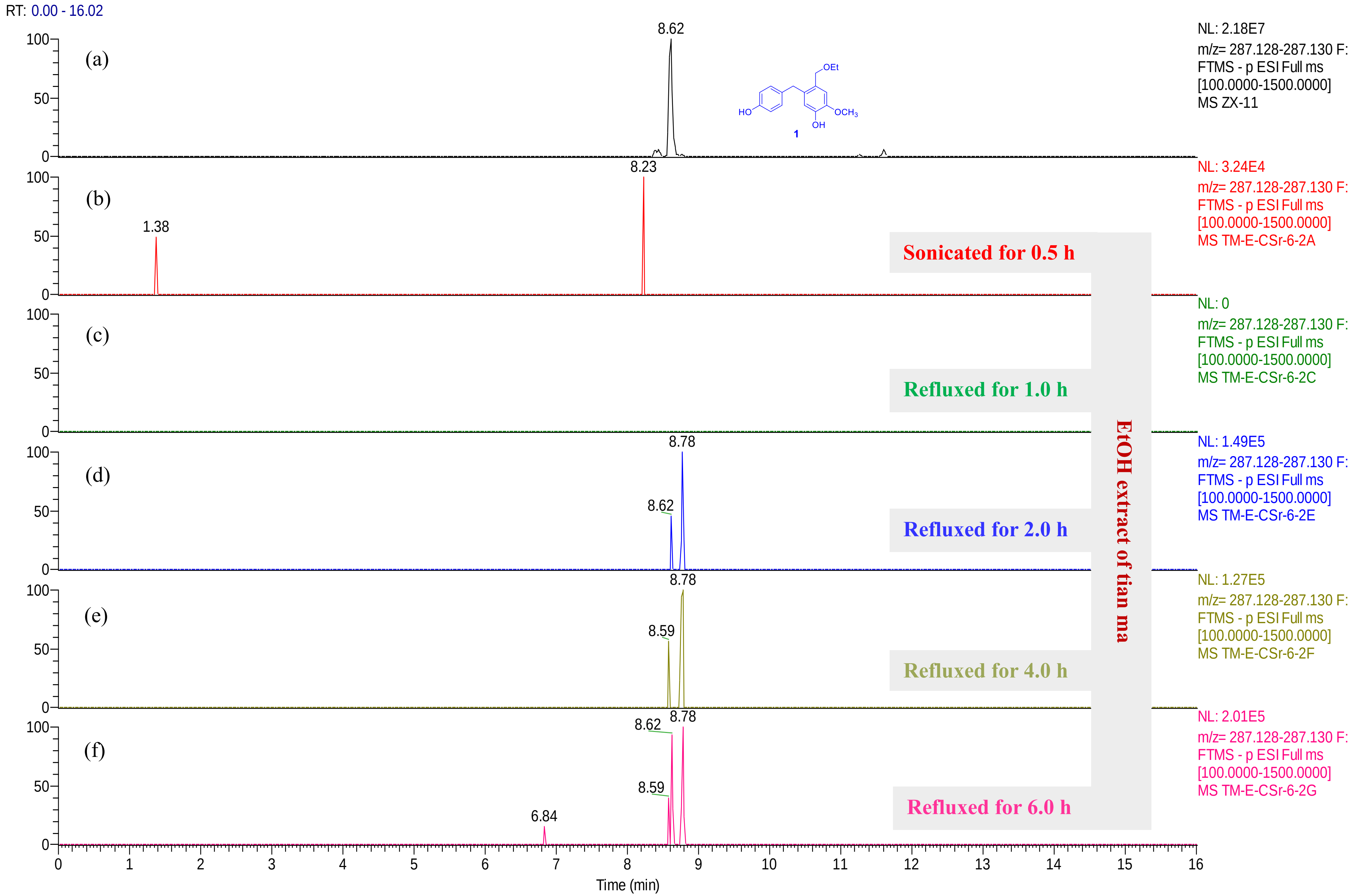

Fig. S279 Overlaid chromatograms of the extracted negative ion at *m/z* 287.129 $[M-H]^-$: (a) compound **1** in CH_3CN; (b)–(f) extracts obtained by sonicating of "tian ma" (the steamed and dried *G. elata* rhizomes) with EtOH for 0.5 h then refluxed for 1.0 h, 2.0 h, 4.0 h, and 6.0 h, respectively.

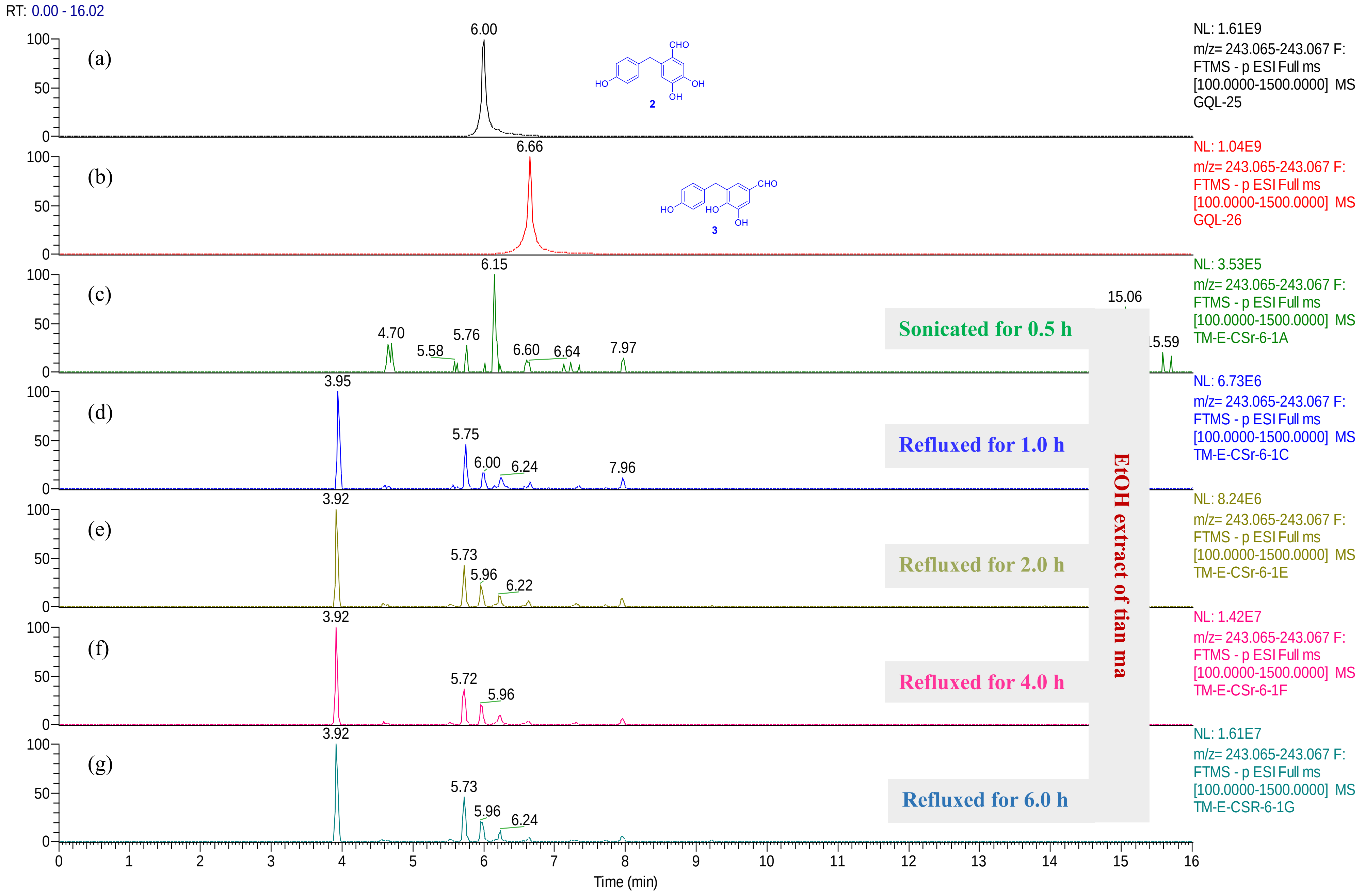

Fig. S280 Overlaid chromatograms of the extracted negative ion at *m/z* 243.066 $[M-H]^-$: (a) and (b) compounds **2** and **3** in CH_3CN, respectively; (c)–(g) extracts obtained by sonicating of "tian ma" (the steamed and dried *G. elata* rhizomes) with EtOH for 0.5 h then refluxed for 1.0 h, 2.0 h, 4.0 h, and 6.0 h, respectively.

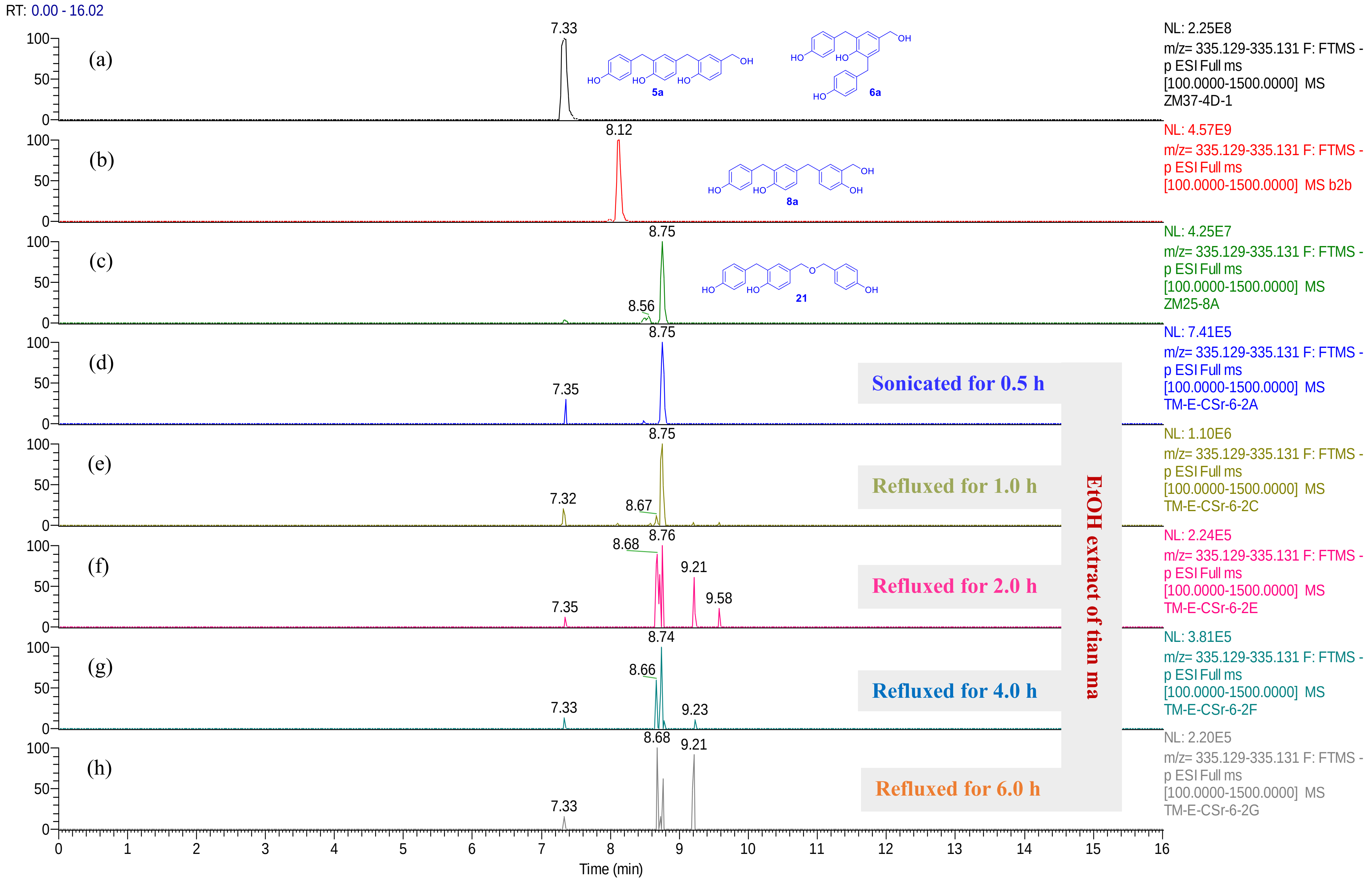

Fig. S281 Overlaid chromatograms of the extracted negative ion at *m/z* 335.130 [M−H]⁻: (a)–(c) compounds **5a**/**6a**, **8a**, and **21** in CH_3CN, respectively; (d)–(h) extracts obtained by sonicating of "tian ma" (the steamed and dried *G. elata* rhizomes) with EtOH for 0.5 h then refluxed for 1.0 h, 2.0 h, 4.0 h, and 6.0 h, respectively.

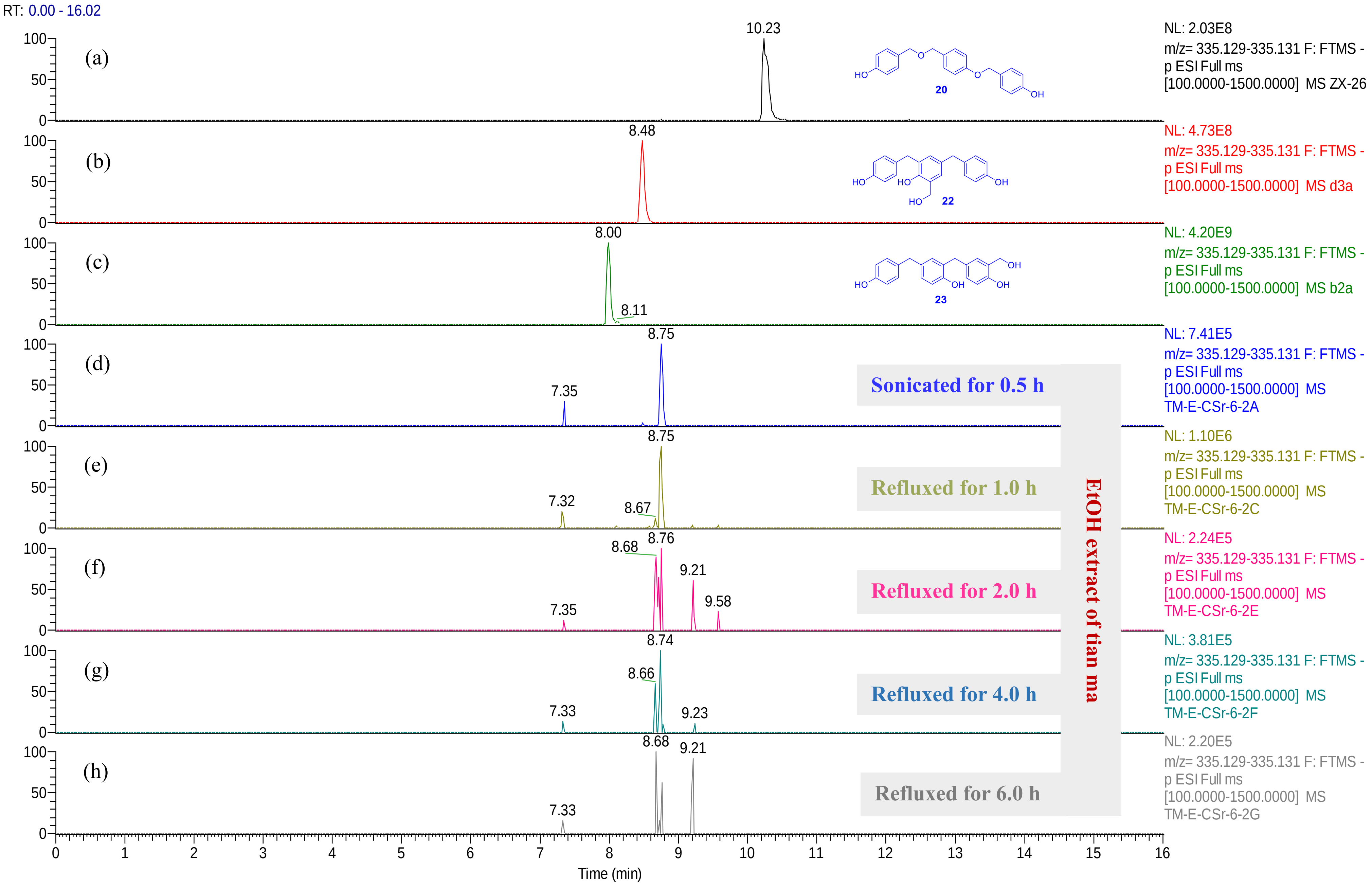

Fig. S282 Overlaid chromatograms of the extracted negative ion at *m/z* 335.130 [M–H]⁻: (a)–(c) compounds **20**, **22**, and **23** in CH_3CN, respectively; (d)–(h) extracts obtained by sonicating of "tian ma" (the steamed and dried *G. elata* rhizomes) with EtOH for 0.5 h then refluxed for 1.0 h, 2.0 h, 4.0 h, and 6.0 h, respectively.

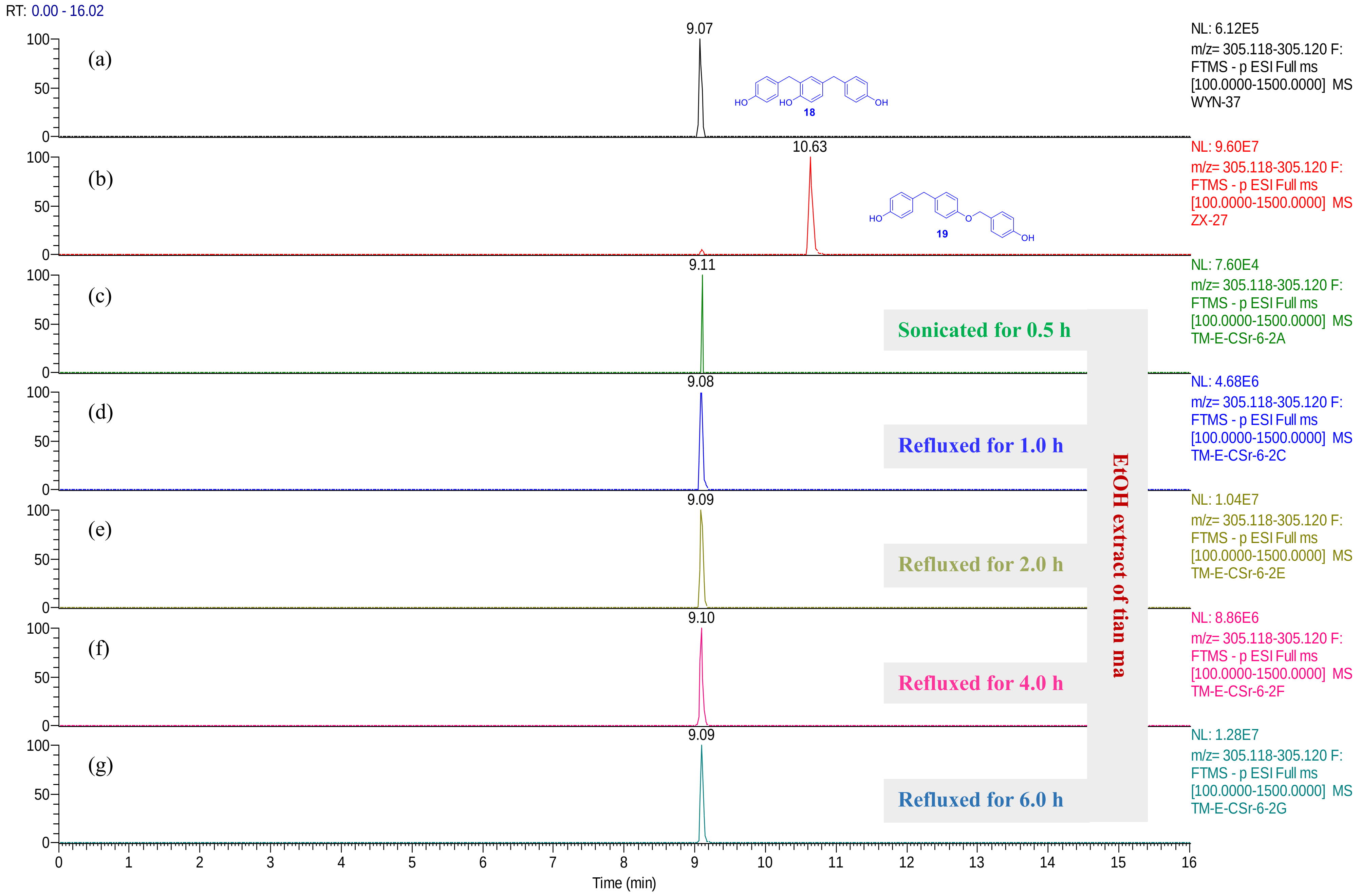

Fig. S283 Overlaid chromatograms of the extracted negative ion at *m/z* 305.119 $[M-H]^-$: (a) and (b) compounds **18** and **19** in CH_3CN, respectively; (c)−(g) extracts obtained by sonicating of "tian ma" (the steamed and dried *G. elata* rhizomes) with EtOH for 0.5 h then refluxed for 1.0 h, 2.0 h, 4.0 h, and 6.0 h, respectively.

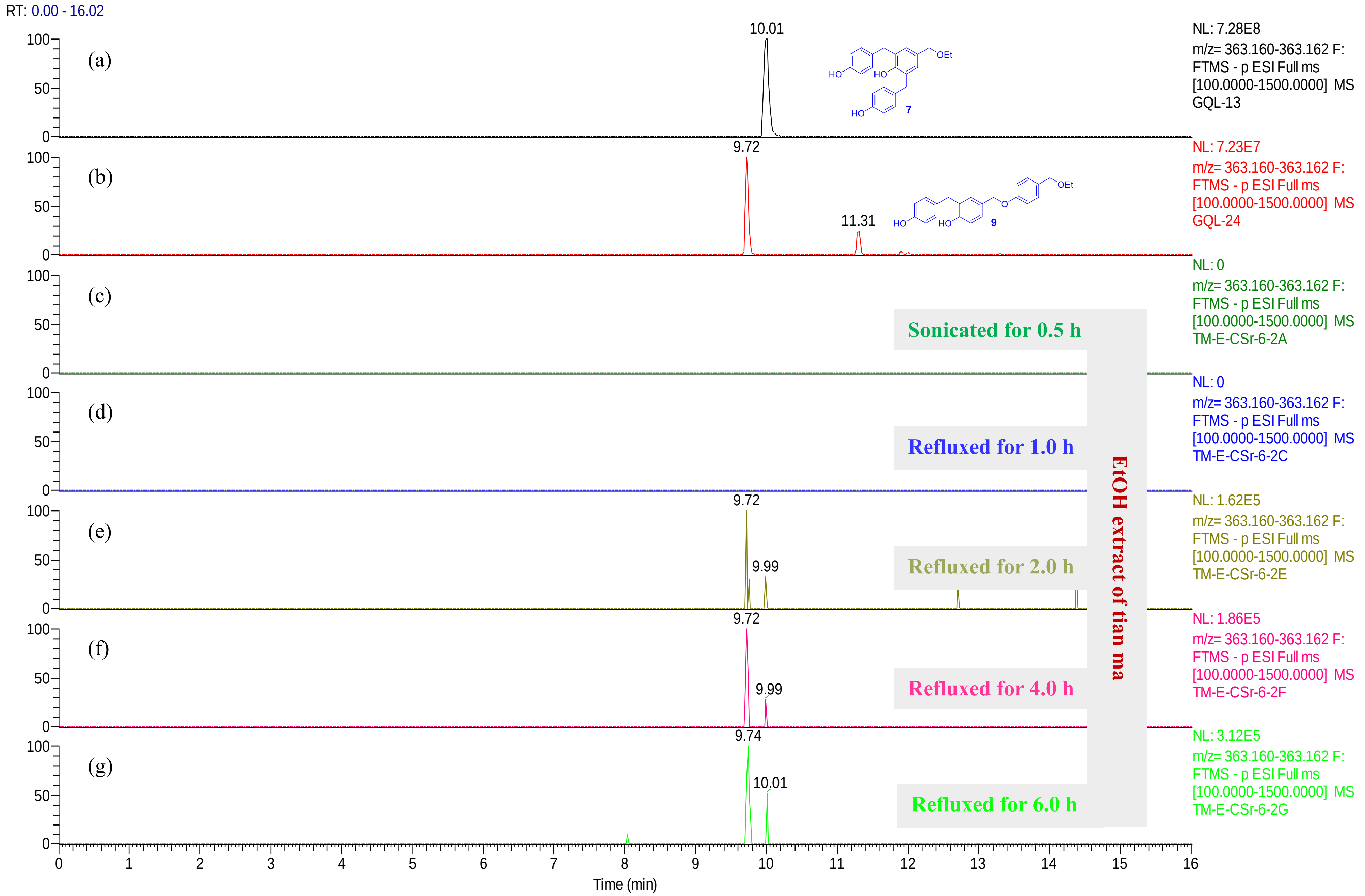

Fig. S284 Overlaid chromatograms of the extracted negative ion at *m/z* 363.161 $[M-H]^-$: (a) and (b) compounds **7** and **9** in CH_3CN, respectively; (c)–(g) extracts obtained by sonicating of "tian ma" (the steamed and dried *G. elata* rhizomes) with EtOH for 0.5 h then refluxed for 1.0 h, 2.0 h, 4.0 h, and 6.0 h, respectively.

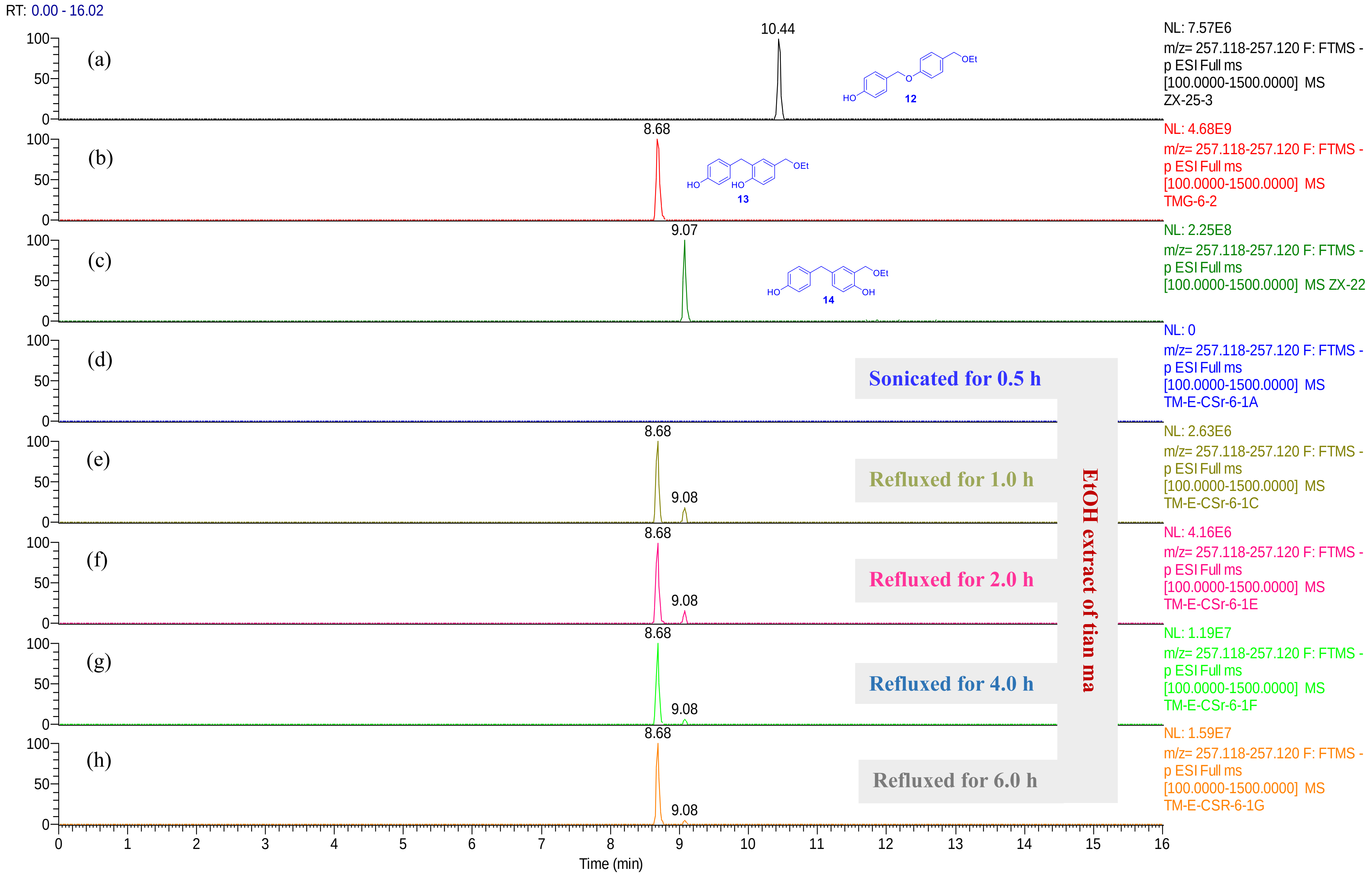

Fig. S285 Overlaid chromatograms of the extracted negative ion at *m/z* 257.119 [M–H]⁻: (a) – (c) compounds **12**, **13**, and **14** in CH_3CN, respectively; (d)–(h) extracts obtained by sonicating of "tian ma" (the steamed and dried *G. elata* rhizomes) with EtOH for 0.5 h then refluxed for 1.0 h, 2.0 h, 4.0 h, and 6.0 h, respectively.

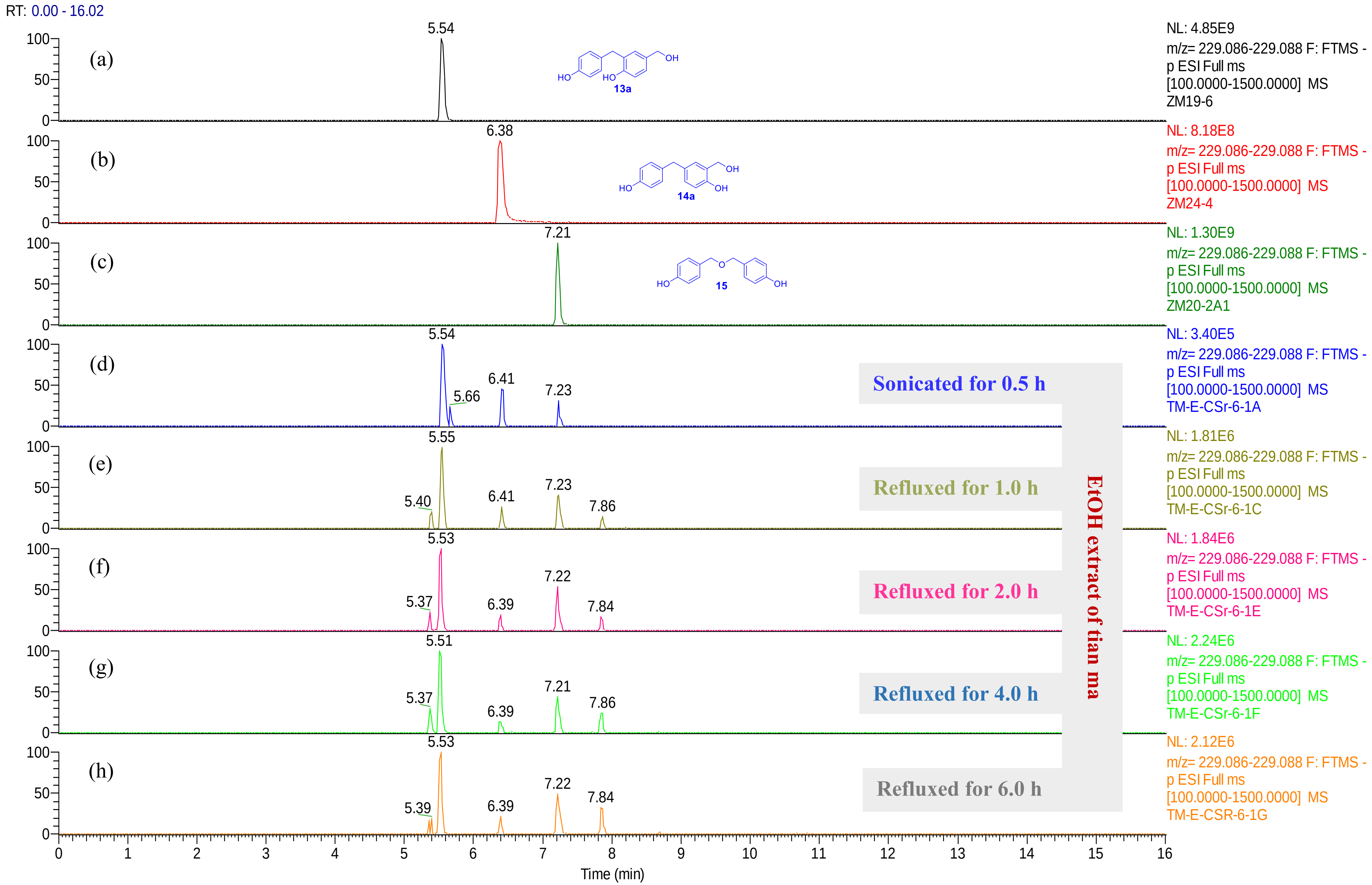

Fig. S286 Overlaid chromatograms of the extracted negative ion at *m/z* 229.087 $[M-H]^-$: (a)–(c) compounds **13a**, **14a**, and **15** in CH_3CN, respectively; (d)–(h) extracts obtained by sonicating of "tian ma" (the steamed and dried *G. elata* rhizomes) with EtOH for 0.5 h then refluxed for 1.0 h, 2.0 h, 4.0 h, and 6.0 h, respectively.

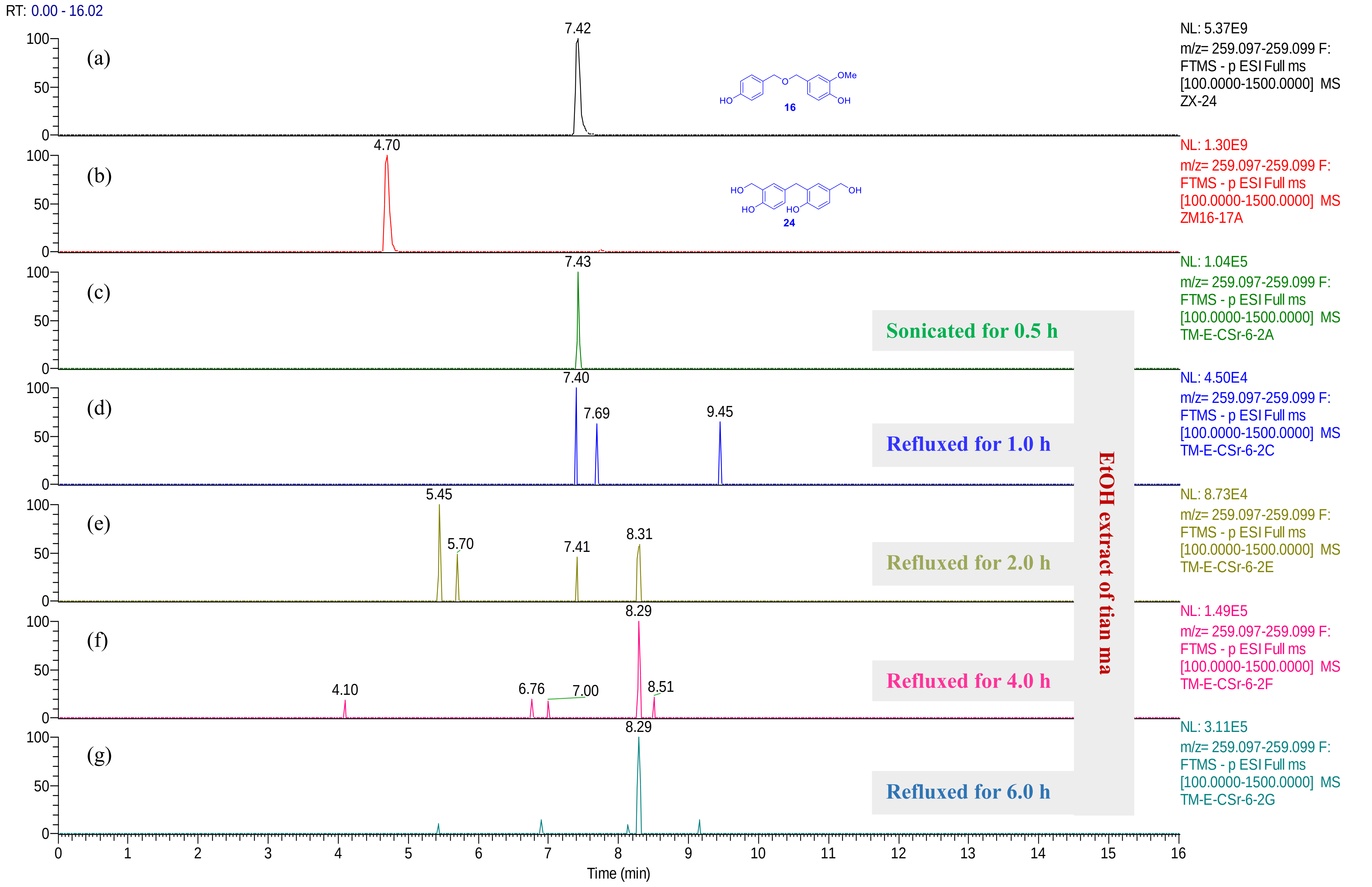

Fig. S287 Overlaid chromatograms of the extracted negative ion at *m/z* 259.098 $[M-H]^-$: (a) and (b) compounds **16** and **24** in CH_3CN, respectively; (c)–(g) extracts obtained by sonicating of "tian ma" (the steamed and dried *G. elata* rhizomes) with EtOH for 0.5 h then refluxed for 1.0 h, 2.0 h, 4.0 h, and 6.0 h, respectively.

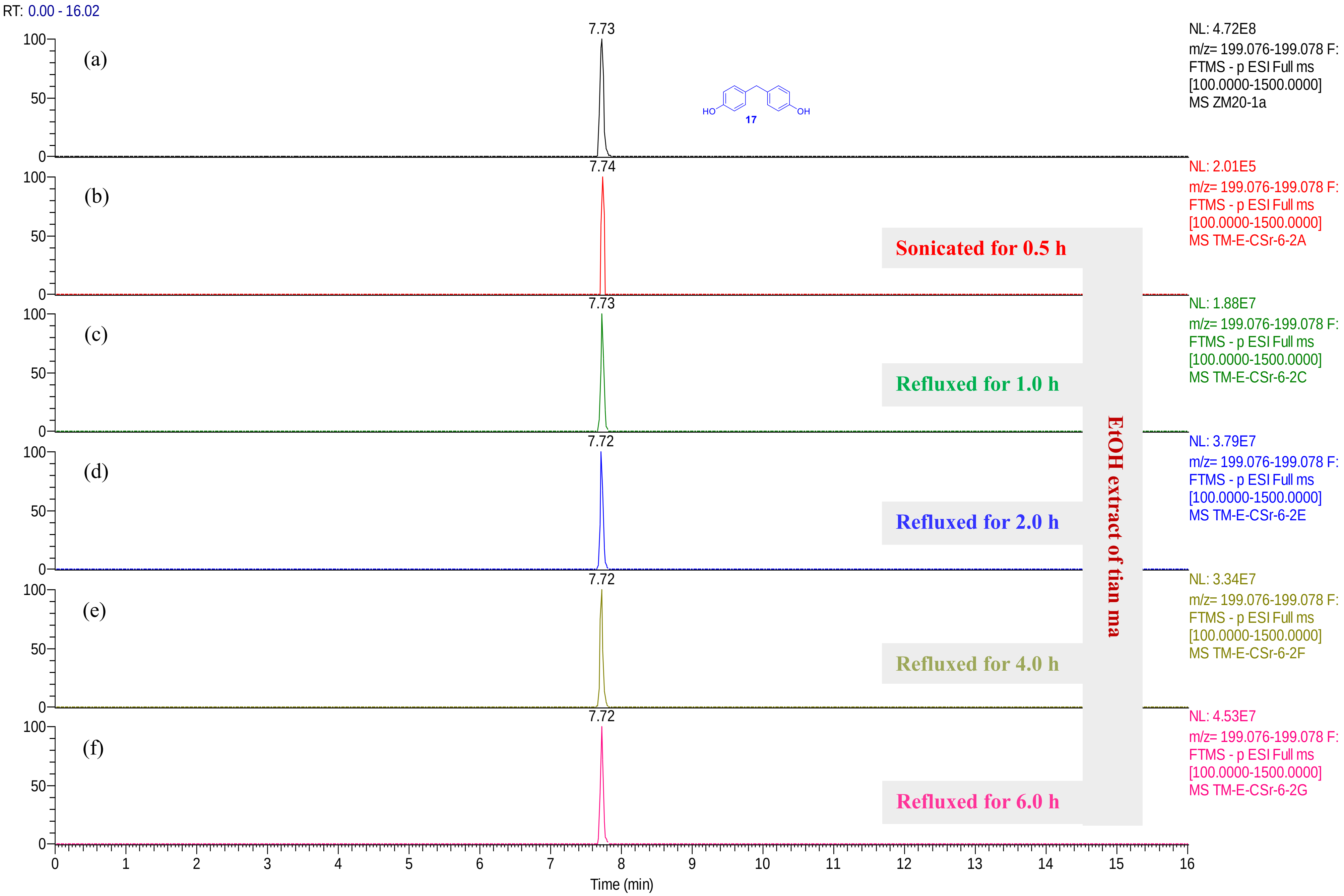

Fig. S288 Overlaid chromatograms of the extracted negative ion at *m/z* 199.077 [M–H]⁻: (a) compound **17** in CH_3CN; (b)–(f) extracts obtained by sonicating of "tian ma" (the steamed and dried *G. elata* rhizomes) with EtOH for 0.5 h then refluxed for 1.0 h, 2.0 h, 4.0 h, and 6.0 h, respectively.

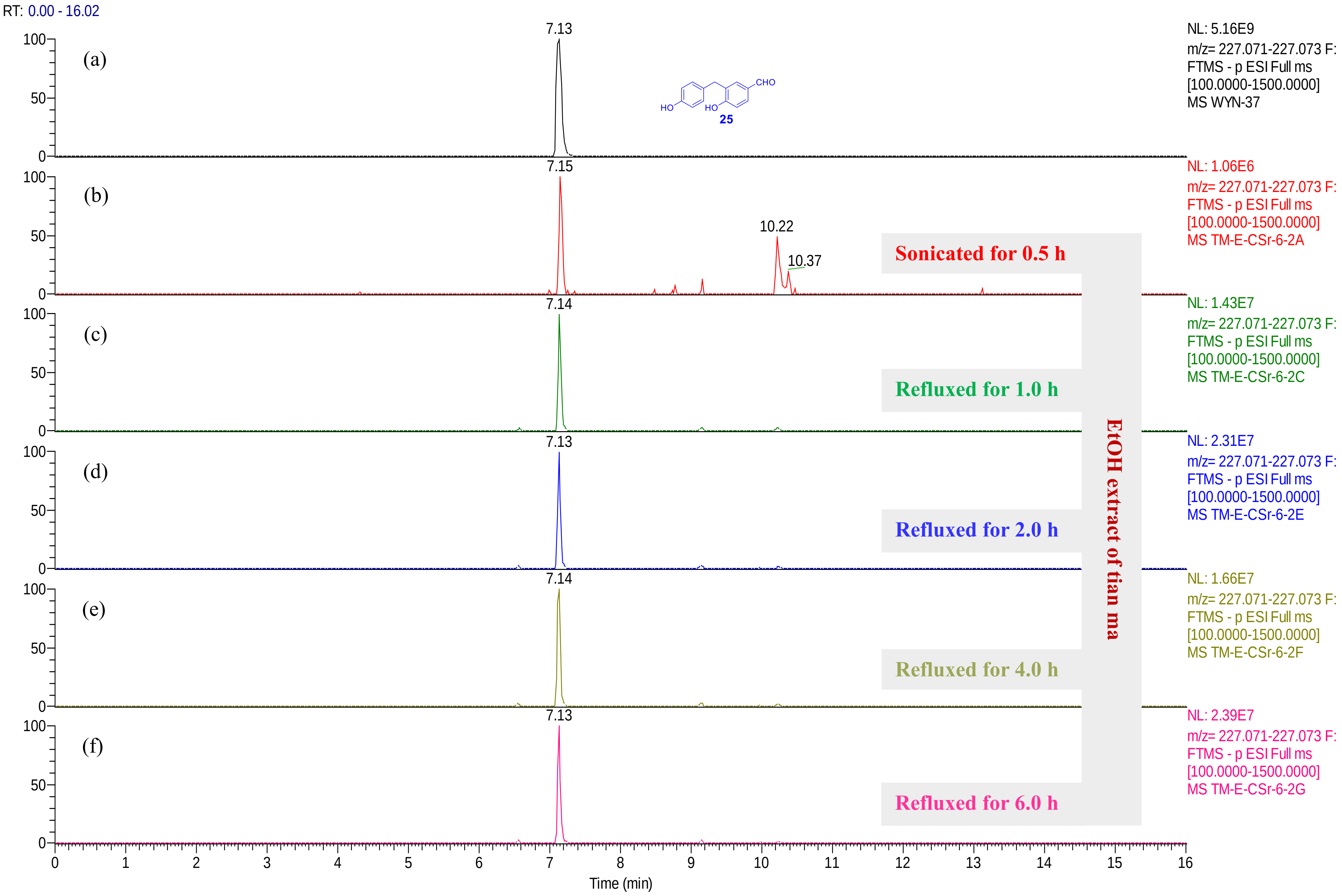

Fig. S289 Overlaid chromatograms of the extracted negative ion at *m/z* 227.072 [M−H]$^-$: (a) compound **25** in CH_3CN; (b)–(f) extracts obtained by sonicating of "tian ma" (the steamed and dried *G. elata* rhizomes) with EtOH for 0.5 h then refluxed for 1.0 h, 2.0 h, 4.0 h, and 6.0 h, respectively.
